# Incidence and prevalence of asthma, chronic obstructive pulmonary disease and interstitial lung disease between 2004 and 2023: harmonised analyses of longitudinal cohorts across England, Wales, South-East Scotland and Northern Ireland

**DOI:** 10.1136/thorax-2024-222699

**Published:** 2025-04-08

**Authors:** Hannah Whittaker, Adriana Kramer Fiala Machado, Sara Hatam, Sarah Cook, Sean Scully, Hywel Turner T Evans, Thomas Bolton, Constantinos Kallis, John Busby, Liam G Heaney, Aziz Sheikh, Jennifer K Quint, on behalf of the CVD-COVID-UK/COVID-IMPACT Consortium, Alastair Proudfoot

**Affiliations:** 1 School of Public Health, Imperial College London, London, UK; 2 Queen’s University Belfast, Belfast, UK; 3 The University of Edinburgh Usher Institute of Population Health Sciences and Informatics, Edinburgh, UK; 4 Imperial College London School of Public Health, London, UK; 5 Swansea University, Swansea, UK; 6 Population Data Science, Swansea University, Swansea, UK; 7 British Heart Foundation Data Science Centre, London, UK; 8 Department of Public Health and Primary Care, University of Cambridge, Cambridge, UK; 9 National Heart and Lung Institute, Imperial College London, London, UK; 10 Centre for Experimental Medicine, Queen’s University Belfast School of Medicine Dentistry and Biomedical Sciences, Belfast, UK; 11 Centre of Infection and Immunity, Queens University Belfast, Belfast, UK; 12 Usher Institute of Population Health Sciences and Informatics, University of Edinburgh, Edinburgh, UK; 13 NHLI, Imperial College London, London, UK

**Keywords:** Asthma Epidemiology, COPD epidemiology, Interstitial Fibrosis

## Abstract

**Background:**

We describe the epidemiology of asthma, chronic obstructive pulmonary disease (COPD) and interstitial lung disease (ILD) from 2004 to 2023 in England, Wales, Scotland and Northern Ireland (NI) using a harmonised approach.

**Methods:**

Data from the National Health Service England (NHSE), Clinical Practice Research Datalink Aurum in England, Secure Anonymised Information Linkage Databank in Wales, DataLoch in South-East Scotland and the Honest Broker Service in NI were used. A harmonised approach to COPD, asthma and ILD case definitions, study designs and study populations across the four nations was performed. Age-sex-standardised incidence rates and point prevalence were calculated between 2004 and 2023 depending on data availability. Logistic and negative binomial regression compared incidence and prevalence rates between the start and end of each study period. Linear extrapolation projected incidence rates between 2020 and 2023 to illustrate how observed and projected rates differed.

**Results:**

Incidence rates were lower in 2019 versus 2005 for asthma (England: incidence rate ratio 0.89, 95% CI 0.88 to 0.90; Wales: 0.66, 0.65 to 0.68; Scotland: 0.67, 0.64 to 0.71; NI: 0.84, 0.81 to 0.86), COPD (England: 0.83, 0.82 to 0.85; Wales: 0.67, 0.65 to 0.69) and higher for ILD (England: 3.27, 3.05 to 3.50; Wales: 1.39, 1.27 to 1.53; Scotland: 1.63, 1.36 to 1.95; NI: 3.03, 2.47 to 3.72). In NHSE, the incidence of asthma was similar in June 2023 versus November 2019, but lower for COPD and higher for ILD. Prevalence of asthma in 2019 in England, Wales, Scotland and NI was 9.7%, 15.9%, 13.2% and 7.0%, respectively, for COPD 4.5%, 5.1%, 4.4% and 3.0%, and for ILD 0.4%, 0.5%, 0.6% and 0.3%. Projected incidence rates were 2.8, 3.4 and 1.8 times lower for asthma, COPD and ILD compared with observed rates at the height of the pandemic.

**Interpretation:**

Asthma, COPD and ILD affect over 10 million people across the four nations, and a substantial number of diagnoses were missed during the pandemic.

**WHAT IS ALREADY KNOWN ON THIS TOPIC**

- The burden of asthma, chronic obstructive pulmonary disease (COPD) and interstitial lung disease (ILD) has been explored in standalone countries with varying study designs and methods, thus limiting the comparability and representativeness of study results.

**WHAT THIS STUDY ADDS**

- This is the first study to describe the burden of asthma, COPD and ILD across England, Wales, Scotland and Northern Ireland over a 20-year period using harmonised electronic health records and paves the way for federated data analyses across the UK.

**HOW THIS STUDY MIGHT AFFECT RESEARCH, PRACTICE OR POLICY**

- This study demonstrates the value of using a harmonised methodological approach to using national electronic healthcare records across the UK to allow for large-scale comparable, replicable and updatable data that can be used for future policy and public health planning.

## Introduction

 Asthma, chronic obstructive pulmonary disease (COPD) and interstitial lung disease (ILD) are the three most common chronic respiratory diseases worldwide and in the UK and are associated with substantial morbidity and mortality.^1 2^ While previous studies have investigated changes in incidence and prevalence of asthma over time, these have been limited to specific populations, such as paediatric asthma populations, or are now outdated with data up until 2016.^3^,^5^ In addition, many studies have used data from England only, and no studies have investigated trends across England, Wales and Scotland combined. Similarly, the prevalence of COPD over time has been investigated in England only up until 2019 and studies investigating trends in incidence and prevalence of COPD and ILD across the UK are lacking and outdated.^6^,^8^

Cross-national studies of the burden of asthma, COPD and ILD in the UK are assessed through the metric of health service use. This metric is important for public health planning and identifying areas for future research and development to improve patient care. It is also important to understand in which groups of people incidence of asthma, COPD and ILD are higher to target these groups with more efficient interventions to reduce the burden of these three conditions. Likewise, given the emphasis on health inequalities in research and policy, it is important to understand where and in whom incidence or prevalence is perhaps lower than expected to target disease awareness and management campaigns. No previous studies have described the burden of these diseases across the UK in a harmonised way whereby results are more comparable. Similarly, no studies have estimated the burden of these diseases across the pandemic period where healthcare utilisation dramatically changed.

Our aim was to describe the changing epidemiology of asthma, COPD and ILD between 2004 and 2023 in England, Wales, Scotland and Northern Ireland (NI) using electronic healthcare records (EHR) through standardised methodologies to improve the understanding of the evolving impacts of these diseases on individuals and provide robust methodology which can be repeated going forward to monitor trends.

## Methods

### Databases

For England, primary care EHRs from the Clinical Practice Research Datalink (CPRD) Aurum (February 2022 build), for data between 2004 and 2019 and the General Practice Extraction Service for Pandemic Planning and Research (GDPPR) through the National Health Service England Secure Data Environment, via the British Heart Foundation Data Science Centre’s CVD-COVID-19 UK/COVID-19 IMPACT consortium, for data between 2019 and June 2023, were used (online supplemental file 1).^9^ For Wales, primary care data from the Welsh Longitudinal General Practice data set (WLGP) through the Secure Anonymised Information Linkage (SAIL) Databank was used to provide data between 2004 and 2019. For Scotland, the DataLoch service was used to provide data between 2004 and March 2023, which included primary care data from~80% of general practices (GPs) in Lothian (South-East Scotland) and linked secondary care data. For NI, primary care data from the Honest Broker Service (HBS) was used to provide data between 2004 and 2022. Harmonisation of key variables between CPRD, SAIL and DataLoch has been described previously, and online supplemental table S1 summarises the harmonisation methods taken to standardise these variables across all five data sources. Our previous work contains detailed information on how key variables were harmonised.^10^

### Chronic respiratory disease definitions (defining the numerator)

People with diagnosed and recorded asthma, COPD and ILD were defined using a harmonised approach to data curation across the databases to ensure cohort definitions were the same. Details of the methodology used have been described previously.^10^ For each disease, to be included as a case, individuals had to have at least one valid code within their primary care record, and the date of diagnosis was defined as a minimum of the earliest event date in primary or secondary care, using International Classification of Disease 10th revision codes in the primary position for hospitalisations. For NI, only primary care data were available and thus only a primary care diagnosis date was used. For COPD and ILD events, people were required to be at least 40 years old. In CPRD Arum, DataLoch and SAIL, for all three diseases, people had to have at least 1 year of follow-up from GP registration prior to their incidence code to be included in the numerator. Code lists for the asthma, COPD and ILD definitions are available on GitHub (https://github.com/NHLI-Respiratory-Epi/Curation-Harmonisation).

### Defining the denominator

The number of patients and patient follow-up time in the total CPRD population were obtained from patient denominator files provided by CPRD per data release for all ‘acceptable’ (ie, research-ready) patients and was limited to those with linkage eligibility.^10^ For National Health Service England (NHSE), the denominator was derived from GDPPR and therefore included patients with active, current GP registrations at participating practices in England (98% of GP practices in England), who were alive on 1 November 2019. Additional inclusion and exclusion criteria were applied for quality assurance purposes (online supplemental file 1). For SAIL Databank, the total number of patients was the population who had an active GP registration in the WLGP data set in Wales and who had 1 year of follow-up following GP registration. For DataLoch, people were included in the denominator if they were alive, registered and research-ready (ie, had a valid unique Community Health Index (CHI) and date of birth and sex matching CHI) with a DataLoch-registered GP and had 1 year of follow-up following GP registration. For HBS, the denominator was the mid-year estimate of the NI population (https://data.nisra.gov.uk/). For incidence rates, the denominator was the person-time for each time period in which individuals were still alive and registered with their GP and had not been diagnosed with the condition of interest. In NI, the denominator was incidence, which was defined as the number of people in each time period who were still alive and had not been diagnosed with the condition of interest. For prevalence, the denominator was the number of people who were still alive and or remained registered at their GP for each time period.

The same age restrictions for the numerator were applied to the denominator; for COPD and ILD, namely people aged 40 years or older were included, and there were no age restrictions for asthma.

### Statistical analysis

Data were analysed separately for each data source. In England, yearly incidence rates were calculated from 2004 to 2019 in CPRD, and monthly incidence rates were calculated between November 2019 and June 2023 in NHSE. In Wales, yearly incidence rates were calculated between 2004 and 2019 in SAIL. In NI, yearly incidence was calculated from 2004 to 2022. In South-East Scotland, yearly incidence rates were calculated from 2004 to 2019 and 3-monthly incidence rates were calculated between January 2020 and March 2023 in DataLoch due to data availability. Monthly or 3-monthly rates were calculated to better understand the changing epidemiology during and after the COVID-19 pandemic in more granular detail; however, yearly incidence was calculated for NI due to data availability. Incidence rates were based on the number of incident cases in a specific year or month divided by the person-time at risk for each person in each time period presented as per 1000 person-years. In NI, incidence was based on the number of incident cases in a specific year divided by mid-year population and presented as per 1000 persons. Incidence or incidence rates for each disease were stratified by age and sex in all nations and were additionally stratified by region for CPRD and NHSE, Index of Multiple Deprivation (IMD) and ethnicity in NHSE and IMD in HBS (online supplemental file 1). In addition, to facilitate comparisons between the four nations, incidence rates were directly standardised to the European 2013 standard population.^11^

Annual point prevalence of diagnosed and recorded disease was calculated using point prevalence for each disease for each year on 1 July from 2004 to 2019 for CPRD, SAIL and from 2004 to 2022 for DataLoch. For HBS, prevalence was calculated from 2011 to 2022 as the data sets used to identify study exit (ie, medications, secondary care utilisation and outpatient use) were available from 2010.

Crude incidence rate ratios (IRR) were calculated to compare the incidence of asthma, COPD and ILD in 2019 compared with 2005 in CPRD, SAIL and DataLoch. For HBS, the incidence risk ratio was calculated for each disease for 2019 compared with 2005. 2005 rather than 2004 was used for this analysis due to the introduction of the quality and outcomes framework in 2004 which could have led to more biased estimates in 2004 compared with those in 2005. For NHSE, IRRs were calculated to compare the incidence rates of asthma, COPD and ILD in June 2023 with November 2019 to determine whether incidence pre-COVID-19 pandemic differed from the most up-to-date estimates. Crude ORs were calculated to compare the prevalence of asthma, COPD and ILD in 2019 with 2005 in CPRD, SAIL and DataLoch, and in 2019 with 2011 in HBS.

### Exploratory analyses

To illustrate how incidence rates of asthma, COPD and ILD would have differed if the pandemic had not occurred, we projected incidence rates for England and South-East Scotland from January 2020 to June or March 2023, respectively. In addition, linear extrapolation was used to understand whether more recent incidence rates were similar to what would have been expected if the pandemic had not occurred. This method used estimated incidence rates based on the last two data points and was based on incidence rates in 2018 and 2019 for each country separately (online supplemental file 1).

## Results

Online supplemental figure S1 reports the number of people in each nation with at least one chronic respiratory condition or a combination of diseases.

### Trends in incidence

#### Asthma

**Figure 1:**
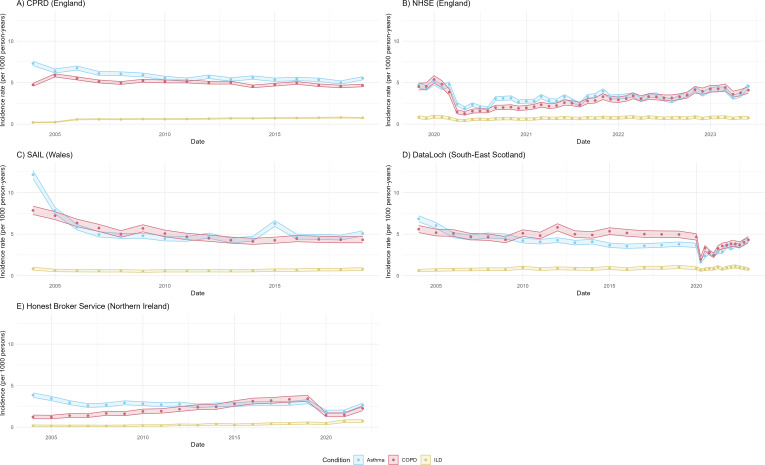
Age and sex adjusted incidence rate of asthma, COPD and ILD. Estimates illustrate incidence rates of asthma, COPD and ILD per 1000 person-years and 95% CIs calculated separately at each time point for England, Wales and Scotland. Yearly incidence rates were calculated for the years 2004 up until the end of 2020 for CPRD, SAIL and DataLoch, monthly incidence rates were calculated from November 2019 up until June 2023 for NHSE and 3-monthly incidence rates were calculated from January 2020 up until March 2023. Yearly incidence was calculated for Northern Ireland. COPD, chronic obstructive pulmonary disease; CPRD, Clinical Practice Research Datalink; ILD, interstitial lung disease; NHSE, National Health Service England; SAIL, Secure Anonymised Information Linkage.

**Figure 2:**
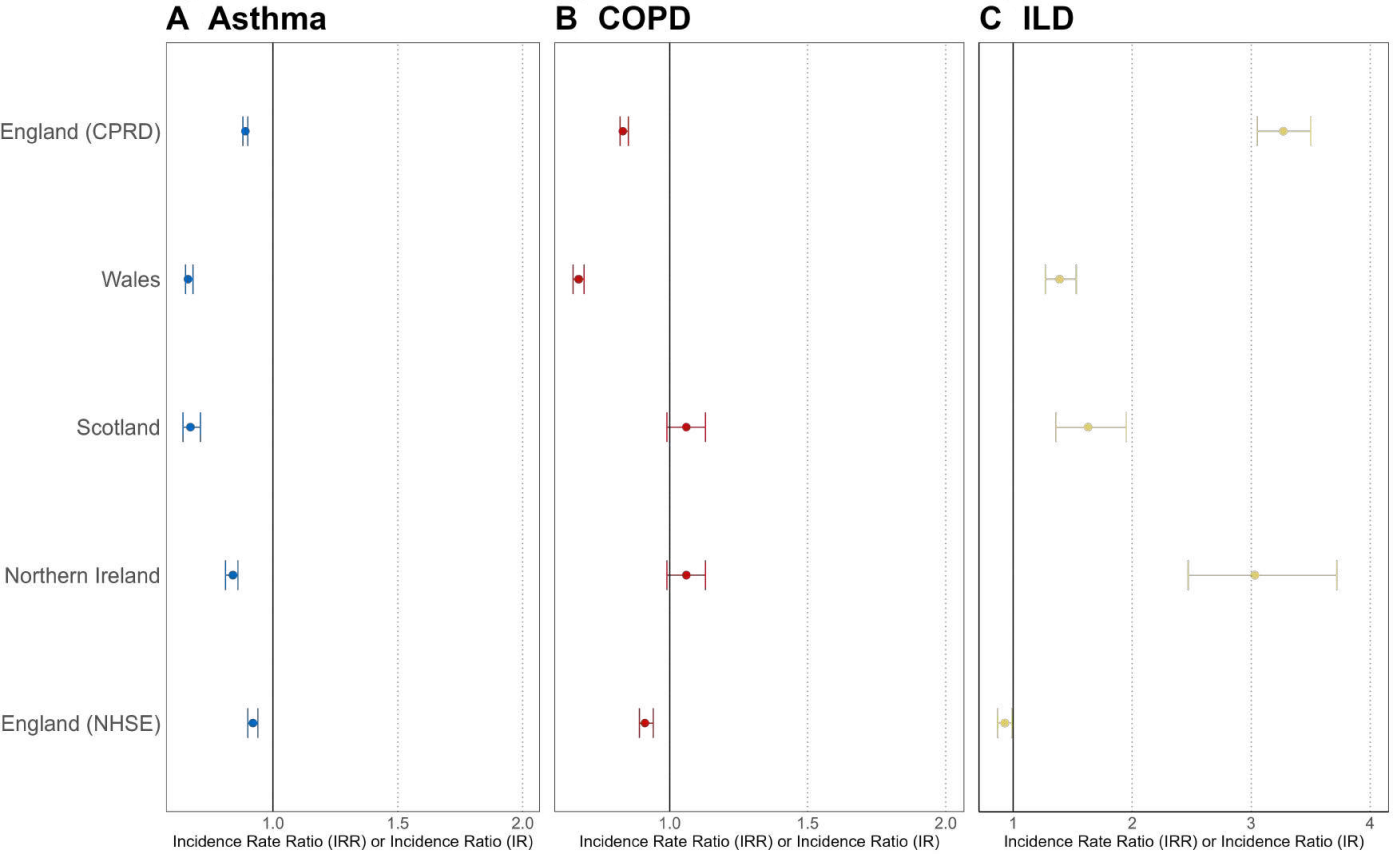
Crude incidence rate ratios (IRR) or incidence ratio (IR) of (A) asthma, (B) COPD and (C) ILD in 2019 versus 2005 for England, Wales, South-East Scotland and Northern Ireland and in June 2023 versus November 2019 for NHSE. Estimates illustrate IRR and 95% CIs for England, Wales and Scotland and IR for Northern Ireland. COPD, chronic obstructive pulmonary disease; CPRD, Clinical Practice Research Datalink; ILD, interstitial lung disease; IR, incidence ratio; IRR, incidence rate ratio; NHSE, National Health Service England.

Yearly adjusted incidence rates of asthma declined between 2004 and 2019 in all four nations (figure 1, online supplemental file 2). Across England, Wales and Scotland, incidence rates of asthma declined more rapidly between 2004 and 2010 (standardised incidence rates for England, Wales and Scotland from 2004 to 2010: 7.30 (95% CI 7.05 to 7.57) to 5.55 (95% CI 5.33 to 5.77), 12.16 (95% CI 11.69 to 12.65) to 4.47 (95% CI 4.18 to 4.77) and 6.85 (95% CI 6.49 to 7.22) to 4.22 (95% CI 3.94 to 4.51) per 1000 person-years (PY), respectively; online supplemental file 2). In NI, a substantial decline in asthma incidence was observed between 2004 and 2007: 3.86 (95% CI 3.59 to 4.14) to 2.60 (95% CI 2.38 to 2.83) per 1000 persons. From 2011 to 2019 the incidence of asthma remained more stable in all four nations (standardised incidence rates for England, Wales and Scotland in 2019: 5.52 (95% CI 5.32 to 5.72), 5.08 (95% CI 4.77 to 5.40) and 3.79 (CI 3.53 to 4.07) per 1000 PY, respectively. Incidence in NI in 2019: 2.66 (95% CI 2.44 to 2.90); online supplemental file 2). During the early COVID-19 pandemic years, the incidence of asthma declined rapidly (2020–2022), before increasing to 4.57 (95% CI 4.27 to 4.87) per 1000 person-years in June 2023 in England and to 4.25 (95% CI 3.97 to 4.55) per 1000 person-years between January and March 2023 in Scotland. Incidence rates of asthma were lower in 2019 compared with 2005 in England (0.89, 95% CI 0.88 to 0.90), Wales (0.66, 95% CI 0.65 to 0.68), South-East Scotland (0.67, 95% CI 0.64 to 0.71) and incidence in NI (0.84, 95% CI 0.81 to 0.86) (figure 2, online supplemental table S2).

Overall, differences were seen across all nations by sex whereby female adults had higher rates of asthma compared with males and males less than 18 had higher rates of asthma than females less than 18. In terms of age, younger individuals had the highest rates of asthma, although the rates have decreased since 2004 in these age groups. Rates were similar over time across regions for asthma. When incidence rates were stratified by IMD and ethnicity in NHSE, the incidence of asthma was higher in those in the more deprived IMD deciles and differed by ethnicity (online supplemental figures S2–S4 and S9–S11).

#### Chronic obstructive pulmonary disease

Incidence rates of COPD remained stable between 2004 and 2019 in England (standardised incidence rates in 2004 and 2019: 4.74 (95% CI 4.57 to 4.91) and 4.66 (95% CI 4.50 to 4.82); online supplemental file 2, figure 1). In Scotland, incidence rates of COPD declined between 2004 and 2009 (standardised incidence rates in 2004 and 2009: 5.60 (95% CI 5.19 to 6.04) and 4.33 (95% CI 3.97 to 4.71)) but remained stable between 2010 and 2019 (standardised incidence rates in 2010 and 2019: 5.11 (95% CI 4.72 to 5.53) and 4.96 (95% CI 4.58 to 5.37)). However, the incidence of COPD declined in Wales between 2004 and 2015 (standardised incidence rates in 2004 and 2015: 7.86 (95% CI 7.36 to 8.37) and 4.26 (95% CI 3.91 to 4.64)) and remained stable between 2016 and 2019 (standardised incidence rates in 2016 and 2019: 4.45 (95% CI 4.09 to 4.84) and 4.32 (95% CI 3.96 to 4.70)). In NI, COPD incidence increased substantially during the period, with largely consistent year-on-year increases observed between 2004 and 2019 (1.23, 95% CI 1.02 to 1.46) and 3.44 (95% CI 3.10 to 3.82) per 1000 persons, respectively. During the pandemic years, the incidence of COPD declined rapidly before increasing back to similar pre-pandemic rates in June 2023 in England and March 2023 in Scotland (standardised incidence rates: 4.09 (95% CI 3.72 to 4.50) and 4.33 (95% CI 3.97 to 4.71), respectively). Crude incidence rates of COPD were also lower in 2019 compared with 2005 in England (0.83, 95% CI 0.82 to 0.85) and Wales (0.67, 95% CI 0.65 to 0.69) but were similar in South-East Scotland (1.06, 95% CI 0.99 to 1.13), and incidence was higher in NI (2.82, 95% CI 2.62 to 3.03).

Males had higher incidence rates than females. In terms of age, older individuals had the highest incidence rates. A slight difference in rates was seen for COPD in more recent years whereby northern regions had higher incidence rates of COPD. When incidence rates were stratified by IMD and ethnicity in NHSE, incidence of asthma was higher in those in the more deprived IMD deciles and differed by ethnicity (online supplemental figures S5 and S6 and S9–S11).

#### Interstitial lung disease

Incidence rates of ILD increased in England, Scotland and NI between 2004 and 2019, but this trend was not seen for Wales (figure 1, online supplemental file 2). In England, the incidence of ILD increased between 2004 and 2006 (standardised incidence rates in 2004 and 2006: 0.20 (95% CI 0.17 to 0.24) and 0.57 (95% CI 0.51 to 0.63)) but remained similar between 2007 and 2010 (standardised incidence rates in 2007 and 2010: 0.59 (95% CI 0.53 to 0.65) and 0.60 (95% CI 0.55 to 0.66)) and increased between 2011 and 2019 (standardised incidence rates in 2011 and 2019: 0.62 (95% CI 0.56 to 0.68) and 0.76 (95% CI 0.71 to 0.82)). In Scotland, incidence rates increased between 2004 and 2010 (standardised incidence rates in 2004 and 2010: 0.61 (95% CI 0.47 to 0.77) and 0.95 (95% CI 0.78 to 1.15)), remained similar between 2011 and 2016 (standardised incidence rates in 2011 and 2016: 0.76 (95% CI 0.62 to 0.96) and 0.77 (95% CI 0.62 to 0.95)) and increased up until 2019 (standardised incidence rates 2019: 1.03 (95% CI 0.85 to 1.24)). In NI, incidence increased substantially between 2004 and 2019 (standardised incidence in 2004 and 2019: 0.18 (95% CI 0.13 to 0.28) and 0.49 (95% CI 0.37 to 0.64), respectively). In Wales, the incidence of ILD decreased between 2004 and 2009 (standardised incidence rates in 2004 and 2009: 0.83 (95% CI 0.67 to 1.01) and 0.52 (95% CI 0.40 to 0.68)), remained similar between 2010 and 2014 (standardised incidence rates for 2010 and 2014: 0.57 (95% CI 0.44 to 0.73) and 0.59 (95% CI 0.46 to 0.75)) and increased between 2015 and 2019 (standardised incidence rates in 2015 and 2019: 0.65 (95% CI 0.51 to 0.82) and 0.78 (95% CI 0.62 to 0.96)). During the pandemic years, the incidence of ILD declined before increasing back to similar pre-pandemic rates in June 2023 in England, but lower rates were seen in March 2023 in Scotland compared with pre-pandemic rates (standardised incidence rates: 0.75 (95% CI 0.59 to 0.93) and 0.78 (95% CI 0.62 to 0.96), respectively). Crude incidence rates of ILD were higher in 2019 compared with 2005 in England (3.27, 95% CI 3.05 to 3.50), Wales (1.39, 95% CI 1.27 to 1.53), South-East Scotland (1.63, 95% CI 1.36 to 1.95) and incidence was higher in NI (3.03, 95% CI 2.47 to 3.72). Crude incidence rates of asthma, COPD and ILD in England were lower in June 2023 compared with November 2019 (0.92, 95% CI 0.90 to 0.94, 0.91, 95% CI 0.89 to 0.94 and 0.93, 95% CI 0.87 to 0.99, respectively.)

Males had higher incidence rates than females. In terms of age, older individuals had the highest incidence rates. Rates were similar over time across regions. When incidence rates were stratified by IMD and ethnicity in NHSE, the incidence of asthma was higher in those in the more deprived IMD deciles and differed by ethnicity (online supplemental figures S7–S11).

### Trends in prevalence

#### Asthma

**Figure 3:**
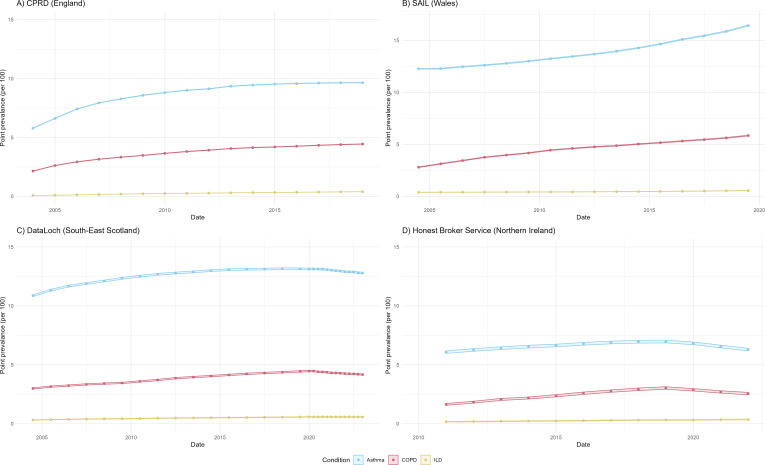
Point prevalence of asthma, COPD and ILD. Estimates illustrate point prevalence per 100 individuals and 95% CIs calculated separately at each time point. Point prevalence was calculated on the first of July of each year between 2004 and 2019 for England and Wales and between 2004 and 2022 for Lothian, Scotland and Northern Ireland. COPD, chronic obstructive pulmonary disease; CPRD, Clinical Practice Research Datalink; ILD, interstitial lung disease; SAIL, Secure Anonymised Information Linkage.

**Figure 4:**
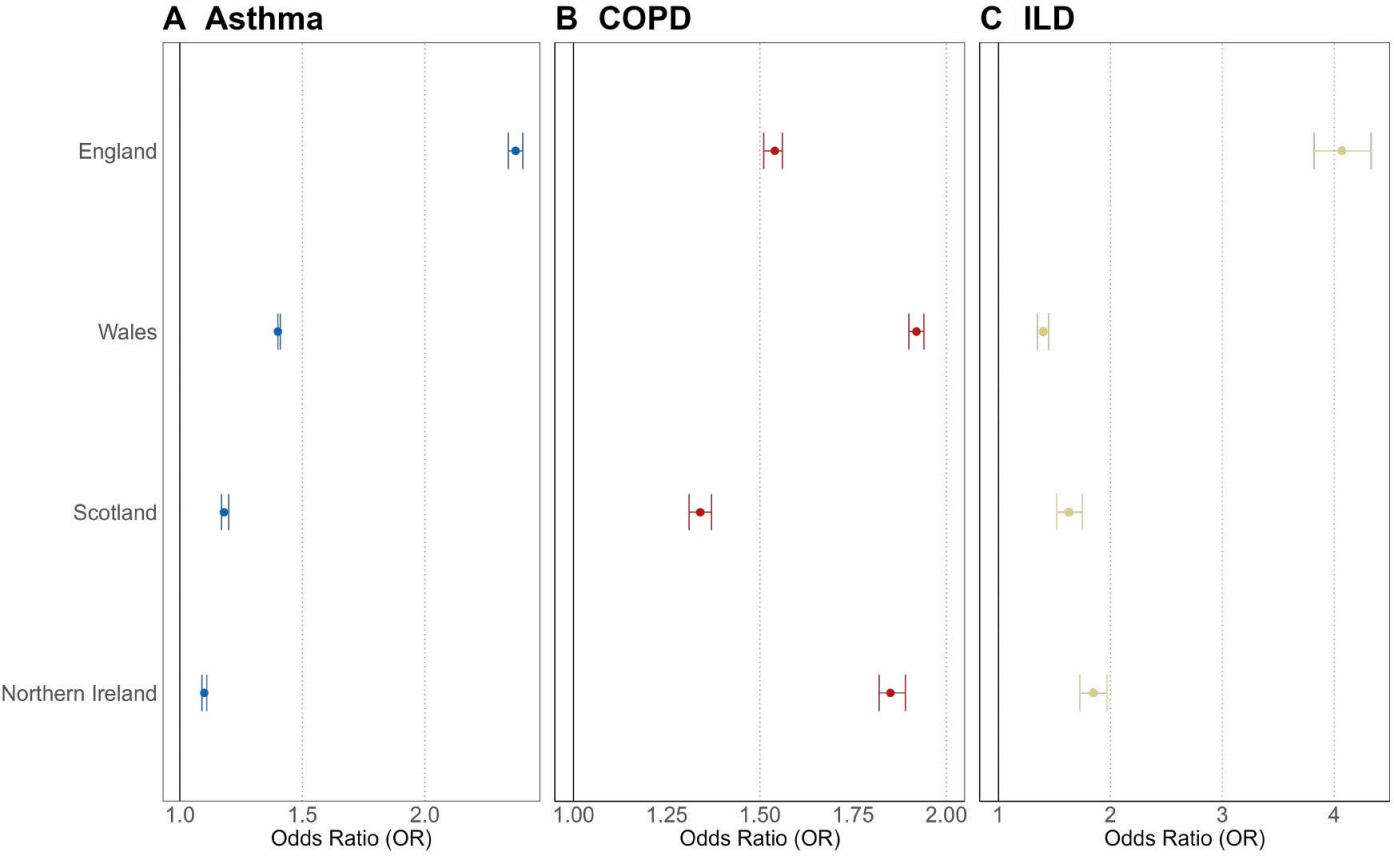
Crude odds ratios (OR) for the odds of (A) asthma, (B) COPD and (C) ILD in 2019 versus 2005 in England, Wales and South-East Scotland and 2019 versus 2011 in Northern Ireland. Estimates illustrate OR and 95% CIs. COPD, chronic obstructive pulmonary disease; ILD, interstitial lung disease.

In England, Wales and Scotland, the prevalence of asthma increased between 2004 and 2019 and between 2011 and 2019 in NI (figure 3, online supplemental table S3, online supplemental file 3). In England and Scotland, the prevalence of asthma increased between 2004 and 2015 (prevalence of asthma in 2004 and 2015: 5.79 (95% CI 5.78 to 5.81) and 9.53 (95% CI 9.52 to 9.55) in England and 10.87 (95% CI 10.79 to 10.94) and 13.07 (95% CI 13.0 to 13.14) in Scotland). The prevalence of asthma in England and Scotland was similar between 2016 and 2019. However, in Wales, the difference in prevalence between 2004 and 2010 was smaller, and prevalence of asthma increased between 2011 and 2019 to a larger extent (prevalence of asthma in 2004, 2010 and 2019 in Wales: 12.28 (95% CI 12.23 to 12.32), 13.25 (95% CI 13.21 to 13.29) and 16.43 (95% CI 16.38 to 16.47)). Crude odds of asthma were higher in 2019 compared with 2005 in England (2.37, 95% CI 2.34 to 2.40), Wales (1.35, 95% CI 1.34 to 1.35) and Lothian Scotland (1.18, 95% CI 1.17 to 1.20) (figure 4). In NI, crude OR of asthma was higher in 2019 compared with 2011 (1.10, 95% CI 1.09 to 1.11).

#### Chronic obstructive pulmonary disease

In England and Scotland, the prevalence of COPD increased between 2004 and 2015 (prevalence of COPD in 2004 and 2015: 2004 and 2015 in England and Scotland: 2.15 (95% CI 2.14 to 2.16) and 4.20 (95% CI 4.18 to 2.22) in England and 3.00 (95% CI 2.94 to 3.05) and 4.14 (95% CI 4.08 to 4.20) in Scotland). The prevalence of COPD in England and Scotland was similar between 2016 and 2019. However, in Wales, the difference in prevalence between 2004 and 2010 was smaller. Crude odds of COPD were also higher in 2019 compared with 2005 in England (1.54, 95% CI 1.51 to 1.56), Wales (1.89, 95% CI 1.87 to 1.91) and South-East Scotland (1.34, 95% CI 1.31 to 1.37). In NI, crude OR of COPD was higher in 2019 compared with 2011 (1.85, 95% CI 1.82 to 1.89).

#### Interstitial lung disease

Prevalence of ILD increased steadily between 2004 and 2019 in England and Scotland (prevalence of ILD in 2004 and 2019 in England: 0.08 (95% CI 0.08 to 0.09) and 0.40 (95% CI 0.40 to 0.41), respectively, and in Scotland: 0.33 (95% CI 0.31 to 0.35) and 0.57 (95% CI 0.55 to 0.60), respectively; figure 4 and online supplemental file 3). In NI, prevalence increased between 2011 and 2019 (0.17 (95% CI 0.15 to 0.2) and 0.36 (95% CI 0.32 to 0.40), respectively; figure 4 and online supplemental file 3). In Wales, prevalence increased steadily between 2004 and 2014 (0.40 (95% CI 0.39 to 0.41) and 0.47 (95% CI 0.46 to 0.48), respectively; figure 4 and online supplemental file 3), before increasing more rapidly up to 2019 (0.56 (95% CI 0.55 to 0.58); figure 4 and online supplemental file 3). Crude odds of ILD were higher in 2019 compared with 2005 in England (4.07, 95% CI 3.82 to 4.33). Crude odds of ILD were also higher in 2019 compared with 2005 in Wales and South-East Scotland, but at a smaller magnitude (Wales 1.34, 95% CI 1.30 to 1.39 and Scotland 1.63, 95% CI 1.42 to 1.75). In NI, crude OR of ILD was higher in 2019 compared with 2011 (1.85 95% CI 1.73 to 1.97).

### Observed versus projected incidence rates during the COVID-19 pandemic

The lowest incidence rate of asthma, COPD and ILD over the COVID-19 period in England was 1.86, 1.28 and 0.42 per 1000 person-years in May 2020, respectively. These were 2.8, 3.4 and 1.8 times lower than projected rates of asthma, COPD and ILD, respectively. The lowest incidence rate of asthma, COPD and ILD over the COVID-19 period in South-East Scotland was 1.65, 1.96 and 0.67 per 1000 person-years in April 2020, respectively. These were 2.4, 2.3 and 1.8 times lower than projected rates of asthma, COPD and ILD, respectively (online supplemental figure S12).

## Discussion

This is the first study to describe incidence and prevalence trends in asthma, COPD and ILD in England, Wales, Scotland and NI using harmonised methodologies and to describe the trends pre and post COVID-19. We found a decrease in the incidence of asthma in all four nations between 2004 and 2019, and a decrease in the incidence of COPD in Wales and England. The incidence of asthma, ILD and COPD declined during the COVID-19 pandemic period, but since then has gradually increased close to pre-pandemic rates. The prevalence of asthma has increased in four nations; however, a plateau was seen in South-East Scotland and England between 2015 and 2020, with a decrease during the pandemic. The prevalence of COPD also increased across all nations and decreased during the pandemic; however, the prevalence of ILD has increased over the study period and the decrease during the pandemic was much smaller relative to the other diseases.

### Asthma

Previous studies have found that the prevalence of asthma has increased over time in adults but has declined in children.^3^,^512 13^ While we did not stratify our prevalence rates by age group, we did find that incidence rates differed by age group and children aged less than 10 years had the steepest decline in asthma incidence between 2004 and 2019 compared with other age groups. The more pronounced decline seen in this population could be due to healthcare providers not giving children with suspected asthma a confirmed diagnosis of asthma, instead using a diagnosis of preschool wheeze when asthma is suspected.^14^ In addition, there has been a shift in attitudes to diagnosing asthma, as previously it tended to be overdiagnosed and in more recent years it is possible that physicians are more cautious when making a diagnosis of asthma, particularly in children.^15^ The incidence of asthma also varied by men and women in different age groups. It is well known that asthma is more common in males during childhood as compared with females, but in an adult population, asthma is more common in females than males.^16^ This is thought to be hormone-related, whereby after puberty the rise in oestrogen has an inflammatory effect which can lead to the development of inflammatory diseases including asthma.^17^

We also found that the incidence of asthma varied by IMD with higher rates of diagnoses made in more deprived individuals. This is consistent with the literature and persisted over the COVID-19 pandemic period too.^18 19^ Higher incidence rates in those who are more deprived may be due to factors including housing conditions, occupational exposures and air pollution.^20^ Similarly, there was some variation by ethnicity whereby people with Asian and mixed ethnicity had higher rates than those with other or unknown ethnicity. While the trends are less clear, other studies have found that people with black and Asian ethnicity also have poorer asthma outcomes, including asthma exacerbations and hospitalisations.^21^ Further research into understanding reasons for these disparities is crucial to determining where efforts are needed to reduce the incidence of asthma in these populations.

### Chronic obstructive pulmonary disease

Previous studies, including global estimates from the Global Burden of Disease study, have found that the incidence of COPD has decreased since 2004 and prevalence has increased, which is in line with our findings.^7 22 23^ While some studies have found that there are still disparities in people being diagnosed with COPD across the UK, others have found that the proportion of people dying of COPD and who had received a diagnosis of COPD has increased with time.^24^ One explanation for the decline in COPD incidence could be reduced levels of smoking over time, notably since the introduction of smoke-free legislation in 2006/2007.^25^ Interestingly, we did not find a decline in the incidence of COPD in Scotland or NI. In Scotland, this could be due to a higher prevalence of cigarette smoking compared with England and Wales, found notably in men in 2006.^26^ In NI, smoking cessation trends have been less pronounced than in other UK nations and air quality in Belfast remains among the worst in the UK.^27 28^ Across all UK nations, COPD prevalence increased, likely due to better diagnosis, improved treatments and ageing population.^6 10^

In addition, one systematic review found that the prevalence of COPD has historically been higher in males compared with females, but that the prevalence is similar between the two sexes in more recent years.^29^ While we did not stratify prevalence by sex, we did find that the incidence of COPD was consistently higher in males compared with females and varied by region, with the highest incidence in Northeast England in keeping with other work.^8 29^ The incidence of COPD was also higher in people with white ethnicity; however, it is possible that healthcare access in this group is more accessible and therefore individuals are more likely to get a diagnosis of COPD compared with other ethnic groups who have worse access to healthcare.^30 31^ On the other hand, in England, the incidence of COPD was higher in the most deprived populations, which could be due to higher smoking rates and more exposure to pollution.^32^

In England, the incidence of COPD was higher in people who were more socioeconomically deprived and who had white ethnicity. As with asthma, factors such as occupation and housing may play a role as well as cigarette smoking.^33^ Unlike asthma, people with white ethnicity had the highest rates of COPD diagnoses. Studies report that white ethnicity was more likely to be breathless, which could suggest that people with white ethnicity were more likely to seek healthcare when symptomatic.^30^

### Interstitial lung disease

There is a smaller body of research for ILD; however, evidence suggests that the incidence and prevalence of ILD have been increasing over time in all nations which were in our study, but also across Europe.^34^ One reason for the increased incidence and prevalence over time could be increased awareness of ILD and improved diagnosis and management of people with this disease.^35^ However, similarly to COPD, it could also be due to an ageing population, given that older age is a risk factor for ILD.^36^ Studies have also found that the prevalence of ILD is higher in males compared with females and a higher mortality rate for more deprived people with ILD.^37 38^ While we did not look at disparities in prevalence or mortality, we did find that the incidence of ILD was higher in males compared with females, and there was evidence to suggest that incidence was higher in people who were most deprived compared with those who were least deprived; however, the difference was relatively small.

While incidence rates of ILD in England were less clear by socioeconomic deprivation, people with Asian and white ethnicity had higher rates over time. It is unclear as to why these differences exist, and they could be largely due to residual confounding, particularly with socioeconomic status. Further understanding into why disparities in chronic respiratory diseases exist is crucial to improving population health.

### COVID-19

The incidence of clinically recorded COPD, asthma and ILD declined during the pandemic and gradually increased close to pre-pandemic levels by June 2023. The reduction in clinically recorded diagnoses reflects access to care during the pandemic and changes in health-seeking behaviours.^39 40^ Specifically, there were fewer face-to-face GP and outpatient appointments during the early months of the pandemic, leading to fewer diagnoses.^41^ The incidence rates of asthma, COPD and ILD in June 2023 remained slightly lower than the incidence rate in November 2019. This lag in diagnosing people with chronic respiratory disease could be due to the need for spirometry, which would have been limited during the pandemic.^42^ In addition, based on projected rates for if the pandemic had not occurred, up to 2.8 times fewer people were diagnosed with asthma, 3.4 times fewer people were diagnosed with COPD and 1.8 times fewer people were diagnosed with ILD at the height of the pandemic. Given the changes in diagnostic practices during the pandemic, the observed decline in incidence rates suggests that there were missed diagnoses, which could have a great impact on the future health of people with chronic respiratory diseases but who may not be diagnosed with the condition due to changes in care during the pandemic. Not only does this affect individuals who may be living with an undiagnosed respiratory disease, but it would affect individuals who are diagnosed with a respiratory disease later on in their disease trajectory. This is particularly important for people with ILD as diagnosis is less straightforward and is already diagnosed at a late stage whereby patients have a short life expectancy. Further studies are needed to quantify the long-term burden of undiagnosed and late-diagnosed individuals.

### Implications for policy, practice and research

Given the Office for National Statistis (ONS) population estimates in 2023 and assuming no dramatic increase in prevalence over the pandemic years, our prevalence estimates suggest that approximately 5.5 million (9.6%) people are living with asthma in England, 590 000 (16.4%) in Wales, 720 000 (13.2%) in Scotland and 146 000 (7.6%) in NI. For COPD our prevalence estimates in 2019 reflect approximately 2.5 million (4.5%) people in England, 185 000 (5.9%) in Wales, 241 000 (4.4%) in Scotland and 58 000 (3.0%) in NI. For ILD our prevalence estimates in 2019 reflect approximately 230 000 (0.4%) in England, 18 000 (0.6%) in Wales, 31 000 (0.6%) in Scotland and 6000 (0.3%) in NI. Overall, these three diseases affect over 10 million people across the UK, highlighting the importance of continued research into these diseases to improve management of people with asthma, COPD and ILD, to reduce morbidity and mortality. These estimates suggest that the UK still has some of the worst respiratory outcomes worldwide, as mean global age-standardised prevalence estimates for 2019 for asthma and COPD were lower than those seen in our study however, the declining trends seen in our study have also been reported globally.^23^

Our study is one of the first studies to use cross-national data in a harmonised approach to describe trends in the three most prevalent chronic respiratory diseases of asthma, COPD and ILD over a 20-year period. Our harmonised methodology not only allows more comparable findings across the four UK nations, but also allows more replicable and updatable cohorts of people with asthma, COPD and ILD in the UK.

### Limitations

Despite our study’s strengths, limitations exist. First, EHR data were used, which may have limitations in terms of coding of diagnoses; however, we used codes that have been validated in CPRD for COPD, asthma and ILD diagnoses to minimise any biases from this.^43^,^45^ Second, the GDPPR primary care data set in NHSE includes all individuals in England who are alive with active registration on 1 November 2019 and therefore we were unable to estimate monthly prevalence rates. Third, we were unable to stratify by region, IMD and ethnicity in CPRD due to the lack of denominator data for these specific variables. Fourth, this paper investigated trends in diagnosed disease and cannot separate out changes related to diagnosis and coding practices from true changes in incidence and prevalence. Fifth, denominators over the pandemic period varied by England and Scotland (monthly vs 3-monthly) which could have led to less variation in rates in Scotland over this period. In addition, the rates over the pandemic period could have been affected by seasonality and should be interpreted with this in mind. Furthermore, there is possible overlap between people included in CPRD and NHSE; however, this would have only occurred for the months of November and December in NHSE, and rates were similar. In addition, extrapolation assumed linearity; however, data from 2004 to 2019 was relatively linear for CPRD and DataLoch. Moreover, for NI data the definition of study exit was pragmatic and may have led to the inappropriate exclusion of some prevalent cases who still resided in NI, especially those with less severe diseases who may not be in regular contact with the healthcare system. Furthermore, data were analysed separately for each data source and aggregate data were combined. Therefore, incidence rates were age and sex standardised to the European Union (EU) standard population to allow for comparable rates across the data sources and no other covariates were adjusted for. Lastly, case definitions were based on diagnoses made in primary and secondary care alone as spirometry measurements were not used. A previous systematic review found that between 50% and 75% of COPD populations in the literature had airflow obstruction as defined using post-bronchodilator spirometry. On the other hand, spirometry-confirmed COPD without a clinically recorded diagnosis was common.^46^ Similarly for asthma, misdiagnosis is possible, notably in those who did not have testing for airflow limitation.^47^ Therefore, it is possible that the incidence and prevalence of COPD, asthma and ILD could have been over or under-estimated; however, we used algorithms for disease diagnosis that have been validated in UK EHRs to reduce the risk of misdiagnosis.^43^,^45^

## Conclusion

This is the first study using harmonised EHR data from the four nations in the UK to describe the trend in epidemiology of three chronic respiratory diseases over a combined 20-year period. In the four nations, the incidence of asthma has decreased, the incidence of ILD has increased; however, the incidence of COPD has decreased in England and Wales only. Changes in incidence rates were likely due to changes in behaviours and disease awareness. Disparities in incidence rates exist by sex, age, region, IMD and ethnicity, and missed diagnoses from the COVID-19 pandemic could have a great impact on the future health of people with chronic respiratory diseases. Overall, these data form a strong baseline for future policy work and public health planning.

## Supplementary material

10.1136/thorax-2024-222699 online supplemental file 1

10.1136/thorax-2024-222699 online supplemental file 2

10.1136/thorax-2024-222699 online supplemental file 3

## Data Availability

All data relevant to the study are included in the article or uploaded as supplementary information.

## References

[1] Mukherjee M, Stoddart A, Gupta RP (2016). The epidemiology, healthcare and societal burden and costs of asthma in the UK and its member nations: analyses of standalone and linked national databases. BMC Med.

[2] GBD 2015 Chronic Respiratory Disease Collaborators (2017). Global, regional, and national deaths, prevalence, disability-adjusted life years, and years lived with disability for chronic obstructive pulmonary disease and asthma, 1990-2015: a systematic analysis for the Global Burden of Disease Study 2015. Lancet Respir Med.

[3] Kallis C, Maslova E, Morgan AD (2023). Recent trends in asthma diagnosis, preschool wheeze diagnosis and asthma exacerbations in English children and adolescents: a SABINA Jr study. Thorax.

[4] Bloom CI, Saglani S, Feary J (2019). Changing prevalence of current asthma and inhaled corticosteroid treatment in the UK: population-based cohort 2006–2016. Eur Respir J.

[5] Simpson CR, Sheikh A (2010). Trends in the epidemiology of asthma in England: a national study of 333,294 patients. J R Soc Med.

[6] Stone PW, Osen M, Ellis A (2023). Prevalence of Chronic Obstructive Pulmonary Disease in England from 2000 to 2019. Int J Chron Obstruct Pulmon Dis.

[7] Simpson CR, Hippisley-Cox J, Sheikh A (2010). Trends in the epidemiology of chronic obstructive pulmonary disease in England: a national study of 51 804 patients. Br J Gen Pract.

[8] Nacul L, Soljak M, Samarasundera E (2011). COPD in England: a comparison of expected, model-based prevalence and observed prevalence from general practice data. J Public Health (Oxf).

[9] NHS (2024). General practice extraction service (GPES) data for pandemic planning and research: a guide for analysts and users of the data. https://digital.nhs.uk/coronavirus/gpes-data-for-pandemic-planning-and-research/guide-for-analysts-and-users-of-the-data.

[10] Hatam S, Scully ST, Cook S (2024). A Harmonised Approach to Curating Research-Ready Datasets for Asthma, Chronic Obstructive Pulmonary Disease (COPD) and Interstitial Lung Disease (ILD) in England, Wales and Scotland Using Clinical Practice Research Datalink (CPRD), Secure Anonymised Information Linkage (SAIL) Databank and DataLoch. Clin Epidemiol.

[11] Eurostat (2013). Revision of the European standard population, EE Commision, editor.

[12] Anandan C, Nurmatov U, Van Schayck OCP (2010). Is the prevalence of asthma declining? Systematic review of epidemiological studies. Allergy.

[13] NICE (2023). What is the prevalence of asthma?, N.I.f.H.a.C. excellence, Editor.

[14] Looijmans-van den Akker I, van Luijn K, Verheij T (2016). Overdiagnosis of asthma in children in primary care: a retrospective analysis. Br J Gen Pract.

[15] Kavanagh J, Jackson DJ, Kent BD (2019). Over- and under-diagnosis in asthma. Breathe (Sheff).

[16] Dharmage SC, Perret JL, Custovic A (2019). Epidemiology of Asthma in Children and Adults. Front Pediatr.

[17] Radzikowska U, Golebski K (2023). Sex hormones and asthma: The role of estrogen in asthma development and severity. Allergy.

[18] Ellison-Loschmann L, Sunyer J, Plana E (2007). Socioeconomic status, asthma and chronic bronchitis in a large community-based study. *Eur Respir J*.

[19] Schyllert C, Lindberg A, Hedman L (2020). Low socioeconomic status relates to asthma and wheeze, especially in women. *ERJ Open Res*.

[20] (2018). On the edge: how inequality affects people with asthma, a. UK, editor.

[21] Hayanga B, Stafford M, Bécares L-O (2021). Ethnic Inequalities in Healthcare Use and Care Quality among People with Multiple Long-Term Health Conditions Living in the United Kingdom: A Systematic Review and Narrative Synthesis. Int J Environ Res Public Health.

[22] Snell N, Strachan D, Hubbard R (2016). S32 Epidemiology of chronic obstructive pulmonary disease (COPD) in the uk: findings from the british lung foundation’s ‘respiratory health of the nation’ project. Thorax.

[23] Momtazmanesh S (2023). Global burden of chronic respiratory diseases and risk factors, 1990–2019: an update from the Global Burden of Disease Study 2019. EClinicalMedicine.

[24] Gayle A, Lenoir A, Minelli C (2022). Are we missing lifetime COPD diagnosis among people with COPD recorded death? A population-based retrospective cohort study. BJGP Open.

[25] Dai X, Gakidou E, Lopez AD (2022). Evolution of the global smoking epidemic over the past half century: strengthening the evidence base for policy action. Tob Control.

[26] S Government (2010). Scottish health survey: topic report UK comparisons, Ed.

[27] Swinney, K.E.V.Q.P (2020). Cities outlook 2020 holding our breath, centreforcities, editor.

[28] ONS (2023). Adult smoking habits in the UK: 2022. https://www.ons.gov.uk/peoplepopulationandcommunity/healthandsocialcare/healthandlifeexpectancies/bulletins/adultsmokinghabitsingreatbritain/2022.

[29] Stolz D, Kostikas K, Loefroth E (2019). Differences in COPD Exacerbation Risk Between Women and Men: Analysis From the UK Clinical Practice Research Datalink Data. Chest.

[30] Martin A, Badrick E, Mathur R (2012). Effect of ethnicity on the prevalence, severity, and management of COPD in general practice. *Br J Gen Pract*.

[31] Ajayi Sotubo O (2021). A perspective on health inequalities in BAME communities and how to improve access to primary care. *Future Healthc J*.

[32] Levin KA, Anderson DR, Crighton EM (2020). Prevalence of COPD by age, sex, socioeconomic position and smoking status; a cross-sectional study. HE.

[33] Hitchman SC, Fong GT, Zanna MP (2014). Socioeconomic status and smokers’ number of smoking friends: Findings from the International Tobacco Control (ITC) Four Country Survey. Drug Alcohol Depend.

[34] Salciccioli JD, Marshall DC, Goodall R (2022). Interstitial lung disease incidence and mortality in the UK and the European Union: an observational study, 2001-2017. ERJ Open Res.

[35] Silva M, Fernandes A, Pereira AR (2022). Awareness towards the main ILD among primary care physicians. Multidiscip Respir Med.

[36] Choi W-I, Dauti S, Kim HJ (2018). Risk factors for interstitial lung disease: a 9-year Nationwide population-based study. BMC Pulm Med.

[37] Kawano-Dourado L, Glassberg MK, Assayag D (2021). Sex and gender in interstitial lung diseases. Eur Respir Rev.

[38] Goobie GC, Ryerson CJ, Johannson KA (2022). Neighborhood-Level Disadvantage Impacts on Patients with Fibrotic Interstitial Lung Disease. Am J Respir Crit Care Med.

[39] Van den Bulck S, Crèvecoeur J, Aertgeerts B (2022). The impact of the Covid-19 pandemic on the incidence of diseases and the provision of primary care: A registry-based study. PLoS ONE.

[40] Mansfield KE, Mathur R, Tazare J (2021). Indirect acute effects of the COVID-19 pandemic on physical and mental health in the UK: a population-based study. Lancet Digit Health.

[41] Bottle A, Neale FK, Foley KA (2022). Impact of COVID-19 on outpatient appointments in children and young people in England: an observational study. *BMJ Open*.

[42] Qi C, Osborne T, Bailey R (2023). Impact of COVID-19 pandemic on incidence of long-term conditions in Wales: a population data linkage study using primary and secondary care health records. Br J Gen Pract.

[43] Quint JK, Müllerova H, DiSantostefano RL (2014). Validation of chronic obstructive pulmonary disease recording in the Clinical Practice Research Datalink (CPRD-GOLD). BMJ Open.

[44] Nissen F, Morales DR, Mullerova H (2017). Validation of asthma recording in the Clinical Practice Research Datalink (CPRD). BMJ Open.

[45] Morgan A, Gupta RS, George PM (2023). Validation of the recording of idiopathic pulmonary fibrosis in routinely collected electronic healthcare records in England. BMC Pulm Med.

[46] Perret J, Yip SWS, Idrose NS (2023). Undiagnosed and “overdiagnosed” COPD using postbronchodilator spirometry in primary healthcare settings: a systematic review and meta-analysis. BMJ Open Respir Res.

[47] Aaron SD, Vandemheen KL, FitzGerald JM (2017). Reevaluation of Diagnosis in Adults With Physician-Diagnosed Asthma. *JAMA*.

