# Supplementary material for: Incidence and prevalence of asthma, chronic obstructive pulmonary disease and interstitial lung disease between 2004 and 2023: harmonised analyses of longitudinal cohorts across England, Wales, South-East Scotland and Northern Ireland
Source: Thorax. 2025 Apr 8;80(7):e222699. doi: 10.1136/thorax-2024-222699 (PMC12322415; doi:10.1136/thorax-2024-222699)
Supplement: online supplemental file 1 [file thorax-80-7-s001.pdf]

Incidence and prevalence of asthma, chronic obstructive pulmonary disease and interstitial lung disease between 2004 and 2023: harmonised analyses of longitudinal cohorts across England, Wales and southeast Scotland

Contents

Additional methods..2

Additional results..3

Figure S1: Number of people who develop asthma, COPD, and ILD in England, Wales, Scotland, and Northern Ireland over study period..4

Figure S2: Age adjusted incidence rates of asthma by sex in people aged <20 years old..5

Figure S3: Age adjusted incidence rates of asthma by sex in people aged 20+ years old..6

Figure S4: Sex adjusted incidence rates of asthma by age..7

Figure S5: Age adjusted incidence rates of COPD by sex..8

Figure S6: Sex adjusted incidence rates of COPD by age..9

Figure S7: Age adjusted incidence rates of ILD by sex..10

Figure S8: Sex adjusted incidence rates of ILD by age..11

Figure S9: Age and sex adjusted incidence rates of COPD, asthma, and ILD by region in England......12

Figure S10: Age and sex adjusted incidence rates of COPD, asthma, and ILD by IMD in NHSE SDE, England..13

Figure S11: Age and sex adjusted incidence rates of COPD, asthma, and ILD by ethnicity in NHSE SDE, England..14

Figure S12: Observed vs. projected incidence rates of asthma, COPD, and ILD during the pandemic.15

Table S1: Harmonisation methods for key variables across CPRD, SAIL, DataLoch, NHSE, and HBS ...16

Table S2: Crude IRR or IR for incidence of asthma, COPD, and ILD in 2019 vs 2005 for England (CPRD), Wales, Scotland, and Northern Ireland, and in June 2023 vs November 2019 for England (NHSE SDE)..17

Table S3: Crude OR for prevalence of asthma, COPD, and ILD in 2019 vs 2005 for England (CPRD), Wales, and Scotland, and in 2019 vs 2011 for Northern Ireland (Honest Broker Service)..18

Additional methods

Additional Data sources Information

Deidentified GDPPR data were accessed and analysed through the British Heart Foundation (BHF) Data Science Centre's CVD-COVID-UK/COVID-IMPACT consortium within NHSE's SDE. CPRD and GDPPR data were linked to secondary care records from Hospital Episode Statistics (HES) and Office for National Statistics (ONS) mortality data by CPRD or NHSE. Data quality checks were implemented to ensure data validity. Specifically, we ensured that: 1) date of birth was recorded before date of death; 2) recorded sex and date of birth were not missing; and 3) there were no recorded pregnancies or births for men and no recorded prostate cancer for women. The SAIL Databank is a secure repository of anonymised health and administrative data in Wales, and this study used linked primary care data from the Welsh Longitudinal General Practice dataset (WLGP), the Welsh Demographic Service Dataset (WDSD), and the Annual District Death Extract dataset (ADDE). This study utilised previously curated asthma, COPD and ILD cohorts generated in CPRD Aurum, SAIL Databank and DataLoch for coverage in England, Wales and South-East, respectively. [1]

The Northern Ireland data consisted of all individuals living in NI who were registered with a General Practitioner (GP). Patients entered the study on 1st January 2004 and left the cohort at the earliest date of last data extraction (31st December 2022) or when they were assumed to be dead or no longer living in NI (defined as one year from their last interaction with healthcare services [medication pickup, hospitalisation, ED attendance or outpatient visit]).

Additional Statistical analyses Information

For asthma, incidence rates were stratified in 5-year age bands up until age 20. At age 20 and older, incidence rates were stratified in 10-year age bands up until 70 years or older. For COPD and ILD, incidence rates were stratified in 10-year age bands up until 70 years or older. Due to known differences in asthma incidence in men and women by age, sex stratified rates were calculated in those with age less than 20 years and 20 years or older separately. Further stratifications were undertaken in the English NHSE data by region, Index of Multiple Deprivation (IMD), and ethnicity (White, Black, Asian, Mixed, Other, Unknown). In addition, to facilitate comparisons between the three nations, incidence rates were directly standardised to the European 2013 standard population. [2]

Yearly point prevalence was calculated based on number of prevalent cases alive and in follow-up on the 1st July for each year. Point prevalence for NHSE data was not calculated. This was because the GDPPR data included all individuals who were alive and registered with a general practice on the 1st November 2019 and those who were born after this date were not included.

Additional Exploratory analyses information

Projected incidence rates for asthma, COPD, and ILD were calculated in England and Scotland from 2020 onwards. For England, projected numerators and denominators over the pandemic period were calculated using CPRD England data from 2004 to 2019. First, annual numerators and denominators were divided by 12 to generate average monthly numerators and denominators in line with the monthly NHSE incidence rates over the pandemic period. Second, the average monthly CPRD numerator and denominators were adjusted in size to match NHSE population (as CPRD Arum covers approximately 20% of the population of England registered at a GP, whereas NHSE covers 98% in England with active GP registration). Third, an average numerator and denominator was calculated for every 2-year period to account for any noise. Fourth, projected numerators and

denominators from January 2020 to June 2023 were projected using linear interpolation based on the 2-yearly average numerators and denominators from CPRD. Lasty, incidence rates were calculated using the projected numerators and denominators. This was repeated using the Scottish data however, projected numerators and denominators were based on the Scottish data alone and the scaling up was not needed, like it was for England.

Additional results

Trends in incidence rates of asthma, COPD, and ILD by sex

Incidence rates of asthma in individuals less than 20 years old were higher in men than in women (**Figure S2, supplement 2**). In those aged 20 years or older, incidence of asthma was higher in women than in men and the difference between men and women was greater than that in those aged less than 20 years old between January 2020 and July 2023 in England **(Figure S3, supplement 2)**.Incidence rates of COPD were consistently higher in men than in women between 2004 and 2023 in England using CPRD and NHSE however, the difference in incidence rates between the sexes were less pronounced between 2020 and March 2023 in Scotland **(Figure S4, supplement 2**). Incidence rates of ILD were consistently higher in men compared with women **(Figure S5, supplement 2**).

Trends in Incidence rates of asthma, COPD, and ILD by age

Incidence of asthma was consistently higher in those aged 0-9 years of age in England, Wales, and Scotland between 2005 and 2019. Whilst incidence of asthma declined dramatically in those aged 0-5 years during this period, all other age groups remained relatively stable. Incidence of asthma declined over the pandemic years but has increased in those aged 0-4, 10-14, and 70+ since (**Figure S6, supplement 2**). In England, the incidence of COPD was higher in individuals aged 70 years or older in both CPRD and NHSE. Whilst yearly incidence of COPD declined slightly between 2004 and 2019 in those aged 60 or older, the incidence remained stable in those aged 40-59. In Wales and Scotland, the incidence of COPD was highest in individuals aged 60-69 and COPD incidence declined for all individuals aged 50 years or older. The incidence remained stable for those aged 40-49 (**Figure S7, supplement 2**). Incidence of ILD was remained the highest in those aged 70 and older in all nations (**Figure S8**)., **supplement 2**

Trends in incidence rates of asthma, COPD, and ILD by region, IMD, and ethnicity in England

Incidence of asthma, and ILD were similar between regions in England from 2004 to July 2023. The incidence of COPD varied slightly between regions, notably in June 2023. The incidence of COPD in November 2019 and June 2023 was highest in Northeast England (**Figure S9, supplement 2**). Incidence of asthma, COPD, and ILD was also higher in people in the most deprived IMD decline in England (**Figure S10, supplement 2**). Incidence of asthma was lowest in people with unknown and other ethnicity and highest in people with Asian or Mixed ethnicity however, incidence of COPD was highest in people with White ethnicity (**Figure S11, supplement 2**).

Figure S1: Number of people who develop asthma, COPD, and ILD in England, Wales, Scotland, and Northern Ireland over study period

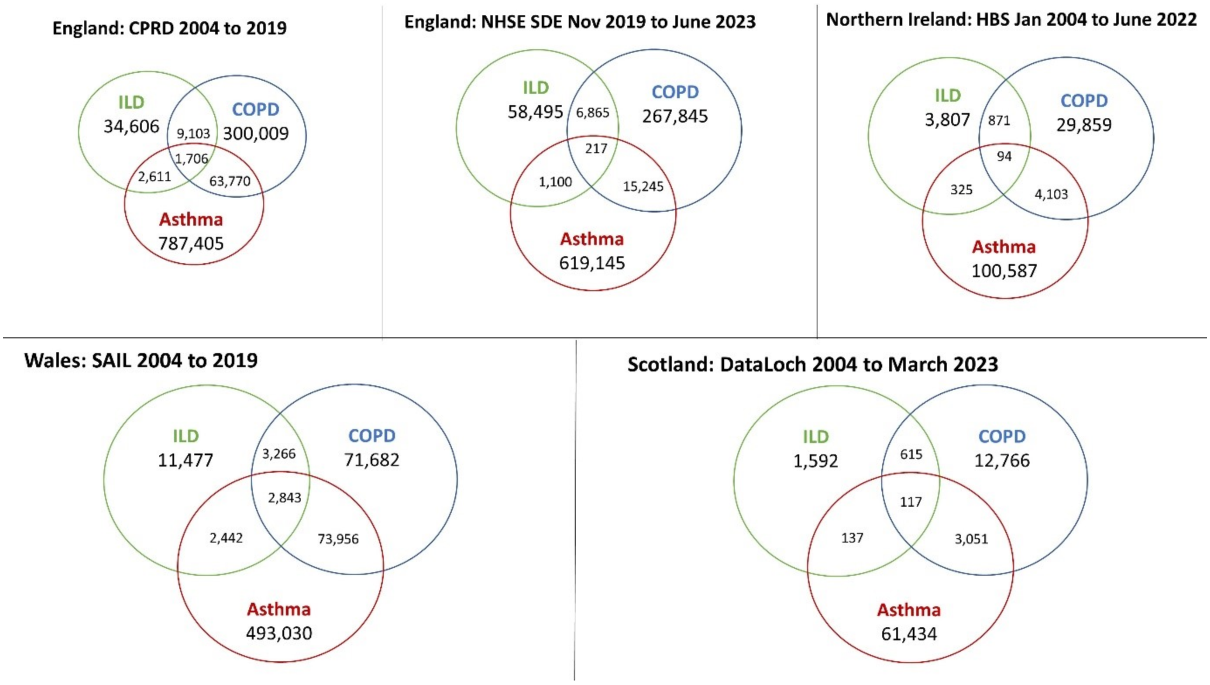

Legend: Number of incident cases of asthma, COPD and ILD in each nation over each nation's total available study period. ILD (interstitial lung disease), COPD (chronic obstructive pulmonary disease), CPRD (clinical practice research datalink).

Figure S2: Age adjusted incidence rates of asthma by sex in people aged <20 years old

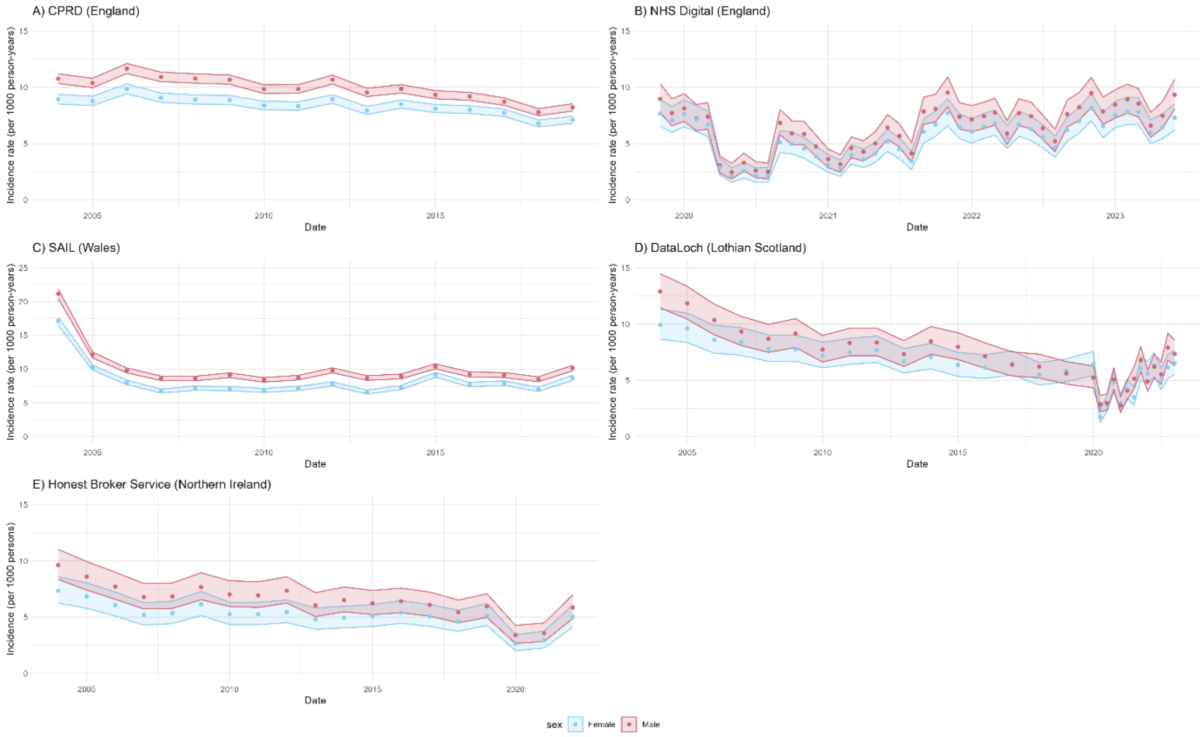

Legend: Estimates illustrate incidence rates of asthma per 1000 person-years and 95% confidence intervals. Yearly incidence rates were calculated for the years 2004 up until the end of 2019 for CPRD, SAIL and DataLoch, monthly incidence rates were calculated from November 2019 up until June 2023 for NHSE, and 3-monthly incidence rates were calculated from January 2020 up until March 2023. Incidence per 1000 persons was calculated for NI for the year 2004 to 2022.

Figure S3: Age adjusted incidence rates of asthma by sex in people aged 20+ years old

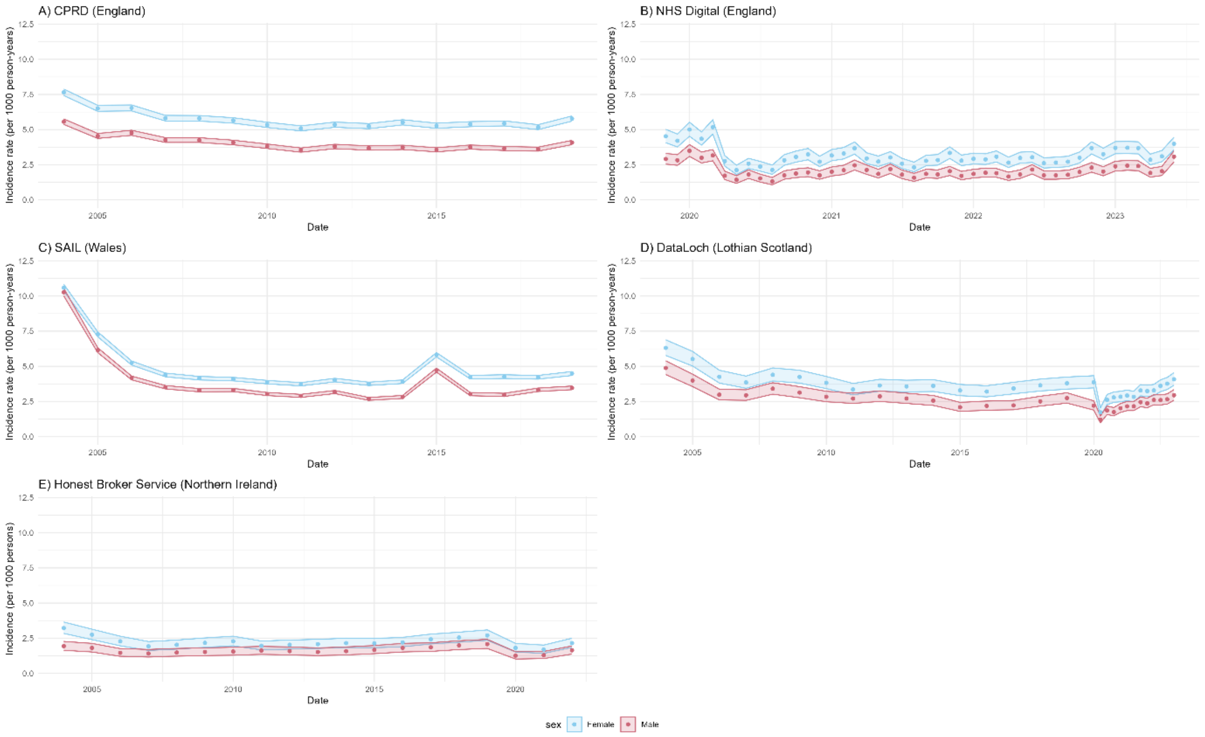

Legend: Estimates illustrate incidence rates of asthma per 1000 person-years and 95% confidence intervals. Yearly incidence rates were calculated for the years 2004 up until the end of 2019 for CPRD, SAIL and DataLoch, monthly incidence rates were calculated from November 2019 up until June 2023 for NHSE, and 3-monthly incidence rates were calculated from January 2020 up until March 2023. Incidence per 1000 persons was calculated for NI for the year 2004 to 2022.

Figure S4: Sex adjusted incidence rates of asthma by age

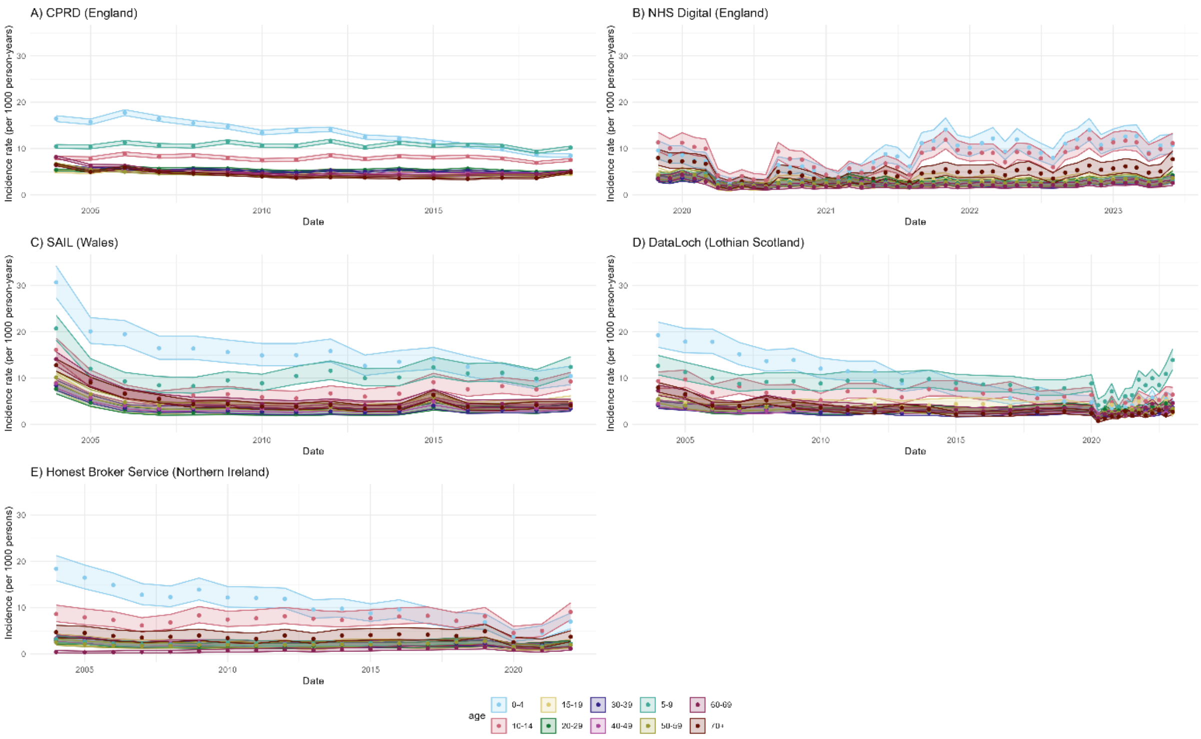

Legend: Estimates illustrate incidence rates of asthma per 1000 person-years and 95% confidence intervals. Yearly incidence rates were calculated for the years 2004 up until the end of 2019 for CPRD, SAIL and DataLoch, monthly incidence rates were calculated from November 2019 up until June 2023 for NHSE, and 3-monthly incidence rates were calculated from January 2020 up until March 2023. Incidence per 1000 persons was calculated for NI for the year 2004 to 2022.

Figure S5: Age adjusted incidence rates of COPD by sex

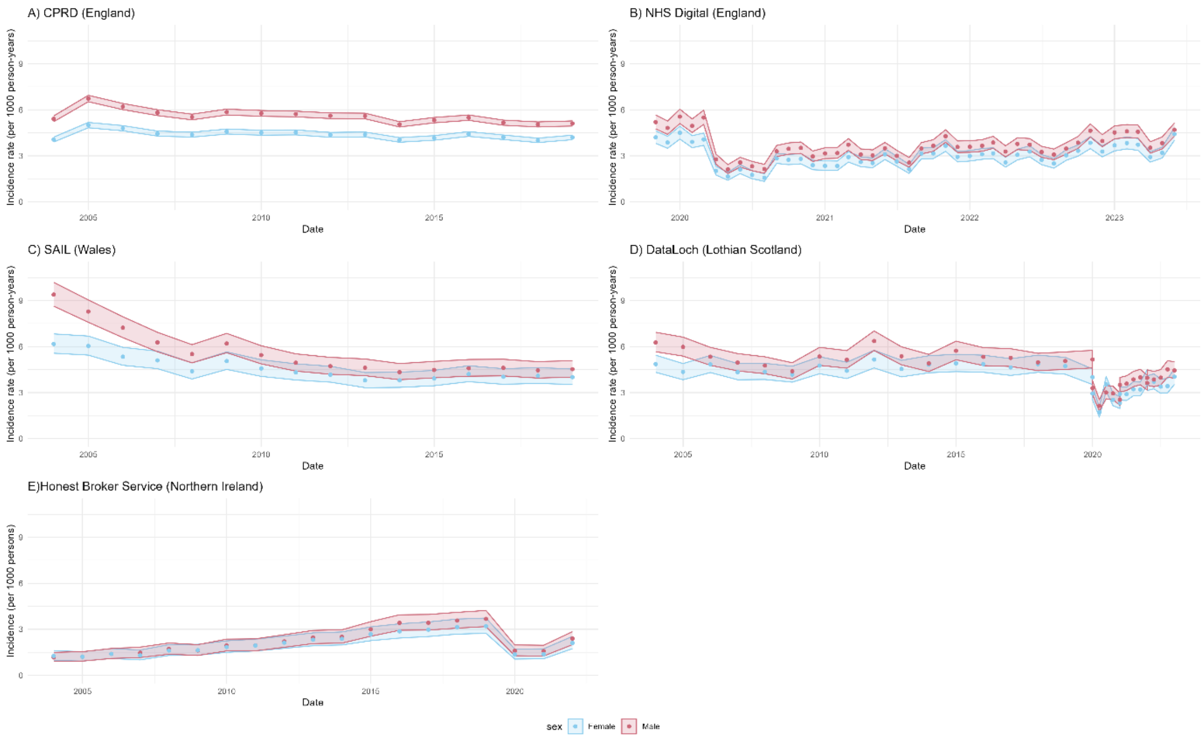

Legend: Estimates illustrate incidence rates of COPD per 1000 person-years and 95% confidence intervals. Yearly incidence rates were calculated for the years 2004 up until the end of 2019 for CPRD, SAIL and DataLoch, monthly incidence rates were calculated from November 2019 up until June 2023 for NHSE, and 3-monthly incidence rates were calculated from January 2020 up until March 2023. Incidence per 1000 persons was calculated for NI for the year 2004 to 2022.

Figure S6: Sex adjusted incidence rates of COPD by age

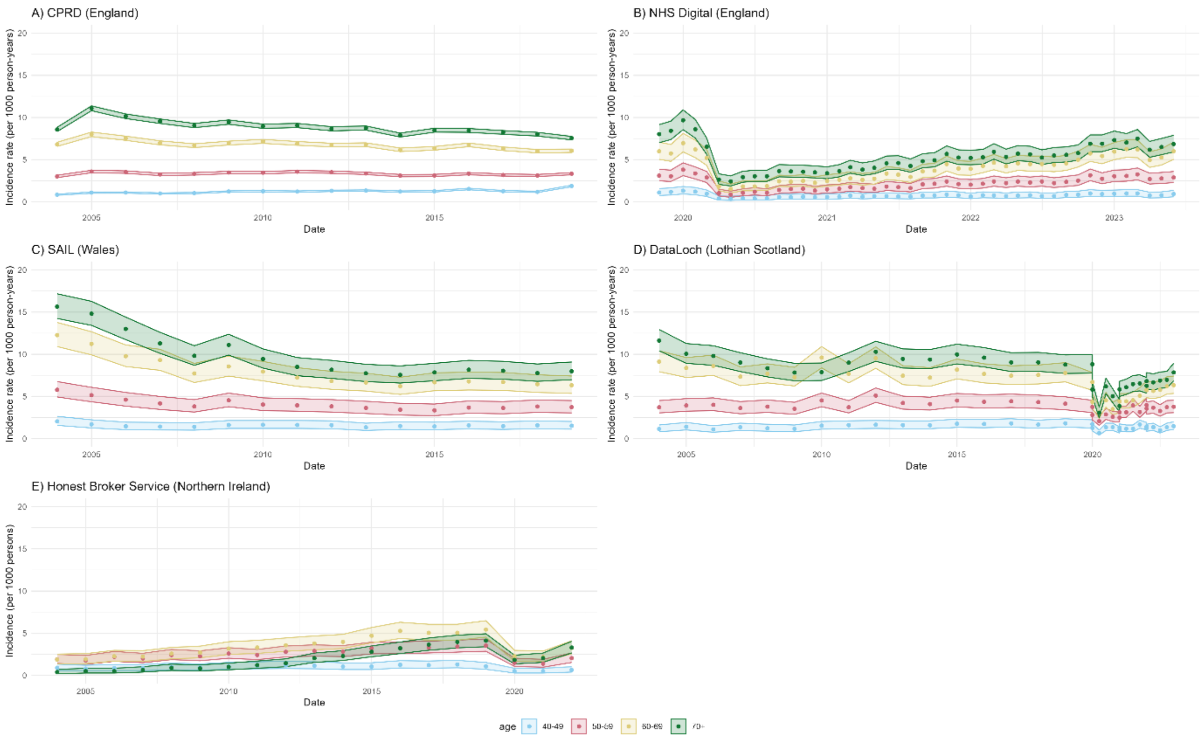

Legend: Estimates illustrate incidence rates of COPD per 1000 person-years and 95% confidence intervals. Yearly incidence rates were calculated for the years 2004 up until the end of 2019 for CPRD, SAIL and DataLoch, monthly incidence rates were calculated from November 2019 up until June 2023 for NHSE, and 3-monthly incidence rates were calculated from January 2020 up until March 2023. Incidence per 1000 persons was calculated for NI for the year 2004 to 2022.

Figure S7: Age adjusted incidence rates of ILD by sex

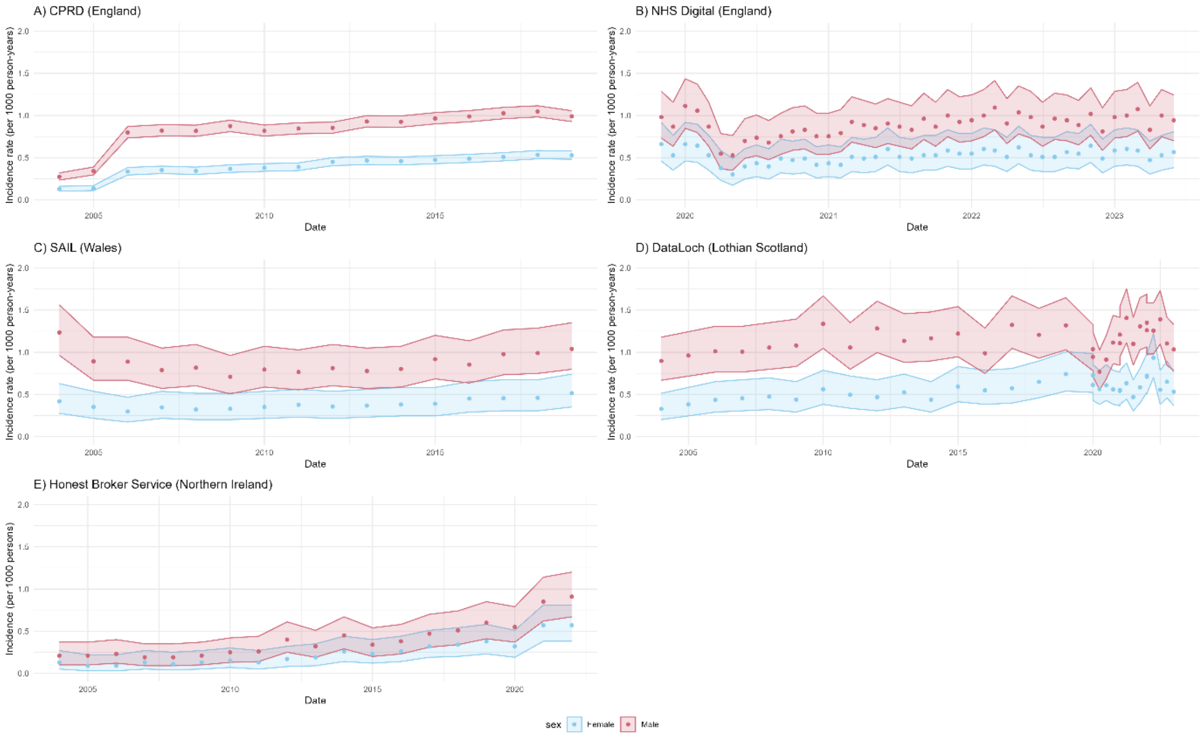

Legend: Estimates illustrate incidence rates of ILD per 1000 person-years and 95% confidence intervals. Yearly incidence rates were calculated for the years 2004 up until the end of 2019 for CPRD, SAIL and DataLoch, monthly incidence rates were calculated from November 2019 up until June 2023 for NHSE, and 3-monthly incidence rates were calculated from January 2020 up until March 2023. Incidence per 1000 persons was calculated for NI for the year 2004 to 2022.

Figure S8: Sex adjusted incidence rates of ILD by age

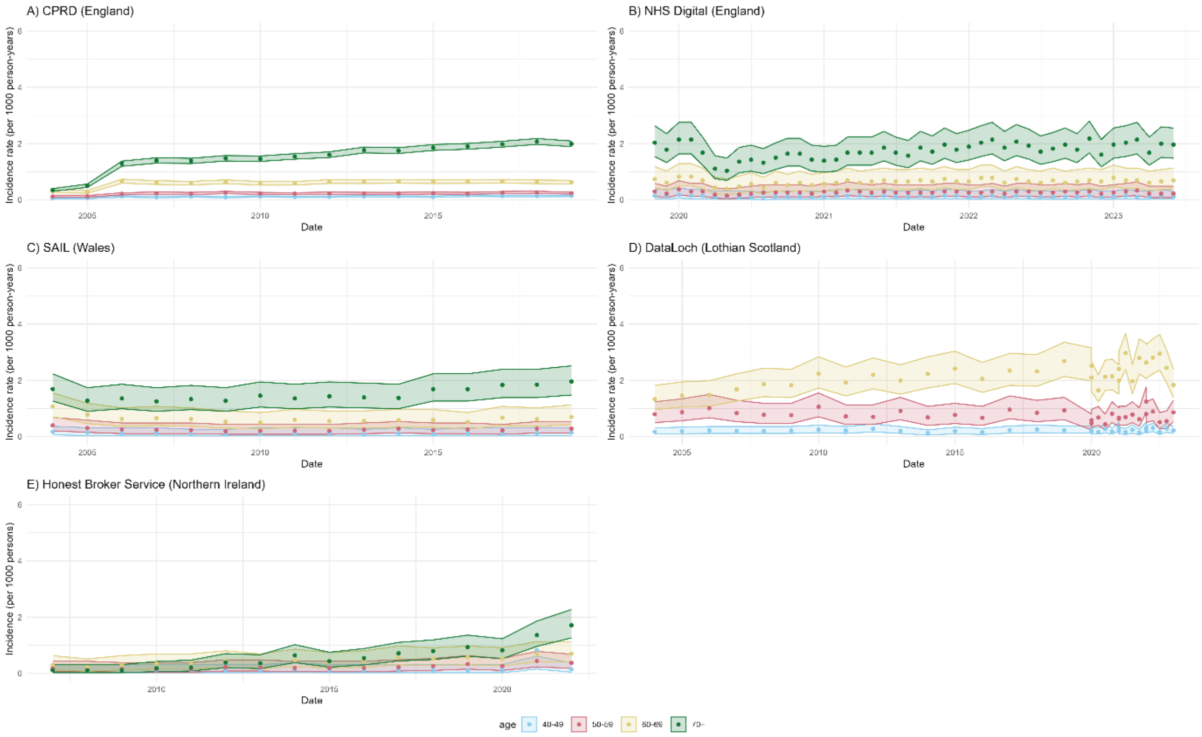

Legend: Estimates illustrate incidence rates of ILD per 1000 person-years and 95% confidence intervals. Yearly incidence rates were calculated for the years 2004 up until the end of 2019 for CPRD, SAIL and DataLoch, monthly incidence rates were calculated from November 2019 up until June 2023 for NHSE, and 3-monthly incidence rates were calculated from January 2020 up until March 2023. Incidence per 1000 persons was calculated for NI for the year 2004 to 2022.

Figure S9: Age and sex adjusted incidence rates of COPD, asthma, and ILD by region in England

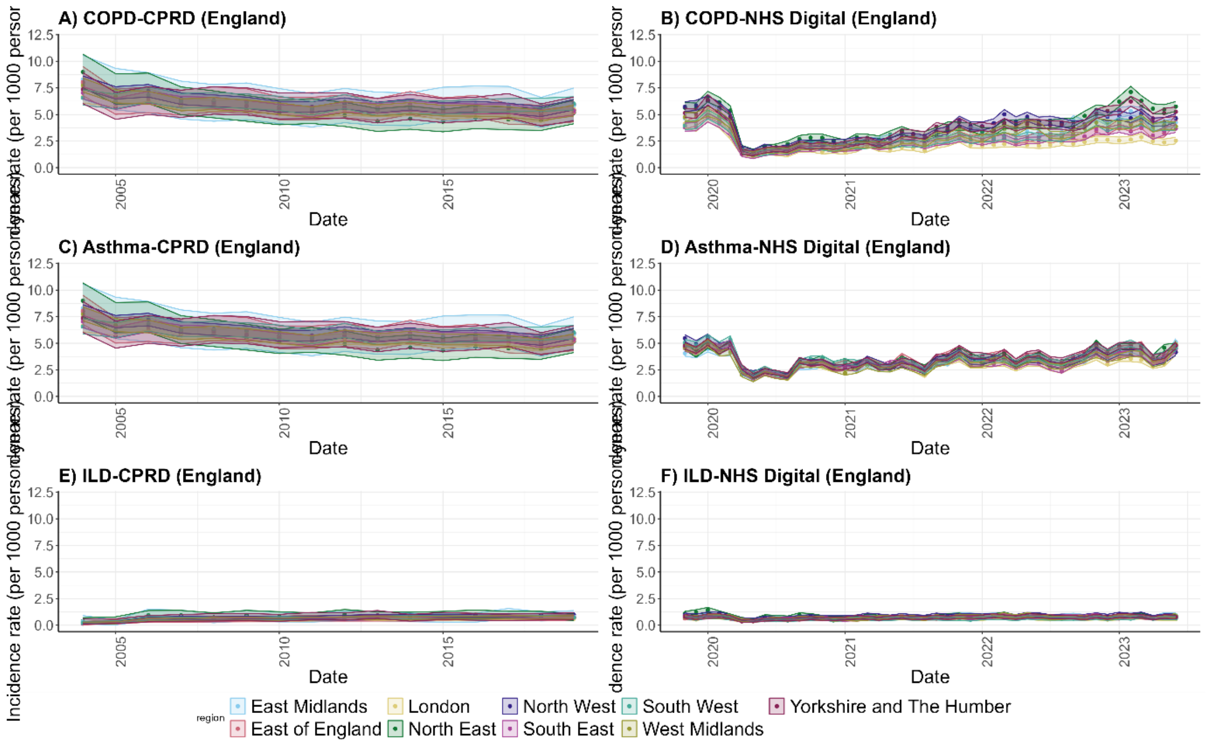

Legend: Estimates illustrate incidence rates of asthma, COPD and ILD per 1000 person-years and 95% confidence intervals. Yearly incidence rates were calculated for the years 2004 up until the end of 2020 for CPRD. Monthly incidence rates were calculated from November 2019 up until June 2023 for NHSE. COPD (chronic obstructive pulmonary disease), ILD (interstitial lung disease).

Figure S10: Age and sex adjusted incidence rates of COPD, asthma, and ILD by IMD in NHSE SDE, England

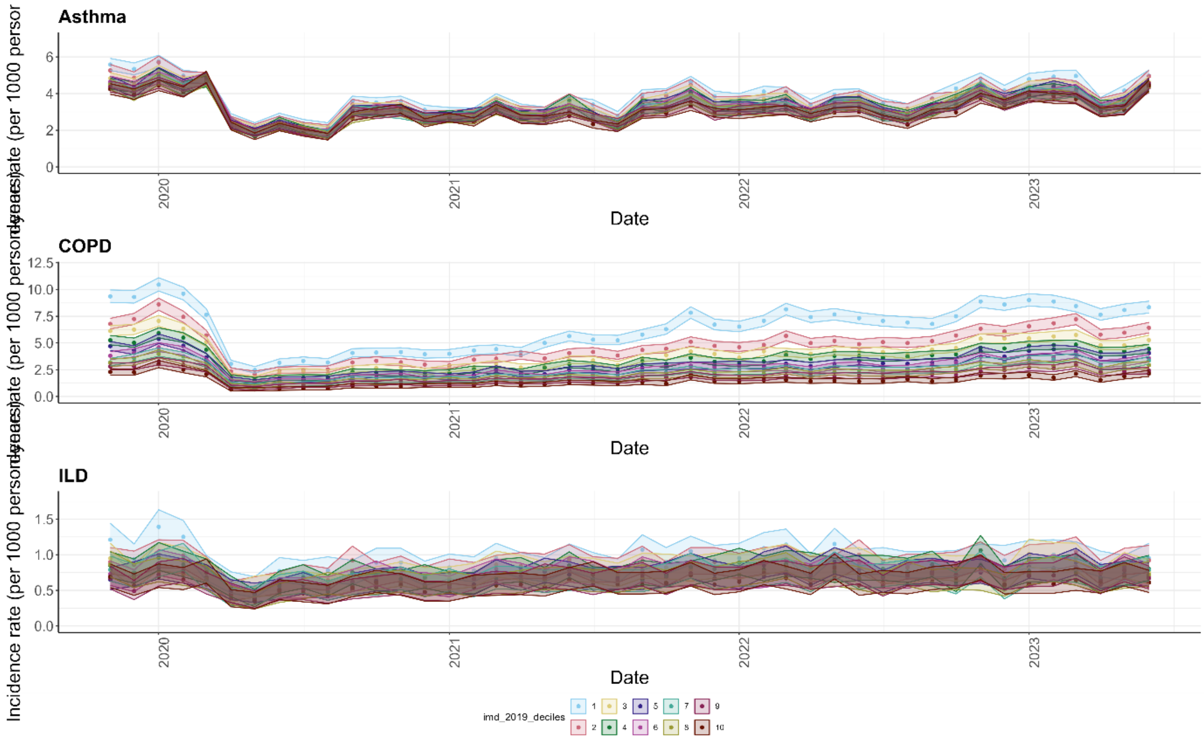

Legend: Estimates illustrate incidence rates of asthma, COPD and ILD per 1000 person-years and 95% confidence intervals. Monthly incidence rates were calculated from November 2019 up until June 2023 for NHSE. COPD (chronic obstructive pulmonary disease), ILD (interstitial lung disease).

Figure S11: Age and sex adjusted incidence rates of COPD, asthma, and ILD by ethnicity in NHSE SDE, England

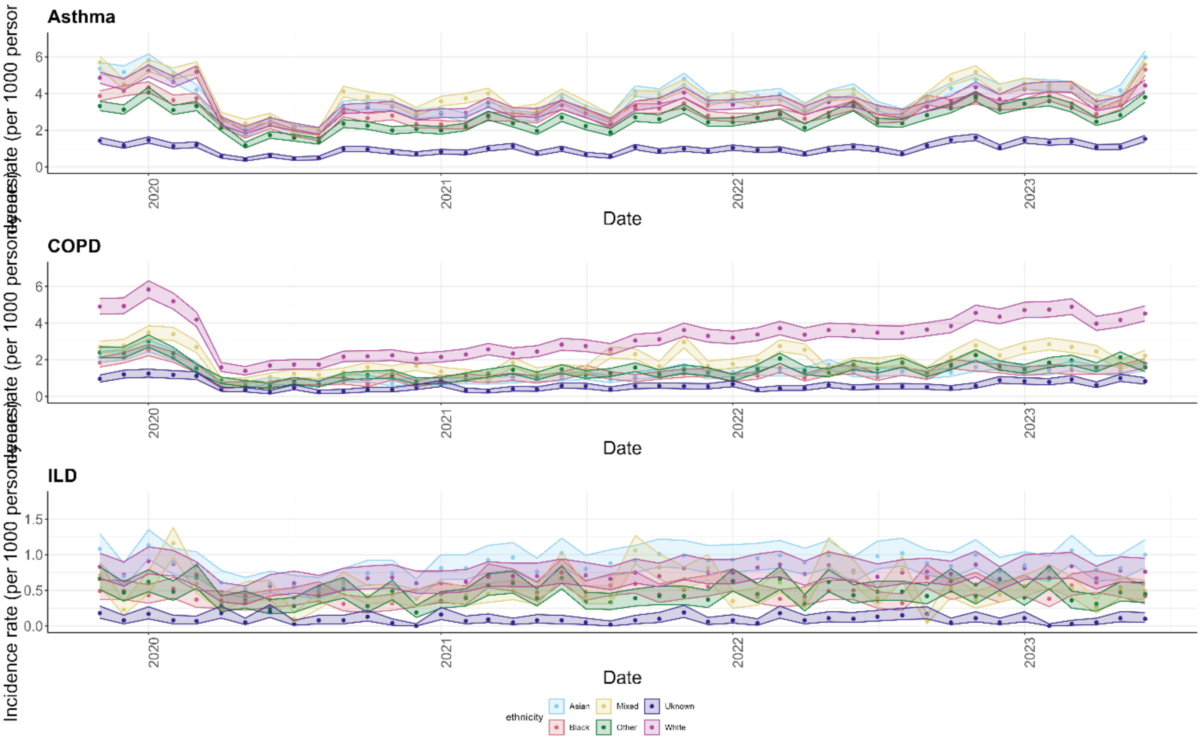

Legend: Estimates illustrate incidence rates of asthma, COPD and ILD per 1000 person-years and 95% confidence intervals. Monthly incidence rates were calculated from November 2019 up until June 2023 for NHSE. COPD (chronic obstructive pulmonary disease), ILD (interstitial lung disease).

Figure S12: Observed vs. projected incidence rates of asthma, COPD, and ILD during the pandemic.

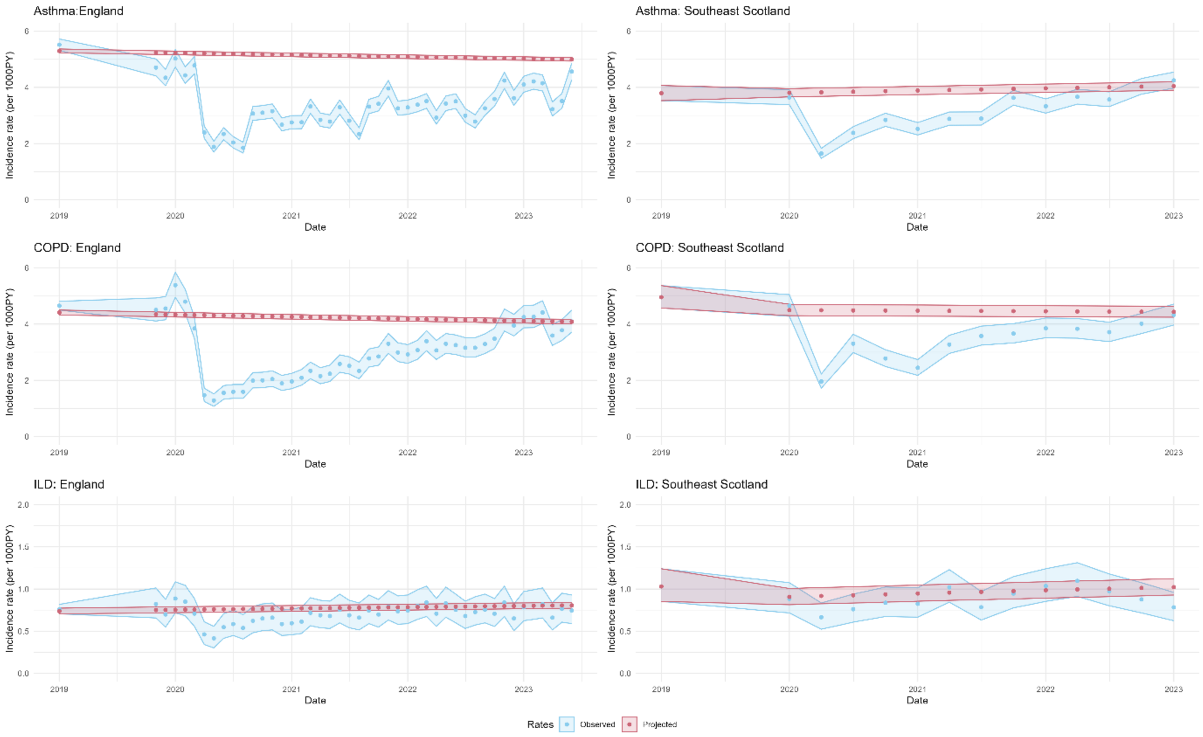

Table S1: Harmonisation methods for key variables across CPRD, SAIL, DataLoch, NHSE, and HBS

Key variable	CPRD Aurum	SAIL Databank	DataLoch	NHSE	Honest Broker Service	Harmonisation approach
Eligible follow-up period	Unique patient identifier for each GP registration period	Unique patient identifier	Unique patient identifier	Unique patient identifier	Unique patient identifier	In CPRD, eligible follow-up period was registration start date to earliest of: death date, last practice collection date, and registration end date to avoid counting duplicates. All GP codes outside the registration period were not used in CPRD. In HBS, eligible follow-up period was registration start date to earliest of: end of study period or when they were assumed to be dead or no longer living in NI (defined as one year from their last interaction with healthcare services).
Date of birth	Year of birth available	Week & year of birth available	Full date of birth available but month & year of birth used for research	Month & year of birth available	Month & year of birth available	In CPRD, date of birth was set to 1st July of that year. In SAIL, the Monday of the week of birth is used as the date of birth. When month and year of birth given in DataLoch, HBS, & NHSE, date of birth is set to 1st of that month.
Coding systems for diagnoses	Medcodeid used in GP data	5-character Read V2 and V3 codes used in GP data	7-character Scottish Read V2 codes used in GP data	SNOMED CT codes used in GP data	SNOMED CT codes used in GP data	All codelists have medcodeid (CPRD Aurum proprietary medical code identifier) corresponding to the equivalent SNOMED-CT concept and description IDs (with complete coverage) and Read code (only where available) using the CPRD Aurum medical dictionary.
Data preparation	File-based	SQL database	SQL database	SQL or Python database	File-based	Scripts coded in Stata and R in CPRD and HBS, SQL and R in SAIL and DataLoch, and Python and Stata for NHSE. R scripts shared across databases where appropriate.

Table S2: Crude IRR or IR for incidence of asthma, COPD, and ILD in 2019 vs 2005 for England (CPRD), Wales, Scotland, and Northern Ireland, and in June 2023 vs November 2019 for England (NHSE SDE)

**Nation**	**Condition**	**IRR (95% CI) or IR (95% CI)**
England (CPRD)	Asthma	0.89 (0.88-0.90)
	COPD	0.83 (0.82-0.85)
	ILD	3.27 (3.05-3.50)
Wales (SAIL)	Asthma	0.66 (0.65-0.68)
	COPD	0.67 (0.65-0.69)
	ILD	1.39 (1.27-1.53)
Scotland (DataLoch)	Asthma	0.67 (0.64-0.71)
	COPD	1.06 (0.99-1.13)
	ILD	1.63 (1.36-1.95)
Northern Ireland (Honest Broker Service)	Asthma	0.75 (0.72-0.79)
	COPD	2.77 (2.51-3.07)
	ILD	2.76 (2.10-3.64)
England (NHSE SDE)	Asthma	1.00 (0.98-1.02)
	COPD	0.91 (0.89-0.94)
	ILD	1.09 (1.02-1.17)

Legend: IR was calculated for NI and IRR were calculated for England (CPRD and NHSE), Wales, and Scotland.

Table S3: Crude OR for prevalence of asthma, COPD, and ILD in 2019 vs 2005 for England (CPRD), Wales, and Scotland, and in 2019 vs 2011 for Northern Ireland (Honest Broker Service)

**Nation**	**Condition**	**OR (95% CI)**
England (CPRD)	Asthma	2.37 (2.34-2.40)
	COPD	1.54 (1.51-1.56)
	ILD	4.07 (3.82-4.33)
Wales (SAIL)	Asthma	1.40 (1.40-1.41)
	COPD	1.92 (1.90-1.94)
	ILD	1.40 (1.35-1.45)
Northern Ireland (Honest Broker Service)	Asthma	1.10 (1.09-1.11)
	COPD	1.85 (1.82-1.89)
	ILD	1.85 (1.73-1.97)
Scotland (DataLoch)	Asthma	1.18 (1.17-1.20)
	COPD	1.34 (1.31-1.37)
	ILD	1.63 (1.52-1.75)

References:

1. Hatam S, scully S, Cook S, et al. A harmonised approach to curating research-ready datasets for Asthma, Chronic Obstructive Pulmonary Disease (COPD) and Interstitial Lung Disease (ILD) in England, Wales and Scotland using Clinical Practice Research Datalink (CPRD), Secure Anonymised Information Linkage (SAIL) Databank and DataLoch. *Clinical Epidemiology* 2024.
2. Eurostat. Revision of the European Standard Population, 2013.
