# Supplementary material for: Incidence and prevalence of asthma, chronic obstructive pulmonary disease and interstitial lung disease between 2004 and 2023: harmonised analyses of longitudinal cohorts across England, Wales, South-East Scotland and Northern Ireland
Source: Thorax. 2025 Apr 8;80(7):e222699. doi: 10.1136/thorax-2024-222699 (PMC12322415; doi:10.1136/thorax-2024-222699)
Supplement: online supplemental file 2 [file thorax-80-7-s002.pdf]

nation	condition	type	year	sex	age	region	overall_numerator
England_NHSE	Asthma	all	01/11/2019				21710
England_NHSE	Asthma	all	01/12/2019				20585
England_NHSE	Asthma	all	01/01/2020				23640
England_NHSE	Asthma	all	01/02/2020				19480
England_NHSE	Asthma	all	01/03/2020				22550
England_NHSE	Asthma	all	01/04/2020				10810
England_NHSE	Asthma	all	01/05/2020				8735
England_NHSE	Asthma	all	01/06/2020				10585
England_NHSE	Asthma	all	01/07/2020				9445
England_NHSE	Asthma	all	01/08/2020				8575
England_NHSE	Asthma	all	01/09/2020				14040
England_NHSE	Asthma	all	01/10/2020				14570
England_NHSE	Asthma	all	01/11/2020				14260
England_NHSE	Asthma	all	01/12/2020				12435
England_NHSE	Asthma	all	01/01/2021				12705
England_NHSE	Asthma	all	01/02/2021				11475
England_NHSE	Asthma	all	01/03/2021				15385
England_NHSE	Asthma	all	01/04/2021				12735
England_NHSE	Asthma	all	01/05/2021				12790
England_NHSE	Asthma	all	01/06/2021				14655
England_NHSE	Asthma	all	01/07/2021				12820
England_NHSE	Asthma	all	01/08/2021				10605
England_NHSE	Asthma	all	01/09/2021				14525
England_NHSE	Asthma	all	01/10/2021				15425
England_NHSE	Asthma	all	01/11/2021				17270
England_NHSE	Asthma	all	01/12/2021				14625
England_NHSE	Asthma	all	01/01/2022				14715
England_NHSE	Asthma	all	01/02/2022				13560
England_NHSE	Asthma	all	01/03/2022				15500
England_NHSE	Asthma	all	01/04/2022				12455
England_NHSE	Asthma	all	01/05/2022				14990
England_NHSE	Asthma	all	01/06/2022				14995
England_NHSE	Asthma	all	01/07/2022				13125
England_NHSE	Asthma	all	01/08/2022				12115
England_NHSE	Asthma	all	01/09/2022				13635
England_NHSE	Asthma	all	01/10/2022				15525
England_NHSE	Asthma	all	01/11/2022				17690
England_NHSE	Asthma	all	01/12/2022				15645
England_NHSE	Asthma	all	01/01/2023				17705
England_NHSE	Asthma	all	01/02/2023				16300
England_NHSE	Asthma	all	01/03/2023				17675
England_NHSE	Asthma	all	01/04/2023				13340
England_NHSE	Asthma	all	01/05/2023				14890
England_NHSE	Asthma	all	01/06/2023				18540
England_NHSE	Asthma	sex_strat_children	01/11/2019	Male			5010
England_NHSE	Asthma	sex_strat_children	01/11/2019	Female			4135
England_NHSE	Asthma	sex_strat_children	01/12/2019	Male			4420
England_NHSE	Asthma	sex_strat_children	01/12/2019	Female			3910
England_NHSE	Asthma	sex_strat_children	01/01/2020	Male			4645
England_NHSE	Asthma	sex_strat_children	01/01/2020	Female			4180
England_NHSE	Asthma	sex_strat_children	01/02/2020	Male			3845
England_NHSE	Asthma	sex_strat_children	01/02/2020	Female			3660
England_NHSE	Asthma	sex_strat_children	01/03/2020	Male			4135
England_NHSE	Asthma	sex_strat_children	01/03/2020	Female			3645
England_NHSE	Asthma	sex_strat_children	01/04/2020	Male			1660
England_NHSE	Asthma	sex_strat_children	01/04/2020	Female			1500
England_NHSE	Asthma	sex_strat_children	01/05/2020	Male			1365
England_NHSE	Asthma	sex_strat_children	01/05/2020	Female			1155
England_NHSE	Asthma	sex_strat_children	01/06/2020	Male			1755
England_NHSE	Asthma	sex_strat_children	01/06/2020	Female			1330
England_NHSE	Asthma	sex_strat_children	01/07/2020	Male			1435
England_NHSE	Asthma	sex_strat_children	01/07/2020	Female			1145

England_NHSE	Asthma	sex_strat_children	01/08/2020	Male	1375
England_NHSE	Asthma	sex_strat_children	01/08/2020	Female	1135
England_NHSE	Asthma	sex_strat_children	01/09/2020	Male	3600
England_NHSE	Asthma	sex_strat_children	01/09/2020	Female	2615
England_NHSE	Asthma	sex_strat_children	01/10/2020	Male	3195
England_NHSE	Asthma	sex_strat_children	01/10/2020	Female	2605
England_NHSE	Asthma	sex_strat_children	01/11/2020	Male	3055
England_NHSE	Asthma	sex_strat_children	01/11/2020	Female	2305
England_NHSE	Asthma	sex_strat_children	01/12/2020	Male	2535
England_NHSE	Asthma	sex_strat_children	01/12/2020	Female	2005
England_NHSE	Asthma	sex_strat_children	01/01/2021	Male	1915
England_NHSE	Asthma	sex_strat_children	01/01/2021	Female	1630
England_NHSE	Asthma	sex_strat_children	01/02/2021	Male	1495
England_NHSE	Asthma	sex_strat_children	01/02/2021	Female	1280
England_NHSE	Asthma	sex_strat_children	01/03/2021	Male	2415
England_NHSE	Asthma	sex_strat_children	01/03/2021	Female	2015
England_NHSE	Asthma	sex_strat_children	01/04/2021	Male	2145
England_NHSE	Asthma	sex_strat_children	01/04/2021	Female	1775
England_NHSE	Asthma	sex_strat_children	01/05/2021	Male	2560
England_NHSE	Asthma	sex_strat_children	01/05/2021	Female	2080
England_NHSE	Asthma	sex_strat_children	01/06/2021	Male	3135
England_NHSE	Asthma	sex_strat_children	01/06/2021	Female	2495
England_NHSE	Asthma	sex_strat_children	01/07/2021	Male	2825
England_NHSE	Asthma	sex_strat_children	01/07/2021	Female	2215
England_NHSE	Asthma	sex_strat_children	01/08/2021	Male	2050
England_NHSE	Asthma	sex_strat_children	01/08/2021	Female	1670
England_NHSE	Asthma	sex_strat_children	01/09/2021	Male	3740
England_NHSE	Asthma	sex_strat_children	01/09/2021	Female	2835
England_NHSE	Asthma	sex_strat_children	01/10/2021	Male	3945
England_NHSE	Asthma	sex_strat_children	01/10/2021	Female	3215
England_NHSE	Asthma	sex_strat_children	01/11/2021	Male	4455
England_NHSE	Asthma	sex_strat_children	01/11/2021	Female	3550
England_NHSE	Asthma	sex_strat_children	01/12/2021	Male	3570
England_NHSE	Asthma	sex_strat_children	01/12/2021	Female	3065
England_NHSE	Asthma	sex_strat_children	01/01/2022	Male	3425
England_NHSE	Asthma	sex_strat_children	01/01/2022	Female	2840
England_NHSE	Asthma	sex_strat_children	01/02/2022	Male	3160
England_NHSE	Asthma	sex_strat_children	01/02/2022	Female	2725
England_NHSE	Asthma	sex_strat_children	01/03/2022	Male	3615
England_NHSE	Asthma	sex_strat_children	01/03/2022	Female	3090
England_NHSE	Asthma	sex_strat_children	01/04/2022	Male	2645
England_NHSE	Asthma	sex_strat_children	01/04/2022	Female	2445
England_NHSE	Asthma	sex_strat_children	01/05/2022	Male	3525
England_NHSE	Asthma	sex_strat_children	01/05/2022	Female	3005
England_NHSE	Asthma	sex_strat_children	01/06/2022	Male	3335
England_NHSE	Asthma	sex_strat_children	01/06/2022	Female	2755
England_NHSE	Asthma	sex_strat_children	01/07/2022	Male	2920
England_NHSE	Asthma	sex_strat_children	01/07/2022	Female	2510
England_NHSE	Asthma	sex_strat_children	01/08/2022	Male	2315
England_NHSE	Asthma	sex_strat_children	01/08/2022	Female	2040
England_NHSE	Asthma	sex_strat_children	01/09/2022	Male	3250
England_NHSE	Asthma	sex_strat_children	01/09/2022	Female	2655
England_NHSE	Asthma	sex_strat_children	01/10/2022	Male	3630
England_NHSE	Asthma	sex_strat_children	01/10/2022	Female	3045
England_NHSE	Asthma	sex_strat_children	01/11/2022	Male	4015
England_NHSE	Asthma	sex_strat_children	01/11/2022	Female	3420
England_NHSE	Asthma	sex_strat_children	01/12/2022	Male	3450
England_NHSE	Asthma	sex_strat_children	01/12/2022	Female	2855
England_NHSE	Asthma	sex_strat_children	01/01/2023	Male	3690
England_NHSE	Asthma	sex_strat_children	01/01/2023	Female	3255
England_NHSE	Asthma	sex_strat_children	01/02/2023	Male	3490
England_NHSE	Asthma	sex_strat_children	01/02/2023	Female	2995
England_NHSE	Asthma	sex_strat_children	01/03/2023	Male	3645

England_NHSE	Asthma	sex_strat_children	01/03/2023	Female	3270
England_NHSE	Asthma	sex_strat_children	01/04/2023	Male	2725
England_NHSE	Asthma	sex_strat_children	01/04/2023	Female	2450
England_NHSE	Asthma	sex_strat_children	01/05/2023	Male	3140
England_NHSE	Asthma	sex_strat_children	01/05/2023	Female	2670
England_NHSE	Asthma	sex_strat_children	01/06/2023	Male	3840
England_NHSE	Asthma	sex_strat_children	01/06/2023	Female	2910
England_NHSE	Asthma	sex_strat_adults	01/11/2019	Male	4900
England_NHSE	Asthma	sex_strat_adults	01/11/2019	Female	7670
England_NHSE	Asthma	sex_strat_adults	01/12/2019	Male	4885
England_NHSE	Asthma	sex_strat_adults	01/12/2019	Female	7370
England_NHSE	Asthma	sex_strat_adults	01/01/2020	Male	6075
England_NHSE	Asthma	sex_strat_adults	01/01/2020	Female	8745
England_NHSE	Asthma	sex_strat_adults	01/02/2020	Male	4875
England_NHSE	Asthma	sex_strat_adults	01/02/2020	Female	7100
England_NHSE	Asthma	sex_strat_adults	01/03/2020	Male	5595
England_NHSE	Asthma	sex_strat_adults	01/03/2020	Female	9175
England_NHSE	Asthma	sex_strat_adults	01/04/2020	Male	2965
England_NHSE	Asthma	sex_strat_adults	01/04/2020	Female	4685
England_NHSE	Asthma	sex_strat_adults	01/05/2020	Male	2495
England_NHSE	Asthma	sex_strat_adults	01/05/2020	Female	3720
England_NHSE	Asthma	sex_strat_adults	01/06/2020	Male	3115
England_NHSE	Asthma	sex_strat_adults	01/06/2020	Female	4385
England_NHSE	Asthma	sex_strat_adults	01/07/2020	Male	2705
England_NHSE	Asthma	sex_strat_adults	01/07/2020	Female	4160
England_NHSE	Asthma	sex_strat_adults	01/08/2020	Male	2305
England_NHSE	Asthma	sex_strat_adults	01/08/2020	Female	3760
England_NHSE	Asthma	sex_strat_adults	01/09/2020	Male	2995
England_NHSE	Asthma	sex_strat_adults	01/09/2020	Female	4830
England_NHSE	Asthma	sex_strat_adults	01/10/2020	Male	3345
England_NHSE	Asthma	sex_strat_adults	01/10/2020	Female	5420
England_NHSE	Asthma	sex_strat_adults	01/11/2020	Male	3350
England_NHSE	Asthma	sex_strat_adults	01/11/2020	Female	5545
England_NHSE	Asthma	sex_strat_adults	01/12/2020	Male	3085
England_NHSE	Asthma	sex_strat_adults	01/12/2020	Female	4810
England_NHSE	Asthma	sex_strat_adults	01/01/2021	Male	3560
England_NHSE	Asthma	sex_strat_adults	01/01/2021	Female	5600
England_NHSE	Asthma	sex_strat_adults	01/02/2021	Male	3415
England_NHSE	Asthma	sex_strat_adults	01/02/2021	Female	5285
England_NHSE	Asthma	sex_strat_adults	01/03/2021	Male	4400
England_NHSE	Asthma	sex_strat_adults	01/03/2021	Female	6550
England_NHSE	Asthma	sex_strat_adults	01/04/2021	Male	3710
England_NHSE	Asthma	sex_strat_adults	01/04/2021	Female	5100
England_NHSE	Asthma	sex_strat_adults	01/05/2021	Male	3290
England_NHSE	Asthma	sex_strat_adults	01/05/2021	Female	4860
England_NHSE	Asthma	sex_strat_adults	01/06/2021	Male	3820
England_NHSE	Asthma	sex_strat_adults	01/06/2021	Female	5205
England_NHSE	Asthma	sex_strat_adults	01/07/2021	Male	3210
England_NHSE	Asthma	sex_strat_adults	01/07/2021	Female	4565
England_NHSE	Asthma	sex_strat_adults	01/08/2021	Male	2790
England_NHSE	Asthma	sex_strat_adults	01/08/2021	Female	4090
England_NHSE	Asthma	sex_strat_adults	01/09/2021	Male	3180
England_NHSE	Asthma	sex_strat_adults	01/09/2021	Female	4770
England_NHSE	Asthma	sex_strat_adults	01/10/2021	Male	3205
England_NHSE	Asthma	sex_strat_adults	01/10/2021	Female	5055
England_NHSE	Asthma	sex_strat_adults	01/11/2021	Male	3515
England_NHSE	Asthma	sex_strat_adults	01/11/2021	Female	5750
England_NHSE	Asthma	sex_strat_adults	01/12/2021	Male	3025
England_NHSE	Asthma	sex_strat_adults	01/12/2021	Female	4965
England_NHSE	Asthma	sex_strat_adults	01/01/2022	Male	3275
England_NHSE	Asthma	sex_strat_adults	01/01/2022	Female	5175
England_NHSE	Asthma	sex_strat_adults	01/02/2022	Male	3065
England_NHSE	Asthma	sex_strat_adults	01/02/2022	Female	4610

England_NHSE	Asthma	sex_strat_adults	01/03/2022	Male		3355
England_NHSE	Asthma	sex_strat_adults	01/03/2022	Female		5440
England_NHSE	Asthma	sex_strat_adults	01/04/2022	Male		2835
England_NHSE	Asthma	sex_strat_adults	01/04/2022	Female		4535
England_NHSE	Asthma	sex_strat_adults	01/05/2022	Male		3180
England_NHSE	Asthma	sex_strat_adults	01/05/2022	Female		5280
England_NHSE	Asthma	sex_strat_adults	01/06/2022	Male		3710
England_NHSE	Asthma	sex_strat_adults	01/06/2022	Female		5200
England_NHSE	Asthma	sex_strat_adults	01/07/2022	Male		3080
England_NHSE	Asthma	sex_strat_adults	01/07/2022	Female		4615
England_NHSE	Asthma	sex_strat_adults	01/08/2022	Male		3080
England_NHSE	Asthma	sex_strat_adults	01/08/2022	Female		4680
England_NHSE	Asthma	sex_strat_adults	01/09/2022	Male		3055
England_NHSE	Asthma	sex_strat_adults	01/09/2022	Female		4675
England_NHSE	Asthma	sex_strat_adults	01/10/2022	Male		3515
England_NHSE	Asthma	sex_strat_adults	01/10/2022	Female		5335
England_NHSE	Asthma	sex_strat_adults	01/11/2022	Male		3930
England_NHSE	Asthma	sex_strat_adults	01/11/2022	Female		6325
England_NHSE	Asthma	sex_strat_adults	01/12/2022	Male		3570
England_NHSE	Asthma	sex_strat_adults	01/12/2022	Female		5765
England_NHSE	Asthma	sex_strat_adults	01/01/2023	Male		4220
England_NHSE	Asthma	sex_strat_adults	01/01/2023	Female		6535
England_NHSE	Asthma	sex_strat_adults	01/02/2023	Male		3885
England_NHSE	Asthma	sex_strat_adults	01/02/2023	Female		5925
England_NHSE	Asthma	sex_strat_adults	01/03/2023	Male		4250
England_NHSE	Asthma	sex_strat_adults	01/03/2023	Female		6510
England_NHSE	Asthma	sex_strat_adults	01/04/2023	Male		3285
England_NHSE	Asthma	sex_strat_adults	01/04/2023	Female		4880
England_NHSE	Asthma	sex_strat_adults	01/05/2023	Male		3600
England_NHSE	Asthma	sex_strat_adults	01/05/2023	Female		5480
England_NHSE	Asthma	sex_strat_adults	01/06/2023	Male		5160
England_NHSE	Asthma	sex_strat_adults	01/06/2023	Female		6630
England_NHSE	Asthma	age_strat	01/11/2019		0-4	2745
England_NHSE	Asthma	age_strat	01/11/2019		5-9	3190
England_NHSE	Asthma	age_strat	01/11/2019		10-14	2075
England_NHSE	Asthma	age_strat	01/11/2019		15-19	1130
England_NHSE	Asthma	age_strat	01/11/2019		20-29	2280
England_NHSE	Asthma	age_strat	01/11/2019		30-39	2185
England_NHSE	Asthma	age_strat	01/11/2019		40-49	2090
England_NHSE	Asthma	age_strat	01/11/2019		50-59	2255
England_NHSE	Asthma	age_strat	01/11/2019		60-69	1845
England_NHSE	Asthma	age_strat	01/11/2019		70+	1915
England_NHSE	Asthma	age_strat	01/12/2019		0-4	2495
England_NHSE	Asthma	age_strat	01/12/2019		5-9	2915
England_NHSE	Asthma	age_strat	01/12/2019		10-14	1945
England_NHSE	Asthma	age_strat	01/12/2019		15-19	970
England_NHSE	Asthma	age_strat	01/12/2019		20-29	2145
England_NHSE	Asthma	age_strat	01/12/2019		30-39	2040
England_NHSE	Asthma	age_strat	01/12/2019		40-49	2075
England_NHSE	Asthma	age_strat	01/12/2019		50-59	2220
England_NHSE	Asthma	age_strat	01/12/2019		60-69	1760
England_NHSE	Asthma	age_strat	01/12/2019		70+	2020
England_NHSE	Asthma	age_strat	01/01/2020		0-4	2430
England_NHSE	Asthma	age_strat	01/01/2020		5-9	3265
England_NHSE	Asthma	age_strat	01/01/2020		10-14	1965
England_NHSE	Asthma	age_strat	01/01/2020		15-19	1165
England_NHSE	Asthma	age_strat	01/01/2020		20-29	2600
England_NHSE	Asthma	age_strat	01/01/2020		30-39	2445
England_NHSE	Asthma	age_strat	01/01/2020		40-49	2475
England_NHSE	Asthma	age_strat	01/01/2020		50-59	2655
England_NHSE	Asthma	age_strat	01/01/2020		60-69	2205
England_NHSE	Asthma	age_strat	01/01/2020		70+	2445
England_NHSE	Asthma	age_strat	01/02/2020		0-4	1950

England_NHSE	Asthma	age_strat	01/02/2020	5-9	2795
England_NHSE	Asthma	age_strat	01/02/2020	10-14	1810
England_NHSE	Asthma	age_strat	01/02/2020	15-19	955
England_NHSE	Asthma	age_strat	01/02/2020	20-29	2055
England_NHSE	Asthma	age_strat	01/02/2020	30-39	2065
England_NHSE	Asthma	age_strat	01/02/2020	40-49	2000
England_NHSE	Asthma	age_strat	01/02/2020	50-59	2165
England_NHSE	Asthma	age_strat	01/02/2020	60-69	1845
England_NHSE	Asthma	age_strat	01/02/2020	70+	1850
England_NHSE	Asthma	age_strat	01/03/2020	0-4	1960
England_NHSE	Asthma	age_strat	01/03/2020	5-9	2885
England_NHSE	Asthma	age_strat	01/03/2020	10-14	1820
England_NHSE	Asthma	age_strat	01/03/2020	15-19	1110
England_NHSE	Asthma	age_strat	01/03/2020	20-29	2960
England_NHSE	Asthma	age_strat	01/03/2020	30-39	2970
England_NHSE	Asthma	age_strat	01/03/2020	40-49	2700
England_NHSE	Asthma	age_strat	01/03/2020	50-59	2690
England_NHSE	Asthma	age_strat	01/03/2020	60-69	1860
England_NHSE	Asthma	age_strat	01/03/2020	70+	1595
England_NHSE	Asthma	age_strat	01/04/2020	0-4	795
England_NHSE	Asthma	age_strat	01/04/2020	5-9	1190
England_NHSE	Asthma	age_strat	01/04/2020	10-14	715
England_NHSE	Asthma	age_strat	01/04/2020	15-19	465
England_NHSE	Asthma	age_strat	01/04/2020	20-29	1400
England_NHSE	Asthma	age_strat	01/04/2020	30-39	1555
England_NHSE	Asthma	age_strat	01/04/2020	40-49	1475
England_NHSE	Asthma	age_strat	01/04/2020	50-59	1425
England_NHSE	Asthma	age_strat	01/04/2020	60-69	925
England_NHSE	Asthma	age_strat	01/04/2020	70+	875
England_NHSE	Asthma	age_strat	01/05/2020	0-4	655
England_NHSE	Asthma	age_strat	01/05/2020	5-9	950
England_NHSE	Asthma	age_strat	01/05/2020	10-14	535
England_NHSE	Asthma	age_strat	01/05/2020	15-19	385
England_NHSE	Asthma	age_strat	01/05/2020	20-29	1150
England_NHSE	Asthma	age_strat	01/05/2020	30-39	1130
England_NHSE	Asthma	age_strat	01/05/2020	40-49	1200
England_NHSE	Asthma	age_strat	01/05/2020	50-59	1140
England_NHSE	Asthma	age_strat	01/05/2020	60-69	790
England_NHSE	Asthma	age_strat	01/05/2020	70+	805
England_NHSE	Asthma	age_strat	01/06/2020	0-4	650
England_NHSE	Asthma	age_strat	01/06/2020	5-9	1145
England_NHSE	Asthma	age_strat	01/06/2020	10-14	760
England_NHSE	Asthma	age_strat	01/06/2020	15-19	525
England_NHSE	Asthma	age_strat	01/06/2020	20-29	1465
England_NHSE	Asthma	age_strat	01/06/2020	30-39	1485
England_NHSE	Asthma	age_strat	01/06/2020	40-49	1340
England_NHSE	Asthma	age_strat	01/06/2020	50-59	1345
England_NHSE	Asthma	age_strat	01/06/2020	60-69	980
England_NHSE	Asthma	age_strat	01/06/2020	70+	880
England_NHSE	Asthma	age_strat	01/07/2020	0-4	565
England_NHSE	Asthma	age_strat	01/07/2020	5-9	970
England_NHSE	Asthma	age_strat	01/07/2020	10-14	630
England_NHSE	Asthma	age_strat	01/07/2020	15-19	420
England_NHSE	Asthma	age_strat	01/07/2020	20-29	1250
England_NHSE	Asthma	age_strat	01/07/2020	30-39	1310
England_NHSE	Asthma	age_strat	01/07/2020	40-49	1255
England_NHSE	Asthma	age_strat	01/07/2020	50-59	1230
England_NHSE	Asthma	age_strat	01/07/2020	60-69	905
England_NHSE	Asthma	age_strat	01/07/2020	70+	910
England_NHSE	Asthma	age_strat	01/08/2020	0-4	550
England_NHSE	Asthma	age_strat	01/08/2020	5-9	925
England_NHSE	Asthma	age_strat	01/08/2020	10-14	585
England_NHSE	Asthma	age_strat	01/08/2020	15-19	450

England_NHSE	Asthma	age_strat	01/08/2020	20-29	1160
England_NHSE	Asthma	age_strat	01/08/2020	30-39	1110
England_NHSE	Asthma	age_strat	01/08/2020	40-49	1030
England_NHSE	Asthma	age_strat	01/08/2020	50-59	1075
England_NHSE	Asthma	age_strat	01/08/2020	60-69	845
England_NHSE	Asthma	age_strat	01/08/2020	70+	845
England_NHSE	Asthma	age_strat	01/09/2020	0-4	1500
England_NHSE	Asthma	age_strat	01/09/2020	5-9	2615
England_NHSE	Asthma	age_strat	01/09/2020	10-14	1330
England_NHSE	Asthma	age_strat	01/09/2020	15-19	765
England_NHSE	Asthma	age_strat	01/09/2020	20-29	1635
England_NHSE	Asthma	age_strat	01/09/2020	30-39	1495
England_NHSE	Asthma	age_strat	01/09/2020	40-49	1325
England_NHSE	Asthma	age_strat	01/09/2020	50-59	1360
England_NHSE	Asthma	age_strat	01/09/2020	60-69	1085
England_NHSE	Asthma	age_strat	01/09/2020	70+	930
England_NHSE	Asthma	age_strat	01/10/2020	0-4	1395
England_NHSE	Asthma	age_strat	01/10/2020	5-9	2275
England_NHSE	Asthma	age_strat	01/10/2020	10-14	1335
England_NHSE	Asthma	age_strat	01/10/2020	15-19	800
England_NHSE	Asthma	age_strat	01/10/2020	20-29	1780
England_NHSE	Asthma	age_strat	01/10/2020	30-39	1745
England_NHSE	Asthma	age_strat	01/10/2020	40-49	1550
England_NHSE	Asthma	age_strat	01/10/2020	50-59	1575
England_NHSE	Asthma	age_strat	01/10/2020	60-69	1080
England_NHSE	Asthma	age_strat	01/10/2020	70+	1030
England_NHSE	Asthma	age_strat	01/11/2020	0-4	1385
England_NHSE	Asthma	age_strat	01/11/2020	5-9	2140
England_NHSE	Asthma	age_strat	01/11/2020	10-14	1110
England_NHSE	Asthma	age_strat	01/11/2020	15-19	725
England_NHSE	Asthma	age_strat	01/11/2020	20-29	1720
England_NHSE	Asthma	age_strat	01/11/2020	30-39	1715
England_NHSE	Asthma	age_strat	01/11/2020	40-49	1655
England_NHSE	Asthma	age_strat	01/11/2020	50-59	1580
England_NHSE	Asthma	age_strat	01/11/2020	60-69	1105
England_NHSE	Asthma	age_strat	01/11/2020	70+	1115
England_NHSE	Asthma	age_strat	01/12/2020	0-4	1195
England_NHSE	Asthma	age_strat	01/12/2020	5-9	1740
England_NHSE	Asthma	age_strat	01/12/2020	10-14	985
England_NHSE	Asthma	age_strat	01/12/2020	15-19	620
England_NHSE	Asthma	age_strat	01/12/2020	20-29	1470
England_NHSE	Asthma	age_strat	01/12/2020	30-39	1580
England_NHSE	Asthma	age_strat	01/12/2020	40-49	1395
England_NHSE	Asthma	age_strat	01/12/2020	50-59	1425
England_NHSE	Asthma	age_strat	01/12/2020	60-69	1045
England_NHSE	Asthma	age_strat	01/12/2020	70+	980
England_NHSE	Asthma	age_strat	01/01/2021	0-4	960
England_NHSE	Asthma	age_strat	01/01/2021	5-9	1270
England_NHSE	Asthma	age_strat	01/01/2021	10-14	755
England_NHSE	Asthma	age_strat	01/01/2021	15-19	560
England_NHSE	Asthma	age_strat	01/01/2021	20-29	1845
England_NHSE	Asthma	age_strat	01/01/2021	30-39	1820
England_NHSE	Asthma	age_strat	01/01/2021	40-49	1585
England_NHSE	Asthma	age_strat	01/01/2021	50-59	1640
England_NHSE	Asthma	age_strat	01/01/2021	60-69	1195
England_NHSE	Asthma	age_strat	01/01/2021	70+	1070
England_NHSE	Asthma	age_strat	01/02/2021	0-4	650
England_NHSE	Asthma	age_strat	01/02/2021	5-9	1005
England_NHSE	Asthma	age_strat	01/02/2021	10-14	590
England_NHSE	Asthma	age_strat	01/02/2021	15-19	530
England_NHSE	Asthma	age_strat	01/02/2021	20-29	1745
England_NHSE	Asthma	age_strat	01/02/2021	30-39	1690
England_NHSE	Asthma	age_strat	01/02/2021	40-49	1620

England_NHSE	Asthma	age_strat	01/02/2021	50-59	1565
England_NHSE	Asthma	age_strat	01/02/2021	60-69	1115
England_NHSE	Asthma	age_strat	01/02/2021	70+	960
England_NHSE	Asthma	age_strat	01/03/2021	0-4	1120
England_NHSE	Asthma	age_strat	01/03/2021	5-9	1615
England_NHSE	Asthma	age_strat	01/03/2021	10-14	980
England_NHSE	Asthma	age_strat	01/03/2021	15-19	720
England_NHSE	Asthma	age_strat	01/03/2021	20-29	2395
England_NHSE	Asthma	age_strat	01/03/2021	30-39	2195
England_NHSE	Asthma	age_strat	01/03/2021	40-49	1875
England_NHSE	Asthma	age_strat	01/03/2021	50-59	1920
England_NHSE	Asthma	age_strat	01/03/2021	60-69	1290
England_NHSE	Asthma	age_strat	01/03/2021	70+	1275
England_NHSE	Asthma	age_strat	01/04/2021	0-4	1035
England_NHSE	Asthma	age_strat	01/04/2021	5-9	1320
England_NHSE	Asthma	age_strat	01/04/2021	10-14	890
England_NHSE	Asthma	age_strat	01/04/2021	15-19	670
England_NHSE	Asthma	age_strat	01/04/2021	20-29	1980
England_NHSE	Asthma	age_strat	01/04/2021	30-39	1870
England_NHSE	Asthma	age_strat	01/04/2021	40-49	1580
England_NHSE	Asthma	age_strat	01/04/2021	50-59	1390
England_NHSE	Asthma	age_strat	01/04/2021	60-69	1055
England_NHSE	Asthma	age_strat	01/04/2021	70+	940
England_NHSE	Asthma	age_strat	01/05/2021	0-4	1465
England_NHSE	Asthma	age_strat	01/05/2021	5-9	1640
England_NHSE	Asthma	age_strat	01/05/2021	10-14	975
England_NHSE	Asthma	age_strat	01/05/2021	15-19	565
England_NHSE	Asthma	age_strat	01/05/2021	20-29	1805
England_NHSE	Asthma	age_strat	01/05/2021	30-39	1555
England_NHSE	Asthma	age_strat	01/05/2021	40-49	1335
England_NHSE	Asthma	age_strat	01/05/2021	50-59	1305
England_NHSE	Asthma	age_strat	01/05/2021	60-69	970
England_NHSE	Asthma	age_strat	01/05/2021	70+	1180
England_NHSE	Asthma	age_strat	01/06/2021	0-4	1705
England_NHSE	Asthma	age_strat	01/06/2021	5-9	1880
England_NHSE	Asthma	age_strat	01/06/2021	10-14	1300
England_NHSE	Asthma	age_strat	01/06/2021	15-19	745
England_NHSE	Asthma	age_strat	01/06/2021	20-29	2005
England_NHSE	Asthma	age_strat	01/06/2021	30-39	1840
England_NHSE	Asthma	age_strat	01/06/2021	40-49	1505
England_NHSE	Asthma	age_strat	01/06/2021	50-59	1465
England_NHSE	Asthma	age_strat	01/06/2021	60-69	1045
England_NHSE	Asthma	age_strat	01/06/2021	70+	1165
England_NHSE	Asthma	age_strat	01/07/2021	0-4	1620
England_NHSE	Asthma	age_strat	01/07/2021	5-9	1695
England_NHSE	Asthma	age_strat	01/07/2021	10-14	1105
England_NHSE	Asthma	age_strat	01/07/2021	15-19	620
England_NHSE	Asthma	age_strat	01/07/2021	20-29	1580
England_NHSE	Asthma	age_strat	01/07/2021	30-39	1510
England_NHSE	Asthma	age_strat	01/07/2021	40-49	1285
England_NHSE	Asthma	age_strat	01/07/2021	50-59	1335
England_NHSE	Asthma	age_strat	01/07/2021	60-69	1025
England_NHSE	Asthma	age_strat	01/07/2021	70+	1045
England_NHSE	Asthma	age_strat	01/08/2021	0-4	1175
England_NHSE	Asthma	age_strat	01/08/2021	5-9	1235
England_NHSE	Asthma	age_strat	01/08/2021	10-14	790
England_NHSE	Asthma	age_strat	01/08/2021	15-19	515
England_NHSE	Asthma	age_strat	01/08/2021	20-29	1355
England_NHSE	Asthma	age_strat	01/08/2021	30-39	1210
England_NHSE	Asthma	age_strat	01/08/2021	40-49	1105
England_NHSE	Asthma	age_strat	01/08/2021	50-59	1230
England_NHSE	Asthma	age_strat	01/08/2021	60-69	935
England_NHSE	Asthma	age_strat	01/08/2021	70+	1045

England_NHSE	Asthma	age_strat	01/09/2021	0-4	2005
England_NHSE	Asthma	age_strat	01/09/2021	5-9	2480
England_NHSE	Asthma	age_strat	01/09/2021	10-14	1245
England_NHSE	Asthma	age_strat	01/09/2021	15-19	850
England_NHSE	Asthma	age_strat	01/09/2021	20-29	1650
England_NHSE	Asthma	age_strat	01/09/2021	30-39	1475
England_NHSE	Asthma	age_strat	01/09/2021	40-49	1280
England_NHSE	Asthma	age_strat	01/09/2021	50-59	1350
England_NHSE	Asthma	age_strat	01/09/2021	60-69	1095
England_NHSE	Asthma	age_strat	01/09/2021	70+	1095
England_NHSE	Asthma	age_strat	01/10/2021	0-4	2105
England_NHSE	Asthma	age_strat	01/10/2021	5-9	2830
England_NHSE	Asthma	age_strat	01/10/2021	10-14	1365
England_NHSE	Asthma	age_strat	01/10/2021	15-19	865
England_NHSE	Asthma	age_strat	01/10/2021	20-29	1740
England_NHSE	Asthma	age_strat	01/10/2021	30-39	1580
England_NHSE	Asthma	age_strat	01/10/2021	40-49	1410
England_NHSE	Asthma	age_strat	01/10/2021	50-59	1385
England_NHSE	Asthma	age_strat	01/10/2021	60-69	1025
England_NHSE	Asthma	age_strat	01/10/2021	70+	1125
England_NHSE	Asthma	age_strat	01/11/2021	0-4	2385
England_NHSE	Asthma	age_strat	01/11/2021	5-9	3255
England_NHSE	Asthma	age_strat	01/11/2021	10-14	1500
England_NHSE	Asthma	age_strat	01/11/2021	15-19	865
England_NHSE	Asthma	age_strat	01/11/2021	20-29	1935
England_NHSE	Asthma	age_strat	01/11/2021	30-39	1840
England_NHSE	Asthma	age_strat	01/11/2021	40-49	1560
England_NHSE	Asthma	age_strat	01/11/2021	50-59	1520
England_NHSE	Asthma	age_strat	01/11/2021	60-69	1235
England_NHSE	Asthma	age_strat	01/11/2021	70+	1180
England_NHSE	Asthma	age_strat	01/12/2021	0-4	1815
England_NHSE	Asthma	age_strat	01/12/2021	5-9	2740
England_NHSE	Asthma	age_strat	01/12/2021	10-14	1360
England_NHSE	Asthma	age_strat	01/12/2021	15-19	720
England_NHSE	Asthma	age_strat	01/12/2021	20-29	1585
England_NHSE	Asthma	age_strat	01/12/2021	30-39	1605
England_NHSE	Asthma	age_strat	01/12/2021	40-49	1360
England_NHSE	Asthma	age_strat	01/12/2021	50-59	1360
England_NHSE	Asthma	age_strat	01/12/2021	60-69	1040
England_NHSE	Asthma	age_strat	01/12/2021	70+	1035
England_NHSE	Asthma	age_strat	01/01/2022	0-4	1675
England_NHSE	Asthma	age_strat	01/01/2022	5-9	2530
England_NHSE	Asthma	age_strat	01/01/2022	10-14	1390
England_NHSE	Asthma	age_strat	01/01/2022	15-19	670
England_NHSE	Asthma	age_strat	01/01/2022	20-29	1655
England_NHSE	Asthma	age_strat	01/01/2022	30-39	1585
England_NHSE	Asthma	age_strat	01/01/2022	40-49	1435
England_NHSE	Asthma	age_strat	01/01/2022	50-59	1475
England_NHSE	Asthma	age_strat	01/01/2022	60-69	1165
England_NHSE	Asthma	age_strat	01/01/2022	70+	1130
England_NHSE	Asthma	age_strat	01/02/2022	0-4	1605
England_NHSE	Asthma	age_strat	01/02/2022	5-9	2305
England_NHSE	Asthma	age_strat	01/02/2022	10-14	1275
England_NHSE	Asthma	age_strat	01/02/2022	15-19	695
England_NHSE	Asthma	age_strat	01/02/2022	20-29	1480
England_NHSE	Asthma	age_strat	01/02/2022	30-39	1390
England_NHSE	Asthma	age_strat	01/02/2022	40-49	1345
England_NHSE	Asthma	age_strat	01/02/2022	50-59	1250
England_NHSE	Asthma	age_strat	01/02/2022	60-69	1120
England_NHSE	Asthma	age_strat	01/02/2022	70+	1090
England_NHSE	Asthma	age_strat	01/03/2022	0-4	1895
England_NHSE	Asthma	age_strat	01/03/2022	5-9	2600
England_NHSE	Asthma	age_strat	01/03/2022	10-14	1415

England_NHSE	Asthma	age_strat	01/03/2022	15-19	800
England_NHSE	Asthma	age_strat	01/03/2022	20-29	1725
England_NHSE	Asthma	age_strat	01/03/2022	30-39	1605
England_NHSE	Asthma	age_strat	01/03/2022	40-49	1475
England_NHSE	Asthma	age_strat	01/03/2022	50-59	1530
England_NHSE	Asthma	age_strat	01/03/2022	60-69	1280
England_NHSE	Asthma	age_strat	01/03/2022	70+	1185
England_NHSE	Asthma	age_strat	01/04/2022	0-4	1355
England_NHSE	Asthma	age_strat	01/04/2022	5-9	1970
England_NHSE	Asthma	age_strat	01/04/2022	10-14	1150
England_NHSE	Asthma	age_strat	01/04/2022	15-19	610
England_NHSE	Asthma	age_strat	01/04/2022	20-29	1325
England_NHSE	Asthma	age_strat	01/04/2022	30-39	1330
England_NHSE	Asthma	age_strat	01/04/2022	40-49	1165
England_NHSE	Asthma	age_strat	01/04/2022	50-59	1360
England_NHSE	Asthma	age_strat	01/04/2022	60-69	1150
England_NHSE	Asthma	age_strat	01/04/2022	70+	1040
England_NHSE	Asthma	age_strat	01/05/2022	0-4	1745
England_NHSE	Asthma	age_strat	01/05/2022	5-9	2660
England_NHSE	Asthma	age_strat	01/05/2022	10-14	1475
England_NHSE	Asthma	age_strat	01/05/2022	15-19	650
England_NHSE	Asthma	age_strat	01/05/2022	20-29	1535
England_NHSE	Asthma	age_strat	01/05/2022	30-39	1560
England_NHSE	Asthma	age_strat	01/05/2022	40-49	1385
England_NHSE	Asthma	age_strat	01/05/2022	50-59	1450
England_NHSE	Asthma	age_strat	01/05/2022	60-69	1310
England_NHSE	Asthma	age_strat	01/05/2022	70+	1215
England_NHSE	Asthma	age_strat	01/06/2022	0-4	1400
England_NHSE	Asthma	age_strat	01/06/2022	5-9	2505
England_NHSE	Asthma	age_strat	01/06/2022	10-14	1500
England_NHSE	Asthma	age_strat	01/06/2022	15-19	685
England_NHSE	Asthma	age_strat	01/06/2022	20-29	1790
England_NHSE	Asthma	age_strat	01/06/2022	30-39	1710
England_NHSE	Asthma	age_strat	01/06/2022	40-49	1505
England_NHSE	Asthma	age_strat	01/06/2022	50-59	1455
England_NHSE	Asthma	age_strat	01/06/2022	60-69	1275
England_NHSE	Asthma	age_strat	01/06/2022	70+	1170
England_NHSE	Asthma	age_strat	01/07/2022	0-4	1235
England_NHSE	Asthma	age_strat	01/07/2022	5-9	2245
England_NHSE	Asthma	age_strat	01/07/2022	10-14	1290
England_NHSE	Asthma	age_strat	01/07/2022	15-19	660
England_NHSE	Asthma	age_strat	01/07/2022	20-29	1415
England_NHSE	Asthma	age_strat	01/07/2022	30-39	1355
England_NHSE	Asthma	age_strat	01/07/2022	40-49	1220
England_NHSE	Asthma	age_strat	01/07/2022	50-59	1350
England_NHSE	Asthma	age_strat	01/07/2022	60-69	1180
England_NHSE	Asthma	age_strat	01/07/2022	70+	1175
England_NHSE	Asthma	age_strat	01/08/2022	0-4	1045
England_NHSE	Asthma	age_strat	01/08/2022	5-9	1715
England_NHSE	Asthma	age_strat	01/08/2022	10-14	975
England_NHSE	Asthma	age_strat	01/08/2022	15-19	625
England_NHSE	Asthma	age_strat	01/08/2022	20-29	1415
England_NHSE	Asthma	age_strat	01/08/2022	30-39	1295
England_NHSE	Asthma	age_strat	01/08/2022	40-49	1210
England_NHSE	Asthma	age_strat	01/08/2022	50-59	1375
England_NHSE	Asthma	age_strat	01/08/2022	60-69	1215
England_NHSE	Asthma	age_strat	01/08/2022	70+	1250
England_NHSE	Asthma	age_strat	01/09/2022	0-4	1345
England_NHSE	Asthma	age_strat	01/09/2022	5-9	2545
England_NHSE	Asthma	age_strat	01/09/2022	10-14	1280
England_NHSE	Asthma	age_strat	01/09/2022	15-19	735
England_NHSE	Asthma	age_strat	01/09/2022	20-29	1525
England_NHSE	Asthma	age_strat	01/09/2022	30-39	1410

England_NHSE	Asthma	age_strat	01/09/2022	40-49	1190
England_NHSE	Asthma	age_strat	01/09/2022	50-59	1300
England_NHSE	Asthma	age_strat	01/09/2022	60-69	1140
England_NHSE	Asthma	age_strat	01/09/2022	70+	1170
England_NHSE	Asthma	age_strat	01/10/2022	0-4	1440
England_NHSE	Asthma	age_strat	01/10/2022	5-9	2890
England_NHSE	Asthma	age_strat	01/10/2022	10-14	1495
England_NHSE	Asthma	age_strat	01/10/2022	15-19	850
England_NHSE	Asthma	age_strat	01/10/2022	20-29	1795
England_NHSE	Asthma	age_strat	01/10/2022	30-39	1655
England_NHSE	Asthma	age_strat	01/10/2022	40-49	1415
England_NHSE	Asthma	age_strat	01/10/2022	50-59	1495
England_NHSE	Asthma	age_strat	01/10/2022	60-69	1255
England_NHSE	Asthma	age_strat	01/10/2022	70+	1235
England_NHSE	Asthma	age_strat	01/11/2022	0-4	1560
England_NHSE	Asthma	age_strat	01/11/2022	5-9	3320
England_NHSE	Asthma	age_strat	01/11/2022	10-14	1715
England_NHSE	Asthma	age_strat	01/11/2022	15-19	835
England_NHSE	Asthma	age_strat	01/11/2022	20-29	1930
England_NHSE	Asthma	age_strat	01/11/2022	30-39	1925
England_NHSE	Asthma	age_strat	01/11/2022	40-49	1660
England_NHSE	Asthma	age_strat	01/11/2022	50-59	1740
England_NHSE	Asthma	age_strat	01/11/2022	60-69	1540
England_NHSE	Asthma	age_strat	01/11/2022	70+	1460
England_NHSE	Asthma	age_strat	01/12/2022	0-4	1195
England_NHSE	Asthma	age_strat	01/12/2022	5-9	2840
England_NHSE	Asthma	age_strat	01/12/2022	10-14	1505
England_NHSE	Asthma	age_strat	01/12/2022	15-19	765
England_NHSE	Asthma	age_strat	01/12/2022	20-29	1630
England_NHSE	Asthma	age_strat	01/12/2022	30-39	1800
England_NHSE	Asthma	age_strat	01/12/2022	40-49	1615
England_NHSE	Asthma	age_strat	01/12/2022	50-59	1600
England_NHSE	Asthma	age_strat	01/12/2022	60-69	1415
England_NHSE	Asthma	age_strat	01/12/2022	70+	1280
England_NHSE	Asthma	age_strat	01/01/2023	0-4	1260
England_NHSE	Asthma	age_strat	01/01/2023	5-9	3205
England_NHSE	Asthma	age_strat	01/01/2023	10-14	1590
England_NHSE	Asthma	age_strat	01/01/2023	15-19	890
England_NHSE	Asthma	age_strat	01/01/2023	20-29	1940
England_NHSE	Asthma	age_strat	01/01/2023	30-39	2020
England_NHSE	Asthma	age_strat	01/01/2023	40-49	1795
England_NHSE	Asthma	age_strat	01/01/2023	50-59	1880
England_NHSE	Asthma	age_strat	01/01/2023	60-69	1590
England_NHSE	Asthma	age_strat	01/01/2023	70+	1530
England_NHSE	Asthma	age_strat	01/02/2023	0-4	1150
England_NHSE	Asthma	age_strat	01/02/2023	5-9	2990
England_NHSE	Asthma	age_strat	01/02/2023	10-14	1545
England_NHSE	Asthma	age_strat	01/02/2023	15-19	805
England_NHSE	Asthma	age_strat	01/02/2023	20-29	1735
England_NHSE	Asthma	age_strat	01/02/2023	30-39	1805
England_NHSE	Asthma	age_strat	01/02/2023	40-49	1665
England_NHSE	Asthma	age_strat	01/02/2023	50-59	1720
England_NHSE	Asthma	age_strat	01/02/2023	60-69	1535
England_NHSE	Asthma	age_strat	01/02/2023	70+	1355
England_NHSE	Asthma	age_strat	01/03/2023	0-4	1230
England_NHSE	Asthma	age_strat	01/03/2023	5-9	3200
England_NHSE	Asthma	age_strat	01/03/2023	10-14	1670
England_NHSE	Asthma	age_strat	01/03/2023	15-19	815
England_NHSE	Asthma	age_strat	01/03/2023	20-29	1830
England_NHSE	Asthma	age_strat	01/03/2023	30-39	1995
England_NHSE	Asthma	age_strat	01/03/2023	40-49	1765
England_NHSE	Asthma	age_strat	01/03/2023	50-59	1900
England_NHSE	Asthma	age_strat	01/03/2023	60-69	1660

England_NHSE	Asthma	age_strat	01/03/2023	70+		1605
England_NHSE	Asthma	age_strat	01/04/2023	0-4		820
England_NHSE	Asthma	age_strat	01/04/2023	5-9		2425
England_NHSE	Asthma	age_strat	01/04/2023	10-14		1310
England_NHSE	Asthma	age_strat	01/04/2023	15-19		625
England_NHSE	Asthma	age_strat	01/04/2023	20-29		1390
England_NHSE	Asthma	age_strat	01/04/2023	30-39		1565
England_NHSE	Asthma	age_strat	01/04/2023	40-49		1315
England_NHSE	Asthma	age_strat	01/04/2023	50-59		1440
England_NHSE	Asthma	age_strat	01/04/2023	60-69		1265
England_NHSE	Asthma	age_strat	01/04/2023	70+		1190
England_NHSE	Asthma	age_strat	01/05/2023	0-4		935
England_NHSE	Asthma	age_strat	01/05/2023	5-9		2785
England_NHSE	Asthma	age_strat	01/05/2023	10-14		1440
England_NHSE	Asthma	age_strat	01/05/2023	15-19		655
England_NHSE	Asthma	age_strat	01/05/2023	20-29		1545
England_NHSE	Asthma	age_strat	01/05/2023	30-39		1690
England_NHSE	Asthma	age_strat	01/05/2023	40-49		1475
England_NHSE	Asthma	age_strat	01/05/2023	50-59		1600
England_NHSE	Asthma	age_strat	01/05/2023	60-69		1465
England_NHSE	Asthma	age_strat	01/05/2023	70+		1300
England_NHSE	Asthma	age_strat	01/06/2023	0-4		835
England_NHSE	Asthma	age_strat	01/06/2023	5-9		2935
England_NHSE	Asthma	age_strat	01/06/2023	10-14		2035
England_NHSE	Asthma	age_strat	01/06/2023	15-19		945
England_NHSE	Asthma	age_strat	01/06/2023	20-29		2420
England_NHSE	Asthma	age_strat	01/06/2023	30-39		2435
England_NHSE	Asthma	age_strat	01/06/2023	40-49		2075
England_NHSE	Asthma	age_strat	01/06/2023	50-59		1770
England_NHSE	Asthma	age_strat	01/06/2023	60-69		1665
England_NHSE	Asthma	age_strat	01/06/2023	70+		1425
England_NHSE	Asthma	region_strat	01/11/2019		East Midlands	1570
England_NHSE	Asthma	region_strat	01/11/2019		East of England	2330
England_NHSE	Asthma	region_strat	01/11/2019		London	3740
England_NHSE	Asthma	region_strat	01/11/2019		North East	960
England_NHSE	Asthma	region_strat	01/11/2019		North West	3225
England_NHSE	Asthma	region_strat	01/11/2019		South East	3390
England_NHSE	Asthma	region_strat	01/11/2019		South West	2180
England_NHSE	Asthma	region_strat	01/11/2019		West Midlands	2240
England_NHSE	Asthma	region_strat	01/11/2019		Yorkshire and The Humber	2080
England_NHSE	Asthma	region_strat	01/12/2019		East Midlands	1540
England_NHSE	Asthma	region_strat	01/12/2019		East of England	2270
England_NHSE	Asthma	region_strat	01/12/2019		London	3485
England_NHSE	Asthma	region_strat	01/12/2019		North East	845
England_NHSE	Asthma	region_strat	01/12/2019		North West	2970
England_NHSE	Asthma	region_strat	01/12/2019		South East	3245
England_NHSE	Asthma	region_strat	01/12/2019		South West	2130
England_NHSE	Asthma	region_strat	01/12/2019		West Midlands	2135
England_NHSE	Asthma	region_strat	01/12/2019		Yorkshire and The Humber	1960
England_NHSE	Asthma	region_strat	01/01/2020		East Midlands	1760
England_NHSE	Asthma	region_strat	01/01/2020		East of England	2540
England_NHSE	Asthma	region_strat	01/01/2020		London	4070
England_NHSE	Asthma	region_strat	01/01/2020		North East	1025
England_NHSE	Asthma	region_strat	01/01/2020		North West	3325
England_NHSE	Asthma	region_strat	01/01/2020		South East	3750
England_NHSE	Asthma	region_strat	01/01/2020		South West	2425
England_NHSE	Asthma	region_strat	01/01/2020		West Midlands	2455
England_NHSE	Asthma	region_strat	01/01/2020		Yorkshire and The Humber	2295
England_NHSE	Asthma	region_strat	01/02/2020		East Midlands	1505
England_NHSE	Asthma	region_strat	01/02/2020		East of England	2150
England_NHSE	Asthma	region_strat	01/02/2020		London	3435
England_NHSE	Asthma	region_strat	01/02/2020		North East	840
England_NHSE	Asthma	region_strat	01/02/2020		North West	2645

England_NHSE	Asthma	region_strat	01/02/2020	South East	3255
England_NHSE	Asthma	region_strat	01/02/2020	South West	1985
England_NHSE	Asthma	region_strat	01/02/2020	West Midlands	1880
England_NHSE	Asthma	region_strat	01/02/2020	Yorkshire and The Humber	1790
England_NHSE	Asthma	region_strat	01/03/2020	East Midlands	1610
England_NHSE	Asthma	region_strat	01/03/2020	East of England	2505
England_NHSE	Asthma	region_strat	01/03/2020	London	4165
England_NHSE	Asthma	region_strat	01/03/2020	North East	1050
England_NHSE	Asthma	region_strat	01/03/2020	North West	2945
England_NHSE	Asthma	region_strat	01/03/2020	South East	3775
England_NHSE	Asthma	region_strat	01/03/2020	South West	2325
England_NHSE	Asthma	region_strat	01/03/2020	West Midlands	2030
England_NHSE	Asthma	region_strat	01/03/2020	Yorkshire and The Humber	2145
England_NHSE	Asthma	region_strat	01/04/2020	East Midlands	825
England_NHSE	Asthma	region_strat	01/04/2020	East of England	1215
England_NHSE	Asthma	region_strat	01/04/2020	London	2020
England_NHSE	Asthma	region_strat	01/04/2020	North East	460
England_NHSE	Asthma	region_strat	01/04/2020	North West	1360
England_NHSE	Asthma	region_strat	01/04/2020	South East	1705
England_NHSE	Asthma	region_strat	01/04/2020	South West	1095
England_NHSE	Asthma	region_strat	01/04/2020	West Midlands	955
England_NHSE	Asthma	region_strat	01/04/2020	Yorkshire and The Humber	1180
England_NHSE	Asthma	region_strat	01/05/2020	East Midlands	655
England_NHSE	Asthma	region_strat	01/05/2020	East of England	1040
England_NHSE	Asthma	region_strat	01/05/2020	London	1570
England_NHSE	Asthma	region_strat	01/05/2020	North East	380
England_NHSE	Asthma	region_strat	01/05/2020	North West	1295
England_NHSE	Asthma	region_strat	01/05/2020	South East	1290
England_NHSE	Asthma	region_strat	01/05/2020	South West	910
England_NHSE	Asthma	region_strat	01/05/2020	West Midlands	745
England_NHSE	Asthma	region_strat	01/05/2020	Yorkshire and The Humber	850
England_NHSE	Asthma	region_strat	01/06/2020	East Midlands	905
England_NHSE	Asthma	region_strat	01/06/2020	East of England	1215
England_NHSE	Asthma	region_strat	01/06/2020	London	1895
England_NHSE	Asthma	region_strat	01/06/2020	North East	485
England_NHSE	Asthma	region_strat	01/06/2020	North West	1365
England_NHSE	Asthma	region_strat	01/06/2020	South East	1605
England_NHSE	Asthma	region_strat	01/06/2020	South West	1090
England_NHSE	Asthma	region_strat	01/06/2020	West Midlands	1010
England_NHSE	Asthma	region_strat	01/06/2020	Yorkshire and The Humber	1010
England_NHSE	Asthma	region_strat	01/07/2020	East Midlands	810
England_NHSE	Asthma	region_strat	01/07/2020	East of England	1055
England_NHSE	Asthma	region_strat	01/07/2020	London	1620
England_NHSE	Asthma	region_strat	01/07/2020	North East	460
England_NHSE	Asthma	region_strat	01/07/2020	North West	1265
England_NHSE	Asthma	region_strat	01/07/2020	South East	1455
England_NHSE	Asthma	region_strat	01/07/2020	South West	915
England_NHSE	Asthma	region_strat	01/07/2020	West Midlands	890
England_NHSE	Asthma	region_strat	01/07/2020	Yorkshire and The Humber	975
England_NHSE	Asthma	region_strat	01/08/2020	East Midlands	710
England_NHSE	Asthma	region_strat	01/08/2020	East of England	1015
England_NHSE	Asthma	region_strat	01/08/2020	London	1410
England_NHSE	Asthma	region_strat	01/08/2020	North East	370
England_NHSE	Asthma	region_strat	01/08/2020	North West	1135
England_NHSE	Asthma	region_strat	01/08/2020	South East	1340
England_NHSE	Asthma	region_strat	01/08/2020	South West	890
England_NHSE	Asthma	region_strat	01/08/2020	West Midlands	825
England_NHSE	Asthma	region_strat	01/08/2020	Yorkshire and The Humber	880
England_NHSE	Asthma	region_strat	01/09/2020	East Midlands	1045
England_NHSE	Asthma	region_strat	01/09/2020	East of England	1580
England_NHSE	Asthma	region_strat	01/09/2020	London	2385
England_NHSE	Asthma	region_strat	01/09/2020	North East	665
England_NHSE	Asthma	region_strat	01/09/2020	North West	1980

England_NHSE	Asthma	region_strat	01/09/2020	South East	2110
England_NHSE	Asthma	region_strat	01/09/2020	South West	1340
England_NHSE	Asthma	region_strat	01/09/2020	West Midlands	1455
England_NHSE	Asthma	region_strat	01/09/2020	Yorkshire and The Humber	1480
England_NHSE	Asthma	region_strat	01/10/2020	East Midlands	1085
England_NHSE	Asthma	region_strat	01/10/2020	East of England	1625
England_NHSE	Asthma	region_strat	01/10/2020	London	2665
England_NHSE	Asthma	region_strat	01/10/2020	North East	670
England_NHSE	Asthma	region_strat	01/10/2020	North West	1900
England_NHSE	Asthma	region_strat	01/10/2020	South East	2290
England_NHSE	Asthma	region_strat	01/10/2020	South West	1515
England_NHSE	Asthma	region_strat	01/10/2020	West Midlands	1440
England_NHSE	Asthma	region_strat	01/10/2020	Yorkshire and The Humber	1375
England_NHSE	Asthma	region_strat	01/11/2020	East Midlands	1095
England_NHSE	Asthma	region_strat	01/11/2020	East of England	1575
England_NHSE	Asthma	region_strat	01/11/2020	London	2415
England_NHSE	Asthma	region_strat	01/11/2020	North East	720
England_NHSE	Asthma	region_strat	01/11/2020	North West	1855
England_NHSE	Asthma	region_strat	01/11/2020	South East	2330
England_NHSE	Asthma	region_strat	01/11/2020	South West	1475
England_NHSE	Asthma	region_strat	01/11/2020	West Midlands	1415
England_NHSE	Asthma	region_strat	01/11/2020	Yorkshire and The Humber	1380
England_NHSE	Asthma	region_strat	01/12/2020	East Midlands	975
England_NHSE	Asthma	region_strat	01/12/2020	East of England	1400
England_NHSE	Asthma	region_strat	01/12/2020	London	2140
England_NHSE	Asthma	region_strat	01/12/2020	North East	625
England_NHSE	Asthma	region_strat	01/12/2020	North West	1650
England_NHSE	Asthma	region_strat	01/12/2020	South East	1915
England_NHSE	Asthma	region_strat	01/12/2020	South West	1310
England_NHSE	Asthma	region_strat	01/12/2020	West Midlands	1230
England_NHSE	Asthma	region_strat	01/12/2020	Yorkshire and The Humber	1190
England_NHSE	Asthma	region_strat	01/01/2021	East Midlands	1050
England_NHSE	Asthma	region_strat	01/01/2021	East of England	1550
England_NHSE	Asthma	region_strat	01/01/2021	London	2290
England_NHSE	Asthma	region_strat	01/01/2021	North East	680
England_NHSE	Asthma	region_strat	01/01/2021	North West	1535
England_NHSE	Asthma	region_strat	01/01/2021	South East	1940
England_NHSE	Asthma	region_strat	01/01/2021	South West	1395
England_NHSE	Asthma	region_strat	01/01/2021	West Midlands	1055
England_NHSE	Asthma	region_strat	01/01/2021	Yorkshire and The Humber	1210
England_NHSE	Asthma	region_strat	01/02/2021	East Midlands	875
England_NHSE	Asthma	region_strat	01/02/2021	East of England	1305
England_NHSE	Asthma	region_strat	01/02/2021	London	2150
England_NHSE	Asthma	region_strat	01/02/2021	North East	500
England_NHSE	Asthma	region_strat	01/02/2021	North West	1485
England_NHSE	Asthma	region_strat	01/02/2021	South East	1740
England_NHSE	Asthma	region_strat	01/02/2021	South West	1210
England_NHSE	Asthma	region_strat	01/02/2021	West Midlands	1025
England_NHSE	Asthma	region_strat	01/02/2021	Yorkshire and The Humber	1175
England_NHSE	Asthma	region_strat	01/03/2021	East Midlands	1160
England_NHSE	Asthma	region_strat	01/03/2021	East of England	1660
England_NHSE	Asthma	region_strat	01/03/2021	London	3105
England_NHSE	Asthma	region_strat	01/03/2021	North East	690
England_NHSE	Asthma	region_strat	01/03/2021	North West	2060
England_NHSE	Asthma	region_strat	01/03/2021	South East	2470
England_NHSE	Asthma	region_strat	01/03/2021	South West	1370
England_NHSE	Asthma	region_strat	01/03/2021	West Midlands	1380
England_NHSE	Asthma	region_strat	01/03/2021	Yorkshire and The Humber	1490
England_NHSE	Asthma	region_strat	01/04/2021	East Midlands	940
England_NHSE	Asthma	region_strat	01/04/2021	East of England	1420
England_NHSE	Asthma	region_strat	01/04/2021	London	2630
England_NHSE	Asthma	region_strat	01/04/2021	North East	560
England_NHSE	Asthma	region_strat	01/04/2021	North West	1750

England_NHSE	Asthma	region_strat	01/04/2021	South East	1930
England_NHSE	Asthma	region_strat	01/04/2021	South West	1160
England_NHSE	Asthma	region_strat	01/04/2021	West Midlands	1180
England_NHSE	Asthma	region_strat	01/04/2021	Yorkshire and The Humber	1165
England_NHSE	Asthma	region_strat	01/05/2021	East Midlands	1085
England_NHSE	Asthma	region_strat	01/05/2021	East of England	1370
England_NHSE	Asthma	region_strat	01/05/2021	London	2355
England_NHSE	Asthma	region_strat	01/05/2021	North East	570
England_NHSE	Asthma	region_strat	01/05/2021	North West	1595
England_NHSE	Asthma	region_strat	01/05/2021	South East	2300
England_NHSE	Asthma	region_strat	01/05/2021	South West	1120
England_NHSE	Asthma	region_strat	01/05/2021	West Midlands	1205
England_NHSE	Asthma	region_strat	01/05/2021	Yorkshire and The Humber	1190
England_NHSE	Asthma	region_strat	01/06/2021	East Midlands	1195
England_NHSE	Asthma	region_strat	01/06/2021	East of England	1795
England_NHSE	Asthma	region_strat	01/06/2021	London	2895
England_NHSE	Asthma	region_strat	01/06/2021	North East	685
England_NHSE	Asthma	region_strat	01/06/2021	North West	1685
England_NHSE	Asthma	region_strat	01/06/2021	South East	2240
England_NHSE	Asthma	region_strat	01/06/2021	South West	1390
England_NHSE	Asthma	region_strat	01/06/2021	West Midlands	1355
England_NHSE	Asthma	region_strat	01/06/2021	Yorkshire and The Humber	1405
England_NHSE	Asthma	region_strat	01/07/2021	East Midlands	1090
England_NHSE	Asthma	region_strat	01/07/2021	East of England	1565
England_NHSE	Asthma	region_strat	01/07/2021	London	2155
England_NHSE	Asthma	region_strat	01/07/2021	North East	615
England_NHSE	Asthma	region_strat	01/07/2021	North West	1575
England_NHSE	Asthma	region_strat	01/07/2021	South East	1960
England_NHSE	Asthma	region_strat	01/07/2021	South West	1275
England_NHSE	Asthma	region_strat	01/07/2021	West Midlands	1215
England_NHSE	Asthma	region_strat	01/07/2021	Yorkshire and The Humber	1365
England_NHSE	Asthma	region_strat	01/08/2021	East Midlands	915
England_NHSE	Asthma	region_strat	01/08/2021	East of England	1280
England_NHSE	Asthma	region_strat	01/08/2021	London	1670
England_NHSE	Asthma	region_strat	01/08/2021	North East	545
England_NHSE	Asthma	region_strat	01/08/2021	North West	1330
England_NHSE	Asthma	region_strat	01/08/2021	South East	1765
England_NHSE	Asthma	region_strat	01/08/2021	South West	985
England_NHSE	Asthma	region_strat	01/08/2021	West Midlands	1010
England_NHSE	Asthma	region_strat	01/08/2021	Yorkshire and The Humber	1105
England_NHSE	Asthma	region_strat	01/09/2021	East Midlands	1255
England_NHSE	Asthma	region_strat	01/09/2021	East of England	1685
England_NHSE	Asthma	region_strat	01/09/2021	London	2580
England_NHSE	Asthma	region_strat	01/09/2021	North East	670
England_NHSE	Asthma	region_strat	01/09/2021	North West	1935
England_NHSE	Asthma	region_strat	01/09/2021	South East	2215
England_NHSE	Asthma	region_strat	01/09/2021	South West	1280
England_NHSE	Asthma	region_strat	01/09/2021	West Midlands	1385
England_NHSE	Asthma	region_strat	01/09/2021	Yorkshire and The Humber	1525
England_NHSE	Asthma	region_strat	01/10/2021	East Midlands	1170
England_NHSE	Asthma	region_strat	01/10/2021	East of England	1890
England_NHSE	Asthma	region_strat	01/10/2021	London	2680
England_NHSE	Asthma	region_strat	01/10/2021	North East	740
England_NHSE	Asthma	region_strat	01/10/2021	North West	2045
England_NHSE	Asthma	region_strat	01/10/2021	South East	2370
England_NHSE	Asthma	region_strat	01/10/2021	South West	1415
England_NHSE	Asthma	region_strat	01/10/2021	West Midlands	1540
England_NHSE	Asthma	region_strat	01/10/2021	Yorkshire and The Humber	1575
England_NHSE	Asthma	region_strat	01/11/2021	East Midlands	1430
England_NHSE	Asthma	region_strat	01/11/2021	East of England	2055
England_NHSE	Asthma	region_strat	01/11/2021	London	2825
England_NHSE	Asthma	region_strat	01/11/2021	North East	815
England_NHSE	Asthma	region_strat	01/11/2021	North West	2275

England_NHSE	Asthma	region_strat	01/11/2021	South East	2625
England_NHSE	Asthma	region_strat	01/11/2021	South West	1610
England_NHSE	Asthma	region_strat	01/11/2021	West Midlands	1785
England_NHSE	Asthma	region_strat	01/11/2021	Yorkshire and The Humber	1855
England_NHSE	Asthma	region_strat	01/12/2021	East Midlands	1210
England_NHSE	Asthma	region_strat	01/12/2021	East of England	1690
England_NHSE	Asthma	region_strat	01/12/2021	London	2435
England_NHSE	Asthma	region_strat	01/12/2021	North East	745
England_NHSE	Asthma	region_strat	01/12/2021	North West	1870
England_NHSE	Asthma	region_strat	01/12/2021	South East	2220
England_NHSE	Asthma	region_strat	01/12/2021	South West	1425
England_NHSE	Asthma	region_strat	01/12/2021	West Midlands	1385
England_NHSE	Asthma	region_strat	01/12/2021	Yorkshire and The Humber	1650
England_NHSE	Asthma	region_strat	01/01/2022	East Midlands	1295
England_NHSE	Asthma	region_strat	01/01/2022	East of England	1745
England_NHSE	Asthma	region_strat	01/01/2022	London	2415
England_NHSE	Asthma	region_strat	01/01/2022	North East	665
England_NHSE	Asthma	region_strat	01/01/2022	North West	1820
England_NHSE	Asthma	region_strat	01/01/2022	South East	2255
England_NHSE	Asthma	region_strat	01/01/2022	South West	1510
England_NHSE	Asthma	region_strat	01/01/2022	West Midlands	1405
England_NHSE	Asthma	region_strat	01/01/2022	Yorkshire and The Humber	1610
England_NHSE	Asthma	region_strat	01/02/2022	East Midlands	1135
England_NHSE	Asthma	region_strat	01/02/2022	East of England	1645
England_NHSE	Asthma	region_strat	01/02/2022	London	2200
England_NHSE	Asthma	region_strat	01/02/2022	North East	625
England_NHSE	Asthma	region_strat	01/02/2022	North West	1840
England_NHSE	Asthma	region_strat	01/02/2022	South East	1980
England_NHSE	Asthma	region_strat	01/02/2022	South West	1360
England_NHSE	Asthma	region_strat	01/02/2022	West Midlands	1400
England_NHSE	Asthma	region_strat	01/02/2022	Yorkshire and The Humber	1375
England_NHSE	Asthma	region_strat	01/03/2022	East Midlands	1255
England_NHSE	Asthma	region_strat	01/03/2022	East of England	1905
England_NHSE	Asthma	region_strat	01/03/2022	London	2545
England_NHSE	Asthma	region_strat	01/03/2022	North East	760
England_NHSE	Asthma	region_strat	01/03/2022	North West	1965
England_NHSE	Asthma	region_strat	01/03/2022	South East	2320
England_NHSE	Asthma	region_strat	01/03/2022	South West	1425
England_NHSE	Asthma	region_strat	01/03/2022	West Midlands	1580
England_NHSE	Asthma	region_strat	01/03/2022	Yorkshire and The Humber	1745
England_NHSE	Asthma	region_strat	01/04/2022	East Midlands	1040
England_NHSE	Asthma	region_strat	01/04/2022	East of England	1490
England_NHSE	Asthma	region_strat	01/04/2022	London	2015
England_NHSE	Asthma	region_strat	01/04/2022	North East	560
England_NHSE	Asthma	region_strat	01/04/2022	North West	1590
England_NHSE	Asthma	region_strat	01/04/2022	South East	1935
England_NHSE	Asthma	region_strat	01/04/2022	South West	1225
England_NHSE	Asthma	region_strat	01/04/2022	West Midlands	1280
England_NHSE	Asthma	region_strat	01/04/2022	Yorkshire and The Humber	1320
England_NHSE	Asthma	region_strat	01/05/2022	East Midlands	1275
England_NHSE	Asthma	region_strat	01/05/2022	East of England	1750
England_NHSE	Asthma	region_strat	01/05/2022	London	2485
England_NHSE	Asthma	region_strat	01/05/2022	North East	690
England_NHSE	Asthma	region_strat	01/05/2022	North West	1895
England_NHSE	Asthma	region_strat	01/05/2022	South East	2280
England_NHSE	Asthma	region_strat	01/05/2022	South West	1435
England_NHSE	Asthma	region_strat	01/05/2022	West Midlands	1550
England_NHSE	Asthma	region_strat	01/05/2022	Yorkshire and The Humber	1625
England_NHSE	Asthma	region_strat	01/06/2022	East Midlands	1295
England_NHSE	Asthma	region_strat	01/06/2022	East of England	1770
England_NHSE	Asthma	region_strat	01/06/2022	London	2485
England_NHSE	Asthma	region_strat	01/06/2022	North East	725
England_NHSE	Asthma	region_strat	01/06/2022	North West	1825

England_NHSE	Asthma	region_strat	01/06/2022	South East	2195
England_NHSE	Asthma	region_strat	01/06/2022	South West	1445
England_NHSE	Asthma	region_strat	01/06/2022	West Midlands	1600
England_NHSE	Asthma	region_strat	01/06/2022	Yorkshire and The Humber	1655
England_NHSE	Asthma	region_strat	01/07/2022	East Midlands	1135
England_NHSE	Asthma	region_strat	01/07/2022	East of England	1560
England_NHSE	Asthma	region_strat	01/07/2022	London	2135
England_NHSE	Asthma	region_strat	01/07/2022	North East	630
England_NHSE	Asthma	region_strat	01/07/2022	North West	1685
England_NHSE	Asthma	region_strat	01/07/2022	South East	1900
England_NHSE	Asthma	region_strat	01/07/2022	South West	1325
England_NHSE	Asthma	region_strat	01/07/2022	West Midlands	1320
England_NHSE	Asthma	region_strat	01/07/2022	Yorkshire and The Humber	1440
England_NHSE	Asthma	region_strat	01/08/2022	East Midlands	1055
England_NHSE	Asthma	region_strat	01/08/2022	East of England	1420
England_NHSE	Asthma	region_strat	01/08/2022	London	1885
England_NHSE	Asthma	region_strat	01/08/2022	North East	610
England_NHSE	Asthma	region_strat	01/08/2022	North West	1560
England_NHSE	Asthma	region_strat	01/08/2022	South East	1685
England_NHSE	Asthma	region_strat	01/08/2022	South West	1270
England_NHSE	Asthma	region_strat	01/08/2022	West Midlands	1260
England_NHSE	Asthma	region_strat	01/08/2022	Yorkshire and The Humber	1375
England_NHSE	Asthma	region_strat	01/09/2022	East Midlands	1110
England_NHSE	Asthma	region_strat	01/09/2022	East of England	1570
England_NHSE	Asthma	region_strat	01/09/2022	London	2260
England_NHSE	Asthma	region_strat	01/09/2022	North East	615
England_NHSE	Asthma	region_strat	01/09/2022	North West	1780
England_NHSE	Asthma	region_strat	01/09/2022	South East	2050
England_NHSE	Asthma	region_strat	01/09/2022	South West	1400
England_NHSE	Asthma	region_strat	01/09/2022	West Midlands	1375
England_NHSE	Asthma	region_strat	01/09/2022	Yorkshire and The Humber	1475
England_NHSE	Asthma	region_strat	01/10/2022	East Midlands	1215
England_NHSE	Asthma	region_strat	01/10/2022	East of England	1805
England_NHSE	Asthma	region_strat	01/10/2022	London	2600
England_NHSE	Asthma	region_strat	01/10/2022	North East	710
England_NHSE	Asthma	region_strat	01/10/2022	North West	2065
England_NHSE	Asthma	region_strat	01/10/2022	South East	2410
England_NHSE	Asthma	region_strat	01/10/2022	South West	1475
England_NHSE	Asthma	region_strat	01/10/2022	West Midlands	1585
England_NHSE	Asthma	region_strat	01/10/2022	Yorkshire and The Humber	1660
England_NHSE	Asthma	region_strat	01/11/2022	East Midlands	1440
England_NHSE	Asthma	region_strat	01/11/2022	East of England	1950
England_NHSE	Asthma	region_strat	01/11/2022	London	2865
England_NHSE	Asthma	region_strat	01/11/2022	North East	900
England_NHSE	Asthma	region_strat	01/11/2022	North West	2295
England_NHSE	Asthma	region_strat	01/11/2022	South East	2795
England_NHSE	Asthma	region_strat	01/11/2022	South West	1825
England_NHSE	Asthma	region_strat	01/11/2022	West Midlands	1725
England_NHSE	Asthma	region_strat	01/11/2022	Yorkshire and The Humber	1890
England_NHSE	Asthma	region_strat	01/12/2022	East Midlands	1330
England_NHSE	Asthma	region_strat	01/12/2022	East of England	1780
England_NHSE	Asthma	region_strat	01/12/2022	London	2500
England_NHSE	Asthma	region_strat	01/12/2022	North East	755
England_NHSE	Asthma	region_strat	01/12/2022	North West	2005
England_NHSE	Asthma	region_strat	01/12/2022	South East	2400
England_NHSE	Asthma	region_strat	01/12/2022	South West	1575
England_NHSE	Asthma	region_strat	01/12/2022	West Midlands	1580
England_NHSE	Asthma	region_strat	01/12/2022	Yorkshire and The Humber	1715
England_NHSE	Asthma	region_strat	01/01/2023	East Midlands	1520
England_NHSE	Asthma	region_strat	01/01/2023	East of England	2025
England_NHSE	Asthma	region_strat	01/01/2023	London	2865
England_NHSE	Asthma	region_strat	01/01/2023	North East	875
England_NHSE	Asthma	region_strat	01/01/2023	North West	2255

England_NHSE	Asthma	region_strat	01/01/2023	South East	2745
England_NHSE	Asthma	region_strat	01/01/2023	South West	1830
England_NHSE	Asthma	region_strat	01/01/2023	West Midlands	1670
England_NHSE	Asthma	region_strat	01/01/2023	Yorkshire and The Humber	1920
England_NHSE	Asthma	region_strat	01/02/2023	East Midlands	1420
England_NHSE	Asthma	region_strat	01/02/2023	East of England	1850
England_NHSE	Asthma	region_strat	01/02/2023	London	2585
England_NHSE	Asthma	region_strat	01/02/2023	North East	800
England_NHSE	Asthma	region_strat	01/02/2023	North West	2035
England_NHSE	Asthma	region_strat	01/02/2023	South East	2510
England_NHSE	Asthma	region_strat	01/02/2023	South West	1755
England_NHSE	Asthma	region_strat	01/02/2023	West Midlands	1615
England_NHSE	Asthma	region_strat	01/02/2023	Yorkshire and The Humber	1740
England_NHSE	Asthma	region_strat	01/03/2023	East Midlands	1455
England_NHSE	Asthma	region_strat	01/03/2023	East of England	2165
England_NHSE	Asthma	region_strat	01/03/2023	London	2740
England_NHSE	Asthma	region_strat	01/03/2023	North East	890
England_NHSE	Asthma	region_strat	01/03/2023	North West	2180
England_NHSE	Asthma	region_strat	01/03/2023	South East	2835
England_NHSE	Asthma	region_strat	01/03/2023	South West	1775
England_NHSE	Asthma	region_strat	01/03/2023	West Midlands	1740
England_NHSE	Asthma	region_strat	01/03/2023	Yorkshire and The Humber	1900
England_NHSE	Asthma	region_strat	01/04/2023	East Midlands	1085
England_NHSE	Asthma	region_strat	01/04/2023	East of England	1485
England_NHSE	Asthma	region_strat	01/04/2023	London	2230
England_NHSE	Asthma	region_strat	01/04/2023	North East	660
England_NHSE	Asthma	region_strat	01/04/2023	North West	1680
England_NHSE	Asthma	region_strat	01/04/2023	South East	2115
England_NHSE	Asthma	region_strat	01/04/2023	South West	1385
England_NHSE	Asthma	region_strat	01/04/2023	West Midlands	1295
England_NHSE	Asthma	region_strat	01/04/2023	Yorkshire and The Humber	1400
England_NHSE	Asthma	region_strat	01/05/2023	East Midlands	1205
England_NHSE	Asthma	region_strat	01/05/2023	East of England	1660
England_NHSE	Asthma	region_strat	01/05/2023	London	2440
England_NHSE	Asthma	region_strat	01/05/2023	North East	860
England_NHSE	Asthma	region_strat	01/05/2023	North West	1845
England_NHSE	Asthma	region_strat	01/05/2023	South East	2425
England_NHSE	Asthma	region_strat	01/05/2023	South West	1520
England_NHSE	Asthma	region_strat	01/05/2023	West Midlands	1445
England_NHSE	Asthma	region_strat	01/05/2023	Yorkshire and The Humber	1495
England_NHSE	Asthma	region_strat	01/06/2023	East Midlands	1725
England_NHSE	Asthma	region_strat	01/06/2023	East of England	2080
England_NHSE	Asthma	region_strat	01/06/2023	London	3255
England_NHSE	Asthma	region_strat	01/06/2023	North East	820
England_NHSE	Asthma	region_strat	01/06/2023	North West	2115
England_NHSE	Asthma	region_strat	01/06/2023	South East	2915
England_NHSE	Asthma	region_strat	01/06/2023	South West	1730
England_NHSE	Asthma	region_strat	01/06/2023	West Midlands	2070
England_NHSE	Asthma	region_strat	01/06/2023	Yorkshire and The Humber	1830
England_NHSE	COPD	all	01/11/2019		9970
England_NHSE	COPD	all	01/12/2019		10415
England_NHSE	COPD	all	01/01/2020		12305
England_NHSE	COPD	all	01/02/2020		10270
England_NHSE	COPD	all	01/03/2020		8845
England_NHSE	COPD	all	01/04/2020		3275
England_NHSE	COPD	all	01/05/2020		2945
England_NHSE	COPD	all	01/06/2020		3470
England_NHSE	COPD	all	01/07/2020		3685
England_NHSE	COPD	all	01/08/2020		3690
England_NHSE	COPD	all	01/09/2020		4475
England_NHSE	COPD	all	01/10/2020		4655
England_NHSE	COPD	all	01/11/2020		4625
England_NHSE	COPD	all	01/12/2020		4400

England_NHSE	COPD	all	01/01/2021		4600
England_NHSE	COPD	all	01/02/2021		4415
England_NHSE	COPD	all	01/03/2021		5480
England_NHSE	COPD	all	01/04/2021		4880
England_NHSE	COPD	all	01/05/2021		5255
England_NHSE	COPD	all	01/06/2021		5885
England_NHSE	COPD	all	01/07/2021		5925
England_NHSE	COPD	all	01/08/2021		5520
England_NHSE	COPD	all	01/09/2021		6380
England_NHSE	COPD	all	01/10/2021		6770
England_NHSE	COPD	all	01/11/2021		7595
England_NHSE	COPD	all	01/12/2021		7105
England_NHSE	COPD	all	01/01/2022		6940
England_NHSE	COPD	all	01/02/2022		6615
England_NHSE	COPD	all	01/03/2022		8065
England_NHSE	COPD	all	01/04/2022		7075
England_NHSE	COPD	all	01/05/2022		7895
England_NHSE	COPD	all	01/06/2022		7520
England_NHSE	COPD	all	01/07/2022		7555
England_NHSE	COPD	all	01/08/2022		7570
England_NHSE	COPD	all	01/09/2022		7650
England_NHSE	COPD	all	01/10/2022		8375
England_NHSE	COPD	all	01/11/2022		9640
England_NHSE	COPD	all	01/12/2022		9515
England_NHSE	COPD	all	01/01/2023		10250
England_NHSE	COPD	all	01/02/2023		9300
England_NHSE	COPD	all	01/03/2023		10685
England_NHSE	COPD	all	01/04/2023		8420
England_NHSE	COPD	all	01/05/2023		9160
England_NHSE	COPD	all	01/06/2023		9285
England_NHSE	COPD	sex_strat	01/11/2019	Female	5100
England_NHSE	COPD	sex_strat	01/11/2019	Male	4870
England_NHSE	COPD	sex_strat	01/12/2019	Female	5295
England_NHSE	COPD	sex_strat	01/12/2019	Male	5120
England_NHSE	COPD	sex_strat	01/01/2020	Female	6380
England_NHSE	COPD	sex_strat	01/01/2020	Male	5920
England_NHSE	COPD	sex_strat	01/02/2020	Female	5380
England_NHSE	COPD	sex_strat	01/02/2020	Male	4895
England_NHSE	COPD	sex_strat	01/03/2020	Female	4665
England_NHSE	COPD	sex_strat	01/03/2020	Male	4180
England_NHSE	COPD	sex_strat	01/04/2020	Female	1680
England_NHSE	COPD	sex_strat	01/04/2020	Male	1595
England_NHSE	COPD	sex_strat	01/05/2020	Female	1515
England_NHSE	COPD	sex_strat	01/05/2020	Male	1435
England_NHSE	COPD	sex_strat	01/06/2020	Female	1770
England_NHSE	COPD	sex_strat	01/06/2020	Male	1700
England_NHSE	COPD	sex_strat	01/07/2020	Female	1945
England_NHSE	COPD	sex_strat	01/07/2020	Male	1740
England_NHSE	COPD	sex_strat	01/08/2020	Female	1915
England_NHSE	COPD	sex_strat	01/08/2020	Male	1775
England_NHSE	COPD	sex_strat	01/09/2020	Female	2290
England_NHSE	COPD	sex_strat	01/09/2020	Male	2180
England_NHSE	COPD	sex_strat	01/10/2020	Female	2370
England_NHSE	COPD	sex_strat	01/10/2020	Male	2285
England_NHSE	COPD	sex_strat	01/11/2020	Female	2375
England_NHSE	COPD	sex_strat	01/11/2020	Male	2250
England_NHSE	COPD	sex_strat	01/12/2020	Female	2355
England_NHSE	COPD	sex_strat	01/12/2020	Male	2045
England_NHSE	COPD	sex_strat	01/01/2021	Female	2370
England_NHSE	COPD	sex_strat	01/01/2021	Male	2230
England_NHSE	COPD	sex_strat	01/02/2021	Female	2355
England_NHSE	COPD	sex_strat	01/02/2021	Male	2055
England_NHSE	COPD	sex_strat	01/03/2021	Female	2810

England_NHSE	COPD	sex_strat	01/03/2021	Male		2670
England_NHSE	COPD	sex_strat	01/04/2021	Female		2510
England_NHSE	COPD	sex_strat	01/04/2021	Male		2370
England_NHSE	COPD	sex_strat	01/05/2021	Female		2680
England_NHSE	COPD	sex_strat	01/05/2021	Male		2575
England_NHSE	COPD	sex_strat	01/06/2021	Female		2990
England_NHSE	COPD	sex_strat	01/06/2021	Male		2895
England_NHSE	COPD	sex_strat	01/07/2021	Female		3120
England_NHSE	COPD	sex_strat	01/07/2021	Male		2805
England_NHSE	COPD	sex_strat	01/08/2021	Female		2865
England_NHSE	COPD	sex_strat	01/08/2021	Male		2655
England_NHSE	COPD	sex_strat	01/09/2021	Female		3270
England_NHSE	COPD	sex_strat	01/09/2021	Male		3115
England_NHSE	COPD	sex_strat	01/10/2021	Female		3445
England_NHSE	COPD	sex_strat	01/10/2021	Male		3320
England_NHSE	COPD	sex_strat	01/11/2021	Female		3830
England_NHSE	COPD	sex_strat	01/11/2021	Male		3760
England_NHSE	COPD	sex_strat	01/12/2021	Female		3575
England_NHSE	COPD	sex_strat	01/12/2021	Male		3530
England_NHSE	COPD	sex_strat	01/01/2022	Female		3515
England_NHSE	COPD	sex_strat	01/01/2022	Male		3425
England_NHSE	COPD	sex_strat	01/02/2022	Female		3405
England_NHSE	COPD	sex_strat	01/02/2022	Male		3205
England_NHSE	COPD	sex_strat	01/03/2022	Female		4235
England_NHSE	COPD	sex_strat	01/03/2022	Male		3830
England_NHSE	COPD	sex_strat	01/04/2022	Female		3700
England_NHSE	COPD	sex_strat	01/04/2022	Male		3380
England_NHSE	COPD	sex_strat	01/05/2022	Female		4050
England_NHSE	COPD	sex_strat	01/05/2022	Male		3845
England_NHSE	COPD	sex_strat	01/06/2022	Female		3900
England_NHSE	COPD	sex_strat	01/06/2022	Male		3615
England_NHSE	COPD	sex_strat	01/07/2022	Female		3980
England_NHSE	COPD	sex_strat	01/07/2022	Male		3575
England_NHSE	COPD	sex_strat	01/08/2022	Female		3950
England_NHSE	COPD	sex_strat	01/08/2022	Male		3620
England_NHSE	COPD	sex_strat	01/09/2022	Female		3995
England_NHSE	COPD	sex_strat	01/09/2022	Male		3655
England_NHSE	COPD	sex_strat	01/10/2022	Female		4260
England_NHSE	COPD	sex_strat	01/10/2022	Male		4115
England_NHSE	COPD	sex_strat	01/11/2022	Female		4970
England_NHSE	COPD	sex_strat	01/11/2022	Male		4670
England_NHSE	COPD	sex_strat	01/12/2022	Female		4780
England_NHSE	COPD	sex_strat	01/12/2022	Male		4735
England_NHSE	COPD	sex_strat	01/01/2023	Female		5270
England_NHSE	COPD	sex_strat	01/01/2023	Male		4985
England_NHSE	COPD	sex_strat	01/02/2023	Female		4850
England_NHSE	COPD	sex_strat	01/02/2023	Male		4445
England_NHSE	COPD	sex_strat	01/03/2023	Female		5585
England_NHSE	COPD	sex_strat	01/03/2023	Male		5100
England_NHSE	COPD	sex_strat	01/04/2023	Female		4380
England_NHSE	COPD	sex_strat	01/04/2023	Male		4045
England_NHSE	COPD	sex_strat	01/05/2023	Female		4800
England_NHSE	COPD	sex_strat	01/05/2023	Male		4365
England_NHSE	COPD	sex_strat	01/06/2023	Female		4875
England_NHSE	COPD	sex_strat	01/06/2023	Male		4410
England_NHSE	COPD	age_strat	01/11/2019		40-49	730
England_NHSE	COPD	age_strat	01/11/2019		50-59	2010
England_NHSE	COPD	age_strat	01/11/2019		60-69	2830
England_NHSE	COPD	age_strat	01/11/2019		70+	4400
England_NHSE	COPD	age_strat	01/12/2019		40-49	810
England_NHSE	COPD	age_strat	01/12/2019		50-59	2025
England_NHSE	COPD	age_strat	01/12/2019		60-69	2810
England_NHSE	COPD	age_strat	01/12/2019		70+	4765

England_NHSE	COPD	age_strat	01/01/2020	40-49	895
England_NHSE	COPD	age_strat	01/01/2020	50-59	2535
England_NHSE	COPD	age_strat	01/01/2020	60-69	3415
England_NHSE	COPD	age_strat	01/01/2020	70+	5455
England_NHSE	COPD	age_strat	01/02/2020	40-49	770
England_NHSE	COPD	age_strat	01/02/2020	50-59	2105
England_NHSE	COPD	age_strat	01/02/2020	60-69	2855
England_NHSE	COPD	age_strat	01/02/2020	70+	4540
England_NHSE	COPD	age_strat	01/03/2020	40-49	655
England_NHSE	COPD	age_strat	01/03/2020	50-59	1950
England_NHSE	COPD	age_strat	01/03/2020	60-69	2540
England_NHSE	COPD	age_strat	01/03/2020	70+	3700
England_NHSE	COPD	age_strat	01/04/2020	40-49	265
England_NHSE	COPD	age_strat	01/04/2020	50-59	680
England_NHSE	COPD	age_strat	01/04/2020	60-69	875
England_NHSE	COPD	age_strat	01/04/2020	70+	1450
England_NHSE	COPD	age_strat	01/05/2020	40-49	255
England_NHSE	COPD	age_strat	01/05/2020	50-59	570
England_NHSE	COPD	age_strat	01/05/2020	60-69	745
England_NHSE	COPD	age_strat	01/05/2020	70+	1380
England_NHSE	COPD	age_strat	01/06/2020	40-49	285
England_NHSE	COPD	age_strat	01/06/2020	50-59	705
England_NHSE	COPD	age_strat	01/06/2020	60-69	860
England_NHSE	COPD	age_strat	01/06/2020	70+	1615
England_NHSE	COPD	age_strat	01/07/2020	40-49	265
England_NHSE	COPD	age_strat	01/07/2020	50-59	790
England_NHSE	COPD	age_strat	01/07/2020	60-69	890
England_NHSE	COPD	age_strat	01/07/2020	70+	1740
England_NHSE	COPD	age_strat	01/08/2020	40-49	295
England_NHSE	COPD	age_strat	01/08/2020	50-59	730
England_NHSE	COPD	age_strat	01/08/2020	60-69	920
England_NHSE	COPD	age_strat	01/08/2020	70+	1745
England_NHSE	COPD	age_strat	01/09/2020	40-49	375
England_NHSE	COPD	age_strat	01/09/2020	50-59	910
England_NHSE	COPD	age_strat	01/09/2020	60-69	1160
England_NHSE	COPD	age_strat	01/09/2020	70+	2025
England_NHSE	COPD	age_strat	01/10/2020	40-49	370
England_NHSE	COPD	age_strat	01/10/2020	50-59	1025
England_NHSE	COPD	age_strat	01/10/2020	60-69	1185
England_NHSE	COPD	age_strat	01/10/2020	70+	2070
England_NHSE	COPD	age_strat	01/11/2020	40-49	410
England_NHSE	COPD	age_strat	01/11/2020	50-59	1020
England_NHSE	COPD	age_strat	01/11/2020	60-69	1200
England_NHSE	COPD	age_strat	01/11/2020	70+	2000
England_NHSE	COPD	age_strat	01/12/2020	40-49	365
England_NHSE	COPD	age_strat	01/12/2020	50-59	905
England_NHSE	COPD	age_strat	01/12/2020	60-69	1120
England_NHSE	COPD	age_strat	01/12/2020	70+	2010
England_NHSE	COPD	age_strat	01/01/2021	40-49	390
England_NHSE	COPD	age_strat	01/01/2021	50-59	1010
England_NHSE	COPD	age_strat	01/01/2021	60-69	1240
England_NHSE	COPD	age_strat	01/01/2021	70+	1960
England_NHSE	COPD	age_strat	01/02/2021	40-49	385
England_NHSE	COPD	age_strat	01/02/2021	50-59	955
England_NHSE	COPD	age_strat	01/02/2021	60-69	1175
England_NHSE	COPD	age_strat	01/02/2021	70+	1895
England_NHSE	COPD	age_strat	01/03/2021	40-49	505
England_NHSE	COPD	age_strat	01/03/2021	50-59	1180
England_NHSE	COPD	age_strat	01/03/2021	60-69	1405
England_NHSE	COPD	age_strat	01/03/2021	70+	2385
England_NHSE	COPD	age_strat	01/04/2021	40-49	405
England_NHSE	COPD	age_strat	01/04/2021	50-59	1065
England_NHSE	COPD	age_strat	01/04/2021	60-69	1265

England_NHSE	COPD	age_strat	01/04/2021	70+	2145
England_NHSE	COPD	age_strat	01/05/2021	40-49	450
England_NHSE	COPD	age_strat	01/05/2021	50-59	1070
England_NHSE	COPD	age_strat	01/05/2021	60-69	1380
England_NHSE	COPD	age_strat	01/05/2021	70+	2355
England_NHSE	COPD	age_strat	01/06/2021	40-49	460
England_NHSE	COPD	age_strat	01/06/2021	50-59	1205
England_NHSE	COPD	age_strat	01/06/2021	60-69	1635
England_NHSE	COPD	age_strat	01/06/2021	70+	2590
England_NHSE	COPD	age_strat	01/07/2021	40-49	440
England_NHSE	COPD	age_strat	01/07/2021	50-59	1180
England_NHSE	COPD	age_strat	01/07/2021	60-69	1630
England_NHSE	COPD	age_strat	01/07/2021	70+	2675
England_NHSE	COPD	age_strat	01/08/2021	40-49	425
England_NHSE	COPD	age_strat	01/08/2021	50-59	1110
England_NHSE	COPD	age_strat	01/08/2021	60-69	1495
England_NHSE	COPD	age_strat	01/08/2021	70+	2495
England_NHSE	COPD	age_strat	01/09/2021	40-49	485
England_NHSE	COPD	age_strat	01/09/2021	50-59	1370
England_NHSE	COPD	age_strat	01/09/2021	60-69	1780
England_NHSE	COPD	age_strat	01/09/2021	70+	2740
England_NHSE	COPD	age_strat	01/10/2021	40-49	505
England_NHSE	COPD	age_strat	01/10/2021	50-59	1455
England_NHSE	COPD	age_strat	01/10/2021	60-69	1895
England_NHSE	COPD	age_strat	01/10/2021	70+	2915
England_NHSE	COPD	age_strat	01/11/2021	40-49	565
England_NHSE	COPD	age_strat	01/11/2021	50-59	1590
England_NHSE	COPD	age_strat	01/11/2021	60-69	2210
England_NHSE	COPD	age_strat	01/11/2021	70+	3225
England_NHSE	COPD	age_strat	01/12/2021	40-49	525
England_NHSE	COPD	age_strat	01/12/2021	50-59	1445
England_NHSE	COPD	age_strat	01/12/2021	60-69	2030
England_NHSE	COPD	age_strat	01/12/2021	70+	3100
England_NHSE	COPD	age_strat	01/01/2022	40-49	445
England_NHSE	COPD	age_strat	01/01/2022	50-59	1400
England_NHSE	COPD	age_strat	01/01/2022	60-69	2015
England_NHSE	COPD	age_strat	01/01/2022	70+	3080
England_NHSE	COPD	age_strat	01/02/2022	40-49	490
England_NHSE	COPD	age_strat	01/02/2022	50-59	1335
England_NHSE	COPD	age_strat	01/02/2022	60-69	1980
England_NHSE	COPD	age_strat	01/02/2022	70+	2810
England_NHSE	COPD	age_strat	01/03/2022	40-49	525
England_NHSE	COPD	age_strat	01/03/2022	50-59	1635
England_NHSE	COPD	age_strat	01/03/2022	60-69	2425
England_NHSE	COPD	age_strat	01/03/2022	70+	3475
England_NHSE	COPD	age_strat	01/04/2022	40-49	455
England_NHSE	COPD	age_strat	01/04/2022	50-59	1465
England_NHSE	COPD	age_strat	01/04/2022	60-69	2125
England_NHSE	COPD	age_strat	01/04/2022	70+	3035
England_NHSE	COPD	age_strat	01/05/2022	40-49	515
England_NHSE	COPD	age_strat	01/05/2022	50-59	1620
England_NHSE	COPD	age_strat	01/05/2022	60-69	2395
England_NHSE	COPD	age_strat	01/05/2022	70+	3365
England_NHSE	COPD	age_strat	01/06/2022	40-49	520
England_NHSE	COPD	age_strat	01/06/2022	50-59	1505
England_NHSE	COPD	age_strat	01/06/2022	60-69	2265
England_NHSE	COPD	age_strat	01/06/2022	70+	3225
England_NHSE	COPD	age_strat	01/07/2022	40-49	480
England_NHSE	COPD	age_strat	01/07/2022	50-59	1625
England_NHSE	COPD	age_strat	01/07/2022	60-69	2355
England_NHSE	COPD	age_strat	01/07/2022	70+	3100
England_NHSE	COPD	age_strat	01/08/2022	40-49	475
England_NHSE	COPD	age_strat	01/08/2022	50-59	1520

England_NHSE	COPD	age_strat	01/08/2022	60-69		2305
England_NHSE	COPD	age_strat	01/08/2022	70+		3265
England_NHSE	COPD	age_strat	01/09/2022	40-49		545
England_NHSE	COPD	age_strat	01/09/2022	50-59		1560
England_NHSE	COPD	age_strat	01/09/2022	60-69		2335
England_NHSE	COPD	age_strat	01/09/2022	70+		3205
England_NHSE	COPD	age_strat	01/10/2022	40-49		595
England_NHSE	COPD	age_strat	01/10/2022	50-59		1705
England_NHSE	COPD	age_strat	01/10/2022	60-69		2630
England_NHSE	COPD	age_strat	01/10/2022	70+		3440
England_NHSE	COPD	age_strat	01/11/2022	40-49		680
England_NHSE	COPD	age_strat	01/11/2022	50-59		2065
England_NHSE	COPD	age_strat	01/11/2022	60-69		2930
England_NHSE	COPD	age_strat	01/11/2022	70+		3965
England_NHSE	COPD	age_strat	01/12/2022	40-49		650
England_NHSE	COPD	age_strat	01/12/2022	50-59		1865
England_NHSE	COPD	age_strat	01/12/2022	60-69		2890
England_NHSE	COPD	age_strat	01/12/2022	70+		4110
England_NHSE	COPD	age_strat	01/01/2023	40-49		670
England_NHSE	COPD	age_strat	01/01/2023	50-59		2060
England_NHSE	COPD	age_strat	01/01/2023	60-69		3170
England_NHSE	COPD	age_strat	01/01/2023	70+		4355
England_NHSE	COPD	age_strat	01/02/2023	40-49		625
England_NHSE	COPD	age_strat	01/02/2023	50-59		1885
England_NHSE	COPD	age_strat	01/02/2023	60-69		3005
England_NHSE	COPD	age_strat	01/02/2023	70+		3785
England_NHSE	COPD	age_strat	01/03/2023	40-49		700
England_NHSE	COPD	age_strat	01/03/2023	50-59		2205
England_NHSE	COPD	age_strat	01/03/2023	60-69		3320
England_NHSE	COPD	age_strat	01/03/2023	70+		4465
England_NHSE	COPD	age_strat	01/04/2023	40-49		505
England_NHSE	COPD	age_strat	01/04/2023	50-59		1780
England_NHSE	COPD	age_strat	01/04/2023	60-69		2570
England_NHSE	COPD	age_strat	01/04/2023	70+		3560
England_NHSE	COPD	age_strat	01/05/2023	40-49		560
England_NHSE	COPD	age_strat	01/05/2023	50-59		1880
England_NHSE	COPD	age_strat	01/05/2023	60-69		2840
England_NHSE	COPD	age_strat	01/05/2023	70+		3885
England_NHSE	COPD	age_strat	01/06/2023	40-49		605
England_NHSE	COPD	age_strat	01/06/2023	50-59		1830
England_NHSE	COPD	age_strat	01/06/2023	60-69		3020
England_NHSE	COPD	age_strat	01/06/2023	70+		3830
England_NHSE	COPD	region_strat	01/11/2019		East Midlands	910
England_NHSE	COPD	region_strat	01/11/2019		East of England	1030
England_NHSE	COPD	region_strat	01/11/2019		London	1105
England_NHSE	COPD	region_strat	01/11/2019		North East	620
England_NHSE	COPD	region_strat	01/11/2019		North West	1660
England_NHSE	COPD	region_strat	01/11/2019		South East	1440
England_NHSE	COPD	region_strat	01/11/2019		South West	990
England_NHSE	COPD	region_strat	01/11/2019		West Midlands	1100
England_NHSE	COPD	region_strat	01/11/2019		Yorkshire and The Humber	1115
England_NHSE	COPD	region_strat	01/12/2019		East Midlands	1010
England_NHSE	COPD	region_strat	01/12/2019		East of England	1145
England_NHSE	COPD	region_strat	01/12/2019		London	1065
England_NHSE	COPD	region_strat	01/12/2019		North East	630
England_NHSE	COPD	region_strat	01/12/2019		North West	1755
England_NHSE	COPD	region_strat	01/12/2019		South East	1490
England_NHSE	COPD	region_strat	01/12/2019		South West	1040
England_NHSE	COPD	region_strat	01/12/2019		West Midlands	1145
England_NHSE	COPD	region_strat	01/12/2019		Yorkshire and The Humber	1130
England_NHSE	COPD	region_strat	01/01/2020		East Midlands	1160
England_NHSE	COPD	region_strat	01/01/2020		East of England	1360
England_NHSE	COPD	region_strat	01/01/2020		London	1325

England_NHSE	COPD	region_strat	01/01/2020	North East	750
England_NHSE	COPD	region_strat	01/01/2020	North West	1880
England_NHSE	COPD	region_strat	01/01/2020	South East	1835
England_NHSE	COPD	region_strat	01/01/2020	South West	1290
England_NHSE	COPD	region_strat	01/01/2020	West Midlands	1285
England_NHSE	COPD	region_strat	01/01/2020	Yorkshire and The Humber	1425
England_NHSE	COPD	region_strat	01/02/2020	East Midlands	960
England_NHSE	COPD	region_strat	01/02/2020	East of England	1140
England_NHSE	COPD	region_strat	01/02/2020	London	1095
England_NHSE	COPD	region_strat	01/02/2020	North East	645
England_NHSE	COPD	region_strat	01/02/2020	North West	1590
England_NHSE	COPD	region_strat	01/02/2020	South East	1525
England_NHSE	COPD	region_strat	01/02/2020	South West	1060
England_NHSE	COPD	region_strat	01/02/2020	West Midlands	1105
England_NHSE	COPD	region_strat	01/02/2020	Yorkshire and The Humber	1150
England_NHSE	COPD	region_strat	01/03/2020	East Midlands	715
England_NHSE	COPD	region_strat	01/03/2020	East of England	940
England_NHSE	COPD	region_strat	01/03/2020	London	865
England_NHSE	COPD	region_strat	01/03/2020	North East	610
England_NHSE	COPD	region_strat	01/03/2020	North West	1450
England_NHSE	COPD	region_strat	01/03/2020	South East	1295
England_NHSE	COPD	region_strat	01/03/2020	South West	945
England_NHSE	COPD	region_strat	01/03/2020	West Midlands	990
England_NHSE	COPD	region_strat	01/03/2020	Yorkshire and The Humber	1040
England_NHSE	COPD	region_strat	01/04/2020	East Midlands	285
England_NHSE	COPD	region_strat	01/04/2020	East of England	390
England_NHSE	COPD	region_strat	01/04/2020	London	365
England_NHSE	COPD	region_strat	01/04/2020	North East	200
England_NHSE	COPD	region_strat	01/04/2020	North West	520
England_NHSE	COPD	region_strat	01/04/2020	South East	480
England_NHSE	COPD	region_strat	01/04/2020	South West	380
England_NHSE	COPD	region_strat	01/04/2020	West Midlands	345
England_NHSE	COPD	region_strat	01/04/2020	Yorkshire and The Humber	315
England_NHSE	COPD	region_strat	01/05/2020	East Midlands	305
England_NHSE	COPD	region_strat	01/05/2020	East of England	400
England_NHSE	COPD	region_strat	01/05/2020	London	315
England_NHSE	COPD	region_strat	01/05/2020	North East	170
England_NHSE	COPD	region_strat	01/05/2020	North West	430
England_NHSE	COPD	region_strat	01/05/2020	South East	405
England_NHSE	COPD	region_strat	01/05/2020	South West	325
England_NHSE	COPD	region_strat	01/05/2020	West Midlands	300
England_NHSE	COPD	region_strat	01/05/2020	Yorkshire and The Humber	300
England_NHSE	COPD	region_strat	01/06/2020	East Midlands	315
England_NHSE	COPD	region_strat	01/06/2020	East of England	400
England_NHSE	COPD	region_strat	01/06/2020	London	400
England_NHSE	COPD	region_strat	01/06/2020	North East	200
England_NHSE	COPD	region_strat	01/06/2020	North West	565
England_NHSE	COPD	region_strat	01/06/2020	South East	515
England_NHSE	COPD	region_strat	01/06/2020	South West	345
England_NHSE	COPD	region_strat	01/06/2020	West Midlands	380
England_NHSE	COPD	region_strat	01/06/2020	Yorkshire and The Humber	350
England_NHSE	COPD	region_strat	01/07/2020	East Midlands	350
England_NHSE	COPD	region_strat	01/07/2020	East of England	445
England_NHSE	COPD	region_strat	01/07/2020	London	385
England_NHSE	COPD	region_strat	01/07/2020	North East	220
England_NHSE	COPD	region_strat	01/07/2020	North West	510
England_NHSE	COPD	region_strat	01/07/2020	South East	575
England_NHSE	COPD	region_strat	01/07/2020	South West	410
England_NHSE	COPD	region_strat	01/07/2020	West Midlands	385
England_NHSE	COPD	region_strat	01/07/2020	Yorkshire and The Humber	400
England_NHSE	COPD	region_strat	01/08/2020	East Midlands	350
England_NHSE	COPD	region_strat	01/08/2020	East of England	450
England_NHSE	COPD	region_strat	01/08/2020	London	355

England_NHSE	COPD	region_strat	01/08/2020	North East	250
England_NHSE	COPD	region_strat	01/08/2020	North West	565
England_NHSE	COPD	region_strat	01/08/2020	South East	555
England_NHSE	COPD	region_strat	01/08/2020	South West	400
England_NHSE	COPD	region_strat	01/08/2020	West Midlands	385
England_NHSE	COPD	region_strat	01/08/2020	Yorkshire and The Humber	380
England_NHSE	COPD	region_strat	01/09/2020	East Midlands	420
England_NHSE	COPD	region_strat	01/09/2020	East of England	525
England_NHSE	COPD	region_strat	01/09/2020	London	470
England_NHSE	COPD	region_strat	01/09/2020	North East	295
England_NHSE	COPD	region_strat	01/09/2020	North West	670
England_NHSE	COPD	region_strat	01/09/2020	South East	695
England_NHSE	COPD	region_strat	01/09/2020	South West	475
England_NHSE	COPD	region_strat	01/09/2020	West Midlands	460
England_NHSE	COPD	region_strat	01/09/2020	Yorkshire and The Humber	470
England_NHSE	COPD	region_strat	01/10/2020	East Midlands	435
England_NHSE	COPD	region_strat	01/10/2020	East of England	530
England_NHSE	COPD	region_strat	01/10/2020	London	505
England_NHSE	COPD	region_strat	01/10/2020	North East	320
England_NHSE	COPD	region_strat	01/10/2020	North West	685
England_NHSE	COPD	region_strat	01/10/2020	South East	700
England_NHSE	COPD	region_strat	01/10/2020	South West	550
England_NHSE	COPD	region_strat	01/10/2020	West Midlands	480
England_NHSE	COPD	region_strat	01/10/2020	Yorkshire and The Humber	450
England_NHSE	COPD	region_strat	01/11/2020	East Midlands	455
England_NHSE	COPD	region_strat	01/11/2020	East of England	575
England_NHSE	COPD	region_strat	01/11/2020	London	450
England_NHSE	COPD	region_strat	01/11/2020	North East	310
England_NHSE	COPD	region_strat	01/11/2020	North West	685
England_NHSE	COPD	region_strat	01/11/2020	South East	745
England_NHSE	COPD	region_strat	01/11/2020	South West	480
England_NHSE	COPD	region_strat	01/11/2020	West Midlands	480
England_NHSE	COPD	region_strat	01/11/2020	Yorkshire and The Humber	450
England_NHSE	COPD	region_strat	01/12/2020	East Midlands	380
England_NHSE	COPD	region_strat	01/12/2020	East of England	540
England_NHSE	COPD	region_strat	01/12/2020	London	455
England_NHSE	COPD	region_strat	01/12/2020	North East	290
England_NHSE	COPD	region_strat	01/12/2020	North West	720
England_NHSE	COPD	region_strat	01/12/2020	South East	675
England_NHSE	COPD	region_strat	01/12/2020	South West	455
England_NHSE	COPD	region_strat	01/12/2020	West Midlands	435
England_NHSE	COPD	region_strat	01/12/2020	Yorkshire and The Humber	450
England_NHSE	COPD	region_strat	01/01/2021	East Midlands	400
England_NHSE	COPD	region_strat	01/01/2021	East of England	525
England_NHSE	COPD	region_strat	01/01/2021	London	450
England_NHSE	COPD	region_strat	01/01/2021	North East	265
England_NHSE	COPD	region_strat	01/01/2021	North West	725
England_NHSE	COPD	region_strat	01/01/2021	South East	715
England_NHSE	COPD	region_strat	01/01/2021	South West	570
England_NHSE	COPD	region_strat	01/01/2021	West Midlands	455
England_NHSE	COPD	region_strat	01/01/2021	Yorkshire and The Humber	495
England_NHSE	COPD	region_strat	01/02/2021	East Midlands	400
England_NHSE	COPD	region_strat	01/02/2021	East of England	455
England_NHSE	COPD	region_strat	01/02/2021	London	460
England_NHSE	COPD	region_strat	01/02/2021	North East	295
England_NHSE	COPD	region_strat	01/02/2021	North West	695
England_NHSE	COPD	region_strat	01/02/2021	South East	665
England_NHSE	COPD	region_strat	01/02/2021	South West	475
England_NHSE	COPD	region_strat	01/02/2021	West Midlands	480
England_NHSE	COPD	region_strat	01/02/2021	Yorkshire and The Humber	495
England_NHSE	COPD	region_strat	01/03/2021	East Midlands	515
England_NHSE	COPD	region_strat	01/03/2021	East of England	670
England_NHSE	COPD	region_strat	01/03/2021	London	690

England_NHSE	COPD	region_strat	01/03/2021	North East	320
England_NHSE	COPD	region_strat	01/03/2021	North West	780
England_NHSE	COPD	region_strat	01/03/2021	South East	860
England_NHSE	COPD	region_strat	01/03/2021	South West	545
England_NHSE	COPD	region_strat	01/03/2021	West Midlands	515
England_NHSE	COPD	region_strat	01/03/2021	Yorkshire and The Humber	590
England_NHSE	COPD	region_strat	01/04/2021	East Midlands	510
England_NHSE	COPD	region_strat	01/04/2021	East of England	535
England_NHSE	COPD	region_strat	01/04/2021	London	510
England_NHSE	COPD	region_strat	01/04/2021	North East	295
England_NHSE	COPD	region_strat	01/04/2021	North West	700
England_NHSE	COPD	region_strat	01/04/2021	South East	760
England_NHSE	COPD	region_strat	01/04/2021	South West	575
England_NHSE	COPD	region_strat	01/04/2021	West Midlands	510
England_NHSE	COPD	region_strat	01/04/2021	Yorkshire and The Humber	475
England_NHSE	COPD	region_strat	01/05/2021	East Midlands	590
England_NHSE	COPD	region_strat	01/05/2021	East of England	550
England_NHSE	COPD	region_strat	01/05/2021	London	510
England_NHSE	COPD	region_strat	01/05/2021	North East	355
England_NHSE	COPD	region_strat	01/05/2021	North West	760
England_NHSE	COPD	region_strat	01/05/2021	South East	825
England_NHSE	COPD	region_strat	01/05/2021	South West	545
England_NHSE	COPD	region_strat	01/05/2021	West Midlands	515
England_NHSE	COPD	region_strat	01/05/2021	Yorkshire and The Humber	600
England_NHSE	COPD	region_strat	01/06/2021	East Midlands	580
England_NHSE	COPD	region_strat	01/06/2021	East of England	685
England_NHSE	COPD	region_strat	01/06/2021	London	555
England_NHSE	COPD	region_strat	01/06/2021	North East	385
England_NHSE	COPD	region_strat	01/06/2021	North West	860
England_NHSE	COPD	region_strat	01/06/2021	South East	910
England_NHSE	COPD	region_strat	01/06/2021	South West	580
England_NHSE	COPD	region_strat	01/06/2021	West Midlands	610
England_NHSE	COPD	region_strat	01/06/2021	Yorkshire and The Humber	720
England_NHSE	COPD	region_strat	01/07/2021	East Midlands	535
England_NHSE	COPD	region_strat	01/07/2021	East of England	680
England_NHSE	COPD	region_strat	01/07/2021	London	555
England_NHSE	COPD	region_strat	01/07/2021	North East	355
England_NHSE	COPD	region_strat	01/07/2021	North West	875
England_NHSE	COPD	region_strat	01/07/2021	South East	960
England_NHSE	COPD	region_strat	01/07/2021	South West	575
England_NHSE	COPD	region_strat	01/07/2021	West Midlands	650
England_NHSE	COPD	region_strat	01/07/2021	Yorkshire and The Humber	730
England_NHSE	COPD	region_strat	01/08/2021	East Midlands	580
England_NHSE	COPD	region_strat	01/08/2021	East of England	625
England_NHSE	COPD	region_strat	01/08/2021	London	485
England_NHSE	COPD	region_strat	01/08/2021	North East	355
England_NHSE	COPD	region_strat	01/08/2021	North West	835
England_NHSE	COPD	region_strat	01/08/2021	South East	815
England_NHSE	COPD	region_strat	01/08/2021	South West	580
England_NHSE	COPD	region_strat	01/08/2021	West Midlands	550
England_NHSE	COPD	region_strat	01/08/2021	Yorkshire and The Humber	690
England_NHSE	COPD	region_strat	01/09/2021	East Midlands	585
England_NHSE	COPD	region_strat	01/09/2021	East of England	675
England_NHSE	COPD	region_strat	01/09/2021	London	600
England_NHSE	COPD	region_strat	01/09/2021	North East	385
England_NHSE	COPD	region_strat	01/09/2021	North West	980
England_NHSE	COPD	region_strat	01/09/2021	South East	945
England_NHSE	COPD	region_strat	01/09/2021	South West	645
England_NHSE	COPD	region_strat	01/09/2021	West Midlands	715
England_NHSE	COPD	region_strat	01/09/2021	Yorkshire and The Humber	855
England_NHSE	COPD	region_strat	01/10/2021	East Midlands	575
England_NHSE	COPD	region_strat	01/10/2021	East of England	715
England_NHSE	COPD	region_strat	01/10/2021	London	695

England_NHSE	COPD	region_strat	01/10/2021	North East	450
England_NHSE	COPD	region_strat	01/10/2021	North West	1075
England_NHSE	COPD	region_strat	01/10/2021	South East	1010
England_NHSE	COPD	region_strat	01/10/2021	South West	695
England_NHSE	COPD	region_strat	01/10/2021	West Midlands	725
England_NHSE	COPD	region_strat	01/10/2021	Yorkshire and The Humber	835
England_NHSE	COPD	region_strat	01/11/2021	East Midlands	655
England_NHSE	COPD	region_strat	01/11/2021	East of England	805
England_NHSE	COPD	region_strat	01/11/2021	London	785
England_NHSE	COPD	region_strat	01/11/2021	North East	440
England_NHSE	COPD	region_strat	01/11/2021	North West	1325
England_NHSE	COPD	region_strat	01/11/2021	South East	1075
England_NHSE	COPD	region_strat	01/11/2021	South West	760
England_NHSE	COPD	region_strat	01/11/2021	West Midlands	800
England_NHSE	COPD	region_strat	01/11/2021	Yorkshire and The Humber	945
England_NHSE	COPD	region_strat	01/12/2021	East Midlands	735
England_NHSE	COPD	region_strat	01/12/2021	East of England	735
England_NHSE	COPD	region_strat	01/12/2021	London	635
England_NHSE	COPD	region_strat	01/12/2021	North East	440
England_NHSE	COPD	region_strat	01/12/2021	North West	1220
England_NHSE	COPD	region_strat	01/12/2021	South East	1065
England_NHSE	COPD	region_strat	01/12/2021	South West	675
England_NHSE	COPD	region_strat	01/12/2021	West Midlands	745
England_NHSE	COPD	region_strat	01/12/2021	Yorkshire and The Humber	860
England_NHSE	COPD	region_strat	01/01/2022	East Midlands	675
England_NHSE	COPD	region_strat	01/01/2022	East of England	780
England_NHSE	COPD	region_strat	01/01/2022	London	630
England_NHSE	COPD	region_strat	01/01/2022	North East	395
England_NHSE	COPD	region_strat	01/01/2022	North West	1195
England_NHSE	COPD	region_strat	01/01/2022	South East	970
England_NHSE	COPD	region_strat	01/01/2022	South West	725
England_NHSE	COPD	region_strat	01/01/2022	West Midlands	675
England_NHSE	COPD	region_strat	01/01/2022	Yorkshire and The Humber	885
England_NHSE	COPD	region_strat	01/02/2022	East Midlands	645
England_NHSE	COPD	region_strat	01/02/2022	East of England	700
England_NHSE	COPD	region_strat	01/02/2022	London	595
England_NHSE	COPD	region_strat	01/02/2022	North East	355
England_NHSE	COPD	region_strat	01/02/2022	North West	1170
England_NHSE	COPD	region_strat	01/02/2022	South East	980
England_NHSE	COPD	region_strat	01/02/2022	South West	660
England_NHSE	COPD	region_strat	01/02/2022	West Midlands	740
England_NHSE	COPD	region_strat	01/02/2022	Yorkshire and The Humber	770
England_NHSE	COPD	region_strat	01/03/2022	East Midlands	760
England_NHSE	COPD	region_strat	01/03/2022	East of England	805
England_NHSE	COPD	region_strat	01/03/2022	London	675
England_NHSE	COPD	region_strat	01/03/2022	North East	470
England_NHSE	COPD	region_strat	01/03/2022	North West	1555
England_NHSE	COPD	region_strat	01/03/2022	South East	1120
England_NHSE	COPD	region_strat	01/03/2022	South West	795
England_NHSE	COPD	region_strat	01/03/2022	West Midlands	905
England_NHSE	COPD	region_strat	01/03/2022	Yorkshire and The Humber	975
England_NHSE	COPD	region_strat	01/04/2022	East Midlands	670
England_NHSE	COPD	region_strat	01/04/2022	East of England	685
England_NHSE	COPD	region_strat	01/04/2022	London	665
England_NHSE	COPD	region_strat	01/04/2022	North East	455
England_NHSE	COPD	region_strat	01/04/2022	North West	1310
England_NHSE	COPD	region_strat	01/04/2022	South East	925
England_NHSE	COPD	region_strat	01/04/2022	South West	680
England_NHSE	COPD	region_strat	01/04/2022	West Midlands	830
England_NHSE	COPD	region_strat	01/04/2022	Yorkshire and The Humber	855
England_NHSE	COPD	region_strat	01/05/2022	East Midlands	730
England_NHSE	COPD	region_strat	01/05/2022	East of England	735
England_NHSE	COPD	region_strat	01/05/2022	London	650

England_NHSE	COPD	region_strat	01/05/2022	North East	490
England_NHSE	COPD	region_strat	01/05/2022	North West	1490
England_NHSE	COPD	region_strat	01/05/2022	South East	1040
England_NHSE	COPD	region_strat	01/05/2022	South West	795
England_NHSE	COPD	region_strat	01/05/2022	West Midlands	1005
England_NHSE	COPD	region_strat	01/05/2022	Yorkshire and The Humber	955
England_NHSE	COPD	region_strat	01/06/2022	East Midlands	710
England_NHSE	COPD	region_strat	01/06/2022	East of England	795
England_NHSE	COPD	region_strat	01/06/2022	London	625
England_NHSE	COPD	region_strat	01/06/2022	North East	460
England_NHSE	COPD	region_strat	01/06/2022	North West	1310
England_NHSE	COPD	region_strat	01/06/2022	South East	1015
England_NHSE	COPD	region_strat	01/06/2022	South West	735
England_NHSE	COPD	region_strat	01/06/2022	West Midlands	945
England_NHSE	COPD	region_strat	01/06/2022	Yorkshire and The Humber	925
England_NHSE	COPD	region_strat	01/07/2022	East Midlands	685
England_NHSE	COPD	region_strat	01/07/2022	East of England	720
England_NHSE	COPD	region_strat	01/07/2022	London	665
England_NHSE	COPD	region_strat	01/07/2022	North East	475
England_NHSE	COPD	region_strat	01/07/2022	North West	1380
England_NHSE	COPD	region_strat	01/07/2022	South East	1185
England_NHSE	COPD	region_strat	01/07/2022	South West	755
England_NHSE	COPD	region_strat	01/07/2022	West Midlands	770
England_NHSE	COPD	region_strat	01/07/2022	Yorkshire and The Humber	930
England_NHSE	COPD	region_strat	01/08/2022	East Midlands	710
England_NHSE	COPD	region_strat	01/08/2022	East of England	785
England_NHSE	COPD	region_strat	01/08/2022	London	720
England_NHSE	COPD	region_strat	01/08/2022	North East	490
England_NHSE	COPD	region_strat	01/08/2022	North West	1325
England_NHSE	COPD	region_strat	01/08/2022	South East	1120
England_NHSE	COPD	region_strat	01/08/2022	South West	735
England_NHSE	COPD	region_strat	01/08/2022	West Midlands	760
England_NHSE	COPD	region_strat	01/08/2022	Yorkshire and The Humber	925
England_NHSE	COPD	region_strat	01/09/2022	East Midlands	915
England_NHSE	COPD	region_strat	01/09/2022	East of England	805
England_NHSE	COPD	region_strat	01/09/2022	London	675
England_NHSE	COPD	region_strat	01/09/2022	North East	480
England_NHSE	COPD	region_strat	01/09/2022	North West	1215
England_NHSE	COPD	region_strat	01/09/2022	South East	1085
England_NHSE	COPD	region_strat	01/09/2022	South West	820
England_NHSE	COPD	region_strat	01/09/2022	West Midlands	795
England_NHSE	COPD	region_strat	01/09/2022	Yorkshire and The Humber	860
England_NHSE	COPD	region_strat	01/10/2022	East Midlands	865
England_NHSE	COPD	region_strat	01/10/2022	East of England	820
England_NHSE	COPD	region_strat	01/10/2022	London	770
England_NHSE	COPD	region_strat	01/10/2022	North East	570
England_NHSE	COPD	region_strat	01/10/2022	North West	1280
England_NHSE	COPD	region_strat	01/10/2022	South East	1200
England_NHSE	COPD	region_strat	01/10/2022	South West	990
England_NHSE	COPD	region_strat	01/10/2022	West Midlands	925
England_NHSE	COPD	region_strat	01/10/2022	Yorkshire and The Humber	955
England_NHSE	COPD	region_strat	01/11/2022	East Midlands	1025
England_NHSE	COPD	region_strat	01/11/2022	East of England	925
England_NHSE	COPD	region_strat	01/11/2022	London	790
England_NHSE	COPD	region_strat	01/11/2022	North East	615
England_NHSE	COPD	region_strat	01/11/2022	North West	1545
England_NHSE	COPD	region_strat	01/11/2022	South East	1445
England_NHSE	COPD	region_strat	01/11/2022	South West	1120
England_NHSE	COPD	region_strat	01/11/2022	West Midlands	1035
England_NHSE	COPD	region_strat	01/11/2022	Yorkshire and The Humber	1145
England_NHSE	COPD	region_strat	01/12/2022	East Midlands	935
England_NHSE	COPD	region_strat	01/12/2022	East of England	965
England_NHSE	COPD	region_strat	01/12/2022	London	820

England_NHSE	COPD	region_strat	01/12/2022	North East	625
England_NHSE	COPD	region_strat	01/12/2022	North West	1500
England_NHSE	COPD	region_strat	01/12/2022	South East	1410
England_NHSE	COPD	region_strat	01/12/2022	South West	995
England_NHSE	COPD	region_strat	01/12/2022	West Midlands	1030
England_NHSE	COPD	region_strat	01/12/2022	Yorkshire and The Humber	1235
England_NHSE	COPD	region_strat	01/01/2023	East Midlands	1105
England_NHSE	COPD	region_strat	01/01/2023	East of England	1145
England_NHSE	COPD	region_strat	01/01/2023	London	820
England_NHSE	COPD	region_strat	01/01/2023	North East	735
England_NHSE	COPD	region_strat	01/01/2023	North West	1535
England_NHSE	COPD	region_strat	01/01/2023	South East	1510
England_NHSE	COPD	region_strat	01/01/2023	South West	1135
England_NHSE	COPD	region_strat	01/01/2023	West Midlands	1000
England_NHSE	COPD	region_strat	01/01/2023	Yorkshire and The Humber	1265
England_NHSE	COPD	region_strat	01/02/2023	East Midlands	1025
England_NHSE	COPD	region_strat	01/02/2023	East of England	945
England_NHSE	COPD	region_strat	01/02/2023	London	755
England_NHSE	COPD	region_strat	01/02/2023	North East	760
England_NHSE	COPD	region_strat	01/02/2023	North West	1310
England_NHSE	COPD	region_strat	01/02/2023	South East	1270
England_NHSE	COPD	region_strat	01/02/2023	South West	980
England_NHSE	COPD	region_strat	01/02/2023	West Midlands	930
England_NHSE	COPD	region_strat	01/02/2023	Yorkshire and The Humber	1320
England_NHSE	COPD	region_strat	01/03/2023	East Midlands	1115
England_NHSE	COPD	region_strat	01/03/2023	East of England	1145
England_NHSE	COPD	region_strat	01/03/2023	London	910
England_NHSE	COPD	region_strat	01/03/2023	North East	735
England_NHSE	COPD	region_strat	01/03/2023	North West	1590
England_NHSE	COPD	region_strat	01/03/2023	South East	1535
England_NHSE	COPD	region_strat	01/03/2023	South West	1160
England_NHSE	COPD	region_strat	01/03/2023	West Midlands	1145
England_NHSE	COPD	region_strat	01/03/2023	Yorkshire and The Humber	1350
England_NHSE	COPD	region_strat	01/04/2023	East Midlands	800
England_NHSE	COPD	region_strat	01/04/2023	East of England	815
England_NHSE	COPD	region_strat	01/04/2023	London	750
England_NHSE	COPD	region_strat	01/04/2023	North East	635
England_NHSE	COPD	region_strat	01/04/2023	North West	1295
England_NHSE	COPD	region_strat	01/04/2023	South East	1195
England_NHSE	COPD	region_strat	01/04/2023	South West	980
England_NHSE	COPD	region_strat	01/04/2023	West Midlands	905
England_NHSE	COPD	region_strat	01/04/2023	Yorkshire and The Humber	1050
England_NHSE	COPD	region_strat	01/05/2023	East Midlands	855
England_NHSE	COPD	region_strat	01/05/2023	East of England	895
England_NHSE	COPD	region_strat	01/05/2023	London	755
England_NHSE	COPD	region_strat	01/05/2023	North East	645
England_NHSE	COPD	region_strat	01/05/2023	North West	1355
England_NHSE	COPD	region_strat	01/05/2023	South East	1420
England_NHSE	COPD	region_strat	01/05/2023	South West	1030
England_NHSE	COPD	region_strat	01/05/2023	West Midlands	1010
England_NHSE	COPD	region_strat	01/05/2023	Yorkshire and The Humber	1200
England_NHSE	COPD	region_strat	01/06/2023	East Midlands	850
England_NHSE	COPD	region_strat	01/06/2023	East of England	1195
England_NHSE	COPD	region_strat	01/06/2023	London	765
England_NHSE	COPD	region_strat	01/06/2023	North East	635
England_NHSE	COPD	region_strat	01/06/2023	North West	1380
England_NHSE	COPD	region_strat	01/06/2023	South East	1425
England_NHSE	COPD	region_strat	01/06/2023	South West	970
England_NHSE	COPD	region_strat	01/06/2023	West Midlands	920
England_NHSE	COPD	region_strat	01/06/2023	Yorkshire and The Humber	1145
England_NHSE	ILD	all	01/11/2019		1860
England_NHSE	ILD	all	01/12/2019		1630
England_NHSE	ILD	all	01/01/2020		2085

England_NHSE	ILD	all	01/02/2020		1870
England_NHSE	ILD	all	01/03/2020		1670
England_NHSE	ILD	all	01/04/2020		1060
England_NHSE	ILD	all	01/05/2020		980
England_NHSE	ILD	all	01/06/2020		1255
England_NHSE	ILD	all	01/07/2020		1390
England_NHSE	ILD	all	01/08/2020		1280
England_NHSE	ILD	all	01/09/2020		1450
England_NHSE	ILD	all	01/10/2020		1550
England_NHSE	ILD	all	01/11/2020		1530
England_NHSE	ILD	all	01/12/2020		1390
England_NHSE	ILD	all	01/01/2021		1435
England_NHSE	ILD	all	01/02/2021		1320
England_NHSE	ILD	all	01/03/2021		1715
England_NHSE	ILD	all	01/04/2021		1610
England_NHSE	ILD	all	01/05/2021		1650
England_NHSE	ILD	all	01/06/2021		1765
England_NHSE	ILD	all	01/07/2021		1680
England_NHSE	ILD	all	01/08/2021		1590
England_NHSE	ILD	all	01/09/2021		1735
England_NHSE	ILD	all	01/10/2021		1695
England_NHSE	ILD	all	01/11/2021		1870
England_NHSE	ILD	all	01/12/2021		1785
England_NHSE	ILD	all	01/01/2022		1805
England_NHSE	ILD	all	01/02/2022		1770
England_NHSE	ILD	all	01/03/2022		2045
England_NHSE	ILD	all	01/04/2022		1665
England_NHSE	ILD	all	01/05/2022		2035
England_NHSE	ILD	all	01/06/2022		1790
England_NHSE	ILD	all	01/07/2022		1670
England_NHSE	ILD	all	01/08/2022		1785
England_NHSE	ILD	all	01/09/2022		1795
England_NHSE	ILD	all	01/10/2022		1750
England_NHSE	ILD	all	01/11/2022		1995
England_NHSE	ILD	all	01/12/2022		1610
England_NHSE	ILD	all	01/01/2023		1940
England_NHSE	ILD	all	01/02/2023		1795
England_NHSE	ILD	all	01/03/2023		2030
England_NHSE	ILD	all	01/04/2023		1580
England_NHSE	ILD	all	01/05/2023		1890
England_NHSE	ILD	all	01/06/2023		1740
England_NHSE	ILD	sex_strat	01/11/2019	Male	1050
England_NHSE	ILD	sex_strat	01/11/2019	Female	810
England_NHSE	ILD	sex_strat	01/12/2019	Male	955
England_NHSE	ILD	sex_strat	01/12/2019	Female	675
England_NHSE	ILD	sex_strat	01/01/2020	Male	1235
England_NHSE	ILD	sex_strat	01/01/2020	Female	850
England_NHSE	ILD	sex_strat	01/02/2020	Male	1110
England_NHSE	ILD	sex_strat	01/02/2020	Female	760
England_NHSE	ILD	sex_strat	01/03/2020	Male	985
England_NHSE	ILD	sex_strat	01/03/2020	Female	690
England_NHSE	ILD	sex_strat	01/04/2020	Male	600
England_NHSE	ILD	sex_strat	01/04/2020	Female	460
England_NHSE	ILD	sex_strat	01/05/2020	Male	585
England_NHSE	ILD	sex_strat	01/05/2020	Female	395
England_NHSE	ILD	sex_strat	01/06/2020	Male	765
England_NHSE	ILD	sex_strat	01/06/2020	Female	490
England_NHSE	ILD	sex_strat	01/07/2020	Male	825
England_NHSE	ILD	sex_strat	01/07/2020	Female	565
England_NHSE	ILD	sex_strat	01/08/2020	Male	765
England_NHSE	ILD	sex_strat	01/08/2020	Female	515
England_NHSE	ILD	sex_strat	01/09/2020	Male	835
England_NHSE	ILD	sex_strat	01/09/2020	Female	615

England_NHSE	ILD	sex_strat	01/10/2020	Male	930
England_NHSE	ILD	sex_strat	01/10/2020	Female	625
England_NHSE	ILD	sex_strat	01/11/2020	Male	925
England_NHSE	ILD	sex_strat	01/11/2020	Female	605
England_NHSE	ILD	sex_strat	01/12/2020	Male	850
England_NHSE	ILD	sex_strat	01/12/2020	Female	540
England_NHSE	ILD	sex_strat	01/01/2021	Male	860
England_NHSE	ILD	sex_strat	01/01/2021	Female	575
England_NHSE	ILD	sex_strat	01/02/2021	Male	825
England_NHSE	ILD	sex_strat	01/02/2021	Female	495
England_NHSE	ILD	sex_strat	01/03/2021	Male	1060
England_NHSE	ILD	sex_strat	01/03/2021	Female	655
England_NHSE	ILD	sex_strat	01/04/2021	Male	980
England_NHSE	ILD	sex_strat	01/04/2021	Female	630
England_NHSE	ILD	sex_strat	01/05/2021	Male	980
England_NHSE	ILD	sex_strat	01/05/2021	Female	670
England_NHSE	ILD	sex_strat	01/06/2021	Male	1010
England_NHSE	ILD	sex_strat	01/06/2021	Female	755
England_NHSE	ILD	sex_strat	01/07/2021	Male	1005
England_NHSE	ILD	sex_strat	01/07/2021	Female	675
England_NHSE	ILD	sex_strat	01/08/2021	Male	955
England_NHSE	ILD	sex_strat	01/08/2021	Female	635
England_NHSE	ILD	sex_strat	01/09/2021	Male	1065
England_NHSE	ILD	sex_strat	01/09/2021	Female	665
England_NHSE	ILD	sex_strat	01/10/2021	Male	1010
England_NHSE	ILD	sex_strat	01/10/2021	Female	685
England_NHSE	ILD	sex_strat	01/11/2021	Male	1120
England_NHSE	ILD	sex_strat	01/11/2021	Female	750
England_NHSE	ILD	sex_strat	01/12/2021	Male	1070
England_NHSE	ILD	sex_strat	01/12/2021	Female	715
England_NHSE	ILD	sex_strat	01/01/2022	Male	1095
England_NHSE	ILD	sex_strat	01/01/2022	Female	715
England_NHSE	ILD	sex_strat	01/02/2022	Male	1050
England_NHSE	ILD	sex_strat	01/02/2022	Female	725
England_NHSE	ILD	sex_strat	01/03/2022	Male	1275
England_NHSE	ILD	sex_strat	01/03/2022	Female	770
England_NHSE	ILD	sex_strat	01/04/2022	Male	1010
England_NHSE	ILD	sex_strat	01/04/2022	Female	655
England_NHSE	ILD	sex_strat	01/05/2022	Male	1210
England_NHSE	ILD	sex_strat	01/05/2022	Female	825
England_NHSE	ILD	sex_strat	01/06/2022	Male	1115
England_NHSE	ILD	sex_strat	01/06/2022	Female	675
England_NHSE	ILD	sex_strat	01/07/2022	Male	1005
England_NHSE	ILD	sex_strat	01/07/2022	Female	665
England_NHSE	ILD	sex_strat	01/08/2022	Male	1115
England_NHSE	ILD	sex_strat	01/08/2022	Female	665
England_NHSE	ILD	sex_strat	01/09/2022	Male	1065
England_NHSE	ILD	sex_strat	01/09/2022	Female	735
England_NHSE	ILD	sex_strat	01/10/2022	Male	1035
England_NHSE	ILD	sex_strat	01/10/2022	Female	715
England_NHSE	ILD	sex_strat	01/11/2022	Male	1160
England_NHSE	ILD	sex_strat	01/11/2022	Female	835
England_NHSE	ILD	sex_strat	01/12/2022	Male	960
England_NHSE	ILD	sex_strat	01/12/2022	Female	650
England_NHSE	ILD	sex_strat	01/01/2023	Male	1150
England_NHSE	ILD	sex_strat	01/01/2023	Female	790
England_NHSE	ILD	sex_strat	01/02/2023	Male	1065
England_NHSE	ILD	sex_strat	01/02/2023	Female	730
England_NHSE	ILD	sex_strat	01/03/2023	Male	1260
England_NHSE	ILD	sex_strat	01/03/2023	Female	770
England_NHSE	ILD	sex_strat	01/04/2023	Male	960
England_NHSE	ILD	sex_strat	01/04/2023	Female	620
England_NHSE	ILD	sex_strat	01/05/2023	Male	1180

England_NHSE	ILD	sex_strat	01/05/2023	Female		710
England_NHSE	ILD	sex_strat	01/06/2023	Male		1035
England_NHSE	ILD	sex_strat	01/06/2023	Female		705
England_NHSE	ILD	age_strat	01/11/2019		40-49	95
England_NHSE	ILD	age_strat	01/11/2019		50-59	205
England_NHSE	ILD	age_strat	01/11/2019		60-69	365
England_NHSE	ILD	age_strat	01/11/2019		70+	1195
England_NHSE	ILD	age_strat	01/12/2019		40-49	90
England_NHSE	ILD	age_strat	01/12/2019		50-59	160
England_NHSE	ILD	age_strat	01/12/2019		60-69	310
England_NHSE	ILD	age_strat	01/12/2019		70+	1070
England_NHSE	ILD	age_strat	01/01/2020		40-49	115
England_NHSE	ILD	age_strat	01/01/2020		50-59	240
England_NHSE	ILD	age_strat	01/01/2020		60-69	435
England_NHSE	ILD	age_strat	01/01/2020		70+	1300
England_NHSE	ILD	age_strat	01/02/2020		40-49	85
England_NHSE	ILD	age_strat	01/02/2020		50-59	185
England_NHSE	ILD	age_strat	01/02/2020		60-69	400
England_NHSE	ILD	age_strat	01/02/2020		70+	1205
England_NHSE	ILD	age_strat	01/03/2020		40-49	90
England_NHSE	ILD	age_strat	01/03/2020		50-59	200
England_NHSE	ILD	age_strat	01/03/2020		60-69	365
England_NHSE	ILD	age_strat	01/03/2020		70+	1020
England_NHSE	ILD	age_strat	01/04/2020		40-49	60
England_NHSE	ILD	age_strat	01/04/2020		50-59	130
England_NHSE	ILD	age_strat	01/04/2020		60-69	220
England_NHSE	ILD	age_strat	01/04/2020		70+	650
England_NHSE	ILD	age_strat	01/05/2020		40-49	65
England_NHSE	ILD	age_strat	01/05/2020		50-59	100
England_NHSE	ILD	age_strat	01/05/2020		60-69	190
England_NHSE	ILD	age_strat	01/05/2020		70+	630
England_NHSE	ILD	age_strat	01/06/2020		40-49	80
England_NHSE	ILD	age_strat	01/06/2020		50-59	135
England_NHSE	ILD	age_strat	01/06/2020		60-69	250
England_NHSE	ILD	age_strat	01/06/2020		70+	790
England_NHSE	ILD	age_strat	01/07/2020		40-49	70
England_NHSE	ILD	age_strat	01/07/2020		50-59	160
England_NHSE	ILD	age_strat	01/07/2020		60-69	300
England_NHSE	ILD	age_strat	01/07/2020		70+	865
England_NHSE	ILD	age_strat	01/08/2020		40-49	60
England_NHSE	ILD	age_strat	01/08/2020		50-59	180
England_NHSE	ILD	age_strat	01/08/2020		60-69	225
England_NHSE	ILD	age_strat	01/08/2020		70+	815
England_NHSE	ILD	age_strat	01/09/2020		40-49	75
England_NHSE	ILD	age_strat	01/09/2020		50-59	175
England_NHSE	ILD	age_strat	01/09/2020		60-69	305
England_NHSE	ILD	age_strat	01/09/2020		70+	890
England_NHSE	ILD	age_strat	01/10/2020		40-49	85
England_NHSE	ILD	age_strat	01/10/2020		50-59	190
England_NHSE	ILD	age_strat	01/10/2020		60-69	265
England_NHSE	ILD	age_strat	01/10/2020		70+	1015
England_NHSE	ILD	age_strat	01/11/2020		40-49	80
England_NHSE	ILD	age_strat	01/11/2020		50-59	165
England_NHSE	ILD	age_strat	01/11/2020		60-69	305
England_NHSE	ILD	age_strat	01/11/2020		70+	975
England_NHSE	ILD	age_strat	01/12/2020		40-49	70
England_NHSE	ILD	age_strat	01/12/2020		50-59	165
England_NHSE	ILD	age_strat	01/12/2020		60-69	280
England_NHSE	ILD	age_strat	01/12/2020		70+	875
England_NHSE	ILD	age_strat	01/01/2021		40-49	85
England_NHSE	ILD	age_strat	01/01/2021		50-59	200
England_NHSE	ILD	age_strat	01/01/2021		60-69	300
England_NHSE	ILD	age_strat	01/01/2021		70+	855

England_NHSE	ILD	age_strat	01/02/2021	40-49	80
England_NHSE	ILD	age_strat	01/02/2021	50-59	170
England_NHSE	ILD	age_strat	01/02/2021	60-69	290
England_NHSE	ILD	age_strat	01/02/2021	70+	785
England_NHSE	ILD	age_strat	01/03/2021	40-49	90
England_NHSE	ILD	age_strat	01/03/2021	50-59	240
England_NHSE	ILD	age_strat	01/03/2021	60-69	360
England_NHSE	ILD	age_strat	01/03/2021	70+	1025
England_NHSE	ILD	age_strat	01/04/2021	40-49	100
England_NHSE	ILD	age_strat	01/04/2021	50-59	195
England_NHSE	ILD	age_strat	01/04/2021	60-69	320
England_NHSE	ILD	age_strat	01/04/2021	70+	1000
England_NHSE	ILD	age_strat	01/05/2021	40-49	90
England_NHSE	ILD	age_strat	01/05/2021	50-59	190
England_NHSE	ILD	age_strat	01/05/2021	60-69	335
England_NHSE	ILD	age_strat	01/05/2021	70+	1030
England_NHSE	ILD	age_strat	01/06/2021	40-49	90
England_NHSE	ILD	age_strat	01/06/2021	50-59	210
England_NHSE	ILD	age_strat	01/06/2021	60-69	350
England_NHSE	ILD	age_strat	01/06/2021	70+	1115
England_NHSE	ILD	age_strat	01/07/2021	40-49	85
England_NHSE	ILD	age_strat	01/07/2021	50-59	190
England_NHSE	ILD	age_strat	01/07/2021	60-69	355
England_NHSE	ILD	age_strat	01/07/2021	70+	1050
England_NHSE	ILD	age_strat	01/08/2021	40-49	85
England_NHSE	ILD	age_strat	01/08/2021	50-59	185
England_NHSE	ILD	age_strat	01/08/2021	60-69	345
England_NHSE	ILD	age_strat	01/08/2021	70+	975
England_NHSE	ILD	age_strat	01/09/2021	40-49	95
England_NHSE	ILD	age_strat	01/09/2021	50-59	175
England_NHSE	ILD	age_strat	01/09/2021	60-69	350
England_NHSE	ILD	age_strat	01/09/2021	70+	1110
England_NHSE	ILD	age_strat	01/10/2021	40-49	80
England_NHSE	ILD	age_strat	01/10/2021	50-59	190
England_NHSE	ILD	age_strat	01/10/2021	60-69	345
England_NHSE	ILD	age_strat	01/10/2021	70+	1080
England_NHSE	ILD	age_strat	01/11/2021	40-49	100
England_NHSE	ILD	age_strat	01/11/2021	50-59	205
England_NHSE	ILD	age_strat	01/11/2021	60-69	380
England_NHSE	ILD	age_strat	01/11/2021	70+	1185
England_NHSE	ILD	age_strat	01/12/2021	40-49	95
England_NHSE	ILD	age_strat	01/12/2021	50-59	210
England_NHSE	ILD	age_strat	01/12/2021	60-69	360
England_NHSE	ILD	age_strat	01/12/2021	70+	1125
England_NHSE	ILD	age_strat	01/01/2022	40-49	85
England_NHSE	ILD	age_strat	01/01/2022	50-59	185
England_NHSE	ILD	age_strat	01/01/2022	60-69	345
England_NHSE	ILD	age_strat	01/01/2022	70+	1195
England_NHSE	ILD	age_strat	01/02/2022	40-49	75
England_NHSE	ILD	age_strat	01/02/2022	50-59	150
England_NHSE	ILD	age_strat	01/02/2022	60-69	390
England_NHSE	ILD	age_strat	01/02/2022	70+	1160
England_NHSE	ILD	age_strat	01/03/2022	40-49	90
England_NHSE	ILD	age_strat	01/03/2022	50-59	215
England_NHSE	ILD	age_strat	01/03/2022	60-69	415
England_NHSE	ILD	age_strat	01/03/2022	70+	1325
England_NHSE	ILD	age_strat	01/04/2022	40-49	70
England_NHSE	ILD	age_strat	01/04/2022	50-59	155
England_NHSE	ILD	age_strat	01/04/2022	60-69	315
England_NHSE	ILD	age_strat	01/04/2022	70+	1125
England_NHSE	ILD	age_strat	01/05/2022	40-49	100
England_NHSE	ILD	age_strat	01/05/2022	50-59	215
England_NHSE	ILD	age_strat	01/05/2022	60-69	415

England_NHSE	ILD	age_strat	01/05/2022	70+		1300
England_NHSE	ILD	age_strat	01/06/2022	40-49		80
England_NHSE	ILD	age_strat	01/06/2022	50-59		195
England_NHSE	ILD	age_strat	01/06/2022	60-69		350
England_NHSE	ILD	age_strat	01/06/2022	70+		1160
England_NHSE	ILD	age_strat	01/07/2022	40-49		70
England_NHSE	ILD	age_strat	01/07/2022	50-59		175
England_NHSE	ILD	age_strat	01/07/2022	60-69		340
England_NHSE	ILD	age_strat	01/07/2022	70+		1085
England_NHSE	ILD	age_strat	01/08/2022	40-49		85
England_NHSE	ILD	age_strat	01/08/2022	50-59		170
England_NHSE	ILD	age_strat	01/08/2022	60-69		385
England_NHSE	ILD	age_strat	01/08/2022	70+		1145
England_NHSE	ILD	age_strat	01/09/2022	40-49		85
England_NHSE	ILD	age_strat	01/09/2022	50-59		195
England_NHSE	ILD	age_strat	01/09/2022	60-69		325
England_NHSE	ILD	age_strat	01/09/2022	70+		1190
England_NHSE	ILD	age_strat	01/10/2022	40-49		85
England_NHSE	ILD	age_strat	01/10/2022	50-59		180
England_NHSE	ILD	age_strat	01/10/2022	60-69		350
England_NHSE	ILD	age_strat	01/10/2022	70+		1135
England_NHSE	ILD	age_strat	01/11/2022	40-49		85
England_NHSE	ILD	age_strat	01/11/2022	50-59		205
England_NHSE	ILD	age_strat	01/11/2022	60-69		365
England_NHSE	ILD	age_strat	01/11/2022	70+		1340
England_NHSE	ILD	age_strat	01/12/2022	40-49		65
England_NHSE	ILD	age_strat	01/12/2022	50-59		160
England_NHSE	ILD	age_strat	01/12/2022	60-69		370
England_NHSE	ILD	age_strat	01/12/2022	70+		1015
England_NHSE	ILD	age_strat	01/01/2023	40-49		85
England_NHSE	ILD	age_strat	01/01/2023	50-59		190
England_NHSE	ILD	age_strat	01/01/2023	60-69		430
England_NHSE	ILD	age_strat	01/01/2023	70+		1235
England_NHSE	ILD	age_strat	01/02/2023	40-49		90
England_NHSE	ILD	age_strat	01/02/2023	50-59		185
England_NHSE	ILD	age_strat	01/02/2023	60-69		345
England_NHSE	ILD	age_strat	01/02/2023	70+		1170
England_NHSE	ILD	age_strat	01/03/2023	40-49		75
England_NHSE	ILD	age_strat	01/03/2023	50-59		215
England_NHSE	ILD	age_strat	01/03/2023	60-69		390
England_NHSE	ILD	age_strat	01/03/2023	70+		1345
England_NHSE	ILD	age_strat	01/04/2023	40-49		70
England_NHSE	ILD	age_strat	01/04/2023	50-59		155
England_NHSE	ILD	age_strat	01/04/2023	60-69		330
England_NHSE	ILD	age_strat	01/04/2023	70+		1020
England_NHSE	ILD	age_strat	01/05/2023	40-49		90
England_NHSE	ILD	age_strat	01/05/2023	50-59		165
England_NHSE	ILD	age_strat	01/05/2023	60-69		365
England_NHSE	ILD	age_strat	01/05/2023	70+		1275
England_NHSE	ILD	age_strat	01/06/2023	40-49		65
England_NHSE	ILD	age_strat	01/06/2023	50-59		145
England_NHSE	ILD	age_strat	01/06/2023	60-69		365
England_NHSE	ILD	age_strat	01/06/2023	70+		1170
England_NHSE	ILD	region_strat	01/11/2019		East Midlands	180
England_NHSE	ILD	region_strat	01/11/2019		East of England	170
England_NHSE	ILD	region_strat	01/11/2019		London	210
England_NHSE	ILD	region_strat	01/11/2019		North East	125
England_NHSE	ILD	region_strat	01/11/2019		North West	280
England_NHSE	ILD	region_strat	01/11/2019		South East	335
England_NHSE	ILD	region_strat	01/11/2019		South West	200
England_NHSE	ILD	region_strat	01/11/2019		West Midlands	180
England_NHSE	ILD	region_strat	01/11/2019		Yorkshire and The Humber	180
England_NHSE	ILD	region_strat	01/12/2019		East Midlands	130

England_NHSE	ILD	region_strat	01/12/2019	East of England	175
England_NHSE	ILD	region_strat	01/12/2019	London	150
England_NHSE	ILD	region_strat	01/12/2019	North East	145
England_NHSE	ILD	region_strat	01/12/2019	North West	260
England_NHSE	ILD	region_strat	01/12/2019	South East	270
England_NHSE	ILD	region_strat	01/12/2019	South West	165
England_NHSE	ILD	region_strat	01/12/2019	West Midlands	185
England_NHSE	ILD	region_strat	01/12/2019	Yorkshire and The Humber	155
England_NHSE	ILD	region_strat	01/01/2020	East Midlands	205
England_NHSE	ILD	region_strat	01/01/2020	East of England	230
England_NHSE	ILD	region_strat	01/01/2020	London	240
England_NHSE	ILD	region_strat	01/01/2020	North East	170
England_NHSE	ILD	region_strat	01/01/2020	North West	315
England_NHSE	ILD	region_strat	01/01/2020	South East	300
England_NHSE	ILD	region_strat	01/01/2020	South West	220
England_NHSE	ILD	region_strat	01/01/2020	West Midlands	205
England_NHSE	ILD	region_strat	01/01/2020	Yorkshire and The Humber	200
England_NHSE	ILD	region_strat	01/02/2020	East Midlands	165
England_NHSE	ILD	region_strat	01/02/2020	East of England	190
England_NHSE	ILD	region_strat	01/02/2020	London	195
England_NHSE	ILD	region_strat	01/02/2020	North East	125
England_NHSE	ILD	region_strat	01/02/2020	North West	305
England_NHSE	ILD	region_strat	01/02/2020	South East	315
England_NHSE	ILD	region_strat	01/02/2020	South West	195
England_NHSE	ILD	region_strat	01/02/2020	West Midlands	185
England_NHSE	ILD	region_strat	01/02/2020	Yorkshire and The Humber	195
England_NHSE	ILD	region_strat	01/03/2020	East Midlands	160
England_NHSE	ILD	region_strat	01/03/2020	East of England	185
England_NHSE	ILD	region_strat	01/03/2020	London	180
England_NHSE	ILD	region_strat	01/03/2020	North East	105
England_NHSE	ILD	region_strat	01/03/2020	North West	245
England_NHSE	ILD	region_strat	01/03/2020	South East	255
England_NHSE	ILD	region_strat	01/03/2020	South West	180
England_NHSE	ILD	region_strat	01/03/2020	West Midlands	195
England_NHSE	ILD	region_strat	01/03/2020	Yorkshire and The Humber	170
England_NHSE	ILD	region_strat	01/04/2020	East Midlands	105
England_NHSE	ILD	region_strat	01/04/2020	East of England	110
England_NHSE	ILD	region_strat	01/04/2020	London	105
England_NHSE	ILD	region_strat	01/04/2020	North East	65
England_NHSE	ILD	region_strat	01/04/2020	North West	160
England_NHSE	ILD	region_strat	01/04/2020	South East	195
England_NHSE	ILD	region_strat	01/04/2020	South West	120
England_NHSE	ILD	region_strat	01/04/2020	West Midlands	115
England_NHSE	ILD	region_strat	01/04/2020	Yorkshire and The Humber	80
England_NHSE	ILD	region_strat	01/05/2020	East Midlands	110
England_NHSE	ILD	region_strat	01/05/2020	East of England	105
England_NHSE	ILD	region_strat	01/05/2020	London	105
England_NHSE	ILD	region_strat	01/05/2020	North East	55
England_NHSE	ILD	region_strat	01/05/2020	North West	155
England_NHSE	ILD	region_strat	01/05/2020	South East	150
England_NHSE	ILD	region_strat	01/05/2020	South West	110
England_NHSE	ILD	region_strat	01/05/2020	West Midlands	100
England_NHSE	ILD	region_strat	01/05/2020	Yorkshire and The Humber	90
England_NHSE	ILD	region_strat	01/06/2020	East Midlands	105
England_NHSE	ILD	region_strat	01/06/2020	East of England	135
England_NHSE	ILD	region_strat	01/06/2020	London	140
England_NHSE	ILD	region_strat	01/06/2020	North East	90
England_NHSE	ILD	region_strat	01/06/2020	North West	205
England_NHSE	ILD	region_strat	01/06/2020	South East	215
England_NHSE	ILD	region_strat	01/06/2020	South West	125
England_NHSE	ILD	region_strat	01/06/2020	West Midlands	125
England_NHSE	ILD	region_strat	01/06/2020	Yorkshire and The Humber	115
England_NHSE	ILD	region_strat	01/07/2020	East Midlands	140

England_NHSE	ILD	region_strat	01/07/2020	East of England	165
England_NHSE	ILD	region_strat	01/07/2020	London	165
England_NHSE	ILD	region_strat	01/07/2020	North East	85
England_NHSE	ILD	region_strat	01/07/2020	North West	190
England_NHSE	ILD	region_strat	01/07/2020	South East	225
England_NHSE	ILD	region_strat	01/07/2020	South West	130
England_NHSE	ILD	region_strat	01/07/2020	West Midlands	155
England_NHSE	ILD	region_strat	01/07/2020	Yorkshire and The Humber	130
England_NHSE	ILD	region_strat	01/08/2020	East Midlands	120
England_NHSE	ILD	region_strat	01/08/2020	East of England	140
England_NHSE	ILD	region_strat	01/08/2020	London	135
England_NHSE	ILD	region_strat	01/08/2020	North East	80
England_NHSE	ILD	region_strat	01/08/2020	North West	195
England_NHSE	ILD	region_strat	01/08/2020	South East	240
England_NHSE	ILD	region_strat	01/08/2020	South West	120
England_NHSE	ILD	region_strat	01/08/2020	West Midlands	120
England_NHSE	ILD	region_strat	01/08/2020	Yorkshire and The Humber	125
England_NHSE	ILD	region_strat	01/09/2020	East Midlands	135
England_NHSE	ILD	region_strat	01/09/2020	East of England	145
England_NHSE	ILD	region_strat	01/09/2020	London	165
England_NHSE	ILD	region_strat	01/09/2020	North East	110
England_NHSE	ILD	region_strat	01/09/2020	North West	220
England_NHSE	ILD	region_strat	01/09/2020	South East	240
England_NHSE	ILD	region_strat	01/09/2020	South West	145
England_NHSE	ILD	region_strat	01/09/2020	West Midlands	165
England_NHSE	ILD	region_strat	01/09/2020	Yorkshire and The Humber	130
England_NHSE	ILD	region_strat	01/10/2020	East Midlands	145
England_NHSE	ILD	region_strat	01/10/2020	East of England	165
England_NHSE	ILD	region_strat	01/10/2020	London	165
England_NHSE	ILD	region_strat	01/10/2020	North East	105
England_NHSE	ILD	region_strat	01/10/2020	North West	250
England_NHSE	ILD	region_strat	01/10/2020	South East	245
England_NHSE	ILD	region_strat	01/10/2020	South West	155
England_NHSE	ILD	region_strat	01/10/2020	West Midlands	170
England_NHSE	ILD	region_strat	01/10/2020	Yorkshire and The Humber	150
England_NHSE	ILD	region_strat	01/11/2020	East Midlands	155
England_NHSE	ILD	region_strat	01/11/2020	East of England	170
England_NHSE	ILD	region_strat	01/11/2020	London	155
England_NHSE	ILD	region_strat	01/11/2020	North East	85
England_NHSE	ILD	region_strat	01/11/2020	North West	220
England_NHSE	ILD	region_strat	01/11/2020	South East	270
England_NHSE	ILD	region_strat	01/11/2020	South West	175
England_NHSE	ILD	region_strat	01/11/2020	West Midlands	160
England_NHSE	ILD	region_strat	01/11/2020	Yorkshire and The Humber	130
England_NHSE	ILD	region_strat	01/12/2020	East Midlands	155
England_NHSE	ILD	region_strat	01/12/2020	East of England	160
England_NHSE	ILD	region_strat	01/12/2020	London	160
England_NHSE	ILD	region_strat	01/12/2020	North East	80
England_NHSE	ILD	region_strat	01/12/2020	North West	225
England_NHSE	ILD	region_strat	01/12/2020	South East	225
England_NHSE	ILD	region_strat	01/12/2020	South West	125
England_NHSE	ILD	region_strat	01/12/2020	West Midlands	130
England_NHSE	ILD	region_strat	01/12/2020	Yorkshire and The Humber	135
England_NHSE	ILD	region_strat	01/01/2021	East Midlands	150
England_NHSE	ILD	region_strat	01/01/2021	East of England	160
England_NHSE	ILD	region_strat	01/01/2021	London	175
England_NHSE	ILD	region_strat	01/01/2021	North East	85
England_NHSE	ILD	region_strat	01/01/2021	North West	230
England_NHSE	ILD	region_strat	01/01/2021	South East	195
England_NHSE	ILD	region_strat	01/01/2021	South West	155
England_NHSE	ILD	region_strat	01/01/2021	West Midlands	145
England_NHSE	ILD	region_strat	01/01/2021	Yorkshire and The Humber	145
England_NHSE	ILD	region_strat	01/02/2021	East Midlands	120

England_NHSE	ILD	region_strat	01/02/2021	East of England	125
England_NHSE	ILD	region_strat	01/02/2021	London	145
England_NHSE	ILD	region_strat	01/02/2021	North East	70
England_NHSE	ILD	region_strat	01/02/2021	North West	220
England_NHSE	ILD	region_strat	01/02/2021	South East	225
England_NHSE	ILD	region_strat	01/02/2021	South West	145
England_NHSE	ILD	region_strat	01/02/2021	West Midlands	120
England_NHSE	ILD	region_strat	01/02/2021	Yorkshire and The Humber	150
England_NHSE	ILD	region_strat	01/03/2021	East Midlands	170
England_NHSE	ILD	region_strat	01/03/2021	East of England	180
England_NHSE	ILD	region_strat	01/03/2021	London	195
England_NHSE	ILD	region_strat	01/03/2021	North East	100
England_NHSE	ILD	region_strat	01/03/2021	North West	295
England_NHSE	ILD	region_strat	01/03/2021	South East	290
England_NHSE	ILD	region_strat	01/03/2021	South West	170
England_NHSE	ILD	region_strat	01/03/2021	West Midlands	155
England_NHSE	ILD	region_strat	01/03/2021	Yorkshire and The Humber	160
England_NHSE	ILD	region_strat	01/04/2021	East Midlands	155
England_NHSE	ILD	region_strat	01/04/2021	East of England	160
England_NHSE	ILD	region_strat	01/04/2021	London	215
England_NHSE	ILD	region_strat	01/04/2021	North East	90
England_NHSE	ILD	region_strat	01/04/2021	North West	265
England_NHSE	ILD	region_strat	01/04/2021	South East	265
England_NHSE	ILD	region_strat	01/04/2021	South West	150
England_NHSE	ILD	region_strat	01/04/2021	West Midlands	175
England_NHSE	ILD	region_strat	01/04/2021	Yorkshire and The Humber	140
England_NHSE	ILD	region_strat	01/05/2021	East Midlands	150
England_NHSE	ILD	region_strat	01/05/2021	East of England	180
England_NHSE	ILD	region_strat	01/05/2021	London	190
England_NHSE	ILD	region_strat	01/05/2021	North East	85
England_NHSE	ILD	region_strat	01/05/2021	North West	245
England_NHSE	ILD	region_strat	01/05/2021	South East	280
England_NHSE	ILD	region_strat	01/05/2021	South West	160
England_NHSE	ILD	region_strat	01/05/2021	West Midlands	190
England_NHSE	ILD	region_strat	01/05/2021	Yorkshire and The Humber	170
England_NHSE	ILD	region_strat	01/06/2021	East Midlands	160
England_NHSE	ILD	region_strat	01/06/2021	East of England	200
England_NHSE	ILD	region_strat	01/06/2021	London	230
England_NHSE	ILD	region_strat	01/06/2021	North East	80
England_NHSE	ILD	region_strat	01/06/2021	North West	275
England_NHSE	ILD	region_strat	01/06/2021	South East	300
England_NHSE	ILD	region_strat	01/06/2021	South West	175
England_NHSE	ILD	region_strat	01/06/2021	West Midlands	170
England_NHSE	ILD	region_strat	01/06/2021	Yorkshire and The Humber	170
England_NHSE	ILD	region_strat	01/07/2021	East Midlands	175
England_NHSE	ILD	region_strat	01/07/2021	East of England	190
England_NHSE	ILD	region_strat	01/07/2021	London	170
England_NHSE	ILD	region_strat	01/07/2021	North East	85
England_NHSE	ILD	region_strat	01/07/2021	North West	255
England_NHSE	ILD	region_strat	01/07/2021	South East	290
England_NHSE	ILD	region_strat	01/07/2021	South West	170
England_NHSE	ILD	region_strat	01/07/2021	West Midlands	185
England_NHSE	ILD	region_strat	01/07/2021	Yorkshire and The Humber	160
England_NHSE	ILD	region_strat	01/08/2021	East Midlands	140
England_NHSE	ILD	region_strat	01/08/2021	East of England	180
England_NHSE	ILD	region_strat	01/08/2021	London	180
England_NHSE	ILD	region_strat	01/08/2021	North East	95
England_NHSE	ILD	region_strat	01/08/2021	North West	240
England_NHSE	ILD	region_strat	01/08/2021	South East	285
England_NHSE	ILD	region_strat	01/08/2021	South West	170
England_NHSE	ILD	region_strat	01/08/2021	West Midlands	150
England_NHSE	ILD	region_strat	01/08/2021	Yorkshire and The Humber	155
England_NHSE	ILD	region_strat	01/09/2021	East Midlands	155

England_NHSE	ILD	region_strat	01/09/2021	East of England	170
England_NHSE	ILD	region_strat	01/09/2021	London	200
England_NHSE	ILD	region_strat	01/09/2021	North East	85
England_NHSE	ILD	region_strat	01/09/2021	North West	285
England_NHSE	ILD	region_strat	01/09/2021	South East	290
England_NHSE	ILD	region_strat	01/09/2021	South West	170
England_NHSE	ILD	region_strat	01/09/2021	West Midlands	200
England_NHSE	ILD	region_strat	01/09/2021	Yorkshire and The Humber	170
England_NHSE	ILD	region_strat	01/10/2021	East Midlands	195
England_NHSE	ILD	region_strat	01/10/2021	East of England	180
England_NHSE	ILD	region_strat	01/10/2021	London	185
England_NHSE	ILD	region_strat	01/10/2021	North East	110
England_NHSE	ILD	region_strat	01/10/2021	North West	265
England_NHSE	ILD	region_strat	01/10/2021	South East	270
England_NHSE	ILD	region_strat	01/10/2021	South West	155
England_NHSE	ILD	region_strat	01/10/2021	West Midlands	165
England_NHSE	ILD	region_strat	01/10/2021	Yorkshire and The Humber	165
England_NHSE	ILD	region_strat	01/11/2021	East Midlands	210
England_NHSE	ILD	region_strat	01/11/2021	East of England	215
England_NHSE	ILD	region_strat	01/11/2021	London	205
England_NHSE	ILD	region_strat	01/11/2021	North East	110
England_NHSE	ILD	region_strat	01/11/2021	North West	270
England_NHSE	ILD	region_strat	01/11/2021	South East	320
England_NHSE	ILD	region_strat	01/11/2021	South West	155
England_NHSE	ILD	region_strat	01/11/2021	West Midlands	210
England_NHSE	ILD	region_strat	01/11/2021	Yorkshire and The Humber	175
England_NHSE	ILD	region_strat	01/12/2021	East Midlands	200
England_NHSE	ILD	region_strat	01/12/2021	East of England	195
England_NHSE	ILD	region_strat	01/12/2021	London	190
England_NHSE	ILD	region_strat	01/12/2021	North East	95
England_NHSE	ILD	region_strat	01/12/2021	North West	295
England_NHSE	ILD	region_strat	01/12/2021	South East	275
England_NHSE	ILD	region_strat	01/12/2021	South West	145
England_NHSE	ILD	region_strat	01/12/2021	West Midlands	205
England_NHSE	ILD	region_strat	01/12/2021	Yorkshire and The Humber	180
England_NHSE	ILD	region_strat	01/01/2022	East Midlands	195
England_NHSE	ILD	region_strat	01/01/2022	East of England	180
England_NHSE	ILD	region_strat	01/01/2022	London	175
England_NHSE	ILD	region_strat	01/01/2022	North East	95
England_NHSE	ILD	region_strat	01/01/2022	North West	270
England_NHSE	ILD	region_strat	01/01/2022	South East	325
England_NHSE	ILD	region_strat	01/01/2022	South West	185
England_NHSE	ILD	region_strat	01/01/2022	West Midlands	200
England_NHSE	ILD	region_strat	01/01/2022	Yorkshire and The Humber	180
England_NHSE	ILD	region_strat	01/02/2022	East Midlands	185
England_NHSE	ILD	region_strat	01/02/2022	East of England	175
England_NHSE	ILD	region_strat	01/02/2022	London	190
England_NHSE	ILD	region_strat	01/02/2022	North East	100
England_NHSE	ILD	region_strat	01/02/2022	North West	260
England_NHSE	ILD	region_strat	01/02/2022	South East	300
England_NHSE	ILD	region_strat	01/02/2022	South West	170
England_NHSE	ILD	region_strat	01/02/2022	West Midlands	195
England_NHSE	ILD	region_strat	01/02/2022	Yorkshire and The Humber	195
England_NHSE	ILD	region_strat	01/03/2022	East Midlands	195
England_NHSE	ILD	region_strat	01/03/2022	East of England	235
England_NHSE	ILD	region_strat	01/03/2022	London	215
England_NHSE	ILD	region_strat	01/03/2022	North East	125
England_NHSE	ILD	region_strat	01/03/2022	North West	290
England_NHSE	ILD	region_strat	01/03/2022	South East	370
England_NHSE	ILD	region_strat	01/03/2022	South West	185
England_NHSE	ILD	region_strat	01/03/2022	West Midlands	245
England_NHSE	ILD	region_strat	01/03/2022	Yorkshire and The Humber	185
England_NHSE	ILD	region_strat	01/04/2022	East Midlands	175

England_NHSE	ILD	region_strat	01/04/2022	East of England	200
England_NHSE	ILD	region_strat	01/04/2022	London	170
England_NHSE	ILD	region_strat	01/04/2022	North East	90
England_NHSE	ILD	region_strat	01/04/2022	North West	255
England_NHSE	ILD	region_strat	01/04/2022	South East	275
England_NHSE	ILD	region_strat	01/04/2022	South West	155
England_NHSE	ILD	region_strat	01/04/2022	West Midlands	195
England_NHSE	ILD	region_strat	01/04/2022	Yorkshire and The Humber	160
England_NHSE	ILD	region_strat	01/05/2022	East Midlands	200
England_NHSE	ILD	region_strat	01/05/2022	East of England	215
England_NHSE	ILD	region_strat	01/05/2022	London	205
England_NHSE	ILD	region_strat	01/05/2022	North East	115
England_NHSE	ILD	region_strat	01/05/2022	North West	315
England_NHSE	ILD	region_strat	01/05/2022	South East	315
England_NHSE	ILD	region_strat	01/05/2022	South West	200
England_NHSE	ILD	region_strat	01/05/2022	West Midlands	255
England_NHSE	ILD	region_strat	01/05/2022	Yorkshire and The Humber	210
England_NHSE	ILD	region_strat	01/06/2022	East Midlands	165
England_NHSE	ILD	region_strat	01/06/2022	East of England	205
England_NHSE	ILD	region_strat	01/06/2022	London	175
England_NHSE	ILD	region_strat	01/06/2022	North East	105
England_NHSE	ILD	region_strat	01/06/2022	North West	295
England_NHSE	ILD	region_strat	01/06/2022	South East	270
England_NHSE	ILD	region_strat	01/06/2022	South West	175
England_NHSE	ILD	region_strat	01/06/2022	West Midlands	205
England_NHSE	ILD	region_strat	01/06/2022	Yorkshire and The Humber	195
England_NHSE	ILD	region_strat	01/07/2022	East Midlands	150
England_NHSE	ILD	region_strat	01/07/2022	East of England	175
England_NHSE	ILD	region_strat	01/07/2022	London	170
England_NHSE	ILD	region_strat	01/07/2022	North East	90
England_NHSE	ILD	region_strat	01/07/2022	North West	255
England_NHSE	ILD	region_strat	01/07/2022	South East	285
England_NHSE	ILD	region_strat	01/07/2022	South West	170
England_NHSE	ILD	region_strat	01/07/2022	West Midlands	175
England_NHSE	ILD	region_strat	01/07/2022	Yorkshire and The Humber	190
England_NHSE	ILD	region_strat	01/08/2022	East Midlands	185
England_NHSE	ILD	region_strat	01/08/2022	East of England	195
England_NHSE	ILD	region_strat	01/08/2022	London	190
England_NHSE	ILD	region_strat	01/08/2022	North East	95
England_NHSE	ILD	region_strat	01/08/2022	North West	270
England_NHSE	ILD	region_strat	01/08/2022	South East	325
England_NHSE	ILD	region_strat	01/08/2022	South West	170
England_NHSE	ILD	region_strat	01/08/2022	West Midlands	175
England_NHSE	ILD	region_strat	01/08/2022	Yorkshire and The Humber	175
England_NHSE	ILD	region_strat	01/09/2022	East Midlands	195
England_NHSE	ILD	region_strat	01/09/2022	East of England	195
England_NHSE	ILD	region_strat	01/09/2022	London	195
England_NHSE	ILD	region_strat	01/09/2022	North East	85
England_NHSE	ILD	region_strat	01/09/2022	North West	260
England_NHSE	ILD	region_strat	01/09/2022	South East	330
England_NHSE	ILD	region_strat	01/09/2022	South West	170
England_NHSE	ILD	region_strat	01/09/2022	West Midlands	195
England_NHSE	ILD	region_strat	01/09/2022	Yorkshire and The Humber	170
England_NHSE	ILD	region_strat	01/10/2022	East Midlands	180
England_NHSE	ILD	region_strat	01/10/2022	East of England	200
England_NHSE	ILD	region_strat	01/10/2022	London	180
England_NHSE	ILD	region_strat	01/10/2022	North East	90
England_NHSE	ILD	region_strat	01/10/2022	North West	270
England_NHSE	ILD	region_strat	01/10/2022	South East	320
England_NHSE	ILD	region_strat	01/10/2022	South West	175
England_NHSE	ILD	region_strat	01/10/2022	West Midlands	180
England_NHSE	ILD	region_strat	01/10/2022	Yorkshire and The Humber	155
England_NHSE	ILD	region_strat	01/11/2022	East Midlands	195

England_NHSE	ILD	region_strat	01/11/2022	East of England	225
England_NHSE	ILD	region_strat	01/11/2022	London	195
England_NHSE	ILD	region_strat	01/11/2022	North East	110
England_NHSE	ILD	region_strat	01/11/2022	North West	300
England_NHSE	ILD	region_strat	01/11/2022	South East	375
England_NHSE	ILD	region_strat	01/11/2022	South West	190
England_NHSE	ILD	region_strat	01/11/2022	West Midlands	210
England_NHSE	ILD	region_strat	01/11/2022	Yorkshire and The Humber	195
England_NHSE	ILD	region_strat	01/12/2022	East Midlands	150
England_NHSE	ILD	region_strat	01/12/2022	East of England	175
England_NHSE	ILD	region_strat	01/12/2022	London	165
England_NHSE	ILD	region_strat	01/12/2022	North East	80
England_NHSE	ILD	region_strat	01/12/2022	North West	270
England_NHSE	ILD	region_strat	01/12/2022	South East	270
England_NHSE	ILD	region_strat	01/12/2022	South West	170
England_NHSE	ILD	region_strat	01/12/2022	West Midlands	170
England_NHSE	ILD	region_strat	01/12/2022	Yorkshire and The Humber	165
England_NHSE	ILD	region_strat	01/01/2023	East Midlands	185
England_NHSE	ILD	region_strat	01/01/2023	East of England	180
England_NHSE	ILD	region_strat	01/01/2023	London	205
England_NHSE	ILD	region_strat	01/01/2023	North East	105
England_NHSE	ILD	region_strat	01/01/2023	North West	325
England_NHSE	ILD	region_strat	01/01/2023	South East	315
England_NHSE	ILD	region_strat	01/01/2023	South West	195
England_NHSE	ILD	region_strat	01/01/2023	West Midlands	210
England_NHSE	ILD	region_strat	01/01/2023	Yorkshire and The Humber	215
England_NHSE	ILD	region_strat	01/02/2023	East Midlands	185
England_NHSE	ILD	region_strat	01/02/2023	East of England	195
England_NHSE	ILD	region_strat	01/02/2023	London	195
England_NHSE	ILD	region_strat	01/02/2023	North East	95
England_NHSE	ILD	region_strat	01/02/2023	North West	260
England_NHSE	ILD	region_strat	01/02/2023	South East	320
England_NHSE	ILD	region_strat	01/02/2023	South West	160
England_NHSE	ILD	region_strat	01/02/2023	West Midlands	200
England_NHSE	ILD	region_strat	01/02/2023	Yorkshire and The Humber	185
England_NHSE	ILD	region_strat	01/03/2023	East Midlands	195
England_NHSE	ILD	region_strat	01/03/2023	East of England	215
England_NHSE	ILD	region_strat	01/03/2023	London	220
England_NHSE	ILD	region_strat	01/03/2023	North East	115
England_NHSE	ILD	region_strat	01/03/2023	North West	330
England_NHSE	ILD	region_strat	01/03/2023	South East	310
England_NHSE	ILD	region_strat	01/03/2023	South West	235
England_NHSE	ILD	region_strat	01/03/2023	West Midlands	210
England_NHSE	ILD	region_strat	01/03/2023	Yorkshire and The Humber	200
England_NHSE	ILD	region_strat	01/04/2023	East Midlands	150
England_NHSE	ILD	region_strat	01/04/2023	East of England	170
England_NHSE	ILD	region_strat	01/04/2023	London	185
England_NHSE	ILD	region_strat	01/04/2023	North East	80
England_NHSE	ILD	region_strat	01/04/2023	North West	230
England_NHSE	ILD	region_strat	01/04/2023	South East	270
England_NHSE	ILD	region_strat	01/04/2023	South West	170
England_NHSE	ILD	region_strat	01/04/2023	West Midlands	165
England_NHSE	ILD	region_strat	01/04/2023	Yorkshire and The Humber	155
England_NHSE	ILD	region_strat	01/05/2023	East Midlands	215
England_NHSE	ILD	region_strat	01/05/2023	East of England	215
England_NHSE	ILD	region_strat	01/05/2023	London	180
England_NHSE	ILD	region_strat	01/05/2023	North East	90
England_NHSE	ILD	region_strat	01/05/2023	North West	275
England_NHSE	ILD	region_strat	01/05/2023	South East	335
England_NHSE	ILD	region_strat	01/05/2023	South West	195
England_NHSE	ILD	region_strat	01/05/2023	West Midlands	195
England_NHSE	ILD	region_strat	01/05/2023	Yorkshire and The Humber	190
England_NHSE	ILD	region_strat	01/06/2023	East Midlands	195

England_NHSE	ILD	region_strat	01/06/2023	East of England	175
England_NHSE	ILD	region_strat	01/06/2023	London	170
England_NHSE	ILD	region_strat	01/06/2023	North East	90
England_NHSE	ILD	region_strat	01/06/2023	North West	265
England_NHSE	ILD	region_strat	01/06/2023	South East	280
England_NHSE	ILD	region_strat	01/06/2023	South West	170
England_NHSE	ILD	region_strat	01/06/2023	West Midlands	220
England_NHSE	ILD	region_strat	01/06/2023	Yorkshire and The Humber	175
Scotland	Asthma	all	01/01/2004		3601
Scotland	Asthma	all	01/01/2005		3206
Scotland	Asthma	all	01/01/2006		2618
Scotland	Asthma	all	01/01/2007		2500
Scotland	Asthma	all	01/01/2008		2673
Scotland	Asthma	all	01/01/2009		2671
Scotland	Asthma	all	01/01/2010		2420
Scotland	Asthma	all	01/01/2011		2376
Scotland	Asthma	all	01/01/2012		2564
Scotland	Asthma	all	01/01/2013		2408
Scotland	Asthma	all	01/01/2014		2516
Scotland	Asthma	all	01/01/2015		2298
Scotland	Asthma	all	01/01/2016		2276
Scotland	Asthma	all	01/01/2017		2357
Scotland	Asthma	all	01/01/2018		2432
Scotland	Asthma	all	01/01/2019		2569
Scotland	Asthma	all	01/01/2020		625
Scotland	Asthma	all	01/04/2020		290
Scotland	Asthma	all	01/07/2020		422
Scotland	Asthma	all	01/10/2020		503
Scotland	Asthma	all	01/01/2021		444
Scotland	Asthma	all	01/04/2021		507
Scotland	Asthma	all	01/07/2021		507
Scotland	Asthma	all	01/10/2021		639
Scotland	Asthma	all	01/01/2022		577
Scotland	Asthma	all	01/04/2022		637
Scotland	Asthma	all	01/07/2022		634
Scotland	Asthma	all	01/10/2022		724
Scotland	Asthma	all	01/01/2023		751
Scotland	COPD	all	01/01/2004		1643
Scotland	COPD	all	01/01/2005		1544
Scotland	COPD	all	01/01/2006		1559
Scotland	COPD	all	01/01/2007		1460
Scotland	COPD	all	01/01/2008		1476
Scotland	COPD	all	01/01/2009		1408
Scotland	COPD	all	01/01/2010		1704
Scotland	COPD	all	01/01/2011		1604
Scotland	COPD	all	01/01/2012		1960
Scotland	COPD	all	01/01/2013		1719
Scotland	COPD	all	01/01/2014		1725
Scotland	COPD	all	01/01/2015		1911
Scotland	COPD	all	01/01/2016		1896
Scotland	COPD	all	01/01/2017		1885
Scotland	COPD	all	01/01/2018		1912
Scotland	COPD	all	01/01/2019		1941
Scotland	COPD	all	01/01/2020		447
Scotland	COPD	all	01/04/2020		194
Scotland	COPD	all	01/07/2020		329
Scotland	COPD	all	01/10/2020		282
Scotland	COPD	all	01/01/2021		243
Scotland	COPD	all	01/04/2021		325
Scotland	COPD	all	01/07/2021		367
Scotland	COPD	all	01/10/2021		371
Scotland	COPD	all	01/01/2022		392
Scotland	COPD	all	01/04/2022		389

Scotland	COPD	all	01/07/2022		381
Scotland	COPD	all	01/10/2022		414
Scotland	COPD	all	01/01/2023		440
Scotland	ILD	all	01/01/2004		156
Scotland	ILD	all	01/01/2005		175
Scotland	ILD	all	01/01/2006		193
Scotland	ILD	all	01/01/2007		198
Scotland	ILD	all	01/01/2008		209
Scotland	ILD	all	01/01/2009		207
Scotland	ILD	all	01/01/2010		265
Scotland	ILD	all	01/01/2011		220
Scotland	ILD	all	01/01/2012		251
Scotland	ILD	all	01/01/2013		247
Scotland	ILD	all	01/01/2014		235
Scotland	ILD	all	01/01/2015		273
Scotland	ILD	all	01/01/2016		242
Scotland	ILD	all	01/01/2017		306
Scotland	ILD	all	01/01/2018		307
Scotland	ILD	all	01/01/2019		349
Scotland	ILD	all	01/01/2020		75
Scotland	ILD	all	01/04/2020		57
Scotland	ILD	all	01/07/2020		65
Scotland	ILD	all	01/10/2020		72
Scotland	ILD	all	01/01/2021		70
Scotland	ILD	all	01/04/2021		85
Scotland	ILD	all	01/07/2021		67
Scotland	ILD	all	01/10/2021		78
Scotland	ILD	all	01/01/2022		91
Scotland	ILD	all	01/04/2022		98
Scotland	ILD	all	01/07/2022		84
Scotland	ILD	all	01/10/2022		79
Scotland	ILD	all	01/01/2023		69
Scotland	Asthma	sex_strat_adults	01/01/2004	Female	1438
Scotland	Asthma	sex_strat_adults	01/01/2005	Female	1268
Scotland	Asthma	sex_strat_adults	01/01/2006	Female	984
Scotland	Asthma	sex_strat_adults	01/01/2007	Female	909
Scotland	Asthma	sex_strat_adults	01/01/2008	Female	1031
Scotland	Asthma	sex_strat_adults	01/01/2009	Female	1006
Scotland	Asthma	sex_strat_adults	01/01/2010	Female	916
Scotland	Asthma	sex_strat_adults	01/01/2011	Female	813
Scotland	Asthma	sex_strat_adults	01/01/2012	Female	899
Scotland	Asthma	sex_strat_adults	01/01/2013	Female	886
Scotland	Asthma	sex_strat_adults	01/01/2014	Female	908
Scotland	Asthma	sex_strat_adults	01/01/2015	Female	842
Scotland	Asthma	sex_strat_adults	01/01/2016	Female	834
Scotland	Asthma	sex_strat_adults	01/01/2017	Female	905
Scotland	Asthma	sex_strat_adults	01/01/2018	Female	973
Scotland	Asthma	sex_strat_adults	01/01/2019	Female	1039
Scotland	Asthma	sex_strat_adults	01/01/2020	Female	268
Scotland	Asthma	sex_strat_adults	01/04/2020	Female	122
Scotland	Asthma	sex_strat_adults	01/07/2020	Female	183
Scotland	Asthma	sex_strat_adults	01/10/2020	Female	197
Scotland	Asthma	sex_strat_adults	01/01/2021	Female	202
Scotland	Asthma	sex_strat_adults	01/04/2021	Female	210
Scotland	Asthma	sex_strat_adults	01/07/2021	Female	201
Scotland	Asthma	sex_strat_adults	01/10/2021	Female	234
Scotland	Asthma	sex_strat_adults	01/01/2022	Female	225
Scotland	Asthma	sex_strat_adults	01/04/2022	Female	233
Scotland	Asthma	sex_strat_adults	01/07/2022	Female	253
Scotland	Asthma	sex_strat_adults	01/10/2022	Female	274
Scotland	Asthma	sex_strat_adults	01/01/2023	Female	292
Scotland	Asthma	sex_strat_children	01/01/2004	Female	487
Scotland	Asthma	sex_strat_children	01/01/2005	Female	476

Scotland	Asthma	sex_strat_children	01/01/2006	Female	426
Scotland	Asthma	sex_strat_children	01/01/2007	Female	429
Scotland	Asthma	sex_strat_children	01/01/2008	Female	414
Scotland	Asthma	sex_strat_children	01/01/2009	Female	423
Scotland	Asthma	sex_strat_children	01/01/2010	Female	399
Scotland	Asthma	sex_strat_children	01/01/2011	Female	426
Scotland	Asthma	sex_strat_children	01/01/2012	Female	450
Scotland	Asthma	sex_strat_children	01/01/2013	Female	402
Scotland	Asthma	sex_strat_children	01/01/2014	Female	436
Scotland	Asthma	sex_strat_children	01/01/2015	Female	394
Scotland	Asthma	sex_strat_children	01/01/2016	Female	396
Scotland	Asthma	sex_strat_children	01/01/2017	Female	427
Scotland	Asthma	sex_strat_children	01/01/2018	Female	367
Scotland	Asthma	sex_strat_children	01/01/2019	Female	399
Scotland	Asthma	sex_strat_children	01/01/2020	Female	114
Scotland	Asthma	sex_strat_children	01/04/2020	Female	31
Scotland	Asthma	sex_strat_children	01/07/2020	Female	51
Scotland	Asthma	sex_strat_children	01/10/2020	Female	85
Scotland	Asthma	sex_strat_children	01/01/2021	Female	53
Scotland	Asthma	sex_strat_children	01/04/2021	Female	76
Scotland	Asthma	sex_strat_children	01/07/2021	Female	62
Scotland	Asthma	sex_strat_children	01/10/2021	Female	108
Scotland	Asthma	sex_strat_children	01/01/2022	Female	100
Scotland	Asthma	sex_strat_children	01/04/2022	Female	114
Scotland	Asthma	sex_strat_children	01/07/2022	Female	92
Scotland	Asthma	sex_strat_children	01/10/2022	Female	112
Scotland	Asthma	sex_strat_children	01/01/2023	Female	119
Scotland	Asthma	sex_strat_adults	01/01/2004	Male	1013
Scotland	Asthma	sex_strat_adults	01/01/2005	Male	848
Scotland	Asthma	sex_strat_adults	01/01/2006	Male	666
Scotland	Asthma	sex_strat_adults	01/01/2007	Male	659
Scotland	Asthma	sex_strat_adults	01/01/2008	Male	746
Scotland	Asthma	sex_strat_adults	01/01/2009	Male	721
Scotland	Asthma	sex_strat_adults	01/01/2010	Male	655
Scotland	Asthma	sex_strat_adults	01/01/2011	Male	640
Scotland	Asthma	sex_strat_adults	01/01/2012	Male	706
Scotland	Asthma	sex_strat_adults	01/01/2013	Male	660
Scotland	Asthma	sex_strat_adults	01/01/2014	Male	633
Scotland	Asthma	sex_strat_adults	01/01/2015	Male	536
Scotland	Asthma	sex_strat_adults	01/01/2016	Male	565
Scotland	Asthma	sex_strat_adults	01/01/2017	Male	581
Scotland	Asthma	sex_strat_adults	01/01/2018	Male	656
Scotland	Asthma	sex_strat_adults	01/01/2019	Male	728
Scotland	Asthma	sex_strat_adults	01/01/2020	Male	146
Scotland	Asthma	sex_strat_adults	01/04/2020	Male	83
Scotland	Asthma	sex_strat_adults	01/07/2020	Male	131
Scotland	Asthma	sex_strat_adults	01/10/2020	Male	124
Scotland	Asthma	sex_strat_adults	01/01/2021	Male	139
Scotland	Asthma	sex_strat_adults	01/04/2021	Male	145
Scotland	Asthma	sex_strat_adults	01/07/2021	Male	148
Scotland	Asthma	sex_strat_adults	01/10/2021	Male	169
Scotland	Asthma	sex_strat_adults	01/01/2022	Male	161
Scotland	Asthma	sex_strat_adults	01/04/2022	Male	177
Scotland	Asthma	sex_strat_adults	01/07/2022	Male	183
Scotland	Asthma	sex_strat_adults	01/10/2022	Male	187
Scotland	Asthma	sex_strat_adults	01/01/2023	Male	200
Scotland	Asthma	sex_strat_children	01/01/2004	Male	663
Scotland	Asthma	sex_strat_children	01/01/2005	Male	614
Scotland	Asthma	sex_strat_children	01/01/2006	Male	542
Scotland	Asthma	sex_strat_children	01/01/2007	Male	503
Scotland	Asthma	sex_strat_children	01/01/2008	Male	482
Scotland	Asthma	sex_strat_children	01/01/2009	Male	521
Scotland	Asthma	sex_strat_children	01/01/2010	Male	450

Scotland	Asthma	sex_strat_children	01/01/2011	Male		497
Scotland	Asthma	sex_strat_children	01/01/2012	Male		509
Scotland	Asthma	sex_strat_children	01/01/2013	Male		460
Scotland	Asthma	sex_strat_children	01/01/2014	Male		539
Scotland	Asthma	sex_strat_children	01/01/2015	Male		526
Scotland	Asthma	sex_strat_children	01/01/2016	Male		481
Scotland	Asthma	sex_strat_children	01/01/2017	Male		444
Scotland	Asthma	sex_strat_children	01/01/2018	Male		436
Scotland	Asthma	sex_strat_children	01/01/2019	Male		403
Scotland	Asthma	sex_strat_children	01/01/2020	Male		97
Scotland	Asthma	sex_strat_children	01/04/2020	Male		54
Scotland	Asthma	sex_strat_children	01/07/2020	Male		57
Scotland	Asthma	sex_strat_children	01/10/2020	Male		97
Scotland	Asthma	sex_strat_children	01/01/2021	Male		50
Scotland	Asthma	sex_strat_children	01/04/2021	Male		76
Scotland	Asthma	sex_strat_children	01/07/2021	Male		96
Scotland	Asthma	sex_strat_children	01/10/2021	Male		128
Scotland	Asthma	sex_strat_children	01/01/2022	Male		91
Scotland	Asthma	sex_strat_children	01/04/2022	Male		113
Scotland	Asthma	sex_strat_children	01/07/2022	Male		106
Scotland	Asthma	sex_strat_children	01/10/2022	Male		151
Scotland	Asthma	sex_strat_children	01/01/2023	Male		140
Scotland	COPD	age_strat	01/01/2004		40 to 49	110
Scotland	COPD	age_strat	01/01/2005		40 to 49	138
Scotland	COPD	age_strat	01/01/2006		40 to 49	110
Scotland	COPD	age_strat	01/01/2007		40 to 49	140
Scotland	COPD	age_strat	01/01/2008		40 to 49	129
Scotland	COPD	age_strat	01/01/2009		40 to 49	124
Scotland	COPD	age_strat	01/01/2010		40 to 49	166
Scotland	COPD	age_strat	01/01/2011		40 to 49	172
Scotland	COPD	age_strat	01/01/2012		40 to 49	179
Scotland	COPD	age_strat	01/01/2013		40 to 49	173
Scotland	COPD	age_strat	01/01/2014		40 to 49	171
Scotland	COPD	age_strat	01/01/2015		40 to 49	189
Scotland	COPD	age_strat	01/01/2016		40 to 49	186
Scotland	COPD	age_strat	01/01/2017		40 to 49	192
Scotland	COPD	age_strat	01/01/2018		40 to 49	177
Scotland	COPD	age_strat	01/01/2019		40 to 49	191
Scotland	COPD	age_strat	01/01/2020		40 to 49	47
Scotland	COPD	age_strat	01/01/2020		40 to 49	135
Scotland	COPD	age_strat	01/04/2020		40 to 49	18
Scotland	COPD	age_strat	01/07/2020		40 to 49	37
Scotland	COPD	age_strat	01/10/2020		40 to 49	38
Scotland	COPD	age_strat	01/01/2021		40 to 49	27
Scotland	COPD	age_strat	01/01/2021		40 to 49	138
Scotland	COPD	age_strat	01/04/2021		40 to 49	32
Scotland	COPD	age_strat	01/07/2021		40 to 49	33
Scotland	COPD	age_strat	01/10/2021		40 to 49	46
Scotland	COPD	age_strat	01/01/2022		40 to 49	38
Scotland	COPD	age_strat	01/01/2022		40 to 49	133
Scotland	COPD	age_strat	01/04/2022		40 to 49	37
Scotland	COPD	age_strat	01/07/2022		40 to 49	28
Scotland	COPD	age_strat	01/10/2022		40 to 49	38
Scotland	COPD	age_strat	01/01/2023		40 to 49	40
Scotland	COPD	age_strat	01/01/2004		50 to 59	302
Scotland	COPD	age_strat	01/01/2005		50 to 59	326
Scotland	COPD	age_strat	01/01/2006		50 to 59	331
Scotland	COPD	age_strat	01/01/2007		50 to 59	298
Scotland	COPD	age_strat	01/01/2008		50 to 59	315
Scotland	COPD	age_strat	01/01/2009		50 to 59	295
Scotland	COPD	age_strat	01/01/2010		50 to 59	379
Scotland	COPD	age_strat	01/01/2011		50 to 59	321
Scotland	COPD	age_strat	01/01/2012		50 to 59	455

Scotland	COPD	age_strat	01/01/2013	50 to 59	389
Scotland	COPD	age_strat	01/01/2014	50 to 59	388
Scotland	COPD	age_strat	01/01/2015	50 to 59	438
Scotland	COPD	age_strat	01/01/2016	50 to 59	436
Scotland	COPD	age_strat	01/01/2017	50 to 59	451
Scotland	COPD	age_strat	01/01/2018	50 to 59	447
Scotland	COPD	age_strat	01/01/2019	50 to 59	434
Scotland	COPD	age_strat	01/01/2020	50 to 59	98
Scotland	COPD	age_strat	01/01/2020	50 to 59	298
Scotland	COPD	age_strat	01/04/2020	50 to 59	54
Scotland	COPD	age_strat	01/07/2020	50 to 59	76
Scotland	COPD	age_strat	01/10/2020	50 to 59	69
Scotland	COPD	age_strat	01/01/2021	50 to 59	65
Scotland	COPD	age_strat	01/01/2021	50 to 59	332
Scotland	COPD	age_strat	01/04/2021	50 to 59	82
Scotland	COPD	age_strat	01/07/2021	50 to 59	105
Scotland	COPD	age_strat	01/10/2021	50 to 59	84
Scotland	COPD	age_strat	01/01/2022	50 to 59	104
Scotland	COPD	age_strat	01/01/2022	50 to 59	379
Scotland	COPD	age_strat	01/04/2022	50 to 59	97
Scotland	COPD	age_strat	01/07/2022	50 to 59	88
Scotland	COPD	age_strat	01/10/2022	50 to 59	100
Scotland	COPD	age_strat	01/01/2023	50 to 59	99
Scotland	COPD	age_strat	01/01/2004	60 to 69	508
Scotland	COPD	age_strat	01/01/2005	60 to 69	467
Scotland	COPD	age_strat	01/01/2006	60 to 69	496
Scotland	COPD	age_strat	01/01/2007	60 to 69	443
Scotland	COPD	age_strat	01/01/2008	60 to 69	474
Scotland	COPD	age_strat	01/01/2009	60 to 69	460
Scotland	COPD	age_strat	01/01/2010	60 to 69	617
Scotland	COPD	age_strat	01/01/2011	60 to 69	504
Scotland	COPD	age_strat	01/01/2012	60 to 69	646
Scotland	COPD	age_strat	01/01/2013	60 to 69	511
Scotland	COPD	age_strat	01/01/2014	60 to 69	505
Scotland	COPD	age_strat	01/01/2015	60 to 69	581
Scotland	COPD	age_strat	01/01/2016	60 to 69	552
Scotland	COPD	age_strat	01/01/2017	60 to 69	535
Scotland	COPD	age_strat	01/01/2018	60 to 69	551
Scotland	COPD	age_strat	01/01/2019	60 to 69	578
Scotland	COPD	age_strat	01/01/2020	60 to 69	124
Scotland	COPD	age_strat	01/01/2020	60 to 69	332
Scotland	COPD	age_strat	01/04/2020	60 to 69	56
Scotland	COPD	age_strat	01/07/2020	60 to 69	87
Scotland	COPD	age_strat	01/10/2020	60 to 69	67
Scotland	COPD	age_strat	01/01/2021	60 to 69	73
Scotland	COPD	age_strat	01/01/2021	60 to 69	346
Scotland	COPD	age_strat	01/04/2021	60 to 69	85
Scotland	COPD	age_strat	01/07/2021	60 to 69	89
Scotland	COPD	age_strat	01/10/2021	60 to 69	100
Scotland	COPD	age_strat	01/01/2022	60 to 69	112
Scotland	COPD	age_strat	01/01/2022	60 to 69	467
Scotland	COPD	age_strat	01/04/2022	60 to 69	113
Scotland	COPD	age_strat	01/07/2022	60 to 69	118
Scotland	COPD	age_strat	01/10/2022	60 to 69	125
Scotland	COPD	age_strat	01/01/2023	60 to 69	128
Scotland	COPD	age_strat	01/01/2004	70plus	700
Scotland	COPD	age_strat	01/01/2005	70plus	600
Scotland	COPD	age_strat	01/01/2006	70plus	606
Scotland	COPD	age_strat	01/01/2007	70plus	553
Scotland	COPD	age_strat	01/01/2008	70plus	526
Scotland	COPD	age_strat	01/01/2009	70plus	505
Scotland	COPD	age_strat	01/01/2010	70plus	511
Scotland	COPD	age_strat	01/01/2011	70plus	583

Scotland	COPD	age_strat	01/01/2012	70plus	652
Scotland	COPD	age_strat	01/01/2013	70plus	625
Scotland	COPD	age_strat	01/01/2014	70plus	641
Scotland	COPD	age_strat	01/01/2015	70plus	685
Scotland	COPD	age_strat	01/01/2016	70plus	692
Scotland	COPD	age_strat	01/01/2017	70plus	676
Scotland	COPD	age_strat	01/01/2018	70plus	700
Scotland	COPD	age_strat	01/01/2019	70plus	695
Scotland	COPD	age_strat	01/01/2020	70plus	168
Scotland	COPD	age_strat	01/01/2020	70plus	471
Scotland	COPD	age_strat	01/04/2020	70plus	61
Scotland	COPD	age_strat	01/07/2020	70plus	124
Scotland	COPD	age_strat	01/10/2020	70plus	102
Scotland	COPD	age_strat	01/01/2021	70plus	75
Scotland	COPD	age_strat	01/01/2021	70plus	480
Scotland	COPD	age_strat	01/04/2021	70plus	121
Scotland	COPD	age_strat	01/07/2021	70plus	134
Scotland	COPD	age_strat	01/10/2021	70plus	131
Scotland	COPD	age_strat	01/01/2022	70plus	130
Scotland	COPD	age_strat	01/01/2022	70plus	575
Scotland	COPD	age_strat	01/04/2022	70plus	134
Scotland	COPD	age_strat	01/07/2022	70plus	142
Scotland	COPD	age_strat	01/10/2022	70plus	144
Scotland	COPD	age_strat	01/01/2023	70plus	160
Scotland	ILD	age_strat	01/01/2004	40 to 59	30
Scotland	ILD	age_strat	01/01/2005	40 to 59	34
Scotland	ILD	age_strat	01/01/2006	40 to 59	39
Scotland	ILD	age_strat	01/01/2007	40 to 59	38
Scotland	ILD	age_strat	01/01/2008	40 to 59	38
Scotland	ILD	age_strat	01/01/2009	40 to 59	39
Scotland	ILD	age_strat	01/01/2010	40 to 59	47
Scotland	ILD	age_strat	01/01/2011	40 to 59	42
Scotland	ILD	age_strat	01/01/2012	40 to 59	54
Scotland	ILD	age_strat	01/01/2013	40 to 59	40
Scotland	ILD	age_strat	01/01/2014	40 to 59	25
Scotland	ILD	age_strat	01/01/2015	40 to 59	40
Scotland	ILD	age_strat	01/01/2016	40 to 59	32
Scotland	ILD	age_strat	01/01/2017	40 to 59	49
Scotland	ILD	age_strat	01/01/2018	40 to 59	54
Scotland	ILD	age_strat	01/01/2019	40 to 59	49
Scotland	ILD	age_strat	01/01/2020	40 to 59	12
Scotland	ILD	age_strat	01/01/2020	40 to 59	41
Scotland	ILD	age_strat	01/04/2020	40 to 59	9
Scotland	ILD	age_strat	01/07/2020	40 to 59	11
Scotland	ILD	age_strat	01/10/2020	40 to 59	10
Scotland	ILD	age_strat	01/01/2021	40 to 59	17
Scotland	ILD	age_strat	01/01/2021	40 to 59	42
Scotland	ILD	age_strat	01/04/2021	40 to 59	9
Scotland	ILD	age_strat	01/07/2021	40 to 59	10
Scotland	ILD	age_strat	01/10/2021	40 to 59	8
Scotland	ILD	age_strat	01/01/2022	40 to 59	13
Scotland	ILD	age_strat	01/01/2022	40 to 59	49
Scotland	ILD	age_strat	01/04/2022	40 to 59	17
Scotland	ILD	age_strat	01/07/2022	40 to 59	9
Scotland	ILD	age_strat	01/10/2022	40 to 59	12
Scotland	ILD	age_strat	01/01/2023	40 to 59	12
Scotland	ILD	age_strat	01/01/2004	60 to 69	46
Scotland	ILD	age_strat	01/01/2005	60 to 69	51
Scotland	ILD	age_strat	01/01/2006	60 to 69	60
Scotland	ILD	age_strat	01/01/2007	60 to 69	51
Scotland	ILD	age_strat	01/01/2008	60 to 69	48
Scotland	ILD	age_strat	01/01/2009	60 to 69	50
Scotland	ILD	age_strat	01/01/2010	60 to 69	69

Scotland	ILD	age_strat	01/01/2011	60 to 69	50
Scotland	ILD	age_strat	01/01/2012	60 to 69	50
Scotland	ILD	age_strat	01/01/2013	60 to 69	66
Scotland	ILD	age_strat	01/01/2014	60 to 69	50
Scotland	ILD	age_strat	01/01/2015	60 to 69	58
Scotland	ILD	age_strat	01/01/2016	60 to 69	51
Scotland	ILD	age_strat	01/01/2017	60 to 69	73
Scotland	ILD	age_strat	01/01/2018	60 to 69	65
Scotland	ILD	age_strat	01/01/2019	60 to 69	74
Scotland	ILD	age_strat	01/01/2020	60 to 69	9
Scotland	ILD	age_strat	01/01/2020	60 to 69	46
Scotland	ILD	age_strat	01/04/2020	60 to 69	13
Scotland	ILD	age_strat	01/07/2020	60 to 69	9
Scotland	ILD	age_strat	01/10/2020	60 to 69	16
Scotland	ILD	age_strat	01/01/2021	60 to 69	12
Scotland	ILD	age_strat	01/01/2021	60 to 69	55
Scotland	ILD	age_strat	01/04/2021	60 to 69	14
Scotland	ILD	age_strat	01/07/2021	60 to 69	16
Scotland	ILD	age_strat	01/10/2021	60 to 69	13
Scotland	ILD	age_strat	01/01/2022	60 to 69	25
Scotland	ILD	age_strat	01/01/2022	60 to 69	69
Scotland	ILD	age_strat	01/04/2022	60 to 69	19
Scotland	ILD	age_strat	01/07/2022	60 to 69	11
Scotland	ILD	age_strat	01/10/2022	60 to 69	12
Scotland	ILD	age_strat	01/01/2023	60 to 69	18
Scotland	ILD	age_strat	01/01/2004	70plus	80
Scotland	ILD	age_strat	01/01/2005	70plus	90
Scotland	ILD	age_strat	01/01/2006	70plus	94
Scotland	ILD	age_strat	01/01/2007	70plus	109
Scotland	ILD	age_strat	01/01/2008	70plus	123
Scotland	ILD	age_strat	01/01/2009	70plus	118
Scotland	ILD	age_strat	01/01/2010	70plus	149
Scotland	ILD	age_strat	01/01/2011	70plus	128
Scotland	ILD	age_strat	01/01/2012	70plus	147
Scotland	ILD	age_strat	01/01/2013	70plus	141
Scotland	ILD	age_strat	01/01/2014	70plus	160
Scotland	ILD	age_strat	01/01/2015	70plus	175
Scotland	ILD	age_strat	01/01/2016	70plus	159
Scotland	ILD	age_strat	01/01/2017	70plus	184
Scotland	ILD	age_strat	01/01/2018	70plus	188
Scotland	ILD	age_strat	01/01/2019	70plus	226
Scotland	ILD	age_strat	01/01/2020	70plus	54
Scotland	ILD	age_strat	01/01/2020	70plus	185
Scotland	ILD	age_strat	01/04/2020	70plus	35
Scotland	ILD	age_strat	01/07/2020	70plus	45
Scotland	ILD	age_strat	01/10/2020	70plus	46
Scotland	ILD	age_strat	01/01/2021	70plus	41
Scotland	ILD	age_strat	01/01/2021	70plus	205
Scotland	ILD	age_strat	01/04/2021	70plus	62
Scotland	ILD	age_strat	01/07/2021	70plus	41
Scotland	ILD	age_strat	01/10/2021	70plus	57
Scotland	ILD	age_strat	01/01/2022	70plus	53
Scotland	ILD	age_strat	01/01/2022	70plus	238
Scotland	ILD	age_strat	01/04/2022	70plus	62
Scotland	ILD	age_strat	01/07/2022	70plus	64
Scotland	ILD	age_strat	01/10/2022	70plus	55
Scotland	ILD	age_strat	01/01/2023	70plus	39
Scotland	Asthma	age_strat	01/01/2004	0 to 4	394
Scotland	Asthma	age_strat	01/01/2004	10 to 14	259
Scotland	Asthma	age_strat	01/01/2004	15 to 19	133
Scotland	Asthma	age_strat	01/01/2004	20 to 29	321
Scotland	Asthma	age_strat	01/01/2004	30 to 39	428
Scotland	Asthma	age_strat	01/01/2004	40 to 49	411

Scotland	Asthma	age_strat	01/01/2004	5 to 9	364
Scotland	Asthma	age_strat	01/01/2004	50 to 59	412
Scotland	Asthma	age_strat	01/01/2004	60 to 69	396
Scotland	Asthma	age_strat	01/01/2004	70plus	483
Scotland	Asthma	age_strat	01/01/2005	0 to 4	374
Scotland	Asthma	age_strat	01/01/2005	10 to 14	267
Scotland	Asthma	age_strat	01/01/2005	15 to 19	122
Scotland	Asthma	age_strat	01/01/2005	20 to 29	306
Scotland	Asthma	age_strat	01/01/2005	30 to 39	374
Scotland	Asthma	age_strat	01/01/2005	40 to 49	367
Scotland	Asthma	age_strat	01/01/2005	5 to 9	327
Scotland	Asthma	age_strat	01/01/2005	50 to 59	314
Scotland	Asthma	age_strat	01/01/2005	60 to 69	345
Scotland	Asthma	age_strat	01/01/2005	70plus	410
Scotland	Asthma	age_strat	01/01/2006	0 to 4	382
Scotland	Asthma	age_strat	01/01/2006	10 to 14	190
Scotland	Asthma	age_strat	01/01/2006	15 to 19	105
Scotland	Asthma	age_strat	01/01/2006	20 to 29	230
Scotland	Asthma	age_strat	01/01/2006	30 to 39	338
Scotland	Asthma	age_strat	01/01/2006	40 to 49	308
Scotland	Asthma	age_strat	01/01/2006	5 to 9	291
Scotland	Asthma	age_strat	01/01/2006	50 to 59	267
Scotland	Asthma	age_strat	01/01/2006	60 to 69	253
Scotland	Asthma	age_strat	01/01/2006	70plus	254
Scotland	Asthma	age_strat	01/01/2007	0 to 4	340
Scotland	Asthma	age_strat	01/01/2007	10 to 14	228
Scotland	Asthma	age_strat	01/01/2007	15 to 19	106
Scotland	Asthma	age_strat	01/01/2007	20 to 29	229
Scotland	Asthma	age_strat	01/01/2007	30 to 39	293
Scotland	Asthma	age_strat	01/01/2007	40 to 49	285
Scotland	Asthma	age_strat	01/01/2007	5 to 9	258
Scotland	Asthma	age_strat	01/01/2007	50 to 59	273
Scotland	Asthma	age_strat	01/01/2007	60 to 69	235
Scotland	Asthma	age_strat	01/01/2007	70plus	253
Scotland	Asthma	age_strat	01/01/2008	0 to 4	323
Scotland	Asthma	age_strat	01/01/2008	10 to 14	194
Scotland	Asthma	age_strat	01/01/2008	15 to 19	103
Scotland	Asthma	age_strat	01/01/2008	20 to 29	245
Scotland	Asthma	age_strat	01/01/2008	30 to 39	313
Scotland	Asthma	age_strat	01/01/2008	40 to 49	285
Scotland	Asthma	age_strat	01/01/2008	5 to 9	276
Scotland	Asthma	age_strat	01/01/2008	50 to 59	291
Scotland	Asthma	age_strat	01/01/2008	60 to 69	318
Scotland	Asthma	age_strat	01/01/2008	70plus	325
Scotland	Asthma	age_strat	01/01/2009	0 to 4	344
Scotland	Asthma	age_strat	01/01/2009	10 to 14	199
Scotland	Asthma	age_strat	01/01/2009	15 to 19	114
Scotland	Asthma	age_strat	01/01/2009	20 to 29	266
Scotland	Asthma	age_strat	01/01/2009	30 to 39	288
Scotland	Asthma	age_strat	01/01/2009	40 to 49	333
Scotland	Asthma	age_strat	01/01/2009	5 to 9	287
Scotland	Asthma	age_strat	01/01/2009	50 to 59	305
Scotland	Asthma	age_strat	01/01/2009	60 to 69	267
Scotland	Asthma	age_strat	01/01/2009	70plus	268
Scotland	Asthma	age_strat	01/01/2010	0 to 4	307
Scotland	Asthma	age_strat	01/01/2010	10 to 14	151
Scotland	Asthma	age_strat	01/01/2010	15 to 19	112
Scotland	Asthma	age_strat	01/01/2010	20 to 29	231
Scotland	Asthma	age_strat	01/01/2010	30 to 39	244
Scotland	Asthma	age_strat	01/01/2010	40 to 49	335
Scotland	Asthma	age_strat	01/01/2010	5 to 9	279
Scotland	Asthma	age_strat	01/01/2010	50 to 59	278
Scotland	Asthma	age_strat	01/01/2010	60 to 69	250

Scotland	Asthma	age_strat	01/01/2010	70plus	233
Scotland	Asthma	age_strat	01/01/2011	0 to 4	301
Scotland	Asthma	age_strat	01/01/2011	10 to 14	207
Scotland	Asthma	age_strat	01/01/2011	15 to 19	107
Scotland	Asthma	age_strat	01/01/2011	20 to 29	236
Scotland	Asthma	age_strat	01/01/2011	30 to 39	244
Scotland	Asthma	age_strat	01/01/2011	40 to 49	290
Scotland	Asthma	age_strat	01/01/2011	5 to 9	308
Scotland	Asthma	age_strat	01/01/2011	50 to 59	246
Scotland	Asthma	age_strat	01/01/2011	60 to 69	242
Scotland	Asthma	age_strat	01/01/2011	70plus	195
Scotland	Asthma	age_strat	01/01/2012	0 to 4	306
Scotland	Asthma	age_strat	01/01/2012	10 to 14	209
Scotland	Asthma	age_strat	01/01/2012	15 to 19	122
Scotland	Asthma	age_strat	01/01/2012	20 to 29	250
Scotland	Asthma	age_strat	01/01/2012	30 to 39	299
Scotland	Asthma	age_strat	01/01/2012	40 to 49	352
Scotland	Asthma	age_strat	01/01/2012	5 to 9	322
Scotland	Asthma	age_strat	01/01/2012	50 to 59	295
Scotland	Asthma	age_strat	01/01/2012	60 to 69	227
Scotland	Asthma	age_strat	01/01/2012	70plus	182
Scotland	Asthma	age_strat	01/01/2013	0 to 4	238
Scotland	Asthma	age_strat	01/01/2013	10 to 14	174
Scotland	Asthma	age_strat	01/01/2013	15 to 19	110
Scotland	Asthma	age_strat	01/01/2013	20 to 29	261
Scotland	Asthma	age_strat	01/01/2013	30 to 39	243
Scotland	Asthma	age_strat	01/01/2013	40 to 49	286
Scotland	Asthma	age_strat	01/01/2013	5 to 9	340
Scotland	Asthma	age_strat	01/01/2013	50 to 59	268
Scotland	Asthma	age_strat	01/01/2013	60 to 69	236
Scotland	Asthma	age_strat	01/01/2013	70plus	252
Scotland	Asthma	age_strat	01/01/2014	0 to 4	262
Scotland	Asthma	age_strat	01/01/2014	10 to 14	231
Scotland	Asthma	age_strat	01/01/2014	15 to 19	119
Scotland	Asthma	age_strat	01/01/2014	20 to 29	278
Scotland	Asthma	age_strat	01/01/2014	30 to 39	258
Scotland	Asthma	age_strat	01/01/2014	40 to 49	293
Scotland	Asthma	age_strat	01/01/2014	5 to 9	363
Scotland	Asthma	age_strat	01/01/2014	50 to 59	257
Scotland	Asthma	age_strat	01/01/2014	60 to 69	230
Scotland	Asthma	age_strat	01/01/2014	70plus	225
Scotland	Asthma	age_strat	01/01/2015	0 to 4	210
Scotland	Asthma	age_strat	01/01/2015	10 to 14	240
Scotland	Asthma	age_strat	01/01/2015	15 to 19	128
Scotland	Asthma	age_strat	01/01/2015	20 to 29	265
Scotland	Asthma	age_strat	01/01/2015	30 to 39	224
Scotland	Asthma	age_strat	01/01/2015	40 to 49	269
Scotland	Asthma	age_strat	01/01/2015	5 to 9	342
Scotland	Asthma	age_strat	01/01/2015	50 to 59	256
Scotland	Asthma	age_strat	01/01/2015	60 to 69	211
Scotland	Asthma	age_strat	01/01/2015	70plus	153
Scotland	Asthma	age_strat	01/01/2016	0 to 4	188
Scotland	Asthma	age_strat	01/01/2016	10 to 14	218
Scotland	Asthma	age_strat	01/01/2016	15 to 19	130
Scotland	Asthma	age_strat	01/01/2016	20 to 29	255
Scotland	Asthma	age_strat	01/01/2016	30 to 39	233
Scotland	Asthma	age_strat	01/01/2016	40 to 49	286
Scotland	Asthma	age_strat	01/01/2016	5 to 9	341
Scotland	Asthma	age_strat	01/01/2016	50 to 59	265
Scotland	Asthma	age_strat	01/01/2016	60 to 69	190
Scotland	Asthma	age_strat	01/01/2016	70plus	170
Scotland	Asthma	age_strat	01/01/2017	0 to 4	156
Scotland	Asthma	age_strat	01/01/2017	10 to 14	256

Scotland	Asthma	age_strat	01/01/2017	15 to 19	116
Scotland	Asthma	age_strat	01/01/2017	20 to 29	263
Scotland	Asthma	age_strat	01/01/2017	30 to 39	294
Scotland	Asthma	age_strat	01/01/2017	40 to 49	251
Scotland	Asthma	age_strat	01/01/2017	5 to 9	343
Scotland	Asthma	age_strat	01/01/2017	50 to 59	292
Scotland	Asthma	age_strat	01/01/2017	60 to 69	214
Scotland	Asthma	age_strat	01/01/2017	70plus	172
Scotland	Asthma	age_strat	01/01/2018	0 to 4	174
Scotland	Asthma	age_strat	01/01/2018	10 to 14	202
Scotland	Asthma	age_strat	01/01/2018	15 to 19	109
Scotland	Asthma	age_strat	01/01/2018	20 to 29	306
Scotland	Asthma	age_strat	01/01/2018	30 to 39	266
Scotland	Asthma	age_strat	01/01/2018	40 to 49	269
Scotland	Asthma	age_strat	01/01/2018	5 to 9	318
Scotland	Asthma	age_strat	01/01/2018	50 to 59	328
Scotland	Asthma	age_strat	01/01/2018	60 to 69	245
Scotland	Asthma	age_strat	01/01/2018	70plus	215
Scotland	Asthma	age_strat	01/01/2019	0 to 4	132
Scotland	Asthma	age_strat	01/01/2019	10 to 14	223
Scotland	Asthma	age_strat	01/01/2019	15 to 19	124
Scotland	Asthma	age_strat	01/01/2019	20 to 29	363
Scotland	Asthma	age_strat	01/01/2019	30 to 39	323
Scotland	Asthma	age_strat	01/01/2019	40 to 49	287
Scotland	Asthma	age_strat	01/01/2019	5 to 9	323
Scotland	Asthma	age_strat	01/01/2019	50 to 59	322
Scotland	Asthma	age_strat	01/01/2019	60 to 69	248
Scotland	Asthma	age_strat	01/01/2019	70plus	224
Scotland	Asthma	age_strat	01/01/2020	0 to 4	35
Scotland	Asthma	age_strat	01/01/2020	10 to 14	60
Scotland	Asthma	age_strat	01/01/2020	15 to 19	25
Scotland	Asthma	age_strat	01/01/2020	20 to 29	84
Scotland	Asthma	age_strat	01/01/2020	30 to 39	71
Scotland	Asthma	age_strat	01/01/2020	40 to 49	76
Scotland	Asthma	age_strat	01/01/2020	5 to 9	91
Scotland	Asthma	age_strat	01/01/2020	50 to 59	76
Scotland	Asthma	age_strat	01/01/2020	60 to 69	58
Scotland	Asthma	age_strat	01/01/2020	70plus	49
Scotland	Asthma	age_strat	01/04/2020	0 to 4	13
Scotland	Asthma	age_strat	01/04/2020	10 to 14	19
Scotland	Asthma	age_strat	01/04/2020	15 to 19	10
Scotland	Asthma	age_strat	01/04/2020	20 to 29	37
Scotland	Asthma	age_strat	01/04/2020	30 to 39	45
Scotland	Asthma	age_strat	01/04/2020	40 to 49	47
Scotland	Asthma	age_strat	01/04/2020	5 to 9	43
Scotland	Asthma	age_strat	01/04/2020	50 to 59	37
Scotland	Asthma	age_strat	01/04/2020	60 to 69	24
Scotland	Asthma	age_strat	01/04/2020	70plus	15
Scotland	Asthma	age_strat	01/07/2020	0 to 4	14
Scotland	Asthma	age_strat	01/07/2020	10 to 14	25
Scotland	Asthma	age_strat	01/07/2020	15 to 19	17
Scotland	Asthma	age_strat	01/07/2020	20 to 29	79
Scotland	Asthma	age_strat	01/07/2020	30 to 39	54
Scotland	Asthma	age_strat	01/07/2020	40 to 49	55
Scotland	Asthma	age_strat	01/07/2020	5 to 9	52
Scotland	Asthma	age_strat	01/07/2020	50 to 59	60
Scotland	Asthma	age_strat	01/07/2020	60 to 69	40
Scotland	Asthma	age_strat	01/07/2020	70plus	26
Scotland	Asthma	age_strat	01/10/2020	0 to 4	26
Scotland	Asthma	age_strat	01/10/2020	10 to 14	47
Scotland	Asthma	age_strat	01/10/2020	15 to 19	34
Scotland	Asthma	age_strat	01/10/2020	20 to 29	62
Scotland	Asthma	age_strat	01/10/2020	30 to 39	67

Scotland	Asthma	age_strat	01/10/2020	40 to 49	58
Scotland	Asthma	age_strat	01/10/2020	5 to 9	75
Scotland	Asthma	age_strat	01/10/2020	50 to 59	61
Scotland	Asthma	age_strat	01/10/2020	60 to 69	39
Scotland	Asthma	age_strat	01/10/2020	70plus	34
Scotland	Asthma	age_strat	01/01/2021	0 to 4	15
Scotland	Asthma	age_strat	01/01/2021	10 to 14	27
Scotland	Asthma	age_strat	01/01/2021	15 to 19	21
Scotland	Asthma	age_strat	01/01/2021	20 to 29	76
Scotland	Asthma	age_strat	01/01/2021	30 to 39	73
Scotland	Asthma	age_strat	01/01/2021	40 to 49	60
Scotland	Asthma	age_strat	01/01/2021	5 to 9	40
Scotland	Asthma	age_strat	01/01/2021	50 to 59	59
Scotland	Asthma	age_strat	01/01/2021	60 to 69	40
Scotland	Asthma	age_strat	01/01/2021	70plus	33
Scotland	Asthma	age_strat	01/04/2021	0 to 4	26
Scotland	Asthma	age_strat	01/04/2021	10 to 14	46
Scotland	Asthma	age_strat	01/04/2021	15 to 19	17
Scotland	Asthma	age_strat	01/04/2021	20 to 29	78
Scotland	Asthma	age_strat	01/04/2021	30 to 39	70
Scotland	Asthma	age_strat	01/04/2021	40 to 49	56
Scotland	Asthma	age_strat	01/04/2021	5 to 9	63
Scotland	Asthma	age_strat	01/04/2021	50 to 59	58
Scotland	Asthma	age_strat	01/04/2021	60 to 69	39
Scotland	Asthma	age_strat	01/04/2021	70plus	54
Scotland	Asthma	age_strat	01/07/2021	0 to 4	29
Scotland	Asthma	age_strat	01/07/2021	10 to 14	36
Scotland	Asthma	age_strat	01/07/2021	15 to 19	27
Scotland	Asthma	age_strat	01/07/2021	20 to 29	66
Scotland	Asthma	age_strat	01/07/2021	30 to 39	66
Scotland	Asthma	age_strat	01/07/2021	40 to 49	60
Scotland	Asthma	age_strat	01/07/2021	5 to 9	66
Scotland	Asthma	age_strat	01/07/2021	50 to 59	72
Scotland	Asthma	age_strat	01/07/2021	60 to 69	44
Scotland	Asthma	age_strat	01/07/2021	70plus	41
Scotland	Asthma	age_strat	01/10/2021	0 to 4	36
Scotland	Asthma	age_strat	01/10/2021	10 to 14	57
Scotland	Asthma	age_strat	01/10/2021	15 to 19	42
Scotland	Asthma	age_strat	01/10/2021	20 to 29	81
Scotland	Asthma	age_strat	01/10/2021	30 to 39	80
Scotland	Asthma	age_strat	01/10/2021	40 to 49	78
Scotland	Asthma	age_strat	01/10/2021	5 to 9	101
Scotland	Asthma	age_strat	01/10/2021	50 to 59	74
Scotland	Asthma	age_strat	01/10/2021	60 to 69	43
Scotland	Asthma	age_strat	01/10/2021	70plus	47
Scotland	Asthma	age_strat	01/01/2022	0 to 4	27
Scotland	Asthma	age_strat	01/01/2022	10 to 14	50
Scotland	Asthma	age_strat	01/01/2022	15 to 19	28
Scotland	Asthma	age_strat	01/01/2022	20 to 29	79
Scotland	Asthma	age_strat	01/01/2022	30 to 39	88
Scotland	Asthma	age_strat	01/01/2022	40 to 49	62
Scotland	Asthma	age_strat	01/01/2022	5 to 9	86
Scotland	Asthma	age_strat	01/01/2022	50 to 59	52
Scotland	Asthma	age_strat	01/01/2022	60 to 69	67
Scotland	Asthma	age_strat	01/01/2022	70plus	38
Scotland	Asthma	age_strat	01/04/2022	0 to 4	40
Scotland	Asthma	age_strat	01/04/2022	10 to 14	45
Scotland	Asthma	age_strat	01/04/2022	15 to 19	40
Scotland	Asthma	age_strat	01/04/2022	20 to 29	95
Scotland	Asthma	age_strat	01/04/2022	30 to 39	66
Scotland	Asthma	age_strat	01/04/2022	40 to 49	56
Scotland	Asthma	age_strat	01/04/2022	5 to 9	102
Scotland	Asthma	age_strat	01/04/2022	50 to 59	61

Scotland	Asthma	age_strat	01/04/2022	60 to 69	82
Scotland	Asthma	age_strat	01/04/2022	70plus	50
Scotland	Asthma	age_strat	01/07/2022	0 to 4	25
Scotland	Asthma	age_strat	01/07/2022	10 to 14	54
Scotland	Asthma	age_strat	01/07/2022	15 to 19	31
Scotland	Asthma	age_strat	01/07/2022	20 to 29	83
Scotland	Asthma	age_strat	01/07/2022	30 to 39	77
Scotland	Asthma	age_strat	01/07/2022	40 to 49	78
Scotland	Asthma	age_strat	01/07/2022	5 to 9	88
Scotland	Asthma	age_strat	01/07/2022	50 to 59	79
Scotland	Asthma	age_strat	01/07/2022	60 to 69	60
Scotland	Asthma	age_strat	01/07/2022	70plus	59
Scotland	Asthma	age_strat	01/10/2022	0 to 4	38
Scotland	Asthma	age_strat	01/10/2022	10 to 14	67
Scotland	Asthma	age_strat	01/10/2022	15 to 19	45
Scotland	Asthma	age_strat	01/10/2022	20 to 29	110
Scotland	Asthma	age_strat	01/10/2022	30 to 39	87
Scotland	Asthma	age_strat	01/10/2022	40 to 49	65
Scotland	Asthma	age_strat	01/10/2022	5 to 9	113
Scotland	Asthma	age_strat	01/10/2022	50 to 59	85
Scotland	Asthma	age_strat	01/10/2022	60 to 69	70
Scotland	Asthma	age_strat	01/10/2022	70plus	44
Scotland	Asthma	age_strat	01/01/2023	0 to 4	32
Scotland	Asthma	age_strat	01/01/2023	10 to 14	65
Scotland	Asthma	age_strat	01/01/2023	15 to 19	22
Scotland	Asthma	age_strat	01/01/2023	20 to 29	82
Scotland	Asthma	age_strat	01/01/2023	30 to 39	108
Scotland	Asthma	age_strat	01/01/2023	40 to 49	84
Scotland	Asthma	age_strat	01/01/2023	5 to 9	140
Scotland	Asthma	age_strat	01/01/2023	50 to 59	77
Scotland	Asthma	age_strat	01/01/2023	60 to 69	86
Scotland	Asthma	age_strat	01/01/2023	70plus	55
Scotland	Asthma	sex_strat	01/01/2004 Female		1925
Scotland	Asthma	sex_strat	01/01/2005 Female		1744
Scotland	Asthma	sex_strat	01/01/2006 Female		1410
Scotland	Asthma	sex_strat	01/01/2007 Female		1338
Scotland	Asthma	sex_strat	01/01/2008 Female		1445
Scotland	Asthma	sex_strat	01/01/2009 Female		1429
Scotland	Asthma	sex_strat	01/01/2010 Female		1315
Scotland	Asthma	sex_strat	01/01/2011 Female		1239
Scotland	Asthma	sex_strat	01/01/2012 Female		1349
Scotland	Asthma	sex_strat	01/01/2013 Female		1288
Scotland	Asthma	sex_strat	01/01/2014 Female		1344
Scotland	Asthma	sex_strat	01/01/2015 Female		1236
Scotland	Asthma	sex_strat	01/01/2016 Female		1230
Scotland	Asthma	sex_strat	01/01/2017 Female		1332
Scotland	Asthma	sex_strat	01/01/2018 Female		1340
Scotland	Asthma	sex_strat	01/01/2019 Female		1438
Scotland	Asthma	sex_strat	01/01/2020 Female		382
Scotland	Asthma	sex_strat	01/01/2020 Female		1106
Scotland	Asthma	sex_strat	01/04/2020 Female		153
Scotland	Asthma	sex_strat	01/07/2020 Female		234
Scotland	Asthma	sex_strat	01/10/2020 Female		282
Scotland	Asthma	sex_strat	01/01/2021 Female		255
Scotland	Asthma	sex_strat	01/01/2021 Female		1198
Scotland	Asthma	sex_strat	01/04/2021 Female		286
Scotland	Asthma	sex_strat	01/07/2021 Female		263
Scotland	Asthma	sex_strat	01/10/2021 Female		342
Scotland	Asthma	sex_strat	01/01/2022 Female		325
Scotland	Asthma	sex_strat	01/01/2022 Female		1471
Scotland	Asthma	sex_strat	01/04/2022 Female		347
Scotland	Asthma	sex_strat	01/07/2022 Female		345
Scotland	Asthma	sex_strat	01/10/2022 Female		386

Scotland	Asthma	sex_strat	01/01/2023	Female	411
Scotland	Asthma	sex_strat	01/01/2004	Male	1676
Scotland	Asthma	sex_strat	01/01/2005	Male	1462
Scotland	Asthma	sex_strat	01/01/2006	Male	1208
Scotland	Asthma	sex_strat	01/01/2007	Male	1162
Scotland	Asthma	sex_strat	01/01/2008	Male	1228
Scotland	Asthma	sex_strat	01/01/2009	Male	1242
Scotland	Asthma	sex_strat	01/01/2010	Male	1105
Scotland	Asthma	sex_strat	01/01/2011	Male	1137
Scotland	Asthma	sex_strat	01/01/2012	Male	1215
Scotland	Asthma	sex_strat	01/01/2013	Male	1120
Scotland	Asthma	sex_strat	01/01/2014	Male	1172
Scotland	Asthma	sex_strat	01/01/2015	Male	1062
Scotland	Asthma	sex_strat	01/01/2016	Male	1046
Scotland	Asthma	sex_strat	01/01/2017	Male	1025
Scotland	Asthma	sex_strat	01/01/2018	Male	1092
Scotland	Asthma	sex_strat	01/01/2019	Male	1131
Scotland	Asthma	sex_strat	01/01/2020	Male	243
Scotland	Asthma	sex_strat	01/01/2020	Male	817
Scotland	Asthma	sex_strat	01/04/2020	Male	137
Scotland	Asthma	sex_strat	01/07/2020	Male	188
Scotland	Asthma	sex_strat	01/10/2020	Male	221
Scotland	Asthma	sex_strat	01/01/2021	Male	189
Scotland	Asthma	sex_strat	01/01/2021	Male	991
Scotland	Asthma	sex_strat	01/04/2021	Male	221
Scotland	Asthma	sex_strat	01/07/2021	Male	244
Scotland	Asthma	sex_strat	01/10/2021	Male	297
Scotland	Asthma	sex_strat	01/01/2022	Male	252
Scotland	Asthma	sex_strat	01/01/2022	Male	1219
Scotland	Asthma	sex_strat	01/04/2022	Male	290
Scotland	Asthma	sex_strat	01/07/2022	Male	289
Scotland	Asthma	sex_strat	01/10/2022	Male	338
Scotland	Asthma	sex_strat	01/01/2023	Male	340
Scotland	COPD	sex_strat	01/01/2004	Female	798
Scotland	COPD	sex_strat	01/01/2005	Female	730
Scotland	COPD	sex_strat	01/01/2006	Female	813
Scotland	COPD	sex_strat	01/01/2007	Female	743
Scotland	COPD	sex_strat	01/01/2008	Female	761
Scotland	COPD	sex_strat	01/01/2009	Female	729
Scotland	COPD	sex_strat	01/01/2010	Female	842
Scotland	COPD	sex_strat	01/01/2011	Female	797
Scotland	COPD	sex_strat	01/01/2012	Female	940
Scotland	COPD	sex_strat	01/01/2013	Female	842
Scotland	COPD	sex_strat	01/01/2014	Female	897
Scotland	COPD	sex_strat	01/01/2015	Female	939
Scotland	COPD	sex_strat	01/01/2016	Female	945
Scotland	COPD	sex_strat	01/01/2017	Female	921
Scotland	COPD	sex_strat	01/01/2018	Female	978
Scotland	COPD	sex_strat	01/01/2019	Female	964
Scotland	COPD	sex_strat	01/01/2020	Female	203
Scotland	COPD	sex_strat	01/01/2020	Female	614
Scotland	COPD	sex_strat	01/04/2020	Female	89
Scotland	COPD	sex_strat	01/07/2020	Female	183
Scotland	COPD	sex_strat	01/10/2020	Female	134
Scotland	COPD	sex_strat	01/01/2021	Female	121
Scotland	COPD	sex_strat	01/01/2021	Female	620
Scotland	COPD	sex_strat	01/04/2021	Female	152
Scotland	COPD	sex_strat	01/07/2021	Female	170
Scotland	COPD	sex_strat	01/10/2021	Female	171
Scotland	COPD	sex_strat	01/01/2022	Female	208
Scotland	COPD	sex_strat	01/01/2022	Female	774
Scotland	COPD	sex_strat	01/04/2022	Female	196
Scotland	COPD	sex_strat	01/07/2022	Female	181

Scotland	COPD	sex_strat	01/10/2022	Female	185
Scotland	COPD	sex_strat	01/01/2023	Female	213
Scotland	COPD	sex_strat	01/01/2004	Male	822
Scotland	COPD	sex_strat	01/01/2005	Male	801
Scotland	COPD	sex_strat	01/01/2006	Male	730
Scotland	COPD	sex_strat	01/01/2007	Male	691
Scotland	COPD	sex_strat	01/01/2008	Male	683
Scotland	COPD	sex_strat	01/01/2009	Male	655
Scotland	COPD	sex_strat	01/01/2010	Male	831
Scotland	COPD	sex_strat	01/01/2011	Male	783
Scotland	COPD	sex_strat	01/01/2012	Male	992
Scotland	COPD	sex_strat	01/01/2013	Male	856
Scotland	COPD	sex_strat	01/01/2014	Male	808
Scotland	COPD	sex_strat	01/01/2015	Male	954
Scotland	COPD	sex_strat	01/01/2016	Male	921
Scotland	COPD	sex_strat	01/01/2017	Male	933
Scotland	COPD	sex_strat	01/01/2018	Male	897
Scotland	COPD	sex_strat	01/01/2019	Male	934
Scotland	COPD	sex_strat	01/01/2020	Male	234
Scotland	COPD	sex_strat	01/01/2020	Male	622
Scotland	COPD	sex_strat	01/04/2020	Male	100
Scotland	COPD	sex_strat	01/07/2020	Male	141
Scotland	COPD	sex_strat	01/10/2020	Male	142
Scotland	COPD	sex_strat	01/01/2021	Male	119
Scotland	COPD	sex_strat	01/01/2021	Male	676
Scotland	COPD	sex_strat	01/04/2021	Male	168
Scotland	COPD	sex_strat	01/07/2021	Male	191
Scotland	COPD	sex_strat	01/10/2021	Male	190
Scotland	COPD	sex_strat	01/01/2022	Male	176
Scotland	COPD	sex_strat	01/01/2022	Male	780
Scotland	COPD	sex_strat	01/04/2022	Male	185
Scotland	COPD	sex_strat	01/07/2022	Male	195
Scotland	COPD	sex_strat	01/10/2022	Male	222
Scotland	COPD	sex_strat	01/01/2023	Male	214
Scotland	ILD	sex_strat	01/01/2004	Female	51
Scotland	ILD	sex_strat	01/01/2005	Female	58
Scotland	ILD	sex_strat	01/01/2006	Female	69
Scotland	ILD	sex_strat	01/01/2007	Female	75
Scotland	ILD	sex_strat	01/01/2008	Female	78
Scotland	ILD	sex_strat	01/01/2009	Female	72
Scotland	ILD	sex_strat	01/01/2010	Female	94
Scotland	ILD	sex_strat	01/01/2011	Female	83
Scotland	ILD	sex_strat	01/01/2012	Female	80
Scotland	ILD	sex_strat	01/01/2013	Female	91
Scotland	ILD	sex_strat	01/01/2014	Female	76
Scotland	ILD	sex_strat	01/01/2015	Female	105
Scotland	ILD	sex_strat	01/01/2016	Female	99
Scotland	ILD	sex_strat	01/01/2017	Female	107
Scotland	ILD	sex_strat	01/01/2018	Female	121
Scotland	ILD	sex_strat	01/01/2019	Female	144
Scotland	ILD	sex_strat	01/01/2020	Female	35
Scotland	ILD	sex_strat	01/01/2020	Female	122
Scotland	ILD	sex_strat	01/04/2020	Female	27
Scotland	ILD	sex_strat	01/07/2020	Female	30
Scotland	ILD	sex_strat	01/10/2020	Female	28
Scotland	ILD	sex_strat	01/01/2021	Female	26
Scotland	ILD	sex_strat	01/01/2021	Female	110
Scotland	ILD	sex_strat	01/04/2021	Female	31
Scotland	ILD	sex_strat	01/07/2021	Female	24
Scotland	ILD	sex_strat	01/10/2021	Female	29
Scotland	ILD	sex_strat	01/01/2022	Female	35
Scotland	ILD	sex_strat	01/01/2022	Female	145
Scotland	ILD	sex_strat	01/04/2022	Female	47

Scotland	ILD	sex_strat	01/07/2022	Female	28
Scotland	ILD	sex_strat	01/10/2022	Female	33
Scotland	ILD	sex_strat	01/01/2023	Female	27
Scotland	ILD	sex_strat	01/01/2004	Male	105
Scotland	ILD	sex_strat	01/01/2005	Male	117
Scotland	ILD	sex_strat	01/01/2006	Male	124
Scotland	ILD	sex_strat	01/01/2007	Male	123
Scotland	ILD	sex_strat	01/01/2008	Male	131
Scotland	ILD	sex_strat	01/01/2009	Male	135
Scotland	ILD	sex_strat	01/01/2010	Male	171
Scotland	ILD	sex_strat	01/01/2011	Male	137
Scotland	ILD	sex_strat	01/01/2012	Male	171
Scotland	ILD	sex_strat	01/01/2013	Male	156
Scotland	ILD	sex_strat	01/01/2014	Male	159
Scotland	ILD	sex_strat	01/01/2015	Male	168
Scotland	ILD	sex_strat	01/01/2016	Male	143
Scotland	ILD	sex_strat	01/01/2017	Male	199
Scotland	ILD	sex_strat	01/01/2018	Male	186
Scotland	ILD	sex_strat	01/01/2019	Male	205
Scotland	ILD	sex_strat	01/01/2020	Male	40
Scotland	ILD	sex_strat	01/01/2020	Male	150
Scotland	ILD	sex_strat	01/04/2020	Male	30
Scotland	ILD	sex_strat	01/07/2020	Male	35
Scotland	ILD	sex_strat	01/10/2020	Male	44
Scotland	ILD	sex_strat	01/01/2021	Male	44
Scotland	ILD	sex_strat	01/01/2021	Male	192
Scotland	ILD	sex_strat	01/04/2021	Male	54
Scotland	ILD	sex_strat	01/07/2021	Male	43
Scotland	ILD	sex_strat	01/10/2021	Male	49
Scotland	ILD	sex_strat	01/01/2022	Male	56
Scotland	ILD	sex_strat	01/01/2022	Male	211
Scotland	ILD	sex_strat	01/04/2022	Male	51
Scotland	ILD	sex_strat	01/07/2022	Male	56
Scotland	ILD	sex_strat	01/10/2022	Male	46
Scotland	ILD	sex_strat	01/01/2023	Male	42
Wales	Asthma	all	01/01/2004		19668
Wales	Asthma	all	01/01/2005		17463
Wales	Asthma	all	01/01/2006		13965
Wales	Asthma	all	01/01/2007		12203
Wales	Asthma	all	01/01/2008		12089
Wales	Asthma	all	01/01/2009		12190
Wales	Asthma	all	01/01/2010		11415
Wales	Asthma	all	01/01/2011		11299
Wales	Asthma	all	01/01/2012		12356
Wales	Asthma	all	01/01/2013		10780
Wales	Asthma	all	01/01/2014		11159
Wales	Asthma	all	01/01/2015		15526
Wales	Asthma	all	01/01/2016		11434
Wales	Asthma	all	01/01/2017		11166
Wales	Asthma	all	01/01/2018		10677
Wales	Asthma	all	01/01/2019		11184
Wales	COPD	all	01/01/2004		8500
Wales	COPD	all	01/01/2005		9608
Wales	COPD	all	01/01/2006		8931
Wales	COPD	all	01/01/2007		8377
Wales	COPD	all	01/01/2008		7504
Wales	COPD	all	01/01/2009		8619
Wales	COPD	all	01/01/2010		7793
Wales	COPD	all	01/01/2011		7329
Wales	COPD	all	01/01/2012		7139
Wales	COPD	all	01/01/2013		6774
Wales	COPD	all	01/01/2014		6571
Wales	COPD	all	01/01/2015		6791

Wales	COPD	all	01/01/2016		7140
Wales	COPD	all	01/01/2017		6987
Wales	COPD	all	01/01/2018		6898
Wales	COPD	all	01/01/2019		6790
Wales	ILD	all	01/01/2004		798
Wales	ILD	all	01/01/2005		745
Wales	ILD	all	01/01/2006		732
Wales	ILD	all	01/01/2007		737
Wales	ILD	all	01/01/2008		749
Wales	ILD	all	01/01/2009		703
Wales	ILD	all	01/01/2010		777
Wales	ILD	all	01/01/2011		807
Wales	ILD	all	01/01/2012		831
Wales	ILD	all	01/01/2013		827
Wales	ILD	all	01/01/2014		860
Wales	ILD	all	01/01/2015		952
Wales	ILD	all	01/01/2016		967
Wales	ILD	all	01/01/2017		1059
Wales	ILD	all	01/01/2018		1079
Wales	ILD	all	01/01/2019		1155
Wales	Asthma	sex_strat_children	01/01/2018	Female	1778
Wales	Asthma	sex_strat_children	01/01/2013	Female	1916
Wales	Asthma	sex_strat_children	01/01/2007	Female	2015
Wales	Asthma	sex_strat_children	01/01/2019	Female	2047
Wales	Asthma	sex_strat_children	01/01/2010	Female	2052
Wales	Asthma	sex_strat_children	01/01/2014	Female	2070
Wales	Asthma	sex_strat_children	01/01/2017	Female	2093
Wales	Asthma	sex_strat_children	01/01/2016	Female	2098
Wales	Asthma	sex_strat_children	01/01/2011	Female	2128
Wales	Asthma	sex_strat_children	01/01/2018	Male	2137
Wales	Asthma	sex_strat_children	01/01/2009	Female	2137
Wales	Asthma	sex_strat_children	01/01/2008	Female	2164
Wales	Asthma	sex_strat_children	01/01/2012	Female	2305
Wales	Asthma	sex_strat_children	01/01/2019	Male	2338
Wales	Asthma	sex_strat_children	01/01/2006	Female	2338
Wales	Asthma	sex_strat_children	01/01/2017	Male	2395
Wales	Asthma	sex_strat_children	01/01/2010	Male	2476
Wales	Asthma	sex_strat_children	01/01/2013	Male	2484
Wales	Asthma	sex_strat_children	01/01/2016	Male	2499
Wales	Asthma	sex_strat_children	01/01/2014	Male	2517
Wales	Asthma	sex_strat_children	01/01/2007	Male	2541
Wales	Asthma	sex_strat_children	01/01/2015	Female	2547
Wales	Asthma	sex_strat_children	01/01/2011	Male	2558
Wales	Asthma	sex_strat_children	01/01/2008	Male	2560
Wales	Asthma	sex_strat_children	01/01/2009	Male	2699
Wales	Asthma	sex_strat_children	01/01/2005	Female	2710
Wales	Asthma	sex_strat_children	01/01/2006	Male	2801
Wales	Asthma	sex_strat_children	01/01/2012	Male	2855
Wales	Asthma	sex_strat_children	01/01/2015	Male	2881
Wales	Asthma	sex_strat_children	01/01/2004	Female	2959
Wales	Asthma	sex_strat_children	01/01/2005	Male	3128
Wales	Asthma	sex_strat_children	01/01/2004	Male	3478
Wales	Asthma	sex_strat_adults	01/01/2013	Male	2614
Wales	Asthma	sex_strat_adults	01/01/2017	Male	2663
Wales	Asthma	sex_strat_adults	01/01/2014	Male	2707
Wales	Asthma	sex_strat_adults	01/01/2016	Male	2785
Wales	Asthma	sex_strat_adults	01/01/2019	Male	2815
Wales	Asthma	sex_strat_adults	01/01/2011	Male	2847
Wales	Asthma	sex_strat_adults	01/01/2018	Male	2881
Wales	Asthma	sex_strat_adults	01/01/2010	Male	2967
Wales	Asthma	sex_strat_adults	01/01/2012	Male	3125
Wales	Asthma	sex_strat_adults	01/01/2008	Male	3183
Wales	Asthma	sex_strat_adults	01/01/2009	Male	3229

Wales	Asthma	sex_strat_adults	01/01/2007	Male		3290
Wales	Asthma	sex_strat_adults	01/01/2006	Male		3732
Wales	Asthma	sex_strat_adults	01/01/2013	Female		3766
Wales	Asthma	sex_strat_adults	01/01/2011	Female		3766
Wales	Asthma	sex_strat_adults	01/01/2014	Female		3865
Wales	Asthma	sex_strat_adults	01/01/2018	Female		3881
Wales	Asthma	sex_strat_adults	01/01/2010	Female		3920
Wales	Asthma	sex_strat_adults	01/01/2019	Female		3984
Wales	Asthma	sex_strat_adults	01/01/2017	Female		4015
Wales	Asthma	sex_strat_adults	01/01/2016	Female		4052
Wales	Asthma	sex_strat_adults	01/01/2012	Female		4071
Wales	Asthma	sex_strat_adults	01/01/2009	Female		4125
Wales	Asthma	sex_strat_adults	01/01/2008	Female		4182
Wales	Asthma	sex_strat_adults	01/01/2007	Female		4357
Wales	Asthma	sex_strat_adults	01/01/2015	Male		4445
Wales	Asthma	sex_strat_adults	01/01/2005	Male		4904
Wales	Asthma	sex_strat_adults	01/01/2006	Female		5094
Wales	Asthma	sex_strat_adults	01/01/2004	Male		5609
Wales	Asthma	sex_strat_adults	01/01/2015	Female		5653
Wales	Asthma	sex_strat_adults	01/01/2005	Female		6721
Wales	Asthma	sex_strat_adults	01/01/2004	Female		7622
Wales	Asthma	age_strat	01/01/2004		0 to 4	2586
Wales	Asthma	age_strat	01/01/2004		10 to 14	1200
Wales	Asthma	age_strat	01/01/2004		15 to 19	839
Wales	Asthma	age_strat	01/01/2004		20 to 29	1396
Wales	Asthma	age_strat	01/01/2004		30 to 39	1785
Wales	Asthma	age_strat	01/01/2004		40 to 49	1903
Wales	Asthma	age_strat	01/01/2004		5 to 9	1812
Wales	Asthma	age_strat	01/01/2004		50 to 59	2307
Wales	Asthma	age_strat	01/01/2004		60 to 69	2712
Wales	Asthma	age_strat	01/01/2004		70plus	3128
Wales	Asthma	age_strat	01/01/2005		0 to 4	2145
Wales	Asthma	age_strat	01/01/2005		10 to 14	1229
Wales	Asthma	age_strat	01/01/2005		15 to 19	763
Wales	Asthma	age_strat	01/01/2005		20 to 29	1340
Wales	Asthma	age_strat	01/01/2005		30 to 39	1662
Wales	Asthma	age_strat	01/01/2005		40 to 49	1846
Wales	Asthma	age_strat	01/01/2005		5 to 9	1701
Wales	Asthma	age_strat	01/01/2005		50 to 59	1992
Wales	Asthma	age_strat	01/01/2005		60 to 69	2258
Wales	Asthma	age_strat	01/01/2005		70plus	2527
Wales	Asthma	age_strat	01/01/2006		0 to 4	2111
Wales	Asthma	age_strat	01/01/2006		10 to 14	993
Wales	Asthma	age_strat	01/01/2006		15 to 19	606
Wales	Asthma	age_strat	01/01/2006		20 to 29	1003
Wales	Asthma	age_strat	01/01/2006		30 to 39	1178
Wales	Asthma	age_strat	01/01/2006		40 to 49	1435
Wales	Asthma	age_strat	01/01/2006		5 to 9	1429
Wales	Asthma	age_strat	01/01/2006		50 to 59	1551
Wales	Asthma	age_strat	01/01/2006		60 to 69	1739
Wales	Asthma	age_strat	01/01/2006		70plus	1920
Wales	Asthma	age_strat	01/01/2007		0 to 4	1825
Wales	Asthma	age_strat	01/01/2007		10 to 14	862
Wales	Asthma	age_strat	01/01/2007		15 to 19	563
Wales	Asthma	age_strat	01/01/2007		20 to 29	868
Wales	Asthma	age_strat	01/01/2007		30 to 39	1058
Wales	Asthma	age_strat	01/01/2007		40 to 49	1255
Wales	Asthma	age_strat	01/01/2007		5 to 9	1306
Wales	Asthma	age_strat	01/01/2007		50 to 59	1329
Wales	Asthma	age_strat	01/01/2007		60 to 69	1522
Wales	Asthma	age_strat	01/01/2007		70plus	1615
Wales	Asthma	age_strat	01/01/2008		0 to 4	1854
Wales	Asthma	age_strat	01/01/2008		10 to 14	977

Wales	Asthma	age_strat	01/01/2008	15 to 19	621
Wales	Asthma	age_strat	01/01/2008	20 to 29	917
Wales	Asthma	age_strat	01/01/2008	30 to 39	1080
Wales	Asthma	age_strat	01/01/2008	40 to 49	1348
Wales	Asthma	age_strat	01/01/2008	5 to 9	1272
Wales	Asthma	age_strat	01/01/2008	50 to 59	1282
Wales	Asthma	age_strat	01/01/2008	60 to 69	1418
Wales	Asthma	age_strat	01/01/2008	70plus	1320
Wales	Asthma	age_strat	01/01/2009	0 to 4	1809
Wales	Asthma	age_strat	01/01/2009	10 to 14	1030
Wales	Asthma	age_strat	01/01/2009	15 to 19	570
Wales	Asthma	age_strat	01/01/2009	20 to 29	947
Wales	Asthma	age_strat	01/01/2009	30 to 39	985
Wales	Asthma	age_strat	01/01/2009	40 to 49	1322
Wales	Asthma	age_strat	01/01/2009	5 to 9	1427
Wales	Asthma	age_strat	01/01/2009	50 to 59	1345
Wales	Asthma	age_strat	01/01/2009	60 to 69	1424
Wales	Asthma	age_strat	01/01/2009	70plus	1331
Wales	Asthma	age_strat	01/01/2010	0 to 4	1750
Wales	Asthma	age_strat	01/01/2010	10 to 14	932
Wales	Asthma	age_strat	01/01/2010	15 to 19	534
Wales	Asthma	age_strat	01/01/2010	20 to 29	910
Wales	Asthma	age_strat	01/01/2010	30 to 39	890
Wales	Asthma	age_strat	01/01/2010	40 to 49	1272
Wales	Asthma	age_strat	01/01/2010	5 to 9	1312
Wales	Asthma	age_strat	01/01/2010	50 to 59	1297
Wales	Asthma	age_strat	01/01/2010	60 to 69	1326
Wales	Asthma	age_strat	01/01/2010	70plus	1192
Wales	Asthma	age_strat	01/01/2011	0 to 4	1774
Wales	Asthma	age_strat	01/01/2011	10 to 14	881
Wales	Asthma	age_strat	01/01/2011	15 to 19	498
Wales	Asthma	age_strat	01/01/2011	20 to 29	866
Wales	Asthma	age_strat	01/01/2011	30 to 39	858
Wales	Asthma	age_strat	01/01/2011	40 to 49	1190
Wales	Asthma	age_strat	01/01/2011	5 to 9	1533
Wales	Asthma	age_strat	01/01/2011	50 to 59	1229
Wales	Asthma	age_strat	01/01/2011	60 to 69	1280
Wales	Asthma	age_strat	01/01/2011	70plus	1190
Wales	Asthma	age_strat	01/01/2012	0 to 4	1883
Wales	Asthma	age_strat	01/01/2012	10 to 14	1012
Wales	Asthma	age_strat	01/01/2012	15 to 19	534
Wales	Asthma	age_strat	01/01/2012	20 to 29	944
Wales	Asthma	age_strat	01/01/2012	30 to 39	878
Wales	Asthma	age_strat	01/01/2012	40 to 49	1344
Wales	Asthma	age_strat	01/01/2012	5 to 9	1731
Wales	Asthma	age_strat	01/01/2012	50 to 59	1280
Wales	Asthma	age_strat	01/01/2012	60 to 69	1407
Wales	Asthma	age_strat	01/01/2012	70plus	1343
Wales	Asthma	age_strat	01/01/2013	0 to 4	1491
Wales	Asthma	age_strat	01/01/2013	10 to 14	875
Wales	Asthma	age_strat	01/01/2013	15 to 19	530
Wales	Asthma	age_strat	01/01/2013	20 to 29	891
Wales	Asthma	age_strat	01/01/2013	30 to 39	811
Wales	Asthma	age_strat	01/01/2013	40 to 49	1154
Wales	Asthma	age_strat	01/01/2013	5 to 9	1504
Wales	Asthma	age_strat	01/01/2013	50 to 59	1154
Wales	Asthma	age_strat	01/01/2013	60 to 69	1274
Wales	Asthma	age_strat	01/01/2013	70plus	1096
Wales	Asthma	age_strat	01/01/2014	0 to 4	1575
Wales	Asthma	age_strat	01/01/2014	10 to 14	928
Wales	Asthma	age_strat	01/01/2014	15 to 19	552
Wales	Asthma	age_strat	01/01/2014	20 to 29	880
Wales	Asthma	age_strat	01/01/2014	30 to 39	830

Wales	Asthma	age_strat	01/01/2014	40 to 49	1167
Wales	Asthma	age_strat	01/01/2014	5 to 9	1532
Wales	Asthma	age_strat	01/01/2014	50 to 59	1241
Wales	Asthma	age_strat	01/01/2014	60 to 69	1262
Wales	Asthma	age_strat	01/01/2014	70plus	1192
Wales	Asthma	age_strat	01/01/2015	0 to 4	1599
Wales	Asthma	age_strat	01/01/2015	10 to 14	1232
Wales	Asthma	age_strat	01/01/2015	15 to 19	727
Wales	Asthma	age_strat	01/01/2015	20 to 29	1214
Wales	Asthma	age_strat	01/01/2015	30 to 39	1189
Wales	Asthma	age_strat	01/01/2015	40 to 49	1625
Wales	Asthma	age_strat	01/01/2015	5 to 9	1870
Wales	Asthma	age_strat	01/01/2015	50 to 59	2016
Wales	Asthma	age_strat	01/01/2015	60 to 69	1937
Wales	Asthma	age_strat	01/01/2015	70plus	2117
Wales	Asthma	age_strat	01/01/2016	0 to 4	1382
Wales	Asthma	age_strat	01/01/2016	10 to 14	1007
Wales	Asthma	age_strat	01/01/2016	15 to 19	539
Wales	Asthma	age_strat	01/01/2016	20 to 29	999
Wales	Asthma	age_strat	01/01/2016	30 to 39	857
Wales	Asthma	age_strat	01/01/2016	40 to 49	1128
Wales	Asthma	age_strat	01/01/2016	5 to 9	1669
Wales	Asthma	age_strat	01/01/2016	50 to 59	1300
Wales	Asthma	age_strat	01/01/2016	60 to 69	1268
Wales	Asthma	age_strat	01/01/2016	70plus	1285
Wales	Asthma	age_strat	01/01/2017	0 to 4	1176
Wales	Asthma	age_strat	01/01/2017	10 to 14	1094
Wales	Asthma	age_strat	01/01/2017	15 to 19	542
Wales	Asthma	age_strat	01/01/2017	20 to 29	943
Wales	Asthma	age_strat	01/01/2017	30 to 39	877
Wales	Asthma	age_strat	01/01/2017	40 to 49	1041
Wales	Asthma	age_strat	01/01/2017	5 to 9	1676
Wales	Asthma	age_strat	01/01/2017	50 to 59	1269
Wales	Asthma	age_strat	01/01/2017	60 to 69	1266
Wales	Asthma	age_strat	01/01/2017	70plus	1282
Wales	Asthma	age_strat	01/01/2018	0 to 4	988
Wales	Asthma	age_strat	01/01/2018	10 to 14	983
Wales	Asthma	age_strat	01/01/2018	15 to 19	506
Wales	Asthma	age_strat	01/01/2018	20 to 29	849
Wales	Asthma	age_strat	01/01/2018	30 to 39	858
Wales	Asthma	age_strat	01/01/2018	40 to 49	973
Wales	Asthma	age_strat	01/01/2018	5 to 9	1438
Wales	Asthma	age_strat	01/01/2018	50 to 59	1369
Wales	Asthma	age_strat	01/01/2018	60 to 69	1253
Wales	Asthma	age_strat	01/01/2018	70plus	1460
Wales	Asthma	age_strat	01/01/2019	0 to 4	1048
Wales	Asthma	age_strat	01/01/2019	10 to 14	1098
Wales	Asthma	age_strat	01/01/2019	15 to 19	542
Wales	Asthma	age_strat	01/01/2019	20 to 29	939
Wales	Asthma	age_strat	01/01/2019	30 to 39	891
Wales	Asthma	age_strat	01/01/2019	40 to 49	994
Wales	Asthma	age_strat	01/01/2019	5 to 9	1697
Wales	Asthma	age_strat	01/01/2019	50 to 59	1287
Wales	Asthma	age_strat	01/01/2019	60 to 69	1226
Wales	Asthma	age_strat	01/01/2019	70plus	1462
Wales	COPD	age_strat	01/01/2004	40 to 49	464
Wales	COPD	age_strat	01/01/2005	40 to 49	569
Wales	COPD	age_strat	01/01/2006	40 to 49	545
Wales	COPD	age_strat	01/01/2007	40 to 49	556
Wales	COPD	age_strat	01/01/2008	40 to 49	562
Wales	COPD	age_strat	01/01/2009	40 to 49	662
Wales	COPD	age_strat	01/01/2010	40 to 49	673
Wales	COPD	age_strat	01/01/2011	40 to 49	665

Wales	COPD	age_strat	01/01/2012	40 to 49	653
Wales	COPD	age_strat	01/01/2013	40 to 49	536
Wales	COPD	age_strat	01/01/2014	40 to 49	583
Wales	COPD	age_strat	01/01/2015	40 to 49	536
Wales	COPD	age_strat	01/01/2016	40 to 49	565
Wales	COPD	age_strat	01/01/2017	40 to 49	510
Wales	COPD	age_strat	01/01/2018	40 to 49	516
Wales	COPD	age_strat	01/01/2019	40 to 49	461
Wales	COPD	age_strat	01/01/2004	50 to 59	1463
Wales	COPD	age_strat	01/01/2005	50 to 59	1738
Wales	COPD	age_strat	01/01/2006	50 to 59	1660
Wales	COPD	age_strat	01/01/2007	50 to 59	1496
Wales	COPD	age_strat	01/01/2008	50 to 59	1379
Wales	COPD	age_strat	01/01/2009	50 to 59	1644
Wales	COPD	age_strat	01/01/2010	50 to 59	1468
Wales	COPD	age_strat	01/01/2011	50 to 59	1450
Wales	COPD	age_strat	01/01/2012	50 to 59	1429
Wales	COPD	age_strat	01/01/2013	50 to 59	1371
Wales	COPD	age_strat	01/01/2014	50 to 59	1300
Wales	COPD	age_strat	01/01/2015	50 to 59	1288
Wales	COPD	age_strat	01/01/2016	50 to 59	1414
Wales	COPD	age_strat	01/01/2017	50 to 59	1387
Wales	COPD	age_strat	01/01/2018	50 to 59	1439
Wales	COPD	age_strat	01/01/2019	50 to 59	1371
Wales	COPD	age_strat	01/01/2004	60 to 69	2561
Wales	COPD	age_strat	01/01/2005	60 to 69	2902
Wales	COPD	age_strat	01/01/2006	60 to 69	2716
Wales	COPD	age_strat	01/01/2007	60 to 69	2738
Wales	COPD	age_strat	01/01/2008	60 to 69	2362
Wales	COPD	age_strat	01/01/2009	60 to 69	2688
Wales	COPD	age_strat	01/01/2010	60 to 69	2528
Wales	COPD	age_strat	01/01/2011	60 to 69	2356
Wales	COPD	age_strat	01/01/2012	60 to 69	2254
Wales	COPD	age_strat	01/01/2013	60 to 69	2200
Wales	COPD	age_strat	01/01/2014	60 to 69	2037
Wales	COPD	age_strat	01/01/2015	60 to 69	2178
Wales	COPD	age_strat	01/01/2016	60 to 69	2182
Wales	COPD	age_strat	01/01/2017	60 to 69	2095
Wales	COPD	age_strat	01/01/2018	60 to 69	1987
Wales	COPD	age_strat	01/01/2019	60 to 69	1911
Wales	COPD	age_strat	01/01/2004	70plus	3924
Wales	COPD	age_strat	01/01/2005	70plus	4298
Wales	COPD	age_strat	01/01/2006	70plus	3901
Wales	COPD	age_strat	01/01/2007	70plus	3488
Wales	COPD	age_strat	01/01/2008	70plus	3087
Wales	COPD	age_strat	01/01/2009	70plus	3503
Wales	COPD	age_strat	01/01/2010	70plus	3006
Wales	COPD	age_strat	01/01/2011	70plus	2738
Wales	COPD	age_strat	01/01/2012	70plus	2700
Wales	COPD	age_strat	01/01/2013	70plus	2578
Wales	COPD	age_strat	01/01/2014	70plus	2566
Wales	COPD	age_strat	01/01/2015	70plus	2695
Wales	COPD	age_strat	01/01/2016	70plus	2887
Wales	COPD	age_strat	01/01/2017	70plus	2913
Wales	COPD	age_strat	01/01/2018	70plus	2870
Wales	COPD	age_strat	01/01/2019	70plus	2964
Wales	ILD	age_strat	01/01/2004	40 to 49	39
Wales	ILD	age_strat	01/01/2005	40 to 49	48
Wales	ILD	age_strat	01/01/2006	40 to 49	46
Wales	ILD	age_strat	01/01/2007	40 to 49	48
Wales	ILD	age_strat	01/01/2008	40 to 49	36
Wales	ILD	age_strat	01/01/2009	40 to 49	33
Wales	ILD	age_strat	01/01/2010	40 to 49	45

Wales	ILD	age_strat	01/01/2011		40 to 49	46
Wales	ILD	age_strat	01/01/2012		40 to 49	52
Wales	ILD	age_strat	01/01/2013		40 to 49	31
Wales	ILD	age_strat	01/01/2014		40 to 49	43
Wales	ILD	age_strat	01/01/2015		40 to 49	30
Wales	ILD	age_strat	01/01/2016		40 to 49	43
Wales	ILD	age_strat	01/01/2017		40 to 49	37
Wales	ILD	age_strat	01/01/2018		40 to 49	39
Wales	ILD	age_strat	01/01/2019		40 to 49	42
Wales	ILD	age_strat	01/01/2004		50 to 59	101
Wales	ILD	age_strat	01/01/2005		50 to 59	102
Wales	ILD	age_strat	01/01/2006		50 to 59	94
Wales	ILD	age_strat	01/01/2007		50 to 59	87
Wales	ILD	age_strat	01/01/2008		50 to 59	84
Wales	ILD	age_strat	01/01/2009		50 to 59	69
Wales	ILD	age_strat	01/01/2010		50 to 59	75
Wales	ILD	age_strat	01/01/2011		50 to 59	79
Wales	ILD	age_strat	01/01/2012		50 to 59	78
Wales	ILD	age_strat	01/01/2013		50 to 59	94
Wales	ILD	age_strat	01/01/2014		50 to 59	110
Wales	ILD	age_strat	01/01/2015		50 to 59	91
Wales	ILD	age_strat	01/01/2016		50 to 59	99
Wales	ILD	age_strat	01/01/2017		50 to 59	87
Wales	ILD	age_strat	01/01/2018		50 to 59	109
Wales	ILD	age_strat	01/01/2019		50 to 59	106
Wales	ILD	age_strat	01/01/2004		60 to 69	227
Wales	ILD	age_strat	01/01/2005		60 to 69	207
Wales	ILD	age_strat	01/01/2006		60 to 69	181
Wales	ILD	age_strat	01/01/2007		60 to 69	201
Wales	ILD	age_strat	01/01/2008		60 to 69	197
Wales	ILD	age_strat	01/01/2009		60 to 69	173
Wales	ILD	age_strat	01/01/2010		60 to 69	167
Wales	ILD	age_strat	01/01/2011		60 to 69	206
Wales	ILD	age_strat	01/01/2012		60 to 69	193
Wales	ILD	age_strat	01/01/2013		60 to 69	196
Wales	ILD	age_strat	01/01/2014		60 to 69	204
Wales	ILD	age_strat	01/01/2015		60 to 69	206
Wales	ILD	age_strat	01/01/2016		60 to 69	179
Wales	ILD	age_strat	01/01/2017		60 to 69	224
Wales	ILD	age_strat	01/01/2018		60 to 69	203
Wales	ILD	age_strat	01/01/2019		60 to 69	225
Wales	ILD	age_strat	01/01/2004		70plus	431
Wales	ILD	age_strat	01/01/2005		70plus	388
Wales	ILD	age_strat	01/01/2006		70plus	411
Wales	ILD	age_strat	01/01/2007		70plus	401
Wales	ILD	age_strat	01/01/2008		70plus	432
Wales	ILD	age_strat	01/01/2009		70plus	428
Wales	ILD	age_strat	01/01/2010		70plus	490
Wales	ILD	age_strat	01/01/2011		70plus	476
Wales	ILD	age_strat	01/01/2012		70plus	508
Wales	ILD	age_strat	01/01/2013		70plus	506
Wales	ILD	age_strat	01/01/2014		70plus	503
Wales	ILD	age_strat	01/01/2015		70plus	625
Wales	ILD	age_strat	01/01/2016		70plus	646
Wales	ILD	age_strat	01/01/2017		70plus	711
Wales	ILD	age_strat	01/01/2018		70plus	728
Wales	ILD	age_strat	01/01/2019		70plus	782
Wales	Asthma	sex_strat	01/01/2004	Female		10581
Wales	Asthma	sex_strat	01/01/2005	Female		9431
Wales	Asthma	sex_strat	01/01/2006	Female		7432
Wales	Asthma	sex_strat	01/01/2007	Female		6372
Wales	Asthma	sex_strat	01/01/2008	Female		6346
Wales	Asthma	sex_strat	01/01/2009	Female		6262

Wales	Asthma	sex_strat	01/01/2010	Female	5972
Wales	Asthma	sex_strat	01/01/2011	Female	5894
Wales	Asthma	sex_strat	01/01/2012	Female	6376
Wales	Asthma	sex_strat	01/01/2013	Female	5682
Wales	Asthma	sex_strat	01/01/2014	Female	5935
Wales	Asthma	sex_strat	01/01/2015	Female	8200
Wales	Asthma	sex_strat	01/01/2016	Female	6150
Wales	Asthma	sex_strat	01/01/2017	Female	6108
Wales	Asthma	sex_strat	01/01/2018	Female	5659
Wales	Asthma	sex_strat	01/01/2019	Female	6031
Wales	Asthma	sex_strat	01/01/2004	Male	9087
Wales	Asthma	sex_strat	01/01/2005	Male	8032
Wales	Asthma	sex_strat	01/01/2006	Male	6533
Wales	Asthma	sex_strat	01/01/2007	Male	5831
Wales	Asthma	sex_strat	01/01/2008	Male	5743
Wales	Asthma	sex_strat	01/01/2009	Male	5928
Wales	Asthma	sex_strat	01/01/2010	Male	5443
Wales	Asthma	sex_strat	01/01/2011	Male	5405
Wales	Asthma	sex_strat	01/01/2012	Male	5980
Wales	Asthma	sex_strat	01/01/2013	Male	5098
Wales	Asthma	sex_strat	01/01/2014	Male	5224
Wales	Asthma	sex_strat	01/01/2015	Male	7326
Wales	Asthma	sex_strat	01/01/2016	Male	5284
Wales	Asthma	sex_strat	01/01/2017	Male	5058
Wales	Asthma	sex_strat	01/01/2018	Male	5018
Wales	Asthma	sex_strat	01/01/2019	Male	5153
Wales	COPD	sex_strat	01/01/2004	Female	3872
Wales	COPD	sex_strat	01/01/2005	Female	4526
Wales	COPD	sex_strat	01/01/2006	Female	4183
Wales	COPD	sex_strat	01/01/2007	Female	4069
Wales	COPD	sex_strat	01/01/2008	Female	3578
Wales	COPD	sex_strat	01/01/2009	Female	4141
Wales	COPD	sex_strat	01/01/2010	Female	3767
Wales	COPD	sex_strat	01/01/2011	Female	3594
Wales	COPD	sex_strat	01/01/2012	Female	3506
Wales	COPD	sex_strat	01/01/2013	Female	3196
Wales	COPD	sex_strat	01/01/2014	Female	3208
Wales	COPD	sex_strat	01/01/2015	Female	3300
Wales	COPD	sex_strat	01/01/2016	Female	3547
Wales	COPD	sex_strat	01/01/2017	Female	3395
Wales	COPD	sex_strat	01/01/2018	Female	3428
Wales	COPD	sex_strat	01/01/2019	Female	3313
Wales	COPD	sex_strat	01/01/2004	Male	4540
Wales	COPD	sex_strat	01/01/2005	Male	4981
Wales	COPD	sex_strat	01/01/2006	Male	4639
Wales	COPD	sex_strat	01/01/2007	Male	4209
Wales	COPD	sex_strat	01/01/2008	Male	3812
Wales	COPD	sex_strat	01/01/2009	Male	4356
Wales	COPD	sex_strat	01/01/2010	Male	3908
Wales	COPD	sex_strat	01/01/2011	Male	3615
Wales	COPD	sex_strat	01/01/2012	Male	3530
Wales	COPD	sex_strat	01/01/2013	Male	3489
Wales	COPD	sex_strat	01/01/2014	Male	3278
Wales	COPD	sex_strat	01/01/2015	Male	3397
Wales	COPD	sex_strat	01/01/2016	Male	3501
Wales	COPD	sex_strat	01/01/2017	Male	3510
Wales	COPD	sex_strat	01/01/2018	Male	3384
Wales	COPD	sex_strat	01/01/2019	Male	3394
Wales	ILD	sex_strat	01/01/2004	Female	246
Wales	ILD	sex_strat	01/01/2005	Female	246
Wales	ILD	sex_strat	01/01/2006	Female	218
Wales	ILD	sex_strat	01/01/2007	Female	261
Wales	ILD	sex_strat	01/01/2008	Female	247

Wales	ILD	sex_strat	01/01/2009	Female	256
Wales	ILD	sex_strat	01/01/2010	Female	271
Wales	ILD	sex_strat	01/01/2011	Female	301
Wales	ILD	sex_strat	01/01/2012	Female	283
Wales	ILD	sex_strat	01/01/2013	Female	300
Wales	ILD	sex_strat	01/01/2014	Female	307
Wales	ILD	sex_strat	01/01/2015	Female	316
Wales	ILD	sex_strat	01/01/2016	Female	373
Wales	ILD	sex_strat	01/01/2017	Female	373
Wales	ILD	sex_strat	01/01/2018	Female	380
Wales	ILD	sex_strat	01/01/2019	Female	422
Wales	ILD	sex_strat	01/01/2004	Male	552
Wales	ILD	sex_strat	01/01/2005	Male	499
Wales	ILD	sex_strat	01/01/2006	Male	514
Wales	ILD	sex_strat	01/01/2007	Male	476
Wales	ILD	sex_strat	01/01/2008	Male	502
Wales	ILD	sex_strat	01/01/2009	Male	447
Wales	ILD	sex_strat	01/01/2010	Male	506
Wales	ILD	sex_strat	01/01/2011	Male	506
Wales	ILD	sex_strat	01/01/2012	Male	548
Wales	ILD	sex_strat	01/01/2013	Male	527
Wales	ILD	sex_strat	01/01/2014	Male	553
Wales	ILD	sex_strat	01/01/2015	Male	636
Wales	ILD	sex_strat	01/01/2016	Male	594
Wales	ILD	sex_strat	01/01/2017	Male	686
Wales	ILD	sex_strat	01/01/2018	Male	699
Wales	ILD	sex_strat	01/01/2019	Male	733
England_CPRD	Asthma	all	01/01/2004		60430
England_CPRD	Asthma	all	01/01/2005		53214
England_CPRD	Asthma	all	01/01/2006		56359
England_CPRD	Asthma	all	01/01/2007		51739
England_CPRD	Asthma	all	01/01/2008		52781
England_CPRD	Asthma	all	01/01/2009		52434
England_CPRD	Asthma	all	01/01/2010		49855
England_CPRD	Asthma	all	01/01/2011		48675
England_CPRD	Asthma	all	01/01/2012		53136
England_CPRD	Asthma	all	01/01/2013		50670
England_CPRD	Asthma	all	01/01/2014		53536
England_CPRD	Asthma	all	01/01/2015		52150
England_CPRD	Asthma	all	01/01/2016		54773
England_CPRD	Asthma	all	01/01/2017		54930
England_CPRD	Asthma	all	01/01/2018		52588
England_CPRD	Asthma	all	01/01/2019		58222
England_CPRD	COPD	all	01/01/2004		19896
England_CPRD	COPD	all	01/01/2005		24701
England_CPRD	COPD	all	01/01/2006		23560
England_CPRD	COPD	all	01/01/2007		22286
England_CPRD	COPD	all	01/01/2008		22044
England_CPRD	COPD	all	01/01/2009		23552
England_CPRD	COPD	all	01/01/2010		23498
England_CPRD	COPD	all	01/01/2011		23698
England_CPRD	COPD	all	01/01/2012		23535
England_CPRD	COPD	all	01/01/2013		23756
England_CPRD	COPD	all	01/01/2014		21921
England_CPRD	COPD	all	01/01/2015		23353
England_CPRD	COPD	all	01/01/2016		25233
England_CPRD	COPD	all	01/01/2017		24358
England_CPRD	COPD	all	01/01/2018		24035
England_CPRD	COPD	all	01/01/2019		25162
England_CPRD	ILD	all	01/01/2004		865
England_CPRD	ILD	all	01/01/2005		1022
England_CPRD	ILD	all	01/01/2006		2452
England_CPRD	ILD	all	01/01/2007		2593

England_CPRD	ILD	all	01/01/2008		2610
England_CPRD	ILD	all	01/01/2009		2832
England_CPRD	ILD	all	01/01/2010		2782
England_CPRD	ILD	all	01/01/2011		2888
England_CPRD	ILD	all	01/01/2012		3134
England_CPRD	ILD	all	01/01/2013		3356
England_CPRD	ILD	all	01/01/2014		3392
England_CPRD	ILD	all	01/01/2015		3604
England_CPRD	ILD	all	01/01/2016		3837
England_CPRD	ILD	all	01/01/2017		4110
England_CPRD	ILD	all	01/01/2018		4325
England_CPRD	ILD	all	01/01/2019		4224
England_CPRD	Asthma	sex_strat_children	01/01/2004	Male	8957
England_CPRD	Asthma	sex_strat_children	01/01/2004	Female	6727
England_CPRD	Asthma	sex_strat_children	01/01/2005	Male	8763
England_CPRD	Asthma	sex_strat_children	01/01/2005	Female	6756
England_CPRD	Asthma	sex_strat_children	01/01/2006	Male	9986
England_CPRD	Asthma	sex_strat_children	01/01/2006	Female	7764
England_CPRD	Asthma	sex_strat_children	01/01/2007	Male	9545
England_CPRD	Asthma	sex_strat_children	01/01/2007	Female	7330
England_CPRD	Asthma	sex_strat_children	01/01/2008	Male	9760
England_CPRD	Asthma	sex_strat_children	01/01/2008	Female	7472
England_CPRD	Asthma	sex_strat_children	01/01/2009	Male	9957
England_CPRD	Asthma	sex_strat_children	01/01/2009	Female	7689
England_CPRD	Asthma	sex_strat_children	01/01/2010	Male	9386
England_CPRD	Asthma	sex_strat_children	01/01/2010	Female	7441
England_CPRD	Asthma	sex_strat_children	01/01/2011	Male	9653
England_CPRD	Asthma	sex_strat_children	01/01/2011	Female	7638
England_CPRD	Asthma	sex_strat_children	01/01/2012	Male	10839
England_CPRD	Asthma	sex_strat_children	01/01/2012	Female	8504
England_CPRD	Asthma	sex_strat_children	01/01/2013	Male	9892
England_CPRD	Asthma	sex_strat_children	01/01/2013	Female	7722
England_CPRD	Asthma	sex_strat_children	01/01/2014	Male	10517
England_CPRD	Asthma	sex_strat_children	01/01/2014	Female	8526
England_CPRD	Asthma	sex_strat_children	01/01/2015	Male	10239
England_CPRD	Asthma	sex_strat_children	01/01/2015	Female	8434
England_CPRD	Asthma	sex_strat_children	01/01/2016	Male	10453
England_CPRD	Asthma	sex_strat_children	01/01/2016	Female	8656
England_CPRD	Asthma	sex_strat_children	01/01/2017	Male	10156
England_CPRD	Asthma	sex_strat_children	01/01/2017	Female	8646
England_CPRD	Asthma	sex_strat_children	01/01/2018	Male	9224
England_CPRD	Asthma	sex_strat_children	01/01/2018	Female	7782
England_CPRD	Asthma	sex_strat_children	01/01/2019	Male	9845
England_CPRD	Asthma	sex_strat_children	01/01/2019	Female	8278
England_CPRD	Asthma	sex_strat_adults	01/01/2004	Male	18975
England_CPRD	Asthma	sex_strat_adults	01/01/2004	Female	25771
England_CPRD	Asthma	sex_strat_adults	01/01/2005	Male	15689
England_CPRD	Asthma	sex_strat_adults	01/01/2005	Female	22006
England_CPRD	Asthma	sex_strat_adults	01/01/2006	Male	16377
England_CPRD	Asthma	sex_strat_adults	01/01/2006	Female	22232
England_CPRD	Asthma	sex_strat_adults	01/01/2007	Male	14892
England_CPRD	Asthma	sex_strat_adults	01/01/2007	Female	19972
England_CPRD	Asthma	sex_strat_adults	01/01/2008	Male	15245
England_CPRD	Asthma	sex_strat_adults	01/01/2008	Female	20304
England_CPRD	Asthma	sex_strat_adults	01/01/2009	Male	14778
England_CPRD	Asthma	sex_strat_adults	01/01/2009	Female	20010
England_CPRD	Asthma	sex_strat_adults	01/01/2010	Male	13930
England_CPRD	Asthma	sex_strat_adults	01/01/2010	Female	19098
England_CPRD	Asthma	sex_strat_adults	01/01/2011	Male	13049
England_CPRD	Asthma	sex_strat_adults	01/01/2011	Female	18335
England_CPRD	Asthma	sex_strat_adults	01/01/2012	Male	14211
England_CPRD	Asthma	sex_strat_adults	01/01/2012	Female	19582
England_CPRD	Asthma	sex_strat_adults	01/01/2013	Male	13794

England_CPRD	Asthma	sex_strat_adults	01/01/2013	Female	19262
England_CPRD	Asthma	sex_strat_adults	01/01/2014	Male	14168
England_CPRD	Asthma	sex_strat_adults	01/01/2014	Female	20325
England_CPRD	Asthma	sex_strat_adults	01/01/2015	Male	13731
England_CPRD	Asthma	sex_strat_adults	01/01/2015	Female	19746
England_CPRD	Asthma	sex_strat_adults	01/01/2016	Male	14914
England_CPRD	Asthma	sex_strat_adults	01/01/2016	Female	20750
England_CPRD	Asthma	sex_strat_adults	01/01/2017	Male	14775
England_CPRD	Asthma	sex_strat_adults	01/01/2017	Female	21353
England_CPRD	Asthma	sex_strat_adults	01/01/2018	Male	14893
England_CPRD	Asthma	sex_strat_adults	01/01/2018	Female	20689
England_CPRD	Asthma	sex_strat_adults	01/01/2019	Male	16869
England_CPRD	Asthma	sex_strat_adults	01/01/2019	Female	23230
England_CPRD	COPD	sex_strat	01/01/2004	Male	10576
England_CPRD	COPD	sex_strat	01/01/2004	Female	9320
England_CPRD	COPD	sex_strat	01/01/2005	Male	13138
England_CPRD	COPD	sex_strat	01/01/2005	Female	11563
England_CPRD	COPD	sex_strat	01/01/2006	Male	12360
England_CPRD	COPD	sex_strat	01/01/2006	Female	11200
England_CPRD	COPD	sex_strat	01/01/2007	Male	11749
England_CPRD	COPD	sex_strat	01/01/2007	Female	10537
England_CPRD	COPD	sex_strat	01/01/2008	Male	11480
England_CPRD	COPD	sex_strat	01/01/2008	Female	10564
England_CPRD	COPD	sex_strat	01/01/2009	Male	12365
England_CPRD	COPD	sex_strat	01/01/2009	Female	11187
England_CPRD	COPD	sex_strat	01/01/2010	Male	12391
England_CPRD	COPD	sex_strat	01/01/2010	Female	11107
England_CPRD	COPD	sex_strat	01/01/2011	Male	12418
England_CPRD	COPD	sex_strat	01/01/2011	Female	11280
England_CPRD	COPD	sex_strat	01/01/2012	Male	12470
England_CPRD	COPD	sex_strat	01/01/2012	Female	11065
England_CPRD	COPD	sex_strat	01/01/2013	Male	12563
England_CPRD	COPD	sex_strat	01/01/2013	Female	11193
England_CPRD	COPD	sex_strat	01/01/2014	Male	11525
England_CPRD	COPD	sex_strat	01/01/2014	Female	10396
England_CPRD	COPD	sex_strat	01/01/2015	Male	12416
England_CPRD	COPD	sex_strat	01/01/2015	Female	10937
England_CPRD	COPD	sex_strat	01/01/2016	Male	13295
England_CPRD	COPD	sex_strat	01/01/2016	Female	11938
England_CPRD	COPD	sex_strat	01/01/2017	Male	12796
England_CPRD	COPD	sex_strat	01/01/2017	Female	11562
England_CPRD	COPD	sex_strat	01/01/2018	Male	12820
England_CPRD	COPD	sex_strat	01/01/2018	Female	11215
England_CPRD	COPD	sex_strat	01/01/2019	Male	13315
England_CPRD	COPD	sex_strat	01/01/2019	Female	11847
England_CPRD	ILD	sex_strat	01/01/2004	Male	562
England_CPRD	ILD	sex_strat	01/01/2004	Female	303
England_CPRD	ILD	sex_strat	01/01/2005	Male	695
England_CPRD	ILD	sex_strat	01/01/2005	Female	327
England_CPRD	ILD	sex_strat	01/01/2006	Male	1629
England_CPRD	ILD	sex_strat	01/01/2006	Female	823
England_CPRD	ILD	sex_strat	01/01/2007	Male	1706
England_CPRD	ILD	sex_strat	01/01/2007	Female	887
England_CPRD	ILD	sex_strat	01/01/2008	Male	1737
England_CPRD	ILD	sex_strat	01/01/2008	Female	873
England_CPRD	ILD	sex_strat	01/01/2009	Male	1885
England_CPRD	ILD	sex_strat	01/01/2009	Female	947
England_CPRD	ILD	sex_strat	01/01/2010	Male	1785
England_CPRD	ILD	sex_strat	01/01/2010	Female	997
England_CPRD	ILD	sex_strat	01/01/2011	Male	1855
England_CPRD	ILD	sex_strat	01/01/2011	Female	1033
England_CPRD	ILD	sex_strat	01/01/2012	Male	1924
England_CPRD	ILD	sex_strat	01/01/2012	Female	1210

England_CPRD	ILD	sex_strat	01/01/2013	Male		2103
England_CPRD	ILD	sex_strat	01/01/2013	Female		1253
England_CPRD	ILD	sex_strat	01/01/2014	Male		2136
England_CPRD	ILD	sex_strat	01/01/2014	Female		1256
England_CPRD	ILD	sex_strat	01/01/2015	Male		2284
England_CPRD	ILD	sex_strat	01/01/2015	Female		1320
England_CPRD	ILD	sex_strat	01/01/2016	Male		2432
England_CPRD	ILD	sex_strat	01/01/2016	Female		1405
England_CPRD	ILD	sex_strat	01/01/2017	Male		2613
England_CPRD	ILD	sex_strat	01/01/2017	Female		1497
England_CPRD	ILD	sex_strat	01/01/2018	Male		2728
England_CPRD	ILD	sex_strat	01/01/2018	Female		1597
England_CPRD	ILD	sex_strat	01/01/2019	Male		2620
England_CPRD	ILD	sex_strat	01/01/2019	Female		1604
England_CPRD	Asthma	age_strat	01/01/2004		0-4	5291
England_CPRD	Asthma	age_strat	01/01/2004		05-Sep	4706
England_CPRD	Asthma	age_strat	01/01/2004		Oct-14	3533
England_CPRD	Asthma	age_strat	01/01/2004		15-19	2154
England_CPRD	Asthma	age_strat	01/01/2004		20-29	5639
England_CPRD	Asthma	age_strat	01/01/2004		30-39	9008
England_CPRD	Asthma	age_strat	01/01/2004		40-49	8433
England_CPRD	Asthma	age_strat	01/01/2004		50-59	7495
England_CPRD	Asthma	age_strat	01/01/2004		60-69	7029
England_CPRD	Asthma	age_strat	01/01/2004		70+	7142
England_CPRD	Asthma	age_strat	01/01/2005		0-4	5295
England_CPRD	Asthma	age_strat	01/01/2005		05-Sep	4696
England_CPRD	Asthma	age_strat	01/01/2005		Oct-14	3444
England_CPRD	Asthma	age_strat	01/01/2005		15-19	2084
England_CPRD	Asthma	age_strat	01/01/2005		20-29	5457
England_CPRD	Asthma	age_strat	01/01/2005		30-39	7720
England_CPRD	Asthma	age_strat	01/01/2005		40-49	7447
England_CPRD	Asthma	age_strat	01/01/2005		50-59	6156
England_CPRD	Asthma	age_strat	01/01/2005		60-69	5486
England_CPRD	Asthma	age_strat	01/01/2005		70+	5429
England_CPRD	Asthma	age_strat	01/01/2006		0-4	6227
England_CPRD	Asthma	age_strat	01/01/2006		05-Sep	5166
England_CPRD	Asthma	age_strat	01/01/2006		Oct-14	3878
England_CPRD	Asthma	age_strat	01/01/2006		15-19	2479
England_CPRD	Asthma	age_strat	01/01/2006		20-29	6546
England_CPRD	Asthma	age_strat	01/01/2006		30-39	7454
England_CPRD	Asthma	age_strat	01/01/2006		40-49	6988
England_CPRD	Asthma	age_strat	01/01/2006		50-59	5617
England_CPRD	Asthma	age_strat	01/01/2006		60-69	5591
England_CPRD	Asthma	age_strat	01/01/2006		70+	6413
England_CPRD	Asthma	age_strat	01/01/2007		0-4	6041
England_CPRD	Asthma	age_strat	01/01/2007		05-Sep	4984
England_CPRD	Asthma	age_strat	01/01/2007		Oct-14	3628
England_CPRD	Asthma	age_strat	01/01/2007		15-19	2222
England_CPRD	Asthma	age_strat	01/01/2007		20-29	6245
England_CPRD	Asthma	age_strat	01/01/2007		30-39	6743
England_CPRD	Asthma	age_strat	01/01/2007		40-49	6518
England_CPRD	Asthma	age_strat	01/01/2007		50-59	5139
England_CPRD	Asthma	age_strat	01/01/2007		60-69	4802
England_CPRD	Asthma	age_strat	01/01/2007		70+	5417
England_CPRD	Asthma	age_strat	01/01/2008		0-4	5987
England_CPRD	Asthma	age_strat	01/01/2008		05-Sep	5121
England_CPRD	Asthma	age_strat	01/01/2008		Oct-14	3783
England_CPRD	Asthma	age_strat	01/01/2008		15-19	2341
England_CPRD	Asthma	age_strat	01/01/2008		20-29	6590
England_CPRD	Asthma	age_strat	01/01/2008		30-39	7301
England_CPRD	Asthma	age_strat	01/01/2008		40-49	6649
England_CPRD	Asthma	age_strat	01/01/2008		50-59	5019
England_CPRD	Asthma	age_strat	01/01/2008		60-69	4875

England_CPRD	Asthma	age_strat	01/01/2008	70+	5115
England_CPRD	Asthma	age_strat	01/01/2009	0-4	5933
England_CPRD	Asthma	age_strat	01/01/2009	05-Sep	5829
England_CPRD	Asthma	age_strat	01/01/2009	Oct-14	3567
England_CPRD	Asthma	age_strat	01/01/2009	15-19	2317
England_CPRD	Asthma	age_strat	01/01/2009	20-29	6577
England_CPRD	Asthma	age_strat	01/01/2009	30-39	7127
England_CPRD	Asthma	age_strat	01/01/2009	40-49	6655
England_CPRD	Asthma	age_strat	01/01/2009	50-59	4855
England_CPRD	Asthma	age_strat	01/01/2009	60-69	4739
England_CPRD	Asthma	age_strat	01/01/2009	70+	4835
England_CPRD	Asthma	age_strat	01/01/2010	0-4	5565
England_CPRD	Asthma	age_strat	01/01/2010	05-Sep	5624
England_CPRD	Asthma	age_strat	01/01/2010	Oct-14	3392
England_CPRD	Asthma	age_strat	01/01/2010	15-19	2246
England_CPRD	Asthma	age_strat	01/01/2010	20-29	6192
England_CPRD	Asthma	age_strat	01/01/2010	30-39	6640
England_CPRD	Asthma	age_strat	01/01/2010	40-49	6454
England_CPRD	Asthma	age_strat	01/01/2010	50-59	4771
England_CPRD	Asthma	age_strat	01/01/2010	60-69	4511
England_CPRD	Asthma	age_strat	01/01/2010	70+	4460
England_CPRD	Asthma	age_strat	01/01/2011	0-4	5915
England_CPRD	Asthma	age_strat	01/01/2011	05-Sep	5833
England_CPRD	Asthma	age_strat	01/01/2011	Oct-14	3500
England_CPRD	Asthma	age_strat	01/01/2011	15-19	2043
England_CPRD	Asthma	age_strat	01/01/2011	20-29	6007
England_CPRD	Asthma	age_strat	01/01/2011	30-39	6335
England_CPRD	Asthma	age_strat	01/01/2011	40-49	6067
England_CPRD	Asthma	age_strat	01/01/2011	50-59	4649
England_CPRD	Asthma	age_strat	01/01/2011	60-69	4168
England_CPRD	Asthma	age_strat	01/01/2011	70+	4158
England_CPRD	Asthma	age_strat	01/01/2012	0-4	6184
England_CPRD	Asthma	age_strat	01/01/2012	05-Sep	6758
England_CPRD	Asthma	age_strat	01/01/2012	Oct-14	4056
England_CPRD	Asthma	age_strat	01/01/2012	15-19	2345
England_CPRD	Asthma	age_strat	01/01/2012	20-29	6413
England_CPRD	Asthma	age_strat	01/01/2012	30-39	6989
England_CPRD	Asthma	age_strat	01/01/2012	40-49	6476
England_CPRD	Asthma	age_strat	01/01/2012	50-59	4952
England_CPRD	Asthma	age_strat	01/01/2012	60-69	4587
England_CPRD	Asthma	age_strat	01/01/2012	70+	4376
England_CPRD	Asthma	age_strat	01/01/2013	0-4	5547
England_CPRD	Asthma	age_strat	01/01/2013	05-Sep	6168
England_CPRD	Asthma	age_strat	01/01/2013	Oct-14	3820
England_CPRD	Asthma	age_strat	01/01/2013	15-19	2079
England_CPRD	Asthma	age_strat	01/01/2013	20-29	6544
England_CPRD	Asthma	age_strat	01/01/2013	30-39	6554
England_CPRD	Asthma	age_strat	01/01/2013	40-49	6321
England_CPRD	Asthma	age_strat	01/01/2013	50-59	4971
England_CPRD	Asthma	age_strat	01/01/2013	60-69	4355
England_CPRD	Asthma	age_strat	01/01/2013	70+	4311
England_CPRD	Asthma	age_strat	01/01/2014	0-4	5454
England_CPRD	Asthma	age_strat	01/01/2014	05-Sep	6958
England_CPRD	Asthma	age_strat	01/01/2014	Oct-14	4291
England_CPRD	Asthma	age_strat	01/01/2014	15-19	2340
England_CPRD	Asthma	age_strat	01/01/2014	20-29	6884
England_CPRD	Asthma	age_strat	01/01/2014	30-39	7205
England_CPRD	Asthma	age_strat	01/01/2014	40-49	6572
England_CPRD	Asthma	age_strat	01/01/2014	50-59	5298
England_CPRD	Asthma	age_strat	01/01/2014	60-69	4345
England_CPRD	Asthma	age_strat	01/01/2014	70+	4189
England_CPRD	Asthma	age_strat	01/01/2015	0-4	5170
England_CPRD	Asthma	age_strat	01/01/2015	05-Sep	6821

England_CPRD	Asthma	age_strat	01/01/2015	Oct-14	4375
England_CPRD	Asthma	age_strat	01/01/2015	15-19	2307
England_CPRD	Asthma	age_strat	01/01/2015	20-29	6472
England_CPRD	Asthma	age_strat	01/01/2015	30-39	6822
England_CPRD	Asthma	age_strat	01/01/2015	40-49	6363
England_CPRD	Asthma	age_strat	01/01/2015	50-59	5268
England_CPRD	Asthma	age_strat	01/01/2015	60-69	4264
England_CPRD	Asthma	age_strat	01/01/2015	70+	4288
England_CPRD	Asthma	age_strat	01/01/2016	0-4	4837
England_CPRD	Asthma	age_strat	01/01/2016	05-Sep	7203
England_CPRD	Asthma	age_strat	01/01/2016	Oct-14	4777
England_CPRD	Asthma	age_strat	01/01/2016	15-19	2292
England_CPRD	Asthma	age_strat	01/01/2016	20-29	7190
England_CPRD	Asthma	age_strat	01/01/2016	30-39	7409
England_CPRD	Asthma	age_strat	01/01/2016	40-49	6595
England_CPRD	Asthma	age_strat	01/01/2016	50-59	5658
England_CPRD	Asthma	age_strat	01/01/2016	60-69	4488
England_CPRD	Asthma	age_strat	01/01/2016	70+	4324
England_CPRD	Asthma	age_strat	01/01/2017	0-4	4522
England_CPRD	Asthma	age_strat	01/01/2017	05-Sep	7202
England_CPRD	Asthma	age_strat	01/01/2017	Oct-14	4777
England_CPRD	Asthma	age_strat	01/01/2017	15-19	2301
England_CPRD	Asthma	age_strat	01/01/2017	20-29	6790
England_CPRD	Asthma	age_strat	01/01/2017	30-39	7303
England_CPRD	Asthma	age_strat	01/01/2017	40-49	6592
England_CPRD	Asthma	age_strat	01/01/2017	50-59	5998
England_CPRD	Asthma	age_strat	01/01/2017	60-69	4629
England_CPRD	Asthma	age_strat	01/01/2017	70+	4816
England_CPRD	Asthma	age_strat	01/01/2018	0-4	3956
England_CPRD	Asthma	age_strat	01/01/2018	05-Sep	6378
England_CPRD	Asthma	age_strat	01/01/2018	Oct-14	4383
England_CPRD	Asthma	age_strat	01/01/2018	15-19	2289
England_CPRD	Asthma	age_strat	01/01/2018	20-29	6664
England_CPRD	Asthma	age_strat	01/01/2018	30-39	7072
England_CPRD	Asthma	age_strat	01/01/2018	40-49	6340
England_CPRD	Asthma	age_strat	01/01/2018	50-59	6040
England_CPRD	Asthma	age_strat	01/01/2018	60-69	4641
England_CPRD	Asthma	age_strat	01/01/2018	70+	4825
England_CPRD	Asthma	age_strat	01/01/2019	0-4	3812
England_CPRD	Asthma	age_strat	01/01/2019	05-Sep	7005
England_CPRD	Asthma	age_strat	01/01/2019	Oct-14	4862
England_CPRD	Asthma	age_strat	01/01/2019	15-19	2444
England_CPRD	Asthma	age_strat	01/01/2019	20-29	6924
England_CPRD	Asthma	age_strat	01/01/2019	30-39	7584
England_CPRD	Asthma	age_strat	01/01/2019	40-49	6788
England_CPRD	Asthma	age_strat	01/01/2019	50-59	6810
England_CPRD	Asthma	age_strat	01/01/2019	60-69	5436
England_CPRD	Asthma	age_strat	01/01/2019	70+	6557
England_CPRD	COPD	age_strat	01/01/2004	40-49	1120
England_CPRD	COPD	age_strat	01/01/2004	50-59	3600
England_CPRD	COPD	age_strat	01/01/2004	60-69	6103
England_CPRD	COPD	age_strat	01/01/2004	70+	9073
England_CPRD	COPD	age_strat	01/01/2005	40-49	1496
England_CPRD	COPD	age_strat	01/01/2005	50-59	4330
England_CPRD	COPD	age_strat	01/01/2005	60-69	7226
England_CPRD	COPD	age_strat	01/01/2005	70+	11649
England_CPRD	COPD	age_strat	01/01/2006	40-49	1553
England_CPRD	COPD	age_strat	01/01/2006	50-59	4233
England_CPRD	COPD	age_strat	01/01/2006	60-69	7022
England_CPRD	COPD	age_strat	01/01/2006	70+	10752
England_CPRD	COPD	age_strat	01/01/2007	40-49	1434
England_CPRD	COPD	age_strat	01/01/2007	50-59	3872
England_CPRD	COPD	age_strat	01/01/2007	60-69	6795

England_CPRD	COPD	age_strat	01/01/2007	70+	10185
England_CPRD	COPD	age_strat	01/01/2008	40-49	1526
England_CPRD	COPD	age_strat	01/01/2008	50-59	4000
England_CPRD	COPD	age_strat	01/01/2008	60-69	6671
England_CPRD	COPD	age_strat	01/01/2008	70+	9847
England_CPRD	COPD	age_strat	01/01/2009	40-49	1843
England_CPRD	COPD	age_strat	01/01/2009	50-59	4252
England_CPRD	COPD	age_strat	01/01/2009	60-69	7109
England_CPRD	COPD	age_strat	01/01/2009	70+	10348
England_CPRD	COPD	age_strat	01/01/2010	40-49	1917
England_CPRD	COPD	age_strat	01/01/2010	50-59	4315
England_CPRD	COPD	age_strat	01/01/2010	60-69	7423
England_CPRD	COPD	age_strat	01/01/2010	70+	9843
England_CPRD	COPD	age_strat	01/01/2011	40-49	1859
England_CPRD	COPD	age_strat	01/01/2011	50-59	4564
England_CPRD	COPD	age_strat	01/01/2011	60-69	7288
England_CPRD	COPD	age_strat	01/01/2011	70+	9987
England_CPRD	COPD	age_strat	01/01/2012	40-49	2002
England_CPRD	COPD	age_strat	01/01/2012	50-59	4634
England_CPRD	COPD	age_strat	01/01/2012	60-69	7167
England_CPRD	COPD	age_strat	01/01/2012	70+	9732
England_CPRD	COPD	age_strat	01/01/2013	40-49	2031
England_CPRD	COPD	age_strat	01/01/2013	50-59	4546
England_CPRD	COPD	age_strat	01/01/2013	60-69	7247
England_CPRD	COPD	age_strat	01/01/2013	70+	9932
England_CPRD	COPD	age_strat	01/01/2014	40-49	1829
England_CPRD	COPD	age_strat	01/01/2014	50-59	4309
England_CPRD	COPD	age_strat	01/01/2014	60-69	6604
England_CPRD	COPD	age_strat	01/01/2014	70+	9179
England_CPRD	COPD	age_strat	01/01/2015	40-49	1874
England_CPRD	COPD	age_strat	01/01/2015	50-59	4504
England_CPRD	COPD	age_strat	01/01/2015	60-69	6929
England_CPRD	COPD	age_strat	01/01/2015	70+	10046
England_CPRD	COPD	age_strat	01/01/2016	40-49	2291
England_CPRD	COPD	age_strat	01/01/2016	50-59	5004
England_CPRD	COPD	age_strat	01/01/2016	60-69	7485
England_CPRD	COPD	age_strat	01/01/2016	70+	10453
England_CPRD	COPD	age_strat	01/01/2017	40-49	1908
England_CPRD	COPD	age_strat	01/01/2017	50-59	4890
England_CPRD	COPD	age_strat	01/01/2017	60-69	6958
England_CPRD	COPD	age_strat	01/01/2017	70+	10602
England_CPRD	COPD	age_strat	01/01/2018	40-49	1786
England_CPRD	COPD	age_strat	01/01/2018	50-59	4913
England_CPRD	COPD	age_strat	01/01/2018	60-69	6733
England_CPRD	COPD	age_strat	01/01/2018	70+	10603
England_CPRD	COPD	age_strat	01/01/2019	40-49	2826
England_CPRD	COPD	age_strat	01/01/2019	50-59	5303
England_CPRD	COPD	age_strat	01/01/2019	60-69	6863
England_CPRD	COPD	age_strat	01/01/2019	70+	10170
England_CPRD	ILD	age_strat	01/01/2004	40-49	67
England_CPRD	ILD	age_strat	01/01/2004	50-59	145
England_CPRD	ILD	age_strat	01/01/2004	60-69	280
England_CPRD	ILD	age_strat	01/01/2004	70+	373
England_CPRD	ILD	age_strat	01/01/2005	40-49	76
England_CPRD	ILD	age_strat	01/01/2005	50-59	150
England_CPRD	ILD	age_strat	01/01/2005	60-69	259
England_CPRD	ILD	age_strat	01/01/2005	70+	537
England_CPRD	ILD	age_strat	01/01/2006	40-49	154
England_CPRD	ILD	age_strat	01/01/2006	50-59	261
England_CPRD	ILD	age_strat	01/01/2006	60-69	642
England_CPRD	ILD	age_strat	01/01/2006	70+	1395
England_CPRD	ILD	age_strat	01/01/2007	40-49	139
England_CPRD	ILD	age_strat	01/01/2007	50-59	286

England_CPRD	ILD	age_strat	01/01/2007	60-69		619
England_CPRD	ILD	age_strat	01/01/2007	70+		1549
England_CPRD	ILD	age_strat	01/01/2008	40-49		169
England_CPRD	ILD	age_strat	01/01/2008	50-59		273
England_CPRD	ILD	age_strat	01/01/2008	60-69		618
England_CPRD	ILD	age_strat	01/01/2008	70+		1550
England_CPRD	ILD	age_strat	01/01/2009	40-49		147
England_CPRD	ILD	age_strat	01/01/2009	50-59		322
England_CPRD	ILD	age_strat	01/01/2009	60-69		685
England_CPRD	ILD	age_strat	01/01/2009	70+		1678
England_CPRD	ILD	age_strat	01/01/2010	40-49		174
England_CPRD	ILD	age_strat	01/01/2010	50-59		286
England_CPRD	ILD	age_strat	01/01/2010	60-69		635
England_CPRD	ILD	age_strat	01/01/2010	70+		1687
England_CPRD	ILD	age_strat	01/01/2011	40-49		162
England_CPRD	ILD	age_strat	01/01/2011	50-59		285
England_CPRD	ILD	age_strat	01/01/2011	60-69		648
England_CPRD	ILD	age_strat	01/01/2011	70+		1793
England_CPRD	ILD	age_strat	01/01/2012	40-49		165
England_CPRD	ILD	age_strat	01/01/2012	50-59		313
England_CPRD	ILD	age_strat	01/01/2012	60-69		725
England_CPRD	ILD	age_strat	01/01/2012	70+		1931
England_CPRD	ILD	age_strat	01/01/2013	40-49		181
England_CPRD	ILD	age_strat	01/01/2013	50-59		313
England_CPRD	ILD	age_strat	01/01/2013	60-69		725
England_CPRD	ILD	age_strat	01/01/2013	70+		2137
England_CPRD	ILD	age_strat	01/01/2014	40-49		170
England_CPRD	ILD	age_strat	01/01/2014	50-59		320
England_CPRD	ILD	age_strat	01/01/2014	60-69		739
England_CPRD	ILD	age_strat	01/01/2014	70+		2163
England_CPRD	ILD	age_strat	01/01/2015	40-49		161
England_CPRD	ILD	age_strat	01/01/2015	50-59		348
England_CPRD	ILD	age_strat	01/01/2015	60-69		740
England_CPRD	ILD	age_strat	01/01/2015	70+		2355
England_CPRD	ILD	age_strat	01/01/2016	40-49		220
England_CPRD	ILD	age_strat	01/01/2016	50-59		350
England_CPRD	ILD	age_strat	01/01/2016	60-69		756
England_CPRD	ILD	age_strat	01/01/2016	70+		2511
England_CPRD	ILD	age_strat	01/01/2017	40-49		207
England_CPRD	ILD	age_strat	01/01/2017	50-59		407
England_CPRD	ILD	age_strat	01/01/2017	60-69		772
England_CPRD	ILD	age_strat	01/01/2017	70+		2724
England_CPRD	ILD	age_strat	01/01/2018	40-49		200
England_CPRD	ILD	age_strat	01/01/2018	50-59		431
England_CPRD	ILD	age_strat	01/01/2018	60-69		756
England_CPRD	ILD	age_strat	01/01/2018	70+		2938
England_CPRD	ILD	age_strat	01/01/2019	40-49		212
England_CPRD	ILD	age_strat	01/01/2019	50-59		384
England_CPRD	ILD	age_strat	01/01/2019	60-69		753
England_CPRD	ILD	age_strat	01/01/2019	70+		2875
England_CPRD	Asthma	region_strat	01/01/2004		North East	2588
England_CPRD	Asthma	region_strat	01/01/2004		North West	12553
England_CPRD	Asthma	region_strat	01/01/2004		Yorkshire and The Humber	2049
England_CPRD	Asthma	region_strat	01/01/2004		East Midlands	1260
England_CPRD	Asthma	region_strat	01/01/2004		West Midlands	10900
England_CPRD	Asthma	region_strat	01/01/2004		East of England	2794
England_CPRD	Asthma	region_strat	01/01/2004		London	9817
England_CPRD	Asthma	region_strat	01/01/2004		South East	11891
England_CPRD	Asthma	region_strat	01/01/2004		South West	6578
England_CPRD	Asthma	region_strat	01/01/2005		North East	2088
England_CPRD	Asthma	region_strat	01/01/2005		North West	11134
England_CPRD	Asthma	region_strat	01/01/2005		Yorkshire and The Humber	1626
England_CPRD	Asthma	region_strat	01/01/2005		East Midlands	1099

England_CPRD	Asthma	region_strat	01/01/2005	West Midlands	9344
England_CPRD	Asthma	region_strat	01/01/2005	East of England	2145
England_CPRD	Asthma	region_strat	01/01/2005	London	9399
England_CPRD	Asthma	region_strat	01/01/2005	South East	10137
England_CPRD	Asthma	region_strat	01/01/2005	South West	6242
England_CPRD	Asthma	region_strat	01/01/2006	North East	2085
England_CPRD	Asthma	region_strat	01/01/2006	North West	11525
England_CPRD	Asthma	region_strat	01/01/2006	Yorkshire and The Humber	1793
England_CPRD	Asthma	region_strat	01/01/2006	East Midlands	1108
England_CPRD	Asthma	region_strat	01/01/2006	West Midlands	9248
England_CPRD	Asthma	region_strat	01/01/2006	East of England	2193
England_CPRD	Asthma	region_strat	01/01/2006	London	10286
England_CPRD	Asthma	region_strat	01/01/2006	South East	11218
England_CPRD	Asthma	region_strat	01/01/2006	South West	6903
England_CPRD	Asthma	region_strat	01/01/2007	North East	1768
England_CPRD	Asthma	region_strat	01/01/2007	North West	10442
England_CPRD	Asthma	region_strat	01/01/2007	Yorkshire and The Humber	1726
England_CPRD	Asthma	region_strat	01/01/2007	East Midlands	1011
England_CPRD	Asthma	region_strat	01/01/2007	West Midlands	8434
England_CPRD	Asthma	region_strat	01/01/2007	East of England	2145
England_CPRD	Asthma	region_strat	01/01/2007	London	9220
England_CPRD	Asthma	region_strat	01/01/2007	South East	10646
England_CPRD	Asthma	region_strat	01/01/2007	South West	6347
England_CPRD	Asthma	region_strat	01/01/2008	North East	1705
England_CPRD	Asthma	region_strat	01/01/2008	North West	10353
England_CPRD	Asthma	region_strat	01/01/2008	Yorkshire and The Humber	1872
England_CPRD	Asthma	region_strat	01/01/2008	East Midlands	1004
England_CPRD	Asthma	region_strat	01/01/2008	West Midlands	8773
England_CPRD	Asthma	region_strat	01/01/2008	East of England	2310
England_CPRD	Asthma	region_strat	01/01/2008	London	9350
England_CPRD	Asthma	region_strat	01/01/2008	South East	10828
England_CPRD	Asthma	region_strat	01/01/2008	South West	6586
England_CPRD	Asthma	region_strat	01/01/2009	North East	1629
England_CPRD	Asthma	region_strat	01/01/2009	North West	10100
England_CPRD	Asthma	region_strat	01/01/2009	Yorkshire and The Humber	1882
England_CPRD	Asthma	region_strat	01/01/2009	East Midlands	1047
England_CPRD	Asthma	region_strat	01/01/2009	West Midlands	8517
England_CPRD	Asthma	region_strat	01/01/2009	East of England	2346
England_CPRD	Asthma	region_strat	01/01/2009	London	9821
England_CPRD	Asthma	region_strat	01/01/2009	South East	10531
England_CPRD	Asthma	region_strat	01/01/2009	South West	6561
England_CPRD	Asthma	region_strat	01/01/2010	North East	1545
England_CPRD	Asthma	region_strat	01/01/2010	North West	9544
England_CPRD	Asthma	region_strat	01/01/2010	Yorkshire and The Humber	1774
England_CPRD	Asthma	region_strat	01/01/2010	East Midlands	996
England_CPRD	Asthma	region_strat	01/01/2010	West Midlands	8179
England_CPRD	Asthma	region_strat	01/01/2010	East of England	2108
England_CPRD	Asthma	region_strat	01/01/2010	London	9261
England_CPRD	Asthma	region_strat	01/01/2010	South East	10019
England_CPRD	Asthma	region_strat	01/01/2010	South West	6429
England_CPRD	Asthma	region_strat	01/01/2011	North East	1597
England_CPRD	Asthma	region_strat	01/01/2011	North West	9598
England_CPRD	Asthma	region_strat	01/01/2011	Yorkshire and The Humber	1781
England_CPRD	Asthma	region_strat	01/01/2011	East Midlands	952
England_CPRD	Asthma	region_strat	01/01/2011	West Midlands	7729
England_CPRD	Asthma	region_strat	01/01/2011	East of England	2110
England_CPRD	Asthma	region_strat	01/01/2011	London	9094
England_CPRD	Asthma	region_strat	01/01/2011	South East	9833
England_CPRD	Asthma	region_strat	01/01/2011	South West	5981
England_CPRD	Asthma	region_strat	01/01/2012	North East	1517
England_CPRD	Asthma	region_strat	01/01/2012	North West	10315
England_CPRD	Asthma	region_strat	01/01/2012	Yorkshire and The Humber	1825
England_CPRD	Asthma	region_strat	01/01/2012	East Midlands	1061

England_CPRD	Asthma	region_strat	01/01/2012	West Midlands	8669
England_CPRD	Asthma	region_strat	01/01/2012	East of England	2270
England_CPRD	Asthma	region_strat	01/01/2012	London	9867
England_CPRD	Asthma	region_strat	01/01/2012	South East	10814
England_CPRD	Asthma	region_strat	01/01/2012	South West	6798
England_CPRD	Asthma	region_strat	01/01/2013	North East	1366
England_CPRD	Asthma	region_strat	01/01/2013	North West	9668
England_CPRD	Asthma	region_strat	01/01/2013	Yorkshire and The Humber	1694
England_CPRD	Asthma	region_strat	01/01/2013	East Midlands	1021
England_CPRD	Asthma	region_strat	01/01/2013	West Midlands	8216
England_CPRD	Asthma	region_strat	01/01/2013	East of England	2161
England_CPRD	Asthma	region_strat	01/01/2013	London	9738
England_CPRD	Asthma	region_strat	01/01/2013	South East	10754
England_CPRD	Asthma	region_strat	01/01/2013	South West	6052
England_CPRD	Asthma	region_strat	01/01/2014	North East	1447
England_CPRD	Asthma	region_strat	01/01/2014	North West	10227
England_CPRD	Asthma	region_strat	01/01/2014	Yorkshire and The Humber	1830
England_CPRD	Asthma	region_strat	01/01/2014	East Midlands	1027
England_CPRD	Asthma	region_strat	01/01/2014	West Midlands	8631
England_CPRD	Asthma	region_strat	01/01/2014	East of England	2442
England_CPRD	Asthma	region_strat	01/01/2014	London	10226
England_CPRD	Asthma	region_strat	01/01/2014	South East	11379
England_CPRD	Asthma	region_strat	01/01/2014	South West	6327
England_CPRD	Asthma	region_strat	01/01/2015	North East	1371
England_CPRD	Asthma	region_strat	01/01/2015	North West	9838
England_CPRD	Asthma	region_strat	01/01/2015	Yorkshire and The Humber	1758
England_CPRD	Asthma	region_strat	01/01/2015	East Midlands	1131
England_CPRD	Asthma	region_strat	01/01/2015	West Midlands	8517
England_CPRD	Asthma	region_strat	01/01/2015	East of England	2271
England_CPRD	Asthma	region_strat	01/01/2015	London	9809
England_CPRD	Asthma	region_strat	01/01/2015	South East	11138
England_CPRD	Asthma	region_strat	01/01/2015	South West	6317
England_CPRD	Asthma	region_strat	01/01/2016	North East	1506
England_CPRD	Asthma	region_strat	01/01/2016	North West	10342
England_CPRD	Asthma	region_strat	01/01/2016	Yorkshire and The Humber	1882
England_CPRD	Asthma	region_strat	01/01/2016	East Midlands	1227
England_CPRD	Asthma	region_strat	01/01/2016	West Midlands	8776
England_CPRD	Asthma	region_strat	01/01/2016	East of England	2434
England_CPRD	Asthma	region_strat	01/01/2016	London	10584
England_CPRD	Asthma	region_strat	01/01/2016	South East	11365
England_CPRD	Asthma	region_strat	01/01/2016	South West	6657
England_CPRD	Asthma	region_strat	01/01/2017	North East	1477
England_CPRD	Asthma	region_strat	01/01/2017	North West	10181
England_CPRD	Asthma	region_strat	01/01/2017	Yorkshire and The Humber	2038
England_CPRD	Asthma	region_strat	01/01/2017	East Midlands	1254
England_CPRD	Asthma	region_strat	01/01/2017	West Midlands	8750
England_CPRD	Asthma	region_strat	01/01/2017	East of England	2166
England_CPRD	Asthma	region_strat	01/01/2017	London	10585
England_CPRD	Asthma	region_strat	01/01/2017	South East	11580
England_CPRD	Asthma	region_strat	01/01/2017	South West	6899
England_CPRD	Asthma	region_strat	01/01/2018	North East	1472
England_CPRD	Asthma	region_strat	01/01/2018	North West	9788
England_CPRD	Asthma	region_strat	01/01/2018	Yorkshire and The Humber	1764
England_CPRD	Asthma	region_strat	01/01/2018	East Midlands	1104
England_CPRD	Asthma	region_strat	01/01/2018	West Midlands	8334
England_CPRD	Asthma	region_strat	01/01/2018	East of England	2126
England_CPRD	Asthma	region_strat	01/01/2018	London	10256
England_CPRD	Asthma	region_strat	01/01/2018	South East	10654
England_CPRD	Asthma	region_strat	01/01/2018	South West	7090
England_CPRD	Asthma	region_strat	01/01/2019	North East	1711
England_CPRD	Asthma	region_strat	01/01/2019	North West	11113
England_CPRD	Asthma	region_strat	01/01/2019	Yorkshire and The Humber	1931
England_CPRD	Asthma	region_strat	01/01/2019	East Midlands	1264

England_CPRD	Asthma	region_strat	01/01/2019	West Midlands	9095
England_CPRD	Asthma	region_strat	01/01/2019	East of England	2280
England_CPRD	Asthma	region_strat	01/01/2019	London	12038
England_CPRD	Asthma	region_strat	01/01/2019	South East	11565
England_CPRD	Asthma	region_strat	01/01/2019	South West	7225
England_CPRD	COPD	region_strat	01/01/2004	North East	1086
England_CPRD	COPD	region_strat	01/01/2004	North West	4819
England_CPRD	COPD	region_strat	01/01/2004	Yorkshire and The Humber	757
England_CPRD	COPD	region_strat	01/01/2004	East Midlands	410
England_CPRD	COPD	region_strat	01/01/2004	West Midlands	3368
England_CPRD	COPD	region_strat	01/01/2004	East of England	843
England_CPRD	COPD	region_strat	01/01/2004	London	2551
England_CPRD	COPD	region_strat	01/01/2004	South East	3681
England_CPRD	COPD	region_strat	01/01/2004	South West	2381
England_CPRD	COPD	region_strat	01/01/2005	North East	1355
England_CPRD	COPD	region_strat	01/01/2005	North West	6157
England_CPRD	COPD	region_strat	01/01/2005	Yorkshire and The Humber	846
England_CPRD	COPD	region_strat	01/01/2005	East Midlands	476
England_CPRD	COPD	region_strat	01/01/2005	West Midlands	4240
England_CPRD	COPD	region_strat	01/01/2005	East of England	905
England_CPRD	COPD	region_strat	01/01/2005	London	3243
England_CPRD	COPD	region_strat	01/01/2005	South East	4427
England_CPRD	COPD	region_strat	01/01/2005	South West	3052
England_CPRD	COPD	region_strat	01/01/2006	North East	1288
England_CPRD	COPD	region_strat	01/01/2006	North West	5538
England_CPRD	COPD	region_strat	01/01/2006	Yorkshire and The Humber	795
England_CPRD	COPD	region_strat	01/01/2006	East Midlands	446
England_CPRD	COPD	region_strat	01/01/2006	West Midlands	3962
England_CPRD	COPD	region_strat	01/01/2006	East of England	868
England_CPRD	COPD	region_strat	01/01/2006	London	3348
England_CPRD	COPD	region_strat	01/01/2006	South East	4200
England_CPRD	COPD	region_strat	01/01/2006	South West	3115
England_CPRD	COPD	region_strat	01/01/2007	North East	1244
England_CPRD	COPD	region_strat	01/01/2007	North West	5571
England_CPRD	COPD	region_strat	01/01/2007	Yorkshire and The Humber	773
England_CPRD	COPD	region_strat	01/01/2007	East Midlands	372
England_CPRD	COPD	region_strat	01/01/2007	West Midlands	3866
England_CPRD	COPD	region_strat	01/01/2007	East of England	835
England_CPRD	COPD	region_strat	01/01/2007	London	3148
England_CPRD	COPD	region_strat	01/01/2007	South East	3793
England_CPRD	COPD	region_strat	01/01/2007	South West	2684
England_CPRD	COPD	region_strat	01/01/2008	North East	1080
England_CPRD	COPD	region_strat	01/01/2008	North West	5352
England_CPRD	COPD	region_strat	01/01/2008	Yorkshire and The Humber	828
England_CPRD	COPD	region_strat	01/01/2008	East Midlands	385
England_CPRD	COPD	region_strat	01/01/2008	West Midlands	3845
England_CPRD	COPD	region_strat	01/01/2008	East of England	873
England_CPRD	COPD	region_strat	01/01/2008	London	3080
England_CPRD	COPD	region_strat	01/01/2008	South East	3893
England_CPRD	COPD	region_strat	01/01/2008	South West	2708
England_CPRD	COPD	region_strat	01/01/2009	North East	1134
England_CPRD	COPD	region_strat	01/01/2009	North West	5606
England_CPRD	COPD	region_strat	01/01/2009	Yorkshire and The Humber	879
England_CPRD	COPD	region_strat	01/01/2009	East Midlands	442
England_CPRD	COPD	region_strat	01/01/2009	West Midlands	3960
England_CPRD	COPD	region_strat	01/01/2009	East of England	910
England_CPRD	COPD	region_strat	01/01/2009	London	3388
England_CPRD	COPD	region_strat	01/01/2009	South East	4340
England_CPRD	COPD	region_strat	01/01/2009	South West	2893
England_CPRD	COPD	region_strat	01/01/2010	North East	1134
England_CPRD	COPD	region_strat	01/01/2010	North West	5595
England_CPRD	COPD	region_strat	01/01/2010	Yorkshire and The Humber	896
England_CPRD	COPD	region_strat	01/01/2010	East Midlands	472

England_CPRD	COPD	region_strat	01/01/2010	West Midlands	3719
England_CPRD	COPD	region_strat	01/01/2010	East of England	1185
England_CPRD	COPD	region_strat	01/01/2010	London	3470
England_CPRD	COPD	region_strat	01/01/2010	South East	4296
England_CPRD	COPD	region_strat	01/01/2010	South West	2731
England_CPRD	COPD	region_strat	01/01/2011	North East	1166
England_CPRD	COPD	region_strat	01/01/2011	North West	5607
England_CPRD	COPD	region_strat	01/01/2011	Yorkshire and The Humber	820
England_CPRD	COPD	region_strat	01/01/2011	East Midlands	476
England_CPRD	COPD	region_strat	01/01/2011	West Midlands	3817
England_CPRD	COPD	region_strat	01/01/2011	East of England	1418
England_CPRD	COPD	region_strat	01/01/2011	London	3328
England_CPRD	COPD	region_strat	01/01/2011	South East	4208
England_CPRD	COPD	region_strat	01/01/2011	South West	2858
England_CPRD	COPD	region_strat	01/01/2012	North East	1247
England_CPRD	COPD	region_strat	01/01/2012	North West	5606
England_CPRD	COPD	region_strat	01/01/2012	Yorkshire and The Humber	862
England_CPRD	COPD	region_strat	01/01/2012	East Midlands	439
England_CPRD	COPD	region_strat	01/01/2012	West Midlands	3781
England_CPRD	COPD	region_strat	01/01/2012	East of England	814
England_CPRD	COPD	region_strat	01/01/2012	London	3421
England_CPRD	COPD	region_strat	01/01/2012	South East	4359
England_CPRD	COPD	region_strat	01/01/2012	South West	3006
England_CPRD	COPD	region_strat	01/01/2013	North East	1092
England_CPRD	COPD	region_strat	01/01/2013	North West	5153
England_CPRD	COPD	region_strat	01/01/2013	Yorkshire and The Humber	790
England_CPRD	COPD	region_strat	01/01/2013	East Midlands	464
England_CPRD	COPD	region_strat	01/01/2013	West Midlands	3667
England_CPRD	COPD	region_strat	01/01/2013	East of England	801
England_CPRD	COPD	region_strat	01/01/2013	London	3175
England_CPRD	COPD	region_strat	01/01/2013	South East	4150
England_CPRD	COPD	region_strat	01/01/2013	South West	4464
England_CPRD	COPD	region_strat	01/01/2014	North East	1074
England_CPRD	COPD	region_strat	01/01/2014	North West	5201
England_CPRD	COPD	region_strat	01/01/2014	Yorkshire and The Humber	785
England_CPRD	COPD	region_strat	01/01/2014	East Midlands	419
England_CPRD	COPD	region_strat	01/01/2014	West Midlands	3700
England_CPRD	COPD	region_strat	01/01/2014	East of England	781
England_CPRD	COPD	region_strat	01/01/2014	London	3234
England_CPRD	COPD	region_strat	01/01/2014	South East	4048
England_CPRD	COPD	region_strat	01/01/2014	South West	2679
England_CPRD	COPD	region_strat	01/01/2015	North East	1047
England_CPRD	COPD	region_strat	01/01/2015	North West	5418
England_CPRD	COPD	region_strat	01/01/2015	Yorkshire and The Humber	805
England_CPRD	COPD	region_strat	01/01/2015	East Midlands	438
England_CPRD	COPD	region_strat	01/01/2015	West Midlands	3780
England_CPRD	COPD	region_strat	01/01/2015	East of England	842
England_CPRD	COPD	region_strat	01/01/2015	London	3442
England_CPRD	COPD	region_strat	01/01/2015	South East	4651
England_CPRD	COPD	region_strat	01/01/2015	South West	2930
England_CPRD	COPD	region_strat	01/01/2016	North East	1048
England_CPRD	COPD	region_strat	01/01/2016	North West	5601
England_CPRD	COPD	region_strat	01/01/2016	Yorkshire and The Humber	898
England_CPRD	COPD	region_strat	01/01/2016	East Midlands	413
England_CPRD	COPD	region_strat	01/01/2016	West Midlands	3999
England_CPRD	COPD	region_strat	01/01/2016	East of England	1262
England_CPRD	COPD	region_strat	01/01/2016	London	4498
England_CPRD	COPD	region_strat	01/01/2016	South East	4585
England_CPRD	COPD	region_strat	01/01/2016	South West	2929
England_CPRD	COPD	region_strat	01/01/2017	North East	1027
England_CPRD	COPD	region_strat	01/01/2017	North West	5319
England_CPRD	COPD	region_strat	01/01/2017	Yorkshire and The Humber	962
England_CPRD	COPD	region_strat	01/01/2017	East Midlands	401

England_CPRD	COPD	region_strat	01/01/2017	West Midlands	4483
England_CPRD	COPD	region_strat	01/01/2017	East of England	967
England_CPRD	COPD	region_strat	01/01/2017	London	3717
England_CPRD	COPD	region_strat	01/01/2017	South East	4477
England_CPRD	COPD	region_strat	01/01/2017	South West	3005
England_CPRD	COPD	region_strat	01/01/2018	North East	1061
England_CPRD	COPD	region_strat	01/01/2018	North West	5371
England_CPRD	COPD	region_strat	01/01/2018	Yorkshire and The Humber	886
England_CPRD	COPD	region_strat	01/01/2018	East Midlands	465
England_CPRD	COPD	region_strat	01/01/2018	West Midlands	4748
England_CPRD	COPD	region_strat	01/01/2018	East of England	853
England_CPRD	COPD	region_strat	01/01/2018	London	3342
England_CPRD	COPD	region_strat	01/01/2018	South East	4238
England_CPRD	COPD	region_strat	01/01/2018	South West	3071
England_CPRD	COPD	region_strat	01/01/2019	North East	1018
England_CPRD	COPD	region_strat	01/01/2019	North West	5467
England_CPRD	COPD	region_strat	01/01/2019	Yorkshire and The Humber	808
England_CPRD	COPD	region_strat	01/01/2019	East Midlands	463
England_CPRD	COPD	region_strat	01/01/2019	West Midlands	6166
England_CPRD	COPD	region_strat	01/01/2019	East of England	839
England_CPRD	COPD	region_strat	01/01/2019	London	3094
England_CPRD	COPD	region_strat	01/01/2019	South East	4372
England_CPRD	COPD	region_strat	01/01/2019	South West	2935
England_CPRD	ILD	region_strat	01/01/2004	North East	56
England_CPRD	ILD	region_strat	01/01/2004	North West	206
England_CPRD	ILD	region_strat	01/01/2004	Yorkshire and The Humber	42
England_CPRD	ILD	region_strat	01/01/2004	East Midlands	27
England_CPRD	ILD	region_strat	01/01/2004	West Midlands	142
England_CPRD	ILD	region_strat	01/01/2004	East of England	27
England_CPRD	ILD	region_strat	01/01/2004	London	104
England_CPRD	ILD	region_strat	01/01/2004	South East	140
England_CPRD	ILD	region_strat	01/01/2004	South West	121
England_CPRD	ILD	region_strat	01/01/2005	North East	62
England_CPRD	ILD	region_strat	01/01/2005	North West	203
England_CPRD	ILD	region_strat	01/01/2005	Yorkshire and The Humber	47
England_CPRD	ILD	region_strat	01/01/2005	East Midlands	12
England_CPRD	ILD	region_strat	01/01/2005	West Midlands	167
England_CPRD	ILD	region_strat	01/01/2005	East of England	48
England_CPRD	ILD	region_strat	01/01/2005	London	142
England_CPRD	ILD	region_strat	01/01/2005	South East	179
England_CPRD	ILD	region_strat	01/01/2005	South West	162
England_CPRD	ILD	region_strat	01/01/2006	North East	138
England_CPRD	ILD	region_strat	01/01/2006	North West	527
England_CPRD	ILD	region_strat	01/01/2006	Yorkshire and The Humber	84
England_CPRD	ILD	region_strat	01/01/2006	East Midlands	62
England_CPRD	ILD	region_strat	01/01/2006	West Midlands	388
England_CPRD	ILD	region_strat	01/01/2006	East of England	90
England_CPRD	ILD	region_strat	01/01/2006	London	352
England_CPRD	ILD	region_strat	01/01/2006	South East	439
England_CPRD	ILD	region_strat	01/01/2006	South West	372
England_CPRD	ILD	region_strat	01/01/2007	North East	143
England_CPRD	ILD	region_strat	01/01/2007	North West	596
England_CPRD	ILD	region_strat	01/01/2007	Yorkshire and The Humber	95
England_CPRD	ILD	region_strat	01/01/2007	East Midlands	60
England_CPRD	ILD	region_strat	01/01/2007	West Midlands	405
England_CPRD	ILD	region_strat	01/01/2007	East of England	91
England_CPRD	ILD	region_strat	01/01/2007	London	328
England_CPRD	ILD	region_strat	01/01/2007	South East	518
England_CPRD	ILD	region_strat	01/01/2007	South West	357
England_CPRD	ILD	region_strat	01/01/2008	North East	118
England_CPRD	ILD	region_strat	01/01/2008	North West	567
England_CPRD	ILD	region_strat	01/01/2008	Yorkshire and The Humber	107
England_CPRD	ILD	region_strat	01/01/2008	East Midlands	43

England_CPRD	ILD	region_strat	01/01/2008	West Midlands	465
England_CPRD	ILD	region_strat	01/01/2008	East of England	109
England_CPRD	ILD	region_strat	01/01/2008	London	345
England_CPRD	ILD	region_strat	01/01/2008	South East	497
England_CPRD	ILD	region_strat	01/01/2008	South West	359
England_CPRD	ILD	region_strat	01/01/2009	North East	151
England_CPRD	ILD	region_strat	01/01/2009	North West	621
England_CPRD	ILD	region_strat	01/01/2009	Yorkshire and The Humber	111
England_CPRD	ILD	region_strat	01/01/2009	East Midlands	65
England_CPRD	ILD	region_strat	01/01/2009	West Midlands	492
England_CPRD	ILD	region_strat	01/01/2009	East of England	113
England_CPRD	ILD	region_strat	01/01/2009	London	340
England_CPRD	ILD	region_strat	01/01/2009	South East	563
England_CPRD	ILD	region_strat	01/01/2009	South West	376
England_CPRD	ILD	region_strat	01/01/2010	North East	138
England_CPRD	ILD	region_strat	01/01/2010	North West	624
England_CPRD	ILD	region_strat	01/01/2010	Yorkshire and The Humber	104
England_CPRD	ILD	region_strat	01/01/2010	East Midlands	52
England_CPRD	ILD	region_strat	01/01/2010	West Midlands	445
England_CPRD	ILD	region_strat	01/01/2010	East of England	118
England_CPRD	ILD	region_strat	01/01/2010	London	343
England_CPRD	ILD	region_strat	01/01/2010	South East	564
England_CPRD	ILD	region_strat	01/01/2010	South West	394
England_CPRD	ILD	region_strat	01/01/2011	North East	125
England_CPRD	ILD	region_strat	01/01/2011	North West	642
England_CPRD	ILD	region_strat	01/01/2011	Yorkshire and The Humber	130
England_CPRD	ILD	region_strat	01/01/2011	East Midlands	52
England_CPRD	ILD	region_strat	01/01/2011	West Midlands	500
England_CPRD	ILD	region_strat	01/01/2011	East of England	136
England_CPRD	ILD	region_strat	01/01/2011	London	352
England_CPRD	ILD	region_strat	01/01/2011	South East	563
England_CPRD	ILD	region_strat	01/01/2011	South West	388
England_CPRD	ILD	region_strat	01/01/2012	North East	175
England_CPRD	ILD	region_strat	01/01/2012	North West	723
England_CPRD	ILD	region_strat	01/01/2012	Yorkshire and The Humber	127
England_CPRD	ILD	region_strat	01/01/2012	East Midlands	64
England_CPRD	ILD	region_strat	01/01/2012	West Midlands	555
England_CPRD	ILD	region_strat	01/01/2012	East of England	137
England_CPRD	ILD	region_strat	01/01/2012	London	389
England_CPRD	ILD	region_strat	01/01/2012	South East	567
England_CPRD	ILD	region_strat	01/01/2012	South West	397
England_CPRD	ILD	region_strat	01/01/2013	North East	142
England_CPRD	ILD	region_strat	01/01/2013	North West	787
England_CPRD	ILD	region_strat	01/01/2013	Yorkshire and The Humber	163
England_CPRD	ILD	region_strat	01/01/2013	East Midlands	66
England_CPRD	ILD	region_strat	01/01/2013	West Midlands	575
England_CPRD	ILD	region_strat	01/01/2013	East of England	138
England_CPRD	ILD	region_strat	01/01/2013	London	414
England_CPRD	ILD	region_strat	01/01/2013	South East	635
England_CPRD	ILD	region_strat	01/01/2013	South West	436
England_CPRD	ILD	region_strat	01/01/2014	North East	144
England_CPRD	ILD	region_strat	01/01/2014	North West	831
England_CPRD	ILD	region_strat	01/01/2014	Yorkshire and The Humber	109
England_CPRD	ILD	region_strat	01/01/2014	East Midlands	55
England_CPRD	ILD	region_strat	01/01/2014	West Midlands	566
England_CPRD	ILD	region_strat	01/01/2014	East of England	141
England_CPRD	ILD	region_strat	01/01/2014	London	394
England_CPRD	ILD	region_strat	01/01/2014	South East	658
England_CPRD	ILD	region_strat	01/01/2014	South West	494
England_CPRD	ILD	region_strat	01/01/2015	North East	154
England_CPRD	ILD	region_strat	01/01/2015	North West	799
England_CPRD	ILD	region_strat	01/01/2015	Yorkshire and The Humber	143
England_CPRD	ILD	region_strat	01/01/2015	East Midlands	54

England_CPRD	ILD	region_strat	01/01/2015		West Midlands	654
England_CPRD	ILD	region_strat	01/01/2015		East of England	148
England_CPRD	ILD	region_strat	01/01/2015		London	417
England_CPRD	ILD	region_strat	01/01/2015		South East	754
England_CPRD	ILD	region_strat	01/01/2015		South West	481
England_CPRD	ILD	region_strat	01/01/2016		North East	177
England_CPRD	ILD	region_strat	01/01/2016		North West	877
England_CPRD	ILD	region_strat	01/01/2016		Yorkshire and The Humber	137
England_CPRD	ILD	region_strat	01/01/2016		East Midlands	59
England_CPRD	ILD	region_strat	01/01/2016		West Midlands	675
England_CPRD	ILD	region_strat	01/01/2016		East of England	194
England_CPRD	ILD	region_strat	01/01/2016		London	407
England_CPRD	ILD	region_strat	01/01/2016		South East	824
England_CPRD	ILD	region_strat	01/01/2016		South West	487
England_CPRD	ILD	region_strat	01/01/2017		North East	165
England_CPRD	ILD	region_strat	01/01/2017		North West	904
England_CPRD	ILD	region_strat	01/01/2017		Yorkshire and The Humber	137
England_CPRD	ILD	region_strat	01/01/2017		East Midlands	99
England_CPRD	ILD	region_strat	01/01/2017		West Midlands	719
England_CPRD	ILD	region_strat	01/01/2017		East of England	164
England_CPRD	ILD	region_strat	01/01/2017		London	530
England_CPRD	ILD	region_strat	01/01/2017		South East	850
England_CPRD	ILD	region_strat	01/01/2017		South West	542
England_CPRD	ILD	region_strat	01/01/2018		North East	166
England_CPRD	ILD	region_strat	01/01/2018		North West	979
England_CPRD	ILD	region_strat	01/01/2018		Yorkshire and The Humber	145
England_CPRD	ILD	region_strat	01/01/2018		East Midlands	78
England_CPRD	ILD	region_strat	01/01/2018		West Midlands	710
England_CPRD	ILD	region_strat	01/01/2018		East of England	165
England_CPRD	ILD	region_strat	01/01/2018		London	543
England_CPRD	ILD	region_strat	01/01/2018		South East	978
England_CPRD	ILD	region_strat	01/01/2018		South West	561
England_CPRD	ILD	region_strat	01/01/2019		North East	131
England_CPRD	ILD	region_strat	01/01/2019		North West	1033
England_CPRD	ILD	region_strat	01/01/2019		Yorkshire and The Humber	156
England_CPRD	ILD	region_strat	01/01/2019		East Midlands	85
England_CPRD	ILD	region_strat	01/01/2019		West Midlands	736
England_CPRD	ILD	region_strat	01/01/2019		East of England	169
England_CPRD	ILD	region_strat	01/01/2019		London	487
England_CPRD	ILD	region_strat	01/01/2019		South East	901
England_CPRD	ILD	region_strat	01/01/2019		South West	526
Northern Ireland	Asthma	all	26/06/1905	All persons		7,407
Northern Ireland	Asthma	all	27/06/1905	All persons		6,694
Northern Ireland	Asthma	all	28/06/1905	All persons		5,778
Northern Ireland	Asthma	all	29/06/1905	All persons		5,106
Northern Ireland	Asthma	all	30/06/1905	All persons		5,331
Northern Ireland	Asthma	all	01/07/1905	All persons		5,878
Northern Ireland	Asthma	all	02/07/1905	All persons		5,639
Northern Ireland	Asthma	all	03/07/1905	All persons		5,454
Northern Ireland	Asthma	all	04/07/1905	All persons		5,646
Northern Ireland	Asthma	all	05/07/1905	All persons		5,161
Northern Ireland	Asthma	all	06/07/1905	All persons		5,437
Northern Ireland	Asthma	all	07/07/1905	All persons		5,443
Northern Ireland	Asthma	all	08/07/1905	All persons		5,744
Northern Ireland	Asthma	all	09/07/1905	All persons		5,778
Northern Ireland	Asthma	all	10/07/1905	All persons		5,696
Northern Ireland	Asthma	all	11/07/1905	All persons		6,145
Northern Ireland	Asthma	all	12/07/1905	All persons		3,676
Northern Ireland	Asthma	all	13/07/1905	All persons		3,725
Northern Ireland	Asthma	all	14/07/1905	All persons		5,375
Northern Ireland	Asthma	stratified by sex	26/06/1905	Males		3,588
Northern Ireland	Asthma	stratified by sex	26/06/1905	Females		3,813
Northern Ireland	Asthma	stratified by sex	27/06/1905	Males		3,263

Northern Ireland	Asthma	stratified by sex	27/06/1905	Females		3,422
Northern Ireland	Asthma	stratified by sex	28/06/1905	Males		2,816
Northern Ireland	Asthma	stratified by sex	28/06/1905	Females		2,957
Northern Ireland	Asthma	stratified by sex	29/06/1905	Males		2,588
Northern Ireland	Asthma	stratified by sex	29/06/1905	Females		2,514
Northern Ireland	Asthma	stratified by sex	30/06/1905	Males		2,672
Northern Ireland	Asthma	stratified by sex	30/06/1905	Females		2,654
Northern Ireland	Asthma	stratified by sex	01/07/1905	Males		2,928
Northern Ireland	Asthma	stratified by sex	01/07/1905	Females		2,943
Northern Ireland	Asthma	stratified by sex	02/07/1905	Males		2,798
Northern Ireland	Asthma	stratified by sex	02/07/1905	Females		2,837
Northern Ireland	Asthma	stratified by sex	03/07/1905	Males		2,818
Northern Ireland	Asthma	stratified by sex	03/07/1905	Females		2,635
Northern Ireland	Asthma	stratified by sex	04/07/1905	Males		2,916
Northern Ireland	Asthma	stratified by sex	04/07/1905	Females		2,728
Northern Ireland	Asthma	stratified by sex	05/07/1905	Males		2,551
Northern Ireland	Asthma	stratified by sex	05/07/1905	Females		2,606
Northern Ireland	Asthma	stratified by sex	06/07/1905	Males		2,709
Northern Ireland	Asthma	stratified by sex	06/07/1905	Females		2,725
Northern Ireland	Asthma	stratified by sex	07/07/1905	Males		2,704
Northern Ireland	Asthma	stratified by sex	07/07/1905	Females		2,736
Northern Ireland	Asthma	stratified by sex	08/07/1905	Males		2,869
Northern Ireland	Asthma	stratified by sex	08/07/1905	Females		2,874
Northern Ireland	Asthma	stratified by sex	09/07/1905	Males		2,806
Northern Ireland	Asthma	stratified by sex	09/07/1905	Females		2,970
Northern Ireland	Asthma	stratified by sex	10/07/1905	Males		2,744
Northern Ireland	Asthma	stratified by sex	10/07/1905	Females		2,948
Northern Ireland	Asthma	stratified by sex	11/07/1905	Males		2,941
Northern Ireland	Asthma	stratified by sex	11/07/1905	Females		3,203
Northern Ireland	Asthma	stratified by sex	12/07/1905	Males		1,731
Northern Ireland	Asthma	stratified by sex	12/07/1905	Females		1,944
Northern Ireland	Asthma	stratified by sex	13/07/1905	Males		1,792
Northern Ireland	Asthma	stratified by sex	13/07/1905	Females		1,931
Northern Ireland	Asthma	stratified by sex	14/07/1905	Males		2,609
Northern Ireland	Asthma	stratified by sex	14/07/1905	Females		2,765
Northern Ireland	Asthma	stratified by age	26/06/1905	All persons	0-4	2009
Northern Ireland	Asthma	stratified by age	26/06/1905	All persons	5-9anos	1,036
Northern Ireland	Asthma	stratified by age	26/06/1905	All persons	10-14anos	606
Northern Ireland	Asthma	stratified by age	26/06/1905	All persons	15-19	417
Northern Ireland	Asthma	stratified by age	26/06/1905	All persons	20-30	641
Northern Ireland	Asthma	stratified by age	26/06/1905	All persons	30-40	801
Northern Ireland	Asthma	stratified by age	26/06/1905	All persons	40-50	812
Northern Ireland	Asthma	stratified by age	26/06/1905	All persons	50-60	628
Northern Ireland	Asthma	stratified by age	26/06/1905	All persons	60-70	384
Northern Ireland	Asthma	stratified by age	26/06/1905	All persons	70+	72
Northern Ireland	Asthma	stratified by age	27/06/1905	All persons	0-4	1,812
Northern Ireland	Asthma	stratified by age	27/06/1905	All persons	5-9anos	944
Northern Ireland	Asthma	stratified by age	27/06/1905	All persons	10-14anos	569
Northern Ireland	Asthma	stratified by age	27/06/1905	All persons	15-19	364
Northern Ireland	Asthma	stratified by age	27/06/1905	All persons	20-30	641
Northern Ireland	Asthma	stratified by age	27/06/1905	All persons	30-40	745
Northern Ireland	Asthma	stratified by age	27/06/1905	All persons	40-50	700
Northern Ireland	Asthma	stratified by age	27/06/1905	All persons	50-60	524
Northern Ireland	Asthma	stratified by age	27/06/1905	All persons	60-70	332
Northern Ireland	Asthma	stratified by age	27/06/1905	All persons	70+	63
Northern Ireland	Asthma	stratified by age	28/06/1905	All persons	0-4	1,659
Northern Ireland	Asthma	stratified by age	28/06/1905	All persons	5-9anos	865
Northern Ireland	Asthma	stratified by age	28/06/1905	All persons	10-14anos	489
Northern Ireland	Asthma	stratified by age	28/06/1905	All persons	15-19	281
Northern Ireland	Asthma	stratified by age	28/06/1905	All persons	20-30	491
Northern Ireland	Asthma	stratified by age	28/06/1905	All persons	30-40	582
Northern Ireland	Asthma	stratified by age	28/06/1905	All persons	40-50	600
Northern Ireland	Asthma	stratified by age	28/06/1905	All persons	50-60	451

Northern Ireland	Asthma	stratified by age	28/06/1905	All persons	60-70	289
Northern Ireland	Asthma	stratified by age	28/06/1905	All persons	70+	71
Northern Ireland	Asthma	stratified by age	29/06/1905	All persons	0-4	1,464
Northern Ireland	Asthma	stratified by age	29/06/1905	All persons	5-9anos	716
Northern Ireland	Asthma	stratified by age	29/06/1905	All persons	10-14anos	439
Northern Ireland	Asthma	stratified by age	29/06/1905	All persons	15-19	262
Northern Ireland	Asthma	stratified by age	29/06/1905	All persons	20-30	453
Northern Ireland	Asthma	stratified by age	29/06/1905	All persons	30-40	464
Northern Ireland	Asthma	stratified by age	29/06/1905	All persons	40-50	531
Northern Ireland	Asthma	stratified by age	29/06/1905	All persons	50-60	414
Northern Ireland	Asthma	stratified by age	29/06/1905	All persons	60-70	280
Northern Ireland	Asthma	stratified by age	29/06/1905	All persons	70+	82
Northern Ireland	Asthma	stratified by age	30/06/1905	All persons	0-4	1,463
Northern Ireland	Asthma	stratified by age	30/06/1905	All persons	5-9anos	776
Northern Ireland	Asthma	stratified by age	30/06/1905	All persons	10-14anos	455
Northern Ireland	Asthma	stratified by age	30/06/1905	All persons	15-19	264
Northern Ireland	Asthma	stratified by age	30/06/1905	All persons	20-30	446
Northern Ireland	Asthma	stratified by age	30/06/1905	All persons	30-40	514
Northern Ireland	Asthma	stratified by age	30/06/1905	All persons	40-50	606
Northern Ireland	Asthma	stratified by age	30/06/1905	All persons	50-60	426
Northern Ireland	Asthma	stratified by age	30/06/1905	All persons	60-70	293
Northern Ireland	Asthma	stratified by age	30/06/1905	All persons	70+	87
Northern Ireland	Asthma	stratified by age	01/07/1905	All persons	0-4	1,696
Northern Ireland	Asthma	stratified by age	01/07/1905	All persons	5-9anos	930
Northern Ireland	Asthma	stratified by age	01/07/1905	All persons	10-14anos	487
Northern Ireland	Asthma	stratified by age	01/07/1905	All persons	15-19	257
Northern Ireland	Asthma	stratified by age	01/07/1905	All persons	20-30	429
Northern Ireland	Asthma	stratified by age	01/07/1905	All persons	30-40	529
Northern Ireland	Asthma	stratified by age	01/07/1905	All persons	40-50	645
Northern Ireland	Asthma	stratified by age	01/07/1905	All persons	50-60	471
Northern Ireland	Asthma	stratified by age	01/07/1905	All persons	60-70	330
Northern Ireland	Asthma	stratified by age	01/07/1905	All persons	70+	104
Northern Ireland	Asthma	stratified by age	02/07/1905	All persons	0-4	1,513
Northern Ireland	Asthma	stratified by age	02/07/1905	All persons	5-9anos	833
Northern Ireland	Asthma	stratified by age	02/07/1905	All persons	10-14anos	420
Northern Ireland	Asthma	stratified by age	02/07/1905	All persons	15-19	251
Northern Ireland	Asthma	stratified by age	02/07/1905	All persons	20-30	472
Northern Ireland	Asthma	stratified by age	02/07/1905	All persons	30-40	492
Northern Ireland	Asthma	stratified by age	02/07/1905	All persons	40-50	648
Northern Ireland	Asthma	stratified by age	02/07/1905	All persons	50-60	528
Northern Ireland	Asthma	stratified by age	02/07/1905	All persons	60-70	348
Northern Ireland	Asthma	stratified by age	02/07/1905	All persons	70+	133
Northern Ireland	Asthma	stratified by age	03/07/1905	All persons	0-4	1,538
Northern Ireland	Asthma	stratified by age	03/07/1905	All persons	5-9anos	866
Northern Ireland	Asthma	stratified by age	03/07/1905	All persons	10-14anos	384
Northern Ireland	Asthma	stratified by age	03/07/1905	All persons	15-19	222
Northern Ireland	Asthma	stratified by age	03/07/1905	All persons	20-30	429
Northern Ireland	Asthma	stratified by age	03/07/1905	All persons	30-40	462
Northern Ireland	Asthma	stratified by age	03/07/1905	All persons	40-50	591
Northern Ireland	Asthma	stratified by age	03/07/1905	All persons	50-60	488
Northern Ireland	Asthma	stratified by age	03/07/1905	All persons	60-70	332
Northern Ireland	Asthma	stratified by age	03/07/1905	All persons	70+	142
Northern Ireland	Asthma	stratified by age	04/07/1905	All persons	0-4	1,519
Northern Ireland	Asthma	stratified by age	04/07/1905	All persons	5-9anos	943
Northern Ireland	Asthma	stratified by age	04/07/1905	All persons	10-14anos	460
Northern Ireland	Asthma	stratified by age	04/07/1905	All persons	15-19	247
Northern Ireland	Asthma	stratified by age	04/07/1905	All persons	20-30	413
Northern Ireland	Asthma	stratified by age	04/07/1905	All persons	30-40	445
Northern Ireland	Asthma	stratified by age	04/07/1905	All persons	40-50	603
Northern Ireland	Asthma	stratified by age	04/07/1905	All persons	50-60	491
Northern Ireland	Asthma	stratified by age	04/07/1905	All persons	60-70	355
Northern Ireland	Asthma	stratified by age	04/07/1905	All persons	70+	170
Northern Ireland	Asthma	stratified by age	05/07/1905	All persons	0-4	1,217

Northern Ireland	Asthma	stratified by age	05/07/1905	All persons	5-9anos	901
Northern Ireland	Asthma	stratified by age	05/07/1905	All persons	10-14anos	373
Northern Ireland	Asthma	stratified by age	05/07/1905	All persons	15-19	188
Northern Ireland	Asthma	stratified by age	05/07/1905	All persons	20-30	430
Northern Ireland	Asthma	stratified by age	05/07/1905	All persons	30-40	477
Northern Ireland	Asthma	stratified by age	05/07/1905	All persons	40-50	533
Northern Ireland	Asthma	stratified by age	05/07/1905	All persons	50-60	520
Northern Ireland	Asthma	stratified by age	05/07/1905	All persons	60-70	366
Northern Ireland	Asthma	stratified by age	05/07/1905	All persons	70+	156
Northern Ireland	Asthma	stratified by age	06/07/1905	All persons	0-4	1,235
Northern Ireland	Asthma	stratified by age	06/07/1905	All persons	5-9anos	893
Northern Ireland	Asthma	stratified by age	06/07/1905	All persons	10-14anos	450
Northern Ireland	Asthma	stratified by age	06/07/1905	All persons	15-19	259
Northern Ireland	Asthma	stratified by age	06/07/1905	All persons	20-30	474
Northern Ireland	Asthma	stratified by age	06/07/1905	All persons	30-40	482
Northern Ireland	Asthma	stratified by age	06/07/1905	All persons	40-50	584
Northern Ireland	Asthma	stratified by age	06/07/1905	All persons	50-60	507
Northern Ireland	Asthma	stratified by age	06/07/1905	All persons	60-70	372
Northern Ireland	Asthma	stratified by age	06/07/1905	All persons	70+	181
Northern Ireland	Asthma	stratified by age	07/07/1905	All persons	0-4	1,104
Northern Ireland	Asthma	stratified by age	07/07/1905	All persons	5-9anos	964
Northern Ireland	Asthma	stratified by age	07/07/1905	All persons	10-14anos	456
Northern Ireland	Asthma	stratified by age	07/07/1905	All persons	15-19	266
Northern Ireland	Asthma	stratified by age	07/07/1905	All persons	20-30	454
Northern Ireland	Asthma	stratified by age	07/07/1905	All persons	30-40	453
Northern Ireland	Asthma	stratified by age	07/07/1905	All persons	40-50	582
Northern Ireland	Asthma	stratified by age	07/07/1905	All persons	50-60	582
Northern Ireland	Asthma	stratified by age	07/07/1905	All persons	60-70	370
Northern Ireland	Asthma	stratified by age	07/07/1905	All persons	70+	212
Northern Ireland	Asthma	stratified by age	08/07/1905	All persons	0-4	1,188
Northern Ireland	Asthma	stratified by age	08/07/1905	All persons	5-9anos	1,020
Northern Ireland	Asthma	stratified by age	08/07/1905	All persons	10-14anos	488
Northern Ireland	Asthma	stratified by age	08/07/1905	All persons	15-19	243
Northern Ireland	Asthma	stratified by age	08/07/1905	All persons	20-30	437
Northern Ireland	Asthma	stratified by age	08/07/1905	All persons	30-40	525
Northern Ireland	Asthma	stratified by age	08/07/1905	All persons	40-50	608
Northern Ireland	Asthma	stratified by age	08/07/1905	All persons	50-60	565
Northern Ireland	Asthma	stratified by age	08/07/1905	All persons	60-70	424
Northern Ireland	Asthma	stratified by age	08/07/1905	All persons	70+	246
Northern Ireland	Asthma	stratified by age	09/07/1905	All persons	0-4	1,010
Northern Ireland	Asthma	stratified by age	09/07/1905	All persons	5-9anos	1,055
Northern Ireland	Asthma	stratified by age	09/07/1905	All persons	10-14anos	484
Northern Ireland	Asthma	stratified by age	09/07/1905	All persons	15-19	217
Northern Ireland	Asthma	stratified by age	09/07/1905	All persons	20-30	474
Northern Ireland	Asthma	stratified by age	09/07/1905	All persons	30-40	599
Northern Ireland	Asthma	stratified by age	09/07/1905	All persons	40-50	605
Northern Ireland	Asthma	stratified by age	09/07/1905	All persons	50-60	629
Northern Ireland	Asthma	stratified by age	09/07/1905	All persons	60-70	442
Northern Ireland	Asthma	stratified by age	09/07/1905	All persons	70+	263
Northern Ireland	Asthma	stratified by age	10/07/1905	All persons	0-4	857
Northern Ireland	Asthma	stratified by age	10/07/1905	All persons	5-9anos	918
Northern Ireland	Asthma	stratified by age	10/07/1905	All persons	10-14anos	467
Northern Ireland	Asthma	stratified by age	10/07/1905	All persons	15-19	239
Northern Ireland	Asthma	stratified by age	10/07/1905	All persons	20-30	471
Northern Ireland	Asthma	stratified by age	10/07/1905	All persons	30-40	580
Northern Ireland	Asthma	stratified by age	10/07/1905	All persons	40-50	640
Northern Ireland	Asthma	stratified by age	10/07/1905	All persons	50-60	723
Northern Ireland	Asthma	stratified by age	10/07/1905	All persons	60-70	500
Northern Ireland	Asthma	stratified by age	10/07/1905	All persons	70+	301
Northern Ireland	Asthma	stratified by age	11/07/1905	All persons	0-4	820
Northern Ireland	Asthma	stratified by age	11/07/1905	All persons	5-9anos	1,038
Northern Ireland	Asthma	stratified by age	11/07/1905	All persons	10-14anos	608
Northern Ireland	Asthma	stratified by age	11/07/1905	All persons	15-19	266

Northern Ireland	Asthma	stratified by age	11/07/1905	All persons	20-30	545
Northern Ireland	Asthma	stratified by age	11/07/1905	All persons	30-40	599
Northern Ireland	Asthma	stratified by age	11/07/1905	All persons	40-50	646
Northern Ireland	Asthma	stratified by age	11/07/1905	All persons	50-60	719
Northern Ireland	Asthma	stratified by age	11/07/1905	All persons	60-70	556
Northern Ireland	Asthma	stratified by age	11/07/1905	All persons	70+	348
Northern Ireland	Asthma	stratified by age	12/07/1905	All persons	0-4	438
Northern Ireland	Asthma	stratified by age	12/07/1905	All persons	5-9anos	569
Northern Ireland	Asthma	stratified by age	12/07/1905	All persons	10-14anos	322
Northern Ireland	Asthma	stratified by age	12/07/1905	All persons	15-19	150
Northern Ireland	Asthma	stratified by age	12/07/1905	All persons	20-30	370
Northern Ireland	Asthma	stratified by age	12/07/1905	All persons	30-40	420
Northern Ireland	Asthma	stratified by age	12/07/1905	All persons	40-50	446
Northern Ireland	Asthma	stratified by age	12/07/1905	All persons	50-60	434
Northern Ireland	Asthma	stratified by age	12/07/1905	All persons	60-70	325
Northern Ireland	Asthma	stratified by age	12/07/1905	All persons	70+	202
Northern Ireland	Asthma	stratified by age	13/07/1905	All persons	0-4	520
Northern Ireland	Asthma	stratified by age	13/07/1905	All persons	5-9anos	627
Northern Ireland	Asthma	stratified by age	13/07/1905	All persons	10-14anos	275
Northern Ireland	Asthma	stratified by age	13/07/1905	All persons	15-19	151
Northern Ireland	Asthma	stratified by age	13/07/1905	All persons	20-30	432
Northern Ireland	Asthma	stratified by age	13/07/1905	All persons	30-40	448
Northern Ireland	Asthma	stratified by age	13/07/1905	All persons	40-50	405
Northern Ireland	Asthma	stratified by age	13/07/1905	All persons	50-60	417
Northern Ireland	Asthma	stratified by age	13/07/1905	All persons	60-70	278
Northern Ireland	Asthma	stratified by age	13/07/1905	All persons	70+	172
Northern Ireland	Asthma	stratified by age	14/07/1905	All persons	0-4	788
Northern Ireland	Asthma	stratified by age	14/07/1905	All persons	5-9anos	1,125
Northern Ireland	Asthma	stratified by age	14/07/1905	All persons	10-14anos	486
Northern Ireland	Asthma	stratified by age	14/07/1905	All persons	15-19	244
Northern Ireland	Asthma	stratified by age	14/07/1905	All persons	20-30	481
Northern Ireland	Asthma	stratified by age	14/07/1905	All persons	30-40	494
Northern Ireland	Asthma	stratified by age	14/07/1905	All persons	40-50	499
Northern Ireland	Asthma	stratified by age	14/07/1905	All persons	50-60	555
Northern Ireland	Asthma	stratified by age	14/07/1905	All persons	60-70	418
Northern Ireland	Asthma	stratified by age	14/07/1905	All persons	70+	285
Northern Ireland	Asthma	stratified by age and sex	26/06/1905	Males	0-4	1,215
Northern Ireland	Asthma	stratified by age and sex	26/06/1905	Males	5-9anos	624
Northern Ireland	Asthma	stratified by age and sex	26/06/1905	Males	10-14anos	320
Northern Ireland	Asthma	stratified by age and sex	26/06/1905	Males	15-19	192
Northern Ireland	Asthma	stratified by age and sex	26/06/1905	Males	20-30	287
Northern Ireland	Asthma	stratified by age and sex	26/06/1905	Males	30-40	301
Northern Ireland	Asthma	stratified by age and sex	26/06/1905	Males	40-50	294
Northern Ireland	Asthma	stratified by age and sex	26/06/1905	Males	50-60	210
Northern Ireland	Asthma	stratified by age and sex	26/06/1905	Males	60-70	125
Northern Ireland	Asthma	stratified by age and sex	26/06/1905	Males	70+	20
Northern Ireland	Asthma	stratified by age and sex	27/06/1905	Males	0-4	1,107
Northern Ireland	Asthma	stratified by age and sex	27/06/1905	Males	5-9anos	540
Northern Ireland	Asthma	stratified by age and sex	27/06/1905	Males	10-14anos	301
Northern Ireland	Asthma	stratified by age and sex	27/06/1905	Males	15-19	144
Northern Ireland	Asthma	stratified by age and sex	27/06/1905	Males	20-30	248
Northern Ireland	Asthma	stratified by age and sex	27/06/1905	Males	30-40	311
Northern Ireland	Asthma	stratified by age and sex	27/06/1905	Males	40-50	272
Northern Ireland	Asthma	stratified by age and sex	27/06/1905	Males	50-60	208
Northern Ireland	Asthma	stratified by age and sex	27/06/1905	Males	60-70	113
Northern Ireland	Asthma	stratified by age and sex	27/06/1905	Males	70+	19
Northern Ireland	Asthma	stratified by age and sex	28/06/1905	Males	0-4	1,024
Northern Ireland	Asthma	stratified by age and sex	28/06/1905	Males	5-9anos	472
Northern Ireland	Asthma	stratified by age and sex	28/06/1905	Males	10-14anos	261
Northern Ireland	Asthma	stratified by age and sex	28/06/1905	Males	15-19	116
Northern Ireland	Asthma	stratified by age and sex	28/06/1905	Males	20-30	188
Northern Ireland	Asthma	stratified by age and sex	28/06/1905	Males	30-40	217
Northern Ireland	Asthma	stratified by age and sex	28/06/1905	Males	40-50	224

Northern Ireland	Asthma	stratified by age and sex	28/06/1905	Males	50-60	180
Northern Ireland	Asthma	stratified by age and sex	28/06/1905	Males	60-70	114
Northern Ireland	Asthma	stratified by age and sex	28/06/1905	Males	70+	20
Northern Ireland	Asthma	stratified by age and sex	29/06/1905	Males	0-4	877
Northern Ireland	Asthma	stratified by age and sex	29/06/1905	Males	5-9anos	414
Northern Ireland	Asthma	stratified by age and sex	29/06/1905	Males	10-14anos	258
Northern Ireland	Asthma	stratified by age and sex	29/06/1905	Males	15-19	118
Northern Ireland	Asthma	stratified by age and sex	29/06/1905	Males	20-30	191
Northern Ireland	Asthma	stratified by age and sex	29/06/1905	Males	30-40	208
Northern Ireland	Asthma	stratified by age and sex	29/06/1905	Males	40-50	209
Northern Ireland	Asthma	stratified by age and sex	29/06/1905	Males	50-60	172
Northern Ireland	Asthma	stratified by age and sex	29/06/1905	Males	60-70	111
Northern Ireland	Asthma	stratified by age and sex	29/06/1905	Males	70+	30
Northern Ireland	Asthma	stratified by age and sex	30/06/1905	Males	0-4	909
Northern Ireland	Asthma	stratified by age and sex	30/06/1905	Males	5-9anos	449
Northern Ireland	Asthma	stratified by age and sex	30/06/1905	Males	10-14anos	229
Northern Ireland	Asthma	stratified by age and sex	30/06/1905	Males	15-19	111
Northern Ireland	Asthma	stratified by age and sex	30/06/1905	Males	20-30	182
Northern Ireland	Asthma	stratified by age and sex	30/06/1905	Males	30-40	215
Northern Ireland	Asthma	stratified by age and sex	30/06/1905	Males	40-50	249
Northern Ireland	Asthma	stratified by age and sex	30/06/1905	Males	50-60	183
Northern Ireland	Asthma	stratified by age and sex	30/06/1905	Males	60-70	117
Northern Ireland	Asthma	stratified by age and sex	30/06/1905	Males	70+	28
Northern Ireland	Asthma	stratified by age and sex	01/07/1905	Males	0-4	1,030
Northern Ireland	Asthma	stratified by age and sex	01/07/1905	Males	5-9anos	525
Northern Ireland	Asthma	stratified by age and sex	01/07/1905	Males	10-14anos	259
Northern Ireland	Asthma	stratified by age and sex	01/07/1905	Males	15-19	108
Northern Ireland	Asthma	stratified by age and sex	01/07/1905	Males	20-30	182
Northern Ireland	Asthma	stratified by age and sex	01/07/1905	Males	30-40	210
Northern Ireland	Asthma	stratified by age and sex	01/07/1905	Males	40-50	254
Northern Ireland	Asthma	stratified by age and sex	01/07/1905	Males	50-60	196
Northern Ireland	Asthma	stratified by age and sex	01/07/1905	Males	60-70	131
Northern Ireland	Asthma	stratified by age and sex	01/07/1905	Males	70+	33
Northern Ireland	Asthma	stratified by age and sex	02/07/1905	Males	0-4	920
Northern Ireland	Asthma	stratified by age and sex	02/07/1905	Males	5-9anos	486
Northern Ireland	Asthma	stratified by age and sex	02/07/1905	Males	10-14anos	246
Northern Ireland	Asthma	stratified by age and sex	02/07/1905	Males	15-19	109
Northern Ireland	Asthma	stratified by age and sex	02/07/1905	Males	20-30	188
Northern Ireland	Asthma	stratified by age and sex	02/07/1905	Males	30-40	199
Northern Ireland	Asthma	stratified by age and sex	02/07/1905	Males	40-50	250
Northern Ireland	Asthma	stratified by age and sex	02/07/1905	Males	50-60	224
Northern Ireland	Asthma	stratified by age and sex	02/07/1905	Males	60-70	142
Northern Ireland	Asthma	stratified by age and sex	02/07/1905	Males	70+	34
Northern Ireland	Asthma	stratified by age and sex	03/07/1905	Males	0-4	941
Northern Ireland	Asthma	stratified by age and sex	03/07/1905	Males	5-9anos	508
Northern Ireland	Asthma	stratified by age and sex	03/07/1905	Males	10-14anos	212
Northern Ireland	Asthma	stratified by age and sex	03/07/1905	Males	15-19	91
Northern Ireland	Asthma	stratified by age and sex	03/07/1905	Males	20-30	171
Northern Ireland	Asthma	stratified by age and sex	03/07/1905	Males	30-40	206
Northern Ireland	Asthma	stratified by age and sex	03/07/1905	Males	40-50	254
Northern Ireland	Asthma	stratified by age and sex	03/07/1905	Males	50-60	233
Northern Ireland	Asthma	stratified by age and sex	03/07/1905	Males	60-70	139
Northern Ireland	Asthma	stratified by age and sex	03/07/1905	Males	70+	63
Northern Ireland	Asthma	stratified by age and sex	04/07/1905	Males	0-4	942
Northern Ireland	Asthma	stratified by age and sex	04/07/1905	Males	5-9anos	571
Northern Ireland	Asthma	stratified by age and sex	04/07/1905	Males	10-14anos	245
Northern Ireland	Asthma	stratified by age and sex	04/07/1905	Males	15-19	108
Northern Ireland	Asthma	stratified by age and sex	04/07/1905	Males	20-30	178
Northern Ireland	Asthma	stratified by age and sex	04/07/1905	Males	30-40	200
Northern Ireland	Asthma	stratified by age and sex	04/07/1905	Males	40-50	245
Northern Ireland	Asthma	stratified by age and sex	04/07/1905	Males	50-60	223
Northern Ireland	Asthma	stratified by age and sex	04/07/1905	Males	60-70	140
Northern Ireland	Asthma	stratified by age and sex	04/07/1905	Males	70+	64

Northern Ireland	Asthma	stratified by age and sex	05/07/1905	Males	0-4	745
Northern Ireland	Asthma	stratified by age and sex	05/07/1905	Males	5-9anos	518
Northern Ireland	Asthma	stratified by age and sex	05/07/1905	Males	10-14anos	190
Northern Ireland	Asthma	stratified by age and sex	05/07/1905	Males	15-19	81
Northern Ireland	Asthma	stratified by age and sex	05/07/1905	Males	20-30	173
Northern Ireland	Asthma	stratified by age and sex	05/07/1905	Males	30-40	202
Northern Ireland	Asthma	stratified by age and sex	05/07/1905	Males	40-50	232
Northern Ireland	Asthma	stratified by age and sex	05/07/1905	Males	50-60	216
Northern Ireland	Asthma	stratified by age and sex	05/07/1905	Males	60-70	140
Northern Ireland	Asthma	stratified by age and sex	05/07/1905	Males	70+	54
Northern Ireland	Asthma	stratified by age and sex	06/07/1905	Males	0-4	733
Northern Ireland	Asthma	stratified by age and sex	06/07/1905	Males	5-9anos	549
Northern Ireland	Asthma	stratified by age and sex	06/07/1905	Males	10-14anos	258
Northern Ireland	Asthma	stratified by age and sex	06/07/1905	Males	15-19	108
Northern Ireland	Asthma	stratified by age and sex	06/07/1905	Males	20-30	175
Northern Ireland	Asthma	stratified by age and sex	06/07/1905	Males	30-40	213
Northern Ireland	Asthma	stratified by age and sex	06/07/1905	Males	40-50	233
Northern Ireland	Asthma	stratified by age and sex	06/07/1905	Males	50-60	215
Northern Ireland	Asthma	stratified by age and sex	06/07/1905	Males	60-70	158
Northern Ireland	Asthma	stratified by age and sex	06/07/1905	Males	70+	67
Northern Ireland	Asthma	stratified by age and sex	07/07/1905	Males	0-4	661
Northern Ireland	Asthma	stratified by age and sex	07/07/1905	Males	5-9anos	561
Northern Ireland	Asthma	stratified by age and sex	07/07/1905	Males	10-14anos	230
Northern Ireland	Asthma	stratified by age and sex	07/07/1905	Males	15-19	124
Northern Ireland	Asthma	stratified by age and sex	07/07/1905	Males	20-30	184
Northern Ireland	Asthma	stratified by age and sex	07/07/1905	Males	30-40	197
Northern Ireland	Asthma	stratified by age and sex	07/07/1905	Males	40-50	247
Northern Ireland	Asthma	stratified by age and sex	07/07/1905	Males	50-60	257
Northern Ireland	Asthma	stratified by age and sex	07/07/1905	Males	60-70	163
Northern Ireland	Asthma	stratified by age and sex	07/07/1905	Males	70+	80
Northern Ireland	Asthma	stratified by age and sex	08/07/1905	Males	0-4	707
Northern Ireland	Asthma	stratified by age and sex	08/07/1905	Males	5-9anos	582
Northern Ireland	Asthma	stratified by age and sex	08/07/1905	Males	10-14anos	257
Northern Ireland	Asthma	stratified by age and sex	08/07/1905	Males	15-19	97
Northern Ireland	Asthma	stratified by age and sex	08/07/1905	Males	20-30	174
Northern Ireland	Asthma	stratified by age and sex	08/07/1905	Males	30-40	233
Northern Ireland	Asthma	stratified by age and sex	08/07/1905	Males	40-50	268
Northern Ireland	Asthma	stratified by age and sex	08/07/1905	Males	50-60	265
Northern Ireland	Asthma	stratified by age and sex	08/07/1905	Males	60-70	181
Northern Ireland	Asthma	stratified by age and sex	08/07/1905	Males	70+	105
Northern Ireland	Asthma	stratified by age and sex	09/07/1905	Males	0-4	597
Northern Ireland	Asthma	stratified by age and sex	09/07/1905	Males	5-9anos	616
Northern Ireland	Asthma	stratified by age and sex	09/07/1905	Males	10-14anos	258
Northern Ireland	Asthma	stratified by age and sex	09/07/1905	Males	15-19	80
Northern Ireland	Asthma	stratified by age and sex	09/07/1905	Males	20-30	172
Northern Ireland	Asthma	stratified by age and sex	09/07/1905	Males	30-40	238
Northern Ireland	Asthma	stratified by age and sex	09/07/1905	Males	40-50	261
Northern Ireland	Asthma	stratified by age and sex	09/07/1905	Males	50-60	258
Northern Ireland	Asthma	stratified by age and sex	09/07/1905	Males	60-70	198
Northern Ireland	Asthma	stratified by age and sex	09/07/1905	Males	70+	128
Northern Ireland	Asthma	stratified by age and sex	10/07/1905	Males	0-4	508
Northern Ireland	Asthma	stratified by age and sex	10/07/1905	Males	5-9anos	526
Northern Ireland	Asthma	stratified by age and sex	10/07/1905	Males	10-14anos	251
Northern Ireland	Asthma	stratified by age and sex	10/07/1905	Males	15-19	95
Northern Ireland	Asthma	stratified by age and sex	10/07/1905	Males	20-30	177
Northern Ireland	Asthma	stratified by age and sex	10/07/1905	Males	30-40	245
Northern Ireland	Asthma	stratified by age and sex	10/07/1905	Males	40-50	263
Northern Ireland	Asthma	stratified by age and sex	10/07/1905	Males	50-60	330
Northern Ireland	Asthma	stratified by age and sex	10/07/1905	Males	60-70	227
Northern Ireland	Asthma	stratified by age and sex	10/07/1905	Males	70+	122
Northern Ireland	Asthma	stratified by age and sex	11/07/1905	Males	0-4	468
Northern Ireland	Asthma	stratified by age and sex	11/07/1905	Males	5-9anos	610
Northern Ireland	Asthma	stratified by age and sex	11/07/1905	Males	10-14anos	307

Northern Ireland	Asthma	stratified by age and sex	11/07/1905	Males	15-19	118
Northern Ireland	Asthma	stratified by age and sex	11/07/1905	Males	20-30	228
Northern Ireland	Asthma	stratified by age and sex	11/07/1905	Males	30-40	241
Northern Ireland	Asthma	stratified by age and sex	11/07/1905	Males	40-50	260
Northern Ireland	Asthma	stratified by age and sex	11/07/1905	Males	50-60	327
Northern Ireland	Asthma	stratified by age and sex	11/07/1905	Males	60-70	236
Northern Ireland	Asthma	stratified by age and sex	11/07/1905	Males	70+	146
Northern Ireland	Asthma	stratified by age and sex	12/07/1905	Males	0-4	267
Northern Ireland	Asthma	stratified by age and sex	12/07/1905	Males	5-9anos	353
Northern Ireland	Asthma	stratified by age and sex	12/07/1905	Males	10-14anos	176
Northern Ireland	Asthma	stratified by age and sex	12/07/1905	Males	15-19	59
Northern Ireland	Asthma	stratified by age and sex	12/07/1905	Males	20-30	144
Northern Ireland	Asthma	stratified by age and sex	12/07/1905	Males	30-40	166
Northern Ireland	Asthma	stratified by age and sex	12/07/1905	Males	40-50	165
Northern Ireland	Asthma	stratified by age and sex	12/07/1905	Males	50-60	179
Northern Ireland	Asthma	stratified by age and sex	12/07/1905	Males	60-70	129
Northern Ireland	Asthma	stratified by age and sex	12/07/1905	Males	70+	93
Northern Ireland	Asthma	stratified by age and sex	13/07/1905	Males	0-4	309
Northern Ireland	Asthma	stratified by age and sex	13/07/1905	Males	5-9anos	377
Northern Ireland	Asthma	stratified by age and sex	13/07/1905	Males	10-14anos	141
Northern Ireland	Asthma	stratified by age and sex	13/07/1905	Males	15-19	60
Northern Ireland	Asthma	stratified by age and sex	13/07/1905	Males	20-30	163
Northern Ireland	Asthma	stratified by age and sex	13/07/1905	Males	30-40	186
Northern Ireland	Asthma	stratified by age and sex	13/07/1905	Males	40-50	185
Northern Ireland	Asthma	stratified by age and sex	13/07/1905	Males	50-60	174
Northern Ireland	Asthma	stratified by age and sex	13/07/1905	Males	60-70	125
Northern Ireland	Asthma	stratified by age and sex	13/07/1905	Males	70+	72
Northern Ireland	Asthma	stratified by age and sex	14/07/1905	Males	0-4	422
Northern Ireland	Asthma	stratified by age and sex	14/07/1905	Males	5-9anos	648
Northern Ireland	Asthma	stratified by age and sex	14/07/1905	Males	10-14anos	273
Northern Ireland	Asthma	stratified by age and sex	14/07/1905	Males	15-19	114
Northern Ireland	Asthma	stratified by age and sex	14/07/1905	Males	20-30	177
Northern Ireland	Asthma	stratified by age and sex	14/07/1905	Males	30-40	221
Northern Ireland	Asthma	stratified by age and sex	14/07/1905	Males	40-50	177
Northern Ireland	Asthma	stratified by age and sex	14/07/1905	Males	50-60	247
Northern Ireland	Asthma	stratified by age and sex	14/07/1905	Males	60-70	202
Northern Ireland	Asthma	stratified by age and sex	14/07/1905	Males	70+	128
Northern Ireland	Asthma	stratified by age and sex	26/06/1905	Females	0-4	792
Northern Ireland	Asthma	stratified by age and sex	26/06/1905	Females	5-9anos	409
Northern Ireland	Asthma	stratified by age and sex	26/06/1905	Females	10-14anos	286
Northern Ireland	Asthma	stratified by age and sex	26/06/1905	Females	15-19	225
Northern Ireland	Asthma	stratified by age and sex	26/06/1905	Females	20-30	354
Northern Ireland	Asthma	stratified by age and sex	26/06/1905	Females	30-40	500
Northern Ireland	Asthma	stratified by age and sex	26/06/1905	Females	40-50	518
Northern Ireland	Asthma	stratified by age and sex	26/06/1905	Females	50-60	418
Northern Ireland	Asthma	stratified by age and sex	26/06/1905	Females	60-70	259
Northern Ireland	Asthma	stratified by age and sex	26/06/1905	Females	70+	52
Northern Ireland	Asthma	stratified by age and sex	27/06/1905	Females	0-4	701
Northern Ireland	Asthma	stratified by age and sex	27/06/1905	Females	5-9anos	401
Northern Ireland	Asthma	stratified by age and sex	27/06/1905	Females	10-14anos	267
Northern Ireland	Asthma	stratified by age and sex	27/06/1905	Females	15-19	220
Northern Ireland	Asthma	stratified by age and sex	27/06/1905	Females	20-30	393
Northern Ireland	Asthma	stratified by age and sex	27/06/1905	Females	30-40	433
Northern Ireland	Asthma	stratified by age and sex	27/06/1905	Females	40-50	428
Northern Ireland	Asthma	stratified by age and sex	27/06/1905	Females	50-60	316
Northern Ireland	Asthma	stratified by age and sex	27/06/1905	Females	60-70	219
Northern Ireland	Asthma	stratified by age and sex	27/06/1905	Females	70+	44
Northern Ireland	Asthma	stratified by age and sex	28/06/1905	Females	0-4	631
Northern Ireland	Asthma	stratified by age and sex	28/06/1905	Females	5-9anos	393
Northern Ireland	Asthma	stratified by age and sex	28/06/1905	Females	10-14anos	227
Northern Ireland	Asthma	stratified by age and sex	28/06/1905	Females	15-19	165
Northern Ireland	Asthma	stratified by age and sex	28/06/1905	Females	20-30	303
Northern Ireland	Asthma	stratified by age and sex	28/06/1905	Females	30-40	365

Northern Ireland	Asthma	stratified by age and sex	28/06/1905	Females	40-50	376
Northern Ireland	Asthma	stratified by age and sex	28/06/1905	Females	50-60	271
Northern Ireland	Asthma	stratified by age and sex	28/06/1905	Females	60-70	175
Northern Ireland	Asthma	stratified by age and sex	28/06/1905	Females	70+	51
Northern Ireland	Asthma	stratified by age and sex	29/06/1905	Females	0-4	587
Northern Ireland	Asthma	stratified by age and sex	29/06/1905	Females	5-9anos	299
Northern Ireland	Asthma	stratified by age and sex	29/06/1905	Females	10-14anos	181
Northern Ireland	Asthma	stratified by age and sex	29/06/1905	Females	15-19	144
Northern Ireland	Asthma	stratified by age and sex	29/06/1905	Females	20-30	262
Northern Ireland	Asthma	stratified by age and sex	29/06/1905	Females	30-40	256
Northern Ireland	Asthma	stratified by age and sex	29/06/1905	Females	40-50	322
Northern Ireland	Asthma	stratified by age and sex	29/06/1905	Females	50-60	242
Northern Ireland	Asthma	stratified by age and sex	29/06/1905	Females	60-70	169
Northern Ireland	Asthma	stratified by age and sex	29/06/1905	Females	70+	52
Northern Ireland	Asthma	stratified by age and sex	30/06/1905	Females	0-4	554
Northern Ireland	Asthma	stratified by age and sex	30/06/1905	Females	5-9anos	324
Northern Ireland	Asthma	stratified by age and sex	30/06/1905	Females	10-14anos	225
Northern Ireland	Asthma	stratified by age and sex	30/06/1905	Females	15-19	153
Northern Ireland	Asthma	stratified by age and sex	30/06/1905	Females	20-30	264
Northern Ireland	Asthma	stratified by age and sex	30/06/1905	Females	30-40	299
Northern Ireland	Asthma	stratified by age and sex	30/06/1905	Females	40-50	357
Northern Ireland	Asthma	stratified by age and sex	30/06/1905	Females	50-60	243
Northern Ireland	Asthma	stratified by age and sex	30/06/1905	Females	60-70	176
Northern Ireland	Asthma	stratified by age and sex	30/06/1905	Females	70+	59
Northern Ireland	Asthma	stratified by age and sex	01/07/1905	Females	0-4	664
Northern Ireland	Asthma	stratified by age and sex	01/07/1905	Females	5-9anos	404
Northern Ireland	Asthma	stratified by age and sex	01/07/1905	Females	10-14anos	226
Northern Ireland	Asthma	stratified by age and sex	01/07/1905	Females	15-19	149
Northern Ireland	Asthma	stratified by age and sex	01/07/1905	Females	20-30	246
Northern Ireland	Asthma	stratified by age and sex	01/07/1905	Females	30-40	318
Northern Ireland	Asthma	stratified by age and sex	01/07/1905	Females	40-50	391
Northern Ireland	Asthma	stratified by age and sex	01/07/1905	Females	50-60	275
Northern Ireland	Asthma	stratified by age and sex	01/07/1905	Females	60-70	199
Northern Ireland	Asthma	stratified by age and sex	01/07/1905	Females	70+	71
Northern Ireland	Asthma	stratified by age and sex	02/07/1905	Females	0-4	593
Northern Ireland	Asthma	stratified by age and sex	02/07/1905	Females	5-9anos	345
Northern Ireland	Asthma	stratified by age and sex	02/07/1905	Females	10-14anos	174
Northern Ireland	Asthma	stratified by age and sex	02/07/1905	Females	15-19	142
Northern Ireland	Asthma	stratified by age and sex	02/07/1905	Females	20-30	284
Northern Ireland	Asthma	stratified by age and sex	02/07/1905	Females	30-40	292
Northern Ireland	Asthma	stratified by age and sex	02/07/1905	Females	40-50	398
Northern Ireland	Asthma	stratified by age and sex	02/07/1905	Females	50-60	304
Northern Ireland	Asthma	stratified by age and sex	02/07/1905	Females	60-70	206
Northern Ireland	Asthma	stratified by age and sex	02/07/1905	Females	70+	99
Northern Ireland	Asthma	stratified by age and sex	03/07/1905	Females	0-4	597
Northern Ireland	Asthma	stratified by age and sex	03/07/1905	Females	5-9anos	358
Northern Ireland	Asthma	stratified by age and sex	03/07/1905	Females	10-14anos	172
Northern Ireland	Asthma	stratified by age and sex	03/07/1905	Females	15-19	131
Northern Ireland	Asthma	stratified by age and sex	03/07/1905	Females	20-30	258
Northern Ireland	Asthma	stratified by age and sex	03/07/1905	Females	30-40	255
Northern Ireland	Asthma	stratified by age and sex	03/07/1905	Females	40-50	337
Northern Ireland	Asthma	stratified by age and sex	03/07/1905	Females	50-60	255
Northern Ireland	Asthma	stratified by age and sex	03/07/1905	Females	60-70	193
Northern Ireland	Asthma	stratified by age and sex	03/07/1905	Females	70+	79
Northern Ireland	Asthma	stratified by age and sex	04/07/1905	Females	0-4	577
Northern Ireland	Asthma	stratified by age and sex	04/07/1905	Females	5-9anos	371
Northern Ireland	Asthma	stratified by age and sex	04/07/1905	Females	10-14anos	215
Northern Ireland	Asthma	stratified by age and sex	04/07/1905	Females	15-19	139
Northern Ireland	Asthma	stratified by age and sex	04/07/1905	Females	20-30	235
Northern Ireland	Asthma	stratified by age and sex	04/07/1905	Females	30-40	244
Northern Ireland	Asthma	stratified by age and sex	04/07/1905	Females	40-50	358
Northern Ireland	Asthma	stratified by age and sex	04/07/1905	Females	50-60	268
Northern Ireland	Asthma	stratified by age and sex	04/07/1905	Females	60-70	215

Northern Ireland	Asthma	stratified by age and sex	04/07/1905	Females	70+	106
Northern Ireland	Asthma	stratified by age and sex	05/07/1905	Females	0-4	472
Northern Ireland	Asthma	stratified by age and sex	05/07/1905	Females	5-9anos	382
Northern Ireland	Asthma	stratified by age and sex	05/07/1905	Females	10-14anos	182
Northern Ireland	Asthma	stratified by age and sex	05/07/1905	Females	15-19	107
Northern Ireland	Asthma	stratified by age and sex	05/07/1905	Females	20-30	256
Northern Ireland	Asthma	stratified by age and sex	05/07/1905	Females	30-40	274
Northern Ireland	Asthma	stratified by age and sex	05/07/1905	Females	40-50	301
Northern Ireland	Asthma	stratified by age and sex	05/07/1905	Females	50-60	304
Northern Ireland	Asthma	stratified by age and sex	05/07/1905	Females	60-70	226
Northern Ireland	Asthma	stratified by age and sex	05/07/1905	Females	70+	102
Northern Ireland	Asthma	stratified by age and sex	06/07/1905	Females	0-4	502
Northern Ireland	Asthma	stratified by age and sex	06/07/1905	Females	5-9anos	344
Northern Ireland	Asthma	stratified by age and sex	06/07/1905	Females	10-14anos	191
Northern Ireland	Asthma	stratified by age and sex	06/07/1905	Females	15-19	151
Northern Ireland	Asthma	stratified by age and sex	06/07/1905	Females	20-30	297
Northern Ireland	Asthma	stratified by age and sex	06/07/1905	Females	30-40	269
Northern Ireland	Asthma	stratified by age and sex	06/07/1905	Females	40-50	351
Northern Ireland	Asthma	stratified by age and sex	06/07/1905	Females	50-60	292
Northern Ireland	Asthma	stratified by age and sex	06/07/1905	Females	60-70	214
Northern Ireland	Asthma	stratified by age and sex	06/07/1905	Females	70+	114
Northern Ireland	Asthma	stratified by age and sex	07/07/1905	Females	0-4	443
Northern Ireland	Asthma	stratified by age and sex	07/07/1905	Females	5-9anos	403
Northern Ireland	Asthma	stratified by age and sex	07/07/1905	Females	10-14anos	226
Northern Ireland	Asthma	stratified by age and sex	07/07/1905	Females	15-19	141
Northern Ireland	Asthma	stratified by age and sex	07/07/1905	Females	20-30	268
Northern Ireland	Asthma	stratified by age and sex	07/07/1905	Females	30-40	256
Northern Ireland	Asthma	stratified by age and sex	07/07/1905	Females	40-50	335
Northern Ireland	Asthma	stratified by age and sex	07/07/1905	Females	50-60	325
Northern Ireland	Asthma	stratified by age and sex	07/07/1905	Females	60-70	207
Northern Ireland	Asthma	stratified by age and sex	07/07/1905	Females	70+	132
Northern Ireland	Asthma	stratified by age and sex	08/07/1905	Females	0-4	481
Northern Ireland	Asthma	stratified by age and sex	08/07/1905	Females	5-9anos	437
Northern Ireland	Asthma	stratified by age and sex	08/07/1905	Females	10-14anos	231
Northern Ireland	Asthma	stratified by age and sex	08/07/1905	Females	15-19	146
Northern Ireland	Asthma	stratified by age and sex	08/07/1905	Females	20-30	263
Northern Ireland	Asthma	stratified by age and sex	08/07/1905	Females	30-40	292
Northern Ireland	Asthma	stratified by age and sex	08/07/1905	Females	40-50	340
Northern Ireland	Asthma	stratified by age and sex	08/07/1905	Females	50-60	300
Northern Ireland	Asthma	stratified by age and sex	08/07/1905	Females	60-70	243
Northern Ireland	Asthma	stratified by age and sex	08/07/1905	Females	70+	141
Northern Ireland	Asthma	stratified by age and sex	09/07/1905	Females	0-4	413
Northern Ireland	Asthma	stratified by age and sex	09/07/1905	Females	5-9anos	439
Northern Ireland	Asthma	stratified by age and sex	09/07/1905	Females	10-14anos	226
Northern Ireland	Asthma	stratified by age and sex	09/07/1905	Females	15-19	137
Northern Ireland	Asthma	stratified by age and sex	09/07/1905	Females	20-30	301
Northern Ireland	Asthma	stratified by age and sex	09/07/1905	Females	30-40	361
Northern Ireland	Asthma	stratified by age and sex	09/07/1905	Females	40-50	343
Northern Ireland	Asthma	stratified by age and sex	09/07/1905	Females	50-60	371
Northern Ireland	Asthma	stratified by age and sex	09/07/1905	Females	60-70	244
Northern Ireland	Asthma	stratified by age and sex	09/07/1905	Females	70+	135
Northern Ireland	Asthma	stratified by age and sex	10/07/1905	Females	0-4	349
Northern Ireland	Asthma	stratified by age and sex	10/07/1905	Females	5-9anos	392
Northern Ireland	Asthma	stratified by age and sex	10/07/1905	Females	10-14anos	216
Northern Ireland	Asthma	stratified by age and sex	10/07/1905	Females	15-19	142
Northern Ireland	Asthma	stratified by age and sex	10/07/1905	Females	20-30	293
Northern Ireland	Asthma	stratified by age and sex	10/07/1905	Females	30-40	335
Northern Ireland	Asthma	stratified by age and sex	10/07/1905	Females	40-50	376
Northern Ireland	Asthma	stratified by age and sex	10/07/1905	Females	50-60	393
Northern Ireland	Asthma	stratified by age and sex	10/07/1905	Females	60-70	273
Northern Ireland	Asthma	stratified by age and sex	10/07/1905	Females	70+	179
Northern Ireland	Asthma	stratified by age and sex	11/07/1905	Females	0-4	352
Northern Ireland	Asthma	stratified by age and sex	11/07/1905	Females	5-9anos	428

Northern Ireland	Asthma	stratified by age and sex	11/07/1905	Females	10-14anos	301
Northern Ireland	Asthma	stratified by age and sex	11/07/1905	Females	15-19	148
Northern Ireland	Asthma	stratified by age and sex	11/07/1905	Females	20-30	316
Northern Ireland	Asthma	stratified by age and sex	11/07/1905	Females	30-40	358
Northern Ireland	Asthma	stratified by age and sex	11/07/1905	Females	40-50	386
Northern Ireland	Asthma	stratified by age and sex	11/07/1905	Females	50-60	392
Northern Ireland	Asthma	stratified by age and sex	11/07/1905	Females	60-70	320
Northern Ireland	Asthma	stratified by age and sex	11/07/1905	Females	70+	202
Northern Ireland	Asthma	stratified by age and sex	12/07/1905	Females	0-4	171
Northern Ireland	Asthma	stratified by age and sex	12/07/1905	Females	5-9anos	216
Northern Ireland	Asthma	stratified by age and sex	12/07/1905	Females	10-14anos	146
Northern Ireland	Asthma	stratified by age and sex	12/07/1905	Females	15-19	91
Northern Ireland	Asthma	stratified by age and sex	12/07/1905	Females	20-30	225
Northern Ireland	Asthma	stratified by age and sex	12/07/1905	Females	30-40	254
Northern Ireland	Asthma	stratified by age and sex	12/07/1905	Females	40-50	281
Northern Ireland	Asthma	stratified by age and sex	12/07/1905	Females	50-60	255
Northern Ireland	Asthma	stratified by age and sex	12/07/1905	Females	60-70	196
Northern Ireland	Asthma	stratified by age and sex	12/07/1905	Females	70+	109
Northern Ireland	Asthma	stratified by age and sex	13/07/1905	Females	0-4	211
Northern Ireland	Asthma	stratified by age and sex	13/07/1905	Females	5-9anos	250
Northern Ireland	Asthma	stratified by age and sex	13/07/1905	Females	10-14anos	134
Northern Ireland	Asthma	stratified by age and sex	13/07/1905	Females	15-19	91
Northern Ireland	Asthma	stratified by age and sex	13/07/1905	Females	20-30	267
Northern Ireland	Asthma	stratified by age and sex	13/07/1905	Females	30-40	262
Northern Ireland	Asthma	stratified by age and sex	13/07/1905	Females	40-50	220
Northern Ireland	Asthma	stratified by age and sex	13/07/1905	Females	50-60	243
Northern Ireland	Asthma	stratified by age and sex	13/07/1905	Females	60-70	153
Northern Ireland	Asthma	stratified by age and sex	13/07/1905	Females	70+	100
Northern Ireland	Asthma	stratified by age and sex	14/07/1905	Females	0-4	366
Northern Ireland	Asthma	stratified by age and sex	14/07/1905	Females	5-9anos	477
Northern Ireland	Asthma	stratified by age and sex	14/07/1905	Females	10-14anos	213
Northern Ireland	Asthma	stratified by age and sex	14/07/1905	Females	15-19	130
Northern Ireland	Asthma	stratified by age and sex	14/07/1905	Females	20-30	303
Northern Ireland	Asthma	stratified by age and sex	14/07/1905	Females	30-40	273
Northern Ireland	Asthma	stratified by age and sex	14/07/1905	Females	40-50	322
Northern Ireland	Asthma	stratified by age and sex	14/07/1905	Females	50-60	308
Northern Ireland	Asthma	stratified by age and sex	14/07/1905	Females	60-70	216
Northern Ireland	Asthma	stratified by age and sex	14/07/1905	Females	70+	157
Northern Ireland	Asthma	age dic	26/06/1905	Males	<20	250568
Northern Ireland	Asthma	age dic	26/06/1905	Females	<20	238057
Northern Ireland	Asthma	age dic	26/06/1905	Males	>=20	587683
Northern Ireland	Asthma	age dic	26/06/1905	Females	>=20	637734
Northern Ireland	Asthma	age dic	27/06/1905	Males	<20	248802
Northern Ireland	Asthma	age dic	27/06/1905	Females	<20	236557
Northern Ireland	Asthma	age dic	27/06/1905	Males	>=20	596499
Northern Ireland	Asthma	age dic	27/06/1905	Females	>=20	645875
Northern Ireland	Asthma	age dic	28/06/1905	Males	<20	247337
Northern Ireland	Asthma	age dic	28/06/1905	Females	<20	235307
Northern Ireland	Asthma	age dic	28/06/1905	Males	>=20	605773
Northern Ireland	Asthma	age dic	28/06/1905	Females	>=20	654696
Northern Ireland	Asthma	age dic	29/06/1905	Males	<20	246808
Northern Ireland	Asthma	age dic	29/06/1905	Females	<20	234831
Northern Ireland	Asthma	age dic	29/06/1905	Males	>=20	615448
Northern Ireland	Asthma	age dic	29/06/1905	Females	>=20	664596
Northern Ireland	Asthma	age dic	30/06/1905	Males	<20	246862
Northern Ireland	Asthma	age dic	30/06/1905	Females	<20	234925
Northern Ireland	Asthma	age dic	30/06/1905	Males	>=20	624136
Northern Ireland	Asthma	age dic	30/06/1905	Females	>=20	673229
Northern Ireland	Asthma	age dic	01/07/1905	Males	<20	246937
Northern Ireland	Asthma	age dic	01/07/1905	Females	<20	234887
Northern Ireland	Asthma	age dic	01/07/1905	Males	>=20	631625
Northern Ireland	Asthma	age dic	01/07/1905	Females	>=20	679884
Northern Ireland	Asthma	age dic	02/07/1905	Males	<20	246731

Northern Ireland	Asthma	age dic	02/07/1905	Females	<20	234656
Northern Ireland	Asthma	age dic	02/07/1905	Males	>=20	637804
Northern Ireland	Asthma	age dic	02/07/1905	Females	>=20	685642
Northern Ireland	Asthma	age dic	03/07/1905	Males	<20	246533
Northern Ireland	Asthma	age dic	03/07/1905	Females	<20	234738
Northern Ireland	Asthma	age dic	03/07/1905	Males	>=20	642789
Northern Ireland	Asthma	age dic	03/07/1905	Females	>=20	690258
Northern Ireland	Asthma	age dic	04/07/1905	Males	<20	246399
Northern Ireland	Asthma	age dic	04/07/1905	Females	<20	234789
Northern Ireland	Asthma	age dic	04/07/1905	Males	>=20	648468
Northern Ireland	Asthma	age dic	04/07/1905	Females	>=20	694947
Northern Ireland	Asthma	age dic	05/07/1905	Males	<20	246118
Northern Ireland	Asthma	age dic	05/07/1905	Females	<20	234575
Northern Ireland	Asthma	age dic	05/07/1905	Males	>=20	651678
Northern Ireland	Asthma	age dic	05/07/1905	Females	>=20	699306
Northern Ireland	Asthma	age dic	06/07/1905	Males	<20	246306
Northern Ireland	Asthma	age dic	06/07/1905	Females	<20	234670
Northern Ireland	Asthma	age dic	06/07/1905	Males	>=20	657281
Northern Ireland	Asthma	age dic	06/07/1905	Females	>=20	704929
Northern Ireland	Asthma	age dic	07/07/1905	Males	<20	246524
Northern Ireland	Asthma	age dic	07/07/1905	Females	<20	235147
Northern Ireland	Asthma	age dic	07/07/1905	Males	>=20	663646
Northern Ireland	Asthma	age dic	07/07/1905	Females	>=20	709626
Northern Ireland	Asthma	age dic	08/07/1905	Males	<20	247034
Northern Ireland	Asthma	age dic	08/07/1905	Females	<20	235429
Northern Ireland	Asthma	age dic	08/07/1905	Males	>=20	669340
Northern Ireland	Asthma	age dic	08/07/1905	Females	>=20	714239
Northern Ireland	Asthma	age dic	09/07/1905	Males	<20	246631
Northern Ireland	Asthma	age dic	09/07/1905	Females	<20	234786
Northern Ireland	Asthma	age dic	09/07/1905	Males	>=20	674832
Northern Ireland	Asthma	age dic	09/07/1905	Females	>=20	718929
Northern Ireland	Asthma	age dic	10/07/1905	Males	<20	246701
Northern Ireland	Asthma	age dic	10/07/1905	Females	<20	234894
Northern Ireland	Asthma	age dic	10/07/1905	Males	>=20	680697
Northern Ireland	Asthma	age dic	10/07/1905	Females	>=20	723967
Northern Ireland	Asthma	age dic	11/07/1905	Males	<20	246503
Northern Ireland	Asthma	age dic	11/07/1905	Females	<20	234850
Northern Ireland	Asthma	age dic	11/07/1905	Males	>=20	687347
Northern Ireland	Asthma	age dic	11/07/1905	Females	>=20	729819
Northern Ireland	Asthma	age dic	12/07/1905	Males	<20	245631
Northern Ireland	Asthma	age dic	12/07/1905	Females	<20	234050
Northern Ireland	Asthma	age dic	12/07/1905	Males	>=20	689483
Northern Ireland	Asthma	age dic	12/07/1905	Females	>=20	731359
Northern Ireland	Asthma	age dic	13/07/1905	Males	<20	245249
Northern Ireland	Asthma	age dic	13/07/1905	Females	<20	233227
Northern Ireland	Asthma	age dic	13/07/1905	Males	>=20	691565
Northern Ireland	Asthma	age dic	13/07/1905	Females	>=20	734523
Northern Ireland	Asthma	age dic	14/07/1905	Males	<20	246734
Northern Ireland	Asthma	age dic	14/07/1905	Females	<20	234309
Northern Ireland	Asthma	age dic	14/07/1905	Males	>=20	693213
Northern Ireland	Asthma	age dic	14/07/1905	Females	>=20	736287
Northern Ireland	COPD	all	26/06/1905	All persons		917
Northern Ireland	COPD	all	27/06/1905	All persons		917
Northern Ireland	COPD	all	28/06/1905	All persons		1072
Northern Ireland	COPD	all	29/06/1905	All persons		1084
Northern Ireland	COPD	all	30/06/1905	All persons		1323
Northern Ireland	COPD	all	01/07/1905	All persons		1309
Northern Ireland	COPD	all	02/07/1905	All persons		1556
Northern Ireland	COPD	all	03/07/1905	All persons		1621
Northern Ireland	COPD	all	04/07/1905	All persons		1833
Northern Ireland	COPD	all	05/07/1905	All persons		2031
Northern Ireland	COPD	all	06/07/1905	All persons		2094
Northern Ireland	COPD	all	07/07/1905	All persons		2457

Northern Ireland	COPD	all	08/07/1905	All persons		2750
Northern Ireland	COPD	all	09/07/1905	All persons		2852
Northern Ireland	COPD	all	10/07/1905	All persons		3029
Northern Ireland	COPD	all	11/07/1905	All persons		3158
Northern Ireland	COPD	all	12/07/1905	All persons		1380
Northern Ireland	COPD	all	13/07/1905	All persons		1395
Northern Ireland	COPD	all	14/07/1905	All persons		2149
Northern Ireland	COPD	stratified by sex	26/06/1905	Males		424
Northern Ireland	COPD	stratified by sex	26/06/1905	Females		493
Northern Ireland	COPD	stratified by sex	27/06/1905	Males		439
Northern Ireland	COPD	stratified by sex	27/06/1905	Females		478
Northern Ireland	COPD	stratified by sex	28/06/1905	Males		514
Northern Ireland	COPD	stratified by sex	28/06/1905	Females		558
Northern Ireland	COPD	stratified by sex	29/06/1905	Males		552
Northern Ireland	COPD	stratified by sex	29/06/1905	Females		532
Northern Ireland	COPD	stratified by sex	30/06/1905	Males		647
Northern Ireland	COPD	stratified by sex	30/06/1905	Females		676
Northern Ireland	COPD	stratified by sex	01/07/1905	Males		618
Northern Ireland	COPD	stratified by sex	01/07/1905	Females		691
Northern Ireland	COPD	stratified by sex	02/07/1905	Males		766
Northern Ireland	COPD	stratified by sex	02/07/1905	Females		790
Northern Ireland	COPD	stratified by sex	03/07/1905	Males		779
Northern Ireland	COPD	stratified by sex	03/07/1905	Females		842
Northern Ireland	COPD	stratified by sex	04/07/1905	Males		894
Northern Ireland	COPD	stratified by sex	04/07/1905	Females		939
Northern Ireland	COPD	stratified by sex	05/07/1905	Males		1000
Northern Ireland	COPD	stratified by sex	05/07/1905	Females		1031
Northern Ireland	COPD	stratified by sex	06/07/1905	Males		1022
Northern Ireland	COPD	stratified by sex	06/07/1905	Females		1072
Northern Ireland	COPD	stratified by sex	07/07/1905	Males		1240
Northern Ireland	COPD	stratified by sex	07/07/1905	Females		1217
Northern Ireland	COPD	stratified by sex	08/07/1905	Males		1426
Northern Ireland	COPD	stratified by sex	08/07/1905	Females		1324
Northern Ireland	COPD	stratified by sex	09/07/1905	Males		1455
Northern Ireland	COPD	stratified by sex	09/07/1905	Females		1397
Northern Ireland	COPD	stratified by sex	10/07/1905	Males		1538
Northern Ireland	COPD	stratified by sex	10/07/1905	Females		1491
Northern Ireland	COPD	stratified by sex	11/07/1905	Males		1608
Northern Ireland	COPD	stratified by sex	11/07/1905	Females		1550
Northern Ireland	COPD	stratified by sex	12/07/1905	Males		714
Northern Ireland	COPD	stratified by sex	12/07/1905	Females		666
Northern Ireland	COPD	stratified by sex	13/07/1905	Males		708
Northern Ireland	COPD	stratified by sex	13/07/1905	Females		687
Northern Ireland	COPD	stratified by sex	14/07/1905	Males		1085
Northern Ireland	COPD	stratified by sex	14/07/1905	Females		1064
Northern Ireland	COPD	stratified by age	26/06/1905	All persons	40-50	214
Northern Ireland	COPD	stratified by age	26/06/1905	All persons	50-60	366
Northern Ireland	COPD	stratified by age	26/06/1905	All persons	60-70	277
Northern Ireland	COPD	stratified by age	26/06/1905	All persons	70+	60
Northern Ireland	COPD	stratified by age	27/06/1905	All persons	40-50	195
Northern Ireland	COPD	stratified by age	27/06/1905	All persons	50-60	351
Northern Ireland	COPD	stratified by age	27/06/1905	All persons	60-70	289
Northern Ireland	COPD	stratified by age	27/06/1905	All persons	70+	82
Northern Ireland	COPD	stratified by age	28/06/1905	All persons	40-50	210
Northern Ireland	COPD	stratified by age	28/06/1905	All persons	50-60	436
Northern Ireland	COPD	stratified by age	28/06/1905	All persons	60-70	351
Northern Ireland	COPD	stratified by age	28/06/1905	All persons	70+	75
Northern Ireland	COPD	stratified by age	29/06/1905	All persons	40-50	220
Northern Ireland	COPD	stratified by age	29/06/1905	All persons	50-60	401
Northern Ireland	COPD	stratified by age	29/06/1905	All persons	60-70	360
Northern Ireland	COPD	stratified by age	29/06/1905	All persons	70+	103
Northern Ireland	COPD	stratified by age	30/06/1905	All persons	40-50	260
Northern Ireland	COPD	stratified by age	30/06/1905	All persons	50-60	491

Northern Ireland	COPD	stratified by age	30/06/1905	All persons	60-70	425
Northern Ireland	COPD	stratified by age	30/06/1905	All persons	70+	147
Northern Ireland	COPD	stratified by age	01/07/1905	All persons	40-50	247
Northern Ireland	COPD	stratified by age	01/07/1905	All persons	50-60	467
Northern Ireland	COPD	stratified by age	01/07/1905	All persons	60-70	458
Northern Ireland	COPD	stratified by age	01/07/1905	All persons	70+	137
Northern Ireland	COPD	stratified by age	02/07/1905	All persons	40-50	286
Northern Ireland	COPD	stratified by age	02/07/1905	All persons	50-60	547
Northern Ireland	COPD	stratified by age	02/07/1905	All persons	60-70	548
Northern Ireland	COPD	stratified by age	02/07/1905	All persons	70+	175
Northern Ireland	COPD	stratified by age	03/07/1905	All persons	40-50	299
Northern Ireland	COPD	stratified by age	03/07/1905	All persons	50-60	521
Northern Ireland	COPD	stratified by age	03/07/1905	All persons	60-70	581
Northern Ireland	COPD	stratified by age	03/07/1905	All persons	70+	220
Northern Ireland	COPD	stratified by age	04/07/1905	All persons	40-50	314
Northern Ireland	COPD	stratified by age	04/07/1905	All persons	50-60	623
Northern Ireland	COPD	stratified by age	04/07/1905	All persons	60-70	640
Northern Ireland	COPD	stratified by age	04/07/1905	All persons	70+	256
Northern Ireland	COPD	stratified by age	05/07/1905	All persons	40-50	298
Northern Ireland	COPD	stratified by age	05/07/1905	All persons	50-60	663
Northern Ireland	COPD	stratified by age	05/07/1905	All persons	60-70	685
Northern Ireland	COPD	stratified by age	05/07/1905	All persons	70+	385
Northern Ireland	COPD	stratified by age	06/07/1905	All persons	40-50	270
Northern Ireland	COPD	stratified by age	06/07/1905	All persons	50-60	664
Northern Ireland	COPD	stratified by age	06/07/1905	All persons	60-70	720
Northern Ireland	COPD	stratified by age	06/07/1905	All persons	70+	440
Northern Ireland	COPD	stratified by age	07/07/1905	All persons	40-50	266
Northern Ireland	COPD	stratified by age	07/07/1905	All persons	50-60	772
Northern Ireland	COPD	stratified by age	07/07/1905	All persons	60-70	865
Northern Ireland	COPD	stratified by age	07/07/1905	All persons	70+	554
Northern Ireland	COPD	stratified by age	08/07/1905	All persons	40-50	315
Northern Ireland	COPD	stratified by age	08/07/1905	All persons	50-60	796
Northern Ireland	COPD	stratified by age	08/07/1905	All persons	60-70	986
Northern Ireland	COPD	stratified by age	08/07/1905	All persons	70+	653
Northern Ireland	COPD	stratified by age	09/07/1905	All persons	40-50	299
Northern Ireland	COPD	stratified by age	09/07/1905	All persons	50-60	831
Northern Ireland	COPD	stratified by age	09/07/1905	All persons	60-70	955
Northern Ireland	COPD	stratified by age	09/07/1905	All persons	70+	767
Northern Ireland	COPD	stratified by age	10/07/1905	All persons	40-50	319
Northern Ireland	COPD	stratified by age	10/07/1905	All persons	50-60	874
Northern Ireland	COPD	stratified by age	10/07/1905	All persons	60-70	981
Northern Ireland	COPD	stratified by age	10/07/1905	All persons	70+	855
Northern Ireland	COPD	stratified by age	11/07/1905	All persons	40-50	263
Northern Ireland	COPD	stratified by age	11/07/1905	All persons	50-60	909
Northern Ireland	COPD	stratified by age	11/07/1905	All persons	60-70	1078
Northern Ireland	COPD	stratified by age	11/07/1905	All persons	70+	908
Northern Ireland	COPD	stratified by age	12/07/1905	All persons	40-50	120
Northern Ireland	COPD	stratified by age	12/07/1905	All persons	50-60	389
Northern Ireland	COPD	stratified by age	12/07/1905	All persons	60-70	462
Northern Ireland	COPD	stratified by age	12/07/1905	All persons	70+	409
Northern Ireland	COPD	stratified by age	13/07/1905	All persons	40-50	122
Northern Ireland	COPD	stratified by age	13/07/1905	All persons	50-60	355
Northern Ireland	COPD	stratified by age	13/07/1905	All persons	60-70	450
Northern Ireland	COPD	stratified by age	13/07/1905	All persons	70+	468
Northern Ireland	COPD	stratified by age	14/07/1905	All persons	40-50	158
Northern Ireland	COPD	stratified by age	14/07/1905	All persons	50-60	526
Northern Ireland	COPD	stratified by age	14/07/1905	All persons	60-70	662
Northern Ireland	COPD	stratified by age	14/07/1905	All persons	70+	773
Northern Ireland	ILD	all	26/06/1905	All persons		128
Northern Ireland	ILD	all	27/06/1905	All persons		117
Northern Ireland	ILD	all	28/06/1905	All persons		120
Northern Ireland	ILD	all	29/06/1905	All persons		123
Northern Ireland	ILD	all	30/06/1905	All persons		115

Northern Ireland	ILD	all	01/07/1905	All persons		132
Northern Ireland	ILD	all	02/07/1905	All persons		155
Northern Ireland	ILD	all	03/07/1905	All persons		156
Northern Ireland	ILD	all	04/07/1905	All persons		222
Northern Ireland	ILD	all	05/07/1905	All persons		208
Northern Ireland	ILD	all	06/07/1905	All persons		289
Northern Ireland	ILD	all	07/07/1905	All persons		236
Northern Ireland	ILD	all	08/07/1905	All persons		264
Northern Ireland	ILD	all	09/07/1905	All persons		337
Northern Ireland	ILD	all	10/07/1905	All persons		367
Northern Ireland	ILD	all	11/07/1905	All persons		433
Northern Ireland	ILD	all	12/07/1905	All persons		381
Northern Ireland	ILD	all	13/07/1905	All persons		643
Northern Ireland	ILD	all	14/07/1905	All persons		670
Northern Ireland	ILD	stratified by sex	26/06/1905	Males		74
Northern Ireland	ILD	stratified by sex	26/06/1905	Females		54
Northern Ireland	ILD	stratified by sex	27/06/1905	Males		77
Northern Ireland	ILD	stratified by sex	27/06/1905	Females		40
Northern Ireland	ILD	stratified by sex	28/06/1905	Males		84
Northern Ireland	ILD	stratified by sex	28/06/1905	Females		36
Northern Ireland	ILD	stratified by sex	29/06/1905	Males		72
Northern Ireland	ILD	stratified by sex	29/06/1905	Females		51
Northern Ireland	ILD	stratified by sex	30/06/1905	Males		67
Northern Ireland	ILD	stratified by sex	30/06/1905	Females		48
Northern Ireland	ILD	stratified by sex	01/07/1905	Males		77
Northern Ireland	ILD	stratified by sex	01/07/1905	Females		55
Northern Ireland	ILD	stratified by sex	02/07/1905	Males		92
Northern Ireland	ILD	stratified by sex	02/07/1905	Females		63
Northern Ireland	ILD	stratified by sex	03/07/1905	Males		99
Northern Ireland	ILD	stratified by sex	03/07/1905	Females		57
Northern Ireland	ILD	stratified by sex	04/07/1905	Males		147
Northern Ireland	ILD	stratified by sex	04/07/1905	Females		75
Northern Ireland	ILD	stratified by sex	05/07/1905	Males		123
Northern Ireland	ILD	stratified by sex	05/07/1905	Females		85
Northern Ireland	ILD	stratified by sex	06/07/1905	Males		168
Northern Ireland	ILD	stratified by sex	06/07/1905	Females		121
Northern Ireland	ILD	stratified by sex	07/07/1905	Males		135
Northern Ireland	ILD	stratified by sex	07/07/1905	Females		101
Northern Ireland	ILD	stratified by sex	08/07/1905	Males		145
Northern Ireland	ILD	stratified by sex	08/07/1905	Females		119
Northern Ireland	ILD	stratified by sex	09/07/1905	Males		188
Northern Ireland	ILD	stratified by sex	09/07/1905	Females		149
Northern Ireland	ILD	stratified by sex	10/07/1905	Males		210
Northern Ireland	ILD	stratified by sex	10/07/1905	Females		157
Northern Ireland	ILD	stratified by sex	11/07/1905	Males		251
Northern Ireland	ILD	stratified by sex	11/07/1905	Females		182
Northern Ireland	ILD	stratified by sex	12/07/1905	Males		229
Northern Ireland	ILD	stratified by sex	12/07/1905	Females		152
Northern Ireland	ILD	stratified by sex	13/07/1905	Males		369
Northern Ireland	ILD	stratified by sex	13/07/1905	Females		274
Northern Ireland	ILD	stratified by sex	14/07/1905	Males		391
Northern Ireland	ILD	stratified by sex	14/07/1905	Females		279
Northern Ireland	ILD	stratified by age	29/06/1905		40-50	23
Northern Ireland	ILD	stratified by age	29/06/1905		50-60	38
Northern Ireland	ILD	stratified by age	29/06/1905		60-70	47
Northern Ireland	ILD	stratified by age	29/06/1905		70+	15
Northern Ireland	ILD	stratified by age	30/06/1905		40-50	28
Northern Ireland	ILD	stratified by age	30/06/1905		50-60	37
Northern Ireland	ILD	stratified by age	30/06/1905		60-70	33
Northern Ireland	ILD	stratified by age	30/06/1905		70+	17
Northern Ireland	ILD	stratified by age	01/07/1905		40-50	29
Northern Ireland	ILD	stratified by age	01/07/1905		50-60	32
Northern Ireland	ILD	stratified by age	01/07/1905		60-70	52

Northern Ireland	ILD	stratified by age	01/07/1905	70+	19
Northern Ireland	ILD	stratified by age	02/07/1905	40-50	32
Northern Ireland	ILD	stratified by age	02/07/1905	50-60	32
Northern Ireland	ILD	stratified by age	02/07/1905	60-70	59
Northern Ireland	ILD	stratified by age	02/07/1905	70+	32
Northern Ireland	ILD	stratified by age	03/07/1905	40-50	25
Northern Ireland	ILD	stratified by age	03/07/1905	50-60	34
Northern Ireland	ILD	stratified by age	03/07/1905	60-70	58
Northern Ireland	ILD	stratified by age	03/07/1905	70+	39
Northern Ireland	ILD	stratified by age	04/07/1905	40-50	31
Northern Ireland	ILD	stratified by age	04/07/1905	50-60	50
Northern Ireland	ILD	stratified by age	04/07/1905	60-70	74
Northern Ireland	ILD	stratified by age	04/07/1905	70+	67
Northern Ireland	ILD	stratified by age	05/07/1905	40-50	25
Northern Ireland	ILD	stratified by age	05/07/1905	50-60	52
Northern Ireland	ILD	stratified by age	05/07/1905	60-70	65
Northern Ireland	ILD	stratified by age	05/07/1905	70+	66
Northern Ireland	ILD	stratified by age	06/07/1905	40-50	38
Northern Ireland	ILD	stratified by age	06/07/1905	50-60	43
Northern Ireland	ILD	stratified by age	06/07/1905	60-70	88
Northern Ireland	ILD	stratified by age	06/07/1905	70+	120
Northern Ireland	ILD	stratified by age	07/07/1905	40-50	30
Northern Ireland	ILD	stratified by age	07/07/1905	50-60	47
Northern Ireland	ILD	stratified by age	07/07/1905	60-70	75
Northern Ireland	ILD	stratified by age	07/07/1905	70+	84
Northern Ireland	ILD	stratified by age	08/07/1905	40-50	31
Northern Ireland	ILD	stratified by age	08/07/1905	50-60	49
Northern Ireland	ILD	stratified by age	08/07/1905	60-70	81
Northern Ireland	ILD	stratified by age	08/07/1905	70+	103
Northern Ireland	ILD	stratified by age	09/07/1905	40-50	30
Northern Ireland	ILD	stratified by age	09/07/1905	50-60	55
Northern Ireland	ILD	stratified by age	09/07/1905	60-70	105
Northern Ireland	ILD	stratified by age	09/07/1905	70+	147
Northern Ireland	ILD	stratified by age	10/07/1905	40-50	26
Northern Ireland	ILD	stratified by age	10/07/1905	50-60	66
Northern Ireland	ILD	stratified by age	10/07/1905	60-70	105
Northern Ireland	ILD	stratified by age	10/07/1905	70+	170
Northern Ireland	ILD	stratified by age	11/07/1905	40-50	30
Northern Ireland	ILD	stratified by age	11/07/1905	50-60	90
Northern Ireland	ILD	stratified by age	11/07/1905	60-70	115
Northern Ireland	ILD	stratified by age	11/07/1905	70+	198
Northern Ireland	ILD	stratified by age	12/07/1905	40-50	25
Northern Ireland	ILD	stratified by age	12/07/1905	50-60	69
Northern Ireland	ILD	stratified by age	12/07/1905	60-70	107
Northern Ireland	ILD	stratified by age	12/07/1905	70+	180
Northern Ireland	ILD	stratified by age	13/07/1905	40-50	78
Northern Ireland	ILD	stratified by age	13/07/1905	50-60	115
Northern Ireland	ILD	stratified by age	13/07/1905	60-70	145
Northern Ireland	ILD	stratified by age	13/07/1905	70+	305
Northern Ireland	ILD	stratified by age	14/07/1905	40-50	37
Northern Ireland	ILD	stratified by age	14/07/1905	50-60	96
Northern Ireland	ILD	stratified by age	14/07/1905	60-70	145
Northern Ireland	ILD	stratified by age	14/07/1905	70+	392

overall_denom_persontime	crude_rate	crude_lb	crude_ub	adj_rate	adj_lb	adj_ub
4488685.5	4.8370509	4.7729225	4.9018254	4.7049999	4.4091468	5.0154839
4633369	4.4423399	4.381856	4.5034499	4.3449998	4.0608773	4.6437593
4628480	5.1077242	5.04282	5.1732554	5.0300002	4.7239366	5.3506885
4325350	4.5036817	4.4406567	4.5673776	4.4299998	4.1430655	4.7315702
4618892	4.88234	4.8188224	4.946485	4.7849998	4.4866018	5.0980277
4464186	2.4212701	2.3758371	2.4673536	2.395	2.1852949	2.6193991
4607894.5	1.8958768	1.8563268	1.9360571	1.885	1.699501	2.0852211
4455654	2.3751845	2.3301451	2.4208755	2.345	2.1375458	2.5671504
4600766	2.0529189	2.0117235	2.0947459	2.04	1.8468308	2.2478814
4597302	1.8656595	1.8263826	1.9055681	1.85	1.6662762	2.0484481
4445585.5	3.158639	3.1066089	3.2113223	3.0699999	2.8319399	3.3227282
4589658.5	3.1740923	3.1227562	3.2260609	3.0999999	2.8607562	3.3539109
4437183	3.2133	3.1607702	3.266484	3.155	2.9136007	3.4110646
4580339.5	2.7148643	2.6673543	2.7630079	2.675	2.4531019	2.9115803
4574801.5	2.7769511	2.7288697	2.8256671	2.7550001	2.5297368	2.9949422
4127115.8	2.7799075	2.72927	2.8312485	2.76	2.534528	3.0001507
4565100.5	3.3699148	3.3168712	3.4235945	3.325	3.0770547	3.5876057
4414112.5	2.8846116	2.8347228	2.935158	2.845	2.616009	3.0886667
4557356	2.8060129	2.7575874	2.8550754	2.7850001	2.5584874	3.0261903
4406718	3.3256042	3.2719769	3.3798902	3.2950001	3.0481975	3.5564637
4549653.5	2.8173573	2.7687929	2.86656	2.8150001	2.587245	3.0574317
4545666.5	2.3325512	2.2883618	2.3773794	2.345	2.1375458	2.5671504
4395230.5	3.3049462	3.2514167	3.3591359	3.3150001	3.0674353	3.5772257
4537320.5	3.3995836	3.3461437	3.4536631	3.415	3.1636569	3.6810009
4386446.5	3.9373558	3.8788509	3.9965219	3.9649999	3.6938004	4.250844
4527824.5	3.2295864	3.1774507	3.2823629	3.2550001	3.0097294	3.5149331
4523122.5	3.2528415	3.2004905	3.3058341	3.29	3.0433886	3.5512729
4081391.3	3.3221514	3.2664657	3.3785486	3.385	3.1347847	3.6498742
4514416.5	3.4334447	3.3796029	3.4879296	3.51	3.2551186	3.7795367
4364511	2.8539279	2.8040264	2.9044948	2.925	2.6927447	3.1719282
4505730.5	3.3264306	3.273387	3.3801186	3.425	3.1732824	3.6913755
4356470.5	3.4420066	3.3871326	3.4975467	3.5050001	3.2503037	3.7743518
4497420.5	2.9181173	2.8684032	2.9684765	2.9949999	2.7599249	3.2447457
4493290.5	2.6966875	2.6488831	2.7451379	2.7850001	2.5584874	3.0261903
4344585.5	3.1388495	3.0863862	3.1919808	3.2550001	3.0097294	3.5149331
4484979	3.4620006	3.407758	3.5168903	3.5899999	3.332176	3.8624773
4335935.5	4.079627	4.0197263	4.1401973	4.2449999	3.9642215	4.5404172
4475420	3.4953144	3.4407523	3.550525	3.6199999	3.3610811	3.8935716
4469820	3.9605622	3.9024324	4.019341	4.105	3.8289702	4.3956714
4032992.5	4.0416636	3.9798529	4.1041942	4.2150002	3.9352324	4.509407
4460558.5	3.9622841	3.9040816	4.0211372	4.1500001	3.8724351	4.4422054
4312383	3.0938811	3.0416031	3.146832	3.2249999	2.9808846	3.483779
4452047.5	3.3447533	3.2912445	3.3989139	3.5150001	3.2599339	3.7847214
4161140	4.45575	4.3918419	4.5203557	4.5650001	4.2736535	4.8709793
542253.81	9.2373714	8.9833107	9.4967957	8.9767447	7.7549057	10.336447
534387.88	7.7340827	7.5000706	7.9735394	7.6744184	6.548089	8.9388733
557517.38	7.9262104	7.6942177	8.1634207	7.7209301	6.5910559	8.9889193
549299.63	7.1145144	6.8931866	7.3411398	7.0697675	5.9905381	8.2872667
554691.88	8.3740187	8.1349125	8.6183681	8.139535	6.9782262	9.4388685
546467.69	7.6454659	7.4153748	7.8808808	7.6279068	6.5051332	8.8888168
516506.31	7.4461818	7.2126913	7.685307	7.2558141	6.1618848	8.4879665
508735.81	7.192338	6.9611621	7.4292355	7.1627908	6.0761876	8.387641
549429.44	7.5223489	7.2947435	7.75525	7.395349	6.2905183	8.6383696
541016	6.7354755	6.5185423	6.9577885	6.6976743	5.648438	7.8852797
529341.81	3.1397481	2.9905975	3.2944119	3.0697675	2.3741579	3.9054968
521094.09	2.8747208	2.7309713	3.0240726	2.8837209	2.2109323	3.6968
544505.06	2.5068638	2.3756237	2.6434679	2.4651163	1.8465413	3.2244327
535877.06	2.1553452	2.0328212	2.2833238	2.139535	1.5664084	2.8538396
524612.13	3.3415163	3.1869066	3.5016878	3.3023255	2.5791438	4.1654344
516158.69	2.5767269	2.4400899	2.7190232	2.5581396	1.9271408	3.3297703
539614.63	2.6593053	2.5234778	2.8005443	2.6046512	1.9675255	3.3823562
530733.31	2.1611607	2.0378847	2.2899444	2.139535	1.5664084	2.8538396

537131.56	2.5598943	2.4263597	2.6988664	2.5116279	1.8868122	3.2771297
528105.69	2.1472974	2.0241225	2.2760079	2.139535	1.5664084	2.8538396
517433.69	6.9574132	6.7319779	7.1884737	6.8372092	5.7766304	8.0361195
508607.47	5.1434555	4.9482255	5.3444142	5.1162791	4.2049594	6.1665001
532100.25	6.0063868	5.7999358	6.2183104	5.9069767	4.9243803	7.0281816
522913.78	4.983613	4.7940912	5.1787066	4.9767442	4.0785618	6.013886
512531.19	5.962564	5.7530184	6.1777911	5.860465	4.8819242	6.9776301
503568.78	4.5793147	4.3942986	4.7701192	4.5581393	3.7005179	5.5549121
527145.88	4.8089156	4.6235185	4.9998398	4.7441859	3.8683167	5.7591181
517844.28	3.8679581	3.7004039	4.0411439	3.860465	3.0748389	4.7856274
524589.94	3.6466577	3.4850581	3.8138173	3.627907	2.8677099	4.527792
515328.44	3.1630313	3.0113246	3.3204014	3.1627908	2.4560308	4.0095906
471909.94	3.1679773	3.009407	3.3327343	3.1627908	2.4560308	4.0095906
463509.41	2.7572255	2.6081131	2.9126415	2.7441862	2.0890017	3.5398002
520160.34	4.6447215	4.4613414	4.8337045	4.604651	3.7424333	5.6059976
510796.56	3.9467769	3.7763543	4.1229081	3.9534883	3.1579061	4.8885498
501179.63	4.2799025	4.1006789	4.4649434	4.2790699	3.4495342	5.2478991
492109.72	3.6048872	3.43907	3.776633	3.627907	2.8677099	4.527792
515439.19	4.9705186	4.7798939	5.1667957	5.0232558	4.1206741	6.0647774
506044	4.1063623	3.9316852	4.2868009	4.139535	3.3243864	5.0940537
496529.03	6.3178582	6.0986862	6.5428944	6.4186049	5.3924146	7.5832434
487419.06	5.1208501	4.921906	5.3257728	5.2093024	4.2893233	6.2681441
510574.31	5.529068	5.3269696	5.7368708	5.6744184	4.7122617	6.7752638
501187.28	4.4234962	4.2412605	4.6115479	4.5116277	3.6586263	5.5038033
508157.91	4.0361471	3.8633428	4.2146893	4.139535	3.3243864	5.0940537
498741.41	3.3524387	3.1936555	3.5170732	3.4418604	2.7026007	4.3209405
489421.06	7.6457682	7.4027367	7.8947463	7.860465	6.7200198	9.1389942
480275.31	5.9007821	5.6855121	6.122117	6.0465117	5.0518446	7.1797414
503123.66	7.8449898	7.6021366	8.093627	8.0930233	6.9351664	9.3889151
493708.5	6.5159907	6.2927489	6.7451296	6.6976743	5.648438	7.8852797
484541.63	9.1963205	8.9282646	9.4703798	9.5348835	8.274251	10.933285
475418.19	7.4629035	7.2193379	7.7125912	7.7209301	6.5910559	8.9889193
498256.66	7.1669888	6.9338298	7.4059901	7.395349	6.2905183	8.6383696
488834.38	6.2720628	6.0519967	6.498086	6.4651165	5.435051	7.6336169
495733.66	6.9109693	6.6814694	7.1463408	7.1627908	6.0761876	8.387641
486377.56	5.837029	5.6242704	6.0557761	6.0465117	5.0518446	7.1797414
445732.63	7.0916953	6.8466058	7.3433175	7.4418607	6.3334188	8.6884813
437285.03	6.2293468	5.9975896	6.4677653	6.5116277	5.4777012	7.6839762
491103.44	7.3609748	7.1229544	7.6049218	7.7674417	6.6340332	9.0389547
481733.31	6.418489	6.1942258	6.648797	6.7906976	5.7338867	7.9858522
473018	5.5875254	5.3765154	5.8046942	5.9069767	4.9243803	7.0281816
463949.19	5.2656627	5.0589075	5.4786992	5.5348835	4.5851922	6.6233134
486338.41	7.2500958	7.0127449	7.4934316	7.7209301	6.5910559	8.9889193
476998.84	6.297709	6.0744948	6.5270286	6.6976743	5.648438	7.8852797
468379.78	7.1202903	6.8806624	7.3661337	7.4418607	6.3334188	8.6884813
459348.72	5.9954453	5.7735968	6.2236347	6.2790699	5.2645907	7.4320388
481505.44	6.0643139	5.8463302	6.2883458	6.3720932	5.3497925	7.532856
472194.53	5.3177238	5.111743	5.5298753	5.5813951	4.6275315	6.6739807
479129.06	4.8358579	4.6409364	5.0368624	5.2093024	4.2893233	6.2681441
469773.16	4.3467789	4.1602697	4.539495	4.6511626	3.7843716	5.6570601
461336.34	7.049087	6.8088732	7.2956119	7.6279068	6.5051332	8.8888168
452296.09	5.8700485	5.6488662	6.097672	6.2325583	5.2220116	7.381609
474184.31	7.6573601	7.4102979	7.9105601	8.2325583	7.0643744	9.5387478
464845.94	6.5527086	6.3220487	6.7896328	6.9767442	5.9049377	8.1868448
456620.16	8.7906761	8.5208111	9.0669136	9.4883718	8.2309284	10.883593
447562.38	7.6369243	7.3830233	7.8973298	8.1860466	7.0212955	9.4888134
469396.47	7.347733	7.104538	7.5971303	7.860465	6.7200198	9.1389942
460059.13	6.2100716	5.9844232	6.4420505	6.5581393	5.5203648	7.7343221
466928.22	7.9069967	7.6539793	8.1662474	8.4651165	7.2799139	9.7882786
457625.31	7.1084356	6.8662362	7.356997	7.5348835	6.4192538	8.7886705
419736.78	8.3195	8.0458269	8.6001072	8.930233	7.7116809	10.286657
411367.59	7.2830238	7.0245452	7.5485821	7.860465	6.7200198	9.1389942
462272.78	7.8827915	7.6289063	8.1429729	8.5581398	7.3661952	9.8880253

453069.31	7.219646	6.9743295	7.4713883	7.8139534	6.6770215	9.0889797
445088.41	6.1268725	5.8990521	6.361238	6.604651	5.5630426	7.7846546
436180.53	5.6215258	5.4011989	5.8485332	6	5.0093408	7.1292372
457474.81	6.8637657	6.6257687	7.1081281	7.4883723	6.3763309	8.7385817
448306.72	5.9602051	5.7363343	6.1905742	6.4651165	5.435051	7.6336169
425710.25	9.0225687	8.7394648	9.3125086	9.3488369	8.1010065	10.734468
417188.34	6.977664	6.7264657	7.2358432	7.3023257	6.204751	8.5381126
1710726.8	2.8631108	2.7834837	2.9444375	2.9044585	2.5396614	3.3069444
1701317.1	4.5094476	4.4090991	4.6115031	4.5222931	4.064045	5.0180683
1768162.4	2.7621899	2.6852603	2.840764	2.8152866	2.4563241	3.2119505
1758389.9	4.192472	4.0973086	4.2892876	4.2038217	3.7624445	4.6827507
1768616.4	3.4343231	3.3484921	3.5217977	3.4904459	3.0893517	3.9291575
1758703.9	4.9718432	4.8681726	5.0771656	5.0063696	4.5235782	5.5266562
1654724.4	2.94611	2.8639832	3.0299945	2.9936306	2.6230879	3.40185
1645383.4	4.3151035	4.2153091	4.4166646	4.3439488	3.8950727	4.8303661
1769229	3.1635249	3.0811832	3.24751	3.1592357	2.7782452	3.5778816
1759217.6	5.2165236	5.1103349	5.3243632	5.1719747	4.6810617	5.7003727
1711616.5	1.7311121	1.6693358	1.7945899	1.7197453	1.4418943	2.0355265
1702133.9	2.753015	2.6747494	2.8329892	2.7515924	2.3968542	3.1440413
1768638.6	1.4101242	1.3553193	1.4665765	1.4267516	1.1747829	1.7167529
1758873.6	2.116127	2.0486839	2.1852248	2.1146498	1.8051937	2.4619334
1712130.5	1.8187866	1.7554607	1.8838131	1.8089172	1.5236359	2.132103
1702753.1	2.5758286	2.5001554	2.6532099	2.5732484	2.2306061	2.95363
1769989.1	1.527128	1.4700944	1.5858074	1.5286624	1.267413	1.8279055
1760428.8	2.3630607	2.2917917	2.4359825	2.3694267	2.0411341	2.7354946
1770759.1	1.3028311	1.2502046	1.3571035	1.312102	1.0709801	1.5913051
1761305.4	2.1353481	2.0676434	2.2047052	2.1273885	1.8169645	2.4756374
1714298.6	1.7464869	1.6844825	1.81019	1.7452229	1.4652297	2.063139
1705245.8	2.8336091	2.7542701	2.9146538	2.8152866	2.4563241	3.2119505
1771974.5	1.8877246	1.8242891	1.9528033	1.8726115	1.5821344	2.2009754
1762669.9	3.0754483	2.9941185	3.1584277	3.0573249	2.6827302	3.4695876
1715013.6	1.9545034	1.8888918	2.0218124	1.9490446	1.6524481	2.2835081
1706069.4	3.2495747	3.1645925	3.3362613	3.2356689	2.8499489	3.659035
1772241.3	1.741298	1.6803981	1.8038409	1.7452229	1.4652297	2.063139
1763107.9	2.7287042	2.652137	2.806921	2.7133758	2.3611958	3.1032724
1771980.6	2.0096157	1.944147	2.0767269	2	1.6993904	2.338464
1762902.8	3.176579	3.0939195	3.2608879	3.1592357	2.7782452	3.5778816
1599932.4	2.1338401	2.0628562	2.2066436	2.1146498	1.8051937	2.4619334
1591763.6	3.3208449	3.2319186	3.4115977	3.2993631	2.9097443	3.7266204
1771578.5	2.4847896	2.4119229	2.5592985	2.4585986	2.123955	2.8310013
1762565.3	3.7161744	3.6267171	3.807281	3.6687899	3.2572656	4.1179132
1714790.5	2.164696	2.0956128	2.2354763	2.1273885	1.8169645	2.4756374
1706032.9	2.9905636	2.9090605	3.0737715	2.9426751	2.5754049	3.3476288
1772448	1.8561898	1.7932992	1.9207231	1.8471338	1.5587242	2.1734371
1763424.9	2.7548664	2.6779373	2.8334448	2.7261147	2.37308	3.1168642
1715725	2.2258811	2.1558397	2.2976189	2.1910827	1.875862	2.5441146
1707045.1	3.0479569	2.9656942	3.1319232	3.0191083	2.6469398	3.4289501
1773423.6	1.8106221	1.7485317	1.8743542	1.7961783	1.5119472	2.1183176
1764468.5	2.5883148	2.5137858	2.6644921	2.5732484	2.2306061	2.95363
1773835.3	1.5722994	1.5144827	1.6317581	1.5796179	1.3138444	1.8833671
1764932.1	2.3179362	2.2474456	2.3900754	2.3184714	1.9938618	2.680866
1717029.4	1.8532007	1.7893637	1.9187334	1.8471338	1.5587242	2.1734371
1708504.8	2.7907443	2.7120864	2.8711045	2.77707	2.4206364	3.1712108
1774626.1	1.8065777	1.7445781	1.8702179	1.8089172	1.5236359	2.132103
1765862.4	2.8626239	2.7842481	2.9426467	2.8280256	2.4682238	3.2255268
1717467.5	2.046618	1.9795127	2.1154182	2.0382166	1.7346306	2.3796477
1709019	3.3656735	3.2792509	3.4537973	3.3375797	2.9456398	3.7671537
1774744.5	1.7033437	1.6431593	1.7651691	1.7070063	1.4302325	2.0217144
1765989.1	2.810323	2.7326741	2.8896186	2.789809	2.4325304	3.1847928
1774914	1.8451598	1.7825011	1.9094589	1.8471338	1.5587242	2.1734371
1766097.3	2.9290574	2.8497763	3.009985	2.9171975	2.5515742	3.3205078
1603170.9	1.9118361	1.8447453	1.9807434	1.9235669	1.6289966	2.2560105
1595202.8	2.8892879	2.8064699	2.9739294	2.8789809	2.5158415	3.2798126

1775197.8	1.8888036	1.8254069	1.9538404	1.8980892	1.6055586	2.2284999
1766382	3.0797415	2.9984396	3.1626894	3.0700636	2.6946638	3.48313
1718046.9	1.6495476	1.5893693	1.7114211	1.6560509	1.3836262	1.966426
1709497.3	2.6534116	2.5767496	2.7317753	2.6496816	2.3018055	3.0352843
1775649.3	1.790331	1.728631	1.853671	1.8089172	1.5236359	2.132103
1766744	2.9879825	2.907917	3.0696936	2.9808917	2.6111646	3.3882973
1718684.1	2.1574645	2.0885754	2.2280471	2.1528661	1.840515	2.5030367
1710057.6	3.0396636	2.9575856	3.1234422	3.0318472	2.6588681	3.4424975
1776311.8	1.7339299	1.6732292	1.7962699	1.7452229	1.4652297	2.063139
1767408.9	2.6100357	2.5352542	2.6864626	2.611465	2.2661963	2.9944665
1776630.4	1.7336189	1.672929	1.7959477	1.7452229	1.4652297	2.063139
1767758.1	2.6462896	2.5709944	2.7232304	2.6496816	2.3018055	3.0352843
1719766.6	1.7758224	1.7133933	1.8399446	1.7834395	1.500262	2.1045284
1711186.5	2.7326069	2.654839	2.8120744	2.7133758	2.3611958	3.1032724
1777415.4	1.9781532	1.9133019	2.0446422	1.9872612	1.68765	2.3247299
1768533.4	3.0160584	2.9356556	3.0981054	2.9936306	2.6230879	3.40185
1720188.4	2.2852149	2.21433	2.3577914	2.2929935	1.9702408	2.6535366
1711564.9	3.6960328	3.6055083	3.7882557	3.6815286	3.2692692	4.1313863
1777494.8	2.0084448	1.9430962	2.0754309	2.0127389	1.711134	2.352195
1768469.8	3.2610114	3.1773844	3.3462822	3.2484076	2.8619049	3.672555
1777250.1	2.3755801	2.3044574	2.4483397	2.3821657	2.0529585	2.7491457
1768016.1	3.6967988	3.607713	3.7875285	3.6942675	3.2812743	4.1448579
1605188	2.4202774	2.3447633	2.4976041	2.433121	2.10028	2.8037255
1596700	3.7120311	3.6181233	3.8077593	3.7070065	3.2932804	4.1583281
1777381.5	2.3900328	2.3186951	2.4630072	2.4076433	2.0766144	2.7764404
1767835.1	3.6830359	3.5941124	3.7736032	3.6815286	3.2692692	4.1313863
1720205.6	1.9096555	1.8449044	1.9760989	1.910828	1.617276	2.2422569
1710908.5	2.8511169	2.7716622	2.9322712	2.8535032	2.492029	3.2526734
1777982.5	2.0242045	1.9586067	2.091439	2.0382166	1.7346306	2.3796477
1768283.5	3.0990505	3.0175359	3.1822095	3.0955415	2.7185361	3.51021
1663688.3	3.1009414	3.0168951	3.1867359	3.0700636	2.6946638	3.48313
1654552.9	4.0071249	3.9112437	4.1047626	3.9872611	3.5577242	4.4543681
283193.31	9.6894941	9.3303089	10.058966	9.6000004	7.776031	11.723243
280331.44	11.386521	10.994902	11.788528	11.363636	9.4589911	13.53926
260131.64	7.9728866	7.6334114	8.3235703	8	6.416234	9.8562241
252985.31	4.4745679	4.2176709	4.7430205	4.4545455	3.2954972	5.8891454
647877.81	3.5207255	3.3777099	3.6682403	3.5	2.7917371	4.3332381
648762.06	3.3648701	3.2251828	3.5090508	3.3703704	2.7136121	4.1380677
574745.25	3.6363938	3.4821482	3.7957122	3.6785715	3.0025692	4.4613376
571490.88	3.9440699	3.7829106	4.1103296	3.9629629	3.2477438	4.788835
437210.31	4.2245116	4.0340271	4.4216681	4.2173915	3.4200203	5.1448598
531957.5	3.5999117	3.4404659	3.7648401	3.5714285	2.9058568	4.3438139
287728.34	8.6748495	8.3378325	9.0219927	8.6000004	6.87889	10.62093
289367.03	10.0668	9.7045155	10.43915	10.090909	8.3012114	12.152058
269092.19	7.2317224	6.9139509	7.5603318	7.2727275	5.7668204	9.0515385
260629.41	3.7294333	3.4986343	3.9714575	3.7272727	2.6747553	5.0564661
668518.56	3.2085869	3.0742254	3.3473096	3.1666667	2.4949732	3.9635556
670740.5	3.0384328	2.9079351	3.1732779	3.074074	2.4484828	3.8107774
594128.5	3.4908273	3.3421924	3.6443698	3.5357144	2.8736541	4.3046055
590638.38	3.7603381	3.6055608	3.9200509	3.7777777	3.0803263	4.5859647
452304.22	3.8867645	3.7071793	4.0728002	3.9130435	3.146549	4.8097949
550222.13	3.6712446	3.5128748	3.834914	3.6428571	2.9703147	4.4221802
282807.19	8.5853548	8.2472229	8.9337912	8.6000004	6.87889	10.62093
289112.69	11.286257	10.902295	11.680288	11.272727	9.376071	13.440392
269476.5	7.2993379	6.9802928	7.6292048	7.2727275	5.7668204	9.0515385
259763.23	4.4848533	4.2309852	4.7499733	4.4545455	3.2954972	5.8891454
667507.25	3.8920925	3.7438545	4.0446954	3.875	3.1276252	4.7471366
670555.31	3.643249	3.5001974	3.7906463	3.6666667	2.9800858	4.464035
594937.44	4.1584206	3.9961569	4.325582	4.2142859	3.4882758	5.0468402
590402.88	4.4952354	4.3258243	4.6695814	4.5185184	3.7523565	5.3951173
453293.28	4.8666062	4.6656218	5.0740218	4.869565	4.0095849	5.8593521
550624.06	4.4367838	4.2625742	4.6162858	4.3928571	3.6508937	5.2413006
259845.33	7.5044641	7.1750426	7.8451095	7.5	5.8992257	9.4013147

270219.59	10.339739	9.9598627	10.730394	10.363636	8.5487223	12.449893
252439.67	7.1621075	6.8357415	7.5000291	7.181818	5.6859131	8.9506884
242737.56	3.9260509	3.6807125	4.1834426	3.909091	2.8290284	5.2655196
622576.06	3.2991953	3.1580451	3.445029	3.2916667	2.6060436	4.1023989
628105.19	3.289258	3.1489377	3.4342206	3.2962964	2.6471965	4.0563765
556020.25	3.5933943	3.4375412	3.754492	3.6428571	2.9703147	4.4221802
552894.13	3.9175675	3.7543056	4.086102	3.925926	3.2142265	4.7482944
424378.78	4.3428183	4.1467896	4.5457211	4.347826	3.5375648	5.2881217
516133.41	3.5804696	3.4190686	3.7475226	3.5357144	2.8736541	4.3046055
268531.75	7.2989507	6.9793596	7.6294022	7.3000002	5.7220345	9.1786537
288581.59	9.9937077	9.632266	10.365241	10	8.2187834	12.052704
270740.84	6.7296829	6.4241886	7.0459518	6.7272725	5.2823558	8.4454746
262591.28	4.2309098	3.9857507	4.4872022	4.181818	3.0616164	5.5779595
658702.19	4.4906487	4.3302617	4.6554565	4.4583335	3.6537118	5.3874393
677022.88	4.3868532	4.2304873	4.5475206	4.4074073	3.6511717	5.2741199
589435.25	4.5840487	4.412817	4.7602229	4.6428571	3.8790948	5.5130157
596059.94	4.512969	4.3440204	4.6868048	4.5555553	3.7861121	5.4354229
451109.5	4.1253843	3.9400625	4.3171721	4.130435	3.3417685	5.0492425
556116.94	2.8645055	2.7255499	3.0087094	2.8571429	2.2655365	3.5559616
255278.48	3.1142459	2.9015	3.3384652	3.0999999	2.1062996	4.4002028
279200.94	4.2585816	4.0199323	4.507699	4.2727275	3.1394393	5.6818213
262364.59	2.7175922	2.5217519	2.9246035	2.7272727	1.8400794	3.8933513
253591.89	1.8257681	1.6632298	1.9998981	1.8181819	1.1105927	2.8080344
636066.06	2.1994572	2.0856996	2.3178058	2.1666667	1.6181703	2.841295
655277	2.3715162	2.2550588	2.4924283	2.3703704	1.825474	3.0269101
570455.88	2.5891573	2.4587822	2.7246506	2.6428571	2.0752113	3.3178651
577031.63	2.4660695	2.3395886	2.597611	2.4814816	1.9231141	3.1513937
437062.41	2.1118267	1.9777662	2.2525826	2.1304348	1.5761074	2.8165479
537857.38	1.6231068	1.5172101	1.7344462	1.6071428	1.172261	2.1504836
258778.05	2.5233979	2.3335407	2.724587	2.5	1.6178682	3.6904931
288645.69	3.2912323	3.0852444	3.5073569	3.2727273	2.2921779	4.5308342
271519.59	1.9630259	1.7998892	2.1369777	2	1.2533894	3.028024
261438.8	1.4687951	1.3255433	1.6233072	1.4545455	0.83139843	2.3620906
655792.81	1.7566524	1.6566646	1.8610973	1.75	1.2612461	2.3654916
677354.25	1.6653029	1.5695264	1.7653952	1.6666666	1.2156781	2.2301311
589634.56	2.0385509	1.9249216	2.1571362	2.0714285	1.5729226	2.6778018
596603.88	1.9091395	1.799863	2.0233157	1.925926	1.4383736	2.5255957
452361.19	1.7441815	1.6245856	1.8702523	1.7391304	1.2424603	2.368202
555765.63	1.4502516	1.3518459	1.5539269	1.4642857	1.0507967	1.9864689
245667.97	2.6499181	2.4502416	2.8615315	2.5999999	1.6984063	3.8096025
279385.22	4.1018634	3.8677862	4.3464036	4.090909	2.9839373	5.473958
263178.66	2.8915718	2.6897519	3.1045237	2.909091	1.9898161	4.1067686
252538.94	2.0788872	1.9048394	2.2645645	2.090909	1.325457	3.1373904
633227.94	2.3135428	2.1965761	2.4351208	2.2916667	1.7263969	2.9829192
655911.5	2.2670741	2.1532967	2.3853028	2.2592592	1.7281543	2.902113
570736.63	2.3478429	2.2238021	2.4770017	2.3928571	1.8544314	3.0388439
577707.63	2.3316293	2.2087612	2.4595535	2.3333333	1.7929974	2.985347
438493.75	2.2372041	2.0993793	2.3817005	2.2608695	1.6885256	2.9648297
538806.13	1.6332406	1.5271002	1.7448136	1.6071428	1.172261	2.1504836
248708.81	2.2677121	2.0843961	2.4628303	2.3	1.4580027	3.4511292
288763.63	3.3556857	3.1477003	3.5738029	3.3636363	2.3683105	4.6363297
272440.78	2.3087585	2.1318331	2.4964504	2.2727273	1.4707893	3.3549938
260434.75	1.6126881	1.4621247	1.774547	1.6363636	0.96981281	2.5861599
652953	1.9159112	1.8112009	2.0250964	1.9166666	1.4032409	2.5565648
678105.69	1.9303776	1.827208	2.0378551	1.925926	1.4383736	2.5255957
590020	2.1287415	2.0126278	2.2498071	2.1785715	1.6664345	2.7984662
597343.06	2.0574441	1.9440107	2.1757696	2.074074	1.5667332	2.6933577
454024.53	1.9954869	1.8676484	2.1297715	2	1.4642514	2.6677198
557971.5	1.634492	1.5301197	1.7441096	1.6071428	1.172261	2.1504836
243731.86	2.2565782	2.0719044	2.4532974	2.2	1.3787283	3.3308265
288779.91	3.2100573	3.0067132	3.4235346	3.1818182	2.216253	4.425139
272776.69	2.1372795	1.9672871	2.3180301	2.1818182	1.3979321	3.2463725
259948.77	1.7272635	1.571172	1.8946657	1.7272727	1.0399311	2.6973503

651684.06	1.7815381	1.680522	1.8870394	1.75	1.2612461	2.3654916
678508	1.63889	1.543967	1.7381219	1.6296296	1.1840913	2.1877017
590301.69	1.7448705	1.6399244	1.8547713	1.7857143	1.3253915	2.3542416
597703.69	1.8002231	1.6942521	1.9110863	1.8148148	1.34261	2.3992815
454756.56	1.8559381	1.7328233	1.9854912	1.8695652	1.3530136	2.5182922
559110.63	1.5113288	1.4111322	1.616762	1.5	1.081068	2.0275643
230940.95	6.4994969	6.1748128	6.8368225	6.5	5.0165629	8.2847834
279345.03	9.3683424	9.0128155	9.7342987	9.363636	7.6429033	11.356133
264472.06	5.036449	4.769577	5.3143654	5	3.7666843	6.5081873
251283.08	3.0483549	2.8362751	3.2720931	3	2.065062	4.2131152
629306.31	2.5949206	2.4705751	2.7239041	2.5833333	1.9806268	3.3117168
656957	2.2756436	2.1617384	2.3939929	2.2962964	1.7605572	2.9437482
571580	2.3181357	2.1949825	2.4463995	2.3571429	1.8230141	2.9988637
578654.06	2.3468254	2.2236516	2.4750466	2.3703704	1.825474	3.0269101
440834.91	2.4657757	2.3213518	2.6168323	2.4782608	1.8770106	3.2108743
542212.25	1.7115068	1.6031474	1.8252634	1.6785715	1.2333511	2.2321441
233459.86	5.9753313	5.6658463	6.2973256	6	4.5786319	7.7231894
288381.88	7.8819098	7.5611811	8.2127466	7.818182	6.2535362	9.6553907
273920.13	4.8663821	4.6086173	5.1348109	4.818182	3.6091487	6.3023005
259252.19	3.0896556	2.8793676	3.3112407	3.090909	2.1405444	4.3192358
648856.38	2.743288	2.6173129	2.8737595	2.7083333	2.0902345	3.4519932
679177.75	2.5707555	2.4515729	2.6942341	2.5925925	2.0210531	3.2755847
590881.06	2.6265862	2.4975209	2.7605915	2.6785715	2.1068664	3.3576124
598179.81	2.6363311	2.5078051	2.7697365	2.6666667	2.0865026	3.3582253
456380.53	2.3686373	2.2295244	2.5141573	2.3913043	1.8014576	3.1126113
561168.88	1.8354546	1.7250602	1.9510608	1.8214285	1.3561732	2.3948421
221167.97	6.2667303	5.9411144	6.6055512	6.1999998	4.7535043	7.9481201
278900.41	7.6729898	7.3513074	8.005126	7.6363635	6.0910625	9.4543381
265644.97	4.1822739	3.9399331	4.4356203	4.181818	3.0616164	5.5779595
250386.63	2.8955221	2.6885614	3.1141868	2.909091	1.9898161	4.1067686
626679.5	2.7478161	2.6195507	2.8807375	2.7083333	2.0902345	3.4519932
657724	2.6074767	2.485517	2.7338729	2.6296296	2.0537627	3.31692
571774.13	2.8979976	2.7601259	3.0409732	2.9285715	2.3291798	3.6351292
579175	2.7297449	2.5968313	2.8676982	2.7407408	2.152071	3.4407489
442382.09	2.5023618	2.357105	2.654227	2.5217392	1.9148623	3.2599325
543348.19	2.0502508	1.9316071	2.1742749	2.0357144	1.5418301	2.6375039
223608.59	5.3352156	5.036726	5.6467748	5.3000002	3.9700637	6.9325304
287990.72	6.0418615	5.7612782	6.3325758	6	4.6403995	7.633471
275051.16	3.5775163	3.3574514	3.808217	3.5454545	2.5211647	4.8467531
258339.67	2.4038119	2.2184432	2.6005356	2.3636363	1.5440058	3.463275
646302.56	2.277571	2.1626935	2.3969662	2.25	1.6902692	2.9357619
679920.75	2.3238003	2.2106171	2.4412763	2.3333333	1.7929974	2.985347
590617.25	2.3585494	2.2363048	2.485739	2.3928571	1.8544314	3.0388439
598965.63	2.3791015	2.2571657	2.5059125	2.4074075	1.8579862	3.0684383
457867.16	2.2866895	2.1502593	2.4295068	2.3043478	1.7261146	3.0141437
561675.94	1.7447783	1.6372359	1.8575293	1.7142857	1.263979	2.2728941
218740.22	4.3841958	4.111073	4.6706948	4.4000001	3.1970468	5.9067945
287811.41	4.405663	4.1664767	4.6550002	4.3636365	3.217401	5.7855487
275526.63	2.7365777	2.5447071	2.9390817	2.7272727	1.8400794	3.8933513
257840.08	2.1796455	2.0031421	2.3675337	2.1818182	1.3979321	3.2463725
644806.69	2.8613226	2.7322357	2.9949336	2.8333333	2.2001941	3.5919251
680238.63	2.6755316	2.5540099	2.801342	2.7037036	2.119272	3.3995016
590483.13	2.6842427	2.5537076	2.819721	2.7142856	2.1385484	3.3973334
599317.5	2.7397833	2.6088517	2.8755841	2.7777777	2.1848984	3.4819684
458622.22	2.6099913	2.4642107	2.7621441	2.6086957	1.9907097	3.3579085
561415.38	1.9094596	1.796852	2.0272758	1.8928572	1.4178799	2.4759037
193392.5	3.3610404	3.1075885	3.6296558	3.3	2.2715681	4.6344271
260032.61	3.8648999	3.6296151	4.1114335	3.8181818	2.7518096	5.1610727
249206.72	2.3594871	2.1726041	2.558145	2.3636363	1.5440058	3.463275
232787.52	2.2767544	2.0870223	2.4791014	2.2727273	1.4707893	3.3549938
581105	3.0063415	2.8670039	3.1506996	3	2.3473153	3.7780035
614596.5	2.7481446	2.6186316	2.8824055	2.7777777	2.1848984	3.4819684
533108.88	3.0425305	2.8962483	3.1942873	3.0714285	2.4567463	3.7931893

541545.31	2.8935714	2.7520607	3.040472	2.925926	2.316483	3.6465766
414733.22	2.6836529	2.5282869	2.8460679	2.6956522	2.066741	3.4557045
506607.22	1.8989071	1.7807921	2.0227971	1.8928572	1.4178799	2.4759037
214040.58	5.2279806	4.9261155	5.5435042	5.1999998	3.8836088	6.819108
283041.13	5.6988187	5.4240704	5.9838781	5.7272725	4.4009938	7.3276696
276235.16	3.5440819	3.3255265	3.7732277	3.5454545	2.5211647	4.8467531
257640.03	2.7984781	2.5979099	3.0104213	2.8181818	1.9148178	4.0001841
641816.75	3.7331529	3.5851548	3.8856914	3.7083333	2.9780962	4.5634232
680546.63	3.2253485	3.0918159	3.3631647	3.2222223	2.580868	3.9745991
589886	3.1819708	3.039633	3.3292534	3.2142856	2.5846653	3.9509029
599868.38	3.2007022	3.0591207	3.3471458	3.2222223	2.580868	3.9745991
460099.47	2.8037415	2.6528106	2.9610224	2.826087	2.1811142	3.6020799
561926.56	2.2672002	2.1443989	2.395201	2.25	1.7289618	2.8787274
202423.7	5.1130376	4.8062434	5.4342794	5.0999999	3.7972851	6.7055583
273983.53	4.8251076	4.5684824	5.0923948	4.818182	3.6091487	6.3023005
267675.25	3.3286605	3.113656	3.5545995	3.3636363	2.3683105	4.6363297
249206.89	2.6925418	2.4926414	2.9042075	2.7272727	1.8400794	3.8933513
619599.44	3.1939991	3.0548124	3.3378923	3.1666667	2.4949732	3.9635556
658693.56	2.838953	2.7117243	2.9706097	2.8518519	2.2506368	3.5643256
570757.38	2.7664995	2.631712	2.9064009	2.7857144	2.2019916	3.4766974
580711.06	2.3970613	2.2727764	2.5263753	2.4074075	1.8579862	3.0684383
446076.97	2.3650627	2.2244844	2.5121968	2.347826	1.7637593	3.0634038
544984.88	1.7229836	1.6145279	1.8368084	1.7142857	1.263979	2.2728941
204339.34	7.1596589	6.7974405	7.5361662	7.0999999	5.5451589	8.9556837
282870.13	5.7906432	5.5135808	6.078023	5.818182	4.4807091	7.4296885
276899.19	3.5175257	3.3000607	3.7455561	3.5454545	2.5211647	4.8467531
257374.53	2.1952443	2.0179403	2.3839531	2.1818182	1.3979321	3.2463725
638660.19	2.8262291	2.6973367	2.9596894	2.7916667	2.1635034	3.5453179
680794.56	2.2840958	2.1719668	2.4005125	2.2962964	1.7605572	2.9437482
589707.56	2.2604425	2.1407101	2.385128	2.2857144	1.7602785	2.9188061
600436.88	2.1750829	2.0587034	2.2963278	2.1851852	1.6634642	2.8187296
461763.41	2.0984771	1.9684136	2.2348766	2.0869565	1.538757	2.7670016
564510.19	2.0903077	1.9727281	2.2130647	2.0714285	1.5729226	2.6778018
192835.61	8.8520994	8.4371023	9.2822285	8.8000002	7.0578575	10.841846
273709.38	6.8649459	6.5580206	7.1825275	6.818182	5.3629327	8.5466499
268292	4.8529215	4.5928688	5.1238627	4.818182	3.6091487	6.3023005
249111.11	2.9906335	2.7797105	3.2133181	3	2.065062	4.2131152
616511.44	3.2489259	3.1081872	3.3943946	3.25	2.5689902	4.0561471
659052.44	2.7888525	2.6627984	2.9193327	2.7777777	2.1848984	3.4819684
570773.63	2.6385241	2.5069323	2.7752311	2.6785715	2.1068664	3.3576124
581295.31	2.5219538	2.3944933	2.6544375	2.5555556	1.9883747	3.2342191
447641	2.3299921	2.1907163	2.4758012	2.347826	1.7637593	3.0634038
547496.38	2.1296945	2.0091927	2.2555349	2.1071429	1.6040548	2.7180607
194230.61	8.3457499	7.9443707	8.7621546	8.3000002	6.6109037	10.289099
282540.75	6.0062132	5.7238159	6.2989383	6	4.6403995	7.633471
277654.94	3.9761584	3.7450426	4.217804	4	2.9064062	5.3698134
257335.3	2.401536	2.2159028	2.5985687	2.3636363	1.5440058	3.463275
635356.19	2.4852202	2.3641369	2.6108973	2.4583333	1.8713973	3.1710708
681277.69	2.2149558	2.1045971	2.3295999	2.2222223	1.6957897	2.8604405
590005.19	2.1813366	2.063776	2.303849	2.2142856	1.6976801	2.8386145
600963.88	2.2197673	2.1022332	2.3421621	2.2592592	1.7281543	2.902113
463424.97	2.2117927	2.0784442	2.3514524	2.2173913	1.6509935	2.9154601
566864.31	1.8417106	1.7316737	1.9569066	1.8214285	1.3561732	2.3948421
189009.98	6.2271843	5.8764663	6.5933657	6.1999998	4.7535043	7.9481201
282572.63	4.3776355	4.1370535	4.6285586	4.3636365	3.217401	5.7855487
277941.44	2.8495212	2.6544976	3.0550826	2.8181818	1.9148178	4.0001841
257375.25	2.0087402	1.8392974	2.1895947	2	1.2533894	3.028024
633812.56	2.1410115	2.0285993	2.2580321	2.125	1.5822021	2.7939825
681571.25	1.7767768	1.6781025	1.8797388	1.7777778	1.310793	2.3570755
590369.19	1.8717102	1.7629647	1.9854078	1.8928572	1.4178799	2.4759037
600963.5	2.0450494	1.9322993	2.1626618	2.074074	1.5667332	2.6933577
464356.88	2.011384	1.8844416	2.144628	2	1.4642514	2.6677198
567694.13	1.8390185	1.7291425	1.954046	1.8214285	1.3561732	2.3948421

177992.45	11.258904	10.771305	11.762887	11.2	9.2220449	13.47651
273383.41	9.0751667	8.7215443	9.4394484	9.090909	7.3967266	11.056981
269194.91	4.6174722	4.3643141	4.881485	4.6363635	3.4520772	6.095962
249125.63	3.4039052	3.1786292	3.6409335	3.3636363	2.3683105	4.6363297
611960.19	2.699522	2.5709009	2.832912	2.6666667	2.0536582	3.4052739
659856.63	2.2368495	2.124177	2.3539469	2.2592592	1.7281543	2.902113
571592.44	2.2411072	2.1200469	2.3672791	2.2857144	1.7602785	2.9188061
581723.88	2.3172505	2.1951849	2.4443374	2.3333333	1.7929974	2.985347
450272.34	2.4340825	2.2920926	2.5825655	2.4347825	1.8392086	3.1617677
550128.75	1.9940786	1.8778083	2.1156638	1.9642857	1.4797689	2.556788
178679.17	11.792085	11.293899	12.306589	11.7	9.676219	14.022143
282403.84	10.014028	9.6483164	10.390054	10	8.2187834	12.052704
278384.59	4.9032884	4.6465902	5.1704788	4.909091	3.6878603	6.4052987
257364.56	3.3571055	3.1369586	3.5886273	3.3636363	2.3683105	4.6363297
631016	2.755873	2.6278543	2.8885157	2.75	2.1268499	3.4986742
682122.25	2.3163004	2.2034824	2.4333973	2.3333333	1.7929974	2.985347
590800.31	2.383208	2.2603362	2.5110228	2.4285715	1.8858807	3.078793
601282.13	2.301748	2.1820652	2.4262881	2.3333333	1.7929974	2.985347
466290.72	2.1981995	2.0656705	2.3370008	2.2173913	1.6509935	2.9154601
568977	1.9772328	1.8633671	2.0962365	1.9642857	1.4797689	2.556788
167937.64	14.189791	13.625731	14.771205	14.1	11.868785	16.628794
273179.91	11.915225	11.509372	12.331736	11.909091	9.9571657	14.131815
269775.5	5.5675921	5.2895532	5.8564534	5.5454545	4.2418332	7.1233683
249066.75	3.4689496	3.2414684	3.7081847	3.4545455	2.4446421	4.7416334
609372.19	3.1721172	3.0322676	3.3167531	3.125	2.4580107	3.9172146
660317.19	2.7835107	2.6576982	2.9137411	2.7777777	2.1848984	3.4819684
571918.19	2.7241659	2.5905607	2.862875	2.75	2.1702571	3.4370282
581998.94	2.6134069	2.4837036	2.748126	2.6296296	2.0537627	3.31692
452066.38	2.7341118	2.5837929	2.8908944	2.7391305	2.1048231	3.5045378
550813.63	2.1441009	2.0235457	2.2699621	2.1071429	1.6040548	2.7180607
168647.91	10.773926	10.284179	11.280971	10.7	8.7689085	12.929854
281852.97	9.7284765	9.3677158	10.099572	9.727273	7.9717345	11.754414
279210.41	4.8708787	4.6154151	5.1368032	4.909091	3.6878603	6.4052987
257379.75	2.7896523	2.5893071	3.001384	2.8181818	1.9148178	4.0001841
628211.63	2.5214434	2.398787	2.648746	2.5	1.9077634	3.2179956
682495.31	2.3531296	2.2394381	2.4710979	2.3703704	1.825474	3.0269101
591200.94	2.3020937	2.1813993	2.4277287	2.3214285	1.7916296	2.9588513
601262.19	2.2635715	2.1448967	2.3871043	2.2962964	1.7605572	2.9437482
468016.31	2.2221448	2.0891268	2.3614113	2.2173913	1.6509935	2.9154601
569547.06	1.8154777	1.706493	1.9295975	1.7857143	1.3253915	2.3542416
163770.64	10.227718	9.7437267	10.729529	10.2	8.3168812	12.382104
281428	8.9827595	8.6359749	9.3398972	9	7.3147559	10.957177
279591.13	4.9715457	4.7135935	5.239943	5	3.7666843	6.5081873
257321.48	2.6115191	2.4177756	2.8166568	2.6363637	1.7656142	3.786258
626523.94	2.6447513	2.5189278	2.7752328	2.625	2.017122	3.3585153
682663.63	2.3203228	2.2074502	2.4374714	2.3333333	1.7929974	2.985347
591418.06	2.4280624	2.3040886	2.5569737	2.4642856	1.9173614	3.1187112
601232.25	2.4516315	2.3280575	2.5800619	2.4814816	1.9231141	3.1513937
469022.09	2.4838915	2.3432894	2.6307256	2.4782608	1.8770106	3.2108743
570151.25	1.9854381	1.8714486	2.1045547	1.9642857	1.4797689	2.556788
143516.77	11.190331	10.649669	11.751328	11.2	9.2220449	13.47651
254177.64	9.0763292	8.7097006	9.4544249	9.090909	7.3967266	11.056981
252920.08	5.0450721	4.7720203	5.3296752	5	3.7666843	6.5081873
232403.14	2.994796	2.7764089	3.2257957	3	2.065062	4.2131152
564410.69	2.6239758	2.4920237	2.761101	2.5833333	1.9806268	3.3117168
616861.38	2.2533426	2.1364264	2.3749931	2.2592592	1.7281543	2.902113
534162.63	2.5160878	2.3833535	2.6542902	2.5357144	1.9804139	3.1984584
543048	2.2999809	2.1741807	2.4311616	2.3333333	1.7929974	2.985347
424374.63	2.6368213	2.4845703	2.7959609	2.652174	2.028703	3.4068284
515516.31	2.1163249	1.9925928	2.2457285	2.1071429	1.6040548	2.7180607
154462.48	12.261877	11.715811	12.826827	12.2	10.131363	14.566817
285490.09	9.1106491	8.7638502	9.4676533	9.090909	7.3967266	11.056981
275524.78	5.1283956	4.8644509	5.4029384	5.090909	3.845618	6.6109691

257359.39	3.1046078	2.8930426	3.3275535	3.090909	2.1405444	4.3192358
628995.75	2.7392871	2.6114564	2.8717568	2.7083333	2.0902345	3.4519932
677204.13	2.3715153	2.2569354	2.4904053	2.3703704	1.825474	3.0269101
596807	2.4698102	2.3453197	2.5991929	2.5	1.9488727	3.1585996
595744.75	2.5648568	2.4378514	2.6967623	2.5925925	2.0210531	3.2755847
474611.06	2.6927311	2.5471065	2.8445117	2.6956522	2.066741	3.4557045
568217.13	2.0837104	1.9666973	2.2058673	2.0714285	1.5729226	2.6778018
144754.09	9.3676109	8.8755922	9.8798075	9.3999996	7.5961571	11.50322
275996.72	7.1413894	6.8295612	7.4637837	7.090909	5.6050692	8.8497753
267160.84	4.3007798	4.0556669	4.5568342	4.2727275	3.1394393	5.6818213
249055.52	2.4492531	2.2587192	2.6515665	2.4545455	1.6175609	3.5712347
606983.81	2.1845722	2.068558	2.3053992	2.1666667	1.6181703	2.841295
655592.38	2.0256488	1.9181544	2.1375992	2.0370371	1.5345751	2.6514838
577436.5	2.0175378	1.9033339	2.1368036	2.0357144	1.5418301	2.6375039
576719.88	2.3616319	2.2378607	2.4904673	2.3703704	1.825474	3.0269101
460055.84	2.5018702	2.3594041	2.6506901	2.5217392	1.9148623	3.2599325
550755.56	1.8846836	1.7717593	2.002918	1.8571428	1.3870031	2.4353957
144882.39	12.058056	11.499191	12.637059	12	9.949193	14.349058
284942.56	9.3352146	8.9837933	9.6968603	9.272727	7.560801	11.256458
276007.38	5.3440599	5.0747838	5.6239147	5.3636365	4.0830483	6.9187002
257504.92	2.5164568	2.326407	2.717895	2.5454545	1.6914361	3.6788905
625826.75	2.4543533	2.3331327	2.5802386	2.4166667	1.8350763	3.1241021
677242.56	2.3034582	2.190558	2.4206688	2.3333333	1.7929974	2.985347
597236.06	2.3223648	2.2017388	2.4478807	2.3571429	1.8230141	2.9988637
595504.69	2.4382679	2.3144519	2.5669873	2.4444444	1.8905332	3.1099327
476549.22	2.7447321	2.5979841	2.8976107	2.7391305	2.1048231	3.5045378
570034	2.1314518	2.0132728	2.2547574	2.1071429	1.6040548	2.7180607
135375.91	10.334188	9.799696	10.890251	10.3	8.4071941	12.491746
275429.69	9.0876188	8.735055	9.4507618	9.090909	7.3967266	11.056981
267595.47	5.6054759	5.3253608	5.8965015	5.6363635	4.3213677	7.225564
249327.42	2.7554128	2.5531952	2.969388	2.7272727	1.8400794	3.8933513
603903.63	2.9640491	2.8283126	3.1046164	2.9583333	2.310483	3.731535
655720.94	2.6062915	2.4841762	2.7328572	2.6296296	2.0537627	3.31692
577892.19	2.6077528	2.4777381	2.7428193	2.6428571	2.0752113	3.3178651
576489.75	2.5204265	2.3924818	2.6534359	2.5555556	1.9883747	3.2342191
461977.94	2.7642012	2.6146538	2.920073	2.7826087	2.1429477	3.5533292
552757.31	2.1166613	1.9970989	2.2415113	2.1071429	1.6040548	2.7180607
135060.73	9.1440344	8.6411037	9.6686001	9.1000004	7.3267522	11.172784
284337.69	7.8990583	7.5757251	8.2326431	7.909091	6.3348575	9.7558346
276466.94	4.6696362	4.4183564	4.9314833	4.6363635	3.4520772	6.095962
257834.58	2.5559022	2.36446	2.7587175	2.5454545	1.6914361	3.6788905
622554.44	2.2744999	2.1575601	2.3961303	2.25	1.6902692	2.9357619
677584.88	1.9982736	1.893241	2.1076169	2	1.5024616	2.6095662
597597.56	2.0398343	1.926918	2.1576407	2.0714285	1.5729226	2.6778018
595574.94	2.2650383	2.1457665	2.3892143	2.2962964	1.7605572	2.9437482
478375.13	2.4708643	2.3319943	2.6158438	2.4782608	1.8770106	3.2108743
572033.75	2.0505784	1.9348948	2.1713712	2.0357144	1.5418301	2.6375039
130036.02	8.0439253	7.563777	8.5465631	8	6.3435025	9.9566927
284174.53	6.0279856	5.7458754	6.320364	6	4.6403995	7.633471
276582.34	3.5324018	3.3143475	3.7610338	3.5454545	2.5211647	4.8467531
258109.33	2.4137058	2.2278671	2.6109092	2.4545455	1.6175609	3.5712347
620832	2.2775888	2.1604085	2.3994727	2.25	1.6902692	2.9357619
677893.94	1.9103284	1.8076875	2.0172787	1.925926	1.4383736	2.5255957
597959.69	2.0235479	1.9111232	2.1408596	2.0357144	1.5418301	2.6375039
595536.94	2.3071616	2.1867673	2.4324601	2.3333333	1.7929974	2.985347
479302.09	2.5307629	2.3903298	2.6772931	2.5217392	1.9148623	3.2599325
572863.94	2.1855102	2.0661125	2.3100085	2.1785715	1.6664345	2.7984662
120997.45	11.124202	10.537784	11.73476	11.1	9.1313324	13.367263
274788.22	9.2689562	8.912447	9.6360693	9.272727	7.560801	11.256458
267846.75	4.7825856	4.5242391	5.0518398	4.818182	3.6091487	6.3023005
250000.06	2.9319992	2.723557	3.1521628	2.909091	1.9898161	4.1067686
599308.44	2.5412624	2.4152217	2.6721745	2.5416667	1.9441736	3.2648771
656211.25	2.1471744	2.0365107	2.2622883	2.1481481	1.631179	2.7769797

579128.56	2.0548115	1.9397082	2.1749613	2.0714285	1.5729226	2.6778018
576298.88	2.255774	2.1348026	2.3818145	2.2592592	1.7281543	2.902113
464737.66	2.450845	2.3105621	2.5974181	2.4782608	1.8770106	3.2108743
555268.31	2.1052885	1.9863185	2.2295225	2.1071429	1.6040548	2.7180607
119991.55	12.017513	11.405175	12.654186	12	9.949193	14.349058
283592.63	10.194201	9.8259497	10.572721	10.181818	8.3836775	12.251373
277000.69	5.3934884	5.1234331	5.6740837	5.3636365	4.0830483	6.9187002
258445.36	3.2888963	3.0714836	3.5176375	3.2727273	2.2921779	4.5308342
617913.06	2.9017026	2.7689312	3.0391955	2.875	2.2369215	3.6384964
678173.13	2.4403799	2.3242099	2.560853	2.4444444	1.8905332	3.1099327
598910.75	2.3642921	2.2427359	2.4907243	2.3928571	1.8544314	3.0388439
595395.5	2.5092564	2.3836164	2.6398001	2.5185184	1.9557282	3.1928225
481116.88	2.6126707	2.4702168	2.7611978	2.6086957	1.9907097	3.3579085
574439.56	2.1499217	2.0316739	2.2732561	2.1428571	1.6352258	2.7582819
111245.95	14.013993	13.326901	14.727324	14	11.777057	16.52055
274085.16	12.113024	11.704457	12.532211	12.090909	10.123466	14.329094
268535.59	6.3939381	6.095046	6.7036963	6.3636365	4.9607668	8.0400715
250315.89	3.3397801	3.1171944	3.5740631	3.3636363	2.3683105	4.6363297
596363.88	3.2362792	3.0934911	3.3839579	3.2083333	2.5319664	4.0098662
656686.06	2.9329083	2.8033724	3.0668855	2.925926	2.316483	3.6465766
579711.38	2.8669438	2.7307525	3.0081694	2.8928571	2.2973461	3.5955572
576280.06	3.0211005	2.8808408	3.1664231	3.0370371	2.4154456	3.7697637
466245.34	3.3029821	3.1400571	3.4721682	3.3043478	2.6034503	4.1358838
556466.56	2.6201036	2.4873242	2.7581298	2.6071429	2.0435839	3.2780907
110115.7	10.834059	10.227926	11.466733	10.8	8.8594494	13.039271
282917.25	10.045341	9.6793814	10.421595	10	8.2187834	12.052704
277587.84	5.4289122	5.1582432	5.7100983	5.4545455	4.1623926	7.0210814
258834.77	2.95169	2.7460716	3.1686268	2.909091	1.9898161	4.1067686
615017.75	2.6487041	2.5216272	2.7805262	2.625	2.017122	3.3585153
678437.88	2.6546278	2.5334284	2.780127	2.6666667	2.0865026	3.3582253
599539.56	2.6954017	2.5655718	2.8300998	2.7142856	2.1385484	3.3973334
595042.5	2.6872029	2.5570903	2.8222208	2.7037036	2.119272	3.3995016
482895.28	2.9281712	2.777519	3.0848706	2.9565217	2.2958548	3.7480958
575031.44	2.2224872	2.1022935	2.3477616	2.2142856	1.6976801	2.8386145
105433.46	11.96015	11.309052	12.63896	11.9	9.8581629	14.240124
282519.66	11.351422	10.961916	11.751235	11.363636	9.4589911	13.53926
277516.66	5.7257824	5.4476833	6.0143991	5.7272725	4.4009938	7.3276696
259083.81	3.4274623	3.2057087	3.6605134	3.4545455	2.4446421	4.7416334
613835.38	3.1588273	3.0197768	3.3026292	3.125	2.4580107	3.9172146
678018.88	2.9792681	2.8507488	3.1120882	3	2.3824329	3.7287261
600267.44	2.9919996	2.8552096	3.1336498	3.0357144	2.4248207	3.7537076
594283.38	3.1634741	3.0220752	3.3097808	3.1851852	2.5477371	3.9336777
484257.44	3.2854426	3.1259689	3.4509437	3.3043478	2.6034503	4.1358838
574603.81	2.6661849	2.5343323	2.803118	2.6428571	2.0752113	3.3178651
90873.039	12.655019	11.934085	13.408119	12.6	10.496137	15.001905
254912.67	11.721662	11.305101	12.149646	11.727273	9.7909842	13.934418
250976.38	6.1479888	5.8450203	6.4625888	6.181818	4.8004236	7.8369274
234342.28	3.4436808	3.2101545	3.6897039	3.4545455	2.4446421	4.7416334
552552.94	3.1435902	2.9974778	3.2949831	3.125	2.4580107	3.9172146
612974.56	2.9462886	2.8119576	3.0853789	2.9629629	2.3494453	3.6876638
541859.06	3.0709093	2.9251151	3.2220888	3.1071429	2.488694	3.8326492
536951.19	3.1995459	3.0500224	3.3545036	3.2222223	2.580868	3.9745991
437951.44	3.5003881	3.3273368	3.680105	3.5217392	2.7967691	4.3772001
519598.84	2.6058564	2.4688883	2.748446	2.6071429	2.0435839	3.2780907
96424.891	12.735301	12.032882	13.468025	12.7	10.587418	15.110591
281815.72	11.354938	10.964889	11.75532	11.363636	9.4589911	13.53926
281961.13	5.9263487	5.6455731	6.217474	5.909091	4.5605116	7.5316215
255140.36	3.1982396	2.9825361	3.425421	3.1818182	2.216253	4.425139
610848.38	2.9974706	2.8617313	3.1379852	2.9583333	2.310483	3.731535
677875.75	2.9400668	2.8123918	3.0720437	2.9629629	2.3494453	3.6876638
600842.31	2.9408715	2.8053329	3.0812657	2.9642856	2.361037	3.6746781
593302.5	3.2024136	3.0600212	3.3497221	3.2222223	2.580868	3.9745991
486586.25	3.4135778	3.2513707	3.5817823	3.4347825	2.7193496	4.2807641

575761.38	2.7910869	2.6562767	2.9309661	2.75	2.1702571	3.4370282
88847.023	9.2406025	8.6192427	9.8949213	9.1999998	7.4164987	11.282983
272388.47	8.8990555	8.5482826	9.2605267	8.909091	7.2328305	10.857328
272828.53	4.79422	4.5378952	5.0612526	4.818182	3.6091487	6.3023005
247204.91	2.5323122	2.3378	2.7386901	2.5454545	1.6914361	3.6788905
589626.63	2.3591201	2.2367589	2.4864342	2.3333333	1.7625749	3.0300274
656033.38	2.3825006	2.265837	2.5036139	2.3703704	1.825474	3.0269101
581455.5	2.2598462	2.1392951	2.3854215	2.2857144	1.7602785	2.9188061
574140	2.5098407	2.381911	2.6428564	2.5185184	1.9557282	3.1928225
471688.59	2.6839743	2.5381463	2.8359962	2.6956522	2.066741	3.4557045
558170	2.1283839	2.0090604	2.2529433	2.1071429	1.6040548	2.7180607
87308.133	10.68629	10.011501	11.394594	10.7	8.7689085	12.929854
281273.19	9.8942957	9.5300732	10.268873	9.909091	8.1363945	11.953313
281713.5	5.1115761	4.8509436	5.3825741	5.090909	3.845618	6.6109691
255486.69	2.5676484	2.3748956	2.771879	2.5454545	1.6914361	3.6788905
608185.63	2.5419872	2.4168398	2.6719341	2.5416667	1.9441736	3.2648771
677376.06	2.4934452	2.3759353	2.6152625	2.4814816	1.9231141	3.1513937
601618.5	2.453382	2.3298025	2.5818148	2.5	1.9488727	3.1585996
592649.88	2.7031138	2.5723517	2.8388007	2.7407408	2.152071	3.4407489
488601.31	3.0004013	2.8487597	3.1580186	3	2.3341792	3.7966919
577834.56	2.249779	2.1291292	2.3754845	2.25	1.7289618	2.8787274
77176.43	10.819366	10.097865	11.578806	10.8	8.8594494	13.039271
262974.06	11.164599	10.764369	11.575904	11.181818	9.2931843	13.341493
263408.38	7.7256465	7.3935957	8.0687675	7.7272725	6.1722708	9.5548925
239339.77	3.9525399	3.704649	4.2126565	4	2.9064062	5.3698134
567528.06	4.2605824	4.0924373	4.4338622	4.25	3.4653671	5.1592102
633615.94	3.8446002	3.693428	4.0003715	3.8518519	3.1472421	4.6671634
563048.44	3.6870718	3.5301559	3.8491662	3.7142856	3.0348406	4.5004787
554384.81	3.1909244	3.0439436	3.3431678	3.2222223	2.580868	3.9745991
457808.22	3.6412628	3.4685445	3.8203554	3.652174	2.9131169	4.5216398
541855.81	2.6261599	2.4914682	2.7662406	2.6071429	2.0435839	3.2780907
375018.75	4.1811242	3.9767101	4.393322	4.0599999	3.7855136	4.3491292
480632.28	4.8519421	4.6569958	5.0529523	4.6750002	4.3801069	4.9845243
828946.69	4.5105433	4.3671122	4.6574855	4.5349998	4.2446284	4.8400054
202409.09	4.7428699	4.4475541	5.0526404	4.6750002	4.3801069	4.9845243
569960.5	5.6617961	5.4681177	5.8605824	5.4699998	5.1506257	5.8039932
719981.88	4.7084517	4.5512724	4.8696747	4.54	4.2494655	4.8451676
422404.66	5.1561933	4.9418988	5.377389	5.0549998	4.7481651	5.3764596
464151.34	4.8238578	4.6260967	5.0278988	4.6149998	4.3220363	4.922596
425180.38	4.8896894	4.6817408	5.104496	4.7649999	4.467236	5.0773935
387090.28	3.9758167	3.77964	4.1795349	3.8900001	3.6214232	4.173223
496110.97	4.5776048	4.3912525	4.7698326	4.4000001	4.1140547	4.7005811
855963.69	4.0714345	3.9373701	4.2088995	4.1500001	3.8724351	4.4422054
208900.05	4.0497837	3.7814512	4.3321319	3.9549999	3.6841488	4.2404962
588222.56	5.0474091	4.8674684	5.2323012	4.9299998	4.6270423	5.2475848
743184.38	4.3676915	4.2187161	4.5205846	4.2550001	3.9738855	4.5507531
435945.94	4.8882208	4.6828594	5.1002698	4.8000002	4.5011268	5.113502
479099.72	4.4604492	4.2733197	4.6536641	4.3150001	4.0318766	4.612761
438851.72	4.4639225	4.2684164	4.6660738	4.3449998	4.0608773	4.6437593
386670.13	4.5490971	4.3389683	4.7667713	4.4699998	4.1817513	4.7728834
495566.38	5.1234307	4.9260626	5.3266788	4.9749999	4.6706409	5.2939854
855385.88	4.7580867	4.6130185	4.9065561	4.8800001	4.5786066	5.1960211
208645.52	4.9030528	4.6071649	5.2129593	4.8299999	4.5301795	5.1444492
587491.88	5.6562486	5.4655514	5.8519011	5.5300002	5.2088528	5.8657656
742388.19	5.0526128	4.8921995	5.2169461	4.9499998	4.6464186	5.268208
435412.47	5.5694318	5.349947	5.7956095	5.4899998	5.1700339	5.824585
478565.97	5.131999	4.9310207	5.339066	5.0250001	4.7190914	5.3455343
438353.47	5.2355008	5.0234718	5.4541793	5.1300001	4.8208618	5.4537621
361337.84	4.1650772	3.9572828	4.3809509	4.0749998	3.7999983	4.3646441
463096.03	4.6469841	4.4527025	4.8475618	4.5149999	4.2252798	4.8193541
799621.44	4.2970333	4.1545434	4.4431639	4.3850002	4.0995507	4.6850853
194949.73	4.2985439	4.0123959	4.5997109	4.25	3.9690535	4.5455852
548950.19	4.8146443	4.632822	5.0017743	4.6999998	4.4043069	5.010324

693736.63	4.6905408	4.5307488	4.8545299	4.5949998	4.3026819	4.9019504
406828.5	4.8767476	4.6644964	5.0961676	4.8099999	4.5108109	5.1238179
447218.06	4.20153	4.0136833	4.3958983	4.085	3.8096552	4.3749871
409611.56	4.3675523	4.1674886	4.574739	4.2750001	3.9932144	4.571424
385857.19	4.1725283	3.9711797	4.3814406	4.1199999	3.8434575	4.4111838
494511.84	5.0635796	4.8671718	5.2658796	4.9349999	4.631886	5.2527404
854129.38	4.8786521	4.7316365	5.0290742	4.7649999	4.467236	5.0773935
208152.16	5.0347786	4.7345309	5.3490758	4.9749999	4.6706409	5.2939854
586140.63	5.0278039	4.8478999	5.212676	4.9299998	4.6270423	5.2475848
740775.75	5.0960093	4.9347291	5.2612176	4.9899998	4.6851749	5.3094506
434369.28	5.3525887	5.1372066	5.5746803	5.355	5.0390515	5.6855693
477563.94	4.2549276	4.0719161	4.4440451	4.165	3.8869252	4.457715
437391.91	4.8994961	4.694232	5.1114259	4.8000002	4.5011268	5.113502
372959.31	2.2066751	2.0584702	2.3627312	2.1949999	1.994447	2.4102569
477946.75	2.5400319	2.3991418	2.6870365	2.5150001	2.2999864	2.7447021
825669.38	2.4464998	2.3409631	2.5555685	2.4400001	2.2282889	2.6664031
201145.63	2.2769573	2.0731745	2.4953554	2.2850001	2.0802791	2.5044203
566415	2.4028318	2.2768559	2.5339644	2.365	2.1566427	2.5880527
715963.69	2.3800089	2.2683342	2.4957595	2.355	2.1470938	2.5776021
419814.41	2.6130595	2.4606977	2.7723856	2.6199999	2.4004445	2.8542397
461537.66	2.0670035	1.9379035	2.2024431	2.0350001	1.8420736	2.2426391
422734.5	2.7866189	2.629741	2.9504111	2.7449999	2.5201547	2.9845247
384955.94	1.7040912	1.5761653	1.8396343	1.7	1.5240816	1.8906535
493314.38	2.1122434	1.9859238	2.2444913	2.085	1.889659	2.2950506
852575.94	1.8426511	1.7526493	1.9360766	1.875	1.6900065	2.0747161
207561.92	1.8211433	1.6421593	2.0143125	1.8099999	1.6283257	2.0064013
584530	2.2188766	2.0997484	2.3430028	2.2149999	2.0135136	2.4311893
738997.38	1.7456083	1.6516387	1.8435313	1.73	1.5524938	1.9222391
433311.28	2.0954912	1.9613916	2.2363451	2.095	1.8991796	2.3055296
476374.69	1.5596967	1.4495493	1.6759946	1.545	1.3775184	1.7272295
436273.09	1.950613	1.8217422	2.0861948	1.9450001	1.7564958	2.1482222
372206.44	2.4260731	2.270396	2.5896134	2.4000001	2.1900709	2.6246226
476994.16	2.5430081	2.4018955	2.6902473	2.52	2.3047678	2.7499204
824676.75	2.3002954	2.1979349	2.4061925	2.3050001	2.099364	2.5253341
200669.89	2.4268713	2.2160881	2.6522975	2.405	2.1948473	2.6298461
565154	2.4170403	2.2905483	2.5487003	2.3800001	2.1709676	2.6037271
714573.94	2.2488925	2.1402705	2.361599	2.2249999	2.0230484	2.441654
418939.66	2.6018066	2.4496219	2.7609706	2.6099999	2.3908732	2.8438115
460624.91	2.1926734	2.059515	2.3321819	2.1500001	1.951563	2.3631432
421814.53	2.3944173	2.249007	2.5467618	2.3699999	2.1614172	2.5932777
384297.63	2.1051393	1.9625571	2.255342	2.0899999	1.8944192	2.3002903
492510.09	2.1420882	2.0147634	2.2753506	2.125	1.9277482	2.3369594
851776.06	1.9030824	1.811556	1.9980352	1.9450001	1.7564958	2.1482222
207172.81	2.2300224	2.0312858	2.4429481	2.24	2.0373526	2.4573488
583509.88	2.1662016	2.048414	2.2889965	2.1500001	1.951563	2.3631432
737835.13	1.9692746	1.8693082	2.0731986	1.96	1.7707517	2.1639655
432524.34	2.1154878	1.9806188	2.257123	2.1300001	1.9325106	2.3421967
475614	1.8691628	1.7482966	1.9961832	1.855	1.6710216	2.0537024
435525.78	2.2432656	2.1047893	2.3884592	2.2349999	2.0325842	2.4521174
383983.84	1.8464319	1.7130029	1.9874936	1.83	1.6472982	2.0274274
492110.06	2.0645788	1.9395643	2.1955369	2.0550001	1.8611042	2.2636073
851379.25	1.6561362	1.5708101	1.7448925	1.675	1.5004156	1.8643216
206992.31	1.7971681	1.619159	1.9894036	1.795	1.6141001	1.990628
583025.31	1.9484575	1.8367854	2.0651433	1.9299999	1.7422427	2.1324763
737251.19	1.8148496	1.7188971	1.914764	1.79	1.6093589	1.9853696
432142.28	2.061821	1.9286441	2.201771	2.04	1.8468308	2.2478814
475246.78	1.7401485	1.6235547	1.8629038	1.715	1.538286	1.906448
435170.75	2.0175989	1.8863332	2.155591	2.0150001	1.8230478	2.2216661
371302.06	2.8144202	2.6463459	2.9903705	2.7550001	2.5297368	2.9949422
475847.31	3.3203928	3.1586695	3.4882503	3.2349999	2.990499	3.4941642
823523.88	2.8973053	2.7822058	3.0159435	2.855	2.6255984	3.0990767
200130.28	3.332829	3.0846655	3.595643	3.28	3.033771	3.5408907
563743.13	3.512238	3.3592217	3.6704273	3.385	3.1347847	3.6498742

712899.88	2.9569371	2.8320429	3.0859206	2.8699999	2.6399841	3.1146908
417822.09	3.2095001	3.0399985	3.3859923	3.1949999	2.952045	3.4526193
459546.28	3.1639903	3.0034311	3.3309035	3.02	2.7839258	3.2707438
420770.59	3.5197327	3.3427355	3.7036688	3.4000001	3.1492202	3.6654382
383326.22	2.8278785	2.6620202	3.0013647	2.77	2.5441113	3.0105672
491254	3.3119323	3.1529393	3.4768662	3.2349999	2.990499	3.4941642
850486.94	3.1334991	3.0156488	3.2547746	3.125	2.8847742	3.3798923
206566.42	3.2338266	2.993212	3.4886355	3.2	2.9568512	3.457813
581936.63	3.2649603	3.1197867	3.4151459	3.1800001	2.9376273	3.4370375
736017.69	3.1086209	2.9825366	3.2386653	3.04	2.8031294	3.2915397
431298.94	3.517282	3.3424926	3.6988401	3.5150001	3.2599339	3.7847214
474411.72	3.0395539	2.8846769	3.2005858	2.9349999	2.7023399	3.1823328
434359.84	3.1701825	3.0049312	3.3421578	3.095	2.855953	3.3487144
370564.78	2.9495518	2.7772601	3.1297336	2.915	2.6831503	3.1615229
474935.19	3.316242	3.1544678	3.4841621	3.26	3.0145376	3.5201249
822554.56	2.9359756	2.8200352	3.0554585	2.845	2.616009	3.0886667
199649.31	3.5963058	3.3380291	3.8692617	3.5799999	3.3225422	3.8521116
562479.56	3.2978976	3.1495118	3.4514694	3.2249999	2.9808846	3.483779
711608.88	3.2742705	3.1426578	3.4099791	3.2049999	2.9616575	3.4630065
416914.66	3.5354958	3.3572896	3.720705	3.55	3.2936432	3.8210111
458625.84	3.0896645	2.9308698	3.2548265	3.02	2.7839258	3.2707438
419850.13	3.289269	3.1180553	3.4674389	3.23	2.9856915	3.4889715
382468.69	2.5518429	2.3942387	2.7170961	2.5250001	2.3095498	2.7551384
490256.03	2.853611	2.7060204	3.0071585	2.8150001	2.587245	3.0574317
849428.94	2.5181625	2.4125664	2.6271904	2.51	2.2952049	2.7394838
206034.88	3.0334671	2.8002775	3.2808933	3.01	2.7743249	3.2603452
580561.19	2.8455226	2.7099452	2.9861269	2.7850001	2.5584874	3.0261903
734581.81	2.6042027	2.4887989	2.723577	2.5799999	2.3621638	2.8125219
430309.19	3.0443227	2.8816793	3.2137549	3.04	2.8031294	3.2915397
473380.84	2.5962183	2.4530807	2.7455289	2.5450001	2.3286791	2.7760081
433317.81	2.7508678	2.5969014	2.9115787	2.7	2.4770448	2.9376361
381992.03	2.7461305	2.5824428	2.917474	2.7449999	2.5201547	2.9845247
489570.34	3.1660414	3.0103695	3.3276761	3.1500001	2.9087958	3.4058697
848670.31	2.6971605	2.5877883	2.809967	2.6400001	2.4195898	2.8750937
205753.13	3.3000715	3.0564864	3.557905	3.3050001	3.057816	3.5668449
579863.38	2.6454508	2.5147078	2.7812281	2.625	2.4052305	2.8594534
733594	2.6472409	2.5307992	2.7676582	2.635	2.4148033	2.8698804
429756.53	3.2506778	3.0824318	3.4257193	3.27	3.0241539	3.530508
472801.44	2.2334957	2.1007996	2.3723769	2.2	1.9992132	2.4154904
432800.59	2.7911236	2.6359274	2.9530723	2.7750001	2.5489032	3.015775
344589.81	2.5450549	2.37938	2.7192247	2.54	2.3238964	2.7707911
441567.81	2.9599078	2.8015954	3.124836	2.9549999	2.7215321	3.2031398
765898.13	2.8097732	2.6923018	2.9310513	2.75	2.5249457	2.9897335
185591.08	2.7048714	2.4734001	2.9521718	2.72	2.4962029	2.9584775
523096.09	2.8407784	2.6981611	2.9889767	2.8199999	2.5920386	3.062638
661747	2.6278925	2.5058188	2.7543752	2.625	2.4052305	2.8594534
387645.81	3.1188264	2.9454794	3.2997122	3.165	2.9232109	3.4214542
426515.75	2.4055383	2.2605786	2.5573554	2.405	2.1948473	2.6298461
390464.03	3.0092402	2.8396161	3.1863494	3.01	2.7743249	3.2603452
381128.91	3.0488372	2.8760364	3.2293072	3.04	2.8031294	3.2915397
488401.22	3.398845	3.2372899	3.566375	3.3800001	3.1299729	3.644686
847434.13	3.6628215	3.5350873	3.7939918	3.47	3.2166026	3.7380538
205252.45	3.3714578	3.1249077	3.6322896	3.3800001	3.1299729	3.644686
578560	3.5622926	3.4101424	3.7194827	3.5150001	3.2599339	3.7847214
731966.81	3.37447	3.242691	3.5102298	3.3499999	3.1011064	3.6135533
428731.72	3.1931391	3.026212	3.3668785	3.22	2.9760776	3.478586
471757.97	2.9209893	2.7687821	3.0793874	2.875	2.6447797	3.119895
431867.53	3.4455011	3.2726395	3.625123	3.415	3.1636569	3.6810009
368511.25	2.5562313	2.3955779	2.7248249	2.5450001	2.3286791	2.7760081
472227.03	3.0070281	2.8526418	3.1675985	2.98	2.7455263	3.2291448
819607.31	3.2100739	3.0885737	3.3351288	3	2.7647247	3.2499456
198435.53	2.8119965	2.5834889	3.0552974	2.8299999	2.6016262	3.07305
559377.25	3.1302667	2.9853497	3.2803998	3.105	2.8655593	3.3591075

707747.94	2.7241337	2.6038804	2.8485079	2.7	2.4770448	2.9376361
414506	2.7936869	2.6350782	2.9593472	2.825	2.5968323	3.0678442
456152.78	2.5890448	2.4434721	2.7410247	2.55	2.333462	2.781225
417547.56	2.7877064	2.6298397	2.9525733	2.75	2.5249457	2.9897335
380450.03	2.8545141	2.6872456	3.0294683	2.845	2.616009	3.0886667
487529.69	2.8141875	2.6672297	2.9671361	2.8050001	2.5776584	3.0470188
846428.06	2.7810986	2.6698749	2.8957658	2.6700001	2.4483137	2.9063685
204843.89	2.777725	2.5541489	3.0156298	2.79	2.5632799	3.0313978
577488.44	2.76196	2.628062	2.9009123	2.74	2.5153639	2.9793155
730692.75	3.1449606	3.0176773	3.276233	3.1300001	2.8895781	3.385088
427909.09	2.6173785	2.4663165	2.7752721	2.6500001	2.4291637	2.8855193
470948.66	2.5586653	2.4162202	2.7073159	2.51	2.2952049	2.7394838
431065.31	2.7582827	2.6037095	2.919636	2.73	2.5057831	2.9688969
367846.81	3.2486348	3.0670335	3.4381812	3.25	3.0049217	3.5097411
471391.41	3.8121188	3.637882	3.9925444	3.7750001	3.5104945	4.0541544
818675.63	3.5374205	3.4097459	3.6686528	3.4200001	3.1684697	3.6861882
198040.33	3.4639409	3.2095435	3.7331412	3.5250001	3.2695644	3.7950907
558355.69	3.0213716	2.8788989	3.1690705	3.0050001	2.7695248	3.2551455
706538.38	3.1703868	3.040441	3.3044584	3.145	2.9039912	3.4006746
413705.03	3.3647161	3.1902595	3.5462322	3.405	3.1540322	3.6706259
455374.59	2.9799643	2.8235033	3.1428392	2.9349999	2.7023399	3.1823328
416790.31	3.3709996	3.197017	3.5519891	3.3499999	3.1011064	3.6135533
379755.94	2.8676312	2.6998222	3.0431397	2.865	2.6351886	3.1094863
486650.25	3.2179167	3.0604944	3.3813372	3.2149999	2.9712708	3.473393
845470.94	2.5476925	2.441227	2.6576061	2.585	2.3669481	2.8177373
204427.81	3.0132885	2.7799981	3.2609291	3.0599999	2.8223357	3.3123326
576420.56	2.7323799	2.599088	2.8707359	2.73	2.5057831	2.9688969
729464	2.6882753	2.5705965	2.809952	2.6949999	2.4722559	2.9324255
427057.63	2.9855456	2.8238981	3.1540344	3.03	2.7935274	3.281142
470132.44	2.5843782	2.4410863	2.733886	2.5599999	2.3430285	2.7916582
430274.19	3.1770439	3.0108385	3.3500373	3.1800001	2.9376273	3.4370375
379383.75	2.4065342	2.2529449	2.5678377	2.4100001	2.1996241	2.6350691
486207.25	2.6285088	2.4863572	2.7766693	2.645	2.4243767	2.8803065
845013.25	1.9786671	1.8849505	2.0758374	1.975	1.7850106	2.1797056
204204.7	2.6786847	2.4588792	2.9128675	2.7249999	2.5009928	2.9636872
575867.94	2.3112938	2.1887774	2.4388828	2.3199999	2.1136804	2.5410171
728812.31	2.4190042	2.3073926	2.5346189	2.425	2.2139554	2.650737
426612.84	2.3088851	2.1669288	2.4576983	2.355	2.1470938	2.5776021
469710.16	2.1502621	2.0196793	2.2870724	2.155	1.9563268	2.3683791
429854.63	2.5683103	2.4190261	2.7243958	2.5699999	2.3525958	2.8020906
366793.44	3.4160917	3.2295389	3.6106105	3.425	3.1732824	3.6913755
470096.38	3.5843713	3.4152513	3.7596991	3.595	3.3369932	3.86766
817306.19	3.1567116	3.0360675	3.2809207	3.1500001	2.9087958	3.4058697
197410.39	3.4040761	3.1515343	3.6714697	3.4400001	3.1877213	3.7069361
556753.38	3.4755065	3.3223596	3.6338921	3.4749999	3.2214167	3.7432396
704699.31	3.1417656	3.0122468	3.2754209	3.145	2.9039912	3.4006746
412439.09	3.101064	2.9334209	3.2757907	3.1900001	2.9472389	3.4474254
454154.56	3.0452187	2.8868215	3.2100468	3.0050001	2.7695248	3.2551455
415577.94	3.6695886	3.4877031	3.8584986	3.7	3.4381831	3.9764676
378626.5	3.0927577	2.9181328	3.2751021	3.0899999	2.85115	3.3435173
485262.81	3.8947966	3.7211657	4.0744386	3.925	3.6551962	4.2094493
844007.75	3.1741414	3.0550721	3.2966623	3.22	2.9760776	3.478586
203747.5	3.6368544	3.3796761	3.9084139	3.72	3.4574633	3.9971869
574688.75	3.561928	3.4092798	3.7196503	3.55	3.2936432	3.8210111
727487.63	3.2550383	3.1252418	3.3888407	3.2550001	3.0097294	3.5149331
425715.22	3.3214693	3.1505821	3.4992161	3.415	3.1636569	3.6810009
468815.72	3.2870059	3.1249208	3.4553175	3.26	3.0145376	3.5201249
428968.78	3.6692648	3.4902132	3.8551207	3.6949999	3.4333632	3.9712877
366016.03	3.9069326	3.707037	4.1148067	3.9549999	3.6841488	4.2404962
469086.63	4.3765903	4.1893005	4.570096	4.4400001	4.1527362	4.741899
816254.88	3.4633789	3.3368745	3.593452	3.53	3.27438	3.8002751
196927.89	4.1436486	3.8641825	4.4379854	4.29	4.0077119	4.586926
555517.94	4.0916772	3.9251792	4.263422	4.1149998	3.8386283	4.406013

703296.25	3.7310023	3.5895987	3.8765485	3.75	3.4863875	4.0282621
411495.22	3.9101305	3.7213862	4.1059675	4.04	3.7662024	4.3284407
453200.56	3.9386535	3.7580364	4.1257081	3.9000001	3.631072	4.1835742
414651.06	4.4712296	4.2699971	4.6794963	4.5	4.2107697	4.8038645
377786.47	3.1975734	3.0197773	3.3831053	3.23	2.9856915	3.4889715
484159.97	3.4864509	3.3220954	3.6568336	3.53	3.27438	3.8002751
842894.38	2.8864827	2.7729151	3.0035076	2.9100001	2.6783533	3.1563201
203229.17	3.670733	3.4120145	3.9438686	3.8050001	3.5394263	4.0852218
573368.94	3.2579372	3.1118546	3.4091072	3.27	3.0241539	3.530508
725968.31	3.0607398	2.9347863	3.1907084	3.075	2.8367422	3.3279257
424686.97	3.3507032	3.1788509	3.5294316	3.48	3.2262309	3.7484255
467779.91	2.9650695	2.8110609	3.1253216	2.95	2.7167337	3.1979382
427950.44	3.8509133	3.6672132	4.0414329	3.8900001	3.6214232	4.173223
377366.47	3.4290276	3.2447176	3.621079	3.46	3.206975	3.7276819
483630.53	3.610194	3.442822	3.7835987	3.675	3.4140851	3.9505661
842337.63	2.8658342	2.7526405	2.9824874	2.8800001	2.6495752	3.1250992
202971.58	3.2763207	3.0320055	3.5350819	3.355	3.1059172	3.6187425
572711.06	3.1761217	3.0318241	3.3255129	3.2	2.9568512	3.457813
725209.25	3.1066895	2.9797187	3.2376797	3.115	2.8751664	3.3695002
424178.13	3.5551102	3.3779213	3.7391818	3.675	3.4140851	3.9505661
467275.81	3.0089295	2.8536885	3.1704204	3.0150001	2.7791252	3.2655447
427442.03	3.7619135	3.5802681	3.9503872	3.8199999	3.553894	4.1007538
340480.78	3.3276474	3.1366813	3.5271995	3.405	3.1540322	3.6706259
436369.44	3.7743249	3.5942242	3.9611132	3.8699999	3.6021268	4.1525197
760355.06	2.8960154	2.7763064	3.0195584	3.04	2.8031294	3.2915397
183109.91	3.4023283	3.1403723	3.6803036	3.49	3.2358596	3.7587965
516735.88	3.5588782	3.3980627	3.7253387	3.6099999	3.3514457	3.8832073
654384.88	3.0287986	2.89691	3.1651437	3.0550001	2.817534	3.3071346
382677.28	3.5539083	3.367516	3.7479331	3.6700001	3.409266	3.9453855
421620.84	3.3181472	3.1465304	3.4966905	3.355	3.1059172	3.6187425
385657.16	3.5627499	3.3768356	3.7562375	3.615	3.3562632	3.8883896
376583.66	3.3379037	3.1559069	3.5276592	3.425	3.1732824	3.6913755
482633.81	3.9470918	3.7718158	4.1284113	4.0599999	3.7855136	4.3491292
841330.19	3.0225945	2.9062467	3.1424055	3.085	2.8463473	3.3383203
202503.97	3.7431364	3.4813735	4.0193667	3.8800001	3.6117747	4.1628718
571500.94	3.4365647	3.2862434	3.591989	3.5150001	3.2599339	3.7847214
723808.19	3.2080321	3.0788622	3.341229	3.2650001	3.0193455	3.5253165
423195.94	3.3695977	3.1969557	3.5491397	3.52	3.2647491	3.789906
466331.34	3.3838601	3.2189424	3.555037	3.46	3.206975	3.7276819
426528.59	4.0958567	3.9060228	4.292531	4.1500001	3.8724351	4.4422054
364055.25	2.8622029	2.6910331	3.0414059	2.9549999	2.7215321	3.2031398
466572.56	3.1977878	3.0375664	3.3642664	3.2750001	3.0289624	3.5356996
813708	2.4738605	2.36696	2.5843444	2.605	2.3860877	2.8385971
195744.67	2.8608696	2.6287973	3.1079383	2.98	2.7455263	3.2291448
552474.19	2.8797727	2.7399898	3.0248384	2.9449999	2.7119355	3.1927366
699770.13	2.7637649	2.6419492	2.8897481	2.79	2.5632799	3.0313978
409055.72	2.9995914	2.8340816	3.1722448	3.1199999	2.8799703	3.3746963
450822.53	2.8348184	2.6815095	2.9946079	2.8800001	2.6495752	3.1250992
412307.97	3.199065	3.0287306	3.3764844	3.28	3.033771	3.5408907
375804.38	3.3953836	3.211617	3.5869243	3.5050001	3.2503037	3.7743518
481631.31	3.6376371	3.4692786	3.8120537	3.74	3.4767456	4.0179043
840341.56	2.9571309	2.8419952	3.0757339	3.085	2.8463473	3.3383203
202049.52	3.4051058	3.1553862	3.6693344	3.5	3.2454889	3.7691667
570299.75	3.3245676	3.1765895	3.4776599	3.4200001	3.1684697	3.6861882
722406.44	3.1575024	3.0292411	3.2897985	3.22	2.9760776	3.478586
422208.69	3.4035301	3.2298098	3.5841668	3.595	3.3369932	3.86766
465389.78	3.3283927	3.164686	3.4983721	3.4200001	3.1684697	3.6861882
425599.13	3.8157973	3.6324487	4.0060034	3.9349999	3.6648464	4.2197986
363322.63	3.5588205	3.3674614	3.7582207	3.5899999	3.332176	3.8624773
465634.13	3.7969725	3.6220272	3.9781837	3.8699999	3.6021268	4.1525197
812770.94	3.0599027	2.9408133	3.1825774	3.0799999	2.8415446	3.333123
195322.97	3.7220404	3.4563627	4.0027213	3.865	3.5973032	4.1473436
551369.25	3.3081279	3.1580367	3.4635098	3.395	3.1444082	3.6602504

698478.13	3.1454098	3.0152454	3.2797477	3.1949999	2.952045	3.4526193
408158.13	3.5451946	3.3648617	3.7326815	3.72	3.4574633	3.9971869
449955.22	3.5514646	3.3794515	3.7299643	3.575	3.3177254	3.8469284
411458.91	4.0198426	3.828428	4.2183495	4.0799999	3.8048265	4.3698158
375043.25	3.0289841	2.8553836	3.2103786	3.105	2.8655593	3.3591075
480664.22	3.2434285	3.0844066	3.4085231	3.3499999	3.1011064	3.6135533
839359.88	2.5447965	2.4380095	2.6550567	2.6500001	2.4291637	2.8855193
201599.88	3.1150813	2.8761792	3.3685331	3.25	3.0049217	3.5097411
569174.38	2.9586716	2.8190327	3.1034374	3.0450001	2.8079307	3.2967381
721070	2.6335862	2.5164557	2.7547617	2.6949999	2.4722559	2.9324255
421298.94	3.1402881	2.9733326	3.314177	3.27	3.0241539	3.530508
464494.59	2.8374927	2.6863537	2.9949205	2.8699999	2.6399841	3.1146908
424715.53	3.3928592	3.2199209	3.5726728	3.49	3.2358596	3.7587965
374665.91	2.818511	2.6510582	2.9937692	2.9100001	2.6783533	3.1563201
480184.16	2.9530337	2.8013141	3.1108348	3.0550001	2.817534	3.3071346
838895.94	2.2470009	2.146698	2.350781	2.395	2.1852949	2.6193991
201375.77	3.0241971	2.7887475	3.2742152	3.135	2.8943825	3.3902838
568611.13	2.7435269	2.6090574	2.8831303	2.8299999	2.6016262	3.07305
720407	2.3375676	2.2272425	2.4519432	2.405	2.1948473	2.6298461
420832.63	3.0178268	2.8541148	3.1884818	3.135	2.8943825	3.3902838
464039.16	2.7152882	2.5674129	2.8694596	2.78	2.5536952	3.0209827
424279	3.240792	3.071739	3.4167287	3.355	3.1059172	3.6187425
362236.34	3.0587764	2.8813002	3.2443237	3.1700001	2.9280162	3.4266489
464260.25	3.3860319	3.2206976	3.5576534	3.5350001	3.2791955	3.8054593
811409.38	2.7852771	2.671617	2.9025297	2.9449999	2.7119355	3.1927366
194681.09	3.1487393	2.9043784	3.408169	3.29	3.0433886	3.5512729
549745.88	3.2396786	3.09095	3.3937144	3.3099999	3.0626256	3.5720353
696566.88	2.9444408	2.818377	3.0746908	3.0650001	2.8271377	3.3175304
406830.78	3.4412341	3.263314	3.6263328	3.5999999	3.3418105	3.8728426
448660.25	3.0691376	2.9091535	3.2356312	3.1600001	2.9184058	3.4162595
410194.88	3.5958519	3.4146643	3.7841575	3.71	3.447823	3.9868276
373922.25	3.2493386	3.0691779	3.4373147	3.355	3.1059172	3.6187425
479234	3.7664273	3.5946562	3.9442856	3.97	3.6986265	4.2560182
837933.75	3.1028707	2.9847367	3.2244816	3.23	2.9856915	3.4889715
200938.47	3.5383966	3.2830541	3.8083248	3.71	3.447823	3.9868276
567453.06	3.6408298	3.4855113	3.8012865	3.7550001	3.4912088	4.0334406
719066.25	3.3543501	3.221807	3.4909461	3.4649999	3.2117887	3.732868
419901.09	3.5174949	3.340374	3.7015693	3.6949999	3.4333632	3.9712877
463133.63	3.41802	3.2516975	3.5906448	3.5250001	3.2695644	3.7950907
423396.63	3.9159501	3.7297049	4.1090875	4.0599999	3.7855136	4.3491292
361477.09	3.978122	3.7751436	4.1891789	4.1399999	3.8627756	4.4318652
463278.59	4.2134476	4.0285845	4.4046054	4.4850001	4.19626	4.7883744
810392.5	3.5365579	3.4082541	3.6684556	3.6900001	3.4285436	3.9661074
194213.8	4.6340685	4.3362207	4.9469857	4.9749999	4.6706409	5.2939854
548540.31	4.1801848	4.0108213	4.3548622	4.3000002	4.0173774	4.5972605
695164.19	4.02351	3.875767	4.1754427	4.1849999	3.9062469	4.4783931
405872.88	4.5014095	4.2973447	4.7126617	4.7600002	4.4623947	5.0722346
447730.78	3.8549953	3.6752539	4.0412536	3.9349999	3.6648464	4.2197986
409265.59	4.6180282	4.4121552	4.8310275	4.7750001	4.4769187	5.0877109
373079.19	3.5649269	3.3758881	3.7617953	3.7	3.4381831	3.9764676
478150.66	3.7268589	3.5558119	3.9040074	3.905	3.6358967	4.1887493
836808.19	2.9851525	2.8692257	3.1045613	3.085	2.8463473	3.3383203
200415.52	3.7721632	3.5080278	4.0509167	4.0100002	3.7372386	4.2974048
566118.06	3.5434306	3.3900478	3.7019641	3.645	3.3851719	3.9194801
717522.44	3.3462367	3.2137146	3.4828205	3.4549999	3.2021613	3.7224956
418842.09	3.7603669	3.5769274	3.9507756	4.0700002	3.7951698	4.3594723
462117.81	3.4168775	3.2504029	3.5896685	3.53	3.27438	3.8002751
422366.06	4.0628266	3.8728502	4.2597113	4.1700001	3.8917556	4.4628849
372577.25	4.0743227	3.8719175	4.284564	4.25	3.9690535	4.5455852
477518.94	4.2364812	4.0538626	4.4252067	4.415	4.1285596	4.7160759
836194.5	3.4262364	3.3019135	3.5540423	3.54	3.2840114	3.8106434
200111.75	4.3625627	4.0779347	4.6618185	4.6799998	4.3849468	4.9896841
565317.5	3.988909	3.825953	4.157021	4.1599998	3.8820951	4.4525456

716614.94	3.8319044	3.6899087	3.9779651	3.95	3.679323	4.235322
418210.81	4.3805656	4.1822472	4.5858598	4.6599998	4.3655882	4.9690433
461506.75	3.618582	3.4470918	3.7963953	3.71	3.447823	3.9868276
421767.31	4.5546441	4.3532233	4.76298	4.7600002	4.4623947	5.0722346
336135.5	4.2185369	4.0017986	4.4439626	4.4250002	4.1382298	4.7264056
430813.41	4.2965236	4.1029992	4.496819	4.5650001	4.2736535	4.8709793
754777.69	3.4235246	3.2927835	3.5581257	3.54	3.2840114	3.8106434
180512.27	4.420752	4.1193118	4.7384176	4.6799998	4.3849468	4.9896841
510017.84	3.9880958	3.8166444	4.1652646	4.1399999	3.8627756	4.4318652
646577.13	3.8819809	3.7315836	4.0368848	4.0050001	3.7324119	4.292232
377264.16	4.6466117	4.4316158	4.8693409	4.9899998	4.6851749	5.3094506
416383.56	3.873832	3.6870692	4.0676045	4.0050001	3.7324119	4.292232
380510.88	4.5701718	4.3578734	4.7901378	4.77	4.4720774	5.082552
371738.5	3.9140418	3.7154877	4.1204505	4.0799999	3.8048265	4.3698158
476451.69	4.5419087	4.3525414	4.7373948	4.8449998	4.5447068	5.1599216
835118.38	3.2797747	3.1580851	3.4049523	3.4349999	3.1829081	3.7017493
199609.75	4.4486804	4.1608548	4.7511697	4.73	4.4333491	5.0412807
564041.63	3.8631902	3.7026703	4.0288782	4.02	3.7468927	4.3077507
715110.94	3.9644198	3.8198166	4.1130962	4.1300001	3.8531163	4.421525
417176.5	4.2571907	4.0614781	4.459897	4.5149999	4.2252798	4.8193541
460507.56	3.7740965	3.5987284	3.9558005	3.96	3.6889746	4.2456703
420803.78	4.5151687	4.3144054	4.7228627	4.7399998	4.4430308	5.051599
359365.03	3.0247796	2.847614	3.2100813	3.085	2.8463473	3.3383203
460589.25	3.2197886	3.0579824	3.3879335	3.4000001	3.1492202	3.6654382
807687.81	2.7622058	2.6487646	2.8792562	2.845	2.616009	3.0886667
192940.06	3.4155686	3.1597352	3.6865995	3.635	3.3755352	3.909117
545261.63	3.0847578	2.9390824	3.2357848	3.1700001	2.9280162	3.4266489
691337.88	3.0607321	2.9316959	3.1939852	3.1949999	2.952045	3.4526193
403236.53	3.4396684	3.2610087	3.625571	3.6849999	3.4237239	3.960927
445187.41	2.9111335	2.7547796	3.0740497	3.01	2.7743249	3.2603452
406777.47	3.4441435	3.2661357	3.6293309	3.6300001	3.370717	3.9039354
370983.28	3.2481248	3.0672963	3.436831	3.415	3.1636569	3.6810009
475479.75	3.4933138	3.327318	3.6654472	3.71	3.447823	3.9868276
834132.81	2.9251935	2.8102665	3.0436144	3.0650001	2.8271377	3.3175304
199145.28	4.3084126	4.0249128	4.6066127	4.5799999	4.2881675	4.8864651
562886.69	3.2741935	3.1264009	3.4271686	3.405	3.1540322	3.6706259
713711.25	3.3991337	3.2652051	3.5371454	3.5599999	3.3032756	3.8313785
416215.13	3.649555	3.4683104	3.8378139	3.9100001	3.6407213	4.1939244
459584.31	3.1397939	2.9798641	3.3060772	3.2850001	3.0385797	3.546082
419909.09	3.562676	3.3844082	3.7478964	3.835	3.5683627	4.1162848
346715.97	4.9810224	4.7488456	5.2216144	5.1199999	4.8111682	5.4434557
444389.19	4.680582	4.4815736	4.8861518	4.8449998	4.5447068	5.1599216
779861.25	4.1725373	4.0303917	4.3184156	4.1900001	3.9110775	4.4835625
186098	4.411654	4.1150041	4.7240396	4.71	4.4139872	5.0206432
526087.13	4.0221477	3.8525796	4.1972575	4.1550002	3.877265	4.4473758
667064.94	4.3683901	4.2112069	4.5299397	4.4850001	4.19626	4.7883744
388957.13	4.44522	4.2381368	4.6598048	4.5749998	4.2833295	4.8813033
429528.09	4.8238988	4.6184053	5.0361805	4.9050002	4.6028233	5.2218037
392438.19	4.6580586	4.446949	4.8766012	4.8000002	4.5011268	5.113502
2329793.5	4.2802076	4.1966066	4.3650551	4.5094337	4.1141863	4.9324064
2409566.5	4.3219395	4.2393255	4.4057584	4.5566039	4.1592469	4.9816833
2412352.3	5.1000013	5.0102763	5.1909299	5.3867927	4.9539595	5.8473148
2258825.3	4.5474963	4.4599752	4.6363034	4.8018866	4.3937345	5.237751
2417419	3.6592746	3.5834119	3.7363389	3.8490567	3.4845865	4.2412858
2340897.8	1.3990359	1.3515269	1.4477887	1.4716982	1.2498163	1.7216159
2421247.3	1.2171413	1.1735898	1.2618955	1.2830188	1.0764571	1.5176677
2346298.8	1.4789251	1.4301226	1.5289681	1.5566038	1.3281502	1.8130734
2428317	1.517512	1.468907	1.5673153	1.5943396	1.3630229	1.853664
2431816.3	1.5169731	1.4684117	1.5667315	1.5943396	1.3630229	1.853664
2356767.5	1.8979386	1.8427216	1.9543899	1.990566	1.7310239	2.2780428
2438567.3	1.9089078	1.8544602	1.9645481	2	1.7398219	2.2881112
2362105.5	1.9588456	1.9028062	2.0161164	2.0471699	1.7838342	2.3384314
2443050.8	1.8006176	1.7477964	1.8546294	1.8962264	1.6431288	2.177274

2444796.3	1.8819565	1.8279659	1.9371368	1.9622642	1.704639	2.2478285
2209046	1.997695	1.9391848	2.0575218	2.0943396	1.8278828	2.3887155
2448692.8	2.237112	2.1782584	2.297153	2.3396227	2.0574739	2.6496575
2372738.8	2.056695	1.999391	2.1152248	2.1509433	1.880787	2.4490108
2455281	2.1406918	2.0832057	2.1993623	2.2358491	1.9602336	2.5393639
2379231.5	2.4726472	2.4098616	2.5366545	2.5849056	2.287869	2.9098005
2461950.5	2.4070346	2.3461359	2.4691141	2.518868	2.2257674	2.839834
2464972.3	2.2393761	2.180686	2.2992456	2.3396227	2.0574739	2.6496575
2388332.5	2.6717386	2.6065822	2.7381117	2.7830188	2.4744663	3.1194098
2470617	2.7398014	2.674917	2.8058622	2.8490567	2.5367572	3.1891882
2392957.3	3.1730614	3.1020873	3.2452495	3.3018868	2.9649866	3.6665821
2474832.8	2.8709011	2.8045294	2.9384468	2.990566	2.6703813	3.3385704
2477466.8	2.8008449	2.7353275	2.8675351	2.9245284	2.6079998	3.2688823
2239647	2.9531441	2.8823972	3.0251884	3.0754716	2.7506452	3.4281108
2482580.5	3.2482331	3.1777196	3.3199167	3.3962264	3.0544181	3.7658229
2404949.3	2.9426816	2.874517	3.0120547	3.0660377	2.7417238	3.418165
2488117.5	3.1726797	3.1030729	3.2434542	3.3018868	2.9649866	3.6665821
2410613.8	3.1187079	3.0486045	3.1900165	3.2547169	2.9202971	3.6169355
2493946.8	3.0297358	2.961803	3.098834	3.1603773	2.8309727	3.5175879
2496805.3	3.0310733	2.9631641	3.1001465	3.1603773	2.8309727	3.5175879
2419254.3	3.1613047	3.090847	3.2329636	3.2924528	2.9560473	3.6566541
2502558.5	3.3461754	3.2748859	3.4186258	3.481132	3.1349645	3.855082
2423913.8	3.9774518	3.8984485	4.0576534	4.1320753	3.7541151	4.5377793
2506625.8	3.7951417	3.7192566	3.8721855	3.9528301	3.5833578	4.3500557
2508317	4.0868039	4.008069	4.1666965	4.2547169	3.8710549	4.6661162
2267192.5	4.1011076	4.0181665	4.1853294	4.2641511	3.8800535	4.6759849
2512812.5	4.2530031	4.1727471	4.334415	4.4150944	4.0240984	4.8338194
2434201	3.4598622	3.3863597	3.5345581	3.5943396	3.2424417	3.9740126
2518528.3	3.6374419	3.563333	3.7127039	3.7830188	3.4217672	4.1720338
2359038.8	3.9367731	3.8571088	4.0176682	4.0943398	3.7181506	4.4982738
1148503.1	4.4405627	4.3195181	4.5641389	4.21	3.8174026	4.6320148
1181290.3	4.1243038	4.0092978	4.2417717	5.1999998	4.7626052	5.6667652
1187958.8	4.4572253	4.3379698	4.5789289	3.8699999	3.4939868	4.2754507
1221607.8	4.1903796	4.0763655	4.3067737	4.8200002	4.3992481	5.2701383
1189549.3	5.3642168	5.2333984	5.4974785	4.5	4.09378	4.9356217
1222802.9	4.8429718	4.7204022	4.9679189	5.5599999	5.1073904	6.0419655
1113958.5	4.8287258	4.7005363	4.9595251	3.9100001	3.5319989	4.3174362
1144866.9	4.2738595	4.1549373	4.3953218	4.9499998	4.5234828	5.4058976
1192277.1	3.9118421	3.8003721	4.0257516	4.0700002	3.6841471	4.4852781
1225141.9	3.4134822	3.310802	3.5185373	5.5	5.0498915	5.9794669
1154582.5	1.4568037	1.3880073	1.5281274	2.02	1.7510258	2.3185995
1186315.3	1.3428134	1.2776743	1.4104128	2.77	2.4533694	3.1161573
1194413.4	1.2675679	1.2045152	1.3330648	1.65	1.4078392	1.9218578
1226834	1.1680472	1.1083466	1.230128	2.1199999	1.8442112	2.4253979
1157617.1	1.5272752	1.4569061	1.6001648	2.1199999	1.8442112	2.4253979
1188681.8	1.4318383	1.3646145	1.5015168	2.5699999	2.2653699	2.9041789
1198244.9	1.6232074	1.5518634	1.6969854	1.76	1.5095785	2.0400949
1230072.1	1.4145513	1.3488597	1.4826148	2.3199999	2.0310485	2.6385314
1200101.6	1.5965315	1.5258368	1.6696564	1.5700001	1.3340214	1.8356942
1231714.5	1.4394569	1.3732265	1.508056	2.1400001	1.8628677	2.4467382
1163179.8	1.969601	1.8897666	2.0519412	2.8299999	2.5098543	3.1796663
1193587.8	1.8281019	1.7521939	1.9064522	3.3	2.9535189	3.6759591
1203680.8	1.9706222	1.8921082	2.0515575	2.74	2.4251411	3.0843887
1234886.5	1.8487529	1.773687	1.9261791	3.46	3.1049876	3.8444781
1166011.6	2.0377154	1.956596	2.1213346	2.8	2.4816072	3.1479163
1196094	1.8819591	1.8050094	1.9613459	3.52	3.1618392	3.9076219
1206058	1.9518132	1.8737549	2.0322881	2.3900001	2.0965767	2.7129941
1236992.8	1.6532029	1.5823197	1.7264433	2.96	2.6323643	3.3171437
1207045.8	1.9643	1.8860215	2.0449929	2.3499999	2.059124	2.6704521
1237750.5	1.8016555	1.7276467	1.8780193	3.1600001	2.8211553	3.5283346
1090750.9	2.1599798	2.0736327	2.2489991	2.3399999	2.0497642	2.6598134
1118294.9	1.8394076	1.7607685	1.9206539	3.1800001	2.840054	3.5494339
1209188	2.3222196	2.2371135	2.4097345	2.9200001	2.594651	3.2748606

1239504.8	2.1540861	2.0731466	2.237376	3.73	3.3610268	4.1284199
1171815.4	2.1419756	2.0589902	2.2274475	2.5999999	2.2935407	2.9360044
1200923.4	1.9734814	1.8948207	2.054569	3.0899999	2.7550368	3.454459
1212728.8	2.2115417	2.1286275	2.2968578	2.53	2.2278254	2.861728
1242552.1	2.071543	1.9922806	2.1531498	3.03	2.6983991	3.3911021
1175286.5	2.5432098	2.4528451	2.6360521	3.0999999	2.7644796	3.4650154
1203945	2.4037642	2.3169765	2.4929712	3.49	3.13341	3.8760533
1216268.9	2.5668666	2.477608	2.6585193	2.6300001	2.321722	2.9678197
1245681.6	2.2509766	2.1684232	2.3358681	3	2.6700928	3.3594112
1217857.3	2.3524926	2.2671311	2.4402454	2.1300001	1.8535388	2.436069
1247115	2.1289136	2.0486963	2.2114668	2.5599999	2.2559819	2.8935678
1180085.1	2.7692916	2.6751511	2.8658996	3.1500001	2.811707	3.5177834
1208247.5	2.5764589	2.4867384	2.6685894	3.48	3.1239352	3.8655291
1220817.4	2.823518	2.7300384	2.9193823	3.1600001	2.8211553	3.5283346
1249799.5	2.6580265	2.5683992	2.7499828	3.6600001	3.2945964	4.0548549
1182504.4	3.2397344	3.1379485	3.343981	3.6600001	3.2945964	4.0548549
1210452.9	3.1079278	3.0093987	3.2088609	4.2800002	3.8840725	4.7053409
1223086.4	2.9221158	2.8270919	3.0195193	2.9300001	2.6040778	3.2854328
1251746.4	2.820859	2.7285757	2.9154677	3.5799999	3.2187178	3.9707391
1224550.4	2.871258	2.7771273	2.9677656	2.99	2.6606591	3.3488457
1252916.4	2.7320259	2.6412613	2.8251138	3.5899999	3.2282002	3.981256
1107085	3.0774512	2.974973	3.1825588	3.1099999	2.7739234	3.4755707
1132562.1	2.8316329	2.7344697	2.9313667	3.6600001	3.2945964	4.0548549
1227292.5	3.4514999	3.348335	3.5570354	3.1600001	2.8211553	3.5283346
1255288	3.0494995	2.9536531	3.1476641	3.8699999	3.4939868	4.2754507
1189030.8	3.1100962	3.0106556	3.2119844	2.5699999	2.2653699	2.9041789
1215918.5	2.778969	2.6860509	2.8742816	3.28	2.9345999	3.65488
1230286.5	3.2911034	3.1905038	3.3940678	3.0699999	2.7361538	3.4333436
1257831	3.0568495	2.960983	3.1550293	3.78	3.4084976	4.1809459
1192075.6	3.2724433	3.1705492	3.3767786	3.28	2.9345999	3.65488
1218538.1	2.9683108	2.8723557	3.0666547	3.73	3.3610268	4.1284199
1233371.1	3.2269282	3.1274457	3.3287699	2.74	2.4251411	3.0843887
1260575.6	2.8367991	2.7445753	2.9313321	3.25	2.9062271	3.623255
1234901.4	3.1978261	3.0988576	3.2991512	2.5	2.1996799	2.8298771
1261903.8	2.8678892	2.7752056	2.9628792	3.0799999	2.745595	3.4439018
1196631.4	3.3368671	3.2341616	3.4420044	3.04	2.7078364	3.4016638
1222622.6	2.9894753	2.8933353	3.0879955	3.47	3.1144609	3.8550041
1237922.4	3.4404418	3.3378835	3.5453506	3.3299999	2.9819038	3.707572
1264636.3	3.2539001	3.1552327	3.3548682	3.8599999	3.4844854	4.2649527
1199129.6	4.1446729	4.0302362	4.2615347	3.8499999	3.4749846	4.2544541
1224784	3.8137336	3.7051404	3.9247019	4.6399999	4.2273607	5.0820341
1240169.8	3.8535047	3.7450168	3.9643378	3.28	2.9345999	3.65488
1266456.1	3.7379897	3.6322587	3.8460174	3.97	3.589036	4.3803954
1241220.5	4.2442098	4.1303649	4.3603973	3.6900001	3.3230624	4.0863872
1267096.5	3.9326129	3.824172	4.0433488	4.52	4.1128569	4.9565439
1121989	4.3244629	4.203629	4.4478889	3.8299999	3.4559851	4.2334552
1145203.6	3.8822789	3.7689915	3.9981067	4.5999999	4.1891847	5.0402122
1243658.5	4.4923906	4.3753567	4.6117625	3.73	3.3610268	4.1284199
1269154	4.018425	3.9088874	4.1302538	4.5700002	4.1605582	5.0088406
1204862.6	3.6344392	3.5275815	3.7437115	2.9200001	2.594651	3.2748606
1229338.3	3.2887611	3.1881595	3.3917298	3.53	3.1713173	3.9181433
1246725	3.8484831	3.7403498	3.9589491	3.2	2.8589563	3.5705299
1271803.1	3.4305625	3.3295155	3.5338967	3.8299999	3.4559851	4.2334552
1167863.9	4.1760006	4.0596123	4.294879	4.4200001	4.0174928	4.851913
1191175	3.7022269	3.5937564	3.8131394	4.6999998	4.2846394	5.1447525
660159.75	1.105793	1.0270213	1.1890035	1.1071428	0.75224984	1.5715009
643360.56	3.1226656	2.9875965	3.2622678	3.1111112	2.481544	3.8517673
473478.34	5.981266	5.7629833	6.2057004	6	5.0407352	7.0886841
552794.75	7.9613638	7.7278695	8.20012	8.0357141	7.0199585	9.1571484
682635.38	1.1880428	1.1076735	1.272702	1.1785715	0.81127435	1.6551524
665131.25	3.0430083	2.9118679	3.1785331	3.0370371	2.4154456	3.7697637
489962.72	5.739212	5.5290265	5.9553428	5.7391305	4.8018827	6.8058763
571837.13	8.3362894	8.101305	8.5763607	8.4285717	7.3874469	9.5753202

683740.94	1.3119004	1.2274414	1.40064	1.3214285	0.93040764	1.8214152
665075.63	3.8146038	3.6675978	3.965991	3.8148148	3.1137755	4.6265726
491192.81	6.9483914	6.7172141	7.1854954	6.9565215	5.9203696	8.1218414
572342.69	9.5327501	9.2814627	9.7891188	9.6785717	8.5604382	10.90218
639299.56	1.2060074	1.1223708	1.2942262	1.2142857	0.84092814	1.6968426
622983.81	3.3821104	3.2392247	3.5296762	3.3703704	2.7136121	4.1380677
459995.66	6.2044063	5.9788465	6.4362979	6.2173915	5.2401423	7.323997
536546.25	8.4615259	8.2171631	8.7113094	8.6071424	7.5546827	9.7652035
678020.56	0.96457249	0.8920537	1.0414163	0.96428573	0.63547039	1.4029851
671929.5	2.8991137	2.7717874	3.0307803	2.8888888	2.2835467	3.605464
489234.75	5.1958694	4.9958277	5.4018669	5.2173915	4.325736	6.2387209
578234.25	6.4022498	6.1976576	6.6118751	6.5357141	5.6230564	7.5542989
656428.5	0.40674651	0.3594167	0.45857605	0.39285713	0.19611287	0.70293
650741.19	1.046499	0.9693661	1.128137	1.037037	0.6891036	1.4988073
474217.41	1.8451452	1.7248974	1.971566	1.826087	1.3160828	2.468339
559510.63	2.595125	2.4633436	2.732125	2.6428571	2.0752113	3.3178651
678671.63	0.37426051	0.3296454	0.42323008	0.35714287	0.17126389	0.6567984
673061.31	0.84539104	0.7773464	0.91779655	0.85185188	0.54000098	1.278196
491082.22	1.5190939	1.412026	1.6321282	1.5217391	1.059947	2.1163707
578432.19	2.3823018	2.2581646	2.511488	2.4285715	1.8858807	3.078793
657083.56	0.43221292	0.3834021	0.48551622	0.42857143	0.22144911	0.748628
651963.25	1.084417	1.005945	1.1673846	1.074074	0.71932429	1.5425495
476264.25	1.8099196	1.6910963	1.9348898	1.826087	1.3160828	2.468339
560987.88	2.8824153	2.7436197	3.0264132	2.9285715	2.3291798	3.6351292
679461.13	0.39295846	0.347233	0.44303107	0.39285713	0.19611287	0.70293
674373.81	1.1699742	1.0897509	1.2545409	1.1851852	0.81066579	1.6731279
493365.97	1.8059616	1.6893111	1.9285448	1.826087	1.3160828	2.468339
581116.06	2.9907968	2.8518255	3.1347888	3.0357144	2.4248207	3.7537076
679955.94	0.43532231	0.3871375	0.48784685	0.42857143	0.22144911	0.748628
674961.19	1.0800622	1.0030715	1.1613942	1.074074	0.71932429	1.5425495
494413.66	1.8628126	1.7444336	1.9871106	1.8695652	1.3530136	2.5182922
582485.38	2.9923499	2.8535037	3.136205	3.0357144	2.4248207	3.7537076
658568.13	0.56941718	0.5132366	0.63006878	0.5714286	0.32662079	0.9279642
653642.5	1.3952581	1.3061622	1.4888314	1.4074074	0.99596524	1.9317766
479501.31	2.4191799	2.2819502	2.5625055	2.4347825	1.8392086	3.1617677
565055.63	3.5854876	3.4310441	3.7450919	3.6428571	2.9703147	4.4221802
681036.69	0.54475772	0.4907288	0.60311067	0.53571427	0.29983521	0.8835793
675909.88	1.5194334	1.427915	1.6152788	1.5185186	1.0897151	2.0600419
496618.97	2.3861351	2.2521946	2.5259607	2.3913043	1.8014576	3.1126113
585001.56	3.5418708	3.3909907	3.6977353	3.6071429	2.9380772	4.3830056
659203.19	0.61892903	0.5603222	0.68199956	0.60714287	0.35368308	0.9720945
654672.44	1.5580311	1.4638714	1.6566584	1.5555556	1.1211076	2.1026592
481599.03	2.4937758	2.3547153	2.6389046	2.4782608	1.8770106	3.2108743
566630.94	3.5261047	3.3731701	3.6841857	3.5714285	2.9058568	4.3438139
681089.06	0.53590643	0.4823318	0.59380531	0.53571427	0.29983521	0.8835793
677291.75	1.3362041	1.2505553	1.4261742	1.3333334	0.93385029	1.8458954
498701.44	2.2418222	2.1123216	2.3771851	2.2608695	1.6885256	2.9648297
585968.5	3.4319251	3.2835519	3.5852747	3.5	2.8414693	4.265379
681109.19	0.57112724	0.5157751	0.63080114	0.5714286	0.32662079	0.9279642
677938.38	1.4883358	1.3979069	1.5830795	1.4814814	1.058392	2.0173573
499764.09	2.4831717	2.3469207	2.6252692	2.4782608	1.8770106	3.2108743
585984.5	3.3482115	3.2016809	3.4997187	3.3928571	2.745024	4.1475921
615085.31	0.62430364	0.5634153	0.68997824	0.60714287	0.35368308	0.9720945
612795.63	1.5584315	1.4611458	1.6604918	1.5555556	1.1211076	2.1026592
452157.28	2.6030765	2.45647	2.7561471	2.6086957	1.9907097	3.3579085
529007.69	3.5859592	3.4263883	3.7510438	3.6428571	2.9703147	4.4221802
680788.75	0.74178666	0.6784924	0.80939597	0.75	0.46426183	1.1464547
679062.31	1.7391629	1.641376	1.8412539	1.7407408	1.2790308	2.3148162
501863.91	2.7995636	2.6550739	2.9498727	2.7826087	2.1429477	3.5533292
586977.69	4.0665941	3.9050765	4.2330766	4.1071429	3.3908677	4.9300032
658891.38	0.61466885	0.5562556	0.67754817	0.60714287	0.35368308	0.9720945
657628.63	1.6179345	1.5221665	1.7181489	1.6296296	1.1840913	2.1877017
486782.28	2.6028063	2.4614437	2.7501712	2.6086957	1.9907097	3.3579085

569436.56	3.7651253	3.6074219	3.9279485	3.8214285	3.131753	4.6178055
680951.19	0.66084033	0.6011855	0.72481287	0.6785714	0.40854433	1.0596733
680184.19	1.5701629	1.4773942	1.6672307	1.5555556	1.1211076	2.1026592
504142.25	2.7393062	2.5967193	2.8876863	2.7391305	2.1048231	3.5045378
590003.38	3.9948924	3.8352263	4.1594982	4.0357141	3.3259997	4.8520417
659233.94	0.69474578	0.6325675	0.76138347	0.6785714	0.40854433	1.0596733
658710.06	1.8278148	1.7260156	1.9340506	1.8148148	1.34261	2.3992815
488913.44	3.3400595	3.1800077	3.5060809	3.347826	2.6420519	4.1842084
572374.13	4.5215182	4.348978	4.6991487	4.5714288	3.8138332	5.4354434
681612.81	0.64699489	0.5880112	0.71029288	0.64285713	0.3809979	1.0159914
681203.75	1.7322277	1.6347901	1.8339559	1.7407408	1.2790308	2.3148162
506353.03	3.2151482	3.0608482	3.3752117	3.2173913	2.5263443	4.0391402
592781	4.5160017	4.3465333	4.6903844	4.6071429	3.8464582	5.4742355
682169.75	0.62154615	0.5637852	0.6836192	0.60714287	0.35368308	0.9720945
681425.44	1.6260033	1.5316594	1.7246376	1.6296296	1.1840913	2.1877017
507569.84	2.941467	2.7941372	3.0945489	2.9565217	2.2958548	3.7480958
593807.19	4.2017007	4.0384326	4.3698754	4.25	3.5207727	5.0857587
660584.06	0.73722637	0.6731954	0.80570555	0.75	0.46426183	1.1464547
659786.25	2.0794613	1.9708711	2.1924782	2.074074	1.5667332	2.6933577
492372.94	3.6171768	3.4511178	3.7891612	3.6086957	2.874306	4.4735212
575589.25	4.7620764	4.585453	4.9437609	4.8214288	4.0424538	5.7067442
682924.94	0.74093062	0.6777705	0.80839223	0.75	0.46426183	1.1464547
682154.75	2.134413	2.0261738	2.2469325	2.1481481	1.631179	2.7769797
510087.06	3.7111311	3.5458176	3.8821628	3.6956522	2.9519558	4.5697312
595450.19	4.8937764	4.7176886	5.0747552	4.9285712	4.140604	5.8228474
661239.75	0.85445559	0.7854435	0.92790675	0.85714287	0.5491876	1.2753606
660453.25	2.4059236	2.2890687	2.5271978	2.4074075	1.8579862	3.0684383
494681.22	4.4715667	4.2871447	4.6618814	4.478261	3.6553016	5.4311938
576583.13	5.5967646	5.4053111	5.7932677	5.6785712	4.8302193	6.6330338
683682.06	0.77082616	0.7064112	0.83953655	0.78571427	0.49240297	1.1895809
682507.5	2.1171927	2.0094244	2.2292397	2.1111112	1.5989349	2.7351892
512308.91	3.9644051	3.7938478	4.1406541	3.9565217	3.1855445	4.8577318
596334.38	5.2017798	5.020319	5.388123	5.25	4.435627	6.1705918
684054.81	0.65053266	0.5914872	0.71387666	0.64285713	0.3809979	1.0159914
682668.31	2.0478466	1.9418932	2.158078	2.0370371	1.5345751	2.6514838
513598.91	3.9271891	3.757654	4.1024017	3.9130435	3.146549	4.8097949
597144.69	5.1562042	4.9756689	5.3416162	5.2142859	4.4028058	6.1319938
617944.44	0.78971499	0.7211939	0.86299115	0.78571427	0.49240297	1.1895809
616777.81	2.1644747	2.0499105	2.2837749	2.1481481	1.631179	2.7769797
464864.5	4.2571545	4.0716381	4.4489441	4.2608695	3.4591799	5.1926351
540060.31	5.2068257	5.0161376	5.4029074	5.2857141	4.4684577	6.2091799
690529.5	0.76318246	0.6994062	0.83121151	0.75	0.46426183	1.1464547
676737.75	2.4160023	2.3003001	2.5360172	2.4074075	1.8579862	3.0684383
519981.44	4.6674743	4.4836097	4.8569431	4.652174	3.8125689	5.621676
595331.81	5.837081	5.6446033	6.0344481	5.9285712	5.0609894	6.9022059
668251.44	0.67938501	0.6183205	0.74484897	0.6785714	0.40854433	1.0596733
655302.56	2.2340825	2.1210949	2.3515258	2.2222223	1.6957897	2.8604405
504213.19	4.2105207	4.0333009	4.3935227	4.2173915	3.4200203	5.1448598
577182.19	5.2600374	5.0745788	5.450542	5.3214288	4.5012984	6.2477584
691305.94	0.74641335	0.6833918	0.81368375	0.75	0.46426183	1.1464547
676813.13	2.3950481	2.2798612	2.5145471	2.4074075	1.8579862	3.0684383
522482.69	4.5800562	4.3983707	4.7673192	4.5652175	3.7338955	5.526474
597515.88	5.6299758	5.4413152	5.8235087	5.7142859	4.8631606	6.6715126
669042.75	0.7802192	0.7147146	0.85011357	0.78571427	0.49240297	1.1895809
655350.19	2.2934303	2.1789367	2.4123788	2.2962964	1.7605572	2.9437482
506689.75	4.4721646	4.289906	4.6601758	4.478261	3.6553016	5.4311938
579531.06	5.568295	5.3778152	5.7637987	5.6428571	4.7972865	6.5945468
692010.38	0.69218618	0.6315786	0.75704044	0.6785714	0.40854433	1.0596733
677223.69	2.3965495	2.2813604	2.516048	2.4074075	1.8579862	3.0684383
524845.75	4.4889379	4.3094883	4.6739402	4.478261	3.6553016	5.4311938
599866.94	5.1644788	4.9842043	5.3496075	5.25	4.435627	6.1705918
692556.06	0.68730897	0.6269435	0.75191802	0.6785714	0.40854433	1.0596733
677342.63	2.2425873	2.1312156	2.358269	2.2222223	1.6957897	2.8604405

526025	4.383822	4.2067041	4.5664806	4.3913045	3.5767896	5.3358331
600881.5	5.4370122	5.2521558	5.6267138	5.5	4.6656442	6.4405098
670883.94	0.81385165	0.7470094	0.8850702	0.8214286	0.52071524	1.2325462
655644.06	2.3808651	2.2642076	2.5019746	2.3703704	1.825474	3.0269101
510185.94	4.578723	4.3949103	4.7682481	4.5652175	3.7338955	5.526474
582540.13	5.5017672	5.3129239	5.6956081	5.5714288	4.7314472	6.5175462
693944.44	0.85885841	0.7912813	0.93066376	0.85714287	0.5491876	1.2753606
677547.13	2.5149543	2.3969474	2.6372676	2.5185184	1.9557282	3.1928225
528287.56	4.9821353	4.7935982	5.1761866	5	4.1280127	6.0017428
602779.5	5.7102141	5.5210266	5.9042306	5.7857141	4.92907	6.7484436
671851.75	1.0136164	0.9389072	1.0926893	1	0.66449273	1.4452784
655926.13	3.149745	3.0153763	3.288559	3.1481481	2.5146289	3.8927341
512088.66	5.7216654	5.5163465	5.9326715	5.7391305	4.8018827	6.8058763
584047.06	6.7871242	6.5774665	7.0017643	6.8928571	5.9546599	7.9369144
695011.63	0.9323585	0.8619443	1.0069922	0.9285714	0.6065737	1.3605723
677448.88	2.7500231	2.6265512	2.8778005	2.7407408	2.152071	3.4407489
530496.63	5.4477253	5.2509007	5.6500401	5.4347825	4.5238652	6.4752979
603668.75	6.8116827	6.6050582	7.0231266	6.8928571	5.9546599	7.9369144
695991.13	0.95978236	0.8883692	1.0354083	0.96428573	0.63547039	1.4029851
676775.63	3.0438449	2.9138072	3.1781909	3.0370371	2.4154456	3.7697637
532137.5	5.9533486	5.7478256	6.1643429	5.9565215	5.0008926	7.0415826
603412.69	7.2172828	7.0045047	7.4348822	7.3214288	6.3534431	8.3952007
628445.94	0.99133426	0.9150084	1.0723279	1	0.66449273	1.4452784
611628.75	3.08357	2.9459596	3.225949	3.074074	2.4484828	3.8107774
481358.94	6.2427425	6.021513	6.4700222	6.2608695	5.2800617	7.3710222
545758.94	6.9334641	6.7142911	7.1579695	7.0357141	6.0874872	8.089776
697001.94	1.0014319	0.928508	1.0785612	1	0.66449273	1.4452784
675993.75	3.2589059	3.1242266	3.3978975	3.2592592	2.6140213	4.0154986
534956.19	6.2098541	6.0004616	6.4246893	6.2173915	5.2401423	7.323997
604860.63	7.3802128	7.1652842	7.5999503	7.5	6.5198607	8.5858974
674676.81	0.75147092	0.6874741	0.81982177	0.75	0.46426183	1.1464547
654332.19	2.721859	2.5969026	2.8512743	2.7037036	2.119272	3.3995016
518718.28	4.9583755	4.7685828	5.1537848	4.9565215	4.0885191	5.9542966
586473.75	6.0735879	5.8757524	6.2763872	6.1785712	5.2921615	7.17098
698239.63	0.79915261	0.7342121	0.86829722	0.78571427	0.49240297	1.1895809
675565.44	2.7843342	2.6599147	2.9130714	2.7777777	2.1848984	3.4819684
537492	5.281939	5.0894136	5.4798837	5.2608695	4.3653302	6.286068
607231.19	6.394599	6.1950331	6.5989575	6.5	5.5899339	7.5159993
653679.5	0.92553002	0.8532397	1.0023086	0.9285714	0.6065737	1.3605723
632075.63	2.893641	2.7625329	3.0293643	2.8888888	2.2835467	3.605464
503771.59	5.9967651	5.784811	6.2145	6	5.0407352	7.0886841
569512.06	6.7285666	6.5171962	6.9450474	6.8571429	5.9214692	7.8986831
203087.2	4.4857578	4.1991596	4.7867665	4.6320753	4.2313666	5.0605035
264694.56	3.8837216	3.6499095	4.128583	4	3.6282756	4.3994751
345964.41	3.1910796	3.0055969	3.3850133	4.1320753	3.7541151	4.5377793
110471.6	5.6123023	5.1791701	6.0719876	5.6415095	5.1983495	6.11235
300708.56	5.5202951	5.2579026	5.7923923	5.7264152	5.2798648	6.2006431
394087.56	3.6540103	3.4676969	3.8477333	3.8018868	3.4397128	4.191823
245800.84	4.0276508	3.7806368	4.2865663	3.9528301	3.5833578	4.3500557
242064.3	4.5525093	4.2876563	4.8294406	4.7452831	4.3395967	5.1786833
222914.34	5.0108933	4.7213078	5.3135939	5.1415095	4.7188544	5.5918627
209967.44	4.8007445	4.5089164	5.1065035	4.9622641	4.5472016	5.4050322
273728.13	4.190289	3.9512684	4.4399886	4.3113208	3.9250548	4.7253213
358421.91	2.9713585	2.7955608	3.1553149	3.7830188	3.4217672	4.1720338
114195.43	5.5256152	5.1028323	5.9740825	5.6132073	5.1711831	6.0829134
310882.19	5.6420088	5.3810301	5.9123721	5.8490567	5.3976531	6.3281341
407529.19	3.6610875	3.4776533	3.8516858	3.8301888	3.4666352	4.2215023
254062.98	4.0974092	3.8522539	4.3540754	4.0283017	3.6552327	4.4291201
250295.11	4.5785952	4.3173127	4.8515568	4.7547169	4.348619	5.188529
230484.05	4.9027252	4.6210012	5.1971331	5.0566039	4.6375284	5.5033808
210105.67	5.5162721	5.2032242	5.8432312	5.6792455	5.2345753	6.1515946
273971.06	4.9676781	4.707232	5.2387848	5.1415095	4.7188544	5.5918627
359568.41	3.6794112	3.483793	3.8831532	4.7358489	4.3305755	5.1688375

114252.66	6.5643988	6.1029434	7.0514979	6.6698112	6.1871614	7.1801119
311142.75	6.0422425	5.7721705	6.3216891	6.2641511	5.7966886	6.759275
407907	4.4985743	4.2950778	4.7092223	4.6981134	4.2944937	5.1294489
254171.22	5.06745	4.7944503	5.3519444	5.0094337	4.59236	5.4542112
250554.48	5.1246338	4.8481297	5.4127984	5.2830191	4.8544631	5.7392678
230678.83	6.1687498	5.8523645	6.4977946	6.3773584	5.9056096	6.8767657
196673.81	4.8760939	4.5723271	5.1947374	5.018868	4.6013932	5.464046
256488.23	4.4485474	4.1941385	4.7143526	4.6132073	4.2133341	5.040801
337237.13	3.2440083	3.0546019	3.4420843	4.1037736	3.7271409	4.5081511
106921.76	6.0324483	5.5758238	6.5165014	6.1320753	5.6696634	6.6221528
291262.88	5.4658527	5.2006254	5.7411013	5.6698112	5.2255182	6.1417837
381893.91	3.9906371	3.7927747	4.1961436	4.1603775	3.7810938	4.5674028
237847.61	4.465044	4.2005062	4.7418761	4.4150944	4.0240984	4.8338194
234576.13	4.7020984	4.4286647	4.9879956	4.8584905	4.447886	5.2968044
215923.91	5.3352127	5.0315356	5.6524277	5.5	5.0625477	5.9651375
210430.8	3.3930395	3.1486919	3.6513147	3.490566	3.1439173	3.8649964
274467.78	3.4248099	3.2093432	3.6509378	3.5377359	3.1886916	3.9145586
361498.88	2.3872826	2.2306442	2.5520194	3.009434	2.6882122	3.358474
114364.97	5.3163133	4.9020772	5.7562027	5.3584905	4.9268198	5.8178515
311613.09	4.6532063	4.4167542	4.8990288	4.7924528	4.3847103	5.2279077
408642.63	3.1739225	3.0035191	3.3514748	3.3018868	2.9649866	3.6665821
254386.27	3.7187543	3.4855256	3.9634852	3.6603773	3.3051782	4.0433474
250999.61	3.9442294	3.7023313	4.1977816	4.0849056	3.7091608	4.4883966
231015.03	4.4932141	4.2239947	4.7750931	4.6226416	4.2223501	5.0506525
203759.64	1.4085224	1.2502631	1.5812675	1.4622642	1.241124	1.7114425
265769.44	1.4712	1.328971	1.6245043	1.518868	1.2933123	1.7724482
350455.41	1.0386486	0.934676	1.1510253	1.2924528	1.0850993	1.5278906
110683.8	1.8069491	1.5651872	2.0754797	1.8207548	1.572929	2.0965436
301623.59	1.7206876	1.5758168	1.8752961	1.7830188	1.5378702	2.0561376
395692.38	1.2080091	1.1021283	1.321317	1.254717	1.0505483	1.4869814
246271.5	1.5430125	1.3917528	1.7062272	1.5283018	1.3020184	1.7826078
242981.67	1.4116291	1.2661744	1.5692114	1.4622642	1.241124	1.7114425
223660.33	1.3994435	1.2486852	1.5633881	1.4433962	1.2237469	1.6910886
210702.83	1.4475363	1.2896242	1.6194489	1.481132	1.2585108	1.731787
274861.28	1.4625559	1.3230616	1.6127568	1.518868	1.2933123	1.7724482
363102.47	0.8620156	0.769153	0.9630006	1.0754716	0.88713151	1.29197
114403.84	1.4859641	1.2709811	1.7268955	1.481132	1.2585108	1.731787
311815.34	1.3758143	1.2486874	1.5123739	1.4339622	1.215062	1.680908
409222.47	0.98479444	0.8909813	1.0857987	1.0283018	0.84434283	1.2404381
254636.61	1.2724015	1.13761	1.4187715	1.254717	1.0505483	1.4869814
251255.16	1.2019653	1.070212	1.3454608	1.245283	1.041918	1.4767468
231247.31	1.2929879	1.1505687	1.4481664	1.3396226	1.128353	1.5789629
204103.69	1.5384338	1.3729589	1.7183578	1.5849056	1.3543016	1.8435196
266322.34	1.5056942	1.3619109	1.6605275	1.5471698	1.3194374	1.8029203
352476.97	1.137663	1.0290241	1.254651	1.4528302	1.2324343	1.7012668
110792.41	1.8142036	1.5720539	2.0830944	1.8301886	1.5816981	2.1066406
302075.28	1.8737051	1.7225019	2.0346253	1.9339622	1.678268	2.2176003
396502.97	1.2938112	1.1842595	1.410771	1.3490566	1.137012	1.5891693
246608.36	1.3989793	1.2552352	1.5546721	1.3867924	1.171675	1.6299676
243418.23	1.5528828	1.4002639	1.7175976	1.6037736	1.3717464	1.8638064
223998.69	1.5669734	1.4073128	1.7397869	1.6226416	1.3891997	1.8840849
211171.31	1.6668931	1.4972864	1.8504511	1.7169812	1.4765863	1.9853584
275579.34	1.6184087	1.4716749	1.7758123	1.6698114	1.4328685	1.934746
365392.81	1.0591341	0.9562276	1.1700971	1.3584906	1.1456738	1.5993727
114607.13	1.9283267	1.6824559	2.2000213	1.9433962	1.6870568	2.2276778
312544.91	1.6317655	1.4932011	1.7797283	1.6886792	1.4503498	1.9549967
410304.44	1.3989612	1.2868416	1.518234	1.4528302	1.2324343	1.7012668
255074.52	1.6152142	1.4629942	1.7789692	1.5754716	1.3455822	1.833373
251865.67	1.5285926	1.3796976	1.6891758	1.5943396	1.3630229	1.853664
231776.89	1.7171686	1.5525898	1.8944453	1.773585	1.5291098	2.0460315
211395.48	1.6651255	1.4956986	1.8484888	1.7075472	1.4678389	1.9752396
275925.06	1.6236292	1.4767433	1.7811712	1.6698114	1.4328685	1.934746
366540.47	0.97124338	0.872961	1.0775626	1.235849	1.0332907	1.4665091

114720.88	2.1792023	1.9174191	2.4667499	2.2169812	1.9425697	2.5192945
312912.28	1.8088137	1.6628469	1.9641607	1.8773584	1.6255687	2.1571014
410821.91	1.3533845	1.2432128	1.4707019	1.4056604	1.1890222	1.6503513
255276.98	1.5590909	1.4096627	1.7200478	1.5377358	1.3107268	1.7927651
252182.17	1.5187434	1.3704319	1.6787289	1.5660378	1.3368651	1.8232242
232040.88	1.6376425	1.4771063	1.810867	1.6981132	1.4590935	1.9651191
204803.11	2.0409846	1.8499906	2.2463431	2.0849056	1.8190702	2.3786616
267367.28	1.9561107	1.7920358	2.13117	2.009434	1.7486215	2.2981782
355811.34	1.3153038	1.1988219	1.4400464	1.7075472	1.4678389	1.9752396
111117.8	2.6548402	2.3604989	2.9757378	2.6886792	2.3855574	3.0196486
303189.78	2.209837	2.0456536	2.3836908	2.2735848	1.9955765	2.5794878
398075.84	1.7408743	1.6136566	1.8754556	1.8018868	1.5553961	2.076344
247232.34	1.9212697	1.7523535	2.1020732	1.8867924	1.6343479	2.1671884
244350.52	1.8907266	1.7222275	2.0712557	1.9433962	1.6870568	2.2276778
224819.42	2.0861187	1.9015669	2.2837443	2.1603773	1.8896092	2.4590552
211844.69	2.0628321	1.8739345	2.2656116	2.1132076	1.845512	2.4088194
276584.78	1.9234608	1.7634653	2.0940735	1.9716982	1.7134324	2.2579014
368809.88	1.3638463	1.2472477	1.4884104	1.726415	1.4853356	1.9954752
114909.17	2.7848082	2.4880142	3.1072628	2.8207548	2.5100558	3.1592884
313611.63	2.1810417	2.0206327	2.3507981	2.2547169	1.9779025	2.5594285
411851.03	1.6947875	1.5713738	1.8253185	1.745283	1.5028398	2.0157032
255644.94	2.1592448	1.9828502	2.3471231	2.1226416	1.8543288	2.4188693
252769.09	1.8950101	1.7290839	2.0725629	1.9528302	1.6958472	2.2377539
232541.86	1.9351355	1.7604486	2.1224661	1.990566	1.7310239	2.2780428
205131.3	2.2180915	2.018939	2.431576	2.2547169	1.9779025	2.5594285
267893.72	2.1463735	1.9744991	2.3292036	2.2075472	1.9337397	2.5092578
357861.66	1.2518804	1.1386257	1.3733513	1.5283018	1.3020184	1.7826078
111236.7	2.7688704	2.4682457	3.0960124	2.8113208	2.501157	3.1493201
303637.84	2.2526836	2.0870056	2.4280164	2.3207548	2.039783	2.6296151
398917.78	1.865046	1.7334216	2.0040154	1.9245284	1.6694808	2.2075212
247499.92	1.9393946	1.7697549	2.1209078	1.9150944	1.6606952	2.1974404
244782.84	1.9650069	1.7933017	2.1487176	2.028302	1.7662249	2.3183076
225143.81	2.0076058	1.8267688	2.2015014	2.0660377	1.8014494	2.3585494
212054.91	1.8014202	1.6252801	1.9914439	1.8490566	1.5992413	2.1268299
277048.94	1.9418952	1.78125	2.1131389	1.990566	1.7310239	2.2780428
370765.53	1.2298878	1.11958	1.3481247	1.5754716	1.3455822	1.833373
114985.02	2.5220678	2.2401183	2.8296845	2.5471699	2.2523761	2.8698258
313952.06	2.2901585	2.125798	2.4638538	2.3773584	2.0928702	2.6897285
412550.75	1.6313144	1.5103786	1.759357	1.6886792	1.4503498	1.9549967
255843.66	1.7823385	1.6224818	1.9536861	1.745283	1.5028398	2.0157032
253099.94	1.7107867	1.5534221	1.8797714	1.7830188	1.5378702	2.0561376
232750.02	1.9419978	1.7670705	2.1295569	1.981132	1.7222275	2.2679729
212136.67	1.880863	1.7008154	2.0747836	1.9150944	1.6606952	2.1974404
277128.91	1.8944253	1.7358208	2.063627	1.9528302	1.6958472	2.2377539
371616.19	1.2163087	1.1067486	1.3337804	1.490566	1.2672077	1.7419558
115021.12	2.2865367	2.0185182	2.5802388	2.2924528	2.0132554	2.5995424
314168.19	2.3044982	2.1396699	2.4786544	2.3679245	2.0840194	2.6797125
412674.16	1.7374482	1.6125841	1.8694141	1.8018868	1.5553961	2.076344
255899.08	2.2274406	2.0483103	2.4180405	2.1792452	1.9072574	2.4791403
253239.39	1.8046166	1.6429348	1.9779073	1.8490566	1.5992413	2.1268299
232912.45	2.1209686	1.9380345	2.3165174	2.1698112	1.8984326	2.4690983
191632.36	2.0821118	1.8827996	2.2967815	2.1415095	1.8719662	2.4389648
250313.17	1.8177229	1.6545178	1.9926732	1.8679246	1.6167914	2.1470125
336275.25	1.3619795	1.2400852	1.4926159	1.735849	1.4940867	2.00559
103894.21	2.8201764	2.5064704	3.1622868	2.8584905	2.5456595	3.1991529
283840.97	2.4556005	2.2766588	2.6448691	2.528302	2.2346358	2.8498323
372778.75	1.7785349	1.6457136	1.9192219	1.8396226	1.5904689	2.1167362
231114.38	2.0509326	1.8704309	2.2441509	2.018868	1.7574224	2.3082438
228741.22	2.102813	1.919066	2.2994072	2.1698112	1.8984326	2.4690983
210455.55	2.3425374	2.1402929	2.558743	2.3867924	2.1017222	2.6997433
212342.06	2.4159133	2.211349	2.6343102	2.471698	2.1814408	2.7898262
277411.56	2.4115794	2.232276	2.6014519	2.471698	2.1814408	2.7898262
373358.25	1.8427341	1.7075938	1.9857261	2.2735848	1.9955765	2.5794878

115107.2	2.7887049	2.4919457	3.1110797	2.8018868	2.4922593	3.1393509
314550.38	2.4829092	2.3118057	2.6633251	2.5471699	2.2523761	2.8698258
413168.94	2.0766325	1.9399871	2.2203634	2.1603773	1.8896092	2.4590552
256050.25	2.124583	1.9497758	2.3108568	2.0754716	1.8102591	2.3686061
253483.98	2.0277414	1.8562086	2.2108619	2.0849056	1.8190702	2.3786616
233220.08	2.5297992	2.3297598	2.7424212	2.5754716	2.2789941	2.8998084
205681.19	2.4892893	2.278311	2.7145488	2.5377359	2.2435055	2.8598297
268754.53	1.9906641	1.8255334	2.1667209	2.0471699	1.7838342	2.3384314
362328.56	1.4130821	1.293317	1.540954	1.773585	1.5291098	2.0460315
111477.65	2.6373	2.3444211	2.95665	2.6603773	2.3589034	2.9897017
304725.97	2.2971458	2.1301033	2.4738073	2.3584905	2.0751698	2.6696954
400305.63	1.9035456	1.7707714	2.0436382	1.9622642	1.704639	2.2478285
247968.11	2.3269122	2.1408973	2.5247626	2.2830188	2.0044155	2.5895157
245570.36	2.0808699	1.9043382	2.2693632	2.1509433	1.880787	2.4490108
225926.8	2.1113033	1.9260602	2.3095546	2.1603773	1.8896092	2.4590552
212749.2	2.7779188	2.5584416	3.0111885	2.8207548	2.5100558	3.1592884
278066.06	1.9779472	1.816076	2.1503763	2.018868	1.7574224	2.3082438
375558.78	1.3606392	1.2452086	1.4838911	1.7169812	1.4765863	1.9853584
115286.02	3.0966461	2.7837167	3.435127	3.1037736	2.7774141	3.4579434
315240.63	2.4108567	2.24248	2.588527	2.490566	2.1991682	2.8098326
414181.63	1.9894654	1.8559285	2.1300721	2.0471699	1.7838342	2.3384314
256437.25	2.1291759	1.9543051	2.3154957	2.0754716	1.8102591	2.3686061
254050.17	2.0310948	1.8596042	2.2141469	2.0943396	1.8278828	2.3887155
233711.17	2.5715501	2.3700404	2.785614	2.6415095	2.3411388	2.9697325
206079.39	2.8095968	2.585376	3.0480587	2.8584905	2.5456595	3.1991529
269426.38	2.5461502	2.3591566	2.7440243	2.6132076	2.3144994	2.9397709
364525.72	1.519783	1.3958473	1.6517721	1.9339622	1.678268	2.2176003
111645	3.4484303	3.1125305	3.8106983	3.490566	3.1439173	3.8649964
305397.34	2.8160036	2.6309185	3.0106747	2.8773584	2.5634668	3.21908
401298.09	2.2701328	2.1250925	2.422466	2.3396227	2.0574739	2.6496575
248339.19	2.327462	2.1415608	2.5251808	2.2735848	1.9955765	2.5794878
246120.28	2.4703369	2.2778533	2.6747406	2.528302	2.2346358	2.8498323
226400.2	3.189043	2.9606383	3.430392	3.2735848	2.9381707	3.6367962
213173.48	2.5190749	2.3104923	2.7414322	2.5849056	2.287869	2.9098005
278750.94	2.4430411	2.2629752	2.6336241	2.509434	2.2168999	2.8298345
377821.84	1.4689463	1.349262	1.5964006	1.8584906	1.6080154	2.1369221
115466.19	3.0918143	2.7793732	3.4297671	3.1226416	2.795264	3.477828
315924.31	2.7759814	2.595274	2.9659543	2.8867924	2.5723715	3.2290421
415213.69	2.3168793	2.172766	2.468039	2.3867924	2.1017222	2.6997433
256799.69	2.2429934	2.0635347	2.4338808	2.1886792	1.9160836	2.4891808
254611.13	2.5568404	2.3641775	2.7610207	2.6226416	2.3233781	2.9497592
234189.3	3.1171365	2.8950858	3.3517001	3.1792452	2.8488317	3.5374632
213331.41	2.7281497	2.5109789	2.9590769	2.7830188	2.4744663	3.1194098
279065.09	2.2396209	2.067456	2.4222968	2.2830188	2.0044155	2.5895157
378927.13	1.2825686	1.1710606	1.4018312	1.6320754	1.3979294	1.8942212
115545.81	3.0723743	2.7610481	3.4091966	3.0471699	2.7238834	3.3982711
316234.03	2.6467741	2.4704785	2.8323283	2.7264152	2.4211097	3.0595644
415668.75	1.9582901	1.8260549	2.0975704	2.028302	1.7662249	2.3183076
256953.06	2.249438	2.0697689	2.4405289	2.2075472	1.9337397	2.5092578
254855.81	2.1659305	1.9889898	2.3543906	2.2264152	1.951401	2.5293298
234391.11	2.9480641	2.7323232	3.1763113	3.018868	2.6971288	3.3684244
206620.03	2.831284	2.6064701	3.0703008	2.8679245	2.5545628	3.2091169
270346.94	2.4967918	2.3119638	2.6924644	2.5471699	2.2523761	2.8698258
367771.5	1.6314478	1.5035013	1.7673723	2.0849056	1.8190702	2.3786616
111902.42	3.4404974	3.1053703	3.8019321	3.4150944	3.0723126	3.7856629
306318.97	3.1960149	2.998924	3.4026563	3.2735848	2.9381707	3.6367962
402692.94	2.344218	2.1970427	2.4986594	2.3962264	2.1105752	2.7097571
248790.63	2.596561	2.4001641	2.8047452	2.5471699	2.2523761	2.8698258
246862.83	2.8882437	2.6801054	3.108254	2.971698	2.6525538	3.3186636
227026.2	3.7616804	3.5135872	4.0226693	3.8584905	3.4935629	4.2511768
213648.89	2.6819704	2.4668405	2.9108377	2.7169812	2.4122202	3.0495868
279617.03	2.5534925	2.3696043	2.7478621	2.6037736	2.3056216	2.9297819
381084.25	1.8184955	1.6856056	1.9590775	2.2924528	2.0132554	2.5995424

115692.02	3.8896375	3.5385156	4.2661734	3.8962264	3.5294743	4.2907348
316791.72	3.3965535	3.1966136	3.6057234	3.481132	3.1349645	3.855082
416517.81	2.4248662	2.277607	2.5791481	2.471698	2.1814408	2.7898262
257199.8	2.6982913	2.5012481	2.9067316	2.6320755	2.332258	2.9597464
255293.17	2.8437893	2.6406636	3.058394	2.8962264	2.5812774	3.2390034
234772.28	3.5481191	3.3112302	3.7974799	3.6320755	3.2782874	4.0136361
206863.7	3.1615019	2.9238131	3.4133666	3.2075472	2.8756256	3.5672708
270783.75	2.980238	2.7781394	3.193152	3.0471699	2.7238834	3.3982711
369719.69	2.1286397	1.9825001	2.2827017	2.7075472	2.4033318	3.0396082
111994.34	3.910912	3.5531814	4.2949009	3.8867924	3.5204957	4.2808461
306740.91	4.3196063	4.0901232	4.5586128	4.4245281	4.0331054	4.8436799
403366.41	2.6700289	2.5129282	2.8343787	2.7452831	2.4388916	3.0795164
248972.53	3.0445125	2.8316054	3.269187	2.9622641	2.6436415	3.3087091
247208.67	3.2361324	3.0157397	3.4683726	3.3301888	2.9918087	3.6963615
227307.33	4.1661658	3.9050126	4.4401913	4.2547169	3.8710549	4.6661162
213851.09	3.4276187	3.1839416	3.6849983	3.471698	3.1260123	3.8451669
279991.25	2.617939	2.4318237	2.8145199	2.6792452	2.3766718	3.0096674
383043.28	1.6525548	1.5263084	1.7864583	2.1037736	1.8366966	2.3987682
115768.38	3.8093305	3.4620504	4.1820116	3.8207548	3.4576604	4.2116098
317154.31	3.843555	3.6307924	4.0655313	3.9245284	3.5564137	4.3203979
417104.66	2.558111	2.4069023	2.7163303	2.6320755	2.332258	2.9597464
257318.27	2.6193244	2.4252846	2.8247578	2.5566037	2.2612476	2.8798211
255608.7	2.9185236	2.7128217	3.1356881	3	2.6792963	3.3485227
234992.89	3.6554298	3.415035	3.9082828	3.7169812	3.3589761	4.1027532
213980.73	3.145143	2.9119809	3.3920066	3.1886792	2.8577623	3.5473998
280214.94	2.790715	2.5985205	2.9933627	2.8301888	2.5189555	3.169256
384180.09	1.6450619	1.5192904	1.7784678	2.0849056	1.8190702	2.3786616
115817.7	3.4278009	3.0988667	3.7821467	3.3962264	3.0544181	3.7658229
317399.28	3.7681246	3.55757	3.9878867	3.8867924	3.5204957	4.2808461
417424.72	2.3285637	2.1844585	2.4796774	2.4056604	2.1194296	2.7197695
257419.78	2.820296	2.6188486	3.0331278	2.7452831	2.4388916	3.0795164
255861.81	2.6420512	2.4466121	2.8489487	2.6981132	2.3944442	3.029629
235167.67	3.7632723	3.5193868	4.0196047	3.8584905	3.4935629	4.2511768
193369.89	3.3459191	3.0930347	3.6139693	3.3962264	3.0544181	3.7658229
253261.75	2.7639389	2.5629523	2.9764991	2.8207548	2.5100558	3.1592884
347877.78	1.7046217	1.570166	1.8475122	2.1320755	1.8631468	2.4289179
104643.6	3.4115798	3.0668252	3.7844849	3.3962264	3.0544181	3.7658229
286860.5	4.0716653	3.8414783	4.3120418	4.1886792	3.8080769	4.5970221
377288.81	2.5948291	2.4348121	2.7626002	2.6603773	2.3589034	2.9897017
232568.44	2.8378744	2.6254697	3.0628872	2.7641509	2.4566772	3.0994649
231253.95	3.2042696	2.9776814	3.4435284	3.2924528	2.9560473	3.6566541
212522.36	3.6184428	3.3671837	3.8834863	3.6886792	3.3320744	4.0730529
214261.3	3.5564053	3.3083425	3.818141	3.6132076	3.2603633	3.9938254
280682.44	2.8751354	2.6801641	3.0805407	2.9433963	2.6258187	3.2887974
386284.69	1.7500046	1.6205524	1.8870466	2.2169812	1.9425697	2.5192945
115943.22	4.0709581	3.7119343	4.4553318	4.0566039	3.6821945	4.4587607
317880.28	4.8854871	4.6455002	5.1346569	5.018868	4.6013932	5.464046
418126.72	2.6738305	2.5193746	2.8352783	2.7641509	2.4566772	3.0994649
257624.44	3.0820057	2.8713319	3.3040483	3	2.6792963	3.3485227
256274.03	3.5313761	3.3050199	3.7691526	3.6320755	3.2782874	4.0136361
235503.39	4.14856	3.8924704	4.4170723	4.2264152	3.8440614	4.6365075
207486.41	3.2387664	2.9984884	3.4931748	3.2830188	2.9471087	3.6467257
271866.69	2.5122607	2.3273592	2.7079456	2.5754716	2.2789941	2.8998084
374835.69	1.7714429	1.6392488	1.9114592	2.2452831	1.9690675	2.5493968
112257.3	4.0710044	3.7062693	4.4619284	4.0754719	3.7001715	4.4785185
307855.72	4.2584882	4.0310636	4.4954023	4.3584905	3.9700677	4.7746453
404982.19	2.2815819	2.1368225	2.4335673	2.3301888	2.0486279	2.639637
249391.91	2.7346518	2.5332375	2.9478209	2.6603773	2.3589034	2.9897017
248207.8	3.3480012	3.1242089	3.5835905	3.4433963	3.0991595	3.815418
228065.69	3.7401505	3.4933352	3.9998028	3.8113208	3.4486864	4.2017164
214577	3.3973818	3.1552048	3.6532154	3.4150944	3.0723126	3.7856629
281215.81	2.613651	2.4280891	2.809633	2.6698112	2.3677871	2.999685
388514.31	1.6781878	1.5518283	1.8120953	2.1509433	1.880787	2.4490108

116072.95	4.2387137	3.8723981	4.630343	4.2169809	3.8350644	4.626637
318376.81	4.6799889	4.4453487	4.9237995	4.7830191	4.3756866	5.2180634
418943.16	2.4800501	2.3315237	2.6355574	2.5471699	2.2523761	2.8698258
257840.8	3.0871763	2.8764098	3.3093021	3	2.6792963	3.3485227
256717.19	3.9226046	3.6840403	4.1725631	4.0471697	3.6732066	4.4488811
235859.56	4.0447798	3.7921526	4.3098121	4.1132073	3.7361319	4.5180278
207813.66	3.4165223	3.1698043	3.6773436	3.4528301	3.1081097	3.825335
272392.06	2.9222584	2.7227511	3.1325183	3.018868	2.6971288	3.3684244
377065.47	1.6548851	1.5275704	1.789979	2.0660377	1.8014494	2.3585494
112382.82	4.075356	3.7106204	4.4662504	4.0471697	3.6732066	4.4488811
308370.25	4.2513828	4.0243378	4.4879012	4.3396225	3.9520612	4.7549171
405843.22	2.4960377	2.3446774	2.6546052	2.5566037	2.2612476	2.8798211
249650.06	2.9481266	2.7389574	3.1690331	2.8679245	2.5545628	3.2091169
248655.5	3.7964172	3.5580697	4.0465322	3.9056604	3.5384536	4.3006229
228440.69	4.0535688	3.7966552	4.3232923	4.1226416	3.7451231	4.5279036
214911	3.1827128	2.9486341	3.4304323	3.2169812	2.8845584	3.5772052
281748.94	2.5519173	2.3687708	2.7454655	2.6132076	2.3144994	2.9397709
390761.22	1.6992475	1.5724411	1.8335575	2.1509433	1.880787	2.4490108
116206.29	4.0875583	3.7281845	4.4722233	4.0849056	3.7091608	4.4883966
318943.59	4.3299193	4.1045375	4.5644579	4.4245281	4.0331054	4.8436799
419797.03	2.8204105	2.6620271	2.9857562	2.8962264	2.5812774	3.2390034
258125.84	2.9171817	2.7125151	3.1331987	2.8301888	2.5189555	3.169256
257203.72	2.98596	2.778487	3.2048228	3.0754716	2.7506452	3.4281108
236249.14	3.9280567	3.6793623	4.1891375	3.990566	3.6192911	4.3895922
215069.56	3.305907	3.0673418	3.5580997	3.3490567	3.0096936	3.7162111
282002.78	2.7907526	2.5991564	2.9927356	2.8396227	2.5278559	3.1792223
391892.81	1.8321337	1.7005548	1.9711907	2.3679245	2.0840194	2.6797125
116283.47	4.1966414	3.8325119	4.5860395	4.1981134	3.8170724	4.606894
319230.03	4.1537447	3.9331548	4.3834848	4.2264152	3.8440614	4.6365075
420195.66	2.6654251	2.5115902	2.8262172	2.7264152	2.4211097	3.0595644
258251.81	2.838315	2.6365328	3.0514438	2.7641509	2.4566772	3.0994649
257443.23	2.9521072	2.745929	3.1696651	3.0377359	2.7149644	3.3883231
236435.8	3.9122672	3.6641777	4.1727338	3.981132	3.6103067	4.3797088
208303.05	4.3926387	4.1125941	4.6867328	4.4716983	4.078146	4.8929768
273173.72	2.9395213	2.7396967	3.1500673	2.990566	2.6703813	3.3385704
380397.13	1.7770902	1.6456344	1.9162531	2.2547169	1.9779025	2.5594285
112611.11	4.2446966	3.8726532	4.6428375	4.2547169	3.8710549	4.6661162
309231.84	3.9258571	3.7080979	4.1530662	3.990566	3.6192911	4.3895922
407068.38	2.6629434	2.5067587	2.8263109	2.7264152	2.4211097	3.0595644
250056.25	3.2832613	3.0624871	3.5157461	3.1981132	2.8666935	3.5573356
249382.47	3.1878746	2.9700985	3.4173949	3.2547169	2.9202971	3.6169355
229030.09	3.7636976	3.5166066	4.023571	3.8113208	3.4486864	4.2017164
215377.72	4.025486	3.7619586	4.3026056	4.0566039	3.6821945	4.4587607
282511.47	2.9025371	2.7072468	3.1081927	2.9528301	2.6347296	3.2987537
394222.25	1.9506763	1.8152243	2.0935593	2.4245284	2.1371415	2.7397912
116435.55	4.9125891	4.5181966	5.3321891	4.8679247	4.456913	5.3066454
319805.56	3.9961782	3.7800622	4.2214298	4.0660377	3.6911826	4.4686399
420989.06	2.8480549	2.6891084	3.0139437	2.9150944	2.5990913	3.2589235
258474.36	3.8379049	3.6027615	4.0843658	3.7452831	3.3858831	4.1324482
257910.69	3.5826356	3.3553281	3.8212895	3.6792452	3.3231084	4.0631514
236831.95	4.0239501	3.7724943	4.2877607	4.0943398	3.7181506	4.4982738
208536.42	4.9056177	4.6095748	5.2156863	4.9150944	4.5020523	5.3558435
273575.44	3.3811514	3.1667418	3.6062582	3.4433963	3.0991595	3.815418
382482.66	2.0602241	1.9188697	2.2092366	2.6226416	2.3233781	2.9497592
112710.2	5.4475994	5.0251708	5.8960562	5.4245281	4.99015	5.8865943
309682.78	4.9857469	4.7401314	5.2407889	5.0943398	4.6736698	5.5427098
407683.91	3.5468655	3.3663862	3.7345073	3.6226416	3.269325	4.0037308
250187.61	4.4846344	4.2260318	4.7549224	4.3773584	3.9880762	4.7943721
249749.08	4.1441593	3.8955004	4.4045286	4.2452831	3.8620565	4.6562471
229305.53	4.988977	4.7040305	5.2866712	5.0471697	4.6284938	5.4935474
215572.61	4.3280082	4.0547152	4.6148753	4.3584905	3.9700677	4.7746453
282840.59	3.4047446	3.1930726	3.626761	3.4528301	3.1081097	3.825335
396255.75	2.0718942	1.9325752	2.2186034	2.6226416	2.3233781	2.9497592

116497.64	5.3734994	4.9607496	5.8114276	5.3301888	4.8996835	5.7883854
320178	4.6848941	4.4507818	4.9281244	4.7830191	4.3756866	5.2180634
421501.19	3.3451862	3.1728382	3.5244627	3.4245284	3.0812609	3.7955821
258528.7	3.8525703	3.6169953	4.0994601	3.7358491	3.3769135	4.1225505
258214.64	3.9889295	3.7490132	4.2401724	4.0943398	3.7181506	4.4982738
237036.66	5.205946	4.9194994	5.5047197	5.3301888	4.8996835	5.7883854
215621.77	5.1247144	4.8269711	5.436017	5.1981134	4.7730885	5.6508346
282943.38	4.0502806	3.8191466	4.2917452	4.1320753	3.7541151	4.5377793
397359.84	2.0585876	1.919914	2.2046306	2.5566037	2.2612476	2.8798211
116487.25	6.2925344	5.8451838	6.7650399	6.2075472	5.7422428	6.7005148
320296.41	4.7924361	4.5556612	5.0383248	4.8962264	4.4839954	5.3361654
421677.03	3.5856826	3.4072034	3.7710853	3.6792452	3.3231084	4.0631514
258488.11	4.3947864	4.1429076	4.6579738	4.2452831	3.8620565	4.6562471
258334.53	3.8709497	3.6347175	4.1185055	3.9528301	3.5833578	4.3500557
237108.72	5.339323	5.0492225	5.6417456	5.4245281	4.99015	5.8865943
194823.56	5.2663035	4.9489517	5.5986676	5.3301888	4.8996835	5.7883854
255687.58	3.6998277	3.4677861	3.9433131	3.7735848	3.4127953	4.162138
359751.5	2.1014507	1.9543024	2.2567427	2.6415095	2.3411388	2.9697325
105216.55	7.2231979	6.7187223	7.7555175	7.1226416	6.6235743	7.6493483
289446.34	4.5327916	4.2908087	4.7848673	4.6509433	4.2494011	5.0802045
381073.47	3.3300664	3.1493456	3.5184543	3.4056604	3.063365	3.7757432
233492.52	4.1928539	3.9342902	4.4639468	4.0754719	3.7001715	4.4785185
233458.36	3.9878633	3.7357829	4.2524781	4.0660377	3.6911826	4.4686399
214242.72	6.1565685	5.8287621	6.4980102	6.2264152	5.7603903	6.7201028
215837.7	5.1751847	4.8761044	5.4878101	5.2075472	4.7821283	5.6606617
283334.91	4.0340953	3.8035877	4.27492	4.0943398	3.7181506	4.4982738
399425.88	2.2757664	2.130209	2.4286511	2.8773584	2.5634668	3.21908
116533.86	6.3243423	5.8759298	6.7978992	6.2830191	5.8148394	6.7788596
320719.47	4.9576035	4.7168894	5.2074184	5.0660377	4.6465631	5.5132136
422297.31	3.6348798	3.4552953	3.8213773	3.7264152	3.3679445	4.1126523
258610.42	4.4932451	4.2385788	4.7592139	4.3679247	3.9790716	4.7845087
258694.97	4.4299278	4.1771288	4.694026	4.5377359	4.1412215	4.9619737
237358	5.6791849	5.380023	5.990653	5.7358489	5.2889237	6.2104521
209002.45	3.8277063	3.5670252	4.1024008	3.8490567	3.4845865	4.2412858
274436.38	2.966079	2.7657921	3.177037	3.018868	2.6971288	3.3684244
387592.19	1.9298635	1.7940226	2.0732636	2.5	2.2080336	2.819834
112814.64	5.6198378	5.1908445	6.0748301	5.5471697	5.1078067	6.0142164
310612.47	4.1659627	3.9420428	4.3992882	4.2547169	3.8710549	4.6661162
408999.22	2.924211	2.7608125	3.0947549	2.990566	2.6703813	3.3385704
250350.45	3.9225013	3.6809735	4.1757145	3.8113208	3.4486864	4.2017164
250544.45	3.6121335	3.3806007	3.8553476	3.6981132	3.3410411	4.0829535
229848.77	4.5638704	4.2918334	4.8486309	4.6320753	4.2313666	5.0605035
216166.48	3.9552848	3.6945729	4.2295399	4.0094337	3.6372607	4.4093571
283888.31	3.1596932	2.9562752	3.3734214	3.2075472	2.8756256	3.5672708
401727.88	1.8744031	1.7428969	2.0132024	2.3773584	2.0928702	2.6897285
116637.46	5.5213823	5.1031246	5.9647832	5.4622641	5.0263462	5.9258685
321299.59	4.217248	3.9956629	4.4479232	4.3018866	3.9160535	4.7154546
423084.06	3.3610342	3.1885922	3.5403783	3.4245284	3.0812609	3.7955821
258840.11	3.9715638	3.7324634	4.2219634	3.8490567	3.4845865	4.2412858
259158.13	3.8895173	3.6530807	4.1372404	4	3.6282756	4.3994751
237726.22	5.0436168	4.7621388	5.337389	5.1226416	4.7007794	5.5722027
202407.91	4.204381	3.9266109	4.4966159	4.2547169	3.8710549	4.6661162
265847.59	4.4875336	4.2364697	4.7495909	4.5566039	4.1592469	4.9816833
376904.31	2.032346	1.8909519	2.1815128	2.5377359	2.2435055	2.8598297
109185.84	5.8157725	5.3721652	6.2862425	5.7452831	5.2979827	6.2202606
300892.22	4.58636	4.3475447	4.8348818	4.6509433	4.2494011	5.0802045
396213.56	3.5965452	3.4122119	3.7882488	3.6792452	3.3231084	4.0631514
242288.31	4.0034947	3.7554839	4.2635808	3.8773584	3.5115175	4.270957
242705.33	3.7947252	3.5535758	4.0479321	3.8962264	3.5294743	4.2907348
222593.7	5.1483936	4.8545947	5.4553246	5.2547169	4.8273349	5.7097931
2419073.8	0.76806259	0.7335319	0.80379903	0.82075471	0.65739089	1.0123979
2502062.8	0.65226179	0.6209967	0.6846934	0.6981132	0.54816902	0.8764172
2505021.5	0.8331266	0.7977626	0.86965448	0.88679248	0.7166186	1.0852094

2345811.5	0.79716551	0.7614403	0.83413416	0.8490566	0.68274176	1.0436348
2510648.3	0.66596347	0.6344211	0.69866818	0.70754719	0.55653071	0.8869165
2430775.5	0.43648621	0.410614	0.46356145	0.46226415	0.34198555	0.6111378
2513681.3	0.39026427	0.3662217	0.41547063	0.41509435	0.30160818	0.5572448
2435493.8	0.51447475	0.4863793	0.5437699	0.5471698	0.41548899	0.7073439
2520283.3	0.55192208	0.5232954	0.58170754	0.58490568	0.44844383	0.7498227
2523582	0.50761181	0.4801915	0.53618979	0.53773582	0.40727589	0.6966991
2445379.8	0.5921371	0.5620272	0.62344104	0.6226415	0.4815509	0.7921526
2529939	0.61345351	0.5833097	0.64475119	0.6509434	0.50647283	0.8238105
2450280	0.62401032	0.5931209	0.65609121	0.66037738	0.51479656	0.8343471
2533852	0.54857188	0.5201089	0.57818747	0.58490568	0.44844383	0.7498227
2535142.3	0.56643766	0.5375161	0.59651107	0.59433961	0.45670688	0.7604185
2290287	0.5767836	0.5460957	0.60874701	0.61320752	0.47326064	0.7815834
2538450	0.6760031	0.6443934	0.70876217	0.71698111	0.56489956	0.8974088
2459474.5	0.65461141	0.6230226	0.68738687	0.68867922	0.53981459	0.8659108
2544804.3	0.64759403	0.6167018	0.67963296	0.67924529	0.53146762	0.8553971
2465770.8	0.71498942	0.6820002	0.7491619	0.75471699	0.59844363	0.9393106
2551282	0.65849245	0.6273774	0.69075131	0.68867922	0.53981459	0.8659108
2554187.3	0.62289876	0.5926635	0.65427667	0.66037738	0.51479656	0.8343471
2474536.5	0.70033318	0.6677449	0.73410052	0.74528301	0.5900476	0.928845
2559611	0.66220999	0.6310564	0.69450366	0.6981132	0.54816902	0.8764172
2478999.5	0.75393319	0.7201365	0.78890651	0.79245281	0.6320914	0.9811105
2563647.5	0.69588351	0.6639631	0.72894192	0.73584908	0.58165812	0.9183729
2566122.8	0.70417517	0.6720783	0.73740894	0.74528301	0.5900476	0.928845
2319641	0.76391131	0.7287534	0.80032694	0.8018868	0.64051867	0.9915454
2571124.8	0.79459387	0.7605082	0.82981366	0.83962262	0.67428595	1.0332279
2490602.5	0.66931593	0.6375679	0.70223576	0.70754719	0.55653071	0.8869165
2576611.3	0.78902084	0.755092	0.82408142	0.83018869	0.66583562	1.0228157
2496246.5	0.716676	0.6838473	0.75067347	0.75471699	0.59844363	0.9393106
2582451.5	0.64705956	0.6164035	0.67884564	0.67924529	0.53146762	0.8553971
2585306.8	0.68966669	0.6580227	0.72243917	0.7264151	0.57327545	0.9078943
2504916.3	0.71738923	0.6846002	0.75134289	0.75471699	0.59844363	0.9393106
2591126	0.67576796	0.644483	0.708179	0.70754719	0.55653071	0.8869165
2509667.3	0.79572302	0.7612023	0.8314057	0.83962262	0.67428595	1.0332279
2595286.8	0.621126	0.5911714	0.65220511	0.6509434	0.50647283	0.8238105
2596879	0.74666554	0.7137975	0.78065658	0.78301889	0.62367016	0.9706697
2347284.3	0.76386148	0.7289099	0.80005586	0.8018868	0.64051867	0.9915454
2601637	0.77950919	0.7459485	0.81419075	0.82075471	0.65739089	1.0123979
2520331.5	0.62610811	0.5955938	0.65778059	0.66037738	0.51479656	0.8343471
2607660.3	0.72517115	0.6928513	0.75860953	0.76415092	0.60684615	0.9497699
2442552.8	0.71277887	0.6796869	0.74706537	0.74528301	0.5900476	0.928845
1193918.3	0.87778205	0.8254358	0.93257779	0.98113209	0.73275638	1.2866242
1225155.4	0.66114062	0.6163884	0.70828325	0.66037738	0.45997703	0.918425
1234997	0.77490067	0.7265769	0.82559353	0.86792451	0.63542986	1.1576897
1267065.9	0.53272682	0.4932911	0.57447642	0.52830189	0.35105276	0.7635433
1236661.1	0.99946541	0.9445158	1.0567778	1.1132076	0.84742516	1.4359566
1268360.3	0.67094505	0.6266178	0.71758056	0.66037738	0.45997703	0.918425
1158176.5	0.9566763	0.9011681	1.0147089	1.0566038	0.79814714	1.3720878
1187635	0.64161128	0.5968583	0.68883103	0.64150941	0.44426394	0.8964452
1239667.5	0.79295456	0.7441529	0.84411609	0.86792451	0.63542986	1.1576897
1270980.6	0.54210109	0.5023734	0.58413523	0.52830189	0.35105276	0.7635433
1200236.9	0.50156766	0.4622962	0.54328364	0.5471698	0.36644822	0.7858271
1230538.5	0.37300742	0.3396596	0.40874425	0.3773585	0.23050037	0.5827996
1241340.9	0.47207016	0.4346175	0.51188689	0.52830189	0.35105276	0.7635433
1272340.3	0.31045154	0.280587	0.34262937	0.3018868	0.17255439	0.4902452
1202878.6	0.63597441	0.5917001	0.68268418	0.6981132	0.49153611	0.9622571
1232615.1	0.39590621	0.3615547	0.43264163	0.39622641	0.2452704	0.6056742
1244893.8	0.66431373	0.6198033	0.7111764	0.73584908	0.52326059	1.0059298
1275389.5	0.44221786	0.4064701	0.48026711	0.43396226	0.27509484	0.6511565
1246628.8	0.61285287	0.5701608	0.65789497	0.67924529	0.47573504	0.9403618
1276953.4	0.40486991	0.370718	0.44132191	0.39622641	0.2452704	0.6056742
1208089.9	0.69117373	0.6450821	0.73968905	0.75471699	0.53918087	1.0277103
1237289.8	0.49543771	0.4569888	0.53625762	0.49056605	0.32045403	0.7187929

1249963.3	0.74242181	0.6954173	0.79176742	0.81132078	0.58715683	1.0928438
1279975.9	0.48750919	0.4500038	0.52730626	0.47169811	0.30525815	0.6963195
1210646.6	0.76240247	0.7140046	0.81321764	0.83018869	0.60321635	1.1144896
1239633.3	0.48885426	0.4507022	0.52937305	0.49056605	0.32045403	0.7187929
1251995.4	0.68051368	0.6355802	0.72778559	0.75471699	0.53918087	1.0277103
1281856.6	0.41970372	0.3849833	0.45671478	0.41509435	0.26013741	0.6284578
1252729.4	0.68809754	0.6429231	0.73560888	0.75471699	0.53918087	1.0277103
1282412.9	0.44759375	0.4117214	0.48575476	0.43396226	0.27509484	0.6511565
1131807.9	0.73068941	0.6817316	0.78223437	0.79245281	0.57113028	1.071166
1158479.3	0.42642111	0.3896422	0.46573621	0.41509435	0.26013741	0.6284578
1254532	0.8465308	0.7963769	0.89901561	0.9245283	0.68397111	1.2222755
1283918.1	0.50937825	0.4710821	0.54995847	0.50943398	0.33572018	0.7411997
1215612.5	0.80617797	0.7564878	0.8582747	0.88679248	0.65158176	1.1792459
1243862.1	0.50648701	0.4677039	0.54762816	0.49056605	0.32045403	0.7187929
1257910.9	0.77747959	0.7295101	0.82777476	0.8490566	0.6193077	1.1361046
1286893.3	0.5206337	0.4819524	0.56159335	0.50943398	0.33572018	0.7411997
1218940.9	0.82858819	0.778269	0.88130701	0.90566039	0.66776246	1.2007743
1246829.9	0.60393161	0.5615604	0.64865273	0.60377359	0.41298068	0.8523482
1261322	0.79678303	0.748277	0.84760815	0.86792451	0.63542986	1.1576897
1289960	0.52327204	0.4845362	0.56428069	0.50943398	0.33572018	0.7411997
1262836.1	0.75544244	0.7082593	0.80494249	0.83018869	0.60321635	1.1144896
1291350.9	0.4932819	0.455714	0.53312105	0.49056605	0.32045403	0.7187929
1223524.4	0.87125361	0.8197305	0.92516667	0.96226418	0.71646887	1.2651997
1251012.1	0.53316832	0.4934684	0.57521188	0.52830189	0.35105276	0.7635433
1265637.9	0.79880667	0.7503197	0.84960473	0.86792451	0.63542986	1.1576897
1293973	0.52860457	0.4897273	0.56974733	0.52830189	0.35105276	0.7635433
1225818.1	0.91367549	0.8609428	0.96879315	1	0.74906862	1.3080246
1253181.6	0.59767872	0.5556364	0.64205903	0.58490568	0.39741501	0.8302269
1267774.9	0.84242088	0.7926488	0.89449948	0.9245283	0.68397111	1.2222755
1295872.9	0.55252331	0.5127883	0.59451997	0.5471698	0.36644822	0.7858271
1269132.4	0.86200625	0.8116767	0.91463953	0.94339621	0.70020688	1.2437503
1296990.5	0.54973418	0.5101182	0.5916099	0.5471698	0.36644822	0.7858271
1147295.6	0.91345245	0.858979	0.9704749	1	0.74906862	1.3080246
1172345.1	0.61756557	0.5733945	0.66423643	0.60377359	0.41298068	0.8523482
1271779.1	1.0017463	0.9474874	1.0583025	1.0943396	0.83097798	1.4146878
1299345.6	0.59183639	0.5507401	0.63518715	0.58490568	0.39741501	0.8302269
1232053.9	0.82058102	0.7707723	0.87276375	0.90566039	0.66776246	1.2007743
1258548.6	0.52123535	0.4821063	0.56269431	0.50943398	0.33572018	0.7411997
1274715.9	0.94766217	0.8949688	1.0026481	1.0377358	0.78176469	1.3507558
1301895.5	0.63369143	0.5911822	0.67844993	0.6226415	0.42859778	0.8744202
1235052.4	0.90117633	0.8490042	0.95571572	0.98113209	0.73275638	1.2866242
1261194.1	0.53599995	0.4963507	0.57797384	0.52830189	0.35105276	0.7635433
1277774.9	0.78574091	0.7378836	0.83588737	0.86792451	0.63542986	1.1576897
1304676.8	0.51123774	0.4731709	0.551552	0.50943398	0.33572018	0.7411997
1279293.6	0.87313807	0.8226784	0.92588305	0.96226418	0.71646887	1.2651997
1306013.1	0.50994891	0.4719501	0.55019283	0.50943398	0.33572018	0.7411997
1239588.5	0.85834938	0.8075424	0.91151536	0.94339621	0.70020688	1.2437503
1265327.8	0.57929653	0.5381131	0.62279588	0.56603771	0.38190329	0.8080541
1282314	0.80635476	0.7579486	0.85704172	0.88679248	0.65158176	1.1792459
1308812	0.54782504	0.5084549	0.58943444	0.5471698	0.36644822	0.7858271
1242095.3	0.935516	0.8824933	0.99089205	1.018868	0.76540494	1.3294016
1267572	0.65873969	0.614811	0.70497841	0.64150941	0.44426394	0.8964452
1284583.3	0.74732411	0.7007918	0.796134	0.81132078	0.58715683	1.0928438
1310703.5	0.49744278	0.4599877	0.53713518	0.49056605	0.32045403	0.7187929
1285575.9	0.89531863	0.8443357	0.9485752	0.98113209	0.73275638	1.2866242
1311303	0.60092902	0.5596985	0.64439315	0.58490568	0.39741501	0.8302269
1162105.6	0.91643995	0.8622196	0.9731766	1	0.74906862	1.3080246
1185178.5	0.61425346	0.5704379	0.66054159	0.60377359	0.41298068	0.8523482
1288148.9	0.97737151	0.9241228	1.0328884	1.0754716	0.81455177	1.3933983
1313488.1	0.585464	0.5448102	0.62834799	0.58490568	0.39741501	0.8302269
1247988.1	0.76843679	0.7205654	0.81865263	0.83018869	0.60321635	1.1144896
1272343.3	0.48650393	0.4489281	0.52638537	0.47169811	0.30525815	0.6963195
1291334.5	0.91300899	0.8616309	0.96665043	1	0.74906862	1.3080246

1316325.8	0.54089957	0.5018933	0.58213228	0.52830189	0.35105276	0.7635433
1209655.5	0.8556155	0.8042766	0.90937215	0.94339621	0.70020688	1.2437503
1232897.4	0.57263488	0.5311683	0.61647874	0.56603771	0.38190329	0.8080541
663262.13	0.14323145	0.1158828	0.17509302	0.14285715	0.03892376	0.365771
656161.81	0.31242293	0.2711167	0.35824338	0.2962963	0.1279197	0.5838218
498156.69	0.73069382	0.6575486	0.80975133	0.73913044	0.43057072	1.1834195
601493.06	1.9850603	1.874048	2.1009314	2.0357144	1.5418301	2.6375039
685849.44	0.13268219	0.1068274	0.16290432	0.14285715	0.03892376	0.365771
678400.63	0.2373229	0.2020799	0.27694479	0.22222222	0.08155164	0.4836842
515524.72	0.60132909	0.5362458	0.67213368	0.60869563	0.33277959	1.0212879
622288.13	1.7194607	1.6179647	1.8256557	1.7857143	1.3253915	2.3542416
686958.06	0.16886038	0.1395326	0.20253195	0.17857143	0.05798166	0.4167261
678375	0.35231251	0.3090587	0.39992541	0.37037036	0.17760698	0.6811243
516809.69	0.83783257	0.7607656	0.92059034	0.82608694	0.49735832	1.2900372
622878.69	2.0854783	1.973597	2.2020497	2.1428571	1.6352258	2.7582819
642315	0.12922008	0.1029231	0.16018774	0.14285715	0.03892376	0.365771
635473.69	0.2895478	0.2492207	0.33454186	0.2962963	0.1279197	0.5838218
484028.28	0.824332	0.745422	0.9093222	0.82608694	0.49735832	1.2900372
583994.56	2.0616629	1.9468398	2.1814904	2.1428571	1.6352258	2.7582819
681182.63	0.13065512	0.1049268	0.16078237	0.14285715	0.03892376	0.365771
685374.44	0.29035223	0.2514105	0.33361727	0.2962963	0.1279197	0.5838218
514702.31	0.70914775	0.6382542	0.7857635	0.69565219	0.39762533	1.1296955
629388.81	1.619031	1.5211375	1.7215717	1.6785715	1.2333511	2.2321441
659462.44	0.09401597	0.0720815	0.12052423	0.10714286	0.02209543	0.3131169
663704.06	0.19436373	0.1622724	0.23094419	0.18518518	0.06012913	0.4321604
498781.13	0.43907034	0.3828405	0.50123435	0.43478259	0.20849515	0.7995807
608827.81	1.0692679	0.9886965	1.1546558	1.1071428	0.75224984	1.5715009
681764.13	0.09387411	0.0722945	0.11987514	0.10714286	0.02209543	0.3131169
686360.63	0.14278208	0.1159174	0.17400563	0.14814815	0.04036538	0.3793181
516350.06	0.3699041	0.3193027	0.42624661	0.39130434	0.17892927	0.7428175
629206.31	0.99808282	0.9215377	1.0792896	1.0357143	0.69363409	1.4874585
660039.81	0.11817469	0.0934122	0.14748736	0.10714286	0.02209543	0.3131169
664753.88	0.20308268	0.1702716	0.24037293	0.18518518	0.06012913	0.4321604
500613.34	0.49738985	0.4375234	0.56316072	0.47826087	0.23874611	0.8557408
610086.75	1.2965369	1.2077458	1.390129	1.3571428	0.96039504	1.8627846
682486.44	0.10110091	0.0786625	0.12794967	0.10714286	0.02209543	0.3131169
687495	0.23127441	0.1967231	0.27014735	0.22222222	0.08155164	0.4836842
518448.66	0.5786494	0.5150158	0.64797378	0.56521738	0.30095446	0.966539
631853.13	1.3658237	1.276207	1.4600737	1.4285715	1.0205923	1.9453088
682942.13	0.08785518	0.0670428	0.11308703	0.07142858	0.00865033	0.2580246
688016.06	0.26307526	0.2261443	0.30431762	0.25925925	0.10423567	0.5341732
519409.06	0.43703511	0.3820273	0.49773926	0.43478259	0.20849515	0.7995807
633214.81	1.2839245	1.1971741	1.3752997	1.3214285	0.93040764	1.8214152
661431.13	0.11490236	0.09053	0.14381744	0.10714286	0.02209543	0.3131169
666209.19	0.26568231	0.2279825	0.30783585	0.25925925	0.10423567	0.5341732
503603	0.60563576	0.5395668	0.67756242	0.60869563	0.33277959	1.0212879
614136.44	1.4491894	1.3555318	1.5476129	1.5	1.081068	2.0275643
683968.19	0.12427478	0.0992663	0.1536677	0.10714286	0.02209543	0.3131169
688842.19	0.27292174	0.2353016	0.31484619	0.25925925	0.10423567	0.5341732
521461.44	0.51010484	0.45064	0.57523423	0.52173913	0.26959023	0.9113733
635667.13	1.5936013	1.4969649	1.6948391	1.6428572	1.2027779	2.1913412
662015.06	0.12084317	0.0958211	0.15039979	0.10714286	0.02209543	0.3131169
667148.94	0.25031891	0.2137929	0.29129559	0.25925925	0.10423567	0.5341732
505567.5	0.60328245	0.5374702	0.67492962	0.60869563	0.33277959	1.0212879
615548.44	1.5872024	1.4892248	1.6899328	1.6428572	1.2027779	2.1913412
683969.31	0.10234378	0.079782	0.1293052	0.10714286	0.02209543	0.3131169
690130.25	0.23618729	0.20132	0.27535832	0.22222222	0.08155164	0.4836842
523374.84	0.5369001	0.4759537	0.60348731	0.52173913	0.26959023	0.9113733
636377.63	1.3765411	1.2868825	1.4707997	1.4285715	1.0205923	1.9453088
683962.06	0.12135176	0.096656	0.15043376	0.10714286	0.02209543	0.3131169
690700.25	0.28811342	0.2494719	0.33104482	0.2962963	0.1279197	0.5838218
524331.06	0.57406479	0.5110373	0.64271909	0.56521738	0.30095446	0.966539
636148.81	1.3408812	1.2523955	1.4339691	1.3928572	0.99045759	1.9040816

617637.56	0.12790672	0.101265	0.15940994	0.14285715	0.03892376	0.365771
624283.44	0.27231219	0.2329152	0.31646439	0.25925925	0.10423567	0.5341732
474255.94	0.6093756	0.5411374	0.68383712	0.60869563	0.33277959	1.0212879
574110.06	1.36385	1.2699816	1.4628204	1.4285715	1.0205923	1.9453088
683585.75	0.13165869	0.1058691	0.16183087	0.14285715	0.03892376	0.365771
691717.19	0.34840828	0.3058058	0.3952854	0.33333334	0.15242122	0.6327705
526259.06	0.68407375	0.6152261	0.75851852	0.69565219	0.39762533	1.1296955
636888.19	1.6093876	1.5123581	1.7110096	1.6785715	1.2333511	2.2321441
661577	0.14813091	0.1202598	0.18052413	0.14285715	0.03892376	0.365771
669823.38	0.2896286	0.2503048	0.3333773	0.2962963	0.1279197	0.5838218
510330.09	0.62704515	0.5602171	0.69965106	0.60869563	0.33277959	1.0212879
617744.19	1.6155555	1.5168658	1.7189804	1.6785715	1.2333511	2.2321441
683701.56	0.133099	0.107163	0.16341609	0.14285715	0.03892376	0.365771
692737.25	0.27571782	0.2380008	0.31771418	0.25925925	0.10423567	0.5341732
528407.81	0.63398004	0.5679006	0.7056374	0.65217394	0.36501679	1.0756617
639957.63	1.6110442	1.5141934	1.712465	1.6785715	1.2333511	2.2321441
661878.94	0.13597652	0.1093412	0.16713823	0.14285715	0.03892376	0.365771
670803.25	0.31305751	0.2721455	0.35838395	0.2962963	0.1279197	0.5838218
512341.41	0.68118638	0.6115863	0.7565372	0.69565219	0.39762533	1.1296955
620747.19	1.7946115	1.6907612	1.9031714	1.8571428	1.3870031	2.4353957
684321.56	0.12274931	0.0979097	0.151972	0.10714286	0.02209543	0.3131169
693652.88	0.27679551	0.2390261	0.31883833	0.25925925	0.10423567	0.5341732
530521.88	0.66915244	0.6013466	0.74251109	0.65217394	0.36501679	1.0756617
642785.69	1.6319592	1.5346835	1.7337846	1.6785715	1.2333511	2.2321441
684863.25	0.12265223	0.0978322	0.1518518	0.10714286	0.02209543	0.3131169
693823.25	0.26952109	0.2322742	0.31104165	0.25925925	0.10423567	0.5341732
531708.13	0.64509076	0.5786204	0.71710324	0.65217394	0.36501679	1.0756617
643792.5	1.5175697	1.4238905	1.6157931	1.5714285	1.1418024	2.1095695
663175.19	0.14325023	0.115898	0.17511599	0.14285715	0.03892376	0.365771
671745.13	0.26051548	0.2233464	0.30210221	0.25925925	0.10423567	0.5341732
515699.47	0.68062896	0.611279	0.75569206	0.69565219	0.39762533	1.1296955
623916.75	1.782289	1.6790606	1.8902035	1.8571428	1.3870031	2.4353957
685590.31	0.11668776	0.0925261	0.14522803	0.10714286	0.02209543	0.3131169
694502.31	0.27357721	0.2360583	0.31536484	0.25925925	0.10423567	0.5341732
534177.81	0.64959645	0.5830383	0.72167057	0.65217394	0.36501679	1.0756617
645340.5	1.670436	1.5721948	1.7732081	1.7142857	1.263979	2.2728941
663813.13	0.14763191	0.1198547	0.17991601	0.14285715	0.03892376	0.365771
672397.69	0.30339187	0.263185	0.34800422	0.2962963	0.1279197	0.5838218
517989.38	0.73553634	0.6635248	0.81323159	0.73913044	0.43057072	1.1834195
624799.44	1.8982091	1.7917016	2.0093944	1.9642857	1.4797689	2.556788
686333.13	0.13550271	0.1093682	0.16599998	0.14285715	0.03892376	0.365771
694828.81	0.30079353	0.2613936	0.34445542	0.2962963	0.1279197	0.5838218
536390.5	0.6674242	0.6000699	0.7402702	0.65217394	0.36501679	1.0756617
646095.19	1.7396817	1.6394523	1.8444359	1.7857143	1.3253915	2.3542416
686688.56	0.1223262	0.0975722	0.15144816	0.10714286	0.02209543	0.3131169
694958.19	0.26908094	0.2318949	0.3105337	0.25925925	0.10423567	0.5341732
537645.5	0.63796681	0.5722306	0.70918405	0.65217394	0.36501679	1.0756617
646830.63	1.8443778	1.7411903	1.9520833	1.8928572	1.4178799	2.4759037
620311.88	0.11768274	0.0922445	0.14796838	0.10714286	0.02209543	0.3131169
627851.38	0.24209552	0.2051386	0.28378728	0.25925925	0.10423567	0.5341732
486579.06	0.79740381	0.7200246	0.88083279	0.78260869	0.4638235	1.2368591
584898.56	1.9815402	1.8690882	2.0989897	2.0357144	1.5418301	2.6375039
693191.56	0.12839164	0.103109	0.15799695	0.14285715	0.03892376	0.365771
688917.56	0.30918068	0.2690509	0.35360819	0.2962963	0.1279197	0.5838218
544280.06	0.76431239	0.6926211	0.84140903	0.78260869	0.4638235	1.2368591
644735.63	2.0551059	1.9459264	2.1688161	2.1428571	1.6352258	2.7582819
670811.94	0.10584188	0.0826634	0.13350514	0.10714286	0.02209543	0.3131169
667089.5	0.23235263	0.1972137	0.27194688	0.22222222	0.08155164	0.4836842
527723.88	0.60069293	0.5363798	0.67059398	0.60869563	0.33277959	1.0212879
624977.06	1.798466	1.6948498	1.9067599	1.8571428	1.3870031	2.4353957
693938.38	0.14410502	0.1172496	0.1752703	0.14285715	0.03892376	0.365771
688965.25	0.31351364	0.2730951	0.35822889	0.2962963	0.1279197	0.5838218
546792.19	0.75897205	0.6876981	0.83562666	0.73913044	0.43057072	1.1834195

646915.5	2.0126276	1.9047774	2.1249936	2.0714285	1.5729226	2.6778018
671581.06	0.12209993	0.0971097	0.15155821	0.10714286	0.02209543	0.3131169
667088.88	0.29381391	0.2541184	0.33795193	0.2962963	0.1279197	0.5838218
530210.19	0.66388768	0.596337	0.73699486	0.65217394	0.36501679	1.0756617
627366.38	1.8474053	1.7425654	1.9569044	1.9285715	1.4488022	2.5163674
694626.19	0.10221325	0.0798294	0.12892811	0.10714286	0.02209543	0.3131169
689340	0.25241536	0.2163027	0.29283324	0.25925925	0.10423567	0.5341732
549166.81	0.62094063	0.5567765	0.69047099	0.60869563	0.33277959	1.0212879
649318.56	1.6709826	1.573022	1.7734462	1.7142857	1.263979	2.2728941
695159	0.12227418	0.0976683	0.15119392	0.10714286	0.02209543	0.3131169
689452.44	0.24947333	0.2135828	0.28966901	0.25925925	0.10423567	0.5341732
550359.56	0.69590867	0.6279504	0.76921624	0.69565219	0.39762533	1.1296955
650335.69	1.7575538	1.6571275	1.862475	1.8214285	1.3561732	2.3948421
673388.88	0.12474219	0.0994993	0.15443932	0.10714286	0.02209543	0.3131169
667358.25	0.29369533	0.2540159	0.33781552	0.2962963	0.1279197	0.5838218
533736.31	0.61078852	0.546278	0.68082261	0.60869563	0.33277959	1.0212879
630432.75	1.8891785	1.7833972	1.9995956	1.9642857	1.4797689	2.556788
696534.75	0.12346835	0.0987588	0.15248242	0.10714286	0.02209543	0.3131169
689662.31	0.25954732	0.2229165	0.30047989	0.25925925	0.10423567	0.5341732
552657.75	0.63692224	0.5721152	0.70705998	0.65217394	0.36501679	1.0756617
652271.19	1.7385406	1.6388131	1.84275	1.7857143	1.3253915	2.3542416
674360.38	0.12604536	0.1006806	0.15585704	0.14285715	0.03892376	0.365771
667666.63	0.30853721	0.2678403	0.3536703	0.2962963	0.1279197	0.5838218
535721	0.68132478	0.6132127	0.75493455	0.69565219	0.39762533	1.1296955
631919.25	2.1221066	2.0100331	2.2388024	2.1785715	1.6664345	2.7984662
697618.69	0.09174066	0.0706515	0.11715078	0.10714286	0.02209543	0.3131169
689595.81	0.2334701	0.1987992	0.27244875	0.22222222	0.08155164	0.4836842
554996.69	0.66667062	0.6004635	0.73818392	0.65217394	0.36501679	1.0756617
653075.63	1.5572469	1.462998	1.6559744	1.6071428	1.172261	2.1504836
698605.31	0.11880814	0.09463	0.14728057	0.10714286	0.02209543	0.3131169
688926.56	0.27433982	0.2366206	0.31636256	0.25925925	0.10423567	0.5341732
556681.31	0.7760275	0.704565	0.85277325	0.78260869	0.4638235	1.2368591
652665.75	1.8922397	1.7881646	2.0007918	1.9642857	1.4797689	2.556788
630808.25	0.1426741	0.1147268	0.17537069	0.14285715	0.03892376	0.365771
622633.31	0.29873121	0.2573409	0.3448841	0.2962963	0.1279197	0.5838218
503565.66	0.6890859	0.6184816	0.76554149	0.69565219	0.39762533	1.1296955
590276.94	1.9821205	1.8701578	2.0990348	2.0357144	1.5418301	2.6375039
699624.56	0.10862969	0.0855878	0.13596626	0.10714286	0.02209543	0.3131169
688182.44	0.31532335	0.2747621	0.36018607	0.33333334	0.15242122	0.6327705
559657.81	0.70042801	0.632798	0.77331734	0.69565219	0.39762533	1.1296955
654172.19	2.0529764	1.9446334	2.1657844	2.1428571	1.6352258	2.7582819
677212.63	0.10041159	0.0779735	0.12729563	0.10714286	0.02209543	0.3131169
666157.94	0.23417871	0.1988725	0.27394599	0.22222222	0.08155164	0.4836842
542669.81	0.61179006	0.547744	0.68126792	0.60869563	0.33277959	1.0212879
634291.06	1.6112477	1.5139657	1.7131408	1.6785715	1.2333511	2.2321441
700857.88	0.1255604	0.1007031	0.15469393	0.14285715	0.03892376	0.365771
687786.69	0.23989996	0.2046913	0.27942643	0.22222222	0.08155164	0.4836842
562298.19	0.64734334	0.5825419	0.71738279	0.65217394	0.36501679	1.0756617
656717.38	1.9399518	1.8348756	2.0494769	2	1.5107785	2.5971663
656124.81	0.09906652	0.0764574	0.1262684	0.10714286	0.02209543	0.3131169
643514.25	0.22377127	0.188716	0.26344952	0.22222222	0.08155164	0.4836842
527021.13	0.69067442	0.6215353	0.76540202	0.69565219	0.39762533	1.1296955
615892.56	1.8964347	1.7892218	2.0083933	1.9642857	1.4797689	2.556788
210990.41	0.86259848	0.7418259	0.99742913	0.89622641	0.7251007	1.0955904
273959.16	0.62053043	0.5307548	0.72114205	0.64150941	0.49815717	0.813266
354589.84	0.58659321	0.5095795	0.67195892	0.76415092	0.60684615	0.9497699
117020.97	1.0767301	0.8969449	1.2819843	1.0849056	0.89570087	1.3022649
315831.97	0.88021487	0.7797757	0.99000263	0.94339621	0.7675848	1.1474226
406771.91	0.82109898	0.7353917	0.91405231	0.8490566	0.68274176	1.0436348
254938.38	0.78058082	0.67589	0.89689416	0.75471699	0.59844363	0.9393106
251418.55	0.71593761	0.6151651	0.82850939	0.74528301	0.5900476	0.928845
233552.47	0.77498645	0.6661926	0.89648122	0.81132078	0.64895189	1.0019746
218156.7	0.60506964	0.5062568	0.71753538	0.6226415	0.4815509	0.7921526

283333.66	0.61058754	0.5229895	0.70866072	0.6226415	0.4815509	0.7921526
367372.13	0.40558329	0.3430755	0.47618538	0.5471698	0.41548899	0.7073439
120964.04	1.2152371	1.0267311	1.4283301	1.226415	1.0246665	1.4562683
326543.94	0.7962175	0.7023682	0.89911467	0.8490566	0.68274176	1.0436348
420674.5	0.63944924	0.5653102	0.72060925	0.66981131	0.52312821	0.8448758
263528.59	0.626118	0.5342264	0.72927868	0.60377359	0.46497923	0.7710054
259988.89	0.70387626	0.6055858	0.81357461	0.7264151	0.57327545	0.9078943
241500.44	0.63767999	0.5409433	0.74672443	0.66037738	0.51479656	0.8343471
218316.27	0.94816571	0.8233917	1.0865059	0.98113209	0.80165601	1.1888057
283600.88	0.8180511	0.7161644	0.93036795	0.83962262	0.67428595	1.0332279
368544.81	0.64849645	0.5688797	0.73613685	0.83962262	0.67428595	1.0332279
121021.84	1.3964422	1.1938376	1.6235778	1.4245284	1.2063795	1.6707249
326817.41	0.96384096	0.8603297	1.0763758	1.0283018	0.84434283	1.2404381
421081.22	0.71245164	0.634104	0.79780602	0.73584908	0.58165812	0.9183729
263657.78	0.8306222	0.7242481	0.94822246	0.81132078	0.64895189	1.0019746
260268.72	0.78764749	0.6835105	0.9031651	0.81132078	0.64895189	1.0019746
241712.52	0.83156639	0.7205735	0.95481628	0.86792451	0.69966966	1.0644324
204382.84	0.8073085	0.6888245	0.94032246	0.83962262	0.67428595	1.0332279
265538	0.71552849	0.6173995	0.82482207	0.73584908	0.58165812	0.9183729
345667.84	0.56701833	0.4904118	0.65219826	0.75471699	0.59844363	0.9393106
113265.7	1.1212574	0.9347418	1.3340836	1.1226416	0.93001539	1.343408
305963.09	1.0001206	0.8911905	1.118692	1.0566038	0.8700043	1.2713689
394260.09	0.79642856	0.7107643	0.8895731	0.83018869	0.66583562	1.0228157
246755.22	0.78215164	0.6756918	0.90062374	0.75471699	0.59844363	0.9393106
243698.2	0.76323909	0.6574896	0.8811568	0.79245281	0.6320914	0.9811105
226280.52	0.85292363	0.7368309	0.98211551	0.8773585	0.70814151	1.0748234
218692.36	0.74076664	0.6310872	0.86402845	0.76415092	0.60684615	0.9497699
284170.06	0.65101862	0.5605834	0.75188965	0.66981131	0.52312821	0.8448758
370536.09	0.48308384	0.4149045	0.55926979	0.61320752	0.47326064	0.7815834
121160.36	0.85836655	0.7013477	1.0400548	0.85849059	0.69120306	1.0540361
327357.53	0.7484172	0.6576282	0.84823698	0.78301889	0.62367016	0.9706697
421898.28	0.59967059	0.528048	0.67829806	0.6226415	0.4815509	0.7921526
263940.31	0.67818362	0.5824691	0.78513831	0.6509434	0.50647283	0.8238105
260777.92	0.75543207	0.6536199	0.86860782	0.78301889	0.62367016	0.9706697
242115.27	0.69388437	0.5929241	0.80710757	0.71698111	0.56489956	0.8974088
211728.09	0.49119604	0.4013428	0.59516621	0.5	0.37453431	0.6540123
275125.94	0.39618221	0.3253068	0.47791365	0.40566039	0.29357842	0.5464219
359147.56	0.29514331	0.2416392	0.35696733	0.38679245	0.27756894	0.5247276
117234.72	0.57150304	0.442907	0.72578865	0.56603771	0.43194643	0.7286028
316798.13	0.50821006	0.4327397	0.59305751	0.52830189	0.39907357	0.6860439
408469.16	0.48228857	0.4172889	0.55454308	0.49056605	0.36637819	0.6433121
255495.2	0.46576217	0.3858453	0.55735385	0.4528302	0.33388123	0.6003872
252406.91	0.45957538	0.379756	0.5512169	0.48113209	0.35823444	0.6325998
234369.78	0.34987447	0.2782655	0.43428645	0.36792454	0.2616303	0.5029649
218898.09	0.50708526	0.4171499	0.61066145	0.52830189	0.39907357	0.6860439
284489.5	0.37259722	0.3050521	0.45064563	0.3773585	0.26959044	0.5138552
372044.34	0.28491229	0.2332628	0.3445932	0.36792454	0.2616303	0.5029649
121136.84	0.44577685	0.3348813	0.58164209	0.46226415	0.34198555	0.6111378
327416.19	0.4734036	0.4018102	0.55407435	0.5	0.37453431	0.6540123
422355.16	0.35988671	0.3049485	0.42186353	0.3773585	0.26959044	0.5138552
264135.09	0.40888166	0.3354136	0.49365917	0.39622641	0.28556514	0.535583
260948.83	0.37938473	0.3083452	0.4618873	0.38679245	0.27756894	0.5247276
242257.05	0.37150621	0.2987349	0.45664421	0.38679245	0.27756894	0.5247276
212012.55	0.49997041	0.4093348	0.60469979	0.50943398	0.38270247	0.6647008
275617.06	0.48981002	0.4106738	0.57974946	0.5	0.37453431	0.6540123
361111.75	0.39046085	0.3286735	0.46048883	0.5	0.37453431	0.6540123
117286.34	0.77587891	0.6246893	0.95260739	0.79245281	0.6320914	0.9811105
317122.81	0.64013052	0.5550963	0.73450619	0.66981131	0.52312821	0.8448758
409172.53	0.52300674	0.4552769	0.59797227	0.5471698	0.41548899	0.7073439
255778.23	0.48088533	0.3996627	0.57376432	0.47169811	0.35010344	0.6218752
252771.92	0.49847308	0.4152414	0.59349573	0.51886791	0.39088234	0.6753779
234620.5	0.48589104	0.4008002	0.58370358	0.50943398	0.38270247	0.6647008
219325.67	0.64287961	0.5411489	0.75817811	0.66037738	0.51479656	0.8343471

285165.38	0.58211833	0.4969317	0.67771822	0.59433961	0.45670688	0.7604185
374302.34	0.4408201	0.3761235	0.51345062	0.58490568	0.44844383	0.7498227
121299.35	0.71723384	0.5744749	0.88470531	0.74528301	0.5900476	0.928845
328057.63	0.58221477	0.5025702	0.6708957	0.61320752	0.47326064	0.7815834
423364.88	0.5361805	0.4686936	0.61065596	0.5471698	0.41548899	0.7073439
264533.28	0.49521178	0.4140455	0.58763856	0.48113209	0.35823444	0.6325998
261509.34	0.58506513	0.4960327	0.68546456	0.60377359	0.46497923	0.7710054
242725.41	0.53558463	0.4474796	0.63596326	0.5471698	0.41548899	0.7073439
219530.36	0.54206628	0.449057	0.6486631	0.55660379	0.42371258	0.7179783
285493.94	0.49738359	0.4189421	0.58624738	0.50943398	0.38270247	0.6647008
375431.5	0.35425904	0.2966137	0.419837	0.47169811	0.35010344	0.6218752
121400.52	0.67545015	0.5372055	0.83841169	0.68867922	0.53981459	0.8659108
328388.13	0.59989989	0.5190494	0.68977445	0.6226415	0.4815509	0.7921526
423850.09	0.56859726	0.4990706	0.64510006	0.59433961	0.45670688	0.7604185
264719.72	0.46086481	0.3827204	0.55027324	0.44339624	0.32579088	0.589623
261803.84	0.46217808	0.3835031	0.55224383	0.48113209	0.35823444	0.6325998
242963.94	0.51036382	0.4244942	0.6085031	0.51886791	0.39088234	0.6753779
212659.41	0.62541318	0.5236454	0.74118537	0.64150941	0.49815717	0.813266
276614	0.51696587	0.4357092	0.60897833	0.52830189	0.39907357	0.6860439
364400.31	0.4500545	0.3838097	0.52444953	0.5754717	0.44019026	0.7392175
117568.47	0.92711931	0.7612615	1.1183819	0.9528302	0.77609587	1.157775
318133.34	0.6978206	0.6090389	0.79590476	0.73584908	0.58165812	0.9183729
410653.13	0.58199972	0.5105469	0.66065347	0.60377359	0.46497923	0.7710054
256353.08	0.57342786	0.4844785	0.67397892	0.5471698	0.41548899	0.7073439
253637.72	0.64264888	0.5477775	0.7492305	0.66037738	0.51479656	0.8343471
235360.31	0.54384702	0.4537185	0.64663589	0.56603771	0.43194643	0.7286028
219946.53	0.65015799	0.5479662	0.76587677	0.66037738	0.51479656	0.8343471
286123.91	0.56968325	0.4855835	0.66416371	0.5754717	0.44019026	0.7392175
377671.09	0.4421837	0.3776612	0.51456821	0.5754717	0.44019026	0.7392175
121554.62	0.87203598	0.7139516	1.0547024	0.89622641	0.7251007	1.0955904
329007.84	0.75986028	0.6685798	0.86012447	0.79245281	0.6320914	0.9811105
424822.97	0.57906473	0.5089579	0.65613025	0.59433961	0.45670688	0.7604185
265055.06	0.59232974	0.5032998	0.69257092	0.56603771	0.43194643	0.7286028
262343.53	0.64419353	0.5507299	0.74897361	0.66037738	0.51479656	0.8343471
243413.38	0.62034386	0.525346	0.72755587	0.64150941	0.49815717	0.813266
212948.56	0.7325713	0.6221246	0.85697359	0.74528301	0.5900476	0.928845
277110.53	0.60625631	0.5180458	0.70518094	0.6226415	0.4815509	0.7921526
366419.28	0.42847091	0.3640697	0.50098187	0.56603771	0.43194643	0.7286028
117646.42	0.73100394	0.5847088	0.90278393	0.73584908	0.58165812	0.9183729
318474.31	0.69707346	0.6083868	0.79505265	0.7264151	0.57327545	0.9078943
411445.19	0.65865397	0.5825619	0.74192393	0.68867922	0.53981459	0.8659108
256591.5	0.68981242	0.5919294	0.79925907	0.66037738	0.51479656	0.8343471
254022.58	0.62986529	0.5360488	0.73537695	0.64150941	0.49815717	0.813266
235621.55	0.56022042	0.4687318	0.66434991	0.58490568	0.44844383	0.7498227
220102.58	0.69967377	0.5935325	0.81931925	0.71698111	0.56489956	0.8974088
286548.97	0.56534839	0.4816418	0.65942109	0.5754717	0.44019026	0.7392175
379582.47	0.42151579	0.3587323	0.49212587	0.55660379	0.42371258	0.7179783
121583.48	0.66620892	0.5290661	0.8280369	0.66981131	0.52312821	0.8448758
329225.78	0.67734671	0.5913566	0.7723254	0.70754719	0.55653071	0.8869165
425456.53	0.52414286	0.4576022	0.59763902	0.5471698	0.41548899	0.7073439
265215.19	0.46754488	0.3888796	0.55745041	0.4528302	0.33388123	0.6003872
262607.97	0.48741856	0.4066416	0.57954228	0.50943398	0.38270247	0.6647008
243529.06	0.55434865	0.4647852	0.65613866	0.5754717	0.44019026	0.7392175
220144.45	0.67228585	0.5683397	0.78974068	0.68867922	0.53981459	0.8659108
286573	0.55134296	0.4687253	0.64432907	0.55660379	0.42371258	0.7179783
380372.94	0.4574458	0.3919997	0.53069407	0.58490568	0.44844383	0.7498227
121585.78	0.69087029	0.5510652	0.85534441	0.70754719	0.55653071	0.8869165
329374.97	0.69829232	0.6109567	0.79460937	0.7264151	0.57327545	0.9078943
425508.19	0.46297583	0.400579	0.53233695	0.48113209	0.35823444	0.6325998
265238.38	0.5881502	0.4994772	0.6880275	0.56603771	0.43194643	0.7286028
262698	0.54435128	0.4587902	0.64123797	0.56603771	0.43194643	0.7286028
243646.59	0.59922856	0.5059729	0.70469207	0.6226415	0.4815509	0.7921526
198830.89	0.61358672	0.5095467	0.73262346	0.63207549	0.48984981	0.8027135

258799.38	0.48686361	0.4055704	0.57967317	0.5	0.37453431	0.6540123
344145.25	0.42423949	0.3582167	0.49890512	0.53773582	0.40727589	0.6966991
109799.08	0.61931306	0.4809208	0.78512686	0.63207549	0.48984981	0.8027135
297516.5	0.73945481	0.6449641	0.84389353	0.7735849	0.61525506	0.9602228
384314.63	0.58285576	0.5090212	0.66439015	0.60377359	0.46497923	0.7710054
239518.91	0.60538018	0.5108568	0.71232295	0.58490568	0.44844383	0.7498227
237244.41	0.49737737	0.4116924	0.5956369	0.51886791	0.39088234	0.6753779
220118.02	0.69053864	0.5851251	0.80945778	0.7264151	0.57327545	0.9078943
220293.78	0.76261801	0.6516569	0.88705665	0.78301889	0.62367016	0.9706697
286792.63	0.63111806	0.5425206	0.73005855	0.64150941	0.49815717	0.813266
382058.88	0.50515777	0.4364	0.58167374	0.6509434	0.50647283	0.8238105
121629.17	0.8303929	0.6763687	1.0090027	0.8490566	0.68274176	1.0436348
329648.25	0.89792681	0.7985373	1.0062677	0.93396229	0.75907844	1.1370655
425911.97	0.6855877	0.6091989	0.76890552	0.70754719	0.55653071	0.8869165
265341.78	0.63314569	0.5410228	0.73645794	0.61320752	0.47326064	0.7815834
262876.75	0.58962995	0.5004594	0.69010633	0.60377359	0.46497923	0.7710054
243896.92	0.66421503	0.5658701	0.77473885	0.68867922	0.53981459	0.8659108
213364.77	0.73114228	0.6209109	0.85530192	0.75471699	0.59844363	0.9393106
277826.75	0.56869972	0.4834812	0.66461313	0.58490568	0.44844383	0.7498227
370747	0.57991028	0.50498	0.66282564	0.76415092	0.60684615	0.9497699
117774.29	0.75568277	0.6068753	0.92993265	0.75471699	0.59844363	0.9393106
319306.63	0.82679147	0.7300564	0.93277812	0.85849059	0.69120306	1.0540361
412620.88	0.63981253	0.564954	0.72183031	0.66037738	0.51479656	0.8343471
256950.53	0.57987815	0.4905083	0.6808207	0.55660379	0.42371258	0.7179783
254643.47	0.69508952	0.5964577	0.80537337	0.71698111	0.56489956	0.8974088
236240.3	0.58415097	0.4907584	0.69014359	0.59433961	0.45670688	0.7604185
220686.89	0.68422735	0.5794464	0.8024801	0.68867922	0.53981459	0.8659108
287433.84	0.62623107	0.5380852	0.72469765	0.64150941	0.49815717	0.813266
384250.22	0.49967441	0.4314926	0.57557058	0.6509434	0.50647283	0.8238105
121783.05	0.68153983	0.5428426	0.84487116	0.6981132	0.54816902	0.8764172
330278.78	0.73574209	0.6461362	0.83429968	0.76415092	0.60684615	0.9497699
426897.75	0.65823722	0.5835172	0.73987287	0.68867922	0.53981459	0.8659108
265715.31	0.60214823	0.5124601	0.70301688	0.58490568	0.44844383	0.7498227
263409.59	0.71751374	0.6188622	0.82742077	0.73584908	0.58165812	0.9183729
244348.77	0.69163436	0.5912877	0.80413085	0.70754719	0.55653071	0.8869165
213760.81	0.75785643	0.6456467	0.88396198	0.76415092	0.60684615	0.9497699
278478.97	0.71100521	0.6154141	0.81723702	0.7264151	0.57327545	0.9078943
372926.69	0.61406171	0.5370991	0.69895738	0.8018868	0.64051867	0.9915454
117925.96	0.69535154	0.5530337	0.8631146	0.67924529	0.53146762	0.8553971
319922.56	0.85645723	0.75804	0.96410477	0.89622641	0.7251007	1.0955904
413592.44	0.72051609	0.6410239	0.80714226	0.73584908	0.58165812	0.9183729
257307.63	0.68789256	0.590282	0.79703456	0.66037738	0.51479656	0.8343471
255168.97	0.6701442	0.5734633	0.77845806	0.68867922	0.53981459	0.8659108
236686.7	0.72669905	0.6221523	0.8437863	0.75471699	0.59844363	0.9393106
221103.39	0.79148495	0.6785597	0.9178316	0.81132078	0.64895189	1.0019746
288102.5	0.65601653	0.5658203	0.75650358	0.66037738	0.51479656	0.8343471
386493.06	0.43726528	0.3738241	0.50838786	0.56603771	0.43194643	0.7286028
121948.68	0.71341485	0.5714161	0.87999457	0.7264151	0.57327545	0.9078943
330904.81	0.77061439	0.6789249	0.87123412	0.8018868	0.64051867	0.9915454
427911.31	0.68238437	0.6063525	0.76531291	0.70754719	0.55653071	0.8869165
266054.16	0.63520902	0.5430489	0.73852777	0.60377359	0.46497923	0.7710054
263951.88	0.70467389	0.6070389	0.8135435	0.7264151	0.57327545	0.9078943
244812.17	0.64539278	0.548682	0.75424069	0.66037738	0.51479656	0.8343471
221252.58	0.6372807	0.536436	0.75157511	0.64150941	0.49815717	0.813266
288410.53	0.62757766	0.5394773	0.72596312	0.64150941	0.49815717	0.813266
387587.78	0.46183085	0.396651	0.53466505	0.58490568	0.44844383	0.7498227
122014.42	0.762205	0.6151978	0.9337526	0.76415092	0.60684615	0.9497699
331185.47	0.72165	0.6330522	0.81917667	0.75471699	0.59844363	0.9393106
428354.91	0.66066712	0.5859287	0.74229711	0.67924529	0.53146762	0.8553971
266192.03	0.64239341	0.5497161	0.74622196	0.61320752	0.47326064	0.7815834
264181.28	0.56022137	0.4736021	0.65809751	0.5754717	0.44019026	0.7392175
245008.17	0.63671345	0.5407188	0.74483758	0.66981131	0.52312821	0.8448758
214276.2	0.72803229	0.6182699	0.85166383	0.74528301	0.5900476	0.928845

279383.03	0.60848361	0.5204508	0.707142	0.61320752	0.47326064	0.7815834
376140.75	0.53703302	0.4655241	0.61641806	0.71698111	0.56489956	0.8974088
118146.57	0.73637348	0.589805	0.90831393	0.74528301	0.5900476	0.928845
320759.28	0.88539916	0.785409	0.99459237	0.93396229	0.75907844	1.1370655
414949.03	0.7037009	0.6252939	0.78921998	0.7264151	0.57327545	0.9078943
257719.7	0.66739172	0.5713772	0.77492326	0.63207549	0.48984981	0.8027135
255872.48	0.78554755	0.680697	0.90197682	0.81132078	0.64895189	1.0019746
237289.47	0.7122103	0.6088784	0.82805353	0.74528301	0.5900476	0.928845
221550.38	0.87113369	0.7525623	1.0030838	0.8773585	0.70814151	1.0748234
288948.47	0.62640929	0.5384729	0.72461158	0.64150941	0.49815717	0.813266
389727.19	0.46955922	0.4039892	0.54273957	0.60377359	0.46497923	0.7710054
122129.3	0.90068471	0.7402533	1.0855687	0.89622641	0.7251007	1.0955904
331698.97	0.80193198	0.7084478	0.90432137	0.83962262	0.67428595	1.0332279
429169.91	0.63378161	0.5606935	0.71375096	0.6509434	0.50647283	0.8238105
266420.03	0.58929503	0.5007212	0.6890226	0.56603771	0.43194643	0.7286028
264591.41	0.63116187	0.5390641	0.73448163	0.6509434	0.50647283	0.8238105
245375.34	0.67651457	0.5775141	0.78761691	0.68867922	0.53981459	0.8659108
214498.77	0.97902662	0.8510823	1.1207763	0.99056602	0.8101849	1.1991405
279802.84	0.77197212	0.6724487	0.88207555	0.79245281	0.6320914	0.9811105
378088.47	0.54220116	0.4705153	0.62172121	0.7264151	0.57327545	0.9078943
118213.2	0.93052214	0.764776	1.1215308	0.93396229	0.75907844	1.1370655
321151.09	0.84383959	0.7463536	0.95052153	0.86792451	0.69966966	1.0644324
415597.16	0.76516402	0.6833671	0.85405636	0.79245281	0.6320914	0.9811105
257889.73	0.60490972	0.51371	0.70763302	0.5754717	0.44019026	0.7392175
256196.08	0.81968468	0.712564	0.93836379	0.8490566	0.68274176	1.0436348
237562.23	0.72823024	0.6237545	0.84519935	0.74528301	0.5900476	0.928845
221729.59	0.90650958	0.7855138	1.0408671	0.93396229	0.75907844	1.1370655
289300.16	0.67404044	0.5827501	0.77557522	0.67924529	0.53146762	0.8553971
391681.13	0.49019468	0.4233064	0.56465095	0.64150941	0.49815717	0.813266
122179.73	0.78572774	0.636442	0.95950806	0.79245281	0.6320914	0.9811105
332035.63	0.88845891	0.7899557	0.99584925	0.91509432	0.74207985	1.1163375
429726	0.64226973	0.5687249	0.72268581	0.66037738	0.51479656	0.8343471
266525.69	0.55154157	0.4659872	0.64825481	0.51886791	0.39088234	0.6753779
264880.5	0.77015865	0.6680936	0.88340682	0.79245281	0.6320914	0.9811105
245589.2	0.72478753	0.6222198	0.83943617	0.74528301	0.5900476	0.928845
221847.64	0.87447405	0.7557439	1.0065643	0.88679248	0.7166186	1.0852094
289513.63	0.61482424	0.5278179	0.71207863	0.63207549	0.48984981	0.8027135
392797.19	0.45061421	0.386673	0.52210933	0.58490568	0.44844383	0.7498227
122214.86	0.76913726	0.6215412	0.94122922	0.7735849	0.61525506	0.9602228
332252.25	0.8096258	0.7157561	0.91238493	0.8490566	0.68274176	1.0436348
430026	0.75809371	0.678025	0.84501809	0.78301889	0.62367016	0.9706697
266610.84	0.70139682	0.6044662	0.8094492	0.66037738	0.51479656	0.8343471
265111.56	0.75439936	0.6534641	0.86651063	0.7735849	0.61525506	0.9602228
245748.83	0.74059355	0.6369029	0.85635394	0.75471699	0.59844363	0.9393106
200467.28	0.92783219	0.7992777	1.0711789	0.94339621	0.7675848	1.1474226
261659.55	0.66880798	0.5733858	0.77557147	0.67924529	0.53146762	0.8553971
355649.59	0.53142196	0.4583563	0.6128239	0.71698111	0.56489956	0.8974088
110407.58	0.88762021	0.720613	1.0817248	0.88679248	0.7166186	1.0852094
300271.28	0.869214	0.766951	0.98131615	0.90566039	0.7335878	1.1059663
388653.5	0.77189577	0.6870111	0.86437178	0.8018868	0.64051867	0.9915454
240861.97	0.71410197	0.6113675	0.82915956	0.67924529	0.53146762	0.8553971
239597.83	0.82221115	0.711399	0.94539148	0.8490566	0.68274176	1.0436348
222072.22	0.87358969	0.7549796	1.0055463	0.89622641	0.7251007	1.0955904
222120.56	0.88690573	0.7673745	1.0197784	0.90566039	0.7335878	1.1059663
289981.44	0.80694818	0.7068604	0.91723573	0.82075471	0.65739089	1.0123979
394880.88	0.5394032	0.4693919	0.61691236	0.70754719	0.55653071	0.8869165
122316.33	1.0055894	0.8357431	1.1998105	1.018868	0.83579713	1.2301199
332739.13	0.87155366	0.77412	0.97785711	0.90566039	0.7335878	1.1059663
430697.63	0.85674953	0.7715535	0.94878328	0.88679248	0.7166186	1.0852094
266801.22	0.69340014	0.5970776	0.80083793	0.6509434	0.50647283	0.8238105
265511.84	0.93027866	0.8178715	1.0538193	0.96226418	0.7846114	1.168123
246075.8	0.75180089	0.6473657	0.8682875	0.7735849	0.61525506	0.9602228
215092.61	0.80430472	0.688915	0.93349296	0.82075471	0.65739089	1.0123979

280856.03	0.70854807	0.6135182	0.81412792	0.71698111	0.56489956	0.8974088
383149.38	0.44108123	0.3770864	0.51282448	0.60377359	0.46497923	0.7710054
118417.74	0.75157654	0.6035777	0.92487961	0.76415092	0.60684615	0.9497699
322244.56	0.78822124	0.6942583	0.8913548	0.82075471	0.65739089	1.0123979
417131.69	0.65686691	0.581385	0.73942804	0.66981131	0.52312821	0.8448758
258268.14	0.5962795	0.5058233	0.69824433	0.56603771	0.43194643	0.7286028
257148.33	0.75831717	0.6556127	0.87254709	0.78301889	0.62367016	0.9706697
238293.88	0.67143983	0.571431	0.78391588	0.6981132	0.54816902	0.8764172
222431.69	0.89915246	0.7788498	1.0327754	0.91509432	0.74207985	1.1163375
290496.34	0.74699736	0.6509081	0.85327655	0.76415092	0.60684615	0.9497699
397104.41	0.52127349	0.4526764	0.59732884	0.71698111	0.56489956	0.8974088
122435.89	0.92293197	0.7606266	1.1096188	0.93396229	0.75907844	1.1370655
333253.34	0.95122826	0.8493851	1.0619203	0.99056602	0.8101849	1.1991405
431487.59	0.73235011	0.6538207	0.81771404	0.75471699	0.59844363	0.9393106
267008.72	0.75652963	0.6557935	0.86836094	0.71698111	0.56489956	0.8974088
265961.13	0.95126683	0.8376508	1.0759948	0.97169811	0.79313147	1.1784666
246432.22	0.84404546	0.733231	0.9668777	0.86792451	0.69966966	1.0644324
215414.36	0.7566812	0.6449758	0.88217479	0.7735849	0.61525506	0.9602228
281363.22	0.72859561	0.632266	0.83545256	0.74528301	0.5900476	0.928845
385374.19	0.4541041	0.3893148	0.52659386	0.58490568	0.44844383	0.7498227
118535.66	0.89424568	0.7321351	1.0815644	0.89622641	0.7251007	1.0955904
322780.09	0.91393495	0.8126072	1.0244046	0.9528302	0.77609587	1.157775
417973.13	0.64118958	0.5667144	0.72273123	0.66037738	0.51479656	0.8343471
258521.52	0.68466252	0.5875103	0.79329211	0.6509434	0.50647283	0.8238105
257609.59	0.79189599	0.6869502	0.90834051	0.82075471	0.65739089	1.0123979
238674.77	0.82120121	0.7102535	0.94456559	0.8490566	0.68274176	1.0436348
222766.88	0.68232763	0.5781676	0.79983276	0.68867922	0.53981459	0.8659108
291016.53	0.60821289	0.5219087	0.70471281	0.6226415	0.4815509	0.7921526
399342.84	0.42319527	0.3617955	0.49202931	0.55660379	0.42371258	0.7179783
122559.7	0.7424953	0.5978109	0.91161972	0.73584908	0.58165812	0.9183729
333844.91	0.76682311	0.6757575	0.86674017	0.8018868	0.64051867	0.9915454
432331.59	0.66152924	0.587075	0.74281102	0.67924529	0.53146762	0.8553971
267293.63	0.6434871	0.5509116	0.74716711	0.61320752	0.47326064	0.7815834
266463.69	0.66050273	0.5665232	0.76561832	0.67924529	0.53146762	0.8553971
246831.92	0.77785724	0.6717168	0.896007	0.79245281	0.6320914	0.9811105
222927.36	0.82538098	0.7104251	0.95364046	0.83962262	0.67428595	1.0332279
291264.25	0.66606182	0.5756285	0.76667118	0.67924529	0.53146762	0.8553971
400471.84	0.47194329	0.4070554	0.54423445	0.64150941	0.49815717	0.813266
122632.91	0.77466971	0.6267541	0.94699353	0.7735849	0.61525506	0.9602228
334137.31	0.8110438	0.7173466	0.91357964	0.8490566	0.68274176	1.0436348
432734.09	0.75334948	0.6737819	0.83972985	0.7735849	0.61525506	0.9602228
267416.41	0.63571268	0.5437405	0.73878592	0.60377359	0.46497923	0.7710054
266699.97	0.66366714	0.5694941	0.76896548	0.68867922	0.53981459	0.8659108
247022.52	0.7165339	0.6148592	0.83022016	0.73584908	0.58165812	0.9183729
215915.22	0.90776372	0.785121	1.0441319	0.9245283	0.75057679	1.1267039
282135.72	0.68761235	0.594253	0.79147691	0.6981132	0.54816902	0.8764172
388697.88	0.50167501	0.4337294	0.5772453	0.6509434	0.50647283	0.8238105
118756.93	0.73258883	0.5867736	0.90364563	0.7264151	0.57327545	0.9078943
323661.75	0.7971285	0.7028198	0.90056622	0.82075471	0.65739089	1.0123979
419200.13	0.7872135	0.7045606	0.87689841	0.8018868	0.64051867	0.9915454
258927.77	0.65655375	0.5615664	0.76300615	0.61320752	0.47326064	0.7815834
258339.56	0.75482053	0.6525896	0.86852372	0.7735849	0.61525506	0.9602228
239281.22	0.7188195	0.6154063	0.83463717	0.73584908	0.58165812	0.9183729
223264.7	0.80621791	0.6927378	0.93298501	0.81132078	0.64895189	1.0019746
291774.44	0.68888831	0.5969394	0.79099131	0.6981132	0.54816902	0.8764172
402805.13	0.44438362	0.3816662	0.51446629	0.56603771	0.43194643	0.7286028
122788.25	0.74925739	0.6040072	0.91889763	0.74528301	0.5900476	0.928845
334714.19	0.80367076	0.7104915	0.90567398	0.83018869	0.66583562	1.0228157
433522.03	0.73352671	0.6551119	0.81874359	0.76415092	0.60684615	0.9497699
267656.94	0.653822	0.5605379	0.75819325	0.61320752	0.47326064	0.7815834
267170.22	0.67747074	0.5823663	0.78367794	0.68867922	0.53981459	0.8659108
247430.02	0.6304813	0.5354263	0.7375471	0.64150941	0.49815717	0.813266
216179.09	0.90202987	0.7798613	1.0379081	0.91509432	0.74207985	1.1163375

282537.03	0.79989517	0.6990006	0.91126209	0.81132078	0.64895189	1.0019746
390787.84	0.49643305	0.4290308	0.57141978	0.6509434	0.50647283	0.8238105
118860.18	0.92545712	0.7606132	1.1154261	0.91509432	0.74207985	1.1163375
324111.53	0.93177801	0.8296412	1.0430173	0.9528302	0.77609587	1.157775
419817.44	0.88848144	0.8005925	0.98338455	0.91509432	0.74207985	1.1163375
259087.64	0.73720229	0.6363561	0.8494904	0.6981132	0.54816902	0.8764172
258719.44	0.81169009	0.7056142	0.92921168	0.83018869	0.66583562	1.0228157
239567.06	0.81814253	0.707608	0.94104743	0.83962262	0.67428595	1.0332279
223483.25	0.66671664	0.5639633	0.78277558	0.66981131	0.52312821	0.8448758
292103.5	0.59910274	0.5136257	0.69473892	0.61320752	0.47326064	0.7815834
404833.03	0.40757543	0.347758	0.47472849	0.52830189	0.39907357	0.6860439
122853.98	0.63490009	0.5018622	0.79238403	0.63207549	0.48984981	0.8027135
335085	0.79979706	0.7068995	0.90150923	0.83018869	0.66583562	1.0228157
434044.38	0.62436014	0.5522298	0.70329452	0.63207549	0.48984981	0.8027135
267742.63	0.63493812	0.543078	0.73788577	0.59433961	0.45670688	0.7604185
267489.78	0.63553828	0.5435913	0.73858327	0.6509434	0.50647283	0.8238105
247651.31	0.67029727	0.5722066	0.78037852	0.68867922	0.53981459	0.8659108
223531.28	0.81867737	0.7043559	0.94626743	0.82075471	0.65739089	1.0123979
292199.13	0.61944062	0.5324825	0.71655041	0.63207549	0.48984981	0.8027135
405925.19	0.50255567	0.4359546	0.5764541	0.66981131	0.52312821	0.8448758
122836.61	0.85479403	0.6991369	1.0347803	0.85849059	0.69120306	1.0540361
335169.34	0.97562623	0.8727354	1.0873128	1.009434	0.82725549	1.2197976
434214.25	0.73005432	0.6518912	0.81500888	0.74528301	0.5900476	0.928845
267691.91	0.72844934	0.6297901	0.83818012	0.68867922	0.53981459	0.8659108
267592.25	0.78851312	0.685702	0.90238988	0.81132078	0.64895189	1.0019746
247719.02	0.87195569	0.7595422	0.99631935	0.89622641	0.7251007	1.0955904
201991.45	0.92083102	0.7932466	1.0630962	0.93396229	0.75907844	1.1370655
264059.88	0.73089486	0.6314116	0.8416031	0.73584908	0.58165812	0.9183729
367487.78	0.53335106	0.4612931	0.61347336	0.6981132	0.54816902	0.8764172
110964.57	0.83810532	0.6764592	1.0267357	0.83018869	0.66583562	1.0228157
302873.81	0.861745	0.7603607	0.97288394	0.90566039	0.7335878	1.1059663
392407.69	0.81547844	0.7285679	0.90990317	0.83962262	0.67428595	1.0332279
241822.66	0.66577715	0.5669076	0.77693093	0.6226415	0.4815509	0.7921526
241824	0.81877726	0.7086967	0.94111139	0.83018869	0.66583562	1.0228157
223852.36	0.82643759	0.7116343	0.95448864	0.85849059	0.69120306	1.0540361
223801.14	0.87130922	0.7533013	1.0025598	0.88679248	0.7166186	1.0852094
292616.53	0.73816746	0.6430022	0.84344941	0.74528301	0.5900476	0.928845
407995.75	0.53677028	0.4680285	0.61276674	0.7264151	0.57327545	0.9078943
122917.91	0.92744827	0.7650304	1.1141487	0.93396229	0.75907844	1.1370655
335589.56	0.98334408	0.8800986	1.0953735	1.018868	0.83579713	1.2301199
434859.53	0.71517348	0.6378895	0.79923987	0.73584908	0.58165812	0.9183729
267854.16	0.88107651	0.772243	1.0009513	0.83962262	0.67428595	1.0332279
267971.78	0.77620113	0.6742939	0.8891601	0.8018868	0.64051867	0.9915454
248030.58	0.80232042	0.6947139	0.92187321	0.83018869	0.66583562	1.0228157
216734.75	0.68286234	0.5772808	0.80216503	0.68867922	0.53981459	0.8659108
283432.94	0.6068455	0.5195415	0.70462173	0.61320752	0.47326064	0.7815834
395897.47	0.4672927	0.4023795	0.53969663	0.63207549	0.48984981	0.8027135
119005.77	0.68063927	0.5405258	0.84597242	0.67924529	0.53146762	0.8553971
325018.75	0.71072823	0.6220235	0.80853444	0.73584908	0.58165812	0.9183729
421177.13	0.64580905	0.5713339	0.72729594	0.66037738	0.51479656	0.8343471
259320.86	0.65170228	0.5571493	0.75770372	0.61320752	0.47326064	0.7815834
259535.91	0.6396032	0.5460042	0.74464369	0.66037738	0.51479656	0.8343471
240207.86	0.64111143	0.5438541	0.75074261	0.6509434	0.50647283	0.8238105
224168.78	0.95463783	0.8310112	1.0914714	0.97169811	0.79313147	1.1784666
293188.56	0.72990566	0.6353821	0.83452719	0.73584908	0.58165812	0.9183729
410317.31	0.44355914	0.3814564	0.51289082	0.58490568	0.44844383	0.7498227
123049.09	0.7476691	0.6027268	0.91694981	0.74528301	0.5900476	0.928845
336197.25	0.81797218	0.7241432	0.92058438	0.85849059	0.69120306	1.0540361
435687.69	0.76660419	0.6865852	0.85338831	0.79245281	0.6320914	0.9811105
268131.03	0.73098588	0.6322267	0.84079778	0.68867922	0.53981459	0.8659108
268464.75	0.71890259	0.6210517	0.82779437	0.73584908	0.58165812	0.9183729
248455.7	0.7687487	0.663587	0.88584185	0.79245281	0.6320914	0.9811105
209905.63	0.92422485	0.7987399	1.06383	0.93396229	0.75907844	1.1370655

274570.38	0.6410014	0.5497966	0.74301344	0.64150941	0.49815717	0.813266
384944.88	0.44162166	0.3777297	0.51322538	0.58490568	0.44844383	0.7498227
115191.6	0.78998816	0.6360492	0.96993041	0.79245281	0.6320914	0.9811105
314845.84	0.84485793	0.7463697	0.95272803	0.8773585	0.70814151	1.0748234
408024.34	0.68623358	0.6082007	0.77150214	0.6981132	0.54816902	0.8764172
250995.52	0.6852712	0.5866845	0.79568356	0.64150941	0.49815717	0.813266
251424.48	0.86705953	0.7557735	0.99011856	0.88679248	0.7166186	1.0852094
232650.05	0.74790442	0.6409029	0.86766225	0.76415092	0.60684615	0.9497699
570052	6.3169675	6.1123123	6.5267286	6.8496542	6.492033	7.2174382
571973.44	5.6051555	5.4127936	5.802608	6.0388689	5.704155	6.3853164
574267.31	4.5588527	4.3858776	4.7369003	4.8781643	4.5786066	5.1908641
582969.5	4.2883892	4.1219177	4.4598584	4.5754361	4.2881675	4.8813033
591431.5	4.5195427	4.3498158	4.6941953	4.839396	4.5398641	5.1496067
594829.94	4.4903588	4.3216658	4.66395	4.7194514	4.4236679	5.0258026
600064.06	4.0329027	3.8738084	4.1968536	4.2187891	3.9400637	4.509407
606358.5	3.9184742	3.7624834	4.0792713	4.0811229	3.8096552	4.3698158
615675.38	4.1645322	4.0048795	4.328917	4.2786708	3.9980466	4.571424
620130.13	3.8830559	3.7294955	4.041316	3.971097	3.7034526	4.2560182
629540.25	3.9965672	3.8419137	4.1558495	4.0945029	3.8193123	4.3801584
638078.19	3.6014397	3.4556816	3.7517655	3.6546488	3.3948092	3.9246614
649576.94	3.503819	3.3613353	3.65079	3.5434399	3.2888272	3.8106434
656580.63	3.5898106	3.4463346	3.7377253	3.6012228	3.346628	3.8728426
665803.31	3.6527302	3.5089858	3.8008518	3.6771894	3.4189045	3.9505661
677576.88	3.7914517	3.6462419	3.9409616	3.7945945	3.5297818	4.0696888
168850.39	3.7015016	3.4169588	4.0034165	3.6402743	3.3851719	3.9142985
170801.58	1.6978766	1.5080658	1.9049667	1.649012	1.4767594	1.83271
173576.13	2.43121	2.2047534	2.6746142	2.3836894	2.1757431	2.6037271
173502.52	2.8990934	2.6512427	3.1638761	2.8425827	2.616009	3.0834613
168938.03	2.6281826	2.389374	2.8843968	2.5234392	2.3095498	2.7499204
172264.27	2.9431527	2.6925075	3.2108502	2.8817508	2.6543713	3.1250992
173800.42	2.9171391	2.6687093	3.1824706	2.8897579	2.6591673	3.1303031
173987.06	3.6726868	3.3934076	3.968823	3.6297221	3.370717	3.8987534
169879.3	3.3965292	3.1250086	3.6853261	3.3318484	3.0866752	3.5927954
175010.19	3.6397882	3.3625848	3.9337499	3.6641979	3.4044471	3.9350235
175079.63	3.6212094	3.3447824	3.9143889	3.5726025	3.3177254	3.8417454
178690.17	4.0517058	3.7619092	4.3579025	4.0325732	3.7613747	4.3180957
175045.48	4.2903132	3.9889159	4.6084485	4.2512226	3.9738855	4.5455852
356403.91	4.6099381	4.3896999	4.8383646	5.6026921	5.1925654	6.0398855
360832.16	4.2789979	4.0681996	4.4978867	5.181386	4.7839379	5.5985126
365449.78	4.265976	4.0568199	4.48312	5.1171293	4.7279038	5.5378804
370849.28	3.9369092	3.7375331	4.1441588	4.6905217	4.3120751	5.0870423
377023.84	3.9148719	3.7176754	4.1198125	4.6210361	4.2481718	5.017612
378547.41	3.7194815	3.527715	3.9189622	4.3288569	3.9688375	4.713613
380345.81	4.4801335	4.2699165	4.6980224	5.1120267	4.7198997	5.5292177
383569.25	4.1817741	3.9796069	4.3915505	4.8251548	4.4479332	5.2345176
388886.19	5.0400348	4.8193526	5.2682166	5.8131018	5.3930993	6.2560182
392190	4.3830795	4.1783051	4.595294	4.9926553	4.6078744	5.4079094
397489.31	4.3397393	4.1373386	4.5494809	4.897336	4.5118961	5.3038878
403494.09	4.7361288	4.5261412	4.9533448	5.3469648	4.9441071	5.7716775
411086.53	4.6121678	4.406878	4.8245525	5.1443443	4.7519169	5.5638671
416540.34	4.525372	4.3233657	4.7343812	5.0034041	4.6158748	5.4165759
422695	4.5233564	4.3228545	4.7307582	4.9798126	4.5918751	5.3905754
429289.16	4.5214281	4.3224974	4.7271523	4.9606709	4.5758772	5.37324
107263.63	4.1673026	3.789885	4.5721321	4.654923	4.2801208	5.0523295
108233.88	1.7924148	1.5490528	2.0631611	1.9619397	1.7237377	2.2253768
110484.86	2.9777834	2.6646717	3.3175766	3.3057539	2.9908633	3.6415863
111078.14	2.5387533	2.2510619	2.8530223	2.7805977	2.4928057	3.0896435
108957.65	2.2302244	1.9586055	2.5289779	2.4491541	2.1779213	2.7378607
110322.74	2.9459021	2.6342957	3.2842319	3.2742515	2.9591739	3.6066091
111666.34	3.286577	2.9588902	3.6406374	3.5798125	3.252605	3.9298449
111674.3	3.3221612	2.9926701	3.6780217	3.6645565	3.332022	4.0170946
110206.66	3.5569539	3.2135115	3.9271049	3.852716	3.5148466	4.2176037
112173.54	3.4678411	3.1317472	3.8301768	3.8363526	3.4989398	4.2001767

114073.84	3.339942	3.0129502	3.6927426	3.7150738	3.3796935	4.0694232
114565.28	3.6136603	3.2739069	3.9790945	4.0163321	3.6660399	4.3830767
112466.49	3.9122765	3.5552158	4.2954845	4.3261371	3.9688377	4.713613
301686.28	0.51709342	0.4391334	0.60490417	0.61264932	0.47326061	0.7710054
312442.03	0.56010389	0.4801909	0.64951462	0.67176437	0.53146762	0.8448759
317349.66	0.60816205	0.5253842	0.70028001	0.72366452	0.57327539	0.8974088
301993.97	0.65564221	0.5674943	0.75360227	0.73056936	0.58165812	0.9078944
330793.28	0.63181454	0.5490554	0.723526	0.76529187	0.61525476	0.9497696
333913	0.61992198	0.5383432	0.71037036	0.75936896	0.60684621	0.9393106
336882.75	0.78662384	0.6947568	0.88725948	0.94893759	0.77609557	1.1474227
341972.16	0.64332718	0.5611201	0.73418909	0.77576053	0.62367004	0.9602228
323348.22	0.77625293	0.6831832	0.87846327	0.87513554	0.70814151	1.0644325
352266.94	0.70117283	0.6164489	0.79428834	0.8294155	0.66583562	1.0123979
357254.06	0.65779519	0.5763753	0.74749386	0.80137569	0.64051872	0.9811105
332769.38	0.82038802	0.7259484	0.92370194	0.90700305	0.74207968	1.1059663
368371.72	0.65694511	0.5767762	0.74514049	0.76829869	0.61525506	0.9497699
344933.09	0.88712859	0.7905052	0.99230397	0.94880027	0.77609557	1.1474227
376997.53	0.81432897	0.7257751	0.91070724	0.92760307	0.7590782	1.1267036
382918.41	0.9114213	0.818297	1.0122401	1.0304458	0.85289264	1.2404383
82376.711	0.91045153	0.7161277	1.1412588	0.88182712	0.71661854	1.0748234
90229.469	0.63172263	0.4784606	0.81847012	0.6660037	0.52312851	0.8343469
84560.086	0.76868415	0.5932541	0.97975105	0.76055247	0.60684597	0.9393105
91703.406	0.78513992	0.6143236	0.98875386	0.83675343	0.67428595	1.0228157
83802.094	0.83530128	0.6511583	1.0553529	0.82473159	0.66583556	1.0123976
84092.016	1.0107975	0.8073893	1.2498667	1.019869	0.84434283	1.2301201
70254.086	0.95368117	0.7390899	1.2111413	0.78426737	0.6320914	0.9706697
79832.578	0.97704476	0.7723134	1.2193962	0.94605154	0.77609605	1.1474226
76735.258	1.1858954	0.9548091	1.4560169	1.0358603	0.85289264	1.2404385
85532.938	1.1457574	0.9301813	1.3963114	1.0957768	0.91285086	1.3125559
73236.18	1.1469741	0.9148713	1.4200319	0.97310722	0.80165607	1.1784666
79419.266	0.99472088	0.7875299	1.239719	0.87757814	0.71661848	1.0748236
85227.297	0.80959976	0.6299169	1.0246003	0.78285235	0.62367016	0.9602228
234351.67	6.1360774	5.8229918	6.4616227	6.3125744	5.7745767	6.8864937
234635.5	5.4041266	5.110734	5.7099719	5.5221224	5.0206876	6.0607648
235460.98	4.1790361	3.9219699	4.4485269	4.2543035	3.8106585	4.7230215
238316.95	3.8142483	3.5702899	4.070487	3.8546841	3.4374511	4.3064198
240274.36	4.2909279	4.0329714	4.5610571	4.3977718	3.9553981	4.8840146
240365.34	4.1852956	3.9306304	4.4521298	4.2473989	3.8106585	4.7230215
242488.03	3.7775061	3.5368075	4.0302734	3.8239601	3.4134104	4.2795048
244447.91	3.3258622	3.1011448	3.5625594	3.3540821	2.9695773	3.7806621
247873.8	3.6268458	3.3936083	3.8718908	3.6382744	3.2332621	4.0774875
250352.41	3.5390112	3.3097866	3.7799277	3.5680459	3.1732762	4.0100842
253839.22	3.5770674	3.3481553	3.8175092	3.611814	3.2092638	4.05053
257456.67	3.2704532	3.0532527	3.4990263	3.2896113	2.9097443	3.7131064
262195.16	3.1808367	2.9685946	3.4042463	3.2039447	2.8260412	3.6184652
265389.44	3.4100831	3.1915016	3.6396928	3.4269314	3.0414245	3.8616807
269005.19	3.6170309	3.393301	3.8516362	3.6506665	3.2452631	4.0909638
273170.31	3.8034878	3.5757027	4.0419788	3.791811	3.3773587	4.2391233
67628.445	3.9628296	3.5025411	4.4667926	3.874121	3.4614964	4.3333297
68698.883	1.7758658	1.4747492	2.1203861	1.7268486	1.45356	2.0355265
69826.93	2.6207654	2.2547975	3.0292089	2.6168075	2.2780638	2.9944665
69774.078	2.8233981	2.442879	3.2463884	2.7869358	2.4325304	3.1712108
68388.938	2.9536941	2.5603931	3.3903131	2.8382516	2.4801254	3.2255268
68959.891	3.0452485	2.6472795	3.4861586	2.9151697	2.5515742	3.3069444
70294.766	2.8593879	2.4777327	3.2831893	2.831486	2.4801254	3.2255268
69701.352	3.3571801	2.9407809	3.8160141	3.2821362	2.8977821	3.6995907
67092.609	3.3535736	2.9296646	3.8215854	3.236876	2.8619049	3.659035
70399.5	3.3096826	2.898324	3.7630568	3.2895422	2.9097443	3.7131064
69133.5	3.6595862	3.2224979	4.1394229	3.6049268	3.1972663	4.0370498
71966.492	3.8073275	3.3698201	4.2858677	3.7656438	3.3533304	4.2121964
69819.891	4.1821895	3.7162061	4.6904407	4.0804772	3.6540239	4.5484452
49655.074	9.8076582	8.9558249	10.718668	9.9282188	8.6645012	11.330553
50182.586	9.4853621	8.6522741	10.377014	9.6136637	8.36092	10.982969

50840.629	8.3791256	7.6022291	9.2138777	8.6028366	7.4093504	9.8880243
51899.559	8.2659664	7.5021811	9.0864239	8.4059486	7.2367878	9.6884947
53215.859	7.7796354	7.0482006	8.5663576	7.8102312	6.6770215	9.0389547
54330.328	7.7857065	7.0613384	8.5642176	7.7818694	6.6770229	9.0389547
55268.043	7.2193623	6.5282817	7.9636927	7.1903911	6.1190305	8.387641
56410.953	7.5517249	6.8515439	8.3040495	7.5074172	6.4192538	8.7385817
57983.309	7.7608542	7.0602732	8.5121431	7.6957808	6.5910554	8.9388733
59166.797	6.7943511	6.1463256	7.492115	6.6821537	5.648438	7.8349738
60602.34	7.1944418	6.5348959	7.9025154	7.0903025	6.0333571	8.2872667
62015.027	6.3532987	5.741374	7.0126853	6.3690434	5.3497925	7.4824548
63523.457	6.2339177	5.6349711	6.8791976	6.1439414	5.1794477	7.280705
64717.059	6.5979514	5.9869018	7.2544513	6.4896207	5.4777012	7.6336169
65873.688	5.5712686	5.0157876	6.1714573	5.5139689	4.5851922	6.5726285
66884.18	5.9655361	5.3944793	6.580595	5.8369675	4.8819242	6.927063
17153.211	6.6459866	5.4821191	7.9838614	6.4497142	5.435051	7.5832434
17189.445	1.8034322	1.2253445	2.5598273	1.7487184	1.2507458	2.3720756
17455.422	2.9217281	2.1754186	3.8415327	2.8515418	2.2109313	3.6445138
17623.482	4.8231101	3.8525298	5.9638505	4.8001757	3.9523509	5.8101144
17270.199	3.0688703	2.2987938	4.0141573	2.9649334	2.2924557	3.7490408
17387.107	4.3710546	3.4438937	5.4710269	4.2189059	3.4077919	5.1453619
17528.928	3.5370104	2.7118056	4.5342875	3.5063655	2.7850869	4.372705
17598.066	6.1370378	5.0343313	7.4094911	6.0415378	5.0518446	7.1292372
17243.195	5.7993894	4.7186146	7.0536118	5.6055827	4.669888	6.6739807
17463.408	6.527935	5.3847418	7.8420458	6.3419175	5.3497925	7.4824548
17713.418	5.1938028	4.1869378	6.3697381	5.0432596	4.1628065	6.0647783
17894.271	6.258986	5.1536298	7.5311875	6.1540146	5.1794477	7.280705
17552.758	6.779561	5.6163044	8.1127548	6.5496392	5.5203648	7.6839762
232563.36	4.3558021	4.0916653	4.6325159	4.8833408	4.4146299	5.3929367
233320.53	3.634485	3.3939488	3.8875697	3.9862552	3.5577242	4.4409242
233728.91	2.8494549	2.6371276	3.074327	2.9803548	2.6111646	3.3747427
237573.77	2.7738752	2.5661063	2.9939871	2.9403431	2.5754049	3.3340693
241581.38	3.0879862	2.8703406	3.3177605	3.4107289	3.0174699	3.8346798
242792.19	2.9696178	2.756784	3.1945221	3.1357963	2.7663002	3.5508177
244287.28	2.6812692	2.4798362	2.894706	2.833725	2.4801254	3.2255268
246493.67	2.5964155	2.3991294	2.8055997	2.7065146	2.3611958	3.0896788
249648.39	2.8279774	2.6231935	3.044502	2.8639345	2.5039344	3.2526734
249415.36	2.6461883	2.4481306	2.8560026	2.7102909	2.3611958	3.089679
252665.58	2.5052879	2.3138971	2.7082868	2.5590272	2.2187469	2.9263945
254813.67	2.1034977	1.9291666	2.2893527	2.0919085	1.7934253	2.4345167
258524.97	2.1854756	2.0089605	2.3733447	2.1841505	1.8758622	2.5304248
260036.58	2.2343011	2.0562928	2.4235952	2.2194555	1.911234	2.5714862
263190.5	2.4924912	2.3053803	2.6907437	2.503649	2.1713328	2.8718975
268610.91	2.7102401	2.5169148	2.914475	2.7368908	2.3849661	3.1168642
66396.43	2.1989133	1.8567047	2.5859196	2.2009826	1.8876495	2.5441146
67187.141	1.2353555	0.9839538	1.5314088	1.2065064	0.97911721	1.4653779
68258.797	1.9191666	1.604611	2.2773616	1.8569776	1.5704275	2.1734371
67902.352	1.8261517	1.5188985	2.1773076	1.7459334	1.4769033	2.063139
65433.777	2.1242852	1.7858298	2.5082269	2.0203021	1.7228807	2.352195
67947.219	2.1340091	1.8008072	2.5109901	2.1540637	1.8522948	2.5030367
67852.531	2.181201	1.8439522	2.562278	2.1583331	1.8522949	2.5030367
68483.875	2.4677343	2.1097	2.8691189	2.4508493	2.123955	2.8173649
67699	2.3781741	2.0250094	2.7752185	2.3707175	2.0529585	2.7354946
69056.836	2.5631061	2.1994069	2.9697723	2.6113205	2.2661963	2.9808564
69880.969	2.6187387	2.2530539	3.0268664	2.5980599	2.2543306	2.9672441
70326.719	2.659018	2.2915509	3.0686479	2.6566906	2.3136795	3.0352843
69520.875	2.8768337	2.4919257	3.3043592	2.9426811	2.5873234	3.3476288
53481.898	12.396718	11.470929	13.377333	12.911386	11.454794	14.493754
53834.789	11.405265	10.520855	12.344168	11.845224	10.449281	13.359732
54236.801	9.9932146	9.1695061	10.871058	10.360523	9.0553417	11.776923
55179.227	9.1157494	8.336421	9.9483175	9.3464994	8.1010065	10.684743
56359.914	8.5521774	7.8056326	9.3508625	8.6973	7.4956861	9.9877348
57342.09	9.0858221	8.3222904	9.9005709	9.1627579	7.9278903	10.485765
58020.699	7.7558527	7.0557227	8.5066566	7.7458668	6.6340327	8.9889193

59005.953	8.4228792	7.6985502	9.1969995	8.3282909	7.1936698	9.638588
60169.926	8.4593754	7.7403426	9.2272282	8.3651848	7.1936707	9.6385889
61195.555	7.5168858	6.8455706	8.2362385	7.3350534	6.2476287	8.5381126
62433.113	8.6332388	7.9196944	9.3938141	8.46735	7.32305	9.7882786
63792.793	8.2454453	7.5557647	8.9811621	7.9788847	6.8490787	9.2389927
65333.332	7.3622451	6.7189202	8.0505495	7.1454782	6.0761876	8.3374596
66437.563	6.6829667	6.0757222	7.3344707	6.3942485	5.3924146	7.532856
67733.961	6.4369483	5.8468451	7.0704699	6.2220359	5.2220116	7.3311644
68911.469	5.8480835	5.2909851	6.4478855	5.6242342	4.669888	6.6739807
17672.309	5.4888129	4.451056	6.6958861	5.23211	4.3315344	6.2681432
17726.117	3.0463526	2.2885156	3.974828	2.8807716	2.2109323	3.6445146
18034.971	3.1605263	2.3937519	4.0948291	2.9877968	2.3332851	3.8012359
18202.592	5.3289113	4.3213868	6.5008197	5.0922117	4.2049594	6.1156497
17845.119	2.8018866	2.0796142	3.6939378	2.7695484	2.1295962	3.5397999
17970.043	4.2292609	3.3321762	5.293551	4.079483	3.2827241	4.9913573
18124.205	5.2967839	4.2904115	6.4682808	5.1564608	4.2471313	6.1664987
18203.768	7.0315118	5.8662214	8.3604898	6.791256	5.7766304	7.9858522
17844.492	5.0996127	4.1058898	6.2611942	4.9141192	4.0364709	5.9120426
18090.441	6.2463923	5.1479115	7.5098877	6.1986341	5.2220116	7.3311644
18351.736	5.7760201	4.7289329	6.98593	5.5421386	4.6275315	6.623313
18502.693	8.1609735	6.9112225	9.5714083	7.9093046	6.8060489	9.1889992
18151.959	7.712666	6.4880366	9.1012487	7.3539243	6.2905178	8.5882473
97345.891	1.1299912	0.9287154	1.3619452	1.1449986	0.81127435	1.613374
100223.13	1.3769276	1.1567879	1.6267674	1.3956467	1.0205923	1.9040812
102074	1.0776496	0.8856969	1.2988594	1.0884298	0.75224984	1.5295312
104271.77	1.3426453	1.129458	1.5843741	1.3532031	0.96039474	1.8214153
106597.28	1.2101622	1.0103525	1.4379222	1.2222545	0.87067074	1.6968427
107325.74	1.1553612	0.9609697	1.3775289	1.1528192	0.81127435	1.6133736
107478.38	1.5444968	1.3184764	1.7981457	1.5347835	1.1114039	2.0275643
108097.87	1.5911508	1.3622392	1.8475204	1.5934705	1.172261	2.1095695
108197.61	1.6543804	1.4208919	1.9152887	1.6422946	1.2027779	2.1504836
107474.52	1.6096839	1.37875	1.868233	1.5929377	1.172261	2.1095695
106521.03	1.6053169	1.3737196	1.8647805	1.5767655	1.172261	2.1095695
105997.47	1.7830614	1.5379069	2.0561864	1.7580963	1.3253922	2.3135927
105666.42	1.7602565	1.5163667	2.0322099	1.7269657	1.2946594	2.2728941
104724.96	1.8333738	1.5832056	2.1118474	1.8005632	1.356174	2.3542414
104246.99	1.6978908	1.4569637	1.9672805	1.6594517	1.2333511	2.1913412
104958.19	1.8197724	1.570835	2.0969539	1.8082161	1.3561727	2.3542414
27420.164	1.7140671	1.259432	2.2793458	1.7099158	1.263979	2.2321441
106718.02	1.265016	1.0606334	1.4972996	1.2594531	0.90049821	1.7384474
27529.404	0.65384632	0.38751	1.0333596	0.67087352	0.40854326	1.0159917
27936.479	1.3244332	0.9325231	1.8255568	1.3423213	0.96039402	1.8214152
28075.289	1.3535035	0.9578194	1.8577893	1.354427	0.96039474	1.8214154
27495.414	0.98198193	0.6471323	1.4287329	0.98715162	0.66449213	1.4029858
107120.29	1.2882714	1.0823058	1.5220248	1.2827805	0.90049833	1.7384474
27719.582	1.1544186	0.7896215	1.6296955	1.1728761	0.81127369	1.6133733
28013.211	1.1780157	0.8108918	1.6543725	1.1678188	0.81127346	1.6133733
28120.711	1.635805	1.1976148	2.1819346	1.6687607	1.2333511	2.1913412
27622.398	1.3756952	0.9735236	1.8882492	1.426079	1.0205923	1.9040816
109306.95	1.2167571	1.0187652	1.4419947	1.222865	0.87067074	1.6968427
28156.449	1.3140862	0.9252379	1.8112947	1.3519533	0.96039468	1.8214153
28670.121	0.97662646	0.6489612	1.4114982	0.97998691	0.66449249	1.4029859
28838.396	1.3176877	0.9324741	1.8086294	1.3512751	0.96039462	1.8214153
28334.74	1.4116946	1.0085353	1.9223273	1.4704159	1.081068	1.9864689
80935.992	3.7313437	3.3223326	4.1768064	3.7110901	3.0468955	4.5046959
82302.539	3.9609959	3.5426416	4.4151711	3.9307518	3.2477438	4.7482948
82311.516	4.0213089	3.5997148	4.4787145	4.0017467	3.3148274	4.82936
82388.445	3.6170118	3.2179587	4.051878	3.6249502	2.9467087	4.3826585
83716.227	3.7627113	3.3586164	4.202033	3.7888618	3.113776	4.5859647
84339.938	3.4977498	3.1099553	3.920532	3.5291657	2.8800111	4.3012066
85557.75	4.4297562	3.9949551	4.8989706	4.5224476	3.7861121	5.3951173
87550.773	3.6664438	3.2762804	4.0902853	3.721894	3.046896	4.5046964
90771.398	5.0125918	4.5625343	5.4950385	5.1001854	4.2939596	5.9983854

93017.156	4.182024	3.7767136	4.6189809	4.2219558	3.4828126	5.0317464
95914.422	4.0452728	3.6527238	4.468513	4.0921149	3.3819752	4.9103618
98510.438	4.4462295	4.0395336	4.8827782	4.5131745	3.7523565	5.3547983
100910.23	4.3206716	3.924577	4.7459102	4.3590331	3.617471	5.1933861
102389.46	4.4047499	4.0075588	4.8306565	4.4254093	3.6848862	5.2741199
103852.01	4.3042016	3.9143858	4.7223301	4.3209162	3.5837848	5.1529975
104943.98	4.13554	3.7555654	4.543539	4.1367755	3.4155724	4.9508371
26113.744	3.7528131	3.046715	4.5734773	3.7454638	3.0803268	4.5453391
106140.74	2.8075931	2.4978404	3.1451442	2.8041811	2.2177539	3.4819679
26160.041	2.0642169	1.5507045	2.6933544	2.0597279	1.5667332	2.6514833
26584.516	2.8588071	2.2524147	3.5782235	2.8612747	2.283546	3.5643256
26648.783	2.5892365	2.0145802	3.2768445	2.5853853	2.0210531	3.2342196
26089.131	2.4914591	1.9228551	3.1755688	2.4904385	1.9557271	3.1513937
106438.79	3.1191635	2.7926295	3.4733908	3.1149135	2.5146289	3.8517673
26386.771	3.1076179	2.4715807	3.857374	3.1111856	2.5146284	3.8517671
26657.553	3.9388463	3.2215869	4.7682137	3.933167	3.2477438	4.7482948
26680.451	3.1483724	2.5112648	3.8978992	3.1428461	2.5146289	3.8517661
26149.834	3.9770806	3.249562	4.8188992	3.9656136	3.2812774	4.7888355
106256.43	3.5668428	3.2167406	3.9446542	3.5555859	2.9133496	4.3419423
26466.861	3.6649604	2.9720352	4.4709411	3.6671371	3.0134821	4.4640355
26751.258	3.2895648	2.6383274	4.0528359	3.2749527	2.6471965	4.0154986
26739.592	3.7397728	3.0428283	4.5485663	3.7296951	3.0468938	4.5046964
26134.008	3.7881677	3.0788357	4.6119585	3.7736373	3.0803254	4.5453396
56344.23	9.0160074	8.2489243	9.8352251	9.1262074	7.937222	10.405981
56524.422	8.2619152	7.5294843	9.0463476	8.3825979	7.2491512	9.6157875
58444.805	8.48664	7.7561088	9.2674408	8.6099424	7.4917517	9.8949289
60929.453	7.2707038	6.6093273	7.9803414	7.3468151	6.2817578	8.4962206
62991.305	7.524848	6.8625894	8.2337646	7.6874566	6.6036553	8.869978
64206.078	7.1644306	6.5245924	7.8500543	7.2792225	6.2415624	8.4494581
65076.012	9.4812202	8.7477617	10.259757	9.6052713	8.3835411	10.916184
66497.211	7.5792651	6.9319234	8.2707844	7.6837344	6.6036553	8.869977
67761.984	9.5333691	8.8122911	10.297726	9.5392265	8.3429327	10.869836
68715.297	7.436481	6.8056026	8.1101055	7.4592304	6.4023995	8.6364498
69759.813	7.2391248	6.6214333	7.8989272	7.2312098	6.2013779	8.4026861
71163.398	8.1643095	7.5138531	8.8560057	8.175252	7.0875754	9.4295368
71867.563	7.6807947	7.0533299	8.3491096	7.6714306	6.6036549	8.869977
72013.648	7.4291472	6.8128805	8.0861902	7.4584184	6.4023995	8.6364498
73130.898	7.5344348	6.9183798	8.1906338	7.5485053	6.4828739	8.7298889
74465.539	7.7619796	7.1420074	8.4213629	7.7826171	6.7245178	9.009985
18553.535	6.683362	5.5588751	7.9685249	6.7031598	5.7199631	7.8406205
76719.516	4.3274517	3.8744264	4.8188977	4.358304	3.5767906	5.2881217
18703.715	2.9940577	2.2616792	3.8880339	3.0842957	2.4109387	3.8452518
19073.18	4.561379	3.6534774	5.6264434	4.536716	3.7338958	5.4788442
19227.082	3.484668	2.7005703	4.425405	3.4971204	2.7967694	4.3289967
18922.527	3.8578358	3.0239275	4.8506494	3.857342	3.1075785	4.7138467
78585.211	4.402864	3.9511089	4.8921142	4.401536	3.6160359	5.3358331
19250.74	4.4154143	3.5268767	5.4597282	4.4177771	3.6160364	5.3358331
19553.016	4.5517273	3.6554122	5.6012926	4.5527854	3.7338963	5.4788446
19678.965	5.0815682	4.1345663	6.1805491	5.0832491	4.2070518	6.0491714
19418.35	5.7677407	4.7491398	6.9400916	5.7978945	4.8814459	6.853045
81372.578	5.7390342	5.2302604	6.2839298	5.743134	4.8416576	6.8058763
19830.615	5.6982603	4.6961727	6.8508816	5.6549788	4.7621226	6.7114973
20253.842	5.826055	4.8223796	6.9770222	5.795331	4.8814459	6.853045
20422.631	6.1206608	5.0947843	7.292491	6.2190785	5.2800617	7.323997
20117.521	6.3626127	5.3081751	7.565167	6.3406191	5.3599377	7.418035
62557.285	11.189744	10.376055	12.050289	11.621968	10.413157	12.940196
62734.746	9.5640783	8.8140144	10.360911	10.057324	8.9301338	11.280359
63398.824	9.5585365	8.8125525	10.350798	9.7987795	8.6948185	11.015673
64264.348	8.6050825	7.9027319	9.3531151	9.0216331	7.9565196	10.182544
65454.926	8.0360651	7.3638973	8.7530985	8.3477306	7.3205862	9.4613323
65748.625	7.680769	7.025393	8.3808241	7.8320866	6.8531237	8.9287939
66125.688	7.7277079	7.0721235	8.4277134	7.880425	6.8864799	8.9668665
66624.297	8.7505608	8.0545692	9.4906006	9.0090294	7.9565196	10.182544

67628.109	9.6409616	8.9150429	10.410242	10.29328	9.1656294	11.544864
68541.602	9.1185493	8.4175863	9.8623095	9.4462471	8.358984	10.637223
69975.945	9.1602907	8.4647875	9.8977051	9.3773355	8.2918644	10.561484
70935.852	9.6566124	8.946907	10.407644	9.9777651	8.8628826	11.204754
73586.609	9.4038849	8.7161913	10.131413	9.602107	8.4932699	10.788651
76282.086	8.861845	8.2063122	9.5558109	9.0345945	7.9565196	10.182544
78447.531	8.9231615	8.274292	9.609396	9.0396852	7.9900351	10.220457
80661.508	8.6162539	7.987494	9.281353	8.7772655	7.7220359	9.9170265
18987.32	8.8480101	7.5606222	10.291766	8.8196983	7.7555203	9.9549713
82761.781	5.6910324	5.1886115	6.2289672	5.8114901	4.9620371	6.7484436
19540.932	3.1216526	2.3878167	4.0098929	3.066551	2.4567463	3.7537076
20289.463	6.111547	5.0832686	7.2867537	6.1930618	5.3252177	7.17098
20406.275	4.9984627	4.0756478	6.0677924	5.0249715	4.2388515	5.9001961
20042.33	3.74208	2.9433823	4.6907296	3.8535376	3.1640892	4.6178064
84197.594	5.7008758	5.2022176	6.2344365	5.8317118	4.9950123	6.7868967
20399.113	5.9316306	4.9219098	7.0875411	6.0872874	5.2260737	7.0558386
20712.17	6.4696264	5.4206419	7.6623836	6.4653206	5.5899339	7.4776931
20376.082	6.4291062	5.3753619	7.6290407	6.5394683	5.6561856	7.5542989
20445.982	6.3582172	5.312274	7.5498667	6.2580986	5.3913522	7.2477026
85832.031	6.6991305	6.1626868	7.2697687	6.817986	5.8882852	7.8221993
20801.014	6.4419942	5.3974895	7.6296568	6.674098	5.7556162	7.6691537
21124.404	6.7220826	5.6619544	7.9230671	6.8822107	5.9546599	7.8986821
21215.971	6.7873402	5.7240572	7.9908442	6.9612384	6.0210605	7.9751391
20832.891	7.6801634	6.5362272	8.9667034	7.844234	6.8531237	8.9287939
180204.42	0.16647761	0.1123217	0.23765704	0.17051479	0.08718887	0.310633
184643.25	0.18413888	0.1275215	0.25731567	0.19065142	0.09983908	0.3343704
186619.14	0.20898178	0.1486064	0.28568485	0.21755427	0.11273726	0.3578552
167511.23	0.22685045	0.1605329	0.31136996	0.20452769	0.11273796	0.3578557
192724.63	0.19717251	0.139531	0.27063468	0.20032889	0.11273775	0.3578547
194157.47	0.20086789	0.1428366	0.27459291	0.21459664	0.11273759	0.3578553
195650.48	0.2402243	0.1765078	0.3194474	0.2464413	0.13916229	0.404189
198444.08	0.21164653	0.1525362	0.28608462	0.21482217	0.11273757	0.3578553
176970.39	0.30513579	0.2292276	0.39813587	0.27614966	0.16627952	0.4498222
203614.86	0.19644931	0.1403463	0.26750818	0.20074877	0.11273775	0.3578547
205733.73	0.12151629	0.0786389	0.17938203	0.12311934	0.05117018	0.237445
178465.8	0.22413258	0.1601236	0.30520496	0.19756034	0.09983914	0.3343701
210232.7	0.15221228	0.1041131	0.21487844	0.15450807	0.07482497	0.2866035
183548.56	0.26695934	0.197498	0.35293439	0.22991632	0.12585385	0.3811197
211960.88	0.25476399	0.1913867	0.33241165	0.25290993	0.13916239	0.4041892
213872.78	0.22910815	0.1694955	0.30289313	0.22666708	0.1258537	0.3811197
41399.547	0.28985822	0.1497747	0.5063234	0.21922535	0.12585378	0.3811192
216834.42	0.18908438	0.1356902	0.25651431	0.18802772	0.09983921	0.3343705
48141.336	0.18694952	0.0854854	0.35488847	0.16416606	0.08718881	0.3106329
41686.684	0.26387325	0.1317244	0.47214144	0.26527703	0.13393056	0.4485453
48560.922	0.2059269	0.09875	0.37870693	0.17969327	0.08718853	0.3106328
47115.246	0.36081737	0.2101894	0.5777036	0.3120214	0.19396228	0.4948848
217375.53	0.19321401	0.1392517	0.26116922	0.19102818	0.09983905	0.3343704
40982.629	0.21960524	0.1004177	0.41687918	0.16191433	0.07482505	0.2866036
27440.117	0.36442992	0.1747583	0.67019969	0.23933105	0.11696113	0.4167028
41659.805	0.19203164	0.0829056	0.37837887	0.14301458	0.06279694	0.2622305
34839.551	0.37313914	0.1986809	0.63807881	0.31134418	0.16882879	0.5112582
219347.56	0.22338976	0.165265	0.29533309	0.22112118	0.12585415	0.3811202
41457.242	0.41006103	0.2388756	0.65654743	0.30815712	0.18005681	0.4724182
28364.178	0.31730163	0.1450908	0.60233736	0.16067868	0.07482502	0.2866036
34282.969	0.35002801	0.1808654	0.61142772	0.29121929	0.15123302	0.4800497
41808.785	0.28702101	0.1483086	0.50136733	0.21458289	0.11273759	0.3578553
59154.742	0.77762151	0.5693166	1.0372381	0.79621434	0.49735752	1.2368591
59509.715	0.85700291	0.638095	1.1268004	0.86959195	0.56518829	1.342973
61592.23	0.97414887	0.7433782	1.2539223	1.0102986	0.66857618	1.500491
64285.918	0.79333079	0.5906869	1.0430834	0.83340627	0.53115255	1.2900372
66528.117	0.7214995	0.5319768	0.95660365	0.77484471	0.46382251	1.1834184
67858.555	0.73682684	0.546887	0.97141421	0.76320672	0.46382257	1.1834195
68916.813	1.0012071	0.7789987	1.2670916	1.0595229	0.70342094	1.5526133

70504.672	0.70917284	0.5263618	0.93495595	0.72226709	0.4305701	1.1296955
72145.234	0.69304645	0.5143924	0.91369528	0.70080137	0.43057013	1.1296955
73280.461	0.90064937	0.6965622	1.1458467	0.91338158	0.59944707	1.3956839
74442.281	0.67166132	0.49852	0.88550168	0.68050051	0.39762533	1.0756619
76097.047	0.76218462	0.5787587	0.98530072	0.76901984	0.46382254	1.1834196
76910.945	0.66310459	0.4937249	0.87186003	0.67015415	0.39762524	1.0756609
77075.625	0.9471218	0.7423923	1.1908635	0.96093565	0.63391423	1.4481856
78271.688	0.83044076	0.6409166	1.0584649	0.84624928	0.53115219	1.2900369
79735.203	0.92807186	0.7287363	1.1651093	0.93741411	0.59944737	1.3956835
19906.227	0.45211986	0.2067384	0.85826439	0.47070298	0.23874611	0.7995807
82103.93	0.56026548	0.4101847	0.74731565	0.58003223	0.33277977	0.966539
20058.725	0.64809704	0.3450843	1.1082649	0.67667627	0.3976253	1.0756623
20438.592	0.44034344	0.2013535	0.83590907	0.43893334	0.23874609	0.7995814
20589.525	0.77709419	0.4441763	1.2619523	0.81163424	0.49735713	1.236859
14550.341	0.82472295	0.4261483	1.4406233	0.63097095	0.36501664	1.0212879
83904.484	0.65550727	0.493818	0.85323316	0.67095071	0.39762524	1.0756607
20590.84	0.679914	0.3717155	1.14078	0.69312423	0.39762497	1.0756613
20906.803	0.76530111	0.4374355	1.2428011	0.7839669	0.49735782	1.2368596
16200.92	0.8024236	0.4272567	1.3721677	0.61581761	0.36501679	1.0212879
20723.805	1.2063421	0.7806811	1.780799	1.2452381	0.84442389	1.75947
86660.297	0.79621238	0.6195006	1.0076576	0.80299073	0.49735734	1.2368597
21151.646	0.8982752	0.5408217	1.402768	0.89241761	0.56518757	1.3429731
21596.887	0.50933266	0.2542567	0.91133553	0.51159394	0.26959005	0.8557408
21773.668	0.55112445	0.2847754	0.96270227	0.5499925	0.30095488	0.9113733
21449.521	0.83917952	0.49735	1.3262662	0.86135948	0.53115433	1.2900374
62327.117	1.2835504	1.0177761	1.5974896	1.3303423	0.96039504	1.8214161
68289.07	1.3179269	1.0597688	1.6199559	1.4543064	1.0507967	1.9453088
69138.281	1.3595942	1.0986905	1.6637989	1.4823602	1.081068	1.9864689
70196.82	1.5527769	1.2749914	1.8731114	1.6793923	1.2639779	2.2321441
71540.531	1.719305	1.4289106	2.0513744	1.8671936	1.4178799	2.4353952
71896.977	1.6412373	1.3584955	1.965472	1.8263049	1.3870032	2.3948419
72315.445	2.0604174	1.742869	2.4190853	2.2380033	1.7289617	2.8386145
73023.414	1.7528625	1.4623712	2.0841591	1.9215449	1.4488027	2.4759033
74232.586	1.9802624	1.6730866	2.327503	2.1949182	1.6976801	2.798466
75371.617	1.8707306	1.5747021	2.2062407	1.9953244	1.5107787	2.5567877
77078.047	2.0758178	1.7666314	2.423548	2.2329552	1.7289623	2.8386145
78206.516	2.2376652	1.9184058	2.5948691	2.4138947	1.8858807	3.0388441
81228.063	1.9574517	1.6650175	2.286463	2.0545776	1.5729233	2.6375039
84308.906	2.1824503	1.8784872	2.5215905	2.3509254	1.8230151	2.9588513
86764.953	2.1667733	1.8681004	2.4996192	2.3197196	1.7916294	2.9188061
89310.414	2.5304999	2.2113161	2.8828137	2.6857159	2.1385484	3.3576133
21070.936	2.5627718	1.9252346	3.3438604	2.5210755	1.9804128	3.1585991
91525.922	2.0212853	1.7405018	2.33447	2.1019845	1.6040548	2.6778018
22029.41	1.5887852	1.1066471	2.2096157	1.6429896	1.2333506	2.1913412
22434.813	2.0058115	1.4630525	2.6839335	2.1302404	1.6352258	2.7180605
22552.955	2.039644	1.4932756	2.7205992	2.148041	1.666434	2.7582815
22136.51	1.8521439	1.3291304	2.5126424	1.9910014	1.5107785	2.5567877
92908.758	2.206466	1.9147431	2.5300696	2.4068081	1.8858807	3.0388446
22518.549	2.7532856	2.1109283	3.5295877	2.9735346	2.3929174	3.6746781
21907.168	1.8715336	1.3430448	2.5389466	1.9745903	1.5107785	2.5567868
21971.852	2.5942283	1.9648433	3.3611243	2.7947092	2.2337518	3.4766974
21171.904	2.5033176	1.8751562	3.2744002	2.6306	2.0648515	3.3156233
94549.195	2.5172081	2.2075403	2.8581531	2.6363168	2.0752108	3.2780914
22924.047	2.7045836	2.0735886	3.4671538	2.809932	2.2337518	3.4766974
23275.115	2.7497177	2.1176176	3.5113282	2.9480491	2.361037	3.6351292
23362.625	2.3541875	1.7734969	3.0642998	2.4440525	1.9173617	3.078793
21968.99	1.7752295	1.2623606	2.4267962	1.834608	1.3870034	2.3948419
20309.998	19.399311	17.530846	21.412699	19.286329	16.673048	22.11631
27604.295	9.3825979	8.2746153	10.597615	9.3705349	7.725049	11.356133
26593.814	5.001163	4.1873693	5.9269433	5.0709729	3.8456182	6.5081868
76853.922	4.176755	3.7322872	4.6595888	4.2581797	3.5029979	5.1592102
103927.27	4.1182647	3.737299	4.5275326	4.1678047	3.4491849	4.9912992
93583.344	4.3918071	3.9774263	4.8376288	4.4708247	3.7486203	5.318995

28628.863	12.714441	11.441679	14.090082	12.666513	10.706415	14.920251
75643.609	5.4465938	4.9332991	5.998785	5.4595079	4.633956	6.3991323
54257.313	7.2985554	6.5973191	8.0540361	7.3188586	6.2817593	8.4962206
62649.594	7.709547	7.0372396	8.4287605	7.972456	6.9865813	9.0810509
20724.293	18.046453	16.263617	19.971376	17.901951	15.466391	20.722965
27403.75	9.7431917	8.6094542	10.984715	9.7458925	8.0540447	11.754415
27101.572	4.5015841	3.7382934	5.3748975	4.5934935	3.4520748	5.9926143
76620.977	3.9936843	3.5587039	4.4671636	4.0326233	3.2775195	4.8846846
102404.34	3.6521893	3.2913837	4.0417495	3.7041562	3.0468955	4.5046964
95398.133	3.8470356	3.4634686	4.2614737	3.9014399	3.2288079	4.6959448
28787.76	11.358994	10.161059	12.659336	11.328486	9.4589911	13.440392
76659.086	4.0960574	3.655483	4.5751023	4.097517	3.3819752	4.9103622
54310.941	6.3523111	5.6996155	7.0592623	6.3341007	5.3599377	7.418035
62562.57	6.5534391	5.9343667	7.2195425	6.5733886	5.6893225	7.5925908
21265.383	17.963467	16.207027	19.858353	17.854769	15.373709	20.615646
27431.346	6.9263825	5.9764848	7.9843554	6.9357986	5.5242906	8.6477575
27376.328	3.8354304	3.1370029	4.6430225	3.884481	2.8290284	5.1610727
77439.844	2.9700472	2.598583	3.3797126	3.0200803	2.384181	3.7780044
99674.375	3.3910422	3.039124	3.7725282	3.4248636	2.7801101	4.1788826
96599.883	3.1884098	2.8422344	3.5651202	3.2204459	2.616698	3.9509029
29004.375	10.032969	8.9132242	11.254465	10.01286	8.3012114	12.052706
76422.5	3.4937353	3.0871973	3.9389236	3.5049706	2.8466916	4.2604523
56045.148	4.5142179	3.9750552	5.106112	4.5309706	3.7338955	5.4788442
63008.141	4.0312252	3.5506678	4.5586843	4.0695863	3.3584266	4.8520412
22296.162	15.249262	13.671246	16.959454	15.169304	12.879658	17.709682
27686.072	8.2351875	7.2008562	9.37638	8.2494373	6.6606822	10.056844
27799.529	3.8130143	3.1217844	4.6117311	3.8660698	2.8290284	5.1610727
80786.664	2.8346264	2.479352	3.226521	2.8274446	2.2001939	3.5453172
98605.531	2.9714358	2.6409042	3.3318949	3.0093606	2.4154456	3.7287259
98240.539	2.9010427	2.5739791	3.2581532	2.9440193	2.361038	3.6351287
29297.021	8.8063564	7.764472	9.9490948	8.792181	7.1509514	10.657494
76144.141	3.5853055	3.17258	4.0368137	3.5983019	2.9467077	4.3826585
58405.68	4.023581	3.5255542	4.5722475	4.0318499	3.2636087	4.9056454
63708.16	3.9712338	3.4969232	4.4919329	3.9992495	3.2935877	4.7740216
23436.451	13.78195	12.319767	15.369935	13.692664	11.502053	16.087278
28230.854	6.8719144	5.9388924	7.909925	6.8546634	5.4435787	8.5466499
27917.242	3.689476	3.0114691	4.4745626	3.7630789	2.7518096	5.0564642
82101.82	2.9840994	2.6221049	3.3821023	3.069572	2.4210796	3.8244393
98030.969	3.1928685	2.8489094	3.5669124	3.2276001	2.6140211	3.9745991
99777.586	2.856353	2.5343277	3.2079623	2.902159	2.3291798	3.5955572
29991.229	9.2026911	8.1489124	10.354924	9.1991014	7.5608025	11.156741
77117.109	3.7734816	3.3523366	4.2328963	3.7954071	3.1137762	4.5859647
60164.473	5.285511	4.7204838	5.8995514	5.3365445	4.4445662	6.3333988
64663.785	5.0259972	4.4943662	5.6032209	5.2026935	4.4028058	6.0933862
24595.25	13.986441	12.547313	15.545377	13.917454	11.777057	16.412275
28476.043	6.9883304	6.0510612	8.0296535	6.9838324	5.5242906	8.6477575
27985.617	4.0735207	3.3601525	4.8935437	4.1261683	3.0616164	5.473958
82753.055	3.2143829	2.8396704	3.6247902	3.2918038	2.6431272	4.1023989
97592.258	2.9510536	2.6200371	3.3123138	2.9769549	2.3824332	3.6876638
99735.469	3.3388321	2.9898126	3.7174056	3.3982201	2.7771533	4.1475921
30615.508	9.3743343	8.3210497	10.524029	9.3568134	7.6429009	11.256459
77305.508	3.9453852	3.5149822	4.4139481	3.9697113	3.2812774	4.7888355
61123.301	4.3682194	3.8599246	4.9248385	4.4342737	3.6160345	5.3358335
64647.949	4.1455297	3.6640203	4.6727271	4.1385388	3.4233229	4.9300027
25355.658	12.107751	10.791099	13.54074	12.073511	10.040259	14.349058
28709.654	5.2595549	4.4541197	6.1685467	5.2625031	4.0038028	6.7136464
27831.891	4.0241609	3.3134816	4.8421116	4.0839677	2.983937	5.3698134
84229.039	2.742522	2.4002326	3.1199319	2.8165205	2.2001941	3.5453181
98173.031	2.4854076	2.1833105	2.8176188	2.5285141	1.9883742	3.1928232
99602.672	3.3633635	3.0128014	3.7435169	3.4063439	2.7771537	4.1475921
31391.541	8.8877449	7.8753495	9.9941921	8.8712044	7.23283	10.757434
78062.82	3.5612345	3.1548705	4.0054212	3.5801868	2.9133506	4.3419418
61863.813	4.0411348	3.5556815	4.5743661	4.0362892	3.2636087	4.9056458

64843.941	3.5932424	3.1466405	4.0854597	3.6387346	2.9703138	4.3830061
26130.035	11.519311	10.254588	12.896944	11.492491	9.4944296	13.694881
28920.367	7.1575856	6.2156816	8.2018976	7.1585321	5.685914	8.8497753
27922.854	3.8319867	3.1404061	4.6305642	3.8903813	2.8290284	5.1610727
84778.773	2.7837157	2.439862	3.1624539	2.8171458	2.2001941	3.5453181
98393.477	2.4798393	2.1784191	2.8113062	2.4892347	1.9557273	3.1513937
99669.18	2.9096258	2.5843499	3.264513	2.9309077	2.3610368	3.6351287
32443.65	9.4933834	8.4626579	10.615026	9.454978	7.8072362	11.455765
79617.406	3.0897765	2.7157004	3.500983	3.1044073	2.4815443	3.8107774
63115.688	3.834229	3.3663268	4.3489771	3.8609257	3.1075783	4.7138462
65367.051	2.9831543	2.5791235	3.4325252	3.0803685	2.4886937	3.7931893
26638.957	11.486937	10.235813	12.848794	11.467335	9.4944296	13.69488
29115.781	7.1782379	6.2379861	8.2201996	7.1720653	5.6859107	8.8497753
28256.711	4.3175583	3.5854712	5.1551704	4.3771195	3.2954998	5.7855487
85139.961	2.9363413	2.5836046	3.3237941	2.9714744	2.3473153	3.731535
100241.46	2.9827979	2.6542499	3.3407791	3.0432684	2.4484828	3.7697637
99114.445	3.55145	3.190089	3.9425352	3.583698	2.9380779	4.3438139
34141.781	9.4312592	8.4291506	10.519728	9.4178514	7.7250504	11.356133
82255.375	3.5863917	3.1887693	4.0198884	3.5947175	2.9467077	4.3826585
64354.051	3.5273614	3.0833867	4.017312	3.5167069	2.7967694	4.3289967
66416.875	2.7402675	2.3566024	3.1685922	2.809093	2.2337518	3.4766974
26746.689	8.8982983	7.8036261	10.103534	8.8849468	7.1474304	10.841846
29527.682	5.8927755	5.0497041	6.8363533	5.8911614	4.5605116	7.4296885
28491.484	3.8608027	3.1731102	4.6533117	3.894717	2.8290284	5.1610727
85536.445	3.051331	2.6923418	3.44486	3.0773389	2.4210792	3.8244381
101026.6	2.4053071	2.112365	2.7275143	2.4286351	1.890533	3.0684388
97952.508	2.9197824	2.5911646	3.2785344	2.9454932	2.361037	3.6351292
35596.5	9.5515013	8.563098	10.622694	9.5341654	7.8072381	11.455766
83989.227	3.1908855	2.8202593	3.5966783	3.1879823	2.5808678	3.9336774
65178.316	3.620836	3.1735785	4.1134686	3.6185358	2.9131167	4.4735212
66084.672	3.8132896	3.3569698	4.314332	3.630161	2.9703157	4.3830061
26973.164	9.7133579	8.5726957	10.963548	9.6890182	7.8660464	11.723244
30231.961	7.6409202	6.6872702	8.6924191	7.6393714	6.1722703	9.4543381
28903.918	4.1170888	3.4106667	4.9267101	4.1435432	3.0616164	5.473958
86714.922	3.2059073	2.8400888	3.6057746	3.2793839	2.6060438	4.0561471
101988.15	2.5297055	2.2304151	2.8579679	2.5363653	1.9883747	3.1928225
96798.82	3.0268965	2.6901958	3.3940835	3.0572939	2.4567456	3.7537069
36926.41	9.8303623	8.8449879	10.895499	9.8170271	8.0540457	11.754416
86266.781	2.9791305	2.6260049	3.3665087	2.9808404	2.3824332	3.6876638
66008.734	3.4843874	3.0485945	3.9649971	3.4899657	2.7967691	4.3289962
68727.391	3.2738039	2.8599782	3.7306836	3.2452793	2.6166971	3.9509029
27173.467	7.7281265	6.7181749	8.8470535	7.7070737	6.1655765	9.6236801
31208.615	7.6901841	6.7479548	8.7271614	7.6838303	6.1722717	9.4543381
29215.563	4.3812265	3.6551526	5.2092929	4.4106393	3.2954972	5.7855487
87498.266	3.0286314	2.6749277	3.4160953	3.0479035	2.4210804	3.8244393
103498.32	2.1642864	1.8901203	2.4670434	2.1761792	1.6634642	2.7769797
95948.953	2.8035741	2.4785218	3.1594086	2.8367276	2.2655373	3.5163417
38210.176	8.9504948	8.0269232	9.95119	8.9427776	7.3147535	10.857328
88284.867	2.8997042	2.5553439	3.2775354	2.9102204	2.316483	3.605464
67278.898	3.1361985	2.7272818	3.5891271	3.121233	2.4493728	3.8937757
69761.031	2.1932015	1.8594503	2.5695632	2.169647	1.6664345	2.7582819
27420.988	6.8560619	5.9110069	7.909246	6.8264294	5.3686104	8.6206226
32460.457	6.7158637	5.8538904	7.6690245	6.7004461	5.2823558	8.3442307
29549.814	4.3993506	3.6756461	5.2238717	4.4210072	3.2954972	5.7855482
88891.172	2.8686764	2.527355	3.243242	2.8684208	2.2369215	3.5919261
105905.62	2.2000721	1.9266264	2.5014472	2.1930447	1.6957898	2.8187292
95443.93	2.9965239	2.6592689	3.3647048	3.0438998	2.4567463	3.7537076
39425.527	8.6492186	7.7554617	9.6177216	8.6474762	7.0691171	10.557507
90243.141	2.9365113	2.5935662	3.3121898	2.9544699	2.3494456	3.6465766
67837.477	2.8008118	2.416703	3.2286227	2.797287	2.1811142	3.5533292
72398.797	2.3481052	2.0083914	2.7288225	2.341917	1.823014	2.9588518
27292.963	5.7157588	4.8540173	6.6863861	5.7086425	4.4041829	7.3850136
34183.484	7.4889965	6.5996256	8.4648123	7.5038853	6.0099125	9.2530565

29591.768	3.9200091	3.2391794	4.7016768	3.9351852	2.9064059	5.2655196
90226.602	2.9148831	2.5732124	3.2892954	2.9424586	2.3104839	3.6850319
107990.39	2.7224646	2.4201279	3.0521271	2.724638	2.152071	3.3995016
94518.398	2.6555676	2.3371754	3.0052299	2.7122595	2.1385484	3.3576124
40086.402	8.5565176	7.6748514	9.5116949	8.5533619	6.9873323	10.457473
91253.328	3.1998832	2.8433492	3.5887573	3.211355	2.580868	3.9336779
67801.398	3.1562772	2.7475359	3.6086841	3.1475823	2.4878411	3.9422643
73635.883	2.3358176	1.9997743	2.7121694	2.2861748	1.7916296	2.9188061
26994.373	6.4457874	5.5235977	7.4779162	6.4230161	5.0165629	8.172657
35791.449	5.643806	4.8923016	6.4780812	5.6453691	4.4009933	7.2255635
30214.064	3.6075914	2.962208	4.3518295	3.6279304	2.5978715	4.8467531
91613.023	3.3401363	2.9763386	3.7361331	3.3482931	2.6802373	4.1486216
109800.67	2.4225719	2.1401637	2.7318819	2.4393311	1.8905338	3.0684383
94262.141	2.8537438	2.5228748	3.215946	2.9025869	2.3291795	3.5955572
40607.758	7.8310156	6.9938707	8.740778	7.8261504	6.3348575	9.6553898
92191.539	3.5578103	3.1831551	3.964442	3.569797	2.9133501	4.3419423
68676.609	3.5674443	3.1346855	4.0432506	3.5566051	2.8355231	4.3771996
75651.695	2.8419719	2.4747605	3.248316	2.765497	2.2019916	3.4370284
26393.207	5.001287	4.1845355	5.9308882	4.9852672	3.7110963	6.4780593
37225.332	5.9905438	5.2300363	6.8305478	5.9928341	4.6403995	7.5316215
30991.563	4.0010891	3.327899	4.7704701	4.0399284	2.983937	5.3698134
92988.047	3.9037278	3.5124264	4.3267035	3.8521037	3.1276264	4.7012429
111987.24	2.8842573	2.5782547	3.2165873	2.8861146	2.2835467	3.5643253
94971.766	3.0219507	2.6824095	3.3925712	3.0686243	2.4567463	3.7537079
41185.543	7.8425574	7.010509	8.7461939	7.836616	6.3348575	9.6553907
92807.102	3.4695621	3.1009071	3.8699865	3.4802799	2.8133914	4.219677
69659.852	3.5601568	3.1308172	4.0319309	3.5686018	2.8743057	4.425374
79367.203	2.8223243	2.4647999	3.217133	2.8628795	2.2973459	3.5559618
7314.0615	4.7853026	3.3331389	6.6551986	4.7778239	3.5391409	6.2500033
9391.6523	6.3886523	4.8752146	8.2234602	6.3900967	5.0410533	8.0400715
7855.5537	3.1824617	2.0595217	4.6979418	3.2334292	2.2921777	4.4251366
23864.891	3.5198152	2.8075418	4.3577704	3.4179518	2.7545428	4.240983
28146.621	2.5225053	1.9700975	3.1817982	2.5246038	1.9883745	3.1928225
23729.283	3.2027936	2.5234368	4.0087738	3.2974155	2.6808214	4.0296369
10264.252	8.8657217	7.1381259	10.885141	8.865654	7.2328296	10.757434
23079.15	3.2930155	2.5945213	4.1216998	3.3044941	2.6803932	4.0563755
17348.633	3.3432031	2.5386343	4.3218665	3.3202689	2.6420522	4.1358843
17856.295	2.7441301	2.0301225	3.6278856	2.456619	1.9173614	3.0787921
7300.9746	1.7805842	0.948086	3.0448511	1.7710052	1.0667918	2.7218633
9481.8838	2.0038214	1.2064342	3.1292152	2.0039034	1.325457	3.0280242
7841.0786	1.2753347	0.6115726	2.3453863	1.2763351	0.76321691	2.1354198
23917.43	1.546989	1.089223	2.1323206	1.4952338	1.0505816	2.0281887
28374.207	1.5859474	1.1568009	2.1221225	1.5863889	1.1525671	2.1026592
23825.568	1.9726707	1.4494441	2.6232338	1.9904752	1.5107785	2.5567877
10291.625	4.1781545	3.023751	5.6279488	4.1583056	3.0616164	5.473958
23100.396	1.6017041	1.1277474	2.2077382	1.6080034	1.1840913	2.1452117
17470.582	1.3737379	0.8801801	2.044013	1.3765111	0.95165116	1.9131316
19197.838	0.78133804	0.4373089	1.2886983	0.74075377	0.46426097	1.1031555
7358.5488	1.902549	1.0401417	3.1921532	1.8794458	1.1439211	2.8447762
9694.4199	2.5788031	1.6688656	3.8068221	2.5777242	1.765614	3.6788933
7999.7344	2.1250706	1.2379317	3.4024439	2.1612489	1.3979323	3.1373911
24176.52	3.2676332	2.5870159	4.0724459	3.2546477	2.6060436	4.0561471
28942.057	1.8657969	1.4016451	2.4344594	1.8726469	1.4064009	2.4414356
24177.279	2.2748632	1.7137389	2.9610484	2.338495	1.823014	2.9588506
10437.689	4.9819455	3.7207553	6.5331578	4.9656925	3.7666845	6.4052973
23451.453	2.5584769	1.9523875	3.2932663	2.5631292	2.0210531	3.2342198
17797.574	2.2474973	1.6056451	3.0604532	2.2616599	1.7261146	2.9648292
19540.844	1.3305465	0.8691571	1.9495595	1.279235	0.90049845	1.7384473
7360.1396	3.5325418	2.3075736	5.1759934	3.496505	2.4378784	4.7511592
9814.2227	4.7889681	3.5187533	6.3683119	4.7871704	3.6091502	6.1991882
8213.6152	4.1394682	2.8667021	5.784493	4.2194142	3.1394393	5.5779595
24875.23	2.4924393	1.9109389	3.1951942	2.4919031	1.9077632	3.1710708
29215.541	2.2933002	1.7772764	2.91241	2.2984378	1.7929974	2.943748

24255.182	2.3912416	1.81577	3.0912354	2.4666166	1.9488726	3.1187112
10438.098	7.1852179	5.651628	9.0067329	7.1498952	5.6859136	8.8497753
23482.277	2.5977037	1.9870373	3.3368585	2.6161268	2.0537634	3.2755859
17924.656	2.1757739	1.5471866	2.9743533	2.1337123	1.6135206	2.8165476
17923.549	1.8969458	1.3136901	2.6507921	1.7381029	1.2952993	2.3159053
7124.186	2.1055036	1.1784344	3.4727082	2.0951591	1.2999358	3.0888379
9678.8584	2.7895851	1.8383542	4.058702	2.7876775	1.9148167	3.8933518
8096.6816	2.5936551	1.6055166	3.9646773	2.5923424	1.7656139	3.6788929
24377.35	3.1176481	2.456352	3.9022017	3.0092747	2.3841808	3.7780035
28674.359	2.5458283	1.9955231	3.2009971	2.5306993	1.9883739	3.1928227
23744.531	2.5268977	1.9282891	3.2526174	2.5851598	2.0435839	3.2382886
10215.592	3.9155831	2.7973499	5.3319125	3.9235954	2.9064062	5.2655196
22963.156	2.5693331	1.9558954	3.3142514	2.5850585	2.0210531	3.2342196
17612.373	2.2711308	1.622529	3.0926352	2.2331815	1.6885259	2.9154596
16450.945	2.0059636	1.3808131	2.8171198	1.6893132	1.263979	2.2321441
7037.2266	3.6946373	2.4134598	5.4135013	3.674875	2.6051412	4.9839172
9833.96	4.6776676	3.4246407	6.2393532	4.6735454	3.5305545	6.0959611
8190.3379	2.0756164	1.2091229	3.3232629	2.1037173	1.3979321	3.1373904
24230.064	3.2191412	2.5445976	4.0176344	3.1100874	2.4580107	3.8708427
28898.021	2.4223113	1.8883103	3.0604446	2.3960462	1.8579851	3.0269103
23924.457	2.3407009	1.7681407	3.0395958	2.3924484	1.8544319	2.9988647
10295.625	6.1191039	4.7020874	7.8289919	6.1015205	4.8004227	7.735239
23201.457	2.4998431	1.8982357	3.2316279	2.4974985	1.9557282	3.1513941
17900.771	2.1786771	1.549251	2.9783218	2.1710465	1.6135201	2.8165476
18752.34	2.8796408	2.1632762	3.7573051	2.8555534	2.2689333	3.5730848
6982.8745	4.1530175	2.7813451	5.9644227	4.1369052	3.0269904	5.5621157
9983.3486	3.6060045	2.5255983	4.9922304	3.6071036	2.5978715	4.846755
8321.2266	3.244714	2.1382868	4.720891	3.2363589	2.2921779	4.425137
23935.678	2.7573903	2.1325653	3.5080762	2.7458687	2.1268499	3.4519932
29092.457	2.2686293	1.7545577	2.8862526	2.264348	1.7605571	2.9021127
24151.604	2.4843071	1.8957881	3.1977952	2.5418329	2.0119846	3.1984587
10365.684	6.3671632	4.9243636	8.1005926	6.3276019	4.9607668	7.9385376
23416.367	3.0747724	2.4058201	3.8721673	3.0817451	2.4815438	3.8107774
18155.818	2.4234655	1.7608936	3.2533891	2.3902087	1.8014584	3.0634036
19395.371	2.1139066	1.5169758	2.8677528	1.9753325	1.5107785	2.5567868
6940.2163	5.1871581	3.6330178	7.1812139	5.1671948	3.8836057	6.7055573
10063.965	5.6637721	4.2896857	7.3380752	5.663413	4.4009929	7.2255635
8423.9453	4.9857874	3.593317	6.739336	5.026834	3.845618	6.5081849
24033.787	3.3702552	2.6764696	4.1889195	3.3338788	2.680238	4.148622
29190.52	2.7406158	2.1731391	3.4109337	2.7534781	2.1848991	3.4407496
24218.217	3.220716	2.5458422	4.0195999	3.2716432	2.6487494	3.9902799
10373.708	9.7361517	7.9302549	11.83031	9.694581	7.971735	11.654906
23420.041	3.159687	2.4810348	3.9666977	3.1655166	2.5477369	3.8927338
18245.199	2.3567843	1.7056165	3.1745741	2.3515024	1.8014576	3.0634027
19077.463	2.46364	1.81019	3.276119	2.481648	1.9294469	3.1433296
6772.126	3.9869311	2.6274128	5.8007784	3.9858828	2.8576586	5.3314285
9887.2139	5.0570364	3.7534297	6.6670713	5.0571771	3.845618	6.5081863
8299.5947	3.3736587	2.2417717	4.8758798	3.3974934	2.4446425	4.6363287
23555.902	3.3537242	2.6551752	4.1797414	3.44965	2.7545438	4.2409849
28740.643	3.061866	2.4557061	3.7723045	3.079005	2.481544	3.8107772
23780.635	2.6071634	1.9988972	3.3422654	2.6423945	2.075212	3.2780905
10128.753	8.4906797	6.7914481	10.485921	8.4830351	6.9055972	10.357389
22945.736	2.2662163	1.6925187	2.9718406	2.2683396	1.7605568	2.902113
17972.586	3.7278998	2.8890717	4.7343011	3.6718485	2.9519558	4.5216398
17796.107	2.1352985	1.5110639	2.9308641	1.9495221	1.4797689	2.5163667
6828.2192	5.8580427	4.1850719	7.9769912	5.8175807	4.4913535	7.4978428
10051.493	4.4769468	3.2655153	5.9905066	4.4762063	3.3737259	5.8891449
8461.9004	4.7270708	3.3770885	6.4369283	4.754415	3.609149	6.1991882
23931.195	3.9697139	3.2117355	4.8527699	3.9018161	3.1650646	4.7471371
29392.932	2.2454379	1.7366215	2.8567474	2.2553332	1.7281548	2.86044
24255.619	2.3087432	1.7440002	2.9980962	2.3446488	1.8230139	2.9588513
10212.238	9.9880161	8.1440315	12.12477	9.9917469	8.2187834	11.95331
23224.488	2.626538	2.0090933	3.3738976	2.6185718	2.0537627	3.2755852

18324.543	4.4748731	3.5589995	5.5544987	4.487761	3.6945889	5.4311938
20327.563	2.4597147	1.8256475	3.2428269	2.4682152	1.9488726	3.118711
6866.1055	3.6410744	2.3563116	5.3749442	3.6396363	2.6051397	4.9839201
10246.272	5.2702093	3.9591463	6.8764777	5.2701569	4.0038028	6.7136464
8660.4951	3.5794718	2.4320772	5.0807734	3.5937939	2.5978715	4.8467531
24255.93	3.4218438	2.7254794	4.24189	3.4268723	2.7545445	4.240984
29985	2.5679507	2.0265865	3.2094967	2.5811236	2.0210531	3.2342184
24704.516	3.1573176	2.4957285	3.9404757	3.2321534	2.6166973	3.9509029
10292.282	8.5500965	6.8574271	10.533957	8.5169306	6.9055958	10.35739
23438.689	3.3704958	2.6684532	4.2006435	3.3746142	2.7468512	4.1380682
18696.375	3.2091782	2.448941	4.1308475	3.2089169	2.5263438	3.9907198
17933.963	3.2898474	2.5043843	4.2436619	3.0607393	2.4398086	3.7868297
6894.4585	5.511673	3.9003868	7.5652018	5.4882007	4.1433535	7.0458283
10291.762	6.5100613	5.0452089	8.2675476	6.5094261	5.1214137	8.1415319
8934.7979	5.0364876	3.6736484	6.7392168	5.0681257	3.8456182	6.5081868
24815.285	4.4327517	3.6431828	5.3426652	4.5613914	3.7669425	5.4786029
30141.275	2.8864074	2.3118939	3.5603726	2.9165595	2.3164835	3.6054647
24814.625	2.6194229	2.021615	3.3386695	2.6233327	2.0752113	3.2780912
10275.948	10.996552	9.0627155	13.220891	10.920571	9.1275082	13.044598
23414.564	3.630219	2.8996904	4.488822	3.6619537	2.9800856	4.4233561
18838.063	3.7158813	2.8967116	4.6947923	3.689188	2.9519558	4.5216389
20269.395	2.1707606	1.5772779	2.9141448	2.0984061	1.6040548	2.6778018
6754.4175	4.7376404	3.2405424	6.688138	4.7402334	3.5391409	6.2500033
10087.827	6.443409	4.9728866	8.2126532	6.4403014	5.0410538	8.0400715
8824.498	2.4930596	1.562384	3.7745223	2.5048687	1.691435	3.5712368
24548.213	3.3403652	2.6566918	4.146275	3.3197913	2.643126	4.1023974
29578.664	3.6512806	2.9952164	4.4083371	3.6757123	3.0134821	4.4640346
24372.502	3.4465072	2.7490685	4.2670102	3.4861343	2.8414688	4.2261348
10037.974	13.947038	11.732504	16.458052	13.920674	11.876185	16.295944
22864.934	3.3676021	2.6576588	4.2089238	3.3869982	2.746851	4.1380682
18537.914	4.6391411	3.7107141	5.7293015	4.634357	3.8125689	5.5740852
19438.543	2.8294301	2.1315148	3.6828938	2.7409911	2.1702571	3.3973331
284006.75	6.778008	6.4785709	7.0877137	7.0899377	6.5776544	7.6212869
284818.09	6.1232066	5.8391681	6.4174891	6.401804	5.923315	6.9156256
286301.63	4.9248762	4.6711407	5.1888118	5.1892381	4.7530351	5.645905
290216.5	4.6103511	4.3665981	4.8641686	4.8332057	4.4183559	5.2805839
293490.22	4.9235029	4.6728888	5.1840663	5.1314507	4.7051911	5.593749
294695.69	4.8490701	4.6008849	5.1071644	5.0073099	4.5808477	5.4580927
297756.06	4.4163666	4.1808643	4.6616797	4.5477428	4.1414762	4.9774642
300858.84	4.1182103	3.8920655	4.3540673	4.2470493	3.8554964	4.6634436
305857.09	4.4105563	4.1783066	4.6523561	4.5106382	4.1128564	4.9460831
309519.22	4.1612926	3.9371104	4.3949137	4.2375789	3.8459721	4.6529679
314441.56	4.2742443	4.0487599	4.5090179	4.3596888	3.9602997	4.7786403
319471.69	3.8688872	3.6561797	4.0907416	3.9516892	3.5795283	4.3594117
325718.63	3.7762654	3.5681515	3.9933507	3.8360441	3.4654846	4.2334552
330106.47	4.0350618	3.8212514	4.2577209	4.0854096	3.7031767	4.4957628
334878.88	4.0014467	3.7900431	4.2215734	4.0512767	3.6746333	4.4643068
340054.5	4.228734	4.0129681	4.4530864	4.2315197	3.8459721	4.6529679
84781.656	4.505692	4.0651321	4.9809771	4.4278736	4.0270271	4.851913
346737.16	3.1897359	3.0044963	3.3834074	3.1586611	2.8211553	3.5177834
85888.328	1.7813829	1.5103004	2.087075	1.7315506	1.491061	2.0078745
87282.352	2.6809545	2.348429	3.0473669	2.6672752	2.359313	2.9996245
87397.563	3.2266345	2.8609927	3.6260557	3.2197824	2.8778622	3.5810766
85659.141	2.9769154	2.6227152	3.3656137	2.8654881	2.5475316	3.2114067
349313.53	3.4295838	3.2381041	3.6294301	3.3870065	3.0386951	3.7602437
86346.992	3.3122172	2.9394314	3.7191875	3.195473	2.8589563	3.5599823
87823.695	2.9946361	2.6436172	3.3792927	2.9765849	2.6512265	3.3277118
87299.422	3.9175518	3.5133123	4.355547	3.8754075	3.5034888	4.2754507
84335.805	3.853642	3.4460182	4.2962236	3.7461479	3.380013	4.1389265
353075.59	4.1662464	3.9560363	4.3847265	4.1686869	3.7793181	4.5796218
87862.914	3.9493341	3.544682	4.3875213	3.9458029	3.5700212	4.3489189
86846.922	3.972507	3.5643346	4.414609	3.9141684	3.5415034	4.3174362
89860.766	4.2955346	3.8776495	4.7461791	4.2791433	3.8840725	4.6948671

87372.648	4.703989	4.2601528	5.1815009	4.6113467	4.2082715	5.0506682
286045.25	5.8592129	5.582027	6.1465998	6.6093707	6.1156325	7.1233087
287155.31	5.0913215	4.8336563	5.3591542	5.6759338	5.2224293	6.1565118
287965.72	4.1949439	3.9616904	4.4383459	4.567091	4.1605582	4.9983821
292753	3.9692166	3.744251	4.2041664	4.3176665	3.9221818	4.7367582
297941.28	4.1216173	3.8942885	4.3587542	4.5473418	4.1414762	4.9774642
300134.28	4.1381478	3.9111791	4.3748522	4.4315929	4.036562	4.8623786
302308	3.6552126	3.4428465	3.87725	3.8898354	3.5129917	4.2859478
305499.63	3.7217722	3.5085585	3.9445546	3.9151964	3.5415034	4.3174362
309818.31	3.9216533	3.7042158	4.1485233	4.0467033	3.6651201	4.4538198
310610.91	3.6057973	3.3976891	3.8233178	3.7046149	3.3420432	4.0968962
315098.69	3.7194695	3.5095472	3.9386678	3.8293166	3.4559851	4.2229548
318606.47	3.3332658	3.135782	3.5399277	3.3576086	3.0102956	3.7286427
323858.28	3.2298076	3.0370178	3.4316275	3.2508359	2.9156837	3.6232553
326474.13	3.1396055	2.9503195	3.3378503	3.1170361	2.7833679	3.4755707
330924.47	3.2998466	3.1070063	3.5015223	3.3031023	2.9629796	3.6759591
337522.34	3.3508892	3.1584218	3.5520177	3.3576696	3.0102956	3.7286427
84068.742	2.8904917	2.5384591	3.2776928	2.852675	2.5381107	3.2008274
344087.06	2.3743989	2.2143552	2.5429535	2.3313434	2.0497644	2.6491733
84913.258	1.6134113	1.3545651	1.907316	1.5664735	1.3340214	1.8249129
86293.766	2.1786046	1.8783008	2.5132678	2.1001036	1.8348854	2.4040511
86104.945	2.5666354	2.239377	2.9282651	2.4653833	2.1715455	2.7873919
83278.898	2.2694826	1.9574496	2.6171165	2.18139	1.9095364	2.4894004
345735.53	2.8663528	2.6906478	3.0505188	2.8495464	2.5286908	3.1902473
85917.266	2.572242	2.2442689	2.9346619	2.5680289	2.2653699	2.8935678
85976.734	2.8379772	2.493026	3.2173145	2.8029306	2.4910219	3.1479163
86687.641	3.4260938	3.047487	3.8387387	3.3840368	3.0386951	3.7602439
85543.492	2.9458699	2.5933504	3.3329387	2.9175489	2.594651	3.2642875
350370.41	3.4791751	3.2865832	3.6801076	3.4708662	3.1239352	3.8550038
87147.273	3.3277001	2.9556863	3.7335799	3.3825929	3.0386951	3.7602439
88232.703	3.2754295	2.9086452	3.6756642	3.2310369	2.8967712	3.6021674
88829.406	3.8050461	3.4101632	4.2331071	3.7860026	3.4179938	4.1809459
87672.836	3.8780539	3.4767473	4.3129745	3.8910983	3.522495	4.2964449
154994.13	5.1485825	4.797513	5.518548	4.857687	4.3244181	5.4404788
156963.41	4.6507654	4.3194661	5.0007343	4.3484554	3.8382223	4.8933406
159153.89	5.1082635	4.7631154	5.4718118	4.8426466	4.3087082	5.4228554
161828.84	4.5912704	4.2670302	4.9336162	4.3338008	3.8382223	4.8933406
164897.69	4.6149831	4.2928758	4.9548564	4.3520403	3.853879	4.9110179
166019.67	4.391046	4.0780373	4.7217059	4.1796799	3.6817677	4.7164617
167231.42	5.0349388	4.7005539	5.3868327	4.7617979	4.2301846	5.3347125
169416.5	4.7043824	4.3834043	5.0426478	4.4215555	3.9165232	4.9817066
172140.44	5.4606576	5.1171083	5.8212047	5.1589899	4.6074662	5.7574315
173648.69	4.8488708	4.5268426	5.1877599	4.5438256	4.0262232	5.105341
175603.7	5.1080928	4.7792382	5.4536142	4.8166847	4.2930007	5.40523
177523.2	5.2894492	4.9564977	5.6388845	4.9123306	4.3715568	5.49334
180129.69	5.2462206	4.9170227	5.5916624	4.8574905	4.3244181	5.4404788
181486.17	5.0747666	4.7522726	5.4133854	4.6423278	4.1203222	5.2112412
183495.86	5.3298202	5.000977	5.6746068	4.8573833	4.3244181	5.4404788
185893.16	5.1857743	4.8635406	5.5237465	4.7260141	4.1987867	5.2994437
45902.223	4.4224439	3.8349707	5.0744524	4.0080752	3.5255189	4.5393772
189222.31	3.2448604	2.9932408	3.5119834	2.9353428	2.5314009	3.4001579
46911.305	1.8971972	1.5236057	2.3346646	1.7155125	1.4011985	2.0636842
47692.848	3.8370535	3.3012412	4.4350543	3.5352099	3.0892313	4.0423298
47928.48	2.7958324	2.3425164	3.3112793	2.5285807	2.1466095	2.9516139
47005.969	2.5741413	2.1359541	3.0757701	2.3233879	1.9628428	2.7353792
191206.61	3.2425659	2.9923193	3.5081539	2.9116507	2.5005314	3.364361
47624.555	3.1916311	2.7044153	3.7412684	2.8977575	2.485101	3.3464572
48244.434	3.5237226	3.0139253	4.0950522	3.219008	2.7943332	3.7038939
48408.781	3.5324168	3.022799	4.1033535	3.2032273	2.7788413	3.6860523
47551.957	4.3741627	3.7998798	5.010725	3.9677005	3.4942946	4.5039344
194116.08	3.9873049	3.7113123	4.2783885	3.59552	3.1358843	4.0956769
48309.027	4.0572128	3.5090663	4.6667056	3.7092557	3.2448308	4.2200642
49062.32	3.6891856	3.1712921	4.26754	3.3897576	2.9494159	3.8821445

49262.078	3.7554243	3.2337456	4.3373027	3.4234605	2.9804668	3.9177604
48359.984	4.4044681	3.8327942	5.037365	4.0330296	3.5411339	4.5570951
142189.27	5.7810268	5.392529	6.1901188	6.2686009	5.6650033	6.933229
144821.42	5.5309496	5.1545019	5.9276199	5.9784322	5.3803334	6.6178985
147075.25	4.9634457	4.6098723	5.3369431	5.3393149	4.7806811	5.9508839
150025.17	4.6058941	4.2688322	4.9624949	4.955296	4.4187107	5.5461864
153862.06	4.4390411	4.112329	4.7848067	4.765799	4.2301846	5.3347125
155600.72	4.2094922	3.89325	4.54458	4.3917689	3.8851967	4.9463663
157006.41	5.2927775	4.9389896	5.6652155	5.3490882	4.7806816	5.9508834
159353.64	4.9135995	4.5754161	5.2701645	5.1465616	4.5917282	5.7398362
162218.66	6.1152029	5.7405324	6.5079064	6.3627315	5.744144	7.0207558
164099.89	5.2163348	4.8726988	5.5778117	5.3670459	4.8121939	5.9860373
166567.5	4.8508863	4.5221334	5.1972208	4.9086442	4.3715577	5.49334
169083.94	5.642168	5.2897716	6.0118685	5.7291799	5.1434045	6.3548269
171901.14	5.3577304	5.0172544	5.7152305	5.3316903	4.7649274	5.9333038
173923.98	5.3644128	5.0256758	5.7199745	5.2645741	4.7019253	5.8629727
176181.58	5.0913386	4.7635627	5.4357266	4.9735537	4.434432	5.5637989
179136.05	5.2139144	4.8848538	5.55931	5.058763	4.5130606	5.6518373
45172.543	5.1801381	4.5376329	5.8881197	5.160748	4.6074662	5.7574315
183117.73	3.3967216	3.1349916	3.6744709	3.2969954	2.8563311	3.7752287
45022.789	2.2210975	1.8071735	2.7014499	2.1378098	1.7950138	2.5365403
46190.789	3.0525565	2.5695133	3.6000235	3.0079503	2.5931823	3.4717104
46428.949	3.0584369	2.5760958	3.6048648	2.9524584	2.5468411	3.4180512
45543.434	2.6128905	2.1645629	3.1267126	2.5349455	2.161953	2.969604
185135.28	3.6513841	3.3812823	3.9373219	3.4974122	3.0426018	3.9889591
46131.652	3.6417513	3.1118755	4.2359867	3.5838335	3.1358843	4.0956769
46691.516	4.0906792	3.531091	4.713757	3.8582833	3.3850811	4.3798141
46447.43	4.0906463	3.5296469	4.7154732	3.9901392	3.5099058	4.521657
46084.605	3.8190627	3.275667	4.4268465	3.6253014	3.1669993	4.1312289
188651.92	4.1345987	3.849494	4.4352307	3.9584255	3.4786863	4.4862099
46945.91	3.9407053	3.3932889	4.5512919	3.8541884	3.3850811	4.3798146
47737.309	4.0848556	3.5316129	4.7001824	3.9745402	3.4942949	4.5039344
47954.512	4.6293874	4.0404034	5.2800841	4.5180554	4.0105457	5.0876842
47059.176	4.5474658	3.9585643	5.1992798	4.4425144	3.932189	4.9993744
155616.47	0.32772878	0.2440156	0.43090281	0.32819945	0.20128153	0.5135594
162499.83	0.35692343	0.2710269	0.46140647	0.38176885	0.24527051	0.5827996
164992.16	0.41820171	0.3253858	0.52926105	0.43617797	0.2901369	0.6511563
146490.58	0.51197833	0.4027033	0.64176929	0.45451921	0.30525765	0.6737754
171288.52	0.45537207	0.3599527	0.56832498	0.47424719	0.32045376	0.6963196
172583.42	0.41718957	0.3264251	0.52538127	0.43866894	0.29013684	0.6511565
174006.63	0.54020929	0.4365441	0.66107941	0.56131452	0.38190323	0.7858272
176499.39	0.4702566	0.3745568	0.58295375	0.49536973	0.33572003	0.7187932
154652.58	0.51728851	0.4101778	0.64381033	0.46763408	0.30525815	0.6737757
181412.05	0.50162047	0.4038735	0.61587882	0.52424467	0.3510533	0.7411999
183707.64	0.41370082	0.3259492	0.51780826	0.43688527	0.29013684	0.6511565
185959.73	0.56463838	0.4618184	0.6835292	0.59196436	0.41298026	0.8302271
188930.45	0.52400231	0.4258832	0.63795406	0.54832602	0.38190278	0.7858267
163242.59	0.6554662	0.5371705	0.79206383	0.57337952	0.39741501	0.8080543
192843.19	0.62745279	0.5206437	0.74972594	0.64853567	0.459977	0.8964452
195539.78	0.73642302	0.6210573	0.86700267	0.74165094	0.53918087	1.0059298
41676.5	0.83980185	0.5849528	1.1679611	0.72664553	0.52326059	0.9841126
198997.28	0.61307371	0.5091206	0.73201084	0.61175686	0.42859817	0.8523482
49337.664	0.54724926	0.3606407	0.79621935	0.56291628	0.3819032	0.7858272
43500.352	0.68964964	0.4653035	0.9845174	0.60830718	0.42859778	0.8523483
50380.063	0.5557754	0.3693087	0.80325079	0.55899131	0.38190326	0.7858273
43693.258	0.59505749	0.3887113	0.87189734	0.54217643	0.36644804	0.7635435
200857.56	0.54765177	0.4501032	0.66006857	0.55158746	0.38190347	0.7858266
50032.414	0.61959833	0.420987	0.87947011	0.63269848	0.44426414	0.8744202
36340.34	0.6604231	0.4231457	0.98265725	0.46947193	0.30525815	0.6737757
37949.406	0.76417536	0.511781	1.0974827	0.58471036	0.39741498	0.8080541
35977.59	0.97282785	0.6776103	1.3529682	0.72124225	0.52326059	0.9841124
203758.42	0.71162701	0.6005143	0.83733869	0.70941216	0.50737798	0.9622571
44354.09	1.0596542	0.778594	1.4091154	0.93515772	0.70020688	1.2222754

30575.865	0.91575491	0.6085125	1.3235219	0.55508697	0.38190311	0.7858275
37485.652	0.88033676	0.6059833	1.2363206	0.65029711	0.45997703	0.8964452
36161.109	0.7466585	0.4920527	1.0863495	0.53182691	0.36644816	0.7635428
146069.81	0.7188344	0.5879355	0.87019289	0.89709914	0.66776246	1.1792459
149942.2	0.78030068	0.6453299	0.9351697	0.96175987	0.71646887	1.2437502
152357.48	0.81387532	0.6769394	0.97037768	1.0111511	0.76540506	1.3080243
155503.41	0.79097944	0.6573812	0.9437505	1.0066196	0.765405	1.3080243
159504.77	0.82129204	0.6866806	0.9745788	1.0563365	0.79814726	1.3507557
161329.58	0.83679634	0.7015992	0.99044973	1.0800689	0.83097804	1.3933979
162876.11	1.0498778	0.8984131	1.2195671	1.3365607	1.0462564	1.6686941
165472.78	0.82793069	0.6951025	0.97874951	1.0561514	0.79814726	1.3507557
168695.64	1.0136598	0.8674203	1.1774955	1.282637	0.99631405	1.6054273
170854.89	0.91305548	0.7753979	1.0681069	1.1345862	0.88038027	1.4572053
173546.42	0.91618139	0.7793081	1.0701745	1.165866	0.8968876	1.4784349
146809.63	1.1443391	0.9778375	1.3310643	1.2220417	0.9465214	1.5420108
179441.25	0.79691821	0.6716587	0.93875813	0.9882713	0.74906874	1.286624
181690.5	1.0952692	0.9483725	1.258474	1.324221	1.0462564	1.6686943
184154.33	1.0100224	0.8700803	1.1660672	1.2066704	0.9299584	1.5208372
187378.63	1.0940416	0.9493954	1.2544954	1.3192408	1.0295931	1.6476212
40700.211	0.98279589	0.7021238	1.338289	1.0370088	0.78176492	1.3294015
191466.98	0.78342485	0.6630707	0.91930819	0.94556069	0.71646899	1.2437502
40891.805	0.73364329	0.4949858	1.0473211	0.76909119	0.55513787	1.0277103
41059.738	0.85241657	0.5937394	1.1855052	0.91279769	0.68397105	1.2007742
41323.34	1.0647736	0.773666	1.4294088	1.1145155	0.8638925	1.4359562
40108.836	1.0970151	0.7970928	1.4726915	1.1072868	0.84742492	1.4146876
193331.22	0.99311435	0.8576015	1.1439598	1.2093378	0.94652128	1.5420107
34059.602	1.5854559	1.1910442	2.0686755	1.4070395	1.1130614	1.7528344
33913.75	1.2679223	0.9176016	1.7078836	1.0990628	0.84742516	1.4146874
41883.172	1.169921	0.865514	1.5466975	1.3073928	1.029593	1.6476212
40757.672	1.3739744	1.0378857	1.7842206	1.3504783	1.062935	1.6897521
196798.64	1.0721619	0.9323669	1.227003	1.261292	0.97969931	1.5843053
41178.844	1.2385	0.9221446	1.6283987	1.2563958	0.97969961	1.5843054
42660.316	1.3126954	0.9915962	1.7046447	1.3911275	1.0963379	1.7318215
41933.609	1.0969721	0.8031214	1.463207	1.1048592	0.84742492	1.4146876
49066.191	0.8559866	0.6169198	1.1570451	1.0338778	0.78176481	1.3294015
1602139.4	12.276086	12.105113	12.448869	12.16396	11.686373	12.653099
2244672.3	7.7797551	7.6647911	7.8960118	7.8382792	7.4567041	8.2327671
2444488	5.7128525	5.6184902	5.8084021	5.8425922	5.5146985	6.1847725
2523446.8	4.8358464	4.750422	4.922421	4.9600844	4.6609516	5.2785192
2571097.5	4.7018833	4.6184368	4.786459	4.7889142	4.4914432	5.0980277
2581583.8	4.7219076	4.6384521	4.8064876	4.7971163	4.5011268	5.1083441
2587148.8	4.4121928	4.3316193	4.4938889	4.4699664	4.1817513	4.7677197
2586714	4.3680902	4.2879152	4.4493871	4.4294887	4.1430655	4.7264056
2584800.8	4.7802525	4.6963325	4.8652954	4.8176622	4.5204949	5.1289759
2556182	4.217227	4.1379886	4.2976012	4.2298231	3.9497266	4.5197439
2517464	4.4326353	4.3507695	4.5156546	4.4373074	4.1527362	4.7367344
2466613.8	6.2944593	6.1958346	6.3942609	6.2778111	5.9374533	6.6320176
2424514	4.7159963	4.6299462	4.8032441	4.6913319	4.3994665	5.0000043
2366502.8	4.7183552	4.6312394	4.8066978	4.6658697	4.375267	4.9742036
2291198.8	4.660006	4.5720286	4.7492504	4.5847545	4.2930055	4.8864651
2165801.5	5.163908	5.0686426	5.2605143	5.0798998	4.7723956	5.3970752
1086042.3	7.8265834	7.6610732	7.9947681	7.8568845	7.3647127	8.3677387
1417265	6.7792544	6.6443696	6.9161882	7.2203078	6.752017	7.7137675
1524611.9	5.8578844	5.7370176	5.9806566	6.34794	5.9070816	6.8087029
1571658.4	5.3300385	5.2165036	5.4454222	5.7383351	5.3208904	6.1782269
1604239.6	4.6776052	4.5723629	4.7846589	5.0137	4.6238747	5.4252424
1615053.1	5.3366666	5.2245893	5.4505429	5.6929326	5.2807827	6.1350017
1622592.8	4.8028069	4.6967592	4.9106455	5.0747976	4.6798849	5.485899
1626957.4	4.5047278	4.4021788	4.6090622	4.7027998	4.328054	5.1043968
1631323.8	4.3762007	4.2752686	4.4789143	4.5072198	4.1363902	4.8960605
1621918.4	4.1765356	4.0776625	4.2772007	4.2667403	3.9130199	4.6527638
1612441.9	4.0751858	3.9772418	4.174932	4.1215777	3.7695692	4.4962144
1597437	4.2511849	4.1506696	4.353519	4.2617359	3.9050474	4.6440697

1589060.9	4.4932199	4.389596	4.5986729	4.4547601	4.0885029	4.8439474
1569243.9	4.4524627	4.3486671	4.5581102	4.3712106	4.008718	4.7570662
1542766.8	4.4711876	4.3662891	4.57797	4.3248234	3.9608626	4.7049208
1497563	4.5340333	4.4268227	4.6431842	4.3233843	3.9608626	4.7049212
987604.06	0.80801612	0.7529195	0.86607832	0.82686836	0.66583556	1.0123979
1270773.1	0.58625728	0.5449098	0.62991041	0.62184513	0.4815509	0.7815834
1366262.1	0.53576833	0.4976538	0.57602763	0.59272289	0.45670685	0.7498228
1413598.9	0.52136433	0.4843983	0.56040329	0.56769842	0.44019011	0.7286027
1449880.8	0.51659423	0.4802556	0.55495363	0.56923038	0.44019026	0.7286028
1469701.1	0.47832856	0.4436186	0.51503283	0.51976424	0.39907342	0.6753782
1487357.1	0.52240312	0.4863121	0.56046355	0.57328039	0.44019026	0.7286028
1503216.6	0.53684878	0.5004435	0.57520235	0.57152557	0.44019026	0.7286028
1517760.5	0.54751718	0.5109192	0.58604443	0.58393228	0.4484438	0.7392176
1516773.6	0.54523629	0.5087043	0.58369887	0.57263798	0.44019026	0.7286028
1512513.9	0.56858987	0.5312187	0.60789663	0.59138519	0.45670688	0.7498227
1499669.6	0.63480651	0.595117	0.67644697	0.65355915	0.5147965	0.8238106
1492299.8	0.64799315	0.6077896	0.69015712	0.65268862	0.5147965	0.8238108
1473978.9	0.71846348	0.6758378	0.76307303	0.71632183	0.56489956	0.8869165
1450528.4	0.74386686	0.7001388	0.78961074	0.7250905	0.57327545	0.8974088
1415131.5	0.81617862	0.7697817	0.86464113	0.77724028	0.62367016	0.960223
254060.64				6.9983292	6.6767788	7.3313627
290405.84				6.5976634	6.3055182	6.8998523
298868.34				6.7420988	6.4509025	7.0430522
234981.55				8.7113218	8.3379936	9.0970592
300163.06				6.8362842	6.5436649	7.138618
284779.41				7.2687836	6.958993	7.5888133
264381.38				7.916594	7.5810328	8.2631836
272213.97				7.7071724	7.3808732	8.0441828
298355.78				7.1324244	6.8325715	7.4420485
251606.72				8.4934139	8.1370888	8.8613262
301592.47				7.0857205	6.7884526	7.3926554
301808.88				7.1701007	6.8711553	7.4787059
295494.53				7.8004832	7.4852557	8.1255741
230212.34				10.155842	9.7483082	10.576035
290781.44				8.0404034	7.7177582	8.3730717
262706.63				9.1166334	8.7551374	9.4892235
294686.88				8.4021387	8.0744143	8.7397509
286435.97				8.6720943	8.3343801	9.0199823
271209.78				9.2142687	8.8565092	9.5827732
282171.5				8.9201069	8.5749969	9.2755442
294130.66				8.6390181	8.3063498	8.9815922
278405.59				9.1485233	8.7966461	9.5108662
292707.38				8.7391033	8.4036903	9.0844707
297325.53				8.6100912	8.2797575	8.9502249
296757.84				9.0949574	8.7550392	9.4446926
265081.97				10.223253	9.8419342	10.615561
285686.19				9.8044643	9.4447002	10.174424
290689.66				9.8214712	9.4644756	10.188486
277017.72				10.400057	10.023725	10.786902
172727.81				17.130999	16.519255	17.759604
258489.03				12.101093	11.680697	12.532754
164284.72				21.170563	20.472761	21.886082
975650.5				2.6792381	2.5775037	2.7839587
898021.56				2.9654076	2.853838	3.0802214
960411.94				2.8185823	2.7133939	2.9268038
922708.19				3.0182891	2.9072216	3.1325135
812544.31				3.4644263	3.3376164	3.5948207
983247.81				2.8955059	2.7901125	3.0038619
865395.44				3.329114	3.208648	3.4529455
980396.06				3.0263278	2.9184029	3.1372235
985547				3.1708281	3.0606201	3.2839906
965008.56				3.2984164	3.184814	3.415036
974191.88				3.3145421	3.2011933	3.4308794

936899.25				3.5115836	3.3926053	3.6336689
894612.38				4.1716394	4.0388618	4.3076701
1003689.8				3.7521555	3.6332655	3.873945
1012403.1				3.7198622	3.6019955	3.8406034
990101.19				3.9036415	3.7815332	4.0286884
920135.94				4.217855	4.0861883	4.352684
1011902.8				3.8738897	3.7535586	3.9970963
888063.38				4.4861665	4.3479319	4.6276774
941393.13				4.264956	4.1340418	4.3989606
958382.19				4.2279582	4.0987697	4.3601832
1013069.5				4.0184803	3.895977	4.143856
1009041.6				4.088038	3.964226	4.2147331
1006954.5				4.1531172	4.0281882	4.2809353
993548.31				4.3852925	4.2560358	4.517477
940226.5				4.7275844	4.5896149	4.8686481
797613.63				6.1483402	5.9774508	6.3228765
973408.13				5.2331595	5.0904264	5.37888
545771				10.277204	10.009988	10.549747
970963.94				5.8220491	5.6712575	5.9758353
923487.56				7.2778459	7.1048799	7.453959
719355.81				10.595591	10.35904	10.83618
83975.383	30.794739	29.619169	32.005005	30.697168	27.36154	34.227848
74516.086	16.103907	15.205536	17.041496	16.104006	13.891861	18.643915
91069.758	9.212718	8.5998001	9.8577871	9.1032391	7.4787436	11.056979
179212.78	7.7896233	7.3863115	8.2092304	7.676764	6.6375427	8.8580227
208495.39	8.5613403	8.1687374	8.9679356	8.4665709	7.4184656	9.6146345
211439.66	9.0002041	8.6003304	9.4138727	8.918829	7.8559999	10.068777
87451.328	20.720097	19.776947	21.696608	20.743425	18.208961	23.599558
224590.23	10.27204	9.8571129	10.699946	10.159282	9.0168495	11.423661
192175.36	14.11211	13.585933	14.653446	14.113645	12.63577	15.707442
249213.42	12.551491	12.115448	12.999218	12.817203	11.529286	14.181211
106417.14	20.156528	19.31246	21.02799	20.104567	17.510258	23.079102
126305.63	9.7303658	9.1939001	10.289968	9.7336464	8.0540447	11.754415
149466.44	5.1048245	4.7489877	5.4802623	5.05091	3.845618	6.5081859
277982.19	4.8204527	4.5657797	5.0856342	4.7424383	3.9181643	5.6607146
301745.16	5.5079594	5.2463088	5.7792811	5.4544377	4.633956	6.3991323
315281.5	5.8550849	5.5910068	6.1284151	5.8115926	4.9620371	6.7484436
141381.8	12.031252	11.466229	12.616914	12.035975	10.123466	14.230469
300789.94	6.6225624	6.3349004	6.9199195	6.5341487	5.6253357	7.5559068
239152.84	9.4416609	9.0562019	9.8393087	9.4616938	8.2617369	10.777119
286149.56	8.8310461	8.4900513	9.1822224	9.0618038	7.9900351	10.220457
107823.45	19.578302	18.751942	20.431702	19.51519	16.951958	22.437407
145478.3	6.8257604	6.4077625	7.2638664	6.8291984	5.4435778	8.5466499
169702.67	3.5709515	3.2922609	3.8669305	3.548399	2.5978715	4.8467517
314767.16	3.1864824	2.9923074	3.3899505	3.1659338	2.4949729	3.9172146
326029.31	3.6131721	3.4097617	3.8255472	3.5881243	2.9133506	4.3419418
350565.69	4.0933843	3.8843093	4.3107886	4.0901318	3.3908677	4.8910284
153463.19	9.3116789	8.8350887	9.8072977	9.317934	7.6429052	11.256459
320923.53	4.8329268	4.5953712	5.0795789	4.8097777	4.0227652	5.6769853
259240.83	6.7080483	6.3964391	7.0309129	6.7162385	5.7199631	7.8406205
296493.97	6.4756799	6.1892314	6.7719655	6.6068845	5.6893225	7.5925899
110208.28	16.559555	15.808444	17.337133	16.493671	14.078392	19.110956
151852.53	5.6765599	5.3038874	6.0685115	5.6795106	4.4009962	7.225564
176551.8	3.1888659	2.9308622	3.4634957	3.1736414	2.2162533	4.3192353
334093.41	2.5980759	2.4280896	2.776824	2.5863268	2.017122	3.3117168
329633.75	3.2096229	3.0191107	3.4090061	3.1983299	2.5808678	3.9336777
365593.59	3.4327734	3.2454569	3.628082	3.4406075	2.8093019	4.186873
154386.41	8.4592934	8.0066729	8.9308367	8.4687948	6.9055967	10.357389
323501.28	4.1081753	3.8902485	4.3351316	4.0988817	3.3819752	4.9103622
275571.5	5.523068	5.2490473	5.807682	5.5682025	4.6826448	6.6170616
302054.06	5.346725	5.0891094	5.6140027	5.4655914	4.6656442	6.401978
112540.3	16.474098	15.732664	17.241451	16.410498	14.078389	19.110956
155373.05	6.2880917	5.899929	6.6950831	6.2922277	4.9607663	7.938539

177808.58	3.4925201	3.223196	3.7783415	3.469954	2.5211647	4.741632
346882.91	2.6435432	2.4751899	2.8203332	2.6338363	2.0536582	3.358515
328061.41	3.2920666	3.0986314	3.4944148	3.2929173	2.6471965	4.0154986
375763.78	3.5873601	3.3983891	3.7841046	3.6023228	2.9380765	4.3438144
153412.47	8.2913733	7.8419285	8.7598619	8.2977066	6.7422714	10.157076
326651.78	3.9246686	3.7127466	4.1455345	3.9250908	3.2142272	4.7077379
287096.22	4.9391108	4.6853514	5.203042	4.9415212	4.0885191	5.9068322
307506.97	4.2925858	4.0641122	4.5305591	4.3714547	3.6508937	5.2024345
115054.88	15.722931	15.006658	16.46456	15.644818	13.340215	18.249128
157479.53	6.5405326	6.1471491	6.9524884	6.5465307	5.2018504	8.2429171
176152.52	3.2358322	2.9756074	3.5127194	3.2184973	2.2921774	4.425139
348327.94	2.7187023	2.5482824	2.8975224	2.7046318	2.0902345	3.4052739
324698.66	3.033582	2.8470695	3.229104	3.0315518	2.4154456	3.7287259
378608.25	3.4917359	3.3060265	3.6851609	3.5076337	2.8736532	4.2653794
149663.38	9.5347309	9.0463867	10.042586	9.5345993	7.8072381	11.455766
327252.47	4.1099763	3.8932374	4.3356409	4.1122074	3.4155724	4.950839
293915.72	4.8449264	4.5965238	5.1032639	4.8468914	4.0095849	5.8118548
310430.41	4.2875953	4.0603199	4.524281	4.3997936	3.6834567	5.2413006
116884.31	14.972069	14.278734	15.690366	14.91834	12.695616	17.493723
158528.23	5.8790789	5.5076475	6.2689695	5.8862948	4.560513	7.4296885
172331.95	3.0986707	2.8413928	3.3729885	3.0718806	2.1405432	4.2131171
349603.69	2.6029475	2.4365535	2.7777126	2.5893724	2.017122	3.3117166
321209.81	2.7707748	2.5917065	2.9589555	2.7790835	2.2177536	3.4819684
377866.97	3.3662641	3.1837914	3.556469	3.3811054	2.745024	4.1082935
147105.44	8.9187727	8.4426441	9.4147596	8.9156885	7.3147583	10.857328
330003.78	3.9302583	3.7192483	4.1501207	3.9389598	3.2477438	4.7482948
299042.84	4.4341474	4.1986666	4.6793962	4.4249454	3.616034	5.3358335
314571.88	3.7892771	3.5771909	4.010654	3.865021	3.1964407	4.6568832
117999.35	15.033981	14.342449	15.750237	14.983998	12.695616	17.493723
155769.28	5.6558008	5.2884493	6.041944	5.6585064	4.4009933	7.2255635
171153.48	2.9096689	2.6596963	3.1768069	2.8957667	1.9898161	4.0001841
349791.56	2.4757602	2.3135931	2.6462963	2.4567773	1.8713973	3.1241021
315344.13	2.7208371	2.5418022	2.9091558	2.7339129	2.1520705	3.3995023
376026.91	3.1646671	2.9873939	3.3497131	3.1777568	2.5526533	3.872088
146141.03	10.489867	9.9712715	11.028438	10.48038	8.713913	12.549096
332724.41	3.6937475	3.4900997	3.906178	3.7096448	3.0468955	4.5046954
303776.34	4.2136264	3.9859262	4.4509439	4.2227826	3.4591787	5.1448598
317987.59	3.7422843	3.5326548	3.9611046	3.8441665	3.164089	4.617806
118243.59	15.924752	15.21352	16.660654	15.866176	13.524613	18.464731
150815.14	6.7102017	6.3030953	7.1367021	6.7197018	5.2823539	8.3442297
168299.84	3.1729085	2.9094667	3.4537983	3.1367075	2.2162528	4.3192358
348990.97	2.704941	2.5351188	2.8831475	2.6807961	2.0902345	3.4052739
312300.31	2.8113966	2.6284864	3.00368	2.8247886	2.2506368	3.5231607
369914.31	3.6332738	3.4416034	3.8328407	3.644912	3.0025694	4.4221802
148825.59	11.631064	11.089534	12.192201	11.611494	9.7079391	13.7369
337257.59	3.7953186	3.5902236	4.0090766	3.8027046	3.1137764	4.5859647
306051.53	4.5972648	4.3601594	4.8439126	4.6016178	3.7732229	5.526475
324101.81	4.1437597	3.9250784	4.3714533	4.1981268	3.4882758	5.0079079
117360.15	12.704483	12.06773	13.366113	12.656222	10.587418	15.001905
145174.28	6.0272379	5.6344438	6.440196	6.0331931	4.7203708	7.633471
164346.22	3.2248993	2.9561539	3.511513	3.2039058	2.2921772	4.425139
342521.34	2.6012979	2.4332752	2.7778659	2.5796299	1.9806268	3.2648778
305673.28	2.6531596	2.4736776	2.8422225	2.6591053	2.0865026	3.31692
358862.09	3.2157199	3.0328393	3.4067459	3.2256436	2.6166968	3.9509034
149961.16	10.029263	9.5287428	10.549253	10.009343	8.3012114	12.052706
338926.47	3.4048681	3.2112305	3.6071303	3.4217918	2.7801113	4.1788831
305714.53	4.1672864	3.9415681	4.4025612	4.1669817	3.3808844	5.0492425
327642.53	3.345109	3.1499753	3.5491662	3.4084864	2.7771537	4.1475921
116003.77	13.577145	12.91482	14.264632	13.534883	11.410446	15.978884
139443.69	6.6550164	6.2336717	7.0973463	6.6598487	5.2823586	8.3442307
160872.92	3.4312797	3.150969	3.7298393	3.4049137	2.4446435	4.6363292
330007.16	2.6666088	2.4933124	2.8487751	2.6390386	2.0536582	3.3585153
299576.19	2.7705808	2.5852759	2.9656601	2.7718842	2.1848984	3.4407487

344041.66	3.3920312	3.2001853	3.5923727	3.4020879	2.7771535	4.1475921
150630.53	10.170581	9.6676083	10.692934	10.156718	8.3836775	12.152055
341091.5	3.6383197	3.4386864	3.8465199	3.6540542	2.9800854	4.4233561
302355.47	4.1738949	3.9467614	4.4106917	4.1783023	3.4200191	5.0970621
333441.13	3.5748441	3.3747597	3.7836933	3.6242747	2.9703155	4.3830056
112962.04	14.155198	13.469811	14.866423	14.106384	11.960542	16.628794
134743.28	9.1433125	8.6398182	9.6684933	9.1422596	7.4787393	11.056982
156558.17	4.643641	4.31218	4.9938202	4.6190486	3.4520772	5.9926143
316397.25	3.8369486	3.6241212	4.0590124	3.8105378	3.0902073	4.6553268
292631.59	4.0631289	3.8354328	4.3008127	4.0847168	3.3819752	4.9103618
326493.53	4.9771276	4.73805	5.2251439	4.9695668	4.2060919	5.861527
151159.8	12.371014	11.816603	12.944721	12.357735	10.373133	14.526258
341836.84	5.8975501	5.6428928	6.160738	5.9154096	5.0432777	6.8787017
298972.41	6.4788589	6.1935158	6.7739568	6.4814382	5.5198331	7.6059666
334858.78	6.3220682	6.0556011	6.5972419	6.3397026	5.4575171	7.3243957
110403.93	12.517671	11.866331	13.195464	12.477487	10.40489	14.784431
132298.3	7.611587	7.1486669	8.0966167	7.6115394	6.0910625	9.3537264
149429.63	3.607049	3.3089232	3.9248245	3.5812314	2.5978715	4.8467531
306324.25	3.2612503	3.0621278	3.4699218	3.2206137	2.5689902	4.0098662
287644.72	2.9793699	2.7832108	3.1857069	2.9813662	2.3824334	3.6876638
311543.81	3.6206784	3.4124427	3.8382976	3.6542168	3.0025697	4.4221802
151291.91	11.031654	10.508693	11.573904	11.012216	9.21033	13.143596
340032.59	3.8231628	3.6181364	4.0367808	3.8356671	3.1472428	4.6265731
294018.94	4.3126473	4.0785117	4.5567207	4.3166351	3.5375662	5.2403889
341526.03	3.7625244	3.5595918	3.9740112	3.7957635	3.131753	4.5787129
106962.1	10.994549	10.375071	11.641352	10.964279	9.0406618	13.148647
132087.58	8.282383	7.7988038	8.7880983	8.28298	6.7422714	10.157076
138416.77	3.9157109	3.5929515	4.2596822	3.873539	2.8290284	5.1610727
292908.03	3.2194405	3.0172114	3.4316592	3.173806	2.5319664	3.9635561
281650.88	3.1137838	2.9110868	3.3268743	3.1036506	2.4815443	3.8107784
294696.44	3.5324485	3.3210957	3.7537251	3.5379982	2.9058576	4.3046055
149621.55	11.201595	10.671675	11.75102	11.187333	9.376071	13.341493
335200.25	3.7857969	3.5803437	3.9999664	3.7944632	3.1137762	4.5859647
285399.59	4.4358859	4.1948714	4.6871371	4.4472089	3.6553023	5.3835235
349559.47	3.6674733	3.4694393	3.8738654	3.6778061	3.0025682	4.4221807
104133.42	9.4878283	8.905364	10.098384	9.4474688	7.6860671	11.50322
129578.88	7.586113	7.1192327	8.0755701	7.5861754	6.0910625	9.3537264
127004.95	3.9840968	3.644475	4.346848	3.9235218	2.9064062	5.2655196
275095.53	3.0862007	2.8820691	3.3009751	3.028053	2.3841808	3.7780037
271990.66	3.15452	2.9469483	3.3728554	3.1351805	2.5146286	3.8517673
275587.44	3.5306396	3.3122537	3.7596416	3.5132651	2.8736532	4.265379
144950.13	9.9206543	9.4144659	10.446988	9.9030628	8.1363945	11.853886
328668.78	4.1652875	3.9475396	4.3919215	4.1644378	3.4491849	4.9912992
280096.31	4.4734612	4.2291656	4.7281885	4.4791789	3.6945887	5.4311938
354092.66	4.1232147	3.9144034	4.3402719	4.1350117	3.4233229	4.9300027
99796.148	10.501408	9.8751593	11.15696	10.478206	8.5879612	12.601342
118161.34	9.2923794	8.7508039	9.8586988	9.291461	7.6429048	11.256458
110880.98	4.888124	4.4852118	5.3175159	4.8159013	3.6091475	6.1991887
247965.33	3.7868199	3.5484531	4.0369873	3.7083197	2.9780972	4.517436
251690	3.5400691	3.3114095	3.7803581	3.4850771	2.8466911	4.2604518
253698.75	3.9180326	3.6782179	4.1693783	3.8751941	3.1964407	4.6568832
136355.42	12.445416	11.860263	13.051969	12.419672	10.456412	14.624798
316790.28	4.0626245	3.8436739	4.2907972	4.0540543	3.3483934	4.8698692
273904.56	4.4760113	4.2289371	4.7337542	4.4800835	3.6945887	5.4311938
356558.75	4.100306	3.8927951	4.3160057	4.1177497	3.4233229	4.9300022
230292.23	2.0148313	1.8356507	2.2067761	2.0404308	1.5729226	2.6375051
336258.03	1.6921529	1.5559533	1.8370813	1.6886698	1.263979	2.2321441
373448.72	1.4593704	1.3394036	1.587199	1.457729	1.0507967	1.9453088
390681.03	1.4231559	1.3073045	1.5465214	1.4163297	1.0205923	1.9040822
401742.75	1.3989052	1.2856245	1.5194924	1.3875123	0.99045837	1.8627853
406893.5	1.6269614	1.5053695	1.7557592	1.6120111	1.2027779	2.1504836
407549.59	1.6513327	1.5289129	1.7809465	1.6275992	1.2027779	2.1504836
407743.84	1.6309259	1.5093077	1.759735	1.6128819	1.2027779	2.1504836

404372.31	1.6148485	1.4933493	1.7435994	1.5911331	1.172261	2.1095695
394500.88	1.3586788	1.2460759	1.4787251	1.3335599	0.9603951	1.8214147
381055.06	1.5299627	1.4082743	1.6593523	1.491855	1.081068	1.9864689
365274.47	1.4673897	1.3457772	1.5970411	1.4382172	1.0507966	1.9453088
351560.63	1.6071197	1.4773169	1.7452718	1.5649648	1.1418023	2.0685971
335319.22	1.5209388	1.3917855	1.6588522	1.473452	1.081068	1.9864691
315474.97	1.6356291	1.4975286	1.7830398	1.5770473	1.172261	2.1095695
292463.97	1.5762625	1.4356395	1.7269369	1.5226744	1.1114039	2.0275643
250023.58	5.8514481	5.5554142	6.1591601	5.7847056	4.9408202	6.7589369
331321.72	5.245657	5.0019107	5.4982095	5.1497493	4.3618731	6.0786209
352697.34	4.7065849	4.4828701	4.9385743	4.6178298	3.853663	5.4757152
355606.63	4.2068958	3.996393	4.4256086	4.176043	3.4491849	4.9912992
359795.72	3.8327305	3.6330857	4.0404925	3.8148971	3.1472421	4.6265726
361259.91	4.5507402	4.333396	4.7761636	4.5431104	3.7861121	5.3951173
365102.53	4.0207887	3.8177128	4.2318611	4.0333138	3.3148274	4.829361
369394.75	3.9253402	3.7258744	4.1327105	3.9439268	3.2477438	4.7482948
375750.09	3.8030598	3.6084113	4.0054793	3.8262739	3.1472423	4.6265726
378951.78	3.6178746	3.4288797	3.8145764	3.6340683	2.9800868	4.4233561
383030.66	3.3939843	3.2119737	3.583622	3.4168944	2.7801111	4.1788826
385736.09	3.3390703	3.159184	3.5265307	3.3508496	2.7136118	4.0972328
386132.91	3.6619515	3.473547	3.857919	3.6755307	3.0134821	4.4640346
382512.41	3.6260262	3.4376869	3.8220007	3.6251624	2.9467087	4.3826585
377287.88	3.8140635	3.6195226	4.0163441	3.8008106	3.1137764	4.5859647
366292.41	3.7429113	3.547385	3.9464114	3.7267225	3.0468938	4.5046964
211745.27	12.094722	11.630787	12.572416	12.271705	10.91241	13.778625
261177.84	11.111202	10.710582	11.522975	11.230812	9.9310837	12.672994
282139.19	9.6264544	9.2677889	9.9954453	9.7783613	8.5460367	11.101516
298867.09	9.1612625	8.82129	9.5109816	9.3020639	8.0994253	10.591604
310569.88	7.6053739	7.3017244	7.9184074	7.7236404	6.6439338	8.9166555
317550.16	8.4648046	8.1477976	8.7909861	8.5545855	7.4108534	9.8019114
322719.91	7.8334184	7.5310044	8.1448603	7.9408946	6.8454604	9.1499128
327718.41	7.1890988	6.9017076	7.485383	7.2338924	6.2013779	8.4026861
330174.22	6.8266988	6.5477519	7.1144743	6.8283215	5.8401747	7.9812794
330214.28	6.6623406	6.3868237	6.9466858	6.6520667	5.6398773	7.7467928
327154.75	6.2264113	5.9589281	6.5028076	6.2043624	5.2401423	7.2769599
324382.41	6.7142978	6.4352479	7.0023346	6.6820993	5.6799145	7.7937121
320449.91	6.8091764	6.5264406	7.1010103	6.7813106	5.7600226	7.8875179
312154.97	6.71141	6.4270673	7.0050931	6.7062821	5.7199631	7.8406205
307711.06	6.4573565	6.1765218	6.7476683	6.4582281	5.4798417	7.5590014
302350.69	6.3204756	6.0402422	6.6103554	6.327702	5.3599377	7.418035
267018.47	14.695614	14.239368	15.162756	15.651584	14.24605	17.178736
305069.38	14.088598	13.670517	14.516215	14.816683	13.429568	16.28093
315162.53	12.37774	11.992334	12.77238	13.007128	11.732533	14.406537
320181.72	10.893814	10.535255	11.261464	11.290091	10.109301	12.601194
325578.25	9.4815922	9.1500378	9.8220901	9.8180304	8.6948195	11.015674
328156.22	10.674794	10.324191	11.034267	11.102142	9.9068689	12.375054
331697.31	9.062479	8.7413769	9.3923607	9.4315996	8.358984	10.637223
335029.22	8.1724215	7.8691449	8.4843922	8.4998817	7.4543266	9.613306
341429.78	7.9079223	7.6124229	8.2119532	8.1618958	7.1535206	9.2712555
345407.38	7.4636507	7.1782928	7.7574439	7.7426901	6.7530866	8.8145447
351657.44	7.2968736	7.0172467	7.5847859	7.549962	6.5864687	8.6240187
353388.84	7.6261606	7.3409281	7.9196362	7.8499522	6.8531237	8.9287939
361578.09	7.9844437	7.6958199	8.2811232	8.1694584	7.1535206	9.2712545
370340.97	7.8657246	7.5826521	8.156661	8.0415716	7.0533414	9.1571484
375474.97	7.643652	7.3665376	7.9285235	7.7645307	6.786427	8.8526335
378473.56	7.8314581	7.5520325	8.1185789	7.9866991	6.9865813	9.0810509
231432.39	0.16851573	0.1198311	0.23036645	0.17062135	0.05798164	0.3657719
337884.56	0.14206035	0.1047441	0.18835142	0.1427803	0.03892462	0.3131173
375323.06	0.12256108	0.0897301	0.1634793	0.12290198	0.03892319	0.3131178
392819.22	0.12219361	0.0900959	0.162011	0.12217845	0.03892319	0.3131179
404067.19	0.0890941	0.0624003	0.12334378	0.0883845	0.02209633	0.2580251
409436.78	0.08059853	0.0554803	0.11319033	0.08094414	0.02209472	0.2580246
410308.31	0.10967363	0.0799967	0.14675194	0.11118457	0.03892313	0.3131159

410720.34	0.11199835	0.0819969	0.1493901	0.11090641	0.03892313	0.3131159
407462.41	0.12761913	0.0953121	0.16735549	0.12757131	0.03892468	0.3131167
397704.44	0.07794733	0.0529614	0.11063999	0.07912574	0.02209487	0.2580247
384224.16	0.11191384	0.0809926	0.15074725	0.11364681	0.03892313	0.3131162
368378.91	0.08143789	0.0549458	0.11625761	0.08061674	0.02209475	0.2580246
354610.88	0.12125968	0.0877562	0.16333604	0.11772221	0.03892315	0.3131162
338256.72	0.10938438	0.0770167	0.15077196	0.10767554	0.03892313	0.3131172
318335.84	0.12251212	0.087118	0.16747802	0.11938471	0.03892316	0.313116
295201.47	0.14227572	0.1025398	0.19231544	0.13990921	0.03892465	0.313116
254230.59	0.39727712	0.3235887	0.48272786	0.40057123	0.20337631	0.6811243
336910.06	0.30275142	0.2468576	0.36751956	0.30200249	0.15242034	0.5838218
359140.78	0.2617358	0.2115092	0.32029831	0.25671151	0.10423549	0.4836853
362703.06	0.23986563	0.1921225	0.29587334	0.23833336	0.10423564	0.4836841
367240.78	0.22873275	0.1824462	0.2831867	0.22806597	0.10423499	0.4836842
369186.72	0.18689729	0.1454172	0.23653048	0.18651882	0.08155163	0.4321602
373618.16	0.2007397	0.1578945	0.25162897	0.20123661	0.0815516	0.4321605
378331.72	0.20881146	0.165318	0.26024139	0.20968935	0.08155157	0.4321606
385094.44	0.20254771	0.1601056	0.25278875	0.20357728	0.08155159	0.4321604
388567.09	0.24191447	0.1954915	0.29604203	0.24416627	0.10423562	0.4836835
392951.09	0.27993307	0.2300709	0.3373951	0.28081289	0.12792072	0.5341733
395902.56	0.22985454	0.1850645	0.28221044	0.23060703	0.10423543	0.4836842
396521.28	0.24967134	0.2029205	0.3039659	0.2507492	0.10423556	0.4836844
393101.53	0.22131687	0.1772658	0.27299353	0.22101553	0.08155153	0.4321601
388155.59	0.28081524	0.2305785	0.33874679	0.2798827	0.12792061	0.5341732
377292.63	0.28094906	0.2300181	0.33979979	0.28007314	0.12792063	0.5341732
219937.11	1.0321132	0.9022053	1.1754737	1.0750307	0.70342094	1.5526135
271888	0.76134288	0.6611538	0.87242502	0.78022611	0.46382254	1.1834183
294767.22	0.61404383	0.5278434	0.7103076	0.62746817	0.3650164	1.0212879
313395.53	0.64136207	0.5557566	0.73642093	0.66085523	0.39762497	1.0756617
326487.81	0.60339159	0.5220705	0.69378936	0.6237725	0.36501589	1.0212879
334643.03	0.51696879	0.4428017	0.60000479	0.52783602	0.3009536	0.9113744
341302.03	0.48930269	0.4179047	0.56940049	0.49788627	0.2695902	0.8557414
347215.97	0.59329069	0.515034	0.68007767	0.59990543	0.33277959	0.9665401
350381.56	0.55082804	0.475854	0.63426173	0.55210674	0.30095515	0.9113733
350802.66	0.55871868	0.4832335	0.64265192	0.55748665	0.30095369	0.9113733
347967.09	0.58626235	0.5085681	0.67246938	0.58254385	0.33277962	0.966539
345389.81	0.5964275	0.517757	0.68367338	0.59117502	0.33277866	0.966539
341549.94	0.52408147	0.450116	0.60673308	0.51661831	0.2695896	0.8557408
332879.53	0.67291611	0.5876729	0.7670489	0.67231905	0.39762527	1.075663
328203.38	0.61851889	0.5363554	0.70970827	0.61914599	0.36501727	1.0212879
322715.19	0.69720924	0.6090784	0.79450911	0.69949692	0.43057016	1.1296955
282003.97	1.5283473	1.3874463	1.6796778	1.6903399	1.263979	2.2321441
324090.53	1.1971964	1.0810217	1.322454	1.279231	0.90049845	1.7384473
337031.06	1.2194722	1.104411	1.3432633	1.358014	0.99045759	1.8627846
344681.03	1.1633945	1.0522984	1.2830285	1.2542987	0.90049797	1.7384475
352084.97	1.2269765	1.1139873	1.3483193	1.3342538	0.96039522	1.8214148
356434.53	1.2007816	1.0897015	1.3201137	1.2732979	0.90049845	1.738447
362128.63	1.3531104	1.2359396	1.478395	1.4560636	1.0507967	1.9453088
366948.53	1.2971846	1.1832545	1.4191238	1.3577461	0.99045759	1.8627846
374822.13	1.3553095	1.2399997	1.4784565	1.4332066	1.0507966	1.9453088
379699.5	1.3326328	1.2190334	1.4539689	1.3953365	1.0205923	1.9040812
387371.5	1.2984951	1.1874834	1.4170904	1.3758667	0.99045759	1.8627846
389998.31	1.602571	1.4793777	1.7332857	1.6855923	1.2639786	2.2321441
399617.69	1.6165451	1.494274	1.7461547	1.6870116	1.2639788	2.2321441
409741.19	1.7352418	1.6100209	1.8676155	1.8387301	1.3870037	2.3948419
415833.59	1.7507004	1.6258204	1.8826275	1.8471296	1.3870022	2.3948419
419922.19	1.8622497	1.7339981	1.9974769	1.9578431	1.4797691	2.5163672
892083.63	11.860995	11.636061	12.089186	12.07085	11.40831	12.770633
1188569.5	7.9347482	7.7754059	8.0965347	8.0500727	7.5130949	8.6258469
1264189.5	5.8788652	5.7459602	6.0140691	6.0183849	5.5486498	6.5102911
1292416.8	4.9302983	4.809978	5.0528679	5.041564	4.6190996	5.4998407
1308763.4	4.8488522	4.7302785	4.9696465	4.9414868	4.5234828	5.3954573
1310634	4.7778401	4.6602268	4.8976712	4.8503399	4.4374661	5.3014741

1312065.9	4.5516005	4.4368854	4.6685314	4.6209378	4.2178159	5.0611243
1310758.9	4.4966316	4.3825588	4.6129222	4.5900617	4.1891847	5.0297551
1308564	4.8725166	4.753643	4.9936113	4.9332604	4.5139236	5.3850164
1294095.6	4.3907113	4.2772799	4.5063891	4.4350615	4.0365615	4.8623786
1274880.6	4.6553378	4.5376453	4.775311	4.6939931	4.2846394	5.1343007
1249369.5	6.5633101	6.4220123	6.7069335	6.5791807	6.0867772	7.092164
1230596.1	4.9975777	4.8734469	5.1240706	5.026454	4.5999727	5.4789677
1205774.5	5.0656238	4.939374	5.1942844	5.0729461	4.6477928	5.5311475
1174196.6	4.8194656	4.6947064	4.9467006	4.8043327	4.3896947	5.2492452
1123044.9	5.3702216	5.2355337	5.5074978	5.3986683	4.9540896	5.864851
710055.75	12.797586	12.535796	13.063468	12.257071	11.583253	12.955689
1056102.6	7.6053214	7.4398966	7.7734971	7.6264868	7.0981426	8.1807985
1180298.5	5.5350404	5.4016261	5.670917	5.6667995	5.2128401	6.1461
1231030	4.7366843	4.6158786	4.8598514	4.8786049	4.4565778	5.3223624
1262334.1	4.5495086	4.4325972	4.6687236	4.6363411	4.2273602	5.0715795
1270949.8	4.6642284	4.5462418	4.7845025	4.7438922	4.3323846	5.1865554
1275083	4.2687416	4.1560822	4.3836818	4.318995	3.9221818	4.7367582
1275955.3	4.236042	4.1238561	4.3505073	4.2689157	3.8745465	4.6843934
1276236.6	4.6856513	4.567636	4.8059444	4.702064	4.2941875	5.1447525
1262086.5	4.0393429	3.9292138	4.1517758	4.0245848	3.6460955	4.4328442
1242583.4	4.2041445	4.0909033	4.3197255	4.1806221	3.7983592	4.6005807
1217244.3	6.0185132	5.8814754	6.1579375	5.9764419	5.5102506	6.4686904
1193918	4.4257646	4.3072286	4.5467362	4.3562102	3.9602997	4.7786403
1160728.1	4.3576093	4.2383375	4.4793868	4.2587929	3.8650212	4.6739187
1117002.1	4.4923816	4.3689351	4.6184316	4.3651762	3.9698308	4.7891092
1042756.7	4.941709	4.8076935	5.0785127	4.7611313	4.3514862	5.2074542
540918.81	7.1581912	6.9344797	7.3872819	6.1698604	5.5700722	6.8281603
669843.56	6.7568011	6.5613704	6.9565744	6.0365486	5.4435611	6.6880045
703981.81	5.9419146	5.7631984	6.124764	5.3413849	4.7806811	5.9508834
719000.38	5.659246	5.4866819	5.8358574	5.102212	4.5602565	5.7046409
730947.75	4.8950148	4.7359228	5.0580888	4.3916421	3.8851967	4.9463663
736515.19	5.6224232	5.452467	5.796329	5.0550752	4.5130606	5.6518369
741428.38	5.0807333	4.9197679	5.2456245	4.5732279	4.0575819	5.1406474
746506.06	4.8144288	4.6583014	4.9744549	4.3183551	3.8225679	4.8756618
751270.94	4.6667585	4.5135484	4.8238435	4.1702638	3.6817677	4.7164617
749336.38	4.2651072	4.1185069	4.4155927	3.7994006	3.3227236	4.3088379
746134.75	4.2994914	4.1519837	4.4509015	3.8002582	3.3383095	4.3265858
738581.31	4.4680252	4.3168688	4.6231232	3.9271281	3.4474752	4.4507537
734626.88	4.8283014	4.6706986	4.9898658	4.2077956	3.7130425	4.7518535
726092.13	4.675715	4.5197425	4.8356967	4.0316639	3.5411334	4.5570951
716070.31	4.7872391	4.6283107	4.9502335	4.0941615	3.6036167	4.6279459
702085.63	4.7187977	4.5594683	4.8822737	4.0096736	3.5255189	4.5393772
418160.75	10.857069	10.543525	11.177569	9.3936844	8.6401281	10.19144
563983.38	8.8318205	8.5882368	9.0805607	8.2884808	7.5869389	9.0446281
619465.94	7.488709	7.274744	7.7073689	7.2352772	6.5846162	7.9469509
646336.13	6.5120916	6.3168273	6.7118573	6.2724614	5.6650033	6.933229
666738.88	5.717381	5.5373087	5.9018178	5.5150442	4.9382992	6.1265988
677344.56	6.4309955	6.2414203	6.624866	6.2018538	5.6017113	6.8631878
685640.94	5.6997762	5.52246	5.8813367	5.446732	4.8752356	6.0563293
693380.19	5.2135901	5.0450063	5.3863711	4.9486938	4.4029913	5.5285721
700455.44	5.039578	4.8746872	5.2086248	4.7204962	4.1987867	5.2994432
699737.94	4.9861526	4.8220615	5.1544032	4.6214719	4.1046348	5.1935959
696763.13	4.7046118	4.5449228	4.8684793	4.3351979	3.8382223	4.8933406
690200.5	4.9217587	4.7576265	5.0901084	4.4767046	3.9635265	5.0347033
685094.63	5.1102428	4.9423542	5.2823801	4.5884266	4.0732646	5.1582985
674235.38	5.2058969	5.0350833	5.3810277	4.610745	4.0889492	5.1759481
659878.56	5.1282163	4.9568739	5.3039703	4.4512606	3.9478569	5.0170398
637495	5.3239632	5.1463404	5.5061526	4.5306783	4.0105462	5.0876842
555142.69	0.44312933	0.3894801	0.50210375	0.41868404	0.27509484	0.628458
688161.75	0.35747409	0.3141951	0.40504897	0.3507328	0.21583426	0.5367502
725241.19	0.30058965	0.2620093	0.34325138	0.29723787	0.1725546	0.4667966
743068.44	0.35124624	0.3099221	0.39654633	0.34659612	0.21583436	0.5367502
757200.44	0.32620162	0.2867861	0.36952105	0.32134956	0.20128165	0.5135593

764700.69	0.33477151	0.295015	0.37839222	0.33004904	0.2012815	0.5135596
771955.63	0.35105643	0.3105	0.39543858	0.35090262	0.21583426	0.5367502
778743.06	0.3865203	0.3440836	0.43274552	0.37728763	0.23050082	0.5598272
785062.69	0.36048076	0.3197011	0.40502065	0.35633612	0.2158353	0.5367502
784084.25	0.38261196	0.3405365	0.42845029	0.36740237	0.2305	0.5598273
781952.81	0.39260679	0.3499129	0.439073	0.38059145	0.24527048	0.5827996
775188.38	0.40764284	0.3639316	0.45515835	0.38929012	0.24527001	0.5827998
772236.69	0.48301256	0.4352327	0.5346055	0.45146573	0.29013693	0.6511567
764380.88	0.48797664	0.4397058	0.54009986	0.45466068	0.30525765	0.6737754
754942.63	0.50334954	0.4540068	0.55659217	0.45943609	0.30525815	0.6737756
741582.63	0.56905323	0.5160484	0.62602484	0.51497447	0.3510527	0.7412002
432461.41	1.2764145	1.1721407	1.3874768	1.2350527	0.96310174	1.5631666
582611.44	0.85648853	0.7829789	0.93504083	0.89295745	0.66776246	1.1792459
641020.94	0.80184585	0.7340152	0.87425864	0.88820791	0.66776198	1.1792459
670530.38	0.70988578	0.6475374	0.77661711	0.78880066	0.57113028	1.0494553
692680.31	0.72472101	0.6627025	0.7909807	0.81711119	0.60321635	1.0928438
705000.38	0.6340422	0.5766193	0.6956358	0.70947951	0.50737798	0.9622571
715401.5	0.70729518	0.6470022	0.7716943	0.79565823	0.58715683	1.0711664
724473.5	0.69843823	0.6389003	0.76203096	0.76576352	0.55513787	1.02771
732697.88	0.74792081	0.6866033	0.8132453	0.8115285	0.60321635	1.0928441
732689.44	0.7192679	0.6591615	0.78338253	0.77787358	0.57113028	1.0494553
730561	0.75695252	0.6951698	0.82275379	0.80217898	0.58715683	1.071166
724481.25	0.87786955	0.8109602	0.94882715	0.91782814	0.68397111	1.2007742
720063.13	0.82492769	0.7599134	0.89401698	0.85391152	0.6354298	1.1361046
709598.06	0.96674442	0.8957451	1.041875	0.977983	0.7327562	1.2651995
695585.81	1.0049084	0.9317827	1.082248	0.99074489	0.7490688	1.286624
673548.81	1.0882655	1.0108984	1.1699833	1.0395061	0.7981469	1.3507564
8501487	7.1081686	7.0516067	7.165071	7.3045597	7.0489426	7.5673795
8533019	6.2362452	6.1833706	6.2894592	6.3994288	6.1603723	6.6456308
8601290	6.5523891	6.4984035	6.6067123	6.7531524	6.5080037	7.0053582
8695364	5.950182	5.8990202	6.001677	6.0996337	5.8685813	6.3376412
8854618	5.960844	5.9100981	6.0119176	6.0635781	5.8357105	6.2982483
8959880	5.852087	5.8021026	5.9023948	5.9201946	5.6969414	6.1501503
9059644	5.5029755	5.4547749	5.5514956	5.5494833	5.3348236	5.7707577
9152614	5.3181529	5.2710114	5.3656111	5.3387418	5.1299806	5.5540276
9312061	5.7061481	5.657733	5.7548752	5.7015448	5.4876657	5.9218135
9364085	5.4111004	5.3640866	5.4584231	5.382906	5.1764278	5.5957108
9458717	5.6599641	5.6121197	5.7081146	5.6058502	5.3966193	5.8213201
9661440	5.3977461	5.3515172	5.4442749	5.3420048	5.1399512	5.5501504
9945706	5.5072012	5.4611754	5.5535178	5.4359441	5.2353168	5.6424842
10177163	5.3973784	5.3523355	5.4427066	5.3399172	5.1432357	5.542376
10411469	5.0509682	5.0078897	5.0943251	5.0114632	4.8230643	5.2055054
10500432	5.5447245	5.4997764	5.5899487	5.518393	5.3215556	5.7208238
4522507	4.3993297	4.3384099	4.4608912	4.7368522	4.5665798	4.9123907
4573457.5	5.4009471	5.3338008	5.4687276	5.8713212	5.6823153	6.0655408
4640926.5	5.0765724	5.0119534	5.1418161	5.5060077	5.3234143	5.6937375
4713740	4.7278805	4.6660094	4.7903671	5.1349039	4.9603982	5.3144612
4810861	4.5821319	4.5218406	4.6430259	4.9551463	4.7847476	5.1304922
4880075	4.8261552	4.7647133	4.8881912	5.2200756	5.0456243	5.3994026
4940181	4.756506	4.6958818	4.8177176	5.1381726	4.9656143	5.3155465
4997644	4.7418342	4.6816516	4.802597	5.1279836	4.956697	5.3040271
5078923	4.6338563	4.5748415	4.6934423	4.9908762	4.8228087	5.1636186
5101840	4.6563592	4.5973334	4.7159538	4.9951196	4.8276739	5.1672125
5145979.5	4.2598305	4.2036238	4.3166008	4.5454535	4.386682	4.7088256
5243911	4.4533553	4.3964195	4.5108447	4.7468553	4.5867009	4.9115191
5373718.5	4.6956315	4.6378708	4.753932	4.9575243	4.794951	5.1244922
5458591	4.4623237	4.4064589	4.5187201	4.6856842	4.5299597	4.8457346
5550379.5	4.3303347	4.2757602	4.3854318	4.5289512	4.3776026	4.6845522
5592333	4.4993744	4.4439497	4.5553174	4.6560769	4.5005498	4.8158188
4571044	0.18923467	0.1768324	0.20227739	0.20164725	0.16836342	0.2402697
4620407	0.22119263	0.2078377	0.23518056	0.23870492	0.20332347	0.2793634
4693501.5	0.52242446	0.5019489	0.54352093	0.56767207	0.5137071	0.6267562
4852169.5	0.53440011	0.514027	0.55537367	0.5875079	0.53381318	0.6461602

4973132	0.52482015	0.5048769	0.54534924	0.57977962	0.52691048	0.6374837
5036754.5	0.56226683	0.5417473	0.58336473	0.62119645	0.56694072	0.680219
5121411.5	0.54320961	0.5232098	0.56377816	0.60035574	0.54728889	0.6581111
5173909	0.55818528	0.5380113	0.57892221	0.61854219	0.565404	0.6763135
5264160.5	0.59534657	0.5746837	0.6165626	0.65191609	0.59756738	0.7108105
5273661.5	0.63637	0.61502	0.65827203	0.69732565	0.64166147	0.7575179
5321043	0.63746899	0.616195	0.65929013	0.69247782	0.63746542	0.7519718
5451175	0.66114187	0.6397312	0.68308645	0.71977055	0.6647504	0.7791666
5602440.5	0.68488008	0.6633793	0.70690036	0.73930526	0.68392694	0.7989347
5694567.5	0.72174054	0.6998421	0.74414992	0.76808828	0.71230257	0.8280571
5755412	0.75146663	0.729236	0.77420276	0.79072863	0.73487186	0.8507261
5839044.5	0.72340602	0.701753	0.74555725	0.75961381	0.70517927	0.8181274
875260.63	10.233523	10.022678	10.447688	10.770999	10.34691	11.20866
764183.81	8.8028555	8.5937386	9.0157757	8.9455185	8.526227	9.3802328
881743.38	9.9382658	9.7312613	10.148565	10.387543	9.9741983	10.814306
776862.69	8.6965179	8.4903679	8.9064083	8.7852411	8.3741283	9.2114716
889898.19	11.221509	11.002483	11.443797	11.671251	11.234847	12.120901
791720.31	9.8064928	9.5895605	10.027096	9.8684378	9.4370098	10.314661
901146.69	10.59206	10.380622	10.806721	10.929007	10.511647	11.359409
809392.19	9.056179	8.8500309	9.2659159	9.0757799	8.6674213	9.4986029
922438.06	10.580656	10.371774	10.792687	10.788721	10.377738	11.212414
836316.88	8.934413	8.7329683	9.1393318	8.914607	8.5161734	9.3270493
938770.5	10.606426	10.399107	10.816839	10.684685	10.281433	11.100414
858268.56	8.9587345	8.7595959	9.1612577	8.8786697	8.4861145	9.2849073
953116.38	9.8476954	9.6494665	10.048971	9.8366976	9.4533319	10.232356
879204.94	8.4633284	8.27211	8.6578512	8.3817654	8.0041161	8.7728119
972424.44	9.9267349	9.7296839	10.126772	9.8554039	9.4774628	10.245407
904434.38	8.4450569	8.2567148	8.6366119	8.3360033	7.9651713	8.7199011
998787.63	10.852157	10.648807	11.058414	10.693812	10.303389	11.095998
935773.44	9.0876703	8.8955364	9.2829075	8.9646769	8.5856533	9.3563595
1017815.4	9.718854	9.5282631	9.9122982	9.5464945	9.1818838	9.9226751
960744.75	8.0375147	7.8592339	8.2188196	7.9372158	7.5847249	8.3020725
1040965.4	10.103122	9.910944	10.298089	9.8709421	9.5031939	10.25002
988752.13	8.6229906	8.4409151	8.8080034	8.5155296	8.1548738	8.8882179
1073097.5	9.5415382	9.3576069	9.7281752	9.3489361	8.995532	9.7133541
1025148.8	8.2270985	8.0524435	8.4045877	8.1167364	7.7708354	8.4742651
1109157.1	9.4242735	9.244463	9.6067019	9.199625	8.8547001	9.5552282
1064258.8	8.1333599	7.9629116	8.3065386	8.0138121	7.6765981	8.3622465
1138139.9	8.9233322	8.7506208	9.0985956	8.7349033	8.4023857	9.0778532
1098039.6	7.8740325	7.7089238	8.0417871	7.7454872	7.4192786	8.0825815
1167445.1	7.9010134	7.7405872	8.0639277	7.7912908	7.4800305	8.1127291
1130475	6.883832	6.7317276	7.038507	6.7882056	6.4872003	7.0997715
1177532.4	8.3607035	8.1963577	8.5275164	8.2209063	7.902894	8.5490179
1144457.6	7.23312	7.0781336	7.390645	7.1203103	6.8142772	7.4367738
3473037.5	5.4635172	5.3860531	5.5418167	5.5603471	5.3675275	5.7583542
3389005	7.6042972	7.5117354	7.6977143	7.6499305	7.4224863	7.8825974
3486669.5	4.4997096	4.4295716	4.5706801	4.5429769	4.3692226	4.7219019
3387744	6.4957685	6.4102244	6.5821686	6.5101557	6.3006558	6.724874
3514096.5	4.6603727	4.5892668	4.7323046	4.7678628	4.590332	4.9505138
3405574.8	6.5281196	6.4425864	6.6145048	6.5382156	6.3288507	6.7527609
3550662.8	4.1941466	4.1270518	4.2620587	4.2519503	4.0854893	4.4234676
3434162.8	5.8156824	5.7353024	5.8969073	5.8094997	5.6134439	6.010685
3605198	4.2286167	4.1617551	4.2962832	4.2526193	4.0877576	4.4224524
3490665.5	5.8166561	5.7369204	5.897223	5.7995348	5.6055293	5.9985814
3635522.3	4.0648904	3.999614	4.1309652	4.0787044	3.9180479	4.2442865
3527319.3	5.6728635	5.5945315	5.752018	5.6464777	5.4562964	5.8416457
3666888	3.7988615	3.7360353	3.8624794	3.8125103	3.6579249	3.9719768
3560435.3	5.3639512	5.2881432	5.4405742	5.3365302	5.1526427	5.5253539
3685346.5	3.5407796	3.4802854	3.6020615	3.5386858	3.3904312	3.6917892
3590408.5	5.1066613	5.0330086	5.1811223	5.0808449	4.9023008	5.2642794
3734856.8	3.8049653	3.7426608	3.8680468	3.7959063	3.6435049	3.9530826
3642643	5.3757672	5.3007336	5.4515972	5.3461499	5.1643767	5.5327387
3732991.8	3.6951597	3.633749	3.7573478	3.6838462	3.533803	3.8386555

3652532.8	5.2736006	5.1993866	5.3486094	5.2420311	5.0624199	5.4264412
3765382.8	3.7626987	3.7009931	3.8251748	3.7330573	3.5830767	3.8877535
3663617	5.5477958	5.4717851	5.624599	5.5135789	5.3299861	5.7019515
3834944.3	3.5804954	3.5208547	3.640893	3.5564103	3.4114099	3.7060351
3728249.8	5.296319	5.2227006	5.3707151	5.2702007	5.0919442	5.4531531
3942227.5	3.7831404	3.7226651	3.844352	3.7517672	3.6049159	3.903111
3830062.3	5.4176664	5.3442001	5.4918909	5.3832641	5.2057905	5.5653114
4034089.5	3.6625364	3.6037152	3.7220771	3.6528463	3.5093405	3.8007421
3906893.5	5.4654675	5.3924031	5.5392742	5.4383001	5.2611227	5.6199698
4128552	3.6073182	3.5496128	3.6657264	3.6043739	3.4633019	3.7497361
3984997.3	5.1917224	5.1212168	5.2629566	5.1705747	4.9993401	5.3462176
4168648.8	4.0466352	3.9857967	4.1081691	4.07692	3.9273753	4.23071
4009792.8	5.7933168	5.7190542	5.8683028	5.7809458	5.5999107	5.9663625
2208710.5	4.7883143	4.6974864	4.8804574	5.4141626	5.2287874	5.6049743
2313796.8	4.0280118	3.9466448	4.1106339	4.0595422	3.9043727	4.2198067
2234860	5.8786683	5.7785702	5.9800649	6.7378645	6.5316048	6.9495153
2338597.3	4.944417	4.854701	5.0353742	5.0047779	4.8330259	5.1815662
2269855.5	5.4452806	5.3497014	5.5421391	6.2133884	6.0156345	6.4164529
2371071.3	4.7236032	4.6365228	4.8119087	4.7986269	4.6311941	4.9710226
2308045.5	5.0904541	4.9988194	5.1833472	5.8174319	5.6286221	6.0114608
2405694.5	4.380024	4.2967877	4.4644675	4.4523754	4.2921748	4.6174617
2357865.8	4.8688102	4.780149	4.9587026	5.5314369	5.3488092	5.7191715
2452995.5	4.3065715	4.2248349	4.3894916	4.3788552	4.220686	4.5418129
2392892.8	5.1673861	5.0767026	5.2592826	5.8562942	5.6686139	6.0490065
2487182.5	4.4978604	4.4148936	4.581995	4.5838571	4.4226346	4.7497988
2423142	5.1136084	5.0239625	5.2044525	5.7679191	5.5822344	5.9585748
2517038.8	4.412725	4.3310375	4.4955664	4.5084257	4.3489938	4.6725187
2447602.8	5.0735354	4.9846888	5.1635685	5.7276812	5.5437799	5.9165001
2550041.5	4.4234576	4.3421984	4.5058551	4.528286	4.3696141	4.6915545
2486853.5	5.014369	4.9267406	5.1031647	5.6143341	5.4331408	5.8003588
2592069.5	4.2687898	4.1896172	4.3490825	4.3674178	4.2124772	4.5268788
2496674.5	5.0318933	4.9442835	5.120666	5.5990958	5.4187641	5.7842274
2605165.5	4.296464	4.2172332	4.3768096	4.3911438	4.2365832	4.5501976
2522675.3	4.5685625	4.4855309	4.6527457	5.0536652	4.8836417	5.2284288
2623304.3	3.9629412	3.8871243	4.039865	4.0372415	3.8897221	4.1892228
2574485.5	4.822711	4.7382498	4.9082999	5.3265796	5.154295	5.5035019
2669425.5	4.097136	4.0207062	4.174654	4.1671309	4.0191073	4.3195362
2641257	5.0335879	4.9483852	5.1198902	5.4906573	5.3174949	5.6683278
2732461.3	4.3689547	4.2909303	4.4480419	4.4243913	4.2724075	4.5806561
2688817.8	4.7589688	4.6768656	4.8421521	5.1617236	4.9961557	5.3317113
2769773.3	4.1743488	4.0986028	4.2511435	4.2096448	4.0637636	4.3597579
2739602.3	4.6795115	4.5988541	4.7612286	5.0489569	4.8871694	5.215075
2810777	3.9899998	3.9164925	4.0645404	4.0089459	3.8680358	4.1540294
2764726.5	4.8160276	4.7345686	4.8985372	5.1111994	4.9464078	5.2802744
2827606.5	4.1897626	4.1146526	4.2658992	4.200954	4.0546918	4.3513627
2261798.8	0.24847481	0.2283538	0.26989362	0.27414897	0.23413841	0.3195551
2309245.3	0.13121171	0.116852	0.14684895	0.12914552	0.10258842	0.1609842
2287107.5	0.30387726	0.2817023	0.32733396	0.34058887	0.29656887	0.3899339
2333299.5	0.14014488	0.125365	0.15618823	0.13682099	0.11007807	0.1687929
2309938.8	0.70521355	0.6713795	0.74031097	0.79896617	0.73331249	0.8698353
2383562.8	0.34528145	0.3220916	0.36969978	0.33637795	0.29410172	0.383677
2373289.5	0.71883351	0.6851239	0.75377256	0.82139707	0.75605637	0.8918191
2478880	0.3578229	0.3346593	0.38216731	0.35361871	0.31156996	0.4005013
2441391.3	0.71147954	0.6784104	0.74574387	0.81740779	0.75336605	0.8863952
2531740.8	0.34482202	0.3223247	0.36847556	0.34215149	0.30045494	0.3885722
2465924.3	0.76441926	0.7302966	0.79972476	0.87475377	0.808927	0.9454653
2570830.3	0.3683635	0.3452729	0.39259225	0.36763912	0.32495448	0.4149727
2515379.5	0.70963442	0.6770923	0.74333644	0.81935602	0.75632662	0.8871843
2606032	0.38257399	0.3591921	0.40707833	0.38135546	0.33825114	0.4290378
2529630.5	0.73330867	0.7003141	0.76745629	0.84621847	0.78311217	0.9140842
2644278.5	0.39065477	0.3671922	0.41522333	0.39086589	0.34769577	0.4385427
2573119.5	0.74773049	0.714689	0.78190547	0.85349286	0.79005587	0.9215989
2691041	0.4496401	0.4246589	0.47570726	0.45033938	0.40507892	0.5000222

2601544.8	0.80836588	0.7741823	0.84367019	0.92939639	0.86439306	0.999015
2672116.8	0.46891662	0.4433091	0.49561763	0.4652549	0.41892987	0.5160207
2591835.5	0.82412642	0.7895438	0.8598339	0.92596316	0.86143076	0.9951055
2729207.5	0.46020684	0.4351046	0.48637971	0.45899248	0.41350013	0.5088381
2685824.8	0.85039055	0.8158692	0.88599718	0.96556836	0.90088111	1.0347121
2765350.3	0.47733557	0.4519293	0.50379819	0.47397268	0.42861965	0.5236211
2756429	0.88230097	0.8475801	0.91807908	0.98945832	0.92479759	1.0584503
2846011.5	0.49367332	0.4681941	0.52017874	0.48915216	0.44305623	0.539419
2807329.5	0.93077785	0.8954282	0.96716523	1.0271438	0.96186131	1.0966719
2887237.8	0.51848865	0.4925533	0.54543519	0.50903273	0.46274373	0.5594422
2861138.5	0.95346659	0.9180195	0.98993182	1.0475583	0.98335147	1.1159296
2894273.5	0.55177927	0.5250459	0.57952106	0.53389895	0.48639229	0.5855225
2888731	0.90697271	0.8725728	0.94238114	0.99034762	0.92827338	1.0565447
2950313.8	0.54367101	0.5173873	0.57094395	0.52887994	0.48208511	0.5797101
319104.41	16.58078	16.136986	17.033688	16.484249	15.864028	17.122749
445779.5	10.556788	10.257303	10.862799	10.461892	10.043579	10.893321
442223.19	7.9891787	7.7278891	8.2570505	7.9794002	7.6106844	8.3613911
432337.34	4.9822206	4.7740188	5.1971655	5.1098566	4.8123002	5.4210777
1054371.8	5.3482084	5.2095184	5.4896555	5.4134078	5.2170753	5.6152811
1395420	6.4554038	6.3227758	6.5901136	6.5098634	6.3221159	6.7017894
1327241.1	6.3537817	6.2188873	6.4908648	6.4295974	6.2382789	6.6253157
1134159.8	6.6084161	6.4596434	6.7597508	6.666573	6.4561801	6.8821077
859635.13	8.1767244	7.9866762	8.3701534	8.198061	7.9301481	8.4727468
1091214.6	6.5450006	6.3940787	6.6985855	6.5263252	6.3116813	6.7464547
334165.38	15.845448	15.421494	16.278103	15.778509	15.185185	16.389278
449304.34	10.451713	10.154895	10.755004	10.376336	9.9608288	10.804852
437094.88	7.8792963	7.6183195	8.1469326	7.8599315	7.4921832	8.2411003
438041.44	4.7575407	4.5554523	4.9662852	4.8937111	4.6049113	5.1959977
1067838.3	5.1103249	4.9756265	5.247746	5.1843362	4.9937639	5.3803811
1366917.1	5.6477456	5.5224562	5.7751603	5.7129502	5.5356388	5.8945303
1356207.3	5.4910488	5.3670354	5.6172047	5.5553684	5.3795271	5.7355161
1136585	5.4162245	5.2817607	5.553246	5.4663134	5.2761645	5.6615963
864560.75	6.3454189	6.1786056	6.515595	6.3611555	6.1260047	6.6030469
1082305.1	5.0161457	4.8835912	5.1513863	4.983923	4.7961283	5.1772437
349237.78	17.830257	17.390116	18.278723	17.775497	17.159052	18.408625
453660.78	11.387363	11.078933	11.702203	11.319713	10.887474	11.764821
435740.59	8.8997908	8.6218634	9.1843958	8.8842497	8.4923744	9.2895727
442979.31	5.5961981	5.3780499	5.8209243	5.7367969	5.4250965	6.0618196
1092136.1	5.9937582	5.8494301	6.140748	6.0818524	5.877809	6.2912426
1346282.6	5.5367274	5.4117408	5.6638722	5.593667	5.4166451	5.7750244
1382033.9	5.0563164	4.9384518	5.1762834	5.1160183	4.948936	5.2873263
1127592.3	4.981411	4.8519816	5.1134186	5.0274801	4.844399	5.2157388
889098.44	6.2883925	6.1246276	6.4554272	6.3029366	6.0719805	6.5404463
1082528.1	5.9240956	5.7799821	6.0708942	5.949132	5.7433729	6.1604023
365493	16.52836	16.114162	16.950512	16.486237	15.905939	17.082474
463464.94	10.75378	10.457277	11.056559	10.694993	10.279736	11.122842
435928.97	8.3224564	8.0538254	8.597765	8.2950382	7.9169292	8.6865873
445651.97	4.9859533	4.7807751	5.1976733	5.122745	4.829752	5.4289827
1121656.4	5.5676584	5.4304175	5.7074914	5.6414976	5.4474673	5.8407331
1329038.6	5.0735922	4.9532084	5.1961627	5.1216946	4.9511657	5.296617
1405478.3	4.637567	4.5256572	4.751545	4.6939206	4.5353079	4.8566895
1113834.1	4.6137929	4.4885015	4.7416954	4.658915	4.4816751	4.8413982
925940.44	5.186079	5.0404224	5.3348761	5.1948938	4.9894276	5.4066577
1088877.6	4.9748473	4.8432398	5.1091251	4.9799671	4.7926736	5.1727328
384803.53	15.558589	15.16695	15.957783	15.518852	14.970165	16.08268
484370.97	10.572475	10.284869	10.866084	10.53227	10.12888	10.947701
441486.53	8.568778	8.2978754	8.8462725	8.535181	8.1541471	8.9294786
448093.91	5.2243514	5.014842	5.4403653	5.3355498	5.0367422	5.6475163
1153775.8	5.7116814	5.5746026	5.8512793	5.7801485	5.5865149	5.9788389
1327381.3	5.5003037	5.3748527	5.6279445	5.5470977	5.3694615	5.7291293
1433961.9	4.6368036	4.5260134	4.7496209	4.6874495	4.5304217	4.8485503
1123521	4.467206	4.3444633	4.5925369	4.5096488	4.336009	4.6884875
953862.69	5.1107984	4.9683275	5.2563181	5.1199312	4.9189854	5.3269887

1103360.8	4.6358366	4.5096536	4.7646556	4.6368356	4.4575181	4.8215442
400556.06	14.811909	14.437383	15.193692	14.767309	14.24312	15.306045
506063.56	11.518316	11.2245	11.817877	11.488479	11.076034	11.912437
444386.53	8.0267954	7.7655196	8.2946215	8.0067711	7.6388745	8.3878422
446032.88	5.1946845	4.9852991	5.410604	5.3173909	5.0188251	5.6291733
1166876.1	5.6364164	5.5010114	5.7743125	5.6993318	5.5083513	5.8953104
1327879.8	5.3672028	5.2433105	5.4932833	5.4138651	5.2385693	5.593554
1452564.3	4.581553	4.4721317	4.6929746	4.6320648	4.4770327	4.7911181
1135243.9	4.2766142	4.1571541	4.3986363	4.3147688	4.1457458	4.4889383
970823.94	4.8814206	4.7434192	5.022418	4.8889236	4.6943188	5.0895343
1109453.5	4.3580017	4.2360182	4.4826064	4.3509512	4.1779585	4.5292978
411936.94	13.50935	13.156718	13.869039	13.465125	12.9717	13.972699
527089.31	10.669919	10.392859	10.952494	10.654002	10.264771	11.054286
451217.16	7.5174446	7.2665677	7.7747731	7.5078745	7.154314	7.8744059
442077.97	5.0805516	4.8725886	5.2951083	5.2059155	4.9098358	5.5153203
1180908.1	5.2434225	5.113625	5.3756814	5.3030109	5.1202664	5.4906945
1329467	4.9944825	4.8750658	5.1160855	5.0369873	4.8680949	5.210268
1462605.1	4.4126744	4.3056684	4.521668	4.4611669	4.3096304	4.6166959
1156436	4.1256065	4.0093617	4.2443666	4.1607261	3.9962232	4.3302813
985167.5	4.578917	4.44626	4.7145267	4.5860367	4.3990555	4.7789364
1112739.4	4.0081263	3.8913488	4.1275182	4.0070581	3.8413804	4.1780682
423068.78	13.981178	13.627123	14.342104	13.928122	13.432782	14.437235
552062.13	10.56584	10.296412	10.840534	10.55719	10.17839	10.946543
459433.06	7.6180849	7.3677697	7.8747354	7.6090794	7.2563753	7.9745116
442294.84	4.6190906	4.4209456	4.8238287	4.7277331	4.4460368	5.0227442
1193969.3	5.0311179	4.9046845	5.1599855	5.089613	4.9115376	5.2725749
1319375.5	4.8015141	4.6839967	4.9212341	4.8378539	4.6718335	5.0082951
1464185.8	4.1436	4.0399833	4.2492023	4.1855297	4.0389338	4.336113
1179741.1	3.9406953	3.8282232	4.0556326	3.9744225	3.8153465	4.1384506
1000609.3	4.165462	4.0399528	4.2938786	4.171411	3.9945745	4.3540759
1117874.1	3.7195604	3.607353	3.8343704	3.6933458	3.5351195	3.8568754
435506.09	14.199572	13.847844	14.557972	14.148714	13.656319	14.654488
579392.31	11.663945	11.387494	11.945412	11.652935	11.264528	12.051392
474208.94	8.5531921	8.2919693	8.82055	8.5453148	8.1771774	8.925766
445453.69	5.2642961	5.0533628	5.4817719	5.3626952	5.0629506	5.6756425
1219430.8	5.2590108	5.1310768	5.389329	5.3199968	5.1397357	5.5050411
1329235.6	5.2579088	5.135354	5.3826499	5.2936006	5.1205297	5.4710565
1466615.6	4.4156084	4.308712	4.5244865	4.4533701	4.3021545	4.6085649
1214028	4.0789833	3.9661572	4.1942048	4.108696	3.9490931	4.2731094
1012376.3	4.5309243	4.4007421	4.66398	4.5362816	4.3528194	4.7255025
1135813.4	3.8527455	3.7394307	3.9686215	3.8243079	3.6646452	3.9891822
441600.69	12.561122	12.232714	12.896113	12.510976	12.051989	12.983162
600462.81	10.272077	10.017307	10.531687	10.261672	9.9038677	10.629176
490589.34	7.7865534	7.5415664	8.0374718	7.7813201	7.4361176	8.138422
445907.34	4.6624036	4.4641209	4.8672252	4.7560997	4.474761	5.0506272
1224045.4	5.3462071	5.217452	5.4773369	5.4006686	5.2193618	5.5867395
1323785.3	4.950954	4.8318086	5.0722952	4.9834218	4.8154149	5.1558309
1442195.8	4.3829002	4.2755103	4.4923053	4.4165707	4.2647333	4.5724535
1238194.1	4.0147176	3.9038801	4.1279039	4.0443673	3.8876333	4.2058172
1016052.1	4.2861977	4.159833	4.4154253	4.2909818	4.1129255	4.4747772
1141251.9	3.777431	3.665503	3.8919079	3.748517	3.5909531	3.9112597
447244.63	12.194668	11.873153	12.522685	12.155293	11.704765	12.618852
618175.94	11.255696	10.992761	11.523333	11.242327	10.872966	11.621106
514370.88	8.3422298	8.0944719	8.595643	8.3366146	7.987546	8.6970215
449926.13	5.2008538	4.9922423	5.4159431	5.3079877	5.0122895	5.616744
1227582.1	5.6077714	5.4760747	5.7418356	5.6774893	5.492218	5.8675108
1332885.6	5.4055653	5.2814608	5.5318499	5.4494309	5.2742763	5.6289587
1419628	4.6293817	4.5181265	4.7426844	4.6671848	4.5098572	4.8286209
1270420.9	4.1702714	4.0587249	4.2841067	4.2022319	4.0444627	4.3645968
1015250.8	4.2797308	4.1534128	4.4089146	4.2859397	4.1079392	4.4696846
1163232.4	3.601172	3.4929359	3.7119095	3.5624468	3.4106307	3.7193375
450515.34	11.475746	11.165042	11.792906	11.434019	10.999367	11.881611
642222.94	10.620922	10.370351	10.876019	10.607563	10.255339	10.968854

542393.81	8.0660954	7.8288331	8.3087215	8.0653687	7.7308798	8.4106073
463114.09	4.9814939	4.7802715	5.1890097	5.0699553	4.7849841	5.3676047
1242234.6	5.2099662	5.0838003	5.3384714	5.2666264	5.0891147	5.448832
1368454.6	4.9851851	4.8675818	5.1049123	5.0225267	4.8565726	5.1927409
1413600	4.5012732	4.3913455	4.6132574	4.5364633	4.3809552	4.6960998
1317857.4	3.9973977	3.890173	4.1068287	4.030333	3.8787296	4.1863666
1034971.8	4.1199193	3.9971771	4.2454724	4.1289058	3.9561234	4.3073239
1186075.6	3.6152837	3.5078757	3.7251449	3.5741754	3.4237072	3.7296133
452610.78	10.686886	10.387815	10.992383	10.650638	10.231463	11.082696
663750.94	10.851962	10.602781	11.105521	10.836006	10.486044	11.194739
576633.56	8.2842903	8.0510139	8.5226107	8.2837286	7.9548225	8.6227427
480420.69	4.7708187	4.5774837	4.970221	4.8423133	4.5689769	5.1278596
1278734.6	5.622746	5.4935217	5.7542429	5.6770344	5.4952173	5.8634095
1422577	5.2081537	5.09023	5.3281202	5.2484417	5.0820618	5.4189191
1419933.4	4.6445842	4.5331573	4.7580576	4.6793389	4.5216722	4.8411145
1367616.1	4.1371255	4.0300207	4.2463565	4.1653008	4.013824	4.3210459
1047176.8	4.285809	4.1613283	4.4130678	4.2915306	4.1160202	4.4726095
1236251.6	3.4976699	3.3941863	3.6035068	3.4626207	3.3175371	3.6124663
451443.06	10.016767	9.7269192	10.313058	9.9839296	9.5777006	10.40308
679756.25	10.594975	10.351678	10.842546	10.582729	10.240541	10.933483
605414.5	7.8904624	7.6682758	8.1174526	7.8899322	7.5766726	8.2128191
499565.63	4.6060014	4.4197068	4.79813	4.6627102	4.3999486	4.937202
1314315.5	5.1661868	5.0440288	5.2905564	5.2183056	5.0469322	5.394114
1479531.6	4.9360213	4.8234558	5.0505509	4.9776077	4.818769	5.1403861
1418824.8	4.6460991	4.5346107	4.7596354	4.6897864	4.5319991	4.8516879
1401274.9	4.2803879	4.1727405	4.3901095	4.3130026	4.1607642	4.4694057
1043484	4.4361005	4.3092179	4.5657706	4.4455876	4.2666965	4.6300669
1283552.3	3.7520874	3.6468585	3.8595819	3.7145231	3.5670562	3.8665698
454115.31	8.7114439	8.4420729	8.9872227	8.6760941	8.2997236	9.0653162
686795.94	9.2866011	9.0600739	9.5173607	9.272646	8.9542236	9.5995474
631157.31	6.9443855	6.740303	7.1530776	6.9441476	6.6564274	7.2411056
525851.56	4.3529396	4.176424	4.5349975	4.3921371	4.143733	4.6516743
1347812.6	4.9443078	4.8263021	5.0644693	4.983974	4.8179598	5.1543131
1534055.5	4.6100025	4.5031781	4.7187209	4.6482387	4.4974289	4.8028488
1425761.5	4.4467468	4.337955	4.5575771	4.4938941	4.339879	4.6520042
1431587.3	4.2190933	4.1133547	4.3268623	4.2521548	4.1025858	4.4058003
1055977	4.3949823	4.2694373	4.5232821	4.403636	4.226552	4.5862427
1318355.4	3.659863	3.5573156	3.7646165	3.6355283	3.4913814	3.7841291
447306.84	8.5221138	8.253705	8.7970285	8.4880486	8.112649	8.8764982
684544.56	10.233081	9.994832	10.475574	10.215422	9.8807659	10.558584
643770	7.5523868	7.3415742	7.7677174	7.5517144	7.2544856	7.8579969
546368.69	4.4731698	4.2975674	4.6541061	4.5015597	4.254993	4.7588325
1354009.4	5.1137018	4.9939532	5.2355962	5.1587014	4.9903688	5.3313384
1555256.1	4.8763676	4.76723	4.9873729	4.9203115	4.7661881	5.0781827
1425796.1	4.760849	4.6482582	4.8754773	4.8091168	4.6496191	4.9727087
1443352	4.7181835	4.606782	4.8315992	4.7469711	4.589323	4.9086614
1063076.9	5.1134586	4.9784193	5.2512331	5.1280699	4.9375658	5.3240576
1336950.9	4.9044433	4.7864442	5.0246167	4.8720055	4.7061062	5.0422893
1328864.4	0.84282494	0.7941814	0.89366847	0.84482974	0.77630609	0.9177808
1194054.4	3.0149381	2.9172475	3.1150663	3.0125661	2.8749311	3.1550918
895335	6.816443	6.6464882	6.9896445	6.8166847	6.5772018	7.0626726
1104253.4	8.2164116	8.0482054	8.387248	8.5831451	8.336504	8.8352346
1371238.6	1.0909845	1.0363942	1.1477038	1.0941893	1.0172111	1.1754495
1203496	3.5978518	3.4914773	3.7066436	3.5949955	3.4450815	3.7497585
903044.25	8.0018225	7.818377	8.1884861	8.0035772	7.745101	8.2685003
1095678.4	10.631769	10.439568	10.826621	11.091985	10.810322	11.379133
1409599.8	1.1017312	1.0476116	1.1579217	1.102071	1.0258644	1.1824416
1200138.4	3.5270934	3.4216318	3.634979	3.5285492	3.3797793	3.6821821
931612.5	7.5374684	7.3621912	7.7158651	7.5395255	7.2925363	7.7927637
1099576	9.7783136	9.5943489	9.96492	10.14639	9.8776913	10.420547
1443123.6	0.99367785	0.942907	1.0464722	0.99642569	0.92485845	1.0720615
1190513	3.2523797	3.1507347	3.3564689	3.2531178	3.1097674	3.4013722
971815.5	6.9920678	6.8267956	7.1603308	6.9941268	6.7611532	7.2330918

1108287.9	9.1898508	9.0122328	9.3700895	9.5607424	9.3012838	9.8256083
1479166.5	1.0316621	0.9805437	1.0847541	1.0381691	0.96608955	1.1142164
1205033.6	3.3194094	3.21733	3.4239035	3.3189499	3.1750195	3.4677246
1002037.3	6.6574373	6.4986277	6.8191471	6.6604314	6.4366159	6.890058
1124623.8	8.7558174	8.5837221	8.9304953	9.0491133	8.7987518	9.3047934
1505787	1.2239447	1.1686976	1.2811291	1.227878	1.1499532	1.3096954
1221402.8	3.4812434	3.3773837	3.5874848	3.4801531	3.333719	3.6313655
1020933.7	6.9632339	6.8022981	7.1270161	6.9674568	6.74055	7.2000666
1131951.9	9.1417313	8.9664335	9.3195944	9.4547062	9.1998014	9.7148867
1520865.9	1.2604662	1.204667	1.3181832	1.2635412	1.184857	1.3460782
1247450.4	3.4590554	3.3566084	3.5638347	3.4586947	3.3142219	3.607846
1036552.3	7.1612401	6.9992461	7.3260369	7.1675982	6.9391613	7.4016519
1135312.3	8.6698618	8.4994221	8.8428602	8.9652719	8.7176571	9.2181396
1528236.9	1.2164345	1.1617603	1.2730172	1.2186835	1.1416255	1.2995749
1276262.4	3.576067	3.473063	3.6813505	3.5769358	3.4316349	3.7268076
1052694	6.9231896	6.7651448	7.0839944	6.9291162	6.7061563	7.1576071
1140450.9	8.7570629	8.5861473	8.9305239	9.0534353	8.8053083	9.3067865
1537723.3	1.3019248	1.2455134	1.3602326	1.3030419	1.2235615	1.3863301
1316545.6	3.5198171	3.4191964	3.6226473	3.5199988	3.3780789	3.6663496
1065664.6	6.7253799	6.5705681	6.8829188	6.7340927	6.5157881	6.9578605
1158989.4	8.3969707	8.2309618	8.5654869	8.6651278	8.4245272	8.910861
1519093.4	1.3369818	1.2794619	1.3964211	1.3379556	1.2569236	1.422841
1346693.6	3.375675	3.2782521	3.4752581	3.3739238	3.2365911	3.515588
1070669.9	6.7686596	6.613709	6.9263244	6.7791109	6.5606298	7.0030303
1165383.3	8.5225182	8.3557234	8.6918049	8.7501593	8.5091829	8.9962292
1500534.9	1.2188987	1.1636715	1.2760699	1.2195405	1.1417644	1.3012201
1385578.4	3.1098926	3.0177231	3.2041616	3.1094861	2.9795232	3.24366
1071448.5	6.1636186	6.0158491	6.3141003	6.1702747	5.9618955	6.3840895
1188417.5	7.7237167	7.5665078	7.8833694	7.9213738	7.6945767	8.1531601
1497675.1	1.2512727	1.1952558	1.3092372	1.2513274	1.1724577	1.3341076
1440718.5	3.1262181	3.0355778	3.2188773	3.1240439	2.9962986	3.2558384
1093382.6	6.3372145	6.188868	6.4882188	6.3438454	6.1346745	6.558341
1212134.9	8.2878571	8.1265726	8.4515362	8.4954233	8.2629976	8.7327328
1504427.1	1.5228388	1.4611131	1.586502	1.5247642	1.4377568	1.6156608
1497722.5	3.3410728	3.2491357	3.4349515	3.3396034	3.2099779	3.4731221
1107883.8	6.7561245	6.6039252	6.9109459	6.761416	6.5467358	6.9813509
1263685	8.2718401	8.1140175	8.4319601	8.4686537	8.2415466	8.7004385
1503369.9	1.2691487	1.2128342	1.3274033	1.2686256	1.1893574	1.3517901
1537097	3.1813216	3.092773	3.2717624	3.1802552	3.0553994	3.3089046
1105945.8	6.2914476	6.1444778	6.4410448	6.2946553	6.0874476	6.5071273
1312178.3	8.0796947	7.926621	8.2349825	8.2327518	8.0130939	8.4569063
1506805	1.1852894	1.1309499	1.241565	1.1833886	1.1070006	1.2636659
1573293.6	3.1227481	3.0360322	3.2113128	3.1206155	2.9983823	3.2465553
1121680.3	6.0026021	5.8600702	6.1477251	6.0046802	5.8037686	6.2107825
1348600.4	7.8622251	7.7132778	8.0133257	8.0203466	7.8066735	8.23839
1500985.8	1.8827627	1.8139802	1.9534857	1.8784734	1.7817526	1.9790902
1589204.6	3.3368893	3.2476759	3.4279325	3.3347416	3.2089686	3.4641833
1132318.6	6.0610147	5.9184575	6.2061391	6.061502	5.8605094	6.2676363
1369824.1	7.4243107	7.2807107	7.5700302	7.5533681	7.3476906	7.7633448
1280100.4	0.05233965	0.0405625	0.0664695	0.05115231	0.03537474	0.0717621
1208302.3	0.12000309	0.1012659	0.14120209	0.11974525	0.09377863	0.1506947
924366.81	0.30291006	0.2684656	0.3405484	0.30261931	0.25560755	0.356082
1158274.4	0.32203075	0.2901754	0.35642847	0.34817782	0.30160832	0.4000215
1328725.6	0.05719766	0.0450653	0.07159141	0.05657749	0.04005806	0.0777104
1198977	0.12510665	0.1058871	0.14680612	0.12286367	0.09708332	0.1536846
929629.56	0.27860558	0.2457053	0.31468415	0.27743652	0.23279622	0.3285016
1163074.9	0.46170714	0.4234772	0.50246173	0.50072116	0.44482496	0.561843
1327404.4	0.11601588	0.0984162	0.13585481	0.11663559	0.09206749	0.1457467
1219035	0.21410377	0.1889145	0.24171665	0.21283212	0.17802778	0.2525313
970932.5	0.66122001	0.6110547	0.71440595	0.66106713	0.59257561	0.7356415
1176129.5	1.1860939	1.1246616	1.2500093	1.2841582	1.1942526	1.3791839
1434886.4	0.09687178	0.0814375	0.11438031	0.09663583	0.07525949	0.1222124
1210344.4	0.23629639	0.2097015	0.26532999	0.23547274	0.19848913	0.2773784

1015170.1	0.60975003	0.5626551	0.65973461	0.6098811	0.54551053	0.6800712
1191768.6	1.2997489	1.2358209	1.3661264	1.3994645	1.3061066	1.4978635
1485078.3	0.11379872	0.0972881	0.13230844	0.11384655	0.09086262	0.1408761
1225896.4	0.22269419	0.1970586	0.25073871	0.22243094	0.18667085	0.2630526
1048472.6	0.58942884	0.5438674	0.63778824	0.58986062	0.52740997	0.6579452
1213684.9	1.2771025	1.2143083	1.3423018	1.3820181	1.2906363	1.4783422
1497860.5	0.09813998	0.0829166	0.11534891	0.09800921	0.07688545	0.1231513
1243268.9	0.25899467	0.2314754	0.28888541	0.25841454	0.22003829	0.3015743
1069945.9	0.64021927	0.5931669	0.69001162	0.64115977	0.57634413	0.7114895
1225679.1	1.3690369	1.3043091	1.4361455	1.4778106	1.3837849	1.5767219
1528192.1	0.11386003	0.0975702	0.13209182	0.1142001	0.09149379	0.1408346
1270630.9	0.22508505	0.199752	0.25274113	0.22434336	0.18910132	0.2642739
1088932.5	0.58313996	0.53866	0.63031334	0.58434528	0.52272147	0.6513892
1233656.1	1.3674799	1.3029964	1.4343288	1.4622463	1.3686595	1.5606806
1521194.1	0.10649528	0.0907274	0.12421585	0.10669039	0.08475261	0.1325762
1301405.3	0.21899404	0.1943047	0.24595164	0.21833473	0.18398225	0.2572643
1108315.3	0.5846712	0.5405152	0.63147318	0.58601058	0.52457184	0.6527726
1242994.5	1.4424844	1.3764814	1.5108342	1.5430307	1.4473954	1.6434711
1531197.1	0.10775883	0.0919437	0.12551342	0.1077109	0.08572563	0.1336167
1343346	0.23300029	0.2078998	0.26029623	0.23255181	0.19754553	0.2719861
1123793.4	0.64513642	0.5990245	0.693856	0.64699221	0.58293402	0.7162957
1265824.3	1.5254881	1.4581993	1.5950811	1.604553	1.5071648	1.7066509
1492762	0.12125174	0.1042302	0.1402604	0.12164889	0.09789239	0.1494287
1374727.8	0.22768144	0.2031539	0.25435427	0.22726724	0.19306	0.2658009
1130729.5	0.64117897	0.59535	0.68959969	0.64292032	0.57903802	0.7120149
1275442.3	1.6754973	1.6052051	1.7480755	1.7709632	1.6694514	1.8771402
1470996.5	0.11556792	0.098848	0.13430588	0.11556768	0.09231177	0.1429008
1415037.8	0.22614239	0.202041	0.25232756	0.22614263	0.19245201	0.2640349
1132506.4	0.65253496	0.60633	0.7013272	0.65427899	0.59012121	0.7236409
1302502.4	1.6606495	1.5913956	1.7321416	1.750446	1.650629	1.854825
1507314.9	0.10681245	0.0909505	0.12464516	0.1066571	0.08463351	0.1326668
1458093	0.23866791	0.214248	0.26510853	0.23893693	0.20475259	0.2771966
1156439	0.63989538	0.5946155	0.68770897	0.64175922	0.57921225	0.7094041
1329327.9	1.771572	1.7007369	1.8445995	1.8606256	1.7587001	1.9670138
1514110.1	0.14529987	0.1267328	0.16582166	0.14556131	0.1196507	0.1754197
1530201.5	0.22872806	0.2053903	0.25399119	0.22823787	0.19568738	0.2646698
1171807.6	0.64515704	0.5999817	0.69283259	0.64645392	0.58358604	0.7143498
1386321.3	1.8112684	1.7411094	1.8835293	1.902135	1.8014284	2.0071142
1513316.6	0.13678566	0.1187853	0.15674308	0.13673142	0.11165272	0.1657644
1571139.8	0.2590476	0.2344888	0.28547925	0.25818583	0.22398704	0.2961586
1169961.8	0.6598506	0.6141191	0.70808595	0.66053659	0.5968852	0.7292226
1440149.1	1.8914709	1.8211005	1.9638641	1.9794829	1.8786352	2.0844374
1479640	0.13516802	0.1170831	0.15525532	0.13531435	0.11007449	0.1646109
1608689	0.26792005	0.24322	0.29444835	0.26800367	0.23340957	0.3062804
1186482.3	0.63717771	0.5925611	0.68426353	0.63745797	0.57516789	0.7047125
1480600.8	1.9843297	1.9132192	2.0574071	2.0761003	1.9744077	2.1817822
1511745.9	0.14023522	0.1219922	0.16043688	0.14045008	0.11499327	0.1698648
1625550	0.23622774	0.2131884	0.26107809	0.23646404	0.20420496	0.2723724
1197667.8	0.62872195	0.5846115	0.67527878	0.62868088	0.5671882	0.6950922
1504081	1.9114662	1.8422272	1.9826416	1.9907954	1.8917973	2.0937183
290482.72	8.9093075	8.5693302	9.2593145	8.9995613	7.5354033	10.677292
1619280.3	7.7522101	7.6171832	7.8890295	7.9830527	7.3796	8.6243525
289121.13	7.0869951	6.7834249	7.400651	7.3669348	6.0219936	8.9323578
167575.88	7.5189819	7.1094966	7.9459028	8.2980957	6.4232259	10.576841
1436297.8	7.5889559	7.4471488	7.7327847	7.7425814	7.1130238	8.4146976
350893.41	7.962532	7.6699929	8.2633724	8.0560398	6.7804723	9.5149183
1574591.6	6.2346325	6.1119051	6.3592048	6.635663	6.0679812	7.2438946
1734289.4	6.8564105	6.7337222	6.980773	7.04562	6.4949679	7.6320906
1038954.8	6.3313627	6.1792741	6.486249	6.5709996	5.8855791	7.3169785
290176.5	7.1956205	6.8902583	7.5110312	7.3058414	5.9905605	8.8349781
1636605.4	6.8031058	6.6773195	6.930666	7.0133047	6.4490771	7.6148839
292485.75	5.5592451	5.2922864	5.8361821	5.7033329	4.5437746	7.0809274
167862.89	6.5470095	6.1656103	6.9458265	7.1821051	5.4356327	9.3326225

1434535.4	6.513607	6.3821988	6.6470399	6.6767974	6.0914526	7.3046203
355225.94	6.0384107	5.7855482	6.29948	6.1093674	5.0127668	7.3864522
1569756.6	5.9875526	5.8671093	6.1098461	6.2990355	5.7446837	6.8940129
1740473.6	5.8242769	5.7114425	5.9387798	5.9878573	5.4814363	6.529789
1045897.4	5.9680805	5.8209343	6.1180062	6.1953549	5.5317526	6.9188099
291777.25	7.1458616	6.8423953	7.459321	7.3484421	6.0221572	8.8867521
1653265.3	6.9710531	6.844357	7.0995054	7.2139282	6.6447845	7.8199039
297374.94	6.0294256	5.753541	6.3151207	6.2010026	4.9942203	7.62112
170956.83	6.4811687	6.1051183	6.8743205	6.8449721	5.1520624	8.933733
1449597.6	6.3797016	6.2503319	6.511075	6.5618854	5.9831495	7.182488
360073.34	6.0904255	5.8381624	6.3507853	6.2835875	5.1694922	7.5742378
1573974.9	6.5350471	6.4093585	6.6625805	6.8127294	6.2341146	7.431849
1753471	6.3975968	6.2797499	6.5170994	6.6013927	6.0701704	7.1676283
1050798.8	6.5692883	6.4152217	6.7261205	6.8388343	6.1411371	7.5957756
294526.81	6.0028491	5.7262678	6.2893362	6.1803484	4.9753842	7.595624
1664703.5	6.2725883	6.1528478	6.394073	6.4777761	5.9412494	7.0507846
300305.06	5.7474885	5.4795084	6.0251846	5.9049168	4.7331061	7.2877126
172702.09	5.8540115	5.4986773	6.2262821	6.1610737	4.5758758	8.1382093
1466998.1	5.749155	5.6271048	5.8731856	5.8824425	5.3397641	6.466373
365250.31	5.872685	5.6267624	6.1265888	6.0321879	4.9480772	7.2891517
1598797.8	5.7668333	5.6497149	5.8857679	5.9357224	5.4026232	6.508678
1775403.5	5.9963832	5.8830123	6.1113896	6.1503887	5.6424675	6.6927266
1056677	6.0065656	5.8596926	6.156189	6.2532201	5.5896883	6.9755378
299228.81	5.6979809	5.4306974	5.9750156	5.8535757	4.6890197	7.2249947
1685349.6	6.1429391	6.0251727	6.2624278	6.2797923	5.7547903	6.8407221
306797.22	6.1017504	5.8284431	6.3845649	6.2211981	5.0280738	7.6200471
176993.52	5.672524	5.3270264	6.0345483	5.8982162	4.3613644	7.8155775
1489284	5.8907504	5.7681212	6.0153298	6.0087185	5.4651322	6.5928044
373778.25	6.1801348	5.9306555	6.4374123	6.3407445	5.2438226	7.6054082
1634178.4	5.7215295	5.6061378	5.8386984	5.7982516	5.2788711	6.3565755
1813674.9	5.9701991	5.8582711	6.0837278	6.0899301	5.5911107	6.6222711
1075333.6	6.1246109	5.9775777	6.2743473	6.3400273	5.6788483	7.0587454
303532.44	5.366807	5.1093235	5.6339049	5.4737864	4.3625293	6.7892728
1693726.8	5.9631815	5.847446	6.0806322	6.0847178	5.5705824	6.6345282
311186.97	6.047811	5.7776322	6.327363	6.1296248	4.9547148	7.5070982
180627.14	5.7964711	5.4506373	6.1584949	6.0567131	4.5156975	7.9685478
1498121.9	5.6851182	5.5650134	5.8071618	5.775888	5.2455406	6.3464241
381171.72	6.154706	5.9081473	6.4089108	6.1924696	5.1286383	7.4202414
1658219.6	5.922617	5.8060551	6.0409303	5.9281635	5.4067502	6.4879513
1844824	5.7084036	5.5998921	5.8184886	5.8012052	5.3203797	6.3148561
1088469.9	6.0277276	5.8827462	6.1753793	6.2191091	5.5695329	6.9253569
308311.63	5.0111637	4.7643752	5.2674208	5.096561	4.03339	6.3613982
1700782.5	5.6115351	5.4995117	5.7252655	5.7033219	5.2069459	6.2351933
314098.75	5.6479053	5.3881135	5.916986	5.7074575	4.5850296	7.0299487
184853.92	5.3880382	5.0585732	5.7333274	5.6097951	4.1455312	7.4378343
1501180	5.4483809	5.330936	5.5677609	5.5314803	5.0142956	6.0887246
385771.69	5.4643717	5.2335701	5.7027316	5.4829974	4.492341	6.6351943
1703605.6	5.4361172	5.3259592	5.5479808	5.4096103	4.9192963	5.9373884
1863005.9	5.3778682	5.2730732	5.4842219	5.4430442	4.9800181	5.9384685
1098034.8	5.8550062	5.7127495	5.9999099	6.0220613	5.3862319	6.7139115
310803.84	5.138289	4.8893423	5.3966269	5.2244925	4.1540108	6.4954009
1712574.5	5.6044278	5.4928603	5.7176909	5.6915412	5.1993489	6.2189245
315805.59	5.6395454	5.3806429	5.9076867	5.7386127	4.6158476	7.0595031
187715.78	5.071497	4.7544165	5.4041648	5.2204161	3.8291652	6.9701734
1507932.5	5.1255608	5.0119214	5.241127	5.1702929	4.6725354	5.7078114
389873.19	5.4120164	5.1835327	5.6479778	5.4672241	4.4807763	6.6126018
1737731.4	5.2332597	5.1262479	5.3419433	5.2023258	4.7261653	5.7151904
1881353	5.2265577	5.1237569	5.3309016	5.2591848	4.8089843	5.7413268
1108823.9	5.3940034	5.2581582	5.5324702	5.5533695	4.9479542	6.2140703
314519.69	4.8232274	4.5835395	5.072197	4.9121847	3.8761032	6.1459222
1730453.1	5.9608665	5.8463817	6.0770292	6.0225582	5.5182486	6.561594
321837.78	5.6705589	5.413353	5.9368286	5.7379451	4.6273379	7.0428886
192332.2	5.5164971	5.1895132	5.858685	5.6664896	4.2269802	7.4557719

1531220.1	5.6614985	5.5429406	5.7819533	5.6927905	5.1740232	6.2505894
398313.31	5.6990314	5.4669757	5.9384046	5.6913509	4.6970367	6.8405504
1790777.9	5.5098958	5.4017086	5.6197052	5.3949666	4.9180274	5.9075003
1909740	5.6625509	5.5563226	5.7702999	5.6746984	5.2098284	6.1708694
1122866.6	6.0541472	5.9110761	6.1998067	6.1920037	5.5560617	6.8822289
315347.94	4.3317232	4.1050296	4.5676789	4.3773522	3.4068418	5.5454392
1725336.4	5.6035452	5.4923978	5.7163754	5.6344776	5.1488118	6.1548705
324614.31	5.2185011	4.9729252	5.4730659	5.2557731	4.2009912	6.502985
195315.61	5.2274365	4.9116683	5.5581794	5.3613963	3.9738796	7.0941067
1538758.5	5.3393693	5.2245321	5.4560943	5.3602109	4.8590069	5.900156
402279.44	5.3718882	5.1477623	5.6032605	5.3938227	4.4320359	6.5084395
1808842	5.3835545	5.2771535	5.4915614	5.277607	4.8090687	5.781147
1929698.4	5.5728917	5.4680552	5.6792326	5.5606537	5.1049047	6.0472493
1123892	5.3848591	5.2500372	5.5222673	5.4740276	4.8802133	6.1218505
316879.06	4.5664105	4.3341317	4.8079047	4.600327	3.6131749	5.7836151
1730812.8	5.9087849	5.7948155	6.0244317	5.9220891	5.4247341	6.4539037
327768.94	5.5832014	5.3302999	5.8450027	5.6235762	4.5416183	6.8952103
196589.97	5.224071	4.9094152	5.5536046	5.3179297	3.9417779	7.0343795
1551208	5.5640512	5.447279	5.6826959	5.5729232	5.0647173	6.1194139
405586.34	6.0209131	5.7844548	6.2645559	6.0060878	4.997921	7.164978
1849242.3	5.5298324	5.4231672	5.6380682	5.3484116	4.8840904	5.8468909
1956301.5	5.8165879	5.7102008	5.9244595	5.7960858	5.3332343	6.2892499
1124328.5	5.6273589	5.4895425	5.7677608	5.6980882	5.0957336	6.3541307
319521.88	4.2907858	4.0666389	4.5240736	4.3297215	3.3726373	5.4810486
1760968.5	5.5866985	5.4768424	5.698204	5.5946212	5.115591	6.1074305
336468.41	5.2248588	4.983448	5.4749413	5.2713461	4.2321863	6.494669
201195.25	5.6214051	5.2985244	5.9588156	5.8038354	4.3768487	7.5601573
1593317.6	5.3454504	5.2325215	5.4602022	5.3380151	4.8474226	5.8657985
412606.84	5.5040288	5.2799621	5.7351599	5.4639645	4.5117803	6.5638862
1908461.9	5.1397409	5.0385251	5.2424788	5.0050035	4.5622458	5.4808455
1992538.5	5.5898542	5.4865193	5.6946468	5.5538568	5.105742	6.0316567
1136361.4	5.5589714	5.4227223	5.6977777	5.6035666	5.0091281	6.2509098
324164.31	4.645792	4.4140911	4.8864989	4.6586976	3.6750247	5.8334141
1800370.4	5.7443738	5.6341901	5.8561702	5.7295146	5.2513447	6.2406826
347337.81	5.4183564	5.1762977	5.6688128	5.4205208	4.3846755	6.6348743
208192.83	5.8935747	5.5683823	6.2328029	5.9551387	4.547215	7.6784534
1643287.6	5.3405137	5.2293577	5.4534369	5.328908	4.8471293	5.8467073
423466.19	5.7478023	5.5217032	5.980783	5.6993756	4.7404475	6.8018289
2000968.5	5.2894387	5.1891422	5.3911858	5.1455956	4.7065849	5.6161275
2040379.5	5.5700421	5.468102	5.6734052	5.5238266	5.0826354	5.9939642
1157538.4	5.7509971	5.613667	5.8908381	5.7771688	5.1798878	6.4262524
327259.69	4.5132351	4.2859745	4.7494178	4.52915	3.5646303	5.6827989
1829103.3	5.5661154	5.4585142	5.6753039	5.5621233	5.0938468	6.0628452
356106.84	5.723002	5.4772043	5.9769874	5.7356691	4.680572	6.9649758
213195.66	5.8819208	5.5608354	6.2167115	5.9437981	4.5557289	7.6388168
1670052.9	5.2393551	5.1301436	5.3503056	5.2189479	4.7464681	5.7268343
430007.22	5.0371246	4.8272061	5.2538228	4.9815054	4.0974302	6.0067167
2094024.9	5.0548587	4.9590154	5.1520891	4.9525037	4.5314674	5.4037127
2075183	5.5802307	5.4790521	5.6828084	5.5336156	5.0957518	5.9999261
1182229.1	5.8355861	5.6986876	5.9749432	5.8528728	5.2569208	6.4994783
331129.53	4.4453902	4.2211709	4.6784272	4.4461403	3.5008817	5.5787158
1858692.5	5.2660675	5.1622529	5.3714442	5.2529874	4.8021436	5.7357254
364734.69	4.8363919	4.613306	5.0674772	4.8689027	3.9090853	5.9996657
221190.36	4.9911757	4.7010617	5.294508	5.0546169	3.7982159	6.6086912
1701640.4	4.8976269	4.7930355	5.0039263	4.8917451	4.4383855	5.3798151
436593.56	4.8695178	4.6647038	5.0810089	4.864594	3.9939606	5.8735752
2176620.8	4.7118912	4.6211352	4.8039808	4.6758013	4.2727728	5.1078491
2116355.8	5.0341253	4.9389825	5.1306396	5.0035133	4.5919642	5.4429274
1204511.5	5.8862038	5.749979	6.0248408	5.8934579	5.3013997	6.5351787
331785.41	5.1569476	4.915463	5.407227	5.1554289	4.1290903	6.3682332
1880553.6	5.90943	5.800065	6.0203395	5.9127517	5.436368	6.4206247
357388.91	5.4030781	5.1647491	5.6495671	5.4403825	4.4111924	6.6449804
220932.61	5.7212019	5.4101105	6.0455179	5.8366075	4.4858289	7.4881907

1697855.3	5.3567581	5.2472267	5.4679999	5.3330498	4.8592997	5.8415675
433142.72	5.263854	5.049984	5.4844532	5.210041	4.3067632	6.252346
2236693.3	5.3820524	5.2863336	5.4790697	5.5050912	5.0717492	5.9666042
2141468	5.4005008	5.3025179	5.4998393	5.3684883	4.9446406	5.8198414
1200611.9	6.017765	5.8797956	6.1581545	6.0179448	5.4198899	6.665853
158755.45	6.8407097	6.439858	7.2599788	7.3260169	6.2238173	8.5824146
869847.5	5.5400519	5.3847265	5.6987205	5.8276143	5.3981271	6.2846303
155128.77	4.879817	4.538343	5.2401767	5.2195745	4.3065324	6.2908454
81169.984	5.0511284	4.5739722	5.5645342	5.5507631	4.2776003	7.1312184
789511.31	4.2659297	4.1230626	4.4124846	4.4970536	4.1098919	4.9144807
204519.81	4.12185	3.8482652	4.4097505	4.4418287	3.71573	5.287787
720842.38	3.5389154	3.4029047	3.678968	4.1308174	3.7292662	4.5670452
955004.94	3.8544302	3.7309089	3.9809992	4.168973	3.8292072	4.5339565
587726.94	4.0512009	3.8900943	4.2172666	4.2456412	3.8118987	4.7202773
159964.72	8.4706182	8.0255499	8.9339447	9.1457529	7.8965368	10.547626
884340.38	6.9622517	6.7894197	7.1383705	7.4495726	6.9697289	7.9565105
158063.33	5.3522849	4.9976506	5.7254424	5.7201905	4.7709661	6.824616
81621.688	5.8317833	5.3195848	6.3799877	6.4194026	5.0408759	8.103734
795027.13	5.3331513	5.1738186	5.4961433	5.6438251	5.2079992	6.1096539
208617.27	4.3380876	4.0600224	4.6301823	4.7100353	3.9661512	5.571547
721880.88	4.4924312	4.3391309	4.6497645	5.3421612	4.881146	5.838006
967775.63	4.5744076	4.440639	4.7111816	4.9712825	4.6013956	5.3660383
596166.25	5.1193771	4.9393473	5.3042917	5.4145093	4.9273362	5.9418898
162259.34	7.9379096	7.5102692	8.3835554	8.5369911	7.3409538	9.8834925
898621.5	6.1627727	6.0015182	6.3272624	6.5669608	6.1159644	7.0446081
161302.33	4.9286327	4.5919394	5.2834845	5.2527456	4.340857	6.3159795
83589.961	5.3355689	4.8518171	5.854497	5.7893066	4.4570208	7.418581
808192.38	4.902298	4.7508254	5.0573711	5.1696053	4.7569017	5.6118355
213332.88	4.0687585	3.8025491	4.3486905	4.3669834	3.6484168	5.2003016
724756.38	4.619483	4.4643178	4.7786655	5.5379214	5.0661259	6.0445881
984575.56	4.2657976	4.1377521	4.3967981	4.653172	4.2998581	5.0309091
604296.25	5.1547565	4.9753084	5.3390226	5.4096656	4.9192772	5.9396224
164482.55	7.5631123	7.1486201	7.995369	8.2754784	7.1217594	9.577467
908818.75	6.1299353	5.9700127	6.293057	6.5524707	6.1056609	7.0255914
163878.48	4.7169099	4.3902082	5.0614867	4.9554834	4.0769286	5.9827576
85098.773	4.3713908	3.9384053	4.8389797	4.8209691	3.6422715	6.2926311
821444.13	4.7063456	4.5591474	4.8570867	4.9629269	4.5603795	5.3944969
217439.47	3.8401492	3.584065	4.1096997	4.1731896	3.4913552	4.9679585
737150.75	4.2704968	4.1226063	4.4223375	5.1528211	4.7019491	5.6380982
1003059.9	3.7814291	3.6620355	3.903724	4.0985928	3.7695925	4.4515343
612367.13	4.3829918	4.2187276	4.5520134	4.5991259	4.1556416	5.081697
167211.97	6.4588675	6.0793576	6.855865	6.8405437	5.7954216	8.0315304
923896.5	5.7928567	5.6386871	5.9501729	6.1825299	5.7500472	6.6408815
167121.97	4.9544654	4.6227021	5.3037496	5.2530122	4.3602633	6.2913489
87568.75	4.3965459	3.9682932	4.8584161	4.8536744	3.6988392	6.2935448
837334.25	4.5919538	4.4479446	4.7394381	4.7991366	4.4045358	5.2221894
223110.98	3.9128509	3.6575639	4.1812582	4.2179441	3.5291603	5.0162649
751547.13	4.0982127	3.9547443	4.2455559	4.9998865	4.5598149	5.4738321
1027220.1	3.7898402	3.6717157	3.9107978	4.0824575	3.7557635	4.4324975
625849.5	4.3269186	4.1654692	4.4930229	4.5086632	4.0719872	4.983521
168756.89	6.7197256	6.3342633	7.12251	7.1280131	6.0626884	8.3374224
933409.5	6.0059385	5.8497376	6.1652541	6.3872485	5.9481301	6.8519545
169726.13	5.1789312	4.8421769	5.5329323	5.4503317	4.5332484	6.5102997
89560.961	4.9351859	4.4857635	5.4174418	5.3754182	4.1392345	6.888556
846537.88	4.6778769	4.5333023	4.8258886	4.9122601	4.513936	5.3387165
227914.55	3.9927244	3.7374887	4.2608008	4.2767997	3.5782504	5.0823107
761820.94	4.4472389	4.2987361	4.5995631	5.4277501	4.967464	5.9215708
1046803.5	4.1459546	4.0235152	4.2711735	4.4761357	4.1361117	4.8390331
635544.81	4.552	4.3876224	4.7209606	4.7419662	4.2975426	5.223949
170711.73	6.642777	6.2617288	7.0409493	7.0051713	5.9535003	8.19911
940294.38	5.950264	5.7953601	6.1082602	6.3293648	5.8933296	6.7907953
171826.92	5.2145495	4.8786573	5.5674753	5.4406505	4.5278754	6.4944201
91525.258	5.1570463	4.7022386	5.6439667	5.6370058	4.3934865	7.1516275

852868.38	4.360579	4.2215471	4.5030231	4.5917206	4.2109709	5.0004106
231008.13	5.1296897	4.8417459	5.430285	5.4688668	4.6826911	6.3603268
778084.69	4.4596686	4.3125062	4.6105723	5.4101262	4.950099	5.9031034
1061727.8	4.0462351	3.9261348	4.1690755	4.3470244	4.0123935	4.7041807
642133.56	4.2530093	4.0949812	4.4155741	4.4453683	4.0139799	4.9138961
172344.02	6.7655382	6.3827324	7.1653032	7.1733704	6.1159806	8.3715076
950419.38	5.8995008	5.7460818	6.0559788	6.2741675	5.8421283	6.7313237
173508.5	4.7259932	4.4080157	5.0608478	4.9343705	4.0779653	5.9305987
92997.813	5.1184001	4.6688576	5.5995445	5.5610423	4.3194427	7.0687585
860313	4.4367571	4.2971101	4.5797873	4.6323481	4.2487001	5.0436535
233076.48	6.0838399	5.7712669	6.4089417	6.6194992	5.7658067	7.5772591
789467.75	4.2154984	4.0734816	4.3612032	5.1108794	4.6651797	5.5891223
1075651.6	3.9120474	3.7947311	4.0320683	4.2202463	3.8949351	4.5677805
649865.69	4.3978319	4.2380605	4.5620852	4.5911951	4.1567469	5.0623064
174673.52	7.1390333	6.7482467	7.5465474	7.4921408	6.4129696	8.7098322
962448.13	5.8247294	5.6732416	5.9792385	6.1726131	5.7449889	6.6250124
176051.91	4.8962831	4.5748367	5.2343583	5.1037436	4.2433248	6.1018519
94650.219	4.6381297	4.2143526	5.0929766	5.0318103	3.8770945	6.4486775
873749	4.3273296	4.190485	4.4675055	4.5373693	4.1609836	4.9409652
237766.97	3.4235201	3.192344	3.6670127	3.6993406	3.0702913	4.431088
806979.56	4.239265	4.0983853	4.3837514	5.1896605	4.7463198	5.6648788
1092939.9	3.9883254	3.8707962	4.1085162	4.2771716	3.9498563	4.6263165
659663.75	4.5568671	4.3954082	4.7227407	4.6992388	4.2584286	5.1760793
174898.64	6.2436161	5.8787446	6.625205	6.5036368	5.5016737	7.6439843
961132.25	5.3613849	5.2159882	5.5098066	5.6766882	5.2693124	6.1088581
176438.19	4.477489	4.1706653	4.8009133	4.6650019	3.8445001	5.6228342
95437.555	4.8618178	4.4294519	5.3249836	5.2356653	4.046145	6.6839643
877685.38	4.1780348	4.0438895	4.315496	4.3554053	3.9882426	4.7496371
240019.39	3.3372304	3.1100919	3.576571	3.6279917	3.0182874	4.3395896
814355.13	3.8987906	3.7643428	4.0368137	4.7125411	4.2920828	5.164506
1103461.9	3.7608912	3.6473286	3.8770905	4.0015383	3.6865315	4.3381467
658411.69	6.7799525	6.5825052	6.9818182	6.9730701	6.4334283	7.5486455
175775.39	6.1100707	5.7500687	6.4867077	6.3116736	5.3358798	7.4250846
964345.19	5.3932972	5.2477069	5.5419025	5.666081	5.2577877	6.0990391
177891.41	4.4128046	4.1094689	4.7326059	4.5598073	3.7541776	5.5015516
96252.313	4.3531423	3.9462523	4.7905955	4.6802292	3.5749285	6.0419621
884689.19	4.1822596	4.0485744	4.3192348	4.3331499	3.9691296	4.7239995
242075.33	3.2262685	3.0039382	3.4606991	3.4696367	2.8700647	4.1699228
829682.94	3.8978746	3.7646794	4.0345788	4.713903	4.2969508	5.161788
1117698.6	3.6217277	3.5110085	3.7350502	3.8385103	3.5339928	4.1643386
657569	4.0740972	3.9212685	4.2313561	4.1710148	3.7583838	4.6197624
176539.73	5.930676	5.5768356	6.3010821	6.167985	5.2114592	7.2615571
977800.69	5.5410066	5.3944349	5.6905518	5.8012466	5.3926473	6.234148
182080.66	4.4211173	4.1209426	4.7373767	4.5688376	3.7785006	5.4921656
98411.266	4.4507103	4.0436044	4.8876987	4.7397766	3.6554894	6.0749331
905315.25	4.1753411	4.0432854	4.3106112	4.3104229	3.9524748	4.6945658
245560.63	3.4288886	3.2011657	3.6685348	3.6754539	3.062238	4.3877501
858571.75	4.0089836	3.8761604	4.1451974	4.8943453	4.4763627	5.3423038
1136532.9	4.0922704	3.9754972	4.2116027	4.3151717	3.9955249	4.6557326
663098.19	4.4186516	4.2600908	4.581605	4.4855566	4.0629325	4.9439788
178049.98	5.8859878	5.5349784	6.2534218	6.0185771	5.0738001	7.099
994104.63	5.6342158	5.4876175	5.7837386	5.8532119	5.4450278	6.2852635
186955.08	4.8032928	4.4942307	5.1280103	4.8906603	4.0734701	5.8368206
102460.3	4.0308299	3.6514072	4.4389672	4.2614255	3.2537248	5.5093255
927393.56	4.3120852	4.1794624	4.4478459	4.4280028	4.0697279	4.8118339
251516.73	5.0175586	4.7445149	5.3022194	5.2962132	4.5516725	6.1366496
903635	4.9776735	4.8332577	5.125309	5.8692608	5.4160504	6.3508821
1158843.4	3.9565313	3.8428278	4.0727448	4.1373663	3.8284624	4.4667788
670759.75	4.3666902	4.2099671	4.5277553	4.4097447	3.992327	4.8625669
178555.27	5.7517209	5.4052835	6.1145382	5.8692741	4.9497604	6.9241099
1001365.3	5.3117476	5.1699476	5.4564514	5.4976616	5.1076493	5.9113646
189771.98	5.0692415	4.7539268	5.3999729	5.1063924	4.2830086	6.0569172
104384.45	3.8415682	3.4747255	4.2366037	4.0455246	3.0757575	5.2508039

934805.19	4.7956519	4.6562858	4.9381294	4.8682914	4.4921908	5.2697344
254394	3.8011904	3.5653529	4.0485282	3.9743886	3.3391654	4.7049112
944673.13	3.9346943	3.8092079	4.0632615	4.7112374	4.3180122	5.1317039
1172005.8	3.819947	3.7088618	3.9335139	3.9631753	3.6625857	4.2840576
678635.81	4.4280009	4.2710819	4.589211	4.4179115	4.0029793	4.8678989
179868.14	5.8987656	5.5491233	6.2646661	6.0138345	5.0808959	7.0808482
1010522.2	5.315074	5.1738691	5.459156	5.441123	5.0557647	5.8499527
191859.56	4.6179609	4.3188519	4.9323263	4.6060815	3.8342938	5.5039701
106646.21	4.3602114	3.9728613	4.7751241	4.4611936	3.4220343	5.7294254
945537.81	5.021481	4.8796535	5.1663842	5.0729823	4.6912861	5.4797411
257523.34	3.3123209	3.0937386	3.5422721	3.4590106	2.8816643	4.1308517
983882.44	3.3967471	3.2825513	3.5139022	4.1710119	3.8122029	4.5560145
1188870.3	3.5647287	3.4582045	3.6737003	3.6657329	3.3785536	3.9729226
685669.31	4.4788356	4.3218145	4.6401029	4.4550171	4.0413814	4.9033756
180315.69	5.6456542	5.3041286	6.0034003	5.6553354	4.7492514	6.6952028
1020292.2	5.3582692	5.2171636	5.502224	5.4702954	5.0849996	5.8788195
187628.33	4.306386	4.014535	4.6138453	4.2759175	3.5253043	5.155757
105354.5	4.3946867	4.0034513	4.8138232	4.4941988	3.4588962	5.7617993
943115.13	6.5379081	6.3757277	6.7031703	6.583282	6.1287589	7.0628581
254730.02	3.2936833	3.0745559	3.5243051	3.4180171	2.8321517	4.0992894
1019150.6	3.0358615	2.929822	3.1447582	3.7115107	3.37871	4.0696235
1200672.4	3.641293	3.5341489	3.7508605	3.7268963	3.4390211	4.0345807
681074.38	4.3093677	4.1548591	4.4681525	4.2585945	3.8485382	4.7036242
141760.11	0.39503357	0.2984041	0.51298398	0.37336329	0.18571229	0.7036535
895363.19	0.23007424	0.1997268	0.26372969	0.24694358	0.16985817	0.3523298
159332.77	0.26359928	0.1899792	0.35630962	0.27913842	0.11400346	0.6146124
82932.453	0.32556614	0.2145501	0.47368166	0.33568555	0.09900305	0.9066946
805761.19	0.17623088	0.1484378	0.20771672	0.18509164	0.11502434	0.2867209
179313.5	0.15057427	0.0992294	0.21907766	0.13971375	0.04485749	0.3505861
733412.63	0.14180285	0.1158632	0.17181788	0.15780556	0.08814441	0.2637567
972211.69	0.14400156	0.1211368	0.16992749	0.15503575	0.09640298	0.2400476
600956.31	0.20134576	0.1670714	0.24058248	0.21380913	0.13177972	0.3389428
167206.13	0.37079981	0.2842901	0.47534862	0.42433444	0.22393176	0.786042
915626.31	0.22170617	0.192255	0.25439274	0.24265172	0.16845998	0.3446173
119556.68	0.39311898	0.288849	0.52276492	0.31903839	0.15833367	0.6033355
51186.906	0.23443496	0.121136	0.4095107	0.16095798	0.03625913	0.5004471
815001.44	0.20490761	0.1750079	0.23845053	0.21538325	0.14183725	0.3201657
213191.66	0.22514953	0.1660075	0.29851559	0.25256118	0.12369037	0.5095936
737401.44	0.19256811	0.1621985	0.2269728	0.22177851	0.13963151	0.3399473
989161.75	0.18096131	0.1554216	0.20950027	0.19399628	0.12897445	0.2847869
612074.81	0.26467353	0.2254854	0.3087146	0.27696842	0.18229163	0.4135339
170385.48	0.80992812	0.6804389	0.95688742	0.93434495	0.63887709	1.3853346
933873.31	0.56431639	0.5171586	0.61461878	0.61438906	0.49274108	0.7624846
144029.41	0.58321422	0.4651945	0.72205889	0.54530573	0.31916443	0.9096871
86117.852	0.71994364	0.5519766	0.92293525	0.80563015	0.41336164	1.509733
831091.25	0.46685609	0.4215529	0.51570129	0.50035328	0.38582587	0.6446937
154313.69	0.58322757	0.4689838	0.71688569	0.4520731	0.28114849	0.7163486
742552.81	0.47404036	0.4258067	0.5262416	0.57350546	0.43719074	0.7447959
1008893	0.43513039	0.3953733	0.47780225	0.47252712	0.36973545	0.5999332
622244.63	0.5978356	0.5386201	0.66178352	0.6365577	0.49118146	0.8220376
173343.63	0.82495099	0.6952853	0.97178042	0.94348228	0.64536351	1.3934994
946987.56	0.62936413	0.5798442	0.68198252	0.68951696	0.56701475	0.8383182
169706.98	0.55978835	0.4529022	0.68431228	0.61173648	0.37217006	1.0070574
74224.563	0.80835772	0.6168621	1.0405166	0.77016306	0.40311658	1.4075575
846599.75	0.47838426	0.4329224	0.52732193	0.50932354	0.39556834	0.6523249
223049.5	0.40798119	0.328481	0.50091046	0.4532035	0.27742061	0.7488085
756796.5	0.43340582	0.387766	0.48294091	0.51007885	0.37984601	0.6746473
1029569.4	0.50312293	0.4607232	0.54837537	0.54997212	0.44164425	0.6823316
631891.5	0.56497037	0.5078777	0.6267249	0.59384215	0.45418322	0.7729496
176535.53	0.66842067	0.5532695	0.80047071	0.7373209	0.46171656	1.1606219
964648	0.58777916	0.5403879	0.63821328	0.64331961	0.5230543	0.7891488
173264.83	0.6175518	0.5060986	0.74624813	0.6611625	0.39014232	1.0808237
90457.242	0.47536272	0.3440224	0.64031047	0.53213167	0.22896643	1.1342041

864331.56	0.53798801	0.4901945	0.58918226	0.57583988	0.45492667	0.7252343
229127.27	0.47571817	0.3906141	0.57385767	0.51215923	0.31468675	0.8241268
772599.38	0.4465445	0.4006624	0.49624059	0.54672444	0.41513428	0.7120524
1055550.5	0.47084433	0.4303539	0.51411813	0.51888579	0.41715699	0.6440919
646617.81	0.55519658	0.4992439	0.61570466	0.58952653	0.4538351	0.7637097
178452.13	0.8461653	0.7165856	0.9924053	0.94348508	0.63630378	1.3957084
976147.69	0.6361742	0.5871159	0.68823755	0.69848084	0.57303166	0.849102
176212.38	0.62992173	0.5182004	0.75858819	0.68254358	0.41939041	1.0931774
78180.297	0.83141154	0.6416659	1.0597023	0.77680594	0.40158436	1.4067088
875111.06	0.56221437	0.513627	0.61415923	0.59989011	0.4780162	0.7498998
234327.64	0.48223078	0.3974264	0.57977438	0.52194864	0.32405403	0.8316633
784053.44	0.43364391	0.3887698	0.48227671	0.53829312	0.40784091	0.702101
1076816	0.5228377	0.4805361	0.56786519	0.57701993	0.46996605	0.707022
657453.75	0.57190335	0.5155507	0.6327346	0.59947503	0.46373093	0.7731177
180827.95	0.76315635	0.6411449	0.90162903	0.84267998	0.55829531	1.2711109
985352	0.63327622	0.5845566	0.68497276	0.69366384	0.57113308	0.8412001
178701.08	0.58197743	0.4755178	0.70516306	0.61799234	0.36339715	1.0172272
94802.203	0.54851043	0.4096538	0.71929848	0.62790561	0.31541851	1.2257153
882983.13	0.50397336	0.4582304	0.55304658	0.54148531	0.42673352	0.6841426
237855.34	0.49609983	0.4106349	0.59410697	0.52725369	0.32704905	0.8367844
802006.31	0.42767745	0.3836094	0.47541973	0.51207244	0.38261941	0.6741112
1093655.1	0.51570189	0.4740139	0.56007385	0.5631845	0.45795929	0.6909697
665228.38	0.59227782	0.535232	0.65374821	0.6302619	0.49394259	0.8040522
182865.44	0.68356276	0.5689916	0.81443411	0.75039881	0.48739377	1.1566722
997643.75	0.64351624	0.594694	0.69527817	0.70459545	0.58044219	0.8533633
180675.59	0.71952158	0.6011584	0.85437351	0.781896	0.51067466	1.1966027
81610.938	0.63716948	0.4758686	0.835563	0.59993684	0.29800802	1.1502373
891939.44	0.5605762	0.5125108	0.61193532	0.59982514	0.48042995	0.7468332
240904.19	0.56453979	0.4736508	0.66778737	0.62173313	0.40116766	0.9490319
814845.06	0.43198398	0.3880295	0.479554	0.52877134	0.40014178	0.6895312
1109401.8	0.50748074	0.4664217	0.55118573	0.56002629	0.45833895	0.6840128
674022.94	0.57564807	0.5197878	0.6358757	0.61632264	0.48068649	0.7887321
185698.45	0.94238806	0.8079327	1.0928237	1.0159098	0.6919592	1.4774619
1011760.8	0.71459585	0.66345	0.76863813	0.78231442	0.6533615	0.9354796
183481.78	0.69216686	0.5770283	0.82354724	0.74614054	0.47646224	1.1563843
83302.656	0.76828283	0.5916714	0.98108	0.74146467	0.42049509	1.3065877
906785	0.6120525	0.5621847	0.66515768	0.64260894	0.51753271	0.7946851
246008.41	0.55689156	0.4675472	0.65833688	0.60057545	0.38518545	0.9205695
833709	0.46658969	0.4213691	0.51534104	0.5836547	0.4510825	0.7476451
1128464.3	0.50245279	0.4619412	0.54556549	0.54290229	0.44036028	0.6672387
684950.44	0.57960397	0.5239847	0.63951999	0.60555875	0.47162607	0.7756917
186138.05	0.76287466	0.6425629	0.89917171	0.83209378	0.54537141	1.2571812
1011911.6	0.77773589	0.7243412	0.83402514	0.85421854	0.72052771	1.0120617
184024.09	0.88575357	0.7549938	1.0326535	0.94051796	0.63739657	1.3834277
99317.641	0.66453451	0.5139509	0.84545088	0.73768121	0.39465538	1.3503139
911678	0.63070518	0.5802004	0.68442917	0.6638518	0.53880596	0.8157576
212900.95	0.64818877	0.544558	0.76580089	0.59993941	0.3947126	0.8963522
841973.94	0.49170169	0.4454723	0.54142535	0.61339211	0.47725165	0.7804416
1140280.3	0.55688065	0.5144037	0.60192978	0.6069988	0.50255948	0.7329844
685437	0.63609058	0.5777774	0.69869429	0.66442454	0.52244282	0.8423674
162813.19	0.88444924	0.7458943	1.0412763	0.82948035	0.55619609	1.221768
1016404.3	0.81758809	0.7629376	0.87511945	0.88521445	0.7475611	1.0468212
185641.02	0.58715475	0.4821152	0.70828336	0.62471217	0.38238877	1.006332
85777.859	0.64119107	0.4830329	0.83459836	0.61080736	0.33182165	1.1286411
919781	0.6153639	0.5657055	0.66821343	0.64297593	0.52276254	0.7898809
250479.83	0.56291962	0.4738419	0.66387755	0.6000914	0.38609588	0.9169749
858229.13	0.45908484	0.4148676	0.50673163	0.56950063	0.43898603	0.7303528
1155799.3	0.56930304	0.5266293	0.61451381	0.61639684	0.51234943	0.7417342
686117.5	0.71999329	0.6578937	0.78637511	0.73932397	0.59144562	0.9231218
188225	0.81816977	0.6940525	0.95807821	0.88479036	0.60823542	1.3004404
1031340.7	0.77471972	0.721926	0.83035326	0.83483881	0.70531571	0.9880936
190097.38	0.75224608	0.6340081	0.88613504	0.79605943	0.52535892	1.2023247
89037.484	0.60648614	0.4556111	0.79133284	0.57222933	0.28780684	1.0843967

941728.75	0.69446748	0.6422558	0.74979299	0.72085935	0.59354645	0.8741091
254069.78	0.58251715	0.4924506	0.6842885	0.62812269	0.41959998	0.9381756
888388.94	0.46938899	0.4254126	0.51667702	0.60623163	0.47698161	0.7649031
1175984.3	0.64116502	0.5962108	0.68861061	0.68517011	0.57380807	0.8173091
692302.56	0.69478291	0.6340717	0.75973892	0.70800287	0.56627625	0.8854398
189853.97	0.9322955	0.8000046	1.0802149	0.99347174	0.69257849	1.4298203
1049229.4	0.83585149	0.7814403	0.89305264	0.8962869	0.76241118	1.0533906
195332.06	0.7013697	0.5888462	0.82913369	0.72270674	0.46419102	1.1126158
106826.64	0.5522967	0.4204338	0.71242249	0.58623803	0.28811121	1.132943
965246.63	0.69930315	0.6475364	0.75410724	0.71514314	0.58835953	0.8671867
260486.94	0.74475908	0.6436407	0.8572557	0.79840857	0.56226498	1.1324456
934682.5	0.43544197	0.3941603	0.47987181	0.56153578	0.43981996	0.711225
1199969.6	0.68668401	0.6405925	0.7352159	0.72773188	0.61405349	0.8616991
700812.69	0.69490749	0.6345521	0.75945574	0.69422752	0.55008423	0.8735279
190465.81	0.86629719	0.7391558	1.0090303	0.90080953	0.62866503	1.3102916
1057645.5	0.85472876	0.799912	0.91231269	0.89741135	0.76376015	1.0540941
198483.91	0.69023234	0.5794956	0.8159675	0.70002729	0.44648099	1.0833338
108827.77	0.90969425	0.7393546	1.1075201	0.95851374	0.5860216	1.5727718
973795.56	0.73834801	0.6853581	0.79434747	0.74835533	0.6221019	0.8997521
263649.38	0.6220383	0.5304787	0.72486258	0.65735483	0.45348677	0.9597895
978135.5	0.54184723	0.4966927	0.59000403	0.68769407	0.55382371	0.848125
1214182.8	0.70005935	0.6537819	0.74874812	0.72650427	0.61321342	0.8598886
709381.13	0.76404625	0.7010684	0.83116311	0.75395042	0.60694987	0.9357918
191880.41	0.8651222	0.738521	1.007199	0.89802563	0.62327921	1.3086215
1067838	0.91680574	0.8602685	0.97608268	0.96061748	0.82529455	1.1187601
177702.64	0.81596988	0.6885652	0.96011412	0.72141802	0.4856787	1.0668027
97328.469	0.80140996	0.6334813	1.0001959	0.72371858	0.40108958	1.2496734
986460.69	0.71974486	0.6677698	0.77469099	0.72102499	0.59920985	0.8677197
266888.81	0.61823493	0.5275002	0.72009677	0.63935339	0.4294402	0.9452999
1018456.3	0.53315985	0.4892528	0.57994974	0.68511814	0.55473715	0.8410358
1231890.6	0.79390168	0.7449189	0.84525919	0.81482363	0.69609213	0.9533196
716966	0.78246391	0.7190462	0.84997582	0.76004577	0.61340177	0.9412118
192397.55	0.68088186	0.5692839	0.8079623	0.69745851	0.44814441	1.0816543
1078676.1	0.95765537	0.9001391	1.0178831	0.98592305	0.84754473	1.1468699
196445.02	0.79411536	0.6743898	0.92896879	0.78607029	0.53410983	1.1711559
109918.24	0.77330202	0.6176862	0.95619994	0.80359292	0.47389948	1.3751587
986869.75	0.74579245	0.6928786	0.80167556	0.73920029	0.61276054	0.8904527
264019.25	0.64010483	0.5472345	0.7442199	0.66257501	0.44766048	0.9745807
1054176.5	0.461972	0.4218479	0.50488347	0.60192382	0.48390332	0.744713
1244304	0.72409958	0.6775846	0.77296668	0.73778456	0.62591445	0.8692891
712238.19	0.73851699	0.6767446	0.80441266	0.71550906	0.57524103	0.8906752
1714042	4.32	4.22	4.42	3.86	3.59	4.14
1727733	3.87	3.78	3.97	3.45	3.2	3.72
1743113	3.31	3.23	3.4	2.95	2.72	3.2
1761683	2.9	2.82	2.98	2.6	2.38	2.83
1779152	3	2.92	3.08	2.68	2.46	2.92
1793333	3.28	3.19	3.36	2.93	2.7	3.18
1804833	3.12	3.04	3.21	2.83	2.6	3.07
1814318	3.01	2.93	3.09	2.71	2.49	2.95
1824603	3.09	3.01	3.18	2.79	2.56	3.03
1831677	2.82	2.74	2.89	2.57	2.35	2.8
1843186	2.95	2.87	3.03	2.69	2.47	2.93
1854943	2.93	2.86	3.01	2.7	2.48	2.94
1866042	3.08	3	3.16	2.85	2.62	3.09
1875178	3.08	3	3.16	2.88	2.64	3.12
1886259	3.02	2.94	3.1	2.86	2.63	3.1
1898519	3.24	3.16	3.32	3.07	2.83	3.32
1900523	1.93	1.87	2	1.85	1.67	2.05
1904564	1.96	1.89	2.02	1.87	1.69	2.07
1910543	2.81	2.74	2.89	2.66	2.44	2.9
875791	4.1	3.96	4.23	3.59	3.23	3.98
838251	4.55	4.4	4.69	4.12	3.73	4.54
882432	3.7	3.57	3.82	3.27	2.93	3.64

845301	4.05	3.91	4.18	3.63	3.27	4.02
890003	3.16	3.05	3.28	2.8	2.48	3.15
853110	3.47	3.34	3.59	3.11	2.77	3.48
899427	2.88	2.77	2.99	2.57	2.27	2.9
862256	2.92	2.8	3.03	2.62	2.31	2.96
908154	2.94	2.83	3.05	2.63	2.32	2.97
870998	3.05	2.93	3.16	2.74	2.43	3.08
914771	3.2	3.08	3.32	2.84	2.52	3.19
878562	3.35	3.23	3.47	3.02	2.69	3.38
920298	3.04	2.93	3.15	2.73	2.42	3.07
884535	3.21	3.09	3.33	2.92	2.59	3.27
924996	3.05	2.93	3.16	2.75	2.43	3.09
889322	2.96	2.85	3.08	2.67	2.36	3.01
929736	3.14	3.02	3.25	2.82	2.5	3.17
894867	3.05	2.93	3.16	2.76	2.44	3.11
933881	2.73	2.63	2.84	2.48	2.18	2.81
897796	2.9	2.79	3.01	2.66	2.35	3
939599	2.88	2.77	2.99	2.64	2.33	2.98
903587	3.02	2.9	3.13	2.75	2.43	3.09
944773	2.86	2.75	2.97	2.64	2.33	2.98
910170	3.01	2.89	3.12	2.76	2.44	3.11
949668	3.02	2.91	3.13	2.8	2.48	3.15
916374	3.14	3.02	3.25	2.88	2.56	3.23
953715	2.94	2.83	3.05	2.76	2.44	3.11
921463	3.22	3.11	3.34	2.99	2.66	3.35
958861	2.86	2.75	2.97	2.73	2.42	3.07
927398	3.18	3.06	3.29	2.99	2.66	3.35
964669	3.05	2.94	3.16	2.91	2.59	3.26
933850	3.43	3.31	3.55	3.23	2.89	3.6
965409	1.79	1.71	1.88	1.72	1.47	2
935114	2.08	1.99	2.17	1.98	1.71	2.28
967750	1.85	1.77	1.94	1.78	1.53	2.06
936814	2.06	1.97	2.15	1.96	1.7	2.25
970596	2.69	2.58	2.79	2.55	2.25	2.88
939947	2.94	2.83	3.05	2.77	2.45	3.12
108549	18.51	17.7	19.32	18.4	15.84	21.26
119550	8.67	8.14	9.19	8.64	6.99	10.56
126883	4.78	4.4	5.16	4.73	3.53	6.2
133643	3.12	2.82	3.42	3.09	2.14	4.32
226501	2.83	2.61	3.05	2.83	2.2	3.59
254852	3.14	2.93	3.36	3.15	2.51	3.89
237343	3.42	3.19	3.66	3.39	2.75	4.15
193599	3.24	2.99	3.5	3.22	2.58	3.97
149397	2.57	2.31	2.83	2.52	1.91	3.26
163725	0.44	0.34	0.54	0.43	0.22	0.75
109168	16.6	15.83	17.36	16.5	14.08	19.22
118318	7.98	7.47	8.49	7.91	6.33	9.76
125257	4.54	4.17	4.92	4.55	3.37	5.99
132616	2.74	2.46	3.03	2.73	1.84	3.89
231787	2.77	2.55	2.98	2.75	2.13	3.5
252011	2.96	2.74	3.17	2.96	2.35	3.69
244100	2.87	2.66	3.08	2.86	2.27	3.56
195659	2.68	2.45	2.91	2.67	2.09	3.36
153177	2.17	1.93	2.4	2.13	1.58	2.82
165640	0.38	0.29	0.47	0.36	0.17	0.66
110804	14.97	14.25	15.69	14.9	12.6	17.49
116683	7.41	6.92	7.91	7.36	5.85	9.15
123667	3.95	3.6	4.3	3.91	2.83	5.27
131490	2.14	1.89	2.39	2.18	1.4	3.25
237906	2.06	1.88	2.25	2.04	1.51	2.7
249501	2.33	2.14	2.52	2.33	1.79	2.99
249332	2.41	2.21	2.6	2.39	1.85	3.04
198619	2.27	2.06	2.48	2.26	1.73	2.9

156788	1.84	1.63	2.06	1.83	1.32	2.47
168323	0.42	0.32	0.52	0.39	0.2	0.7
113714	12.87	12.21	13.53	12.8	10.68	15.22
114551	6.25	5.79	6.71	6.18	4.8	7.84
123294	3.56	3.23	3.89	3.55	2.52	4.85
130080	2.01	1.77	2.26	2	1.25	3.03
245180	1.85	1.68	2.02	1.83	1.33	2.46
247669	1.87	1.7	2.04	1.89	1.41	2.48
254153	2.09	1.91	2.27	2.07	1.57	2.68
200824	2.06	1.86	2.26	2.07	1.57	2.69
161251	1.74	1.53	1.94	1.74	1.24	2.37
170967	0.48	0.38	0.58	0.46	0.25	0.79
117855	12.41	11.78	13.05	12.3	10.22	14.68
112738	6.88	6.4	7.37	6.82	5.36	8.55
122887	3.7	3.36	4.04	3.73	2.67	5.06
128307	2.06	1.81	2.31	2.09	1.33	3.14
250434	1.78	1.62	1.95	1.79	1.3	2.41
245595	2.09	1.91	2.27	2.07	1.57	2.69
258054	2.35	2.16	2.54	2.36	1.82	3
204338	2.08	1.89	2.28	2.07	1.57	2.69
165276	1.77	1.57	1.98	1.78	1.28	2.42
173668	0.5	0.4	0.61	0.5	0.27	0.84
120954	14.02	13.35	14.69	13.9	11.69	16.41
111280	8.36	7.82	8.89	8.36	6.74	10.26
122151	3.99	3.63	4.34	4	2.91	5.37
127439	2.02	1.77	2.26	2	1.25	3.03
252811	1.7	1.54	1.86	1.71	1.23	2.32
244060	2.17	1.98	2.35	2.15	1.63	2.78
260886	2.47	2.28	2.66	2.46	1.92	3.12
208054	2.26	2.06	2.47	2.26	1.73	2.9
169161	1.95	1.74	2.16	1.96	1.43	2.62
176537	0.59	0.48	0.7	0.57	0.33	0.93
123590	12.24	11.63	12.86	12.2	10.13	14.57
110969	7.51	7	8.02	7.45	5.93	9.25
120587	3.48	3.15	3.82	3.45	2.44	4.74
126241	1.99	1.74	2.23	2	1.25	3.03
252150	1.87	1.7	2.04	1.88	1.37	2.51
243178	2.02	1.84	2.2	2	1.5	2.61
262821	2.47	2.28	2.66	2.46	1.92	3.12
212268	2.49	2.28	2.7	2.48	1.92	3.15
173285	2.01	1.8	2.22	2	1.46	2.67
179744	0.74	0.61	0.87	0.71	0.44	1.1
126094	12.2	11.59	12.81	12.1	10.04	14.46
111821	7.74	7.23	8.26	7.73	6.17	9.55
118348	3.24	2.92	3.57	3.27	2.29	4.53
125008	1.78	1.54	2.01	1.82	1.11	2.81
250043	1.72	1.55	1.88	1.71	1.23	2.32
241937	1.91	1.74	2.08	1.89	1.41	2.48
263633	2.24	2.06	2.42	2.25	1.73	2.88
217643	2.24	2.04	2.44	2.26	1.73	2.9
177080	1.87	1.67	2.08	1.87	1.35	2.52
182711	0.78	0.65	0.91	0.79	0.49	1.19
127052	11.96	11.35	12.56	11.9	9.86	14.24
114316	8.25	7.72	8.78	8.18	6.58	10.06
115660	3.98	3.61	4.34	4	2.91	5.37
124160	1.99	1.74	2.24	2	1.25	3.03
248954	1.66	1.5	1.82	1.67	1.19	2.27
240731	1.85	1.68	2.02	1.85	1.37	2.44
263387	2.29	2.11	2.47	2.29	1.76	2.92
223861	2.19	2	2.39	2.19	1.66	2.82
179658	1.98	1.77	2.18	1.96	1.43	2.62
186824	0.91	0.77	1.05	0.89	0.58	1.32
126151	9.65	9.11	10.19	9.6	7.78	11.72

118060	7.63	7.13	8.13	7.64	6.09	9.45
113223	3.29	2.96	3.63	3.27	2.29	4.53
123259	1.53	1.31	1.74	1.55	0.9	2.47
246301	1.75	1.58	1.91	1.75	1.26	2.37
239582	1.99	1.81	2.17	1.96	1.47	2.57
261791	2.04	1.86	2.21	2.04	1.54	2.64
229937	2.26	2.07	2.46	2.26	1.73	2.9
181370	2.02	1.81	2.22	2	1.46	2.67
192003	0.81	0.68	0.94	0.79	0.49	1.19
125137	9.87	9.32	10.42	9.8	7.96	11.94
121288	7.36	6.88	7.85	7.36	5.85	9.15
112125	4.01	3.64	4.38	4	2.91	5.37
122426	2.12	1.86	2.37	2.09	1.33	3.14
245097	1.93	1.76	2.11	1.92	1.4	2.56
240452	2	1.83	2.18	2	1.5	2.61
259077	2.25	2.07	2.44	2.25	1.73	2.88
236416	2.14	1.96	2.33	2.15	1.63	2.78
182957	2.03	1.83	2.24	2.04	1.5	2.72
198211	0.91	0.78	1.05	0.89	0.58	1.32
124548	8.86	8.34	9.39	8.8	7.06	10.84
123774	7.79	7.3	8.28	7.73	6.17	9.55
112434	4.06	3.68	4.43	4.09	2.98	5.47
120915	2.2	1.94	2.46	2.18	1.4	3.25
243687	1.86	1.69	2.03	1.83	1.33	2.46
243021	1.86	1.69	2.04	1.85	1.37	2.44
255483	2.28	2.09	2.46	2.29	1.76	2.92
242907	2.4	2.2	2.59	2.41	1.86	3.07
184864	2	1.8	2.21	2	1.46	2.67
203310	1.04	0.9	1.18	1.04	0.69	1.49
123778	9.6	9.05	10.14	9.6	7.78	11.72
126248	8.08	7.58	8.58	8.09	6.5	9.96
113665	4.29	3.91	4.67	4.27	3.14	5.68
118772	2.05	1.79	2.3	2.09	1.33	3.14
241298	1.81	1.64	1.98	1.79	1.3	2.41
246003	2.13	1.95	2.32	2.11	1.6	2.74
251840	2.41	2.22	2.61	2.39	1.85	3.04
247882	2.28	2.09	2.47	2.3	1.76	2.94
187859	2.26	2.04	2.47	2.26	1.69	2.96
208697	1.18	1.03	1.33	1.18	0.81	1.66
121936	8.28	7.77	8.79	8.2	6.52	10.18
127777	8.26	7.76	8.75	8.27	6.66	10.16
116148	4.17	3.8	4.54	4.18	3.06	5.58
115556	1.88	1.63	2.13	1.91	1.18	2.92
239052	1.98	1.8	2.16	2	1.47	2.65
249272	2.4	2.21	2.6	2.41	1.86	3.07
248218	2.44	2.24	2.63	2.43	1.89	3.08
252123	2.49	2.3	2.69	2.48	1.92	3.15
190282	2.32	2.11	2.54	2.3	1.73	3.01
214814	1.22	1.08	1.37	1.25	0.87	1.74
120569	7.11	6.63	7.58	7.1	5.55	8.96
127409	7.21	6.74	7.67	7.18	5.69	8.95
119763	3.9	3.55	4.25	3.91	2.83	5.27
113854	2.1	1.83	2.37	2.09	1.33	3.14
237325	1.98	1.81	2.16	2	1.47	2.65
252683	2.3	2.11	2.48	2.3	1.76	2.94
245219	2.61	2.41	2.81	2.61	2.04	3.28
255551	2.83	2.62	3.04	2.81	2.22	3.52
193998	2.58	2.35	2.8	2.57	1.95	3.31
219888	1.37	1.21	1.52	1.36	0.96	1.86
119070	6.89	6.42	7.36	6.9	5.37	8.73
126678	8.19	7.7	8.69	8.18	6.58	10.06
122777	4.95	4.56	5.35	4.91	3.69	6.41
112828	2.36	2.07	2.64	2.36	1.54	3.46

236356	2.31	2.11	2.5	2.29	1.73	2.98
254122	2.36	2.17	2.55	2.33	1.79	2.99
244229	2.65	2.44	2.85	2.64	2.08	3.32
258416	2.78	2.58	2.99	2.78	2.18	3.48
198052	2.81	2.57	3.04	2.78	2.14	3.55
225991	1.54	1.38	1.7	1.54	1.11	2.07
116140	3.77	3.42	4.12	3.8	2.69	5.22
125694	4.53	4.15	4.9	4.55	3.37	5.99
124750	2.58	2.3	2.86	2.55	1.69	3.68
113097	1.33	1.11	1.54	1.36	0.76	2.25
230523	1.61	1.44	1.77	1.62	1.16	2.22
252755	1.66	1.5	1.82	1.67	1.22	2.23
244284	1.83	1.66	2	1.82	1.36	2.39
259977	1.67	1.51	1.83	1.67	1.22	2.23
202606	1.6	1.43	1.78	1.61	1.13	2.22
230697	0.88	0.75	1	0.89	0.58	1.32
113032	4.6	4.21	5	4.6	3.37	6.14
124536	5.03	4.64	5.43	5	3.77	6.51
127130	2.16	1.91	2.42	2.18	1.4	3.25
113778	1.33	1.12	1.54	1.36	0.76	2.25
226247	1.91	1.73	2.09	1.92	1.4	2.56
253060	1.77	1.61	1.93	1.78	1.31	2.36
243479	1.66	1.5	1.83	1.68	1.23	2.23
260149	1.6	1.45	1.76	1.59	1.15	2.15
207874	1.34	1.18	1.49	1.35	0.92	1.91
235279	0.73	0.62	0.84	0.71	0.44	1.1
111932	7.04	6.55	7.53	7	5.46	8.84
123658	9.1	8.57	9.63	9.09	7.4	11.06
129289	3.76	3.42	4.09	3.73	2.67	5.06
116164	2.1	1.84	2.36	2.09	1.33	3.14
220634	2.18	1.99	2.37	2.21	1.65	2.89
253615	1.95	1.78	2.12	1.93	1.44	2.53
243559	2.05	1.87	2.23	2.04	1.54	2.64
259587	2.14	1.96	2.32	2.15	1.63	2.78
213167	1.96	1.77	2.15	1.96	1.43	2.62
238938	1.19	1.05	1.33	1.18	0.81	1.66
55866	21.75	20.53	22.97	21.8	17.9	26.3
61456	10.15	9.36	10.95	10.18	7.69	13.22
64748	4.94	4.4	5.48	4.91	3.24	7.14
68498	2.8	2.41	3.2	2.73	1.53	4.5
113061	2.54	2.24	2.83	2.5	1.69	3.57
125436	2.4	2.13	2.67	2.37	1.62	3.35
117133	2.51	2.22	2.8	2.5	1.74	3.48
95959	2.19	1.89	2.48	2.22	1.5	3.17
71575	1.75	1.44	2.05	1.74	1.06	2.69
64519	0.31	0.17	0.45	0.29	0.08	0.73
56051	19.75	18.59	20.91	19.8	16.09	24.11
60900	8.87	8.12	9.61	8.91	6.59	11.78
63980	4.7	4.17	5.24	4.73	3.09	6.93
67871	2.12	1.78	2.47	2.18	1.13	3.81
115711	2.14	1.88	2.41	2.17	1.42	3.17
124040	2.51	2.23	2.79	2.52	1.74	3.52
120305	2.26	1.99	2.53	2.29	1.56	3.23
97186	2.14	1.85	2.43	2.15	1.44	3.09
73582	1.54	1.25	1.82	1.57	0.93	2.47
65675	0.29	0.16	0.42	0.29	0.08	0.73
56802	18.03	16.92	19.13	18	14.47	22.13
59938	7.87	7.16	8.59	7.82	5.66	10.53
63296	4.12	3.62	4.62	4.18	2.65	6.27
67301	1.72	1.41	2.04	1.64	0.75	3.11
118599	1.59	1.36	1.81	1.58	0.95	2.47
122759	1.77	1.53	2	1.78	1.14	2.65
122830	1.82	1.58	2.06	1.86	1.21	2.72

98881	1.82	1.55	2.09	1.85	1.2	2.73
75484	1.51	1.23	1.79	1.48	0.86	2.37
67220	0.3	0.17	0.43	0.29	0.08	0.73
58442	15.01	14.01	16	15	11.8	18.8
58872	7.03	6.35	7.71	7.09	5.04	9.69
63022	4.09	3.59	4.59	4.18	2.65	6.27
66472	1.78	1.45	2.1	1.82	0.87	3.34
121951	1.57	1.34	1.79	1.58	0.95	2.47
121704	1.71	1.48	1.94	1.7	1.08	2.56
125257	1.67	1.44	1.89	1.64	1.04	2.47
100117	1.72	1.46	1.97	1.7	1.08	2.56
77735	1.43	1.16	1.69	1.39	0.8	2.26
68684	0.44	0.28	0.59	0.43	0.16	0.93
60564	15.01	14.03	15.98	15	11.8	18.8
57892	7.76	7.04	8.47	7.82	5.66	10.53
62948	3.64	3.17	4.11	3.64	2.22	5.62
65458	1.7	1.38	2.01	1.64	0.75	3.11
124424	1.46	1.25	1.68	1.5	0.89	2.37
120498	1.78	1.55	2.02	1.78	1.14	2.65
127193	1.96	1.71	2.2	1.93	1.27	2.81
101989	1.79	1.53	2.05	1.78	1.14	2.65
79700	1.47	1.2	1.73	1.48	0.86	2.37
70332	0.4	0.25	0.55	0.43	0.16	0.93
62084	16.59	15.58	17.6	16.6	13.22	20.58
57246	9.17	8.39	9.96	9.09	6.75	11.99
62649	4.13	3.63	4.64	4.18	2.65	6.27
64958	1.66	1.35	1.98	1.64	0.75	3.11
126008	1.44	1.23	1.65	1.42	0.83	2.27
119498	1.76	1.52	2	1.78	1.14	2.65
128509	1.98	1.73	2.22	2	1.33	2.89
103764	1.89	1.62	2.15	1.93	1.26	2.82
81820	1.6	1.33	1.88	1.57	0.93	2.47
72026	0.46	0.3	0.61	0.43	0.16	0.93
63311	14.53	13.59	15.47	14.6	11.44	18.36
56988	8.53	7.77	9.29	8.55	6.28	11.36
61967	3.97	3.47	4.47	4	2.51	6.06
64465	1.69	1.37	2.01	1.64	0.75	3.11
125678	1.5	1.28	1.71	1.5	0.89	2.37
119051	1.67	1.44	1.9	1.7	1.08	2.56
129385	1.93	1.69	2.17	1.93	1.27	2.81
105734	2.12	1.84	2.4	2.15	1.44	3.09
84044	1.69	1.41	1.97	1.65	0.99	2.58
73912	0.46	0.31	0.61	0.43	0.16	0.93
64543	14.58	13.65	15.51	14.6	11.44	18.36
57415	8.85	8.08	9.62	8.91	6.59	11.78
60751	3.49	3.02	3.96	3.45	2.08	5.39
63824	1.43	1.13	1.72	1.45	0.63	2.87
124766	1.37	1.17	1.58	1.33	0.76	2.17
118254	1.74	1.5	1.98	1.78	1.14	2.65
129619	1.96	1.72	2.2	1.93	1.27	2.81
108322	2.15	1.87	2.43	2.15	1.44	3.09
86136	1.61	1.35	1.88	1.65	0.99	2.58
75692	0.83	0.63	1.04	0.86	0.44	1.5
65012	14.49	13.56	15.41	14.4	11.27	18.13
58737	9.72	8.92	10.52	9.64	7.22	12.6
59344	4.13	3.61	4.65	4.18	2.65	6.27
63306	1.71	1.38	2.03	1.64	0.75	3.11
124397	1.43	1.22	1.64	1.42	0.83	2.27
117505	1.7	1.47	1.94	1.7	1.08	2.56
129624	1.89	1.65	2.13	1.86	1.21	2.72
111220	2.01	1.74	2.27	2	1.32	2.91
87636	1.6	1.33	1.86	1.57	0.93	2.47
78086	0.82	0.62	1.02	0.79	0.39	1.41

64436	11.56	10.73	12.39	11.6	8.81	15
60629	8.54	7.81	9.28	8.55	6.28	11.36
57981	3.28	2.81	3.74	3.27	1.94	5.17
63072	1.28	1	1.56	1.27	0.51	2.62
122515	1.41	1.2	1.62	1.42	0.83	2.27
116700	1.73	1.49	1.97	1.7	1.08	2.56
128513	1.81	1.57	2.04	1.79	1.16	2.64
114207	1.89	1.64	2.14	1.93	1.26	2.82
88693	1.58	1.32	1.84	1.57	0.93	2.47
81050	0.67	0.49	0.84	0.64	0.29	1.22
63910	11.47	10.64	12.3	11.4	8.63	14.77
62203	8.83	8.09	9.56	8.91	6.59	11.78
57403	4.49	3.95	5.04	4.55	2.94	6.71
62790	1.72	1.4	2.04	1.64	0.75	3.11
121943	1.44	1.22	1.65	1.42	0.83	2.27
117009	1.82	1.58	2.06	1.85	1.2	2.73
127019	1.83	1.6	2.07	1.86	1.21	2.72
117081	1.84	1.59	2.08	1.85	1.2	2.73
89802	1.76	1.49	2.03	1.74	1.06	2.69
84427	0.79	0.6	0.98	0.79	0.39	1.41
63607	10.39	9.6	11.18	10.4	7.77	13.64
63401	8.85	8.12	9.58	8.91	6.59	11.78
57456	4	3.49	4.52	4	2.51	6.06
62060	2	1.65	2.35	2	1	3.58
121543	1.51	1.3	1.73	1.5	0.89	2.37
118342	1.66	1.43	1.9	1.63	1.02	2.47
125430	1.97	1.72	2.21	2	1.33	2.89
119985	2.14	1.88	2.4	2.15	1.44	3.09
91064	1.79	1.52	2.06	1.83	1.13	2.79
87282	0.92	0.72	1.12	0.93	0.49	1.59
63502	11.13	10.31	11.95	11.2	8.46	14.54
64567	9.01	8.28	9.75	9.09	6.75	11.99
58155	4.42	3.88	4.96	4.36	2.8	6.49
60810	1.6	1.28	1.91	1.64	0.75	3.11
120699	1.44	1.23	1.66	1.42	0.83	2.27
119841	1.94	1.69	2.19	1.93	1.26	2.82
123522	2.17	1.91	2.43	2.14	1.45	3.06
122286	2.17	1.91	2.43	2.15	1.44	3.09
92741	1.95	1.67	2.24	1.91	1.2	2.9
90251	1.16	0.94	1.39	1.14	0.65	1.86
62562	9.54	8.78	10.31	9.6	7.08	12.73
65300	9.43	8.69	10.18	9.45	7.06	12.4
59586	4.33	3.8	4.86	4.36	2.8	6.49
59183	1.35	1.06	1.65	1.27	0.51	2.62
119801	1.44	1.22	1.65	1.42	0.83	2.27
121291	1.96	1.71	2.21	1.93	1.26	2.82
121820	2.14	1.88	2.4	2.14	1.45	3.06
124179	2.08	1.82	2.33	2.07	1.38	3
94146	2.1	1.81	2.4	2.09	1.34	3.11
93595	1.37	1.13	1.6	1.36	0.82	2.12
61939	8.2	7.49	8.91	8.2	5.88	11.12
65047	8.09	7.4	8.78	8	5.81	10.74
61433	4.09	3.58	4.59	4	2.51	6.06
58282	1.63	1.3	1.96	1.64	0.75	3.11
119349	1.48	1.26	1.7	1.5	0.89	2.37
122871	1.99	1.74	2.24	2	1.32	2.91
120272	2.19	1.92	2.45	2.21	1.5	3.14
125814	2.62	2.34	2.91	2.59	1.81	3.61
95988	2.36	2.06	2.67	2.35	1.55	3.42
96403	1.27	1.04	1.49	1.29	0.76	2.03
61124	7.66	6.96	8.35	7.6	5.38	10.43
64712	9.43	8.68	10.17	9.45	7.06	12.4
62929	4.88	4.33	5.42	4.91	3.24	7.14

57738	2.04	1.67	2.41	2	1	3.58
119201	1.91	1.66	2.16	1.92	1.22	2.88
123690	1.95	1.7	2.19	1.93	1.26	2.82
119662	2.17	1.91	2.44	2.14	1.45	3.06
127197	2.57	2.29	2.85	2.59	1.81	3.61
97818	2.41	2.1	2.72	2.43	1.62	3.52
99779	1.46	1.23	1.7	1.43	0.87	2.21
59686	4.47	3.94	5.01	4.4	2.76	6.66
64210	5.5	4.92	6.07	5.45	3.68	7.79
63893	2.75	2.35	3.16	2.73	1.53	4.5
57842	1.02	0.76	1.28	1.09	0.4	2.37
116665	1.23	1.03	1.44	1.25	0.7	2.06
122751	1.35	1.15	1.56	1.33	0.79	2.11
119626	1.38	1.17	1.59	1.36	0.82	2.12
127979	1.4	1.19	1.6	1.41	0.85	2.2
100020	1.29	1.07	1.51	1.3	0.73	2.15
102442	0.91	0.72	1.09	0.93	0.49	1.59
58049	5.32	4.73	5.92	5.4	3.56	7.86
63779	5.91	5.31	6.51	6	4.13	8.43
65027	2.17	1.81	2.53	2.18	1.13	3.81
58394	1.03	0.77	1.29	1.09	0.4	2.37
114816	1.42	1.2	1.64	1.42	0.83	2.27
122632	1.52	1.3	1.73	1.48	0.9	2.29
118929	1.56	1.33	1.78	1.57	0.98	2.38
127757	1.36	1.16	1.56	1.33	0.79	2.11
102352	1.22	1.01	1.44	1.22	0.67	2.04
105079	0.69	0.53	0.84	0.71	0.34	1.31
57367	7.36	6.65	8.06	7.4	5.21	10.2
63324	10.23	9.45	11.02	10.18	7.69	13.22
66103	4.13	3.64	4.62	4.18	2.65	6.27
59940	1.9	1.55	2.25	1.82	0.87	3.34
112538	1.57	1.34	1.8	1.58	0.95	2.47
122874	1.8	1.56	2.04	1.78	1.14	2.65
118525	1.49	1.27	1.71	1.5	0.93	2.29
127371	1.94	1.7	2.18	1.93	1.26	2.82
104798	1.93	1.66	2.19	1.91	1.2	2.9
107107	1.2	0.99	1.4	1.21	0.71	1.94
52683	15.03	13.99	16.08	15	11.8	18.8
58094	7.04	6.36	7.72	7.09	5.04	9.69
62135	4.6	4.07	5.14	4.55	2.94	6.71
65145	3.45	3	3.91	3.45	2.08	5.39
113440	3.12	2.8	3.45	3.08	2.17	4.25
129416	3.86	3.52	4.2	3.85	2.88	5.05
120210	4.31	3.94	4.68	4.29	3.27	5.52
97640	4.28	3.87	4.69	4.3	3.26	5.55
77822	3.33	2.92	3.73	3.3	2.34	4.54
99206	0.52	0.38	0.67	0.5	0.2	1.03
53117	13.2	12.22	14.17	13.2	10.21	16.79
57418	6.98	6.3	7.67	6.91	4.89	9.48
61277	4.36	3.83	4.88	4.36	2.8	6.49
64745	3.4	2.95	3.85	3.45	2.08	5.39
116076	3.39	3.05	3.72	3.42	2.45	4.64
127971	3.38	3.06	3.7	3.41	2.49	4.55
123795	3.46	3.13	3.78	3.43	2.53	4.55
98473	3.21	2.86	3.56	3.19	2.31	4.29
79595	2.75	2.39	3.12	2.78	1.9	3.93
99965	0.44	0.31	0.57	0.43	0.16	0.93
54002	11.68	10.77	12.6	11.6	8.81	15
56745	6.93	6.24	7.61	6.91	4.89	9.48
60371	3.76	3.27	4.25	3.82	2.36	5.84
64189	2.57	2.18	2.96	2.55	1.39	4.27
119307	2.54	2.25	2.83	2.5	1.69	3.57
126742	2.88	2.58	3.18	2.89	2.05	3.95

126502	2.97	2.67	3.27	3	2.16	4.06
99738	2.72	2.39	3.04	2.74	1.93	3.78
81304	2.15	1.83	2.47	2.17	1.41	3.21
101103	0.5	0.37	0.64	0.5	0.2	1.03
55272	10.62	9.76	11.48	10.6	7.94	13.87
55679	5.37	4.76	5.98	5.45	3.68	7.79
60272	3	2.57	3.44	3.09	1.8	4.95
63608	2.26	1.89	2.63	2.18	1.13	3.81
123229	2.13	1.87	2.38	2.17	1.42	3.17
125965	2.03	1.78	2.28	2	1.32	2.91
128896	2.5	2.23	2.77	2.5	1.74	3.48
100707	2.4	2.1	2.71	2.37	1.62	3.35
83516	2.02	1.72	2.33	2	1.27	3
102283	0.51	0.37	0.65	0.5	0.2	1.03
57291	9.67	8.86	10.48	9.6	7.08	12.73
54846	5.91	5.26	6.55	5.82	3.98	8.21
59939	3.75	3.26	4.24	3.82	2.36	5.84
62849	2.43	2.05	2.82	2.36	1.26	4.04
126010	2.1	1.84	2.35	2.08	1.35	3.08
125097	2.39	2.12	2.66	2.37	1.62	3.35
130861	2.73	2.45	3.01	2.71	1.92	3.73
102349	2.37	2.08	2.67	2.37	1.62	3.35
85576	2.06	1.75	2.36	2.09	1.34	3.11
103336	0.57	0.43	0.72	0.57	0.25	1.13
58870	11.28	10.42	12.14	11.2	8.46	14.54
54034	7.48	6.75	8.21	7.45	5.35	10.11
59502	3.8	3.3	4.29	3.82	2.36	5.84
62481	2.38	2	2.77	2.36	1.26	4.04
126803	1.94	1.7	2.18	1.92	1.22	2.88
124562	2.55	2.27	2.83	2.52	1.74	3.52
132377	2.95	2.66	3.25	2.93	2.1	3.97
104290	2.64	2.33	2.95	2.67	1.87	3.69
87341	2.28	1.96	2.59	2.26	1.48	3.31
104511	0.68	0.52	0.84	0.71	0.34	1.31
60279	9.84	9.05	10.63	9.8	7.25	12.96
53981	6.39	5.72	7.07	6.36	4.43	8.85
58620	2.97	2.53	3.41	2.91	1.66	4.72
61776	2.3	1.92	2.68	2.36	1.26	4.04
126472	2.25	1.98	2.51	2.25	1.48	3.27
124127	2.35	2.08	2.62	2.37	1.62	3.35
133436	2.98	2.69	3.28	3	2.16	4.06
106534	2.85	2.53	3.17	2.89	2.05	3.95
89241	2.31	1.99	2.62	2.35	1.55	3.42
105832	0.94	0.75	1.12	0.93	0.49	1.59
61551	9.7	8.92	10.48	9.6	7.08	12.73
54406	6.58	5.9	7.26	6.55	4.58	9.06
57597	2.99	2.54	3.43	2.91	1.66	4.72
61184	2.14	1.77	2.51	2.18	1.13	3.81
125277	2.06	1.81	2.31	2.08	1.35	3.08
123683	2.06	1.81	2.31	2.07	1.38	3
134014	2.51	2.25	2.78	2.5	1.74	3.48
109321	2.33	2.05	2.62	2.3	1.56	3.26
90944	2.12	1.82	2.42	2.09	1.34	3.11
107019	0.74	0.58	0.9	0.71	0.34	1.31
62040	9.3	8.54	10.06	9.4	6.91	12.5
55579	6.68	6	7.35	6.73	4.74	9.27
56316	3.82	3.31	4.33	3.82	2.36	5.84
60854	2.28	1.9	2.66	2.36	1.26	4.04
124557	1.89	1.65	2.13	1.92	1.22	2.88
123226	1.98	1.73	2.23	2	1.32	2.91
133763	2.68	2.4	2.95	2.64	1.86	3.64
112641	2.38	2.09	2.66	2.37	1.62	3.35
92022	2.34	2.02	2.65	2.35	1.55	3.42

108738	0.97	0.79	1.16	1	0.55	1.68
61715	7.65	6.96	8.34	7.6	5.38	10.43
57431	6.65	5.98	7.32	6.73	4.74	9.27
55242	3.29	2.82	3.77	3.27	1.94	5.17
60187	1.78	1.44	2.11	1.82	0.87	3.34
123786	2.07	1.81	2.32	2.08	1.35	3.08
122882	2.23	1.97	2.49	2.22	1.5	3.17
133278	2.26	2	2.51	2.29	1.56	3.23
115730	2.63	2.33	2.92	2.59	1.81	3.61
92677	2.44	2.12	2.76	2.43	1.62	3.52
110953	0.92	0.74	1.1	0.93	0.49	1.59
61227	8.2	7.48	8.92	8.2	5.88	11.12
59085	5.82	5.21	6.44	5.82	3.98	8.21
54722	3.49	3	3.99	3.45	2.08	5.39
59636	2.53	2.13	2.94	2.55	1.39	4.27
123154	2.41	2.14	2.69	2.42	1.62	3.47
123443	2.18	1.92	2.44	2.15	1.44	3.09
132058	2.66	2.38	2.94	2.64	1.86	3.64
119335	2.45	2.17	2.73	2.44	1.68	3.43
93155	2.3	1.99	2.61	2.26	1.48	3.31
113784	1	0.82	1.19	1	0.55	1.68
60941	7.27	6.59	7.95	7.2	5.04	9.97
60373	6.68	6.02	7.33	6.73	4.74	9.27
54978	4.11	3.57	4.65	4.18	2.65	6.27
58855	2.4	2	2.79	2.36	1.26	4.04
122144	2.19	1.93	2.46	2.17	1.42	3.17
124679	2.05	1.8	2.3	2.07	1.38	3
130053	2.58	2.3	2.85	2.57	1.8	3.56
122922	2.64	2.36	2.93	2.67	1.87	3.69
93800	2.21	1.91	2.51	2.17	1.41	3.21
116028	1.14	0.94	1.33	1.14	0.65	1.86
60276	7.98	7.27	8.69	8	5.72	10.89
61681	7.08	6.42	7.75	7.09	5.04	9.69
55510	4.16	3.62	4.7	4.18	2.65	6.27
57962	2.52	2.11	2.93	2.55	1.39	4.27
120599	2.18	1.92	2.44	2.17	1.42	3.17
126162	2.31	2.05	2.58	2.3	1.56	3.26
128318	2.65	2.37	2.93	2.64	1.86	3.64
125596	2.39	2.12	2.66	2.37	1.62	3.35
95118	2.55	2.23	2.88	2.52	1.69	3.62
118446	1.19	0.99	1.39	1.21	0.71	1.94
59374	6.96	6.29	7.63	7	4.88	9.74
62477	7.03	6.37	7.68	7.09	5.04	9.69
56562	4	3.47	4.52	4	2.51	6.06
56373	2.43	2.02	2.84	2.36	1.26	4.04
119251	2.52	2.24	2.81	2.5	1.69	3.57
127981	2.82	2.53	3.11	2.81	1.99	3.86
126398	2.71	2.43	3	2.71	1.92	3.73
127944	2.9	2.6	3.19	2.89	2.05	3.95
96136	2.54	2.22	2.86	2.52	1.69	3.62
121219	1.11	0.93	1.3	1.14	0.65	1.86
58630	5.95	5.33	6.58	6	4.05	8.57
62362	6.29	5.66	6.91	6.36	4.43	8.85
58330	3.7	3.21	4.2	3.64	2.22	5.62
55572	2.56	2.13	2.98	2.55	1.39	4.27
117976	2.48	2.2	2.77	2.5	1.69	3.57
129812	2.58	2.3	2.86	2.59	1.81	3.61
124947	3.01	2.71	3.31	3	2.16	4.06
129737	3.03	2.73	3.33	3.04	2.18	4.12
98010	2.79	2.46	3.12	2.78	1.9	3.93
123485	1.45	1.24	1.66	1.43	0.87	2.21
57946	6.07	5.44	6.71	6	4.05	8.57
61966	6.91	6.25	7.56	6.91	4.89	9.48

59848	5.03	4.46	5.6	5.09	3.38	7.36
55090	2.69	2.25	3.12	2.73	1.53	4.5
117155	2.7	2.4	2.99	2.67	1.82	3.76
130432	2.74	2.46	3.03	2.74	1.93	3.78
124567	3.1	2.79	3.41	3.07	2.22	4.14
131219	2.99	2.69	3.28	2.96	2.12	4.03
100234	3.19	2.84	3.54	3.22	2.27	4.43
126212	1.6	1.38	1.82	1.57	0.98	2.38
56454	3.03	2.58	3.48	3	1.68	4.95
61484	3.51	3.04	3.98	3.45	2.08	5.39
60857	2.4	2.01	2.79	2.36	1.26	4.04
55255	1.65	1.31	1.99	1.64	0.75	3.11
113858	1.98	1.72	2.23	2	1.28	2.98
130004	1.95	1.71	2.19	1.93	1.26	2.82
124658	2.25	1.99	2.52	2.29	1.56	3.23
131998	1.93	1.69	2.17	1.93	1.26	2.82
102586	1.91	1.64	2.18	1.91	1.2	2.9
128255	0.85	0.69	1.01	0.86	0.44	1.5
54983	3.84	3.32	4.36	3.8	2.29	5.93
60757	4.11	3.6	4.62	4.18	2.65	6.27
62103	2.16	1.79	2.52	2.18	1.13	3.81
55384	1.64	1.31	1.98	1.64	0.75	3.11
111431	2.4	2.11	2.68	2.42	1.62	3.47
130428	2.01	1.77	2.25	2	1.32	2.91
124550	1.77	1.53	2	1.79	1.16	2.64
132392	1.84	1.6	2.07	1.85	1.2	2.73
105522	1.45	1.22	1.68	1.48	0.86	2.37
130200	0.77	0.62	0.92	0.79	0.39	1.41
54565	6.71	6.02	7.39	6.8	4.71	9.5
60334	7.91	7.2	8.62	7.82	5.66	10.53
63186	3.37	2.92	3.82	3.45	2.08	5.39
56224	2.31	1.91	2.71	2.36	1.26	4.04
108096	2.8	2.49	3.12	2.83	1.96	3.96
130741	2.09	1.84	2.34	2.07	1.38	3
125034	2.58	2.29	2.86	2.57	1.8	3.56
132216	2.33	2.07	2.59	2.3	1.56	3.26
108369	1.99	1.73	2.26	2	1.27	3
131831	1.19	1	1.38	1.21	0.71	1.94
2351	9.38	9.01	9.77	9.63	8.36	11.03
1712	7.19	6.85	7.54	7.35	6.25	8.59
1237	2.1	1.99	2.23	1.94	1.64	2.27
2101	3.29	3.16	3.44	3.22	2.84	3.65
2092	8.41	8.05	8.78	8.6	7.41	9.94
1589	6.72	6.39	7.06	6.84	5.78	8.04
1171	1.96	1.85	2.08	1.81	1.52	2.13
1833	2.84	2.71	2.97	2.75	2.4	3.14
1873	7.57	7.23	7.92	7.72	6.59	8.99
1416	6.02	5.71	6.34	6.09	5.09	7.23
943	1.56	1.46	1.66	1.46	1.21	1.76
1541	2.35	2.24	2.47	2.28	1.96	2.64
1667	6.75	6.43	7.09	6.79	5.73	7.99
1211	5.16	4.87	5.46	5.21	4.29	6.27
921	1.5	1.4	1.6	1.41	1.16	1.7
1303	1.96	1.86	2.07	1.92	1.63	2.26
1698	6.88	6.56	7.21	6.84	5.78	8.04
1256	5.35	5.05	5.65	5.35	4.42	6.42
974	1.56	1.46	1.66	1.48	1.22	1.77
1398	2.08	1.97	2.19	2.03	1.72	2.37
1922	7.78	7.44	8.14	7.67	6.55	8.94
1443	6.14	5.83	6.47	6.14	5.14	7.28
1006	1.59	1.5	1.69	1.52	1.26	1.81
1500	2.21	2.1	2.32	2.17	1.85	2.52
1761	7.14	6.81	7.48	7.02	5.95	8.24

1254	5.34	5.05	5.65	5.26	4.33	6.32
1037	1.63	1.53	1.73	1.55	1.29	1.86
1583	2.31	2.2	2.43	2.28	1.96	2.64
1752	7.11	6.78	7.45	6.93	5.86	8.14
1258	5.36	5.07	5.66	5.26	4.33	6.32
1066	1.66	1.56	1.76	1.62	1.35	1.92
1377	1.99	1.89	2.1	1.96	1.66	2.3
1866	7.57	7.23	7.92	7.35	6.25	8.59
1302	5.55	5.25	5.86	5.44	4.5	6.52
1050	1.62	1.52	1.72	1.58	1.31	1.88
1426	2.05	1.95	2.16	2.03	1.72	2.37
1534	6.23	5.92	6.55	6.05	5.05	7.18
1143	4.87	4.59	5.16	4.79	3.91	5.81
1017	1.56	1.47	1.66	1.52	1.26	1.81
1463	2.09	1.99	2.2	2.08	1.77	2.42
1648	6.69	6.37	7.02	6.51	5.48	7.68
1188	5.06	4.78	5.36	4.93	4.04	5.96
1061	1.61	1.52	1.71	1.58	1.31	1.88
1537	2.18	2.07	2.29	2.15	1.84	2.5
1576	6.39	6.08	6.72	6.23	5.22	7.38
1213	5.16	4.87	5.46	5.07	4.16	6.12
1128	1.7	1.6	1.8	1.67	1.4	1.98
1523	2.15	2.04	2.26	2.13	1.82	2.48
1643	6.65	6.33	6.98	6.42	5.39	7.58
1295	5.5	5.21	5.81	5.4	4.46	6.47
1226	1.83	1.73	1.94	1.81	1.52	2.13
1579	2.21	2.1	2.32	2.2	1.89	2.56
1551	6.29	5.98	6.61	6.09	5.09	7.23
1215	5.17	4.89	5.47	5.07	4.16	6.12
1255	1.86	1.76	1.97	1.85	1.56	2.17
1755	2.44	2.33	2.56	2.42	2.09	2.79
1380	5.59	5.3	5.9	5.44	4.5	6.52
1099	4.68	4.41	4.96	4.6	3.74	5.61
1364	2	1.9	2.11	1.99	1.69	2.32
1849	2.55	2.44	2.67	2.55	2.21	2.93
1503	6.1	5.79	6.41	5.95	4.97	7.08
1229	5.23	4.94	5.53	5.16	4.25	6.22
1438	2.09	1.99	2.2	2.08	1.77	2.42
1974	2.7	2.59	2.83	2.7	2.35	3.09
855	3.48	3.25	3.72	3.4	2.66	4.27
624	2.67	2.46	2.88	2.65	2.01	3.43
876	1.27	1.19	1.36	1.26	1.02	1.54
1320	1.8	1.71	1.9	1.81	1.52	2.13
887	3.62	3.38	3.86	3.58	2.83	4.48
686	2.94	2.73	3.17	2.93	2.25	3.75
905	1.31	1.22	1.4	1.29	1.05	1.56
1245	1.69	1.6	1.79	1.69	1.42	2.01
1457	5.91	5.61	6.22	5.86	4.88	6.98
1186	5.06	4.78	5.36	5.02	4.12	6.06
1152	1.66	1.57	1.76	1.64	1.37	1.95
1579	2.14	2.04	2.25	2.15	1.84	2.5
744064	1.23	1.15	1.31	1.23	1.02	1.46
758576	1.21	1.13	1.29	1.21	1.01	1.44
773062	1.39	1.3	1.47	1.4	1.18	1.64
787195	1.38	1.3	1.46	1.39	1.17	1.63
801336	1.65	1.56	1.74	1.67	1.43	1.93
814638	1.61	1.52	1.7	1.62	1.39	1.88
828118	1.88	1.79	1.97	1.9	1.64	2.18
841067	1.93	1.83	2.02	1.94	1.69	2.23
853730	2.15	2.05	2.25	2.17	1.9	2.47
865101	2.35	2.25	2.45	2.4	2.11	2.71
876661	2.39	2.29	2.49	2.44	2.15	2.76
886564	2.77	2.66	2.88	2.84	2.53	3.18

896278	3.07	2.95	3.19	3.14	2.81	3.5
905437	3.15	3.04	2.27	3.21	2.88	3.57
914656	3.31	3.19	3.43	3.36	3.02	3.73
926688	3.41	3.29	3.53	3.44	3.1	3.82
937564	1.47	1.4	1.55	1.48	1.26	1.73
946781	1.47	1.4	1.55	1.48	1.26	1.73
955251	2.25	2.16	2.35	2.26	1.99	2.57
349186	1.21	1.1	1.34	1.17	0.9	1.5
394878	1.25	1.14	1.36	1.26	0.98	1.61
356748	1.23	1.12	1.35	1.21	0.93	1.54
401828	1.19	1.09	1.3	1.21	0.93	1.54
364415	1.41	1.29	1.54	1.4	1.1	1.75
408647	1.37	1.25	1.48	1.4	1.1	1.75
371793	1.48	1.36	1.61	1.47	1.16	1.84
415402	1.28	1.17	1.39	1.3	1.01	1.65
379214	1.71	1.58	1.84	1.72	1.38	2.11
422122	1.6	1.48	1.73	1.62	1.3	2
386119	1.6	1.48	1.73	1.62	1.3	2
428519	1.61	1.49	1.74	1.64	1.31	2.02
393075	1.95	1.81	2.09	1.94	1.59	2.36
435043	1.82	1.69	1.95	1.85	1.5	2.25
399769	1.95	1.81	2.09	1.96	1.6	2.38
441298	1.91	1.78	2.04	1.94	1.59	2.36
406566	2.2	2.06	2.35	2.21	1.83	2.65
447164	2.1	1.97	2.24	2.13	1.76	2.56
412463	2.42	2.28	2.58	2.47	2.07	2.93
452638	2.28	2.14	2.42	2.32	1.93	2.77
418329	2.44	2.3	2.6	2.51	2.1	2.97
458332	2.34	2.2	2.48	2.38	1.98	2.83
423761	2.93	2.77	3.09	3	2.55	3.5
462803	2.63	2.48	2.78	2.68	2.26	3.16
428800	3.33	3.16	3.5	3.42	2.94	3.95
467478	2.83	2.68	2.99	2.87	2.43	3.36
433740	3.35	3.18	3.53	3.43	2.95	3.97
471697	2.96	2.81	3.12	2.98	2.53	3.48
438477	3.51	3.33	3.69	3.57	3.08	4.11
476179	3.13	2.97	3.29	3.15	2.69	3.67
444456	3.62	3.44	3.8	3.68	3.18	4.23
482232	3.21	3.06	3.38	3.21	2.74	3.73
450067	1.59	1.47	1.71	1.6	1.28	1.98
487497	1.37	1.26	1.47	1.36	1.06	1.71
454117	1.56	1.45	1.68	1.58	1.26	1.96
492664	1.39	1.29	1.5	1.38	1.08	1.73
457801	2.37	2.23	2.52	2.4	2	2.85
497450	2.14	2.01	2.27	2.11	1.74	2.54
237343	0.9	0.78	1.02	0.89	0.58	1.32
193599	1.89	1.7	2.08	1.89	1.41	2.48
149397	1.85	1.64	2.07	1.87	1.35	2.52
163725	0.37	0.27	0.46	0.39	0.2	0.7
244100	0.8	0.69	0.91	0.79	0.49	1.19
195659	1.79	1.61	1.98	1.78	1.31	2.36
153177	1.89	1.67	2.1	1.91	1.39	2.57
165640	0.5	0.39	0.6	0.5	0.27	0.84
249332	0.84	0.73	0.96	0.86	0.55	1.28
198619	2.2	1.99	2.4	2.19	1.66	2.82
156788	2.24	2	2.47	2.26	1.69	2.96
168323	0.45	0.34	0.55	0.5	0.27	0.84
254153	0.87	0.75	0.98	0.86	0.55	1.28
200824	2	1.8	2.19	2	1.5	2.61
161251	2.23	2	2.46	2.22	1.65	2.92
170967	0.6	0.49	0.72	0.64	0.38	1.02
258054	1.01	0.89	1.13	1	0.66	1.45
204338	2.4	2.19	2.62	2.41	1.86	3.07

165276	2.57	2.33	2.82	2.57	1.95	3.31
173668	0.85	0.71	0.98	0.89	0.58	1.32
260886	0.95	0.83	1.06	0.93	0.61	1.36
208054	2.24	2.04	2.45	2.26	1.73	2.9
169161	2.71	2.46	2.96	2.7	2.07	3.46
176537	0.78	0.65	0.91	0.82	0.52	1.23
262821	1.09	0.96	1.21	1.07	0.72	1.53
212268	2.58	2.36	2.79	2.59	2.02	3.28
173285	3.16	2.9	3.43	3.17	2.49	3.99
179744	0.97	0.83	1.12	1	0.66	1.45
263633	1.13	1.01	1.26	1.14	0.78	1.61
217643	2.39	2.19	2.6	2.41	1.86	3.07
177080	3.28	3.01	3.55	3.3	2.6	4.14
182711	1.2	1.04	1.36	1.21	0.84	1.7
263387	1.19	1.06	1.32	1.18	0.81	1.66
223861	2.78	2.56	3	2.78	2.18	3.48
179658	3.56	3.29	3.84	3.57	2.84	4.43
186824	1.37	1.2	1.54	1.43	1.02	1.95
261791	1.14	1.01	1.27	1.14	0.78	1.61
229937	2.88	2.66	3.1	2.89	2.28	3.61
181370	3.78	3.49	4.06	3.78	3.03	4.67
192003	2.01	1.8	2.21	2.04	1.54	2.64
259077	1.04	0.92	1.17	1.04	0.69	1.49
236416	2.81	2.59	3.02	2.81	2.22	3.52
182957	3.94	3.65	4.22	3.96	3.19	4.86
198211	2.22	2.01	2.43	2.29	1.76	2.92
255483	1.04	0.92	1.17	1.04	0.69	1.49
242907	3.18	2.95	3.4	3.19	2.55	3.93
184864	4.68	4.37	4.99	4.7	3.85	5.67
203310	2.72	2.5	2.95	2.79	2.2	3.48
251840	1.25	1.11	1.39	1.25	0.87	1.74
247882	3.21	2.99	3.43	3.22	2.58	3.97
187859	5.25	4.92	5.58	5.26	4.37	6.29
208697	3.13	2.89	3.37	3.21	2.58	3.95
248218	1.2	1.07	1.34	1.21	0.84	1.7
252123	3.3	3.07	3.52	3.3	2.65	4.06
190282	5.02	4.7	5.34	5.04	4.17	6.05
214814	3.57	3.32	3.82	3.64	2.97	4.42
245219	1.3	1.16	1.44	1.29	0.9	1.78
255551	3.42	3.19	3.65	3.41	2.75	4.18
193998	5.06	4.74	5.37	5.04	4.17	6.05
219888	3.89	3.63	4.15	3.96	3.26	4.77
244229	1.08	0.95	1.21	1.07	0.72	1.53
258416	3.52	3.29	3.75	3.52	2.85	4.3
198052	5.44	5.12	5.77	5.43	4.52	6.48
225991	4.02	3.76	4.28	4.11	3.39	4.93
244284	0.49	0.4	0.58	0.5	0.27	0.84
259977	1.5	1.35	1.64	1.48	1.06	2.02
202606	2.28	2.07	2.49	2.26	1.69	2.96
230697	1.77	1.6	1.94	1.82	1.36	2.39
243479	0.5	0.41	0.59	0.5	0.27	0.84
260149	1.36	1.22	1.51	1.37	0.96	1.89
207874	2.16	1.96	2.36	2.17	1.61	2.87
235279	1.99	1.81	2.17	2.04	1.54	2.64
243559	0.65	0.55	0.75	0.64	0.38	1.02
259587	2.03	1.85	2.2	2.04	1.53	2.65
213167	3.11	2.87	3.34	3.26	2.56	4.09
238938	3.24	3.01	3.46	3.29	2.65	4.03
744064	0.17	0.14	0.2	0.18	0.11	0.28
758576	0.15	0.13	0.18	0.16	0.09	0.26
773062	0.16	0.13	0.19	0.16	0.09	0.26
787195	0.16	0.13	0.19	0.16	0.09	0.26
801336	0.14	0.12	0.17	0.15	0.09	0.25

814638	0.16	0.14	0.19	0.17	0.1	0.27
828118	0.19	0.16	0.22	0.2	0.12	0.3
841067	0.19	0.16	0.22	0.2	0.12	0.3
853730	0.26	0.23	0.3	0.28	0.19	0.4
865101	0.24	0.21	0.28	0.25	0.17	0.37
876661	0.33	0.29	0.37	0.36	0.25	0.49
886564	0.27	0.23	0.3	0.28	0.19	0.4
896278	0.29	0.26	0.33	0.36	0.22	0.45
905437	0.37	0.33	0.41	0.4	0.29	0.54
914656	0.4	0.36	0.44	0.42	0.31	0.57
926688	0.47	0.42	0.51	0.49	0.37	0.64
937564	0.41	0.37	0.45	0.42	0.31	0.57
946781	0.68	0.63	0.73	0.71	0.56	0.89
955251	0.7	0.65	0.76	0.74	0.58	0.92
349186	0.21	0.17	0.27	0.21	0.1	0.37
394878	0.14	0.1	0.18	0.13	0.05	0.27
356748	0.22	0.17	0.27	0.21	0.1	0.37
401828	0.1	0.07	0.14	0.09	0.03	0.22
364415	0.23	0.18	0.29	0.23	0.12	0.4
408647	0.09	0.06	0.12	0.09	0.03	0.22
371793	0.19	0.15	0.24	0.19	0.09	0.35
415402	0.12	0.09	0.16	0.13	0.05	0.27
379214	0.18	0.14	0.22	0.19	0.09	0.35
422122	0.11	0.08	0.15	0.11	0.04	0.25
386119	0.2	0.16	0.25	0.21	0.1	0.37
428519	0.13	0.1	0.17	0.13	0.05	0.27
393075	0.23	0.19	0.29	0.25	0.13	0.42
435043	0.14	0.11	0.19	0.15	0.07	0.3
399769	0.25	0.2	0.3	0.26	0.14	0.44
441298	0.13	0.1	0.17	0.13	0.05	0.27
406566	0.36	0.31	0.42	0.4	0.25	0.61
447164	0.17	0.13	0.21	0.17	0.08	0.32
412463	0.3	0.25	0.36	0.32	0.19	0.51
452638	0.19	0.15	0.23	0.19	0.09	0.35
418329	0.4	0.34	0.47	0.45	0.29	0.67
458332	0.26	0.22	0.32	0.26	0.14	0.44
423761	0.32	0.27	0.38	0.34	0.2	0.54
462803	0.22	0.18	0.27	0.23	0.12	0.4
428800	0.34	0.29	0.4	0.38	0.23	0.58
467478	0.25	0.21	0.3	0.26	0.14	0.44
433740	0.43	0.37	0.5	0.47	0.31	0.7
471697	0.32	0.27	0.37	0.32	0.19	0.51
438477	0.48	0.42	0.55	0.51	0.34	0.74
476179	0.33	0.28	0.39	0.34	0.2	0.54
444456	0.56	0.5	0.64	0.6	0.41	0.85
482232	0.38	0.32	0.44	0.38	0.23	0.58
450067	0.51	0.45	0.58	0.55	0.37	0.79
487497	0.31	0.26	0.37	0.32	0.19	0.51
454117	0.81	0.73	0.9	0.85	0.62	1.14
492664	0.56	0.49	0.63	0.57	0.38	0.81
457801	0.85	0.77	0.94	0.91	0.67	1.2
497450	0.56	0.5	0.63	0.57	0.38	0.81
254153	0.09	0.06	0.14	0.11	0.02	0.31
200824	0.19	0.13	0.26	0.19	0.06	0.43
161251	0.29	0.21	0.39	0.3	0.12	0.63
170967	0.09	0.05	0.14	0.11	0.02	0.31
258054	0.11	0.07	0.16	0.11	0.02	0.31
204338	0.18	0.13	0.25	0.19	0.06	0.43
165276	0.2	0.14	0.28	0.22	0.07	0.51
173668	0.1	0.06	0.16	0.11	0.02	0.31
260886	0.11	0.07	0.16	0.11	0.02	0.31
208054	0.15	0.11	0.22	0.15	0.04	0.38
169161	0.31	0.23	0.4	0.3	0.12	0.63

176537	0.11	0.06	0.17	0.11	0.02	0.31
262821	0.12	0.08	0.17	0.11	0.02	0.31
212268	0.15	0.1	0.21	0.15	0.04	0.38
173285	0.34	0.26	0.44	0.35	0.15	0.69
179744	0.18	0.12	0.25	0.18	0.06	0.42
263633	0.09	0.06	0.14	0.11	0.02	0.31
217643	0.16	0.11	0.22	0.15	0.04	0.38
177080	0.33	0.25	0.42	0.35	0.15	0.69
182711	0.21	0.15	0.29	0.21	0.08	0.47
263387	0.12	0.08	0.17	0.11	0.02	0.31
223861	0.22	0.17	0.29	0.22	0.08	0.48
179658	0.41	0.32	0.52	0.43	0.21	0.8
186824	0.36	0.28	0.46	0.39	0.2	0.7
261791	0.1	0.06	0.14	0.11	0.02	0.31
229937	0.23	0.17	0.3	0.22	0.08	0.48
181370	0.36	0.28	0.46	0.35	0.15	0.69
192003	0.34	0.27	0.44	0.36	0.17	0.66
259077	0.15	0.1	0.2	0.14	0.04	0.37
236416	0.18	0.13	0.24	0.19	0.06	0.43
182957	0.48	0.39	0.59	0.48	0.24	0.86
198211	0.61	0.5	0.72	0.64	0.38	1.02
255483	0.12	0.08	0.17	0.11	0.02	0.31
242907	0.19	0.14	0.26	0.19	0.06	0.43
184864	0.41	0.32	0.51	0.39	0.18	0.74
203310	0.41	0.33	0.51	0.43	0.22	0.75
251840	0.12	0.08	0.17	0.11	0.02	0.31
247882	0.2	0.14	0.26	0.19	0.06	0.43
187859	0.43	0.32	0.54	0.43	0.21	0.8
208697	0.49	0.33	0.6	0.54	0.3	0.88
248218	0.12	0.08	0.17	0.11	0.02	0.31
252123	0.22	0.16	0.28	0.22	0.08	0.48
190282	0.55	0.45	0.67	0.57	0.3	0.97
214814	0.68	0.58	0.8	0.71	0.44	1.1
245219	0.11	0.07	0.16	0.11	0.02	0.31
255551	0.26	0.2	0.33	0.26	0.1	0.53
193998	0.54	0.44	0.66	0.52	0.27	0.91
219888	0.77	0.66	0.9	0.79	0.49	1.19
244229	0.12	0.08	0.18	0.11	0.02	0.31
258416	0.35	0.28	0.43	0.33	0.15	0.63
198052	0.58	0.48	0.7	0.57	0.3	0.97
225991	0.88	0.76	1.01	0.93	0.61	1.36
244284	0.1	0.07	0.15	0.11	0.02	0.31
259977	0.27	0.21	0.34	0.26	0.1	0.53
202606	0.53	0.43	0.64	0.52	0.27	0.91
230697	0.78	0.67	0.9	0.82	0.52	1.23
243479	0.32	0.25	0.4	0.82	0.15	0.61
260149	0.44	0.36	0.53	0.44	0.23	0.78
207874	0.7	0.59	0.82	0.7	0.4	1.13
235279	1.3	1.15	1.45	1.36	0.96	1.86
243559	0.15	0.11	0.21	0.14	0.04	0.37
259587	0.37	0.3	0.45	0.37	0.18	0.68
213167	0.68	0.57	0.8	0.7	0.4	1.13
238938	1.64	1.48	1.81	1.71	1.26	2.27
