# Supplementary material for: Incidence and prevalence of asthma, chronic obstructive pulmonary disease and interstitial lung disease between 2004 and 2023: harmonised analyses of longitudinal cohorts across England, Wales, South-East Scotland and Northern Ireland
Source: Thorax. 2025 Apr 8;80(7):e222699. doi: 10.1136/thorax-2024-222699 (PMC12322415; doi:10.1136/thorax-2024-222699)
Supplement: online supplemental file 3 [file thorax-80-7-s003.pdf]

nation	condition	date	numerator	denominator	rate_per_value	lower_per_value	upper_per_value
Scotland	Asthma	01-Jul-04	73306	674668	10.865493	10.791327	10.939994
Scotland	Asthma	01-Jul-05	77090	680365	11.330683	11.255457	11.406238
Scotland	Asthma	01-Jul-06	80608	690193	11.679052	11.603371	11.755055
Scotland	Asthma	01-Jul-07	83561	702148	11.900767	11.825116	11.976732
Scotland	Asthma	01-Jul-08	86349	712882	12.112664	12.037008	12.188627
Scotland	Asthma	01-Jul-09	88409	716467	12.339578	12.263504	12.415956
Scotland	Asthma	01-Jul-10	90580	723524	12.51928	12.443107	12.595755
Scotland	Asthma	01-Jul-11	92682	731319	12.673265	12.5971	12.749727
Scotland	Asthma	01-Jul-12	95037	743141	12.788555	12.712704	12.864697
Scotland	Asthma	01-Jul-13	97366	755740	12.883532	12.808076	12.959271
Scotland	Asthma	01-Jul-14	99730	767485	12.99439	12.91924	13.069821
Scotland	Asthma	01-Jul-15	101713	778036	13.073046	12.998213	13.148152
Scotland	Asthma	01-Jul-16	103539	790164	13.103482	13.029153	13.178082
Scotland	Asthma	01-Jul-17	105370	803017	13.121764	13.047988	13.195807
Scotland	Asthma	01-Jul-18	106985	813006	13.159189	13.085778	13.232863
Scotland	Asthma	01-Jul-19	108563	825732	13.147486	13.07467	13.220561
Scotland	Asthma	01-Jan-20	109442	833491	13.130556	13.058118	13.20325
Scotland	Asthma	01-Apr-20	109783	835888	13.133698	13.061357	13.206294
Scotland	Asthma	01-Jul-20	109632	835202	13.126406	13.054051	13.199015
Scotland	Asthma	01-Oct-20	109818	837793	13.108011	13.035812	13.180465
Scotland	Asthma	01-Jan-21	109853	840241	13.073987	13.001973	13.146255
Scotland	Asthma	01-Apr-21	109831	842545	13.035625	12.963799	13.107704
Scotland	Asthma	01-Jul-21	109783	844808	12.995024	12.923389	13.066912
Scotland	Asthma	01-Oct-21	109887	848153	12.956035	12.884634	13.027689
Scotland	Asthma	01-Jan-22	110186	853944	12.903188	12.832153	12.974475
Scotland	Asthma	01-Apr-22	110460	857430	12.882685	12.811842	12.953777
Scotland	Asthma	01-Jul-22	110658	859147	12.879985	12.809219	12.951
Scotland	Asthma	01-Oct-22	110779	864934	12.807798	12.737439	12.878407
Scotland	Asthma	01-Jan-23	110989	867892	12.788342	12.718149	12.858785
Scotland	COPD	01-Jul-04	11258	375690	2.9966195	2.9423308	3.0516348
Scotland	COPD	01-Jul-05	11984	380481	3.1496973	3.0944264	3.2056835
Scotland	COPD	01-Jul-06	12540	386794	3.2420359	3.1864407	3.2983327
Scotland	COPD	01-Jul-07	13123	392794	3.3409369	3.284956	3.3976076
Scotland	COPD	01-Jul-08	13593	398858	3.4079797	3.3518867	3.464751
Scotland	COPD	01-Jul-09	13817	399259	3.4606609	3.4041777	3.5178208
Scotland	COPD	01-Jul-10	14430	401655	3.5926354	3.535291	3.6506507
Scotland	COPD	01-Jul-11	15070	405902	3.7127187	3.6547604	3.7713392
Scotland	COPD	01-Jul-12	15898	411612	3.8623753	3.8037109	3.9216902
Scotland	COPD	01-Jul-13	16516	417231	3.9584787	3.8995152	4.0180826
Scotland	COPD	01-Jul-14	17174	424115	4.0493736	3.9902465	4.109129
Scotland	COPD	01-Jul-15	17829	430583	4.1406651	4.0813503	4.2005982
Scotland	COPD	01-Jul-16	18552	438100	4.2346497	4.1752071	4.2946982
Scotland	COPD	01-Jul-17	19193	445711	4.3061538	4.2467437	4.366158
Scotland	COPD	01-Jul-18	19748	451980	4.3692198	4.3098097	4.4292159
Scotland	COPD	01-Jul-19	20413	459306	4.444314	4.3848948	4.5043082
Scotland	COPD	01-Jan-20	20704	463398	4.4678655	4.4085593	4.5277424
Scotland	COPD	01-Apr-20	20787	464954	4.4707646	4.4115391	4.5305591
Scotland	COPD	01-Jul-20	20581	466379	4.4129348	4.3541665	4.47227
Scotland	COPD	01-Oct-20	20608	468570	4.3980622	4.3395257	4.4571633
Scotland	COPD	01-Jan-21	20537	470258	4.367177	4.3089428	4.4259748
Scotland	COPD	01-Apr-21	20404	472045	4.3224692	4.2646308	4.3808694
Scotland	COPD	01-Jul-21	20423	474214	4.3067055	4.2490997	4.3648701
Scotland	COPD	01-Oct-21	20410	476143	4.2865272	4.2291675	4.3444443
Scotland	COPD	01-Jan-22	20367	478081	4.2601566	4.2030821	4.3177867
Scotland	COPD	01-Apr-22	20375	480056	4.2442966	4.1874409	4.3017054
Scotland	COPD	01-Jul-22	20406	482696	4.2275057	4.1709127	4.2846484
Scotland	COPD	01-Oct-22	20408	485631	4.2023678	4.1461067	4.2591758
Scotland	COPD	01-Jan-23	20339	487523	4.1719055	4.1159496	4.2284074
Scotland	ILD	01-Jul-04	1022	314295	0.32517222	0.30556887	0.34570009
Scotland	ILD	01-Jul-05	1126	319290	0.35265747	0.33239061	0.37383345
Scotland	ILD	01-Jul-06	1218	325018	0.37474847	0.35403186	0.39635748
Scotland	ILD	01-Jul-07	1335	331414	0.40281942	0.3815386	0.42497456

Scotland	ILD	01-Jul-08	1410	338173	0.41694635	0.39550671	0.43924236
Scotland	ILD	01-Jul-09	1450	340222	0.42619231	0.40457866	0.44865692
Scotland	ILD	01-Jul-10	1515	343565	0.44096458	0.41908219	0.46368924
Scotland	ILD	01-Jul-11	1645	349255	0.47100255	0.44856396	0.49426889
Scotland	ILD	01-Jul-12	1723	355155	0.48514029	0.46255267	0.50854152
Scotland	ILD	01-Jul-13	1808	360445	0.50160217	0.4787991	0.52520651
Scotland	ILD	01-Jul-14	1880	366204	0.51337504	0.49048424	0.53705418
Scotland	ILD	01-Jul-15	1943	371067	0.52362514	0.50065583	0.54737216
Scotland	ILD	01-Jul-16	2001	376418	0.53158987	0.50860882	0.55533743
Scotland	ILD	01-Jul-17	2098	381722	0.54961467	0.52640623	0.57357848
Scotland	ILD	01-Jul-18	2168	386157	0.56142968	0.53810549	0.58550036
Scotland	ILD	01-Jul-19	2249	392336	0.57323313	0.54984826	0.5973525
Scotland	ILD	01-Jan-20	2316	395777	0.58517802	0.56165153	0.60943234
Scotland	ILD	01-Apr-20	2322	397045	0.58482039	0.56133831	0.60902792
Scotland	ILD	01-Jul-20	2319	398237	0.58231658	0.55891979	0.60643673
Scotland	ILD	01-Oct-20	2346	400166	0.58625668	0.56283665	0.61039656
Scotland	ILD	01-Jan-21	2355	401584	0.58642775	0.5630452	0.61052752
Scotland	ILD	01-Apr-21	2360	403055	0.58552802	0.56220579	0.6095649
Scotland	ILD	01-Jul-21	2374	404836	0.58641028	0.56312132	0.61041075
Scotland	ILD	01-Oct-21	2372	406309	0.58379215	0.56059712	0.60769606
Scotland	ILD	01-Jan-22	2360	407742	0.5787974	0.55574244	0.60255879
Scotland	ILD	01-Apr-22	2377	409342	0.58068806	0.55764008	0.60443968
Scotland	ILD	01-Jul-22	2390	411378	0.58097416	0.55797708	0.60467154
Scotland	ILD	01-Oct-22	2409	413719	0.58227926	0.55932081	0.60593396
Scotland	ILD	01-Jan-23	2382	415242	0.57364142	0.55089599	0.59708059
Wales	Asthma	01-Jul-04	325254	2649324	12.276868	12.237373	12.316444
Wales	Asthma	01-Jul-05	352732	2869656	12.291787	12.253819	12.329832
Wales	Asthma	01-Jul-06	369433	2962991	12.468246	12.43065	12.505915
Wales	Asthma	01-Jul-07	380484	3013565	12.62571	12.58823	12.663263
Wales	Asthma	01-Jul-08	389243	3041525	12.797626	12.760102	12.835221
Wales	Asthma	01-Jul-09	397761	3058727	13.004135	12.96646	13.041881
Wales	Asthma	01-Jul-10	406130	3065626	13.247865	13.209934	13.285866
Wales	Asthma	01-Jul-11	412941	3067907	13.460023	13.421851	13.498265
Wales	Asthma	01-Jul-12	419152	3062076	13.688491	13.65001	13.727041
Wales	Asthma	01-Jul-13	424770	3043359	13.957275	13.91836	13.996261
Wales	Asthma	01-Jul-14	429621	3008673	14.279418	14.239903	14.319002
Wales	Asthma	01-Jul-15	432825	2953553	14.654384	14.61407	14.694768
Wales	Asthma	01-Jul-16	440698	2918586	15.09971	15.05865	15.140839
Wales	Asthma	01-Jul-17	442984	2865386	15.459837	15.417995	15.501748
Wales	Asthma	01-Jul-18	441667	2782727	15.871733	15.828817	15.91472
Wales	Asthma	01-Jul-19	436817	2659058	16.427509	16.382992	16.472099
Wales	COPD	01-Jul-04	35782	1274444	2.8076558	2.7790439	2.8364823
Wales	COPD	01-Jul-05	42863	1365767	3.1383832	3.109205	3.1677599
Wales	COPD	01-Jul-06	48734	1412753	3.4495769	3.4195433	3.4798014
Wales	COPD	01-Jul-07	54410	1445251	3.764744	3.7337699	3.7959034
Wales	COPD	01-Jul-08	58433	1468452	3.9792244	3.9476657	4.0109649
Wales	COPD	01-Jul-09	62235	1488862	4.180038	4.1479468	4.2123079
Wales	COPD	01-Jul-10	66972	1504086	4.4526711	4.4197621	4.4857554
Wales	COPD	01-Jul-11	70155	1518957	4.6186299	4.5853052	4.6521277
Wales	COPD	01-Jul-12	72874	1528848	4.7665954	4.7328758	4.8004866
Wales	COPD	01-Jul-13	74768	1531432	4.8822279	4.8481503	4.9164762
Wales	COPD	01-Jul-14	76956	1528409	5.0350399	5.0004258	5.0698242
Wales	COPD	01-Jul-15	78220	1512167	5.172709	5.1374621	5.208128
Wales	COPD	01-Jul-16	80091	1503793	5.3259325	5.2900958	5.3619413
Wales	COPD	01-Jul-17	81404	1488597	5.4685049	5.4320335	5.5051494
Wales	COPD	01-Jul-18	82395	1462818	5.6326213	5.595314	5.6701045
Wales	COPD	01-Jul-19	83791	1432080	5.8509998	5.812614	5.8895645
Wales	ILD	01-Jul-04	5095	1274444	0.39978218	0.38890037	0.41089037
Wales	ILD	01-Jul-05	5515	1365767	0.40380239	0.39323539	0.41458058
Wales	ILD	01-Jul-06	5785	1412753	0.40948418	0.3990202	0.42015225
Wales	ILD	01-Jul-07	6020	1445251	0.41653663	0.40610132	0.42717141
Wales	ILD	01-Jul-08	6225	1468452	0.4239158	0.41347125	0.4345566
Wales	ILD	01-Jul-09	6366	1488862	0.42757487	0.41715696	0.43818635

Wales	ILD	01-Jul-10	6538	1504086	0.43468258	0.42423126	0.44532546
Wales	ILD	01-Jul-11	6695	1518957	0.44076297	0.43029001	0.45142558
Wales	ILD	01-Jul-12	6874	1528848	0.44961959	0.43907586	0.46035171
Wales	ILD	01-Jul-13	7080	1531432	0.4623124	0.45162964	0.47318316
Wales	ILD	01-Jul-14	7176	1528409	0.46950784	0.45873159	0.48047242
Wales	ILD	01-Jul-15	7349	1512167	0.4859913	0.47496894	0.49720395
Wales	ILD	01-Jul-16	7557	1503793	0.50252926	0.49128979	0.51396
Wales	ILD	01-Jul-17	7737	1488597	0.51975113	0.50826275	0.53143269
Wales	ILD	01-Jul-18	7924	1462818	0.54169416	0.52986336	0.55372143
Wales	ILD	01-Jul-19	8070	1432080	0.56351602	0.55132115	0.57591146
CPRD	COPD	01-Jan-04	106102	4931757	2.1514037	2.1386163	2.164247
CPRD	COPD	01-Jan-05	131472	5007318	2.6255972	2.6116097	2.6396396
CPRD	COPD	01-Jan-06	149708	5098463	2.9363358	2.9216988	2.9510267
CPRD	COPD	01-Jan-07	164221	5196224	3.1603911	3.1453657	3.1754687
CPRD	COPD	01-Jan-08	176011	5283113	3.3315775	3.3162909	3.3469152
CPRD	COPD	01-Jan-09	186810	5366458	3.4810669	3.4655745	3.4966099
CPRD	COPD	01-Jan-10	199212	5450907	3.6546578	3.6389208	3.6704443
CPRD	COPD	01-Jan-11	210648	5527538	3.8108828	3.7949371	3.8268771
CPRD	COPD	01-Jan-12	220688	5617782	3.9283831	3.9123333	3.9444804
CPRD	COPD	01-Jan-13	229851	5655872	4.0639358	4.0476775	4.0802407
CPRD	COPD	01-Jan-14	238546	5756505	4.1439381	4.1276712	4.1602511
CPRD	COPD	01-Jan-15	246787	5876966	4.1992245	4.183023	4.2154717
CPRD	COPD	01-Jan-16	255276	5985438	4.2649512	4.2487769	4.2811694
CPRD	COPD	01-Jan-17	264562	6094445	4.3410354	4.3248701	4.3572435
CPRD	COPD	01-Jan-18	272987	6199537	4.4033451	4.387208	4.4195247
CPRD	COPD	01-Jan-19	278703	6269084	4.4456735	4.4295526	4.4618363
CPRD	Asthma	01-Jan-04	579795	10006581	5.794137	5.7796688	5.8086305
CPRD	Asthma	01-Jan-05	672688	10155856	6.6236463	6.6083589	6.6389589
CPRD	Asthma	01-Jan-06	767280	10338561	7.421536	7.4055648	7.4375305
CPRD	Asthma	01-Jan-07	835487	10527307	7.936379	7.9200578	7.952724
CPRD	Asthma	01-Jan-08	886458	10710915	8.2762117	8.2597179	8.2927275
CPRD	Asthma	01-Jan-09	933528	10881345	8.5791597	8.5625267	8.5958157
CPRD	Asthma	01-Jan-10	974363	11047964	8.8193893	8.8026743	8.8361263
CPRD	Asthma	01-Jan-11	1008823	11198241	9.0087633	8.9920006	9.025547
CPRD	Asthma	01-Jan-12	1040902	11397743	9.1325274	9.1158094	9.1492662
CPRD	Asthma	01-Jan-13	1074449	11488829	9.3521194	9.335289	9.3689699
CPRD	Asthma	01-Jan-14	1106485	11705561	9.4526443	9.4358902	9.4694176
CPRD	Asthma	01-Jan-15	1141328	11973599	9.5320377	9.5154104	9.5486851
CPRD	Asthma	01-Jan-16	1173518	12250589	9.579278	9.5628033	9.5957727
CPRD	Asthma	01-Jan-17	1206343	12542202	9.6182709	9.6019592	9.6346016
CPRD	Asthma	01-Jan-18	1234726	12800212	9.6461372	9.6299696	9.662323
CPRD	Asthma	01-Jan-19	1248916	12949670	9.6443844	9.6283121	9.6604757
CPRD	ILD	01-Jan-04	4134	4931757	0.083824083	0.08128912	0.086417936
CPRD	ILD	01-Jan-05	5191	5007318	0.10366827	0.10086851	0.10652598
CPRD	ILD	01-Jan-06	7070	5098463	0.13866924	0.13545768	0.14193764
CPRD	ILD	01-Jan-07	8978	5196224	0.17277931	0.1692266	0.17638773
CPRD	ILD	01-Jan-08	10610	5283113	0.20082857	0.19702891	0.20468296
CPRD	ILD	01-Jan-09	12120	5366458	0.22584729	0.22184859	0.22989984
CPRD	ILD	01-Jan-10	13601	5450907	0.24951811	0.24534723	0.25374198
CPRD	ILD	01-Jan-11	14820	5527538	0.26811212	0.26381835	0.27245814
CPRD	ILD	01-Jan-12	16101	5617782	0.28660777	0.28220385	0.29106304
CPRD	ILD	01-Jan-13	17366	5655872	0.30704373	0.30250069	0.31163773
CPRD	ILD	01-Jan-14	18715	5756505	0.32511047	0.32047653	0.3297945
CPRD	ILD	01-Jan-15	19879	5876966	0.33825275	0.33357459	0.34297997
CPRD	ILD	01-Jan-16	21327	5985438	0.35631478	0.3515569	0.36112079
CPRD	ILD	01-Jan-17	22733	6094445	0.37301183	0.36818737	0.37788352
CPRD	ILD	01-Jan-18	24157	6199537	0.38965812	0.38476911	0.39459357
CPRD	ILD	01-Jan-19	25229	6269084	0.40243518	0.39749429	0.40742201
HBS	asthma	2011	126363	1814318	6.08	5.97	6.19
HBS	asthma	2012	130365	1824603	6.26	6.15	6.37
HBS	asthma	2013	133527	1831677	6.41	6.3	6.53
HBS	asthma	2014	136297	1843186	6.54	6.43	6.66
HBS	asthma	2015	138389	1854943	6.64	6.53	6.75

HBS	asthma	2016	141528	1866042	6.79	6.67	6.9
HBS	asthma	2017	143468	1875178	6.9	6.78	7.01
HBS	asthma	2018	144472	1886259	6.96	6.84	7.08
HBS	asthma	2019	144245	1898519	6.97	6.86	7.09
HBS	asthma	2020	140179	1900523	6.83	6.71	6.94
HBS	asthma	2021	133430	1904564	6.55	6.44	6.67
HBS	asthma	2022	127477	1910543	6.29	6.18	6.4
HBS	COPD	2011	14030	841067	1.64	1.57	1.72
HBS	COPD	2012	15818	853730	1.83	1.75	1.91
HBS	COPD	2013	18142	865101	2.07	1.98	2.15
HBS	COPD	2014	19334	876661	2.18	2.1	2.27
HBS	COPD	2015	21226	886564	2.37	2.28	2.47
HBS	COPD	2016	23362	896278	2.59	2.49	2.69
HBS	COPD	2017	25267	905437	2.77	2.67	2.87
HBS	COPD	2018	26908	914656	2.92	2.82	3.03
HBS	COPD	2019	28261	926688	3.03	2.93	3.14
HBS	COPD	2020	27207	937564	2.88	2.78	2.98
HBS	COPD	2021	25750	946781	2.7	2.6	2.8
HBS	COPD	2022	24688	955251	2.56	2.46	2.66
HBS	ILD	2011	1454	841067	0.17	0.15	0.2
HBS	ILD	2012	1626	853730	0.19	0.16	0.22
HBS	ILD	2013	1775	865101	0.21	0.18	0.23
HBS	ILD	2014	2003	876661	0.23	0.2	0.26
HBS	ILD	2015	2133	886564	0.24	0.22	0.28
HBS	ILD	2016	2309	896278	0.26	0.23	0.29
HBS	ILD	2017	2538	905437	0.29	0.26	0.32
HBS	ILD	2018	2739	914656	0.31	0.27	0.34
HBS	ILD	2019	2952	926688	0.33	0.29	0.36
HBS	ILD	2020	3042	937564	0.33	0.3	0.37
HBS	ILD	2021	3251	946781	0.35	0.32	0.39
HBS	ILD	2022	3357	955251	0.36	0.32	0.4
